# A high-density genetic map of cucumber derived from Specific Length Amplified Fragment sequencing (SLAF-seq)

**DOI:** 10.3389/fpls.2014.00768

**Published:** 2015-01-07

**Authors:** Xuewen Xu, Ruixue Xu, Biyun Zhu, Ting Yu, Wenqin Qu, Lu Lu, Qiang Xu, Xiaohua Qi, Xuehao Chen

**Affiliations:** Department of Horticulture, School of Horticulture and Plant Protection, Yangzhou UniversityYangzhou, China

**Keywords:** cucumber, F_2_ population, SLAF-seq, SNP, genetic map

## Abstract

High-density genetic map provides an essential framework for accurate and efficient genome assembly and QTL fine mapping. Construction of high-density genetic maps appears more feasible since the advent of next-generation sequencing (NGS), which eases SNP discovery and high-throughput genotyping of large population. In this research, a high-density genetic map of cucumber (*Cucumis sativus* L.) was successfully constructed across an F_2_ population by a recently developed Specific Length Amplified Fragment sequencing (SLAF-seq) method. In total, 18.69 GB of data containing 93,460,000 paired-end reads were obtained after preprocessing. The average sequencing depth was 44.92 in the D8 (female parent), 42.16 in the Jin5-508 (male parent), and 5.01 in each progeny. 79,092 high-quality SLAFs were detected, of which 6784 SLAFs were polymorphic, and 1892 of the polymorphic markers met the requirements for constructing genetic map. The genetic map spanned 845.87 cm with an average genetic distance of 0.45 cm. It is a reliable linkage map for fine mapping and molecular breeding of cucumber for its high marker density and well-ordered markers.

## Introduction

Cucumber (*Cucumis sativus* L.), a diploid species (2n = 2x =14), is an important crop all over the world ranking fourth in quantity of world vegetable production after tomato, cabbage and onion (FAO STAT 2011, http://faostat.fao.org). Great progresses have been made on the study of cucumber genetics and gene/QTL mapping in the past decades for its economic importance and research values. For example, Fazio et al. (2003) developed a cucumber genetic map spanned 706 cm with 27 AFLPs, 62 RAPD, 14 SSR, 24 SCAR, one SNP, and three MTM molecular markers. Yuan et al. (2008) constructed another map spanned 1005.8 cm included 206 SRAPs, 22 SSRs, 25 SCARs, one STS and three MTM. Ren et al. (2009) developed a linkage map with 995 SSR markers spanning 572.9 cm. Miao et al. (2011) developed a linkage map of cultivated cucumber with 248 SSR into seven linkage groups spanning 711.9 cm, with a mean marker interval of 2.8 cm. Li et al. (2011) constructed a framework genetic map consisting of 187 SSR loci in seven linkage groups (chromosomes) covering 527.5 cm, and subsequently another five SNPs-derived markers were successfully added in this map (Li et al., 2013). Zhang et al. (2012) constructed a consensus map consisted of 1152 SSR, 192 SRAP, 21 SCAR and one STS locus spanned 700.5 cm. Yang et al. (2013) constructed a map spanning 730.0 cm in seven linkage groups by integrating three component maps with a bin-mapping strategy, which has been considered to be the densest genetic maps of cucumber so far. Despite of significant improvements in map construction, marker density of these maps are still far from being satisfactory for many molecular marker-based applications such as marker-assisted breeding, map-based gene cloning or assembly of a more complete cucumber genome (Yang et al., 2013).

Molecular markers and genetic maps provide important foundations for quantitative trait loci (QTL) mapping. RAPD and AFLP markers are dominant and cannot be transferred readily to other populations. SSR marker has played a more important role than other molecular markers in constructing of high-density genetic map in recent decade (Gerber et al., 2000). However, the genotyping of SSR markers are time- and cost-consuming, despite of it is one of the most reliable markers for genetic map construction (Woodhead et al., 2005). Even though hundreds of QTLs have been reported in cucumber, most of them have not been cloned. There is a growing need for generating new maps with more informative and transferable markers that are amenable to large-scale genotyping, and the marker should be high-throughput and cost effective to provide high reliable genotyping data. SNP (single nucleotide polymorphism) is considered more useful than many other conventional markers such as AFLP, RAPD, and SSR, because SNP is the most common type of genetic variation and stabile in most genomes (Fusari et al., 2008; Wang et al., 2012). SNP is highly prevalent, usually biallelic and co-dominant in nature, sequence-tagged, and amenable to development of low-cost multiplexed marker assays that can provide sufficiently dense genome coverage for the dissection of complex traits (Leonforte et al., 2013). Application of next generation sequencing technologies and high-throughput genotyping facilities enabled obtaining of thousands of SNPs throughout the genome for high-density genetic maps construction. SNPs have become the first choice for marker development in most species for genome-wide association studies (GWAS), phylogenetic analyses, marker-assisted selection, bulked segregant analysis (BSA) and genomic selection (Liu et al., 2013). The development of high-throughput SNP markers in cucumber will benefits researchers in gene cloning, QTL mapping and genome assembly for its speedy, inexpensive genotyping.

Specific Length Amplified Fragment sequencing (SLAF-seq) was developed based on high-throughput sequencing technology. It allows researchers to design the experimental system through bioinformatics and screen for fragments of a specific length from the constructed SLAF-seq library. SLAF-seq technology has several obvious advantages, such as high-throughput, high accuracy, low cost and short cycle, which enable its sequencing results to be directly used for molecular markers development. This technology has been reported for haplotype mapping, genetic mapping, and polymorphism mapping. It can also provide an important basis for molecular breeding, system evolution and germplasm resource identification for multiple purposes, but in particular will facilitate gene mapping studies (Chen et al., 2013; Liu et al., 2013). In this study, SLAF-seq was employed for rapid mass discovery of SNP markers and high-density genetic map construction in cucumber.

## Materials and methods

### Plant material and DNA extraction

An F_2_ mapping population consisted of 102 individuals derived from a cross of “D8” (female parent, dwaft) and “Jin5-508” (male parent, sprawl). Seedlings of progeny and parents were planted in the experimental farm of the Department of Horticulture in Yangzhou University. Young healthy leaves from the two parents and F_2_ individuals were collected, frozen in liquid nitrogen, and used for DNA extraction. Total genomic DNA was prepared from each plant by CTAB method (Doyle and Doyle, 1987) with some modification to the components of the CTAB buffer (8.18 g sodium chloride and 2 g CTAB in a total volume of 100 ml of 20 mM EDTA, 100 mM Tris, pH 8.0). DNA concentrations and qualities were estimated with a Biophotometer Plus (Expander, Germany) and by electrophoresis in 1.0% agarose gels.

### Genotyping

The procedure was performed as described by Sun et al. (Sun et al., 2013) with small modifications. In brief, a pilot SLAF experiment was performed to establish conditions to optimize SLAF yield, avoid repetitive SLAFs, and obtain an even distribution of SLAFs for maximum SLAF-seq efficiency. Based on the result of the pilot experiment, the SLAF library was constructed as following. Genomic DNA from each sample was first incubated with *Hae*III and *Rsa*I (NEB, Ipswich, MA, USA), T4 DNA ligase (NEB), ATP (NEB), and *Rsa*I (NEB) adapter at 37°C. Then restriction–ligation reaction solutions were diluted and mixed with dNTP, Taq DNA polymerase (NEB) and primer containing barcode 1 for PCR reactions. The E.Z.N.A.® Cycle Pure Kit (Omega, London, UK) were used to purify the PCR products. The purified PCR products were pooled and incubated at 37°C with *Mse*I, T4 DNA ligase, ATP, and Solexa adapter. After incubation, the reaction products were then purified using a Quick Spin column (Qiagen, Hilden, Germany), and electrophoresed on a 2% agarose gel. After samples were gel purified, DNA fragments with indices and adaptors (SLAFs) of 314–414 bp were excised and diluted for pair-end sequencing on an Illumina High-seq 2500 sequencing platform according to the Illumina sample preparation guide (Illumina, Inc.; San Diego, CA, US) at Beijing Biomarker Technologies Corporation (http://www.biomarker.com.cn).

According to the barcode sequences, raw reads were demultiplexed to individuals. Real-time monitoring was performed for each cycle during sequencing, quality scores lower than 30 (means a quality score of 20, indicating a 0.1% chance of an error, and thus 99.9% confidence quality score, 30) were filtered out. After barcodes were trimmed from reads, reads of 100 bases from the same samples were mapped onto the cucumber genome sequence (http://www.icugi.org, version 2) using SOAPdenovo2 software (Luo et al., 2012). All sequence mapped to the same position were defined as a SLAF loci. In each of the SLAF, polymorphic loci between the parents were found, and most of them are SNPs. All polymorphic SLAFs loci were genotyped with consistency in the offspring and parental SNP loci. A SLAF which has less than three SNP and average depths of each sample above three was used as high quality SLAF markers, and were used to construct high-density genetic map with parental homozygous.

### Linkage map construction and evaluation

High Map software (Liu et al., 2014) was used to order SLAF markers and correct genotyping errors within linkage groups (LGs). All high quality of SLAFs markers were allocated into seven LGs based on their locations on chromosomes. In order to correct genotyping errors and deletion caused by next generation sequencing (NGS), SMOOTH algorithms (van Os et al., 2005) and Detaily MSTmap (Wu et al., 2008) were applied. SMOOTH algorithms were used to correct genotyping errors following marker ordering. Detaily MSTmap algorithm was used to order SLAFs markers. The procedures of constructing linkage groups were described as followings: markers were aligned by their location on chromosomes and corrected by SMOOTH algorithm firstly, and then MSTmap was introduced to order the map, after that SMOOTH algorithm was again used to correct the new ordered genotypes. As four or more cycles, the final high-quality map was obtained. Kosambi mapping function was used to calculate map distance in cm (Kosambi, 1944). Haplotype map and heat map were used to evaluate the quality of constructed linkage map. The detailed approaches/methods were according to Liu et al. (2013). “draw_haplotype-map.pl” was used to construct haplotype map, and “draw_heatmap.pl” was used to construct heat map, both of which were programmed by Beijing Biomarker Technologies Corporation, and can be download at http://highmap.biomarker.com.cn/.

### Validation of genotyping results based on SLAF-seq

A total of 14 mapped SLAF markers were randomly selected from seven linkage groups for genotyping validation. For SNP genotyping, SNP-based polymerase chain reaction (PCR) amplification of derived CAPS (dCAPS) (Michaels and Amasino, 1998) markers were developed. Primers for dCAPS markers were designed with Primer Premier 5.0 (http://www.premierbiosoft.com/) and dCAPS Finder 2.0 (http://helix.wustl.edu/dcaps/dcaps.html) (Neff et al., 2002), respectively. Each PCR contained 25 ng template DNA, 0.5 μM each of forward and reverse primers, 0.2 mM dNTPmix, 0.5 unit of Taq DNA polymerase and 1× PCR buffer (Takara, China) in a total volume of 10.0 μl. For genotyping with dCAPS markers, after performing specific primer-based PCR, the appropriate restriction enzyme was added to the PCR reaction and incubated for 2 h at the temperatures in accordance with manufacturer's instructions. Digested products were then separated in 9% polyacrylamide gel and visualized with silver staining as described above. Information of the 14 markers developed from the present study including primer sequences is provided in Table S1.

## Results

### Analysis of SLAF-seq data and SLAF markers

**Figure 1:**
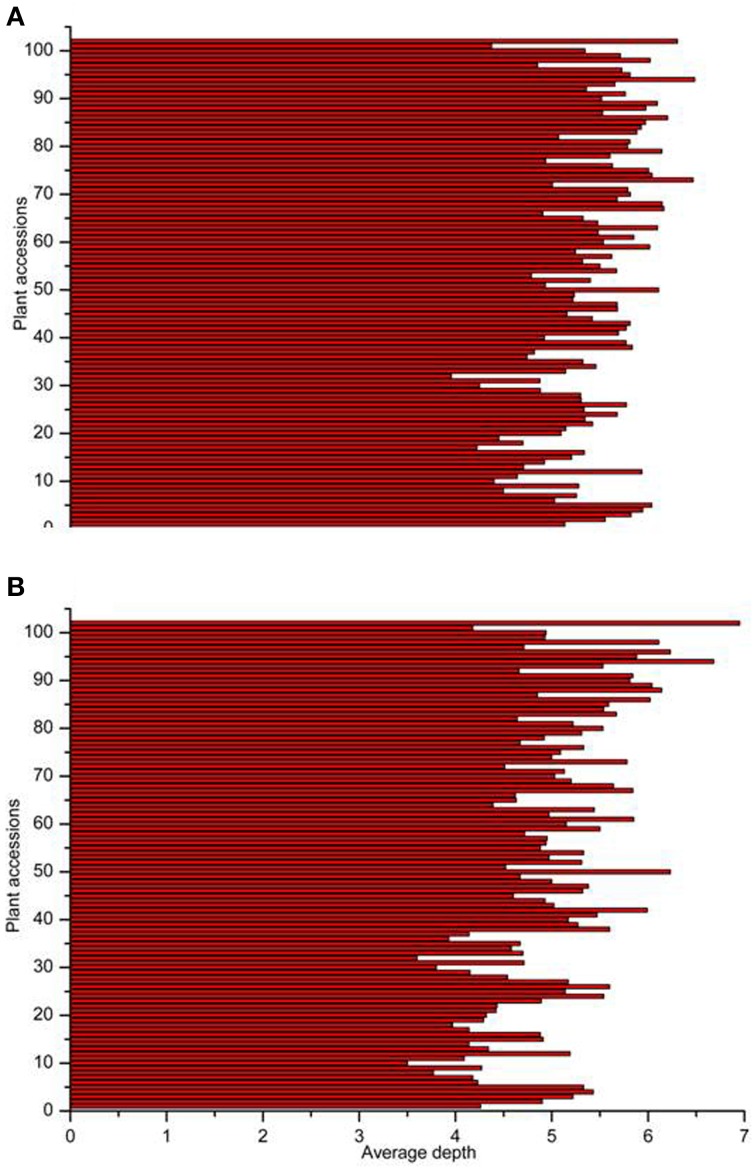
**Number of markers and average sequencing depths of F_2_ population**. The x-axes indicate the number of markers **(A)** and average depths **(B)**, the y-axes indicate individual F_2_ plant accessions.

**Figure 2:**
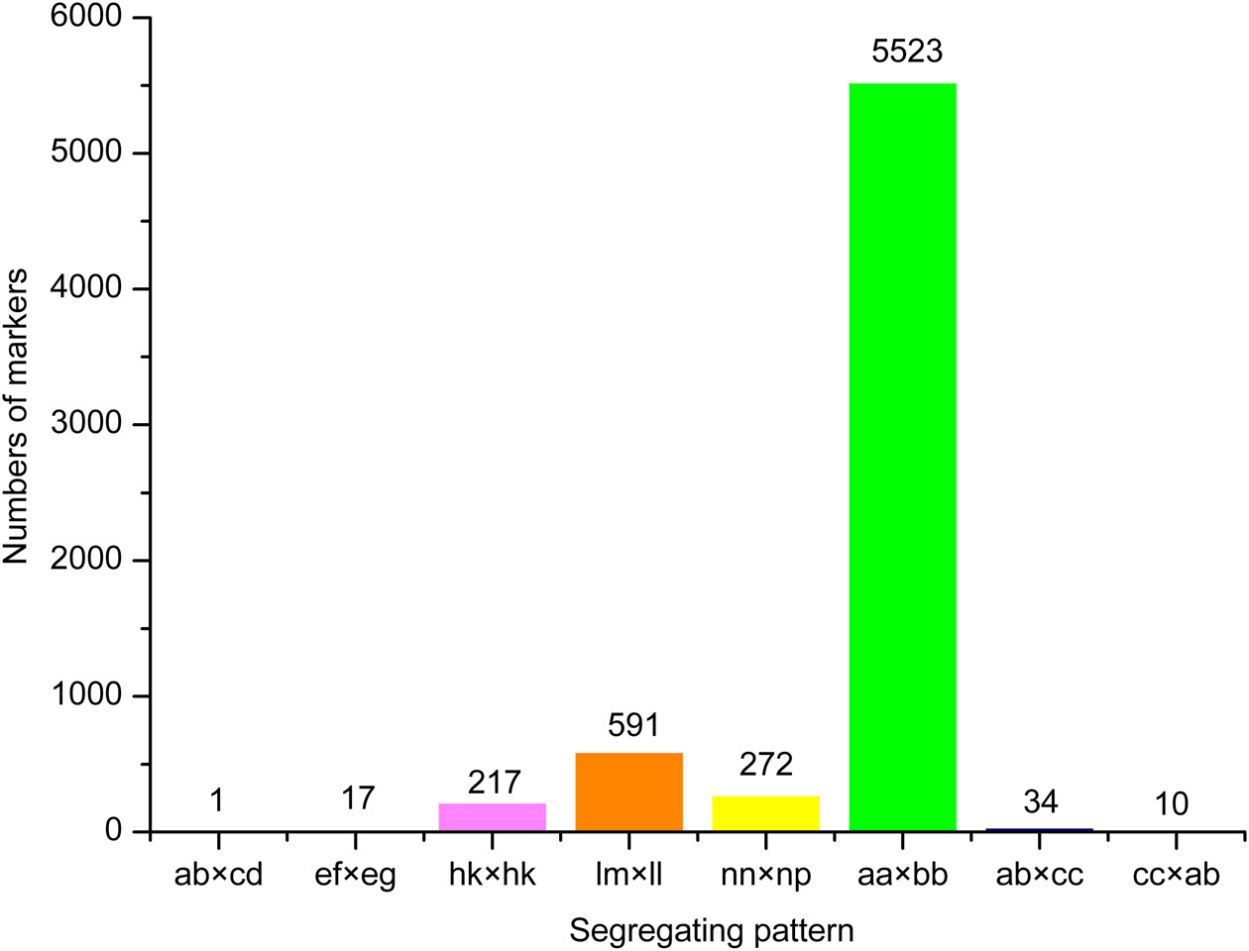
**Number of markers in each of eight segregation patterns**.

18.69 GB of data (93.46 M reads) was obtained with each read being ~80 bp in length after SLAF library constructed and high-throughput sequencing. Among them, 82.80% bases were of high-quality with quality scores of at least 30. The SLAFs numbers in the male and female parents were 75,655 and 70,819, respectively (Table 1). The read numbers for SLAFs were 3,189,643 and 3,180,935 in the male and female parents, respectively. The average coverage was 42.16-fold in the male parent and 44.92-fold in the female parent. In the F_2_ population, the average numbers of SLAF markers was 47,830. The average read numbers for SLAFs was 239,677, and the average coverage was 5.01-fold (Figure 1). The average coverage in parent and offsprings revealed that the sequencing results are reliable for marker exploring. Among the 79,092 high-quality SLAFs that were detected, 6784 were polymorphic with a polymorphism rate of 8.60% (Table 2). Of the 6784 polymorphic SLAFs, 6665 were classified into eight segregation patterns (Figure 2). The number of SLAF markers per chromosome ranged from 570 to 1395 (Table 2). 5523 of the 6784 polymorphic SLAFs with aa × bb segregation pattern in F_2_ population were selected to construct a linkage map as result of the F_2_ population is obtained by selfing the F_1_ from a cross between two fully homozygous parents with genotype aa or bb. With the criteria of segregation distortion (*P* < 0.05), sequence depth in parents more than 10-fold of parental, and the marker missing data less than 30% in F_2_ population, 1892 markers were finally used for construction of genetic map.

**Table 1:** **Summary of marker depths**.

**Samples**	**Marker numbers**	**Total depth**	**Average depth**
Jin5-508	75,655	3,189,643	42.16
D8	70,819	3,180,935	44.92
Offspring	47,830	239,677	5.01

**Table 2:** **SLAF markers numbers on each chromosome**.

**Chromosome ID**	**Length of the Chromosome**	**SLAF numbers**	**Ration of SLAF on chromosome**	**Polymorphic SLAF**	**Ration of Polymorphic SLAF on chromosome**	**Ration of Polymorphic SLAF on detected SLAF**
Chr1	29,972,036	12,062	0.0402%	1209	0.0040%	10.02%
Chr2	23,828,421	9463	0.0397%	570	0.0024%	6.02%
Chr3	40,905,010	16,108	0.0394%	1395	0.0034%	8.66%
Chr4	24,086,726	9914	0.0412%	1191	0.0049%	12.01%
Chr5	28,814,066	11,555	0.0401%	632	0.0022%	5.47%
Chr6	29,896,516	11,951	0.0400%	1032	0.0035%	8.64%
Chr7	19,768,912	8039	0.0407%	755	0.0038%	9.39%
Total	197,271,687	79,092	0.0402%	6784	0.0035%	8.60%

### Main characteristics of the genetic map

**Figure 3:**
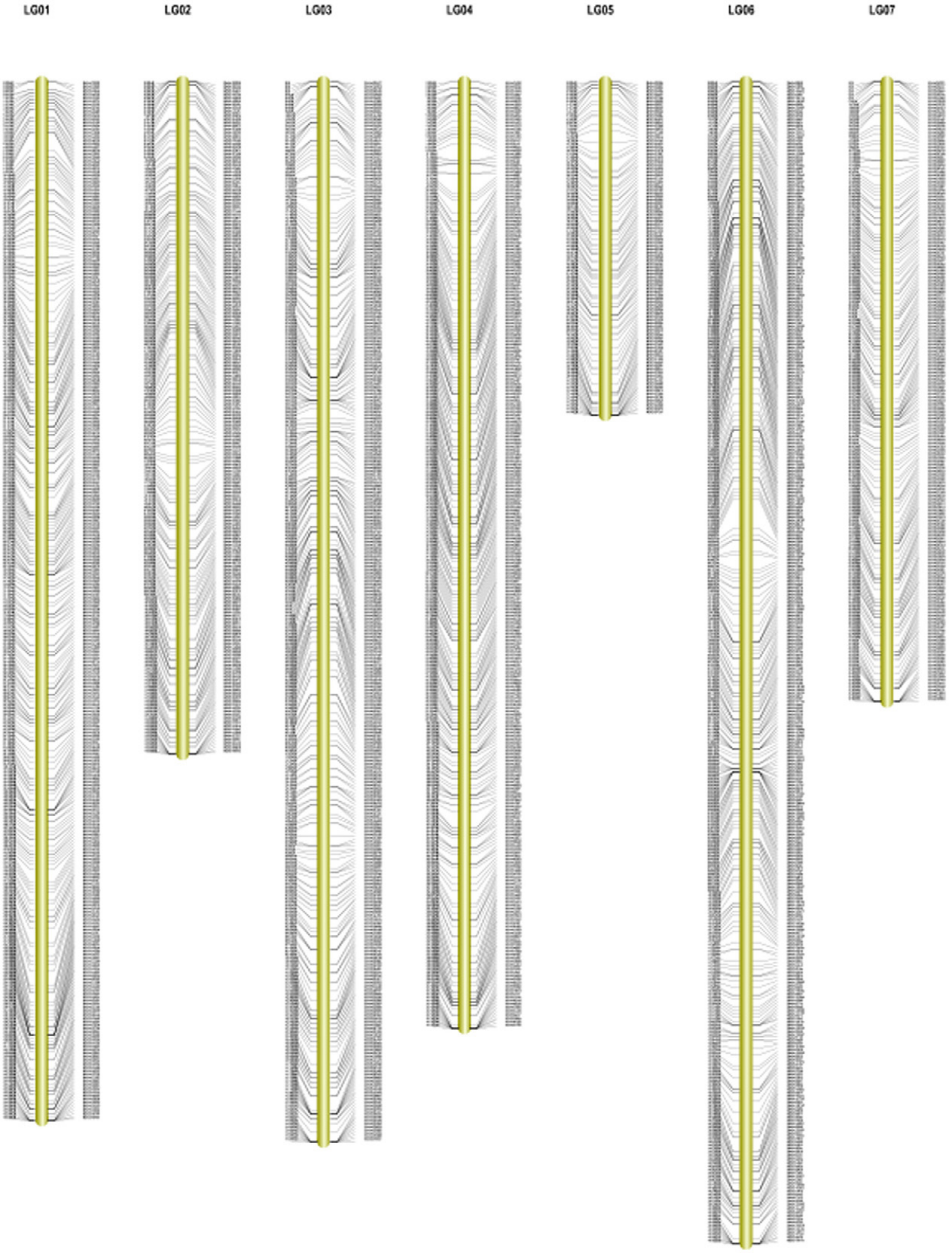
**High-density genetic map of cucumber**.

In total, 1892 markers were mapped onto the seven LGs, including the SLAF markers physical positions. The map spanned a total of 845.87 cm with an average inter-marker distance of 0.45 cm (Figure 3). On average, each LG contained 270 markers that spanned an average of 120.84 cm. The length of the LGs ranged from 77.44 cm (Chr5) to 151.58 cm (Chr6). The largest LG was LG6, contained 377 markers with a genetic length of 151.58 cm, and an average marker density of 0.40 cm. The smallest LG was LG5, only harbored 108 markers, a genetic length of 77.44 cm with 0.72 cm average inter-marker distance. The largest gap on this map was only 3.74 cm located in LG6 (Table 3). The LGs were numbered according to the chromosome numbers. This map is the densest genetic map to date for cucumber up to now.

**Table 3:** **Description on basic characteristics of the seven linkage groups**.

**Linkage group ID**	**Marker number**	**Total distance (cm)**	**Minimum distance (cm)**	**Maximum distance (cm)**	**Average distance(cm)**	**Gaps ≤ 5**
Chr1	337	144.81	0	2.45	0.43	100%
Chr2	218	118.23	0	2.66	0.54	100%
Chr3	344	114.46	0	1.92	0.33	100%
Chr4	307	141.46	0	2.94	0.46	100%
Chr5	108	77.44	0	2.66	0.72	100%
Chr6	377	151.58	0	3.74	0.40	100%
Chr7	201	97.89	0	1.96	0.49	100%
Total	1892	845.87	0	2.82	0.45	100%

*“Gap ≤ 5” indicated the percentages of gaps in which the distance between adjacent markers was smaller than 5 cm*.

### Evaluation of the genetic map

In order to validate the results of SNPs mined and genotyped from parents and segregating mapping individuals using SLAF-seq, 14 pairs of mapped SLAF markers were randomly selected from seven linkage groups for genotyping validation. The genotyping results obtained from SNP-based PCR amplification and restriction endonuclease reaction are complete correlation with the data obtained by SLAF-seq (PCC value = 1), suggesting the SNPs mined from parents are accurate, and genotyping results in the 102 mapping individuals are reliable for map construction (Table S2).

**Figure 4:**
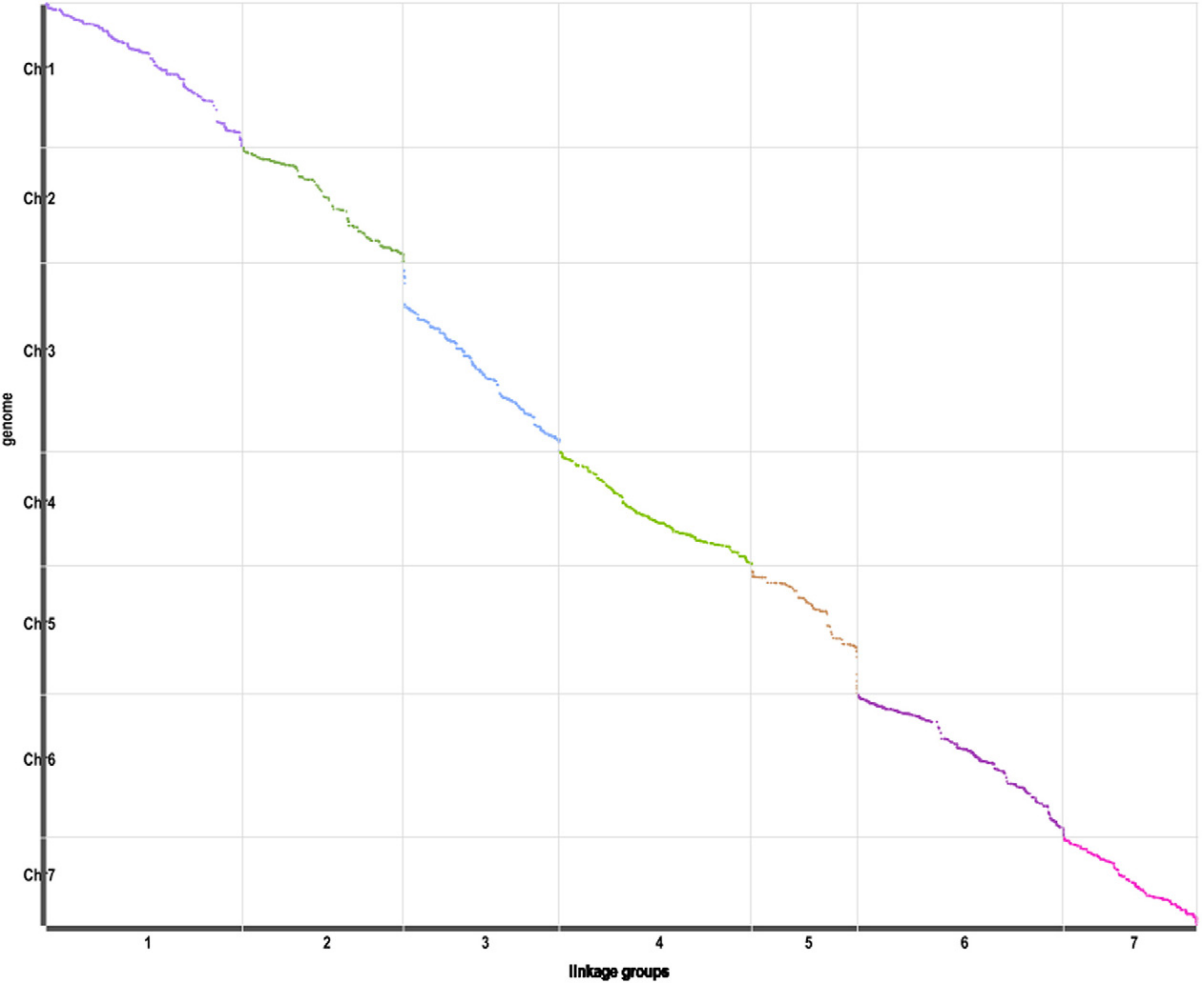
**Correlation of the genetic and physical positions**. Axis of abscissa represents the genetic group, axis of ordinate represents the physical positions.

The correlation of genetic and physical positions is another important evaluation of the genetic map (Sim et al., 2012). The correlation of genomic location of mapped SLAF markers with the corresponding physical positions will reveals the collinear relationship of genetic map with genome. The more Spearman Correlation Coefficient close to 1, the better collinearity of genetic map with physical map. As is described in the Figure 4, the validation of correlation of the genetic and physical positions shows good in this genetic map.

**Figure 5:**
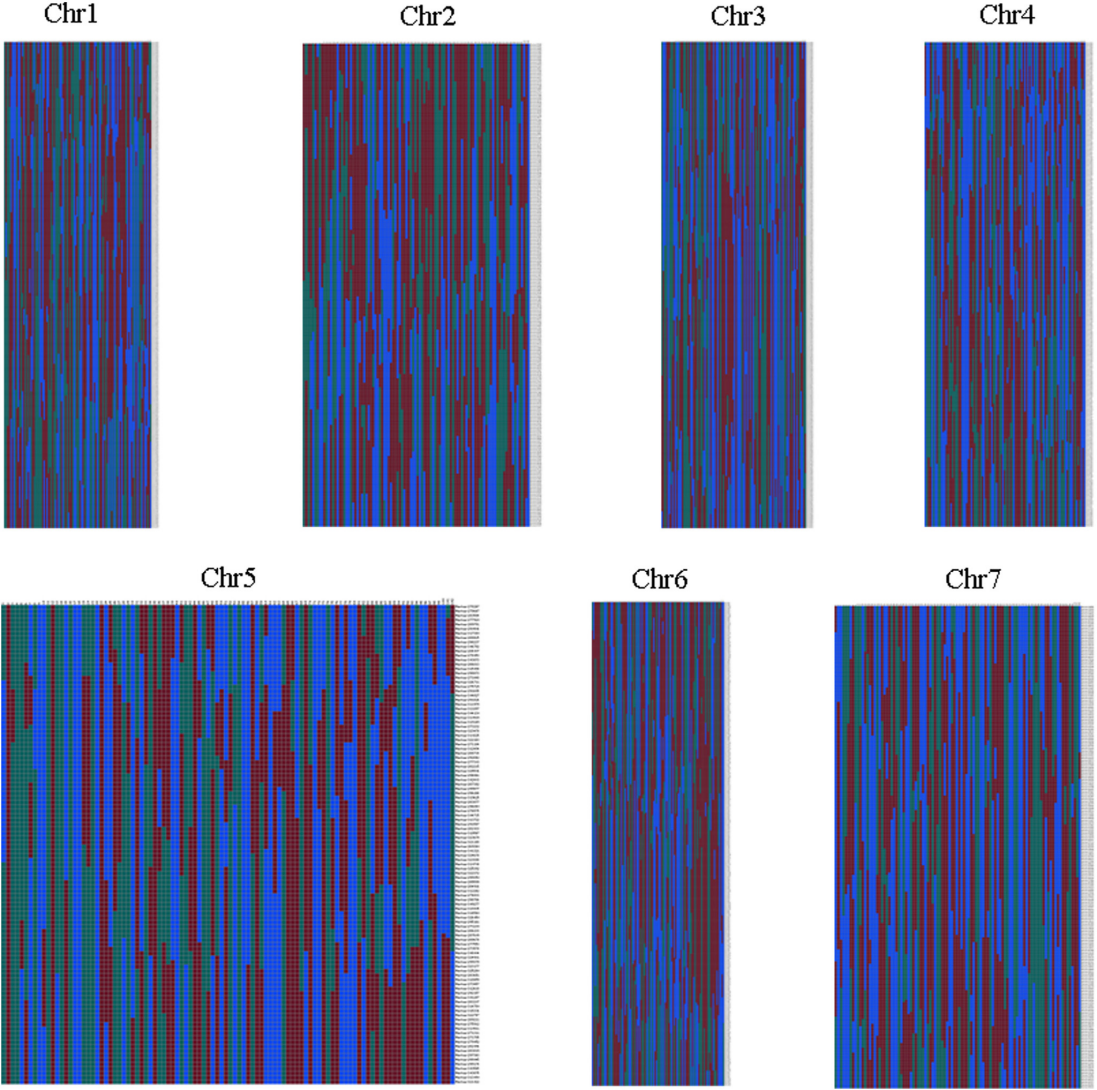
**Haplotype map of the genetic map**. Green represents Jin5-508, blue represents D8, gray represents missing data, and red indicates heterozygosity. Each two columns represent the genotype of an individual. Rows correspond to genetic markers.

**Figure 6:**
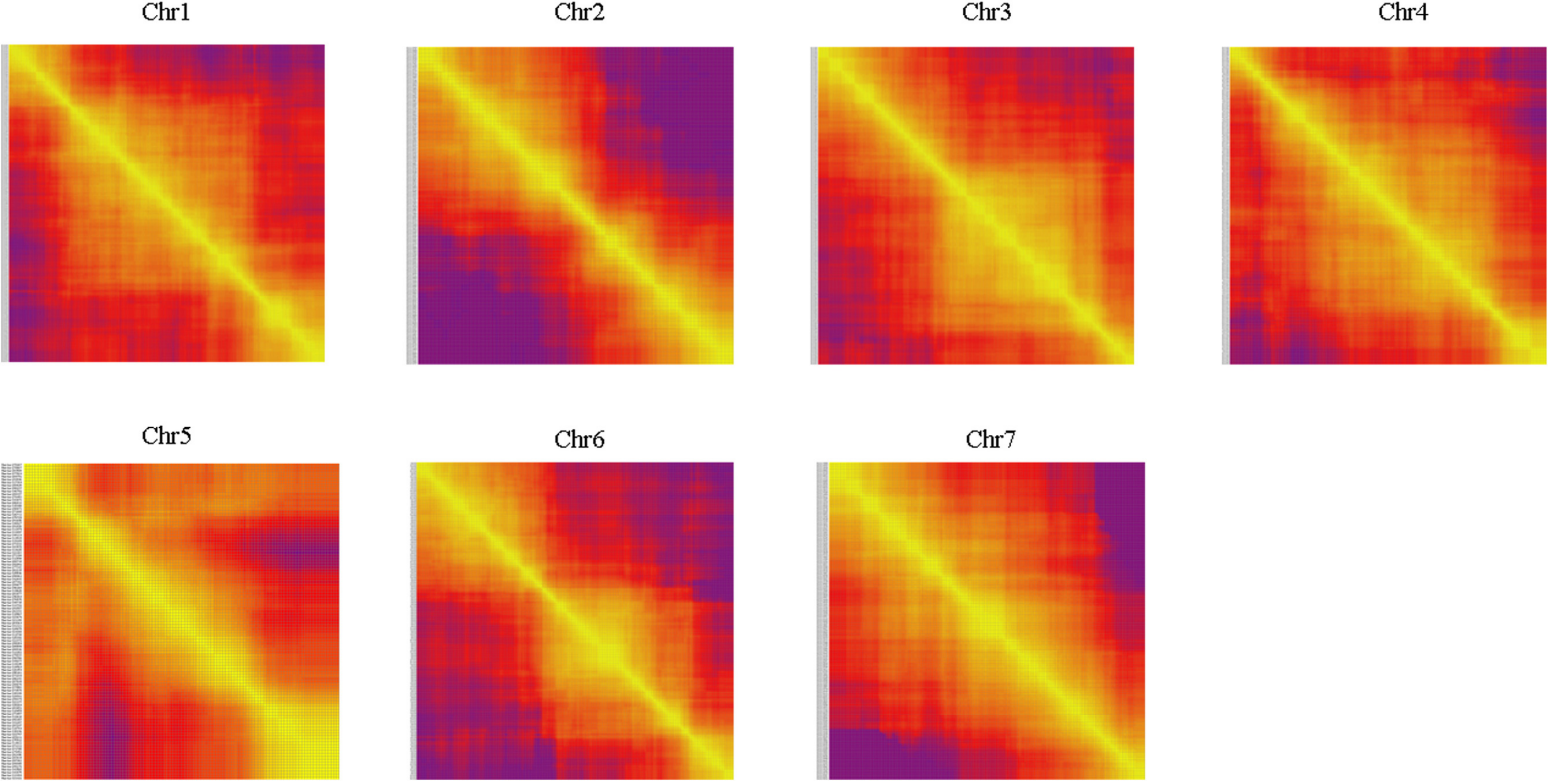
**Heat map of the genetic map**. Each cell represents the recombination rate of two markers. Yellow indicates a lower recombination rate and purple a higher one.

Haplotype map and heat map were also used to evaluation the quality of genetic map. The population of double crossover and deletions can be reflected by haplotype map on the genotyping and marker-order errors. Haplotype maps were generated for each of the 102 F_2_ individuals and for the parental controls using 1892 SLAF markers as described by West et al. (2006). Haplotype maps intuitively displayed the recombination events of each individual. As seen in Figure 5, there was no double crossover and deletion in any linkage group. Heat maps were also generated to evaluate the genetic map quality by using pair-wise recombination values for the 1892 mapped SLAF markers (Figure 6). Also, the relationship of recombination between markers from one single linkage group can be reflected by heat map. Hence, it was used to find potential ordering errors of markers. Pair-wise recombination mainly caused by hot spot region of genomic recombination and genotyping errors based on sequencing. In general, most of the LGs performed well in visualization.

## Discussion

High-density linkage maps play an important role in facilitating discovery of functional genes and comparative analysis of genome structure (Shifman et al., 2006; Wang et al., 2009; Graham et al., 2010; Hyten et al., 2010). However, most current genetic maps contain only about hundreds of markers, due to discovery technologies and genotyping costs (Liu et al., 2014). Advances in genome sequencing technologies have paved the way for significant improvements in the rapid detection of genetic variation as well as the throughput and wealth of the information obtained (Elshire et al., 2011). Based on advanced generation sequencing, genotyping approaches including traditional methods (Illumina GoldenGate assay, Infinium assay), and NGS-based methods (Reduced-Representation Libraries (RRLs), RAD (restriction site associated DNA) genotyping, and SLAF-seq (Baird et al., 2008; Sun et al., 2013) allow millions of SNPs to be identified in plant genome. The Illumina GoldenGate assay is a large-scale genotyping assay, but which can only analyze 384 to 3072 different loci in up to 96 individuals. The Infinium assay is limited to bi-allelic SNPs and cannot detect alternative alleles (David, 2015). Individuals can be directly compared for sequence variations by using reduced-representation sequencing, which only a few targeted genomic regions were sequenced, rather than the entire genome. Reads from reduced-representation sequencing can be mapped to a reference genome for polymorphism detection and haplotype analysis. However, reduced-representation sequencing is not suitable to be applied to genomes with high ploidy levels or large repetitive genome fractions (Appleby et al., 2009; David, 2015). RAD genotyping is measured by randomly interrupted genomic DNA with the restriction enzymes, but SLAF is measured by sequencing the paired-ends of the sequence-specific restriction fragment length. SLAF have better repeatability, and is considered to be better than the RAD, because of fragment length selection is not through random interrupted. It can generate large amounts of sequence information and handle whole genome density distributions (Qi et al., 2014). SLAF-seq methods have been used in several researches, such as the first high-density genetic map for sesame (Zhang et al., 2013), draft genome of the kiwifruit (*Actinidia chinensis*) (Huang et al., 2013), and high-density genetic map for soybean (Qi et al., 2014). In this research, a large amount of markers were developed in cucumber. In total, 79,092 SLAF markers were developed based on high-throughput sequencing, and 6784 polymorphic markers were identified with a polymorphism rate of 8.6%. Despite of the polymorphism rate was low, the number of SLAF markers covered all cucumber chromosomes, which had from 108 to 377 polymorphic markers each, and a total of 1892 polymorphic markers were identified for genetic map construction. In addition, marker quality met the requirements for construction of a genetic map, and marker integrity and accuracy were also high. Results accurately reflect the genetic and polymorphism characteristics of cucumber. Therefore, SLAF-seq is a useful technology to develop chromosome-specific molecular markers with the characteristic of high success rates, specificity, stability, and low cost.

The distribution rate of detected SLAFs on each chromosome is about 0.04%, which suggests SLAFs in this research distribute uniformly on each chromosome. The mean distribution rate of polymorphic SLAF on seven chromosomes is 0.0402%, but polymorphic rate is among 5.47–12.01%, which may caused by uneven distribution of genes on chromosome, polymorphic rate is high in intergenic region and low in gene region (Table 2). In order to further evaluate the quality of the high-density genetic map constructed in this research, BLASTn sequence alignment was used to assign the 1892 mapped markers to the published Gy14, 9930 and PI183967 (wild type) draft genome scaffolds (Huang et al., 2009). The SLAF-associate DNA sequences were provided in Data Sheet 1. In total, 257 Gy14 draft genome scaffolds (172.9 Mbp), 185 9930 draft genome scaffolds (172.3 Mbp), 96 PI183967 draft genome sequences (137.0 Mbp) were anchored onto the genetic map (Table S3). This SNP map showed several significant improvements over the cucumber genetic map by Yang et al. (Yang et al., 2012) in number of mapped loci (from 735 to 1892) and mapped of Gy14 draft genome scaffolds (from 244 to 257). Interesting, 75 new Gy14 draft genome scaffolds (34.6 Mbp) could be anchored onto our genetic map (Table S3), which can be used to improve Gy14 genome assembly. However, there are remaining four markers cannot be anchored to 9930 draft genome scaffolds, 30 markers cannot be anchored to the Gy14 draft genome scaffolds, 12 markers cannot be anchored to PI183967 draft genome scaffolds (Table S3), because of more than one scaffold can be anchored, which may be due to locating at overlap regions of genome of these markers.

In our study, chr5 had the shortest genetic length which resulted from two factors. First, the ration of polymorphic SLAF had the lowest ratio on chromosome 5 between Jin5-508 and D8, which resulted in only 108 SLAF markers mapped on this chromosome. Usually the lower density markers will lead to a shorter genetic length of the map. Second, there was low recombination on the end of chromosome 5 which maybe because of some structure changes between Jin5-508 and D8 in this region. The low recombination on the top of chromosome 3 also made its shorter genetic length, which usually was the longest linkage group in seven cucumber chromosomes. To the best of our knowledge, the genetic map presented in this paper had the smallest average distance (0.45 cm) to date for cucumber. Jin5-508 and D8 are two elite genetic materials and showed obvious difference in several important agronomic traits such as powder mildew resistance, plant height, fruit neck length, and pulp thickness. Therefore, the high-density genetic map developed in this study will help to precisely map and estimate the effects of quantitative trait loci for these traits. More importantly, all of the markers on this genetic map are SNPs, which are the most abundant type of genetic variation between individuals. Without doubly, our genetic map can serve as a valuable tool to cucumber breeders for marker-trait association in QTL or association mapping of important agronomic traits, marker assisted breeding, map-based gene cloning, and comparative mapping.

### Conflict of interest statement

The authors declare that the research was conducted in the absence of any commercial or financial relationships that could be construed as a potential conflict of interest.
