# Supplementary material for: A high-density genetic map of cucumber derived from Specific Length Amplified Fragment sequencing (SLAF-seq)
Source: Front Plant Sci. 2015 Jan 7;5:768. doi: 10.3389/fpls.2014.00768 (PMC4285734; doi:10.3389/fpls.2014.00768)
Supplement: Supplementary file 2 [file DataSheet1.DOC]

The DNA sequences of the sequence-associated markers

>Marker1405

TTATTAGCAAAGGTGGCGCACTCTTGTAAATCAGCCAAAGAATATGAACGCCAAGTGGAGAAATTTTTTTGAGGTGGCACXXXXXXXXXXGAACTGATCGGTCTTTTTGATATACCACCACCGTTGTTCTTGAGAGGAAAACTCCTATTCTTCTCCCACACAAGATTGTA

TTATTAGCAAAGGTGGCGCACTCTTGTAAATCAGCCAAAGAATATGAACGCCAAGTGGAGAAAGTTTTTTGAGGTGGCACXXXXXXXXXXGAACTGATCGGTCTTTTTGATATACCACCACCGTTGTTCTTGAGAGGAAAACTCCTATTCTTCTCCCACACAAGATTGTA

>Marker1784

CATTTTGATTTCGCTTTTAGTTTTGAAATTTATGCTTATTTCCTCTTAATTTTCCATTATAATTTTTACATTGTTAAAAAXXXXXXXXXXCTAAATATTTCAAAATTGTAAAAGTAATATTCAACTGAGACAAGAACATACTTGTATCCCGGAAGAGGTTCTAAACGAAA

CATTTTGATTTCGCTTTTAGTTTTGAAATTTATGCTTATTTCCTCTTAATTTTCCATTATAATTTTTACATTGTTAAAAAXXXXXXXXXXCTAAATATTTCAAAATTGTAAAAGTAATATTCAACAGAGACAAGAACATACTTGTATCCCGGAAGAGGTTCTAAACGAAA

>Marker2027

ATATGCTATATATATAATATTCTATATATACTATACATATATTGCCTTGTCAGCTTCTATTGTGGGTCGTGCCTTGCCAGXXXXXXXXXXCAAATTTCTGATTTTAGGATGCCATCTCTAGTTTATTCTTTTGATTGGAGGTTTGCCAGTAAAATATGGTTTTCTTTTTG

ATATGCTATATATATAATATTCTATATATACTATACATATATTGCCTTGTCAGCTTCTATTGTAGGTCATGCCTTGCCAGXXXXXXXXXXCAAAATTCTGATTTTAGGATGCTATCTCTAGTTTATTCTTTTGATTGGAGGTTTGCCAGTAAAATATGGTTTTCTTTTTG

>Marker2133

TTGATCCTAATGATATGGAAAGCAAGAAAGCCAAAGAAGAGCACCAGTCATAGTAGATTCATTCAATAAGGGAAACTTCCXXXXXXXXXXTTTAACGACTGTTTGGTTGGTTCATTGGAGAGATTTTCAATTCAATGTTTGTTAGCAAAAAGCCACTATGACGCTCGAGT

TTGATCCTAATGATATGGAAAGCAAGAAAGCCAAAGAAGAGCACCAGTCATAGTAGATTCATTCAATAAGGGAAACTTCCXXXXXXXXXXATTAACGACTGTTTGGTTGGTTCATTGGAGAGATTTTCAATTCAATGTTTGTTAGCAAAAAGCCACTATGACGCTCGAGT

>Marker2459

TAAATAGGGAATACACAAATCTCAGCATAAATTAAATTGATTGGTAAAGGAATATGTTCTAAATTAGTTAGAGGTTTAGGXXXXXXXXXXATAAATCAATCAATTGTTGTCAATATTTATGTTCTAATGATAAACGAAATTCCACACAACCTTTTACCTTTATGACTAAA

TAAATAGGGAATACACAAATCTCAGCATAAATTAAATTGATTGGTAAACGAATATGTTCTAAATTAGTTAGAGGTTTAGGXXXXXXXXXXATAAATCAATCAATTGTTGTCAATATTTATGTTCTAATGATAAACGAAATTCCACACAACCTTTTACCTTTATGACTAAA

>Marker2608

TTTTCCGTAATTTCGGACCTCTAAAACCTTCGTGACTGAAATAATTAATGTTATATTATTGATTGAATTATTATTCATTTXXXXXXXXXXCATATTTTTACTTCTAGTATTTTATTTATAATCTAAAAGAAGACAACAGTTAATTATTATCTAAAATAAATGAAACTATA

TTTTCCGTAATTTCGGACCTCTAAAACCCTCGTGACTGAAATAATTAATGTTATATTATTGATTGAATTATTATTCATTTXXXXXXXXXXCATATTTTTACTTCTAGTATTTTATTTATAATCTAAAAGAAGACAACAGTTAATTATTATCTAAAATAAATGAAACTATA

>Marker2784

TATCTCTCACACATCTCGTGTTAAACACCAAAAGTTTCTTAAACCTTTGAAACTTGTTACGGTTAGTTAAGGACAAAAAAXXXXXXXXXXAATGGGCGGAAAGATGAAGGTTGAGAACCTCTAAGTATAAGTGTTGGGCAAGCTTTGGCGTTTTGGAGCAAGGACTATGT

TATCTCTCACACATCTCGTGTTAAACACCAAAAGTTTCTTAAACCTTTGAAACTTGTTAAGGTTAGTTAAGGACAAAAAAXXXXXXXXXXAATGGGCGGAAAGATGAAGGTTGAGAACCTCTAAGTATAAGTGTTGGGCAAGCTTTGGCGTTTTGGAGCAAGGACTATGT

>Marker2930

ATCCACATGATGACTTTACACATTATATGCACACTACATAATAGCAACTCACTTGATAGGAATAAGGATAATGGTTTACTXXXXXXXXXXTTATAAAGGTCACTCATGAAAATACTTGATTCCCTTCTTCTCATAACGCCTAATTTCTTCAACAAGCAGCTCAACTCTTA

ATCCATATGATGACTTTACACATTATATGCACACTACATAATAGCAACTCACTTGATAGGAATAAGGATAATGGTTTACTXXXXXXXXXXTTATAAAGGTCACTCATGAAAATACTTGATTCCCTTCTTCTCATAACGCCTAATTTCTTCAACAAGCAGCTCAACTCTTA

>Marker3313

ATCAATTGTAGCACACGCCCCCACAAGATAAATCATTTACGTATCAGCATTCAATTTCATAAAATATCCTACGAGTCATTXXXXXXXXXXTCTTTGTTGCTAACTTTAAATTAAATTTTGGTCTATTTCTTGCCCTATAAATTGTAATTTGACACAAGTTTATCTTTTTG

TTCAATTGTAGCACACGCCCCCACAAGATAAATCATTTACGTATCAGCATTCAATTTCATAAAATATCCTACGAGTCATTXXXXXXXXXXTCTTTGTTGCTAACTTTAAATTAAATTTTGGTCTATTTCTTGCCCTATAAATTGTAATTTGACACAAGTTTATCTTTTTG

>Marker3384

TATTCTGTGGATTTTGGACCTCTCAACATCTTACGTGCTGCTGCTTTTCATGGGGCTAACTATATTTCTCCTCTCAAAAAXXXXXXXXXXTGAGGCTTCGTAATTGTTTGCCAAGTATGAATGTTAAGAAGTTGGAGAGTTGGAAACTTTGATTTGATTATTTTTCTTAA

TATTCTGTGGATTTTGGACCTCTCAACATCTTACGTGCTGCTGCTTTTCATGGGGCTAACTATATTTCTCCTCTCAAAAAXXXXXXXXXXTGAGGCTTCGTAATTGTTTGCCAAGTATGAATGTTAAGAAGTTTGAGAGTTGGAAACTTTGATTTGATTATTTTTCTTAA

>Marker3780

ACACACCCATATTAAAATATATAATACCTTTTTGGTTTTTTGTATTGATAGAGCAGGGGTTTTCACATAGGCTTCGACAAXXXXXXXXXXAGAATATCACAAGTGAAAATACCATAATGAAATAAGGTCCACATGAACATACTGGAGGTGTGTAAAGTTTGTTTTACAGA

ACACACCCATATTAAAATATATAATACCTTTTTGGTTTTTTGTATTGATAAAGCAGGGGTTTTCACATAGGCTTCGACAAXXXXXXXXXXAGAATATCACAAGTGAAAATACCATAATGAAATAAGGTCCACATGAACATACTGGAGGTGTGTAAAGTTTGTTTTACAGA

>Marker4028

TTAATGTCGAGTTTCAAGTTGAAAATAGGAAGAGACCTAAAAAGCACTAGAAAAGTATCTTCTTTTGTTCTTATTTAAAGXXXXXXXXXXCATGGTGTCACAATTGCAACATTTCCATTACGAGACAAGCGATTGTGCAACACTTGCTCTAATTCAACAAGCAGGGGTTA

TTAATGTTGAGTTTCAAGTTGAAAATAGGAAGAGACCTAAAAAGCACTAGAAAAGTATCTTCTTTTGTTCTTATTTAAAGXXXXXXXXXXCATGGTGTCACAATTGCAATATTTCCAATACGAGACAAGCGATTGTGCAACACTTGCTCTAATTCAACAAGCAGGGGTTA

>Marker4271

ATAAATGTGTTATTGTTATCTTATATCGGTTGTAAATGATATTGATTTTCTATCTAAGTGGTGTCTAATTGTTAATTGATXXXXXXXXXXCTTGTCACATGATATTGATTGATATCAAAAGATTATTAGACCTCTCTAATTTAATATCTATTAAATACAAGTTAAAGGAG

ATAAATGTGTTATTGTTATCTTATATCGGTTGTAAATGATATTGATTTTCTATCTAAGTGGTGTCTAATTGTTAATTGATXXXXXXXXXXCTTGTCACATGATATTGATTGATATCAAAAGATTATTATACCTCTCTAATTTAATATCTATTAAATACAAGTTAAAGGAG

>Marker4685

TGTAATTACTCTAGGTTTAGTTGTCTTGCTAAACAGCTTTGTTTCGGTGTAATCTCGTTTTCAGTTGAAGGCTGTTCTATXXXXXXXXXXTAATATTGTCCACCATGGCTTATGGTAAAAAACAATATTACACCTTGAATCAAGATGACGTCAAATTTCTGATGAGCAGA

TGTAATTACTCTAGGTTTAGTTGTCTTGCTAAACAGCTTTGTTTCGGTGTAATCTCGTTTTCAGTTGAAGGCTGTTCTATXXXXXXXXXXTAATATTGTCCACCATGGCTTATGGTAAAAAATAATATTACACCTTGAATCAAGATGACGTCAAATTTCTGATGAGCAGA

>Marker4701

CTTTACTGTAAATGAATGTTTAATTGATTTCAAAAAATATTTTATACATTTGTTCTAACATACAAGAGCTAAATGTTTCTXXXXXXXXXXCAGGTTAAAGTATCATATTCCACTAAGCATGTTTCAACAACTAGTAAATCATATTTACCTTGTATTTCCTTCTCAACAAG

CTTTACTGAAAATGAATGTTTAATTGATTTCAAAAAATATTTTATACATATGTTCTAACATACAAGAGCTAAATGTTTCTXXXXXXXXXXTAGGTTAAAGTATCATATTCCACTAAGCATGTTTCAACAACTAGTAAATCATATTTACCTTGTATTTCCTTCTCAACAAG

>Marker4941

TTGCAAAGAAACTACGGACTAAACAGCCACCAGACAATCTTGAAACAGAAACAGAGAGCCAAAAGGCATGTGTTAAATATXXXXXXXXXXAGAACTGAGAAACCGAAATAATGTATAATAATATAACCATGTATCTGTTAAAAACCACAAAATTGAATCAAAGAAATTAA

TTGCAAAGAAACTACGGACTCAACAGCCACCAGACAATCTTGAAACAGAAACAGAGAGCCAAAAGGCATGTGTTAAATATXXXXXXXXXXAGAACTGAGAAACCGAAATAATGTATAATAATATAACCATGTATCTGTTAAAAACCACAAAATTGAATCAAAGAAATTAA

>Marker5116

ATTACTTGATTTACCAGCCGGTGAGCTATCTACTTCCAGGAAATAAAGATCCTACAATAATATCTCAGCAAGTTAGAACTXXXXXXXXXXAAATTATTTCTCAACTTTAATCCAAATATGAATGACAAAAATTATTTAATGTGTGAAGTCTTTTTTTTTCTTTTTTTTGT

ATTACTTGATTTACTAGCCGGTGAGCTATCTACTTCCAGGAAATAAAGATCCTACAATAATATCTCAGCAAGTTAGAACTXXXXXXXXXXAAATTATTTCTCAACTTTAATCCAAATATGAATGACAAAAATTATTTAATGTGTGAAGTCTTTTTTTTTCTTTTTTTTGT

>Marker5156

TGATACAATATCACCTCTAACAGGTTCACCACTGACAGATTCTTGTGCACTCACCAACGGTGGCAATTCTGGCAAAAAATXXXXXXXXXXTAACTTAGAGTGGGGAAGGGGGCAGAATAATCAAAACATAAATGGAAAATTCTGAATTCATACCTGGTGAGGCAGCTCAA

TGATACAATATCACCTCTAACAGGTTCACCACTGAGAGATTCTTGTGCACTCACCAACGGTGGCAATTCTGGCAAAAAATXXXXXXXXXXTAACTTAGAGTGGGGAAGGGGGCAGAATAATCAAAACATAAATGGAAAATTCTGAATTCATACCTGGTGAGGCAGCTCAA

>Marker5314

TTCAATGTTGATAGAAATATAAGTCTTAATTTTATTGAAATTTCAATGAAAAAGGTGATTTCGAGGAATGTTTTTGAGAAXXXXXXXXXXAAATATCGAACATTGATATCAAACGTGAAAATGTCAATGAAAATATCTACATATATTATTCATAATTTAATACATGGTTG

TTCAATGTTGATAGAAATATAAGTCTTAATTTTATTGAAATTTCAATGAAAAAGACGATTTCGAGGAATGTTTTTGAGAAXXXXXXXXXXAAATATCGAACATTGATATCAAACGTGAAAATGTCAATGAAAATATCTACATATATTATTCATAATTTAATACATGGTTG

>Marker5423

TGAAATTGATTCAACGAAATCCTTATAATATATGAACAATTCACAATCCTATTACGTTTGATTAATTTTGTTGGATGAAAXXXXXXXXXXTTTTAAAGCATGCATTGAGTTGGATCCAAGTTTATTTGTTTTACTCTACACATTTTTGTTTTGTAGTATGCTCAAATAAT

TGAAATTGATTCAACGAAATCCTTATAATATATGAACAATTCACAATCCTATTACGTTTGATTAATTTTGTTGGATGAAAXXXXXXXXXXTTTTAAAGCATGCATTGAGTTGGATCCAAGTTTATTTGTTTTACTCTACAAATTTTTGTTTTGTAGTATGCTCAAATAAT

>Marker5588

ATAAAAATATCTCCTCAACCCAATTACAACACTCCCTCCAAGCATTTGTGATGATTTGAAACTAACGACATAGGCTCTTAXXXXXXXXXXAATATCTTGTTACACAAAGGCAAAAGGAATAATAGAGCACTAGAGATGTTGAGAATCTAGGGCACAATATTTGAAATCAT

ATAAAAATATCTCCTCAACCCAATTACAACACTCCCTCCAAGCATTTGTGATGATTTGAAACTAACGACATAGGCTCTTAXXXXXXXXXXAATATCTTGTTACACAAAGGCAAAAGGAATAATAGAGCACTAGAAATGTTGAGAATCTAGGGCACAATATTTGAAATCAT

>Marker5600

CCAACTCCAATGAATATGGAAATAACCAGACGATTCTTCATCATACAATTGGGTTGCCGGATTTCAGTCAAGAACTTCAAXXXXXXXXXXGCAGCAATTCCTCATCCTTCTTTCTCTATGCAACAACTTATTCCACTTTCCACCTCTCCCAAGTAATTTCTATTCTTAAA

CCAACTCCAATGAATATGGAAATAACCAGACGATTCTTCATCATACAATTGGGTTGCCGGATTTCAGTCAAGAACTCCAAXXXXXXXXXXGCAGCAATTCCTCATCCTTCTTTCTCTATGCAACAACTTATTCCACTTTCCACCTCTCCCAAGTAATTTCTATTCTTAAA

>Marker5766

CAAATCTGACCCAGTCCCCGGCAATGGAAAGAACCCGTTAAGAGAAGATGAAAGAGAAACAGAGATGAAGCATCAGACCGXXXXXXXXXXCAAAGTGAAAGAGTGAGAAGTGGAAAAGGGAATTGCTTTCAGCAGGTGTGGGTTTTGATTTTGGTTGGTGGGATCCACGT

CAAATCTGACCCAGTCCCCGGCAATGGAAAGAACCCGTTAAGAGAAGATGAAAGAGAAACAGAGATGAAGCATCAGACCGXXXXXXXXXXCAAAGTGAAAGAGTGAGAAGTGGAAAAGGGAATTGCTTTCAGCAGGTGTGGGTTTTGATTTTGGTTGGTGGGACCCACGT

>Marker6036

TGAGAAACAAGGACGAATCTGCATGTTCTTGGATTCTGTTGATACAGTAGTCAAACGCAAGGTAATGAAACCAAGCGTAGXXXXXXXXXXGGTATTGCGAAAAGGATTTCATAAAACCTTAGTCTTTTAGACATTCTTTCAAAAATTAATCTTCCATGATCAGGCGTTTA

TGAGAAACAAGGACGAATCTGCATGTTCTTGGATTCTGTTGATACAGTAGTCAAACACAAGGTAATGAAACCAAGCGTAGXXXXXXXXXXGGTATTGCGAAAAGGATTTCATAAAACCTTAGTCTTTTAGACATTCTTTCAAAAATTAATCTTCCATGATCAGGCGTCTA

>Marker6350

TGGATCTTCCTGTGTAGCATTACTGTCAAAAGAAAAGCAAAAAATTAATGGTCTAATGCTCCGACGGTTTGTTTGCTTGTXXXXXXXXXXTCACAAAGTGGGACGATGAGAATGTCATCCTCCACCCACGAAAAAGTAGATCAATGAAAATATTACCCAGTTCGAGATGG

TGGATCTTCCTGTGTAGCATTACTGTCAAAAGAAAAGCAAAAAATTAATGGTCTAATGCTCCGACGGTTTGTTTGCTTGTXXXXXXXXXXTCACAAAGTGGGACGATGAGAATGTCATCCTCCACCCACGAAAAAGTCGATCAATGAAAATATTACCCAGTTCGAGATGG

>Marker6643

CGATCAAAATTATAAGATCGTTAATATTAGAAAAACTAGTTTGAAAACCTTGAAACGAAATGAATTTAATTACATTATAAXXXXXXXXXXTCATTGATTTGAAATTTTAGTTAATGATTCAACTCCTCGCTTTTGACCGAAACATAACTTTGTGAAGAAAATAATAAATG

CGATCAAAATTATAAGATCGTTAATATTAGAAAAACTAATTTGAAAACCTTGAAACGAAATGAATTTAATTACATTATAAXXXXXXXXXXTCATTGATTTGAAATTTTAGTTAATGATTCAACTCCTCGCTTTTGACCGAAACATAACTTTGTGAAGAAAATAATAAATG

>Marker6706

TTATCTTAATGTGTTCTCATGGTTGAATGAAATAATGAGTTATTGATTGAAAAATCATAAATATTAGTCGACTAAATAACXXXXXXXXXXATGGTCATAAAAGAAAAGCTCCATAAGAAAAAGAGAAAAATATGGAAGCAATTTTTTCATATCAAATTAATAATATATTT

TTATCTTAATGTGTTCTCATGGTTGAATGAAATAATGAGTTATTGATTGAAAAATCATAAATATTAGTCGACTAAATAACXXXXXXXXXXATGGTCATAAAAGAAAAGCTCCATAAGAAAAAGAGAAAAATGTGGAAGCAATTTTTTCATATCAAATTAATAATATATTT

>Marker6768

TTTTCCTTTATTAGTATTTTGGTTTCTATTTATTTACCAATGTAAAAGGTGATCGTTCTGAATATTCAATACATTATATAXXXXXXXXXXTCAATTGGAGGCTTAGCACATTACTAGAGTGATTACTTGTCTTTGGTGAACAAGGTCTCTCCTTTTTCTAATCGTATGTT

TTTTCCTTTATTAGTATTTTGGTTTCTATTTATTTACCAATGTAAAAGGTGATCGTTCTGAATATTCAATACATTATATAXXXXXXXXXXTCAATTGGAGGCTTAGCACATTACTAGAGTGATTACTTGTCTTTGGTGAACAAGGTATCTCCTTTTTCTAATCGTATGTT

>Marker7129

TAAGCAACTGAAAATGAACCAGATAATTCTCTAAGGGATATTGTTGATGAAAATGTCATCATAGTAGTCTATGTGTGGGAXXXXXXXXXXGCTCTCAGGTAATTTCAAAGTTGTGTTTGGTCGAGACTAGAGATAGAGAATGTTCTGTTGAGGTTCTCGAAGTGATCTCA

TAAGCAACTGAAAATGAACCAGATAATTCTCGAAGGGATATTGTTGATGAAAATGTCATCATAGTAGTCTATGTGTGGGAXXXXXXXXXXGCTCTCGGGTAATTTCAAAGTTGTGTTTGGTCGAGACTAGAGATAGAGAATGTTCTGTTGAGGTTCTCGAAGTGATCTCA

>Marker7605

CTGTGCCTCTTGTCTCTCCATTTCTCTTTCATTAACTTTTCTCGCTTCCCTTGGATATCTGATCCCTTTATCATGTCCTAXXXXXXXXXXCAAAAAACGTGGAGGCGAACAAGCTCCTAATCAGCAAGATAAAGTTAAGCTAACAATTACAAAAAAGCTTCGTAACTGAA

CTGTGCCTCTTGTCTCTCCATTTCTCTTTCATTAACTTTTCTCGCTTCCCTTGGATATCTGATCCCTTTATCATGTCCTAXXXXXXXXXXCAAAAAACGTGGAGGCGAACAAGCTCCTAATCAGCAAGAGAAAGTTAAGCTAACAATTACAAAAAAGCTTCGTAACTGAA

>Marker7752

CTTTGTTAAGAATTCAATCCCTCACCACTTTTTGCTGAAGTGTCTGATTTGTTGTTCTATGAGACTATGAGCCATAGTAGXXXXXXXXXXTCGCTGCTTGCTTGTGGCTTGAGGTTATCTATTCTAAGAGAATAAAAGATCGTAGTATTTATTGAATTTCGTATTATTAT

CTTTGTTAAGAATTCAATCCCTCACCACTTTTTGCTGAAGTGTCTGATTTGTTGTTCTATGAGACTATGAGCCATAGCAGXXXXXXXXXXTCGCTGCTTGCTTGTGGCTTGAGGTTATCTATTCTAAGAGAATAAAAGATCGTAGTATTTATTGAATTTCGTATTATTAT

>Marker7950

AAATTTAATTTTGTAATCAATGAAAATAATAGATAGATACCAATAATTTACTTTGAAATTATGTTTAATTACTTTAAGATXXXXXXXXXXATTATAGTTATGGACGTAATGAAGGAAGATGAAGAAGCAGAAGCAGGCAGCAGCACAGAATTATGTTTACATGACATCAT

AAATTTAATTTTGTAATCAATGAAAATAATAGATAGATACCAATAATTTACTTTGAAATTATGTTTAATTACTTTAAGATXXXXXXXXXXATTATAGTTATGGATGTAACGAAGGAAGATGAAGAAGCAGAAGCAGGCAGCAGCACAGAATGATGTTTACATGACATCAT

>Marker7980

TTTGATCTTGCCCGGCTTGCAAATGATGATGATGTCATGCTTCTTGATTGGGTATGTTTTGCCTTACCTCTATATGTTTGXXXXXXXXXXTATTATCAGAAGAGTTTAAATAAAAGTGATATTTTTTAGAAACATTTTTTTCCTAGTCAATCCTAACACTCTTCATACAA

TTTGATCTTGCCCGGCTTGCAAATGATGATGATGTCATGCTTCTTGATTGGGTATGTTTTGCCTTACCTCTATATGTTTGXXXXXXXXXXTATTATCATAAGAGTTTAAATAAAAGTGATATTTTTTAGAAACATTTTTTTCCTAGTCAATCCTAATACTCTTCATACAA

>Marker8373

TAAATAATATGGCAGCTGCTTTGTTTCTGAGGGAAGATGTTCAGGTTTGATGAAACAATTGGAGATTTGTCGGATGTTTGXXXXXXXXXXAAGAAACGTTTAACATATTTATATCTATTCATATCTTAATTTTACGGACATCTTGATAGAAAATTGATAAAAAAAAATAT

TAAATAATATGGCAGCTGCTTTGTTTCTGAGGGAAGATGTTCAGGTTTGATGAAACAATTGGAGATTTTTCGGATTGGAGXXXXXXXXXXAAGAAACGTTTAACATATTTATATCTATTCATATCTTAATTTTACGGACATCTTGATAGAAAATTGATAAAAAAAAATAT

>Marker8660

TCTCTCAATAAAACTTTCAAGGCTGCTATTTTCAACCCTTTGGTTCAAGAAAGCAGCTTCTACTTTTGTGTTATTCATGTXXXXXXXXXXTGATCGATCAAATGTCTCCCTTTGGGAGAGTTTCCGTTATCTAACTGACACCATTGTAGTTGGGCAATCTTTGTCTGTTG

TCTCTCAATAAAACTTTCAAGGCTGCTATTTTCAACCCTTTGGTTCAAGAAAGCAGCTTCTACTTTTGTGTTATTCATGTXXXXXXXXXXTGATCGATCAAATGTCTCCCTTTGGGAGAGTTTCCGTTATCCAACTGACACCATTGTAGTTGGGCAATCTTTGTCTGTTG

>Marker8701

ATTAATCCACTTAGTATAAAAAATGAATAAAAAACTTTACTTATAATTCTTATTGTGTTAGTCGTGCTATTTTCATCACTXXXXXXXXXXGATTATATTTTATTTTACTAGGCTTAGTCATTTAGATTTCGAAAGGAGACCAAATGTGGAGTTAAATGACTATGAGAGAA

ATTAATCCACTTTGTATAAAAAATGAATAAAAAACTTTACTTATAATTCTTATTGTGTTAGTCGTGCTATTTTCATCACTXXXXXXXXXXGATTATGTTTTATTTTACTAGGCTTAGTCATTTAGATTTCGAAAGGAGACCAAACGTGGAGTTAAATGACTCTGAGAGAA

>Marker8790

TCTAACCAATTGCCCTTTAAATAAAACCATAAATAAAACCAATTAGTATATGAATGCATTAATTTCAATAGCTCTCTGGTXXXXXXXXXXCAGTGAGCAATGAACAATGAATTAAAATGAAGAATGAAAGTGGAAGGTGTCTGTGAGCCAAAGCATCTGCTAGAAAAAGG

TCTAACCAATTGCCCTTTAAATAAAACCATAAATAAAACCAATTAGTATATGAATGCATTAATTTCAATAGCTCTCTGGTXXXXXXXXXXCAGTAAGCAATGAACAATGAATTAAAATGAAGAATGAAAGTGGAAGGTGTCTGTGAGCCAAAGCATCTGCTAGAAAAAGG

>Marker9045

TCAATCCTATACATAACTCGTCCAAAAAAAACGTGATGATTTACCAAAGAATTTTGATCAAGTGTTTGAATGAAAAATCAXXXXXXXXXXAAGAGAAGAGAAGTGATTAGAAAAATTGGAAAGAAGAAAAAGTGGAGAGGGAAGGCATGTGAGCTTGATTTCCGGGAAAA

TCAATCCTATACATAACTCGTCCAAAAAAAACATGATGATTTACCAAAGAATTTTGATCAAGTGTTTGCATGAAAAATCAXXXXXXXXXXAAGAGAAGAGAAGTGATTAGAAAAATTGGAAAGAAGAAAAAGTGGAGAGGGAAGGCATGTGAGCTTGATTTCCGGGAAAA

>Marker9121

ATGGATTTGGAAAGTTCGTTCAGGTGAAAGTTTTGTTAGATGCCTATGAGCCCAACCATGGAATTTAATTGCTAGGACAAXXXXXXXXXXAAAAAGAGCTTTGTTTCATTGACGTCAATGTATGTTAAAGAATTAAGAGGTCTATAGTTGAAATTTCTTCACTCCTGTAT

ATGGATTTGGAAAGTTCGTTCAGGTGAAAGTTTTGTCAGATGCCTATGAGCCCAACCATGGAATTTAATTGCTAGGACAAXXXXXXXXXXAAAAAGAGCTTCGTTTAATTGACGTCAATGTATGTTTAAGAATTAAGAGGTCTATAGTTGAAATTTCTTCACTCCTGTAT

>Marker9128

ATTTCAAAATGGAGAAAAAATTACTTTTATTAGGTTCGAAGTATTAATCCAAATGGTTTTGGTTTTGCTCTATTACTAAGXXXXXXXXXXACTAAAAGACGAAAATGTTGATGTGAACAGGCTATGTCAAAGGTTGCAACCACAGGAGCGATGACATTTTCTTTCATAAA

ATTTCAAAATGGAGAAAAAATTACTTTTATTAGGTTCGAAGTATTAATCCAAATGGTTTTGGTTTTGCTCTATTACTAAGXXXXXXXXXXACTAAAAGATGAAAATGTTGATGTGAACAGGCTATGTCAAAGGTTGCAACCACAGGAGCGATGACATTTTCTTTCATAAA

>Marker10080

TCAACAGAAGGAAATTTGCAAAAATGTTCTTCCTGAGGATTAATTCCTAATAATTTAAACTGAAGGTAAACGTCCTATCGXXXXXXXXXXAAAGAAAAAGAAGAAGAAAAATTACCCCATAAGATTAACACGAAGCCGAACACTCAATTCGACAAAAAGCTCGAACATCT

TCAACAGAAGGAAATTTGCAAAAATGTTCTTCCTGAGGATTAATTCCTAATAATTTAAACTGAAGGTAAACATCCTATCGXXXXXXXXXXAAAGAAAAAGAAGAAGAAAAATTACCCCATAAGATTAACACGAAGCCGAACACTCAATTCGACAAAAAGCTCGAACATCT

>Marker10125

TCTCTTCAAAACTACCCATATGTGTTACAGACCTTGTAACTTAATCTTATGATGACTTTTCGTTAGATACTCCGATTTCTXXXXXXXXXXGAAAGACATGATTTACGTATATCTGCCTCTAAGCCTTTCAAATTATGGGCAAATCTTTTTAATGGTTGCTTATTTTATCT

TCTCGTCAAAACTACCCATATGTGTTACAGACCTTGTAACTTAATCTTATGATGACTTTTCGTTAGATACTCCGATCTCTXXXXXXXXXXGAAAGACATGATTTACATATATCTGCCTCTAAGCCTTTCAAATTATGGGCAAATCTTTTTAATGATTGCTTATTTTATCT

>Marker10285

ATGCTAAACAATCGTGTTAACTATGTTAAGCAATTGTTTAGATCATATCTCCACACGACCATATAATTCTTTTTAAACGAXXXXXXXXXXAGAATGGTTAAACGATCATTCAGATCATATCAAAGTGATATTTAAATGATTCTGATACTTTCAAATATGAGAAAGATGAA

ATGCTAAACAATCGTGTTAACTATGTTAAACAATTGTTTAGATCATATCTCCACACGACCATATAATTCTTTTTAAACGAXXXXXXXXXXAGAATGGTTAAACGATCATTCAGATCATATCAAAGTGATATTTAAATGATTCTGATACTTTCAAATATGAGAAAGATGAA

>Marker10439

TCGCGACATACAATATACTTGTTCAAAGACAAGTATACAGCAGGAGATAAAACCAGCAGGAAAGATTGATTGGAGCAATCXXXXXXXXXXCAATAGGAAAAGAGTAAAATAATATACATAAACAAGGCATGAACAATTAGCATTTAACATGCAAACCTCTGAAAGTAAGT

TCACGACATACAATATACTTGTTCAAAGACAAGTATACAGCAGGAGATAAAACCAGCAGGAAAGATTGATTGGAGCGATCXXXXXXXXXXCAATAGGAAAAGAGTAAAATAATATACATAAACAAGGCATGAACAATTGGCATTTAACATGCAAACCTCTGAAAGTAAGT

>Marker10750

TCAATGGTTTTTCTGTTTGTCTTTCTGAACATCAACTCTTTACCAGGTATCACCTTCAAGTATCTCAAGATTTTATTAACXXXXXXXXXXGGACATCCCATCATACCTGTCTAAGTCAACAAATCAAGGGTGTATTTTCTCTGAGATATGGATATGCCTTCTTTAGATCT

TCAATGGTTTTTCTGTTTGTCTTTCTGAACATCAACTCTTTACCAGGTATCATCTTCAAGTATCTCAAGATTCTATTAACXXXXXXXXXXGGACATCCCATCATACCTGTCTAAGTCAACAAATCAAGGGTGTATTTTCTCTGAGATATGGATATGCCTTCTTTAGATCT

>Marker11206

TCTACTCTCAGGGAAGAAGCTTTTGGTTTCAGTTGCTGCTGGGGTAAAACTGAAGGATCTACAGGTTCTTAACTTTACTAXXXXXXXXXXCTCTCCCAACATTTTGTCGTGTTGGGTTAAATGGTTAAAAGTAAGTTTTTATAATTTGAATCATTCTTAAGCATTTTGAA

TCTACTCTCAGGGAAGAAGCTTTTGGTTTCAGTTGCTGCTGGGGTAAAACTGAAGGATCTACAGGTTCTTAACTTTACTAXXXXXXXXXXCTCTCCCAACATTTTGTCGTGTTGGGTTAAATGGTTAAAAGTAAGTTTTTATAATTTGAATCATTCTCAAGCATTTTGAA

>Marker11269

ATTTCTACTGTTTTGTCTTTTAGCTCATATCTAACACAAACTTTTATATTGTTTACATCACAGGTCAAAAACTTTTATGTXXXXXXXXXXAAAGTATCAGGATTTAGAGTTTCTCAAATGTCAAATAATAATAATAACAGTAAATAAACCATCCATAAAGGAAAAGAATT

GTTTCTACTGTTTTGTCTTTTAGCTCATATCTAACACAAACTTTTATACTGTTTACATCACAGGTCAAAAACTTTTATGTXXXXXXXXXXAAAGTATCAGGATTTAGAGTTTCTCAAATGTCAAATAATAACAATAACAGTAAATAAACCATCCATAAAGGAAAAGAATT

>Marker11314

ATATTTGTCCGACGTGCATTATGGTGCATCGACAGAAAGAACCTCCTCCAACGTCATACTTCCTACGTCGAAAGAAACCCXXXXXXXXXXCATTTGGGGGCAGACCCTTGAGAACCATAGTAGTATCGTCCACTTGTAAGAGTAGAGGGGAAAACTTCCACTATAGATAA

ATATTTGTCCGACGTGCATTATGGTGCATCGACAGAAAGAACCTCCTCCAACGTCATACTTCCTACGTCGAAAGAAACCCXXXXXXXXXXCATTTGGGGGCAGACCCTTGAGAACCATAGTAGTATCGTCCACTTGTAAGGGTAGAGGGGAAACCTTCCACTATAGATAA

>Marker11523

TTACAAATATGGACATGTGCAGATGGAGGAGATCGAAAAGCTAGTCACTGAAATGCTTCCTGCAGGGGTGATTAGACCGAXXXXXXXXXXAACTGGATCTGAAATCTGGTTATCACCAGATAAGAATGAGAGACGAGGACATAGAAAAAACAGCTTTTCGCACACATGAG

TTACAAATATGGACATGTGCAGATGGAGGAGATCGAAAAGCTAGTCACTGAAATGCTTCCTGCAGGGGCAATTAGACCGAXXXXXXXXXXAACTGGATCTGAAATCTGGTTATCACCAGATAAGAATGAGAGACGAGGACATAGAAAAAACAGCTTTTCGCACACATGAG

>Marker11537

ACCAAAGTTTCCATTCGAATAGTTTAATTTGAAGATTTCAATTCCATTCAAGATCACGTTAGTGAATCTACTCCGGGAGTXXXXXXXXXXCTATCACCTCTAGAATTGATTTCTTCCTGAAACTCACAAAAATGAAGACGAATCATGTAAAAGAATCCAGGGTCTACCGG

ACCAAAGTTTCCATTCGAATAGTTTAATTTGAAGATTTCAATTCCATTCAAGATCACGTTAGTGAATCTACTCCGGGAGTXXXXXXXXXXCTATCACCTCTAGAATTGATTTCTTCCTGAAACTCACAAAAATGAAGACGAATCATGTAAAAGAATCCAGGGGCTACCGG

>Marker12033

GATTCTTAACGATCAAGCAGGAGACTACAATGGAGGAGTATCGAAACCAATTCGATAAGATGGCACCGGTAGCATTCCTTXXXXXXXXXXGGAGGGAAATCAAATTTCAATTCTAACATTGAAGCCATCTTCAATAGTGACGACCAGCAAAGTGCAAACAGGGGGAAGTT

GATTCTTAACGATCAAGCAGGAGACTACAATGGAGGAGTATCGAAACCAATTCGATAAGATGGCGCCGGTAGCATTCCTTXXXXXXXXXXGGAGGGAAATCAAATTTCAATTCTAACATTGAAGCCATCTTCAATAGTGACGACCAGCAAAGTGCAAACAGGGGGAAGTT

>Marker12071

TAACAACTTAGTAACTTTTGTAAGTGTAACTCGCCATTTCGAATCATATAGATCATAATCAATATGCTTCTGAAAGTGGAXXXXXXXXXXCTTCCTATGACATACTTCCCTTATTCACCTTGATATTGATCCATGCAAACACTCTCTGAAGGGAGTCCGCTTCTATGCAC

TAACAACTTAGTAACTTTTGTAAGTGTAACTCGCCATTTCGAATCATATAGATCATAATCAATATGCTTCTGAAAGTGGAXXXXXXXXXXCTTACTATGACATACTTCCCTTATTCACCTTGATATTGATCCATGCAAACACTCTCTGAAGGGAGTCCGCTTCTATGCAC

>Marker12487

TTCATTGTTATCATATTTCTACCTATATAGCCTTGAAGCTTAAACGTAGTTGTTTGTATTATGTTTCTTTTATATATATAXXXXXXXXXXAGGAAGGGGTCCCATGTGTGTTGAATAGGATGGAGAATTGAGAGACAATGGAGAGGACATCCCTTTCTGATAGTGAAAGG

TTCATTGTTATCATATTTCTACCTATATAGCCTTGAAGCTTAAACGTAGTTGTTTGTATTATGTGTCTTTTATATATATAXXXXXXXXXXAGGAAGGGGTCCCATGTGTGTTGAATAGGATGGAGAATTGAGAGACAATGGAGAGGACATCCCTTTCTGATAGTGAAAGG

>Marker12502

CATACTTATTGCCTATATGTTCATCTTCTTTCCAATGATTGAAGGTAGAGTATCAAGACCAATTGCCAACTGAAATTCTGXXXXXXXXXXCGAATTAATTGCACATCCTTTACAACAAACTCGGCTGCCCCATTCTGATCTTCACTATTGAGGACTGAGCATACATCACT

CATACTTATTGCCTATATGTTCATCTTCTTTCCAATGATTGAAGGTAGAGTATCAAGACCAATTGCCAACTGAAATTCTGXXXXXXXXXXCGAATTAATTGCACATCCTTTACAACAAACTCGGCTGCCCCATTCTGATCTTCACTATTGAGGACTGAACATACATCACT

>Marker12616

TATTCCTTTCTTCTTGGAATCAGTATATCTCTTTTTCTCATGTAGAACAATAGAATCATCTTTACCCTCTAGAGACAAGAXXXXXXXXXXTATTTCGCCTTGACTTCACTGAATTTTACAGCCAATTACGACCTCATGTGTAGAGTGGATGTTAGATGTATATTTCTCAT

TATTCCTTTCTTCTTGTAATCAGTATATCTCTTTTTCTCATGTAGAACAATAGAATCATCTTTACCCTCTAGAGACAAGAXXXXXXXXXXTATTTCGCCTTGACTTCACTGAATTTTACAGCCAATTACGACCTCATGTGTAGAGTGGATGTCAGATGTATATTTCTCAT

>Marker12704

ATAGATCAAAGCCATAAACAACTTGCAAGCGGGAGGGAGGTGGGGGTCAGAGACGTGTTACAATGATGCGAAGTTAATGAXXXXXXXXXXTCCCGCCACGAGATGTTCATCAGTAAACAATCGCAACAATATACGTTACACACAGATCCAACGAGACTAAAACATTTAAA

ATAGATCAAAGCCATAAACAACTTGCAAGCGGGAGGGAGGTGGGGGTCAGAGACGTGTTACAATGATGCGAAGTTAATGAXXXXXXXXXXTCCCGCCACGAGATGTTCATCAGTAAACAATCGCAACAATATACGTTACACACAGATCCAACGAGACTAAAACATTAAAA

>Marker12797

TTCTATTTCTAAGACTTCCCAAGACTTTTTGTTACTACTCTTTCTTGTCCCCTTTCCATAGAGAGGCTACAGGCTTCCGTXXXXXXXXXXACAAGTGGAAGCAAAATCAGTATTTTGAGGTCTCAGATGCAAATAGTTCGAGTGTATTAAGAAACTACTAGAGTATTAGA

TTCTATTTCTAAGACTTCCCAAGACTTTCTGTTATTACTCTTTCTTGTCCCCTTTCCATAGAGAGGCTAGAGGCTTCCGTXXXXXXXXXXACAAGTGGAAGCAAAATCAGTATTTTGAGGTCTCAGATGCAAATAGTTCGAGTGTATTAAGAAACTACTAGAGTATTAGA

>Marker12838

AGTGAATTGGTTAATTGGAAAGGCATAACCATGAATTTGTAATGTCCTTCGTGTGTGCCAAAAGTTATCTTCTCAATATCXXXXXXXXXXGTAGGGGCTTTGACTAAGTCTGATGATTCCTGCTTGCAACATTTTATTAACTAGTCTTTCAATTTCTTCCTTTTGGATGT

AGTGAATTGGTTAATTGGAAAGGCATAACTATGAATTTGTAATGTCCTTTGTGTGTGCGAAAAGTTATCTTCTCAATATCXXXXXXXXXXGTAGGGGCTTTGACTAGGTCTGATGATTTCTGCTTGCAACATTTTATTAACTAGTCTTTCAATTTCTTCCTTTTGGATGT

>Marker12865

ATAACTATTATTCAAAATTCAAGCCTCAATATATTCAAGTCATACTTAAATTTAGCATTTTGATATCCAAAACATATGATXXXXXXXXXXGAAATTATTGTCCTTCCGAGACTTGGTTGGTTTCCAAATTTCTCTTGCGATGGAAGCAAGGTTGTAACTACAATTTTTAT

ATAACTATTATTCAAAATTCAAGCCTCACTATATTCAAGTCATACTTAAATTTAGCATTTTGATATCCAAAACATATGATXXXXXXXXXXGAAATTATTGTCCTTTCGAGATTTGGTTGGTTTCCAAATTTCTCTTGCATTGGAAGCAAGGTTGTAACTACAATTTTTAT

>Marker12885

AAAATTATATAATCACATTAACAATCTCTTCTCTCCACCCTAATTACATAAGAATTTTCTCAAAGTAGTATACAGTATAAXXXXXXXXXXTGAAGTTATAAAGGAGAGCACAGAAAGAAGCGGTAAGATTATCCAATTGGTATGTAGTCGAAAACCTAATACTTTGAAAG

AAAATTATATAATCACATTAACAATCTCTTCTCTCCACCCTAATTACATAAGAATTCTCTCAAAGTAGTATACAGTATAAXXXXXXXXXXTGAAGTTATAAAGGAGAGCACAGAAAGAAGCGGTAAGATTATCCAATTGGTATGTAGTCGAAAACCTAATACTTTGAAAG

>Marker13099

AGAGCAGAAATCAAGCCACGACAAGAGGCGAGAAGAACGGGAACATCAATCACAGAAACGCACGCACAGAAACGCGTAAAXXXXXXXXXXGTTAAACTCATGTAAGTTGAAGAAAGAAAAAAAAGAAAGGAACATATGATTCAGCAGCGTGGTTTTGACTCCAATTCCAT

AGAGCAGAAATCAAGCCACGACAAGAGGCGAGAAGAACGGGAACATCAATCGCAGAAACGCACGCACAGAAACGCGTAAAXXXXXXXXXXGTTAAACTCATGTAAGTTGAAGAAAGAAAAAAAAGAAAGGAACATATGATTCAGCAGCGTGGTTTTGACTCCAATTCCAT

>Marker13203

CCTAAAAAGATGTGAACTAAAGTAGGGAAGTCAGAAGAATAACGGGGTTTTGTCATTTTTTGTAGTGAGGAAAAGAAGAAXXXXXXXXXXAACAAGAGGGAATATATGATTGGGGAATAGTTGTTGGTCCATTTGGAAGTGGACATGACTTGATTGATTGAGCCTTTGAG

CCTAAAAAGATGTGAACTAAAGTAGGGAAGTCAGAAGAATAATGGGGTTTTGTCATTTTTTGTAGTGAGGAAAAGAAGAAXXXXXXXXXXAAAAAGAGGGAATATATGATTGGGGAATAGTTGTTGGTCCATTTGGAAGTGGACATGACTTGATTGATTGAGCCTTTGAG

>Marker13319

TTTCCTTTTCATATTCCAATTGGTGAAGACAAACAGGGGTAGAGAACTTTTTGTAAAATGCTTGGCAGATTGGAAGCAAGXXXXXXXXXXAATCTATAGGTAGTATTGAATTTTTTCAGCTGCTGTTTTGGTTGTTTTTCTTGCCTTGATGGGAGTTTGTCATGTTCTTA

TTTCCTTTTCATATTCCAATTGGTGAAGACAAACAGGGGTGGAGAACTTTTTGTAAAATGCTTGGCAGATTGGAAGCAAGXXXXXXXXXXAATCTATAGGTAGTATTGAATTTTTTCAGCTGCTGTTTTGGTTGTTTTTCTTGCCTTGATGGGAGTTTGTCATGTTCTTA

>Marker13338

CTCCACAAGCAAATAGCAAAAAAGAAGTTGAGAGCTCTAACCTTATTTCAAAGAGCACAAATCAGGCGATTCATCCCTATXXXXXXXXXXGATAGCAACTATGGTTCATAAAACGAGCAACATTGCCAGCATTTTTTGCACTAATAACTAGGGGAAATTGGAGTTTTGGA

CTCCACAAGCAAATAGCAAAAAAGAAGTTTAGAGCTCTAACCTTATTTCAAAGAGCACAAATCAGGCGATTCATCCCTATXXXXXXXXXXGATAGCAACTATGGTTCATAAAACGAGCAACATTGCCAGCATTTTTTGCACTAATAACTAGGGGAAATTGGAGTTTTGGA

>Marker13352

AAAGAAGCTCAAGGGCTAGAGATCGATAAGTCCCATAAAAATATCCTATAAATTACAATAATATTTTGTTACGGCTTACTXXXXXXXXXXCAATAGAAAAAATAAGTTATGTAAAGATCTCCAATTTTTTCTCTCATTTTGTCAATGTATGATGGGGTCGTTAACACGGT

AAAGAAGCTCAAGGGCTAGAGATCGATAAGCCCCGTAAAAATATCCTATAAATTACAATAATATTTTGTTACGGCTTACTXXXXXXXXXXCAATAGAAAAAATAAGTTATGTAAAGATCTCCAATTTTTTCTCTTATTTTGTCAATGTATGATGGGGTCGTTAACACGGT

>Marker14439

ACCAAATATAAATCACTTTCTCATCCTTATTAATGATGTGTTTAAAGGTATTTATATACATGAAAGAATAATTAGTTACAXXXXXXXXXXTCGTGGTTTAAGATTGCATACATAGTTTGGTTTTGATCTTACAATTATATGAAACAGCAAAGAGATGTGAAGATTCTGTT

ACCAAATATAAATCACTTTCTCATCCTTGGTAATGATGTGTTTAAAGGTATTTATATACATGAAAGAATAATTAGTTACAXXXXXXXXXXTCGTGGTTTAAGATTGCATACATAGTTTGGTTTTGATCTTACAATTATATGAAACAGCAAAGAGATGTGAAGATTCTGTT

>Marker14447

TCCCTTATGATTTAGTTCACAGGTAATTAATTGAATTTAATTAACGCGAAGTTAATAACGGAATGGAATTATTGACAATGXXXXXXXXXXTGCAGTAACTAATGATAAAGAGCTGCAAGCTTGGTGGAAAGAAATCAAAGACAAAGGGCACCCAGATATAAAGGAAGGAT

TCCCTTATGATTTAGTTCACAGGTAATTAATTGAATTTAATTAACGCAAAGTTAATAACGGAATGGAATTATTGACAATGXXXXXXXXXXTGCAGTAACTAATGATAAAGAGCTGCAAGCTTGGTGGAAAGAAATCAAAGACAAAGGGCACCCAGATATAAAGGAAGGAT

>Marker14910

AAATATTAGGTTCATTTACGCCTTTTTTTAATGAAACTGATCCATCAAGGTTGTGTGAATGGCATCGATCCAAGCAAAGTXXXXXXXXXXAGTTATGATGTCTGACTAACTGTGCCTGATTTCTCAAGATAATGAAAAATGGGGTTTTAGAGAGGATTGAATAGAGCTGT

AAATATTAGGTTCATTTACGCCTTTTTTTAATGAAACTGATCCATCAAGGTTGTGTGAATGGCATCGATCCAAGCAAAGTXXXXXXXXXXAGTTATGATGTCTGACTAACTGTGCCTGATTTCTCAAGATAATGAAAAATGGGGTTTCAGAGAGGATTGAATAGAGCTGT

>Marker14942

TTATATGATCAAACGAATTAAAACCATCTGAAGCTAACCCCAAACGAACGTTTCATTCATCTGCAACGAAATCAAGGAATXXXXXXXXXXAGACTAACCTTGAACTGAGACTGACCACAAGTTGGACAAAACTGCAAATCCTCAAACTCCTTCCGGTACAGGACACAGTC

TTATATGATCAAACGAATTAAAACCATCTGAAGCTAACCCCAAACGAACGTTTCATTCATCTGCAACGAAATCAAGGAATXXXXXXXXXXAGACTAACCTTGAACTGAGACTGACCACAAGTTGGACAAAACTGCAAATCCTCAAACTTCTTCCGGTACAGGACACAGTC

>Marker14969

AAAATAATAGCAGCATCCGTCAAACCTCTTGTGATCCACTAACACAACTCAAACCAAAACAATTCCACATTTGTCAACAAXXXXXXXXXXTATTTTTGCTTCCTCGCTGCTCACACTAAAAGGAGAGTCGAATCTGATTCTTCTTTTTTGATGAAGCGAATGTAGTTTGG

AAAATAATAGCAGCATCCGTCAAACCTCTTGTGATCCACTAACACAACTCAAACCAAAACAATTCCACATTTGTCAACAAXXXXXXXXXXTATTTTTGCTTCCTCGCTGCTCACACTAAAAGGAGAGTCGAATCTGATTCTTCTTTTTTGATGAAGCGAATGGAGTTTGG

>Marker14974

TTGTCTAAGGCTATAAATTAATATTCCATCACAAACTTATAATCATTCATTCAAAAGATCATAAAATTTCCAACCATAAAXXXXXXXXXXTTTATTGGCTTCTCGTTGGACATTATTGCAATAGCATGAATTTTCAATAGTTGTCCAAAGTCAAAGAATATTGCAATTAT

TTGTCTAAGGCTATAAATTAATATTCCATCACAAACTTATAATCATTCATTCAAAAGATCATAAAATTTCCAACCATAAAXXXXXXXXXXTTTGTTGGCTTCTCGTTCGACATTATTGCAATAGCATGAATTTTCAATAGTTGTCCAAAGTCAAAGAATATTGCAATTAT

>Marker15025

TGCACTCGTCGTTTCCTCCTCGACCCTTATATTAGATAAATCATCTAATATGTTCTCTTCGCGCCTTACCCATTTTTTGAXXXXXXXXXXAGACAGAAAGACCATTGAGGCATACACTGACTCGGATTGGGCAGGATCTGTTGTTGACAGAAAATCTACCTCTGGTTATT

TGCACTCGTCGTTTCCTCCTCGACCCTTATATTAGATAAATCATCTAATATGTTCTCTTCGCGCCTTACCCATTTTTTGAXXXXXXXXXXAGACAGAAAGACCATTGAGGCATACACTAACTCGGATTGGGCAGGATCTGTTGTTGACAGAAAATCTACCTCTGGTTATT

>Marker15251

TAACAATAATCTGTTTAATACCCTAAACCCTAACCCTTTGTGTAATAGGGGTTGTATTTGGGGGCTTATGCGAGCAACAAXXXXXXXXXXCAACGTCTGAACAAAGGCTTGGTATGTTTGATTAAATAGATGGAAGAGAAATTATAAAAAAGTATATATATTGACTGTGG

TAACAATAATCTGTTTAATACCCTAAACCCTAACCCTTTGTGTAATAGGGGTTGTATTTGAGGGCTTATGCGAGCAACAAXXXXXXXXXXCAACGTCTGAACAAAGGTTTGGTATGTTTGATTAAATAGATGGAAGAGAAATTATAAAAAAGTATATATATTGACTGTGG

>Marker15292

TGATGTTACTCTCTAATTTGGTCTCACTGTTGTTCTGTATTTGCAGCAAGCATGCCATTGACTAAGCAGTTGACTATTGCXXXXXXXXXXATTATTGAATAACACTGTTATCTGGGCTAACTTTGTCAGAAAGTCTGAAAGCATATCATTTTTATTTCATATTTTTTGTT

TGATGTTACTCTCTAATTTGGTCTCACTGTTGTTCTGTATTTGCAGCAAGCATGCCATTGACTAAGCAGTTGTCTATTGCXXXXXXXXXXATTATTGAATAACACTGTTATCTGGGCTAACTTTGTCAGAAAGTCTGAAAGCATATCATTTTTATTTCATATTTTTTGTT

>Marker15304

CAATGGCTTTCGCAATATACCCAAAAGTGTTCACAGTAGCCCGACGGATACCCTTCTTGTGAGCTTTAAGCATCTCAAGCXXXXXXXXXXAGCCTTGAGAGCTCCCAAAATTGAACCCAGGACCTCCGGGTATTCTTCTCCCAAATATTCATACAAGACAACGCCAAGAT

CAATGGCTTTCGCAATATACCCAAAAGTGTTCACAGTAGCCCGACGGATACCCTTCTTGTGAGCTTTAAGCATCTCAAGCXXXXXXXXXXAGCCTTGAGAGCTCCCAAAATTGAACCCAGGACCTCTGGGTATTCTTCTCCCAAATATTCATACAAGACAACGCCAAGAT

>Marker15575

CATAGGAAGAATTGAGAAAAGCAGAACGTAAAAACCGTGACGAGTTTCGCAAGATGATGGAAGAACACATTGCTGCTGGGXXXXXXXXXXCTCCAAAAGATTTGTTTGAAGATGTTGCGGAAGAGCTACAAAAACAAGTTAATAAACTACATCTTTTGATCTCTTTGCGA

CATAGGAAGAATTGAGAAAAGTAGAACGTAAAAACCGTGACGAGTTTCGCAAGATGATGGAAGAACACATTGCTGCTGGGXXXXXXXXXXCTCCAAAAGATTTGTTTGAAGATGTTGCGGAAGAGCTACAAAAACAAGTTAATAAACTACATCTTTTGATCTCTTTGCGA

>Marker15912

AACTTAATCCTCTTAATTATAAAAAAGTTGCTAACAATATATTCTAAAAAGTATACATTTAAAACTAATAAAATTATATAXXXXXXXXXXTAAGTAAAAATCATATTAAGATATTAGTTCTAAAAATGTTTATAGAATTAAACTCTTCATTTTAAAACACTTTATAAAAA

AACTTAATCCTCTTAATTATAAAAAAGTTGCTAACAATATATTCTAAAAAGTATACATTTAAAACGAATAAAATTATATAXXXXXXXXXXTAAGTAAAAATCATATTAAGATATTAGTTCTAAAAATGCTTATAGAATTAAACTCTTCATTTTAAAACACTTTATAAAAA

>Marker15992

GGAGTTAATGGCTTGGCTTGCTGACAAACAGAACATTTTGCAACATAGGTGCAGGTATGTGCGCTAATAGACCTCCCTTGXXXXXXXXXXCCGAATACTATTAAGAGATTCATTCCCAACCACTTGATCAATGAAAACCGAAGGATTTAGCCCACCCACAACACTTAGGA

GGAGTTAATGGCTTGGCTTGCTAACAAACAGAACATTTTGCAACATAGGTGCAAGTATGTGCGCTAATAGACCTCCCTTGXXXXXXXXXXCCGAATACTATTAAGAGATTCATTCCCAACCACTTGATCAATGAAAACCGAAGGATTTAGCCCACCCACAACACTTAGGA

>Marker16012

ACCTTCAGCTTCGGGTCCTCCTGCATAGATAGTCTCAGACATGTTCCTCAATGTAGCATTTCCACCGAGCAGGTCAGAAGXXXXXXXXXXCCAAGCAATCATTCACATGCTCTGAAACATTGAGAACCAGATGCCCATAAGTCCCACCGTGATTTTGAGGAAGATAGAAT

ACCTTCAGCTTCGGGTCCTCCTGCATAGATAGTCTCAGACATGTTCCTCAATGTAGCATTTCCACCGAGCAGGTCAGAAGXXXXXXXXXXCCAAGCAATCATTCACATGCTCTGAAACATTGAGAACTAGATGCCCATAAGTCCCACCGTGATTTTGAGGAAGATAGAAT

>Marker16261

TACTGAAGATTGAAGGGTGTAAGGAAGGTCGAAAAGGAGCGGTATGTATTGATGCAGAGATTTTTGAAGTTACCCCATCAXXXXXXXXXXACATGAAGCTCAACAACAATCTTCTAGTGATTCATTATAAATAACGCAGGACTGGTGGAGGCGTCTTCCTAGAGTATGAG

TACTGAAGATTGAAGGGTGTAAGGAAGGTCGAAAAGGAGCGGTATGTATTGATGCAGAGATTTTTGAAGTTACCCCATCAXXXXXXXXXXACATGAAGCTCAACAACAATCTTCTAGTGATTCATTATAAATAACGCAGGACTGGTGGAGGCGTCTTCCTAGACTATGAG

>Marker16902

TTTTCGATAGAACAATGGAACCAAAATGCATGATAATGTGTCAAAACGCGTAGCTTCTATTACAGGTTCATAATTATAAGXXXXXXXXXXTCAGAAGAATAGAAGTTTGCCGCCGAAAGTAGTCTATAAAAGGACTTCATTCCTACCATGTATTTTGGTTGGCTTGAATG

TTTTCGATAGAACAATGGAACCAAAATGCATGATAATGTGTCAAAACGCGTAGCTTCTATTACAGGTTCATAATTATAAGXXXXXXXXXXTCAGAAGAATAGAAGTTTGCCGCCGAAAGTAGTCTATAAAAGGACTTCATTCCTACCATGTATTTTGGCTGGCTTGAATG

>Marker16937

TGGTGGGAAGGAGGAGAGATCACATCCCTCATCAAGCAAAGCTTCTCTTACCCTTAGCCGGTCAACAAAATTCCTGTAAAXXXXXXXXXXGCTTCAAAGATACTTCGACATCAGAAATCAAGGGAAGGTTCTTCACTTCAATCCAACCTCCATAGCTTTTCAACATCAGT

TGGTGGGAAGGAGGAGAGATCACATCCCTCATCAAGCAAAGCTTCTCTTACCCTTAGCCGGTCAACAAAATTCCTGTAAAXXXXXXXXXXGCTTCAAAGATACTTCTACATCAGAAATCAAGGGAAGGTTCTTCACTTCAATCCAACCTCCATAGCTTTTCAACATCAGT

>Marker17000

CTTGAACCTCTTGTTGCGATTTTTGGCTAATTGCGTGATTCACCAAGATCAATTACTTTGCCCAAACTCAAAGTAAGACTXXXXXXXXXXATGAGTCATGGGTCATGATCATAGCATGTAAGATGCTCACTATGTGCCTCTTCATCGCATGATGCATAAGTTCTTGTTGA

CTTGAACCTCTTCTTGCGATTTTTGGCTAATTGCGTGATTCACCAAAATCAATTACTTTGCCCAAACTCAAAGTAAGACTXXXXXXXXXXATGAGTCATGGGTCATGATCATAGCATGAAAGATGCTCACTATGTGCCTCTTCATCACATGATGCATAAGTTCTTGTTGA

>Marker17131

TTCCTTGACAACTTTATAGAATAACACCTTCTTTGATCCTTTCAATCTTATTTATACTAATCCTTTACCTTGATTAGTCAXXXXXXXXXXCTTTAAATGTCGCCCCGACTCATCCTCAGGATGGCTCGAGATCTCTTTTGACTTTCTTCCCTTCGTTCACGACCGAACTT

TTCCTTGACAACTTTATAGAATAACACCTTCTTTGATCCTTTCAATCTTATTTATACTAATCCTTTACCTTGATTAGTCAXXXXXXXXXXCTTTAAATGTCGCCCCGACTCATCCTCAGGATGGCTTGAGATCTCTTTTGACTTACTTCCCTTCATTCACGACCGAACTT

>Marker17143

AATTTTTGACTATTAAACAGAAATTCATAGTGGAAGAATATCGCAATTTATTTGATTAAGTTTTAGCTCCTGTAGCCTTTXXXXXXXXXXCAAATATACACCAGGGGGAAATACACCTATTAGAACGATAACATTGAAAGGGGTTATGCCGGGGGAGAACTGAAGGGAAG

AATTTTTGACTATTAAACAGAAATTCATAGTGGAAGAATATTGCAATTTATTTGATTAAGTTTTAGCTCCTGTAGCCTTTXXXXXXXXXXCAAATATACACCAGGGGGAAATACACCTATTAGAACGATAACATTGAAAGGGGTTATGCCGGGGGAGAACTGAAGGGAAG

>Marker17198

TTTCTCTTTTGGTAACCAATATAAAATTATCAAATACATCTTTTGGAAGCTGTAATTCCACCTTGCCCCCTCTTATCTTAXXXXXXXXXXTGTAATTAACATATGTATACTTTTTTTGCATTTCCTAGACAATGGAATTTACAAATTTTACAATAAGATTTCTAGGTAAA

TTTCTCTTTTGATAACCAATATAAAATTATCAAATACATCTTTTGGAAGCTGTAATTCCACCTTGCCCCCTCTTATCTTAXXXXXXXXXXTGTAATTAATATATGTATACTTTTTTTGCATTTCCTAGACAATGGAATTTACAAATTTTACAATAAGATTTCTAGGTAAA

>Marker17241

TTTTGTAATTATCAGATAGGTAAGTGCAAATCTTCCAAATACAATGTGACCCCTGCAACTAAGGATTGAACAACTCACATXXXXXXXXXXGTTATATTTATTAGCTATAATAATAATCAGTTAGGGAGTGAGTTTTATATTTGTTGGTTACAAACCCAGCTTCCCTGTAA

TTTTGTAATTATCAGATAGGTAAGTGCAAATCTTCCAAATACAATGTGACCCCTGCAACTAAGGATTGAACAATTCACATXXXXXXXXXXGTTATATTTATTGGCTATAATAATAATCAGTTAGGGAGTGAGTTTTATATTTGTTGGTTACAAACCCAGCTTCCCTGTAA

>Marker17290

ATAAATGCATAAATTCATTAACCATAAACTTCTTTCTAGGCTTGTAGACTTCTAGACTCCTAGTATAAAGTTGATATATAXXXXXXXXXXTCCAAACCAGTTTTACCATTCTCCCAATCCTAGCAATTAGCTGGATAAGGTTTTACAAACTTGGGAGGAGCCCAAGAGAT

ATAAATGCATAAATTCATTAACCATAAACTTCTTTCTAGGCTTGTAGACTTCTAGACTCCTAGTATAAATTTGATATATAXXXXXXXXXXTCCAAACCAGTTTTACCATTCTCCCAATCCTAGCAATTAGCTGGATAAGGTTTTACAAACTTGGGAGGAGCCCAAGAGAT

>Marker17321

TAGGAGAACTCCCCCAAACATCCCAAGAGTCATCCTGGGTGCAAATGCTATAAAAATTTGAGAATCAATACCCTCAAAGAXXXXXXXXXXAAGAAATTTGAACTAAATACCTGGGAGAACATCTCAAGTGGGGAGTTTTCTTAAACTACAACTTGTCCCTTGAACTACTG

TAGGAGAACTCCCCCAAACATCCCAAGAGTCATCCTGGGTGCAAATGCTATAAAAATTTGAGAATCAATACCCTCAAAGAXXXXXXXXXXAAGAAATTTGAACTAAATACCTGGGAGAACATCTCAAGTGGGGAGTTCTCTTAAACTACAACTTGTCCCTTGAACTACTG

>Marker17424

TTCCTTTATTGAGGTATCAATCAAATTAATCTGGGAGGAATTTAAATGACTATTTAATGGCAATGTGACTTTTGGTTTCTXXXXXXXXXXGAAGTTACTTGCACTAAAATGCAAGCTCCAACCCGTTCCACTAAGTCATATTCTCTTTCCTCTCATCCTACGTAGTTTAA

TTCCTTTATTGAGGTATCAATCAAATTAATCTGGGAGGAATTTAAATGACTATTTAATGGCAATGTGACTTTTGGTTTCTXXXXXXXXXXGAAGTTACTTGCACTAAAATGCAAGCTCCAACCCGTTCCACTAAGTCAGATTCTCTTTCCTCTCATCCTACGTAGTTTAA

>Marker17546

TTAAAAGCACTTTTACCAACCAACTTATTTTTCTAAGACGTAAAAGCACTTGTCATTATAGTTTATTATCAAAGGTGATTXXXXXXXXXXGTTGATTTTTCATCGAAGTATGTTATTGGAACCAAAACTTGGTCATCAACGTTTGTTATGCACAGTCGGTCGTTGGAGGT

TTAAAAGCACTTTTACCAACCAACTTATTTTTCTAAGATGTAAAAGCACTTGTCATTATAGTTTATTATCAAAGGTGATTXXXXXXXXXXGTTGATTTTTCATCGAAGTATGTTATTGGAACCAAAACTTGGTCATCAACGTTTGTTATGCAAAGTCGGTCGTTGGAGGT

>Marker17782

ATAAAGAAAATTGGTTTCTTGATGGACTTTTCGGTTTCTTTGATGGACAAAAAACATCGAAGTCCCTACCATCAAGAAAAXXXXXXXXXXACCAAAGAAATAGTATCCTACCTCAACTAGTGGAACTTCATCATGTTGTCCACCGATATCTACTTAATTGTAAATTGGGA

ATAAAGAAAATTGGTTTCTTGATGGACTTTTCGGTTTCTTTGATGGACAAAAAACATCGAAGTCCCTACCATCAAGAAAGXXXXXXXXXXACCAAAGAAATAGTATCCTACCTCAACTAGTGGAACTTCATCATGTTGTCCACCGATATCTACTTAATTGTAAATTGGGA

>Marker18322

ATTCATTTTTGTGCATTTGAAGGTGTTTGGTTGTTTGCTGCATACGTAGGAATATAATTGGGTCTTTTCGGCTATGTTTTXXXXXXXXXXAGAACACGCAGACATGCAAAAGAAACCAAGGCTAGGAAAGGAAACACCCTAGAAAAGGGTCTCCAATCTAACAAAAATAA

ATTCATTTTTGTGCATTTGAAGGTGTTTGGTTGTTTGCTGCATACGTAGGAATATAATTGGGTCTTTTCGGCTATGTTTTXXXXXXXXXXAGAACACGCAGACATGCAAAAGAAACCAAGGCTAGGAAAGGAAACATCCTAGAAAAGGATCTCCAATCTAACAAAAATAA

>Marker18776

TAACTTATTGTCTTCAGGTCCTCTAAATTGGGGTCCGTATCCTTTCCAGTTTGAGAATACGTTGCGCCGCCCGTGACACCXXXXXXXXXXCCTAGAGAATGATCACAAAAGTCTTCAAAACCAAAGGCAAGGAGAAACATGAAACCTCACCAAGAACAACACATCACCAT

TAACTTATTGTCTTCAGGTCCTCTAAATTGGGGTCCGTATCCTTTCCAGTTTGAGAATACATTGCGCCGCCCGTGACACCXXXXXXXXXXCCTAGAGAATGATCACAAAAGTCTTCAAAACCAAAGGCAAGGAGAAACATGAAACCTCACCAAGAACAACACATCACCAT

>Marker18821

GTTGATCCTCGACAATTTTGTTGAATGGTTACAAGCAAAGCAGAATTAGAAACCTGATACAATGTATTTTGATGTATACGXXXXXXXXXXATTATGTTCAAATTATCATTTCAAACATTCATGGATTATTCTTCTGCTAAATTATTGAAGTTTCTGCCTCTGCTTTTCGT

GTTGATCCTCGACAATTTTGTTGAATGGTTACAAGCAAAGCAGAATTAGAAACCTGATACAATGTATTTTGATGTATACGXXXXXXXXXXATTATGTTCAAATTATCATTCCAAACATTCATGGATTATTCTTCTGCTAAATTATTGAAGTTTCTGCCTCTGCTTTTCGT

>Marker18830

CAACATATAACCCGTCCATTCAAAATAACTAACATGATAGATTTACAAATAAGAGAAATACTTCGAAAGGCAACTACGGTXXXXXXXXXXAATTTTATTCGAACTAACTATTCAACTCCAATGATAACATGGAACACTATGGAAAATAGATAATTTCAAAATAAAATCAA

CAACATATAACCCGTCCATTCAAAATAACTAACATGTTAGATTTACAAATAAGAGAAATACTTCGAAAGGCAACTACGGTXXXXXXXXXXAATTTTATTCGAACTAACTATTCAACTCCAATGATAACATGGAACACTATGGAAAATAGACAATTTCAAAATAAAATCAA

>Marker19354

AACTCCGTGTTCTAGCAATCACCTCCATTGACAACTTCGACGACCACCTTCCACCATCAACCTGAATTCCCGCAACCACTXXXXXXXXXXCTTTTTACAAAACTATCCAACAACAAATGGAGGCTAATATTTACATTTCAGAAATCTTCCCTCTATTTCCTTTCCTCCTA

AACTCCGTGTTCTAGCAATCACCTCCATTGACAACTTCGAATACCACCTTCCACCATCAACCTGAATTCCCGCAACCACTXXXXXXXXXXCTTTTTACAAAACTATCCAACAACAAATGGAGGCTAATATTTACATTTCAGAAATCTTCCCTCTATTTCCTTTCCTCCTA

>Marker19407

CTTTGTTTGGAAAAATCTTGTAACTTGGGGGAGTAAGAAGTAAAGTGATGTGACCAGAAGCAATGTTGAGGCTGAATACTXXXXXXXXXXTCCCTTCGAGCCAACAAGTTGCTGATGTTCTCACCAAGGGACTTCTCATACCAAACTTCGACTTTTGTGTTAGCAAGTTG

CTTTGTTTGGAAAAATCTTGTAACTTGGGGGAGTAAGAAGTAAAGTGATGTGACCAGAAGCAATGTTGAGGCTGAATACTXXXXXXXXXXTCCCTTCGAGCCAACAAGTTGTTGATGTTCTCACCAAGGGACTTCTCATACCAAACTTCGACTTTTGTGTTAGCAAGTTG

>Marker19485

AAACTCAAAAAAAGAGGTTCACCATAGGATCACACACATAAAGAGATCCCAAACTTGGCTTATACCGTTTTACTATTATTXXXXXXXXXXTGGGACAAATACCATGTTTGAATCAACTCGGTCGCCCTCGTTCGAACTGAAGCCATCACTTCACGAATGAAGACGGAGGT

AAACTCAAAAAAAGAGGTTCACCATAGGATCACACACATAAAGGGATCCCAAACTTGGCTTATACCGTTTTACTATTATTXXXXXXXXXXTGGGACAAATACCATGTTTGAATCAACTCAGTTGCCCTCGTTCGAACTGAAGCCATCACTTCACGAATGAAGATGGAGGT

>Marker19576

CCAAATTTGCAATTTATTTTAAGTGGGGGCGAGCTAGAATTTCATTGGATTTTGTTTCTTCAAGTTTCCTCCTGACATTTXXXXXXXXXXGGTTCTTCCAAAACTCCTCAACCAGGTGACTTTGCAGTATCCATTTGATCTGTAATGTTGATATGTTTTATCTGTAATCT

CCAAATTTGCAATTTATTTTAAGTGGGGGCGAGCTAGAATTTCATTGGATGTTGTTTCTTCAAGTTTCCTCCTGACATTTXXXXXXXXXXGGTTCTTCCAAAACTCCTCAACCAGGTGACTTTGCAGTATCCATTTGATCTGTAATGTTGATATGTTTTATCTGTAATCT

>Marker19827

TTGTGAAGGAGAAGTCGCTGAAGAAATCCTTTCCACCACCCAATGGCTTAATTTTAGCTTTAGAACCTAAGACAGTGCATXXXXXXXXXXTCAACAATCAAACACAGCAAGTTGCAGGCATAGTAGACAATGTTGAACACCAAACTTTTAGCATTAGACCCTATGACAGC

TTGTGATGGAGAAGTCGCTGAAGAAATCCTTTCCACCACCCAATGGCTTAATTTTAGCTTTAGAACCTAAGACAGTGCACXXXXXXXXXXTCAACAATCAAACACAGCAAGTTGCAGGCATAGTAGACAATGTTGAACACCAAACTTTTAGCATTAGACCCTATGACAGC

>Marker19868

ACCACTTGAAGCCAGACATTTTGGGAATGGTCGTAGGGCATCTTTATTACTTTTTGACTGTTCTACATCCTCTTGCTGGTXXXXXXXXXXTGTGAGAACGAAGCTTCGAGTTTTGGATGGAAATGATTTCCATTTCAAATTGTCCGTCTACATGTTGAGCTTAATATCAT

ACCACTTGAAGCCAGACATTTTGGGAATGGTCGTAGGGCATCTTTATTACTTTTTGACTGTTCTACATCCTCTTGCTGGTXXXXXXXXXXTGTGAGAACGAAGCTTCGAGTTTTGGATGGAAATGATTTCCATTTCAGATTGTCCGTCTACATGTTGAGCTTAATATCAT

>Marker20095

TCTTGCTCATGGTTGTGCAAGAGTGCCAGAACGTTCAGGTTTTTATCCTATTTATTTATTTTTTGTTATTGGCGTCGTAAXXXXXXXXXXTAGTTTGTTTCAGCCTGTGTGGTCCCGATTTTCCATCAATGAAAAATTACATTTTTTAAAATATATGAAAAGAAATCCCA

TCTTGCTCATGGTTGTGTAAGAGTGCCAGAACGTTCAGGTTTTTATCCTATTTATTTATTTTTTGTTATTGGCGTCGTAAXXXXXXXXXXTAGTTTGTTTCAGCCTGTGTGGTCCCGATTTTCCATCAATGAAAAATTACATTTTTTAAAATATATGAAAAGAAATCCCA

>Marker20421

TTATAACCAAAATTTTACTTCATATACTCAATTTAAAAATGAGTCTCATAATTTGATACAAACACAGTGATCCAATGCGTXXXXXXXXXXTAACGAATATTGATTAATGTGGTTAATGAGTTTAGGCAATTAATCTCATATTGTTGTAGCTTTTGATCTATAGTCCATTA

TTATAACCAAAATTTTACTTCATATACTCAATTTAAAAATGAGTCTCATAATTTGATACAAACACAGTGATCCAATGCGTXXXXXXXXXXTAACGAATATTGATTAATGTGGTTAATGAGTTTAGACAATTAATCTCATATTGTTGTAGCTTTTGATCTATAGTCCATTA

>Marker20755

TAGTTAGTTTAATAGCTCAAGTTTTAAGTGCATAAGCATGTTGATAGATAATCAAAGATCTTGCCGATTATAAATCTTGTXXXXXXXXXXAGAAATCATATACCAAACAAAATATATGATTTAAATATTTGTAAGAACGAGAGAGGAGACATTGGGAGGACAACTTTTTG

TAGTTAGTTTAATAGCTCAAGTTTTAAGTGCATAAGCATGTTGATGGATAATCAAAGATCTTGCCGATTATAAATCTTGTXXXXXXXXXXAGAAATCATATACCAAACAAAATATATGATTTAAATATTTGTAAGAACGAGAGAGGAGACATTGGGAGGACAACTTTTTG

>Marker21146

CATGTGGTTGCAGGTTGTGACTATGAAAGCAACACCAAATGGGGTTCCAAGGTATGGCTTAGGGTTAGTATATGGATAAAXXXXXXXXXXAAGTGAAGAGATGGGTTATTGGGCTTTTGGAAGTCGCTTTCTCAAAATACAACGGATTCACATTCGGCGTAAGGAATTTA

CATGTGGTTGCAGGTTGTGACTATGAAAGCAACACCAAATGGGGTTCCAAGGTATGGCTTAGGGTTAGTATATGATAAAGXXXXXXXXXXAAGTGAAGAGATGGGTTATTGGGCTTTTGGAAGTCGCTTTCTCAAAATACAACGGATTCACATTCGGCGTAAGGAATTTA

>Marker21167

TTATCTTGATTTTATTTAAAAAGCATTCTTTTCACAAGGTATTACAAACATTAATAAAAAAACCTGCAGTAGTGATCATAXXXXXXXXXXTTAATTCTAAAAGTGCTATATTATTTATTTAAGAAAATATTAAATAAAAAACTTATAATCATCCAAAATCACTTGTGCTA

TTATCTTGATTTTATTTAAAAAGCATTCTTTTCACAAGGTATTACAAACATTAATAAAAAAATCTGCAGTAGTGATCATAXXXXXXXXXXTTAATTCTAAAAGTGCTATATTATTTATTTAAGAAAATATTAAATAAAAAACTTATAATCATCCAAAATCACTTGTGCTA

>Marker21216

ATGTTATAGTCTAACGCTGAAAAACACTAAAACATAATTGTCTCTCTTGTTGACGTTTTTACTATATTGGTAAATATTTTXXXXXXXXXXAAAGCTACAAAACAATAATTGAAATGATTCAAAATGCCAAGTCTCGAGTTGACAACGATTACATCAATGCATACCAACAA

ATGTTATAGTCTAACGCTGAAAAACACTAAAACATAATTGTCTCTATTGCTGACGTTTTTACTATATTGGTAAATATTTTXXXXXXXXXXAAAGCTACAAAACAATAATTGAAATGATTCAAAATGCCAAGTCTCGAGTTGACAACGATTACATCAATGCATACCAACAA

>Marker21523

ACAACCAACCCAGGGGGAGTGAGTTATCTTCAATAAAGTATCAACCACATATACAGGGAGCTTGTTTAAATTAAGTTTCAXXXXXXXXXXAAACTTTAGGTAGTGTAGATTTTCCAAAGAGTTTAAGAGGTTAGAAAGAAAGAAAAATTTTGGTGCAATATCAAAGAATA

ACAACCAACCCAGGGGGAGTGAGTTATCTTCAATAAAGTATCAACCACATATGCAGGGAGCTTGTTTAAATTAAGTTTCAXXXXXXXXXXAAACTTTAGGTAGTGTAGATTTTCCAAAGAGTTTAAGAGGTTAGAAAGAAAGAAAATTTTTGGTGCAGTATCAAAGAATA

>Marker21571

ATTTATTTTAACAAACATTCAGTGGACTTTTTAATAAGGAAATAAAATCTGTTTAATTTCAATGTGTTTCTTTGTGTAGAXXXXXXXXXXTTCATGAAATTTTGGTTATGTTTGGTTGAGAAATTTAAGAAAATTTATAACAAAAATCATGAAACTAATTTGAATATATA

ATTTATTTTAACAAACATTCAGTGGACTTTTTAATAAGGAAATAAAATCTGTTTAATTTCAATGTGTTTCTTTGTGTAGAXXXXXXXXXXTTCATGAAATTTTGGTTATTTTTGGTTGAGAAATTTAAGAAAATTTATAACAAAAATCATGAAACTAATTTGAATATATA

>Marker21707

TGTTATACAAACAACATTTATGAGTGGTTCAAGGTATTTTTTCTGACTCATTGATGATCATTTCAAGAAATGGTCGGGTGXXXXXXXXXXCACGGAGAGAGTTTGATTTCAGTTATTTGATGCAATGGTTCTTGAACATTTGTGAGCAGAAGTTGTTTCTTATATCATGT

TGTTATACAAACAACATTTATGAGTGGTTCAAGGTATTTTTTCTCACTCATTGATGATCATTTCAAGAAATAGTCGGGTGXXXXXXXXXXCACGGAGAGAGTTTGATTTCAGTTATTTGATGCAATGGTTCTTGAACATTTGTGAGCAGAAGTTGTTTCTTATATCATGT

>Marker21977

TCAGATTTTCAAGTAAATTTGTGTGTTCTGTGTCTCTACTTTTAATAATTTTATTAATATAATTGCTTCTATTGAAAGAAXXXXXXXXXXGCCCCCTGTTTTATTTTGTTTTCTGCACTCCCGAGTTTCTTGTTGTTTTCTTCCGGTCCCATACTGTTTTTTGGACCTTT

TCAGATTTTCAAGTAAATTTGTGTGTTCTGTGTCTCTACTTTTAATAATTTTATTAATATAATTGCTTCTATTGAAGGAAXXXXXXXXXXGCCCCCTGTTTTATTTTGTTTTCTGCACTCCCGAGTTTCTTGTTGTTTTCCTCCGGTCCCATACTGTTTTTTGGACCTTT

>Marker22367

TAATTACTATACGTAACTTCATGCACCAGAAGGTATTAGGTATTAGAGCTTACATTACCAGAACAAACCCAAGAACATCAXXXXXXXXXXTTTGGAGGAAGATATGGTAGTTAAGTTGATATCACTTGAATTACCTGGTCATTGATCAAAGCATTTGTTTCTGATGCGTT

TAATTACTATACGTAACTTCATGCACCAGAAGGTATTAGGTATTAGAGCTTACATTACCAGAACAAACCCAAGAACATCAXXXXXXXXXXTTTGGAGGAAGAGATGGTAGTTAAGTTGATATCACTTGAATTACCTGGTCATTGATCAAAGCATTTGTTTCTGATGCGTT

>Marker22502

GCTTCCCTTCTTTCTCTAGGTTAAGTTCACAAGCATGTATATCAATTTTTGCATTCCATTCATGAAGTGACGGAGATGCGXXXXXXXXXXTTTTCTCTACACGAAAAACATTTCCCCGCTCGCTACGTTGGATAATTAGTTTATTACTCCACAAGGTGCTGGGTGGTGGT

GCTTCCCTTCTTTCTCTAGGTTAAGTTCACAAGCATGTATATCAATTTTTGCATTCCATTCATGAAGTGACGGAGATGCGXXXXXXXXXXTTTTCCCTACACGAAAAACATTTCCCCGCTCGCTACGTTGGATAATTAGTTTATTACTCCACAAGGTGCTGGGTGGTGGT

>Marker22544

TATAAAAATGCGATTAAACAACCCAATTCAACAATCCTAAGGGGACACGAGTAAGTGAGAAATTCTAACTTTCTTTTACTXXXXXXXXXXTAACTAATTGACAATGTAGACATAAAATTTAATTAGTTAGTTTATTTGAAAAGTAATATATTGGAGAGAATTTAAAAGAT

TATAAAAATGCGATTAAACAACTCAATTCAACAATCCTAAGGGGACACGAGTAAGTGAGAAATTCTAACTTTCTTTTACTXXXXXXXXXXTAACTAATTGACAATGTAGACATAAAATTTAATTAGTTAGTTTATTTGAAAAGTAATATATTGGAGAGAATTTAAAAGAT

>Marker22771

CAATTCCTTAGAGTTGCTTTTTGGGGTTAAAAGTTATTTTAGCGTCTTTTTCTTGGAGTTACTTTTCCTGTGTCCAACTTXXXXXXXXXXGACCTTGAACTTCTTTTCACCCTCATTTGGGCTATTTAAGAACATAGCAACTTGATTATTTTATAAACAGTTCAGTGAAT

CAATTCCTTAGAGTTGCTTTTTGGGGTTAAAAGTTATTTTAGCGTCTTTTTCTTGGAGTTACTTTTCCTGTGTCCAACTTXXXXXXXXXXGACCTTGAACTTTTTTTCACCCTCATTTGGGCTATTTAAGAACATAGCAACTTGATTATTTTATAAACAGTTCAGTGAAT

>Marker23009

AAGGTTTTCGTTCACACTCTTGGTTGGTCGCTTTGGTCTCTTTGGTTTCTGAGTTTGAGGTTCAAGATTCTGCCGGGACTXXXXXXXXXXATTGGAACAGTGCCGCTTAATTGAATCTATGAATCTACAAACAACAATAATGAACTGTAATAAATGCAACAAAACTTCAT

AAGGTTTTCGTTCACACTCTTGGTTGGTCGCTTTGGTCTCTTTGGTTTCTGAGTTTGAGGTTCAAGATTCTGCCGGGACTXXXXXXXXXXATTGGAACAGTCCCGCTTAATTGAATCTATGAATCTACAAACAACAATAATGAACTGTAATAAATGCAACAAAACTTCAT

>Marker23122

AATGAATAAATTGAATGGAGACGGAAGAATTGAGACAACCTACAATGCCACTATAAATAACATTATTTCACAAAACACCAXXXXXXXXXXTGCAGCATATAGGTTAGACATAACTACATAGTTTCCTATATCATCAGCTTTAGTTTCAAACAGTTTCTCTGCCACAACAA

AATGAATAAATTGAATGGAGACGGAAGAATTGAGACAACCTACAATGCCACTATAAATAACATTATTTCACAAAACACCAXXXXXXXXXXTGCAGCATATAGGTTAGACATAACTACATAGTTTCCTATATCATCAGCTTTAGTTTCAAACAGTTGCTCTGCCACAACAA

>Marker23275

TTTCGTAATTGTTCTTTCTGTTATTAACTTCTAGTAAAACAAACAATACAACAAGAATTAAGTATGTGTTTTCTTAAACGXXXXXXXXXXATAGGAGGGATGGAAGTTGAAACTTTTAAACCTAAGTTATAACTTTAGAAGAAAAAAAATCTAAAAATATAATAATGATC

TTTCGTAATTGTTCTTTCTGTTATTAACTTCTAGTAAAACAAACAATACAACAAGAATTAAGTATGTGTTTTCTTAAACGXXXXXXXXXXATAGGAGGGATGGAAGTTGAAACTTTTAAAGCTAAGTTATAACTTTAGAAGAAAAAAAATCTAAAAATATAATAATGATC

>Marker23330

ACCACCCGCCGGAGTTCATCGTTTTCAAGTCACTGGCAGCCAAATTTCGGAGACACCAAAAACAGAGCAATAGTGATCTAXXXXXXXXXXAGTCTACCACAGGTTTTCATGGGGGGGAAGCACATTGGTGGGGCAGAGGAGATACGGCAAATGAATGAGAGCGGGGAGTT

ACCACCCGCCGGAGTTCATCGTTTTCAAGTCACTGGCAGGCAAATTTCGGAGACACCAAAAACAGAGCAATAGTGATCTAXXXXXXXXXXAGTCTACCACAGGTTTTCATGGGGGGGAAGCACATTGGTGGGGCAGAGGAGATACGGCAAATGAATGAGAGCGGGGAGTT

>Marker23414

CTTTTTCAAACCCTAACCCTAAATTTAACAAGAAACAGTTCCACGGCGAAATGAATGGTTTTCAGATGGATGATTCTCCGXXXXXXXXXXGAAAAGTCGAATCAATTCGGGTGAACTGCATTCCAGACCAAAACATCAGAAAAAATTCTCAAAAACTTTGGGAACTAAGC

CTTTTTCAAACCCTAACCCTAAATTTAACAAGAAACAGTTCCACGGCGAAATGAATGGTTTTCAGATGGATGATTCTCCGXXXXXXXXXXGAAAAGTCGAATCAATTCGGGTGAACTGCATTCCAGACCAAAACATCAGAAAAAATTCTCAAAAACTTTGGGAACCAAGC

>Marker24112

AATTTCTTCCAATTAAAATCAATGATTATTTTTGCTAGACTTTAAAATCAATGAGGGAGGACTATCATCTGGAGCACTTGXXXXXXXXXXGGGTCATTTTACCTTGAAACAAATTAAAGCTGCCACCAACAATTTTGATCCTAAAAGTAAAATAGGCGAAGGTGGATTTG

AATTTCTTCCAATTGAAATCAATGATTATTTTTGCTAGACTTTAAAATCAATGAGGGAGGACTATCATCTGGAGCACTTGXXXXXXXXXXGGGTCATTTTACCTTGAAACAAATTAAAGCTGCCACCAACAATTTTGATCCTAAAAGTAAAATAGGCGAAGGTGGATTTG

>Marker24876

ACATAGTGATGAGAAGCTAGTTAATATTTTGAAAGGAGCGTCTTGAACTTGGTGGTCTGAACAATAATGACGAATCTATAXXXXXXXXXXTTCATAAAAAGGATTTTATAAAACCTCATTAAGTCTTTGGCTTATGTTTTAAAGTTTATCTTCCATGTTTTAGGCGTTAT

ACATAGTGATGATAAGCTAGTTAATATTTTGGAAGGAGCGTCTTGAACTTGGTGGTCTGAACAATAATGACGAATCTGTAXXXXXXXXXXTTCATAAAAAGGATTTTATAAAACCTCATTAAGTCTTTGGCTTATGTTTTAAAGTTTATCTTCCATGTTTTAGGCGTTAT

>Marker25336

TCTTGTGTTGGTGTTTATATAAATAATGGCGTCTAACTTATTTTTTTCTGCACTAGTTCAAATACTTACATAAAACTCTGXXXXXXXXXXCTCTGTGCTTACTTTAATATAACGTGGATTGTAACGCTTCCCAATATTGGTGAAGAGAAGAGGAGAAGAGAGAAGAGAAG

TCTTGTGTTGGTGTTTATATAAATAATGGCGTCTAACTTATTTTTTTCTGCACTAGTTCAAATACTTACATAATCCTCTCXXXXXXXXXXCTCTGTGCTTACTTTAATATAATGTGGATTGTAACGCTTCCCAATATTGGTGAAGAGAAGAGGAGAAGAGAGAAGAGAAG

>Marker25380

TTATGATATTATGGCTCAATTATATTTCGAAATAATAAGTGTTTCATTTTTATATTATCATAATTTATATTTGAATTAAAXXXXXXXXXXTGTAGAAATATAGTCTTTATAGAAATAATTATTGAGTTTATTTGCTATTGTTATTTAATTATGTAATAAGTATATTCTTT

TTATGATATTATGGCTCAATTATATTTCGAAATAATAAGTGTTTCGTTTTTATATTATCATAATTTATATTTGAATTAAAXXXXXXXXXXTGTAGAAATATAGTATTTTTAGAAATAATTATTGAGTTTATTTGCTATTGTTATTTAATTATGTAATAAGTATATTCTTT

>Marker25603

TAGTGGCGAGTTCATTTCACCAGCCTAGTAGGTCGCCGCAAGGTCAAAACTTTTTCACTACTTCCACCTTTGTGCTGAGAXXXXXXXXXXCATTAGATAACATGAATTTTTAACTCAAAACAAATTGACAATGAAAAGAGTAACTCATTTATGTTATACAAGTATTATAA

TAGTGGCGAGTTCATTTCACCAACCTAGTAGGTCGCCGCAAGGTCAAAACTTTTTCACTACTTCCACCTTTGTGCTGAGAXXXXXXXXXXCATTAGATAACATGAATTTTTAACTCAAAACAAATTGACAATGAAAAGAGTAATTCATTTATGTTATACAAGTATTATAA

>Marker26346

TTAATGGTCTTTCTCTTTTTCTTTTTGAGCATCAACCCTTTACGAGGTGTCGTTTCCAAGTATCTCAGGATTCTGTTGACXXXXXXXXXXCCACATCCCAATATACCTGTCTCGGTCAACAAATCAATGGTGTATTTTCTCTGAGATATAGAGATGCCTTCTTTAGATTT

TTAATGGTCTTTCTCTTTTTCTTTTTGAGCATCAACCCTTTACGAGGTGTCGTTTCCAAGTATCTCAGGATTCTGTTGACXXXXXXXXXXCCAGATCCCAATATACCTGTCTCAGTCAACAAATCAATGGTGTATTTTCTCTGAGATATAGAGATGCCTTCTTTAGATTT

>Marker26867

AGCTAGCCTTATATGTGAAATTTTTACACTAGTAGATCAATGACAATATACTGGTTATCATTTACTAATTGCATTTGCCTXXXXXXXXXXCATTTTTCTTACCCCTGTTAATATTTATTCTAAATTTTTGACATTCTTTCTCCAGGGACGTTTTTCTATTTTCAAGAATG

AGCTAGCCTTATATGTGAAATTTTTACACTAGTAGATCAATGACAATATACTGGTTATCATTTACTAATTGCATTTGCCTXXXXXXXXXXCATTTTTCTTACCCCTGTTAATATTTATTCTAAATTTTTGACATTCTCTCTCCAGGGACGTTTTTCTATTTTCAAGAATG

>Marker26916

TTCTTAGCTTTCCCCGTCCGTCATAGAATCTTCTAGAATTTCCTAGGGTCTTTGCGGCTCTTTTGGGTGCGCACTCATCGXXXXXXXXXXGAAAGAAATACATTGAAATCAACGTTATAATATGCATTGAAGACTGTCAGTTACAAGAGAAAAATGTTTGCTTGCAAAAA

TTCTTAGTTTTCCCCGTCCGTCATAGAATCTTCTAGAATTTCCTAGGGTCTTCGCGGCTCTTTTGGGTGTGCACTCATCGXXXXXXXXXXGAAAGAAATACATTGAAATCAACGTTATAATATGCATTGAAGACTGTCAGTTGCAAGAGAAAAATGTTTGCTTGCAAAAA

>Marker27085

CTTCAAATATTCACCATATATTATATAACCATCTAAATTATTACAGAAGTCTTCCAGTAATAATCCCCTCGTCTATTTATXXXXXXXXXXAGATTATAGAGTATTCAAGTGCATTTTTCCCACCTCCTAAGCTTCATTTAGTATTTTACCATCCCAACAAATTAAAATAA

CTTCAAATATTCACCATATATTATATAACCATCTAAATTATTACACAAGTCCTCCAGTAATAATCCCCTCGTCTATTTATXXXXXXXXXXAGATTATAGAGTATTCAAGTGCATTTTTCCCACCTCCTAAGCTTCATTTAGTATTTTACCATCCCAACAAATTAAAATAA

>Marker27159

TCGAATTCAGATTCCAATTCGTCGATTCGTTCCTGACATTCCCCAGCTTGTTCATACAAACAAGACATCCGGGAATGATTXXXXXXXXXXATCCTCTTTGATCTCTTGAAGAAAATGTTCCATTTCTCTCCTCAAATCTACCATCTTCGAAAGTTCATTTTTACTTGCAA

TCAAATTCAGATTCCAATTCGTCGATTCGTTCCTGACATTCCCCAGCTTGTTCATACAAACAAGACATCCGGGAATGATTXXXXXXXXXXATCCTCTTTGATCTCTTGAAGAAAATGTTCCATTTCTCTCCTCAAATCTACCATCTTCGAAAGTTCATTTTTACTTGCAA

>Marker27500

AAGACTATTATGGAGTAATTTGTCCAAAGCACAGCTCATGGAAATATGGTTTGAACGAAACCAAAGGATATTCTACGACAXXXXXXXXXXGTCGGCTTTTGCTTGTGTATTCTTGCAGCTTTAAAATTCTAGTAGCTTTTATGTCCCGTTTTCCTCTGCCTTACTTCGAT

AAGACTATTATGGAGTAATTTGTCCAAAGCACAGCTCATGGAAATATGGTTTGAACGAAACCAAAGGATATTCTACGACAXXXXXXXXXXGTCGGCTTTTGCATGTGTATTCTTGCAGCTTTAAAATTCTAGTAGCTTTTATGTCCCGTTTTCCTCTGCCTTACTTCGAT

>Marker27713

TAAAGATTTAAATTAGCGACCTATAACAATAATAATGATAACGAAGTCAACTTCCTTAAATATGTCAATGTAACATTAGGXXXXXXXXXXAGCGGAAAAACAGAGGGGTTCTAATATCCATTGCCAGAATCTAGATAACATGTTAAATATTACAGGTCCCTTATCAACAT

TAAAGATTTAAATTAGCGACCTATAACAATAATAATGATAACGAAGTCAACTTCCTTAAATATGTCAATGTAACATTAGGXXXXXXXXXXAGCGGAAAAACAGAGGGGTTCTAATATCCATTGCCAGAATCTAGGTAACATGTTAAATATTACAGGTCCCTTATCAACAT

>Marker28473

TAAACTCACTAAAGGTTGGATAAGCAATCTCACTAAAGGTTGGAGAAGCAATCTCACCAAAGGTTGGAAAAAAGCGATCAXXXXXXXXXXTCCATCATAAAAGTAAAATTTCGATAAATACAAACTTCACTTTATATCTTCAATAAAAAAATCAGAGGTTCAAATCCCCT

TAAACTCACTAAAGGTTGGATAAGCAATCTCACTAAAGGTTGGAGAAGCAATCTCACCAAAGGTTGGAAAAAAGCGATCAXXXXXXXXXXTCCATCATAAAAGTAAAATTTCAATAAATACAAACTTCACTTTATATCTTCAATAAAAAAATCAGAGGTTCAAATCCCCT

>Marker28616

AGTTCTAAATCCAGCTGAAGTTCTAATTGCTATTCATGGAATTGATCCTGACAGAGATGGAATTCCTCTCAAGAAGGTTGXXXXXXXXXXACAGCAAGCTGCTTGTCCTTTGCAGGTTGAGCAGATCCCTCTTCCATTATTGTTCATGCGCACAGTATTGCAAGCCATTG

AGTTCTAAATCCAGCTGAAGTTCTAATTGCTATTCATGGAATTGATCCTGACAGAGATGGAATTCCTCTCAAGAAGGTTGXXXXXXXXXXACAGCAAGATGCTTGTCCTTTGCAGGTTGAGCAGATCCCTCTTCCATTATTGTTCATGCGCACAGTATTGCAAGCCATTG

>Marker28836

TTTTTGTGGGCAACTTCAAAACAATCCCAACCATCCAAGGGATTTATCATGAACATGAACACCCTTTGGTTCCTTTCAAAXXXXXXXXXXCCAATGGCATAATGGGCAGATAGAGGGAGAAAGACAACTGGTAGGAAGTTTATGTTGCAAGGCTGAGGATACATTTAGAT

TTTTTGTGGGCAACTTCAAAACAATCCCAACCATCCAAGGGATTTATCATGAAAATGAACACCCTTTGGTTCCTTTCAAAXXXXXXXXXXCCAATGGCATAATGGGCAGATAGAGGGAGAAAGACAACTGGTAGGAAGTTTATGTTGCAAGGCTGAGGATACATTTAGAT

>Marker28839

CTTTCCTTAGAACTAAGGACATAGGGTGAACAGTGAACATTGTTTCTTCTCAACCCCCCCACCCACCAAAAAAAACAAAGXXXXXXXXXXTTAAATCCTAAGTTAGGTGTCAACCATCATTATTTTCTTTTTTCCCCTCTACTTTTTATAACTGTTCTTATTGAACACTA

CTTTCCTTAGAACTAAGGACATAGGGTGAACAGTGAACATTGTTTCTTCTCAACCCCCCCACCCACCATAAAAAACAAAGXXXXXXXXXXTTAAATCCTAAGTTAGGTGTCAACCATCATTATTTTCTTTTTTCCCCTCTACTTTTTATAACTGTTCTTATTGAACACTA

>Marker28940

ACCTTTCTGTTCTTCCTTTGAACATTCTCTTTTCATAGACCCACCTTCAACTTCTTCTTCTCTTACTCCTCTTTTTCTATXXXXXXXXXXCCATGGATCTGTTCCTTACTGAAGCAACTATGTTGATCACTTGATTGTTTGTCAACACATCCCATATCTGCAATTTCATG

ACCTTTCTGTTCTTCCTTTGAACATTCTCTTTTCATAGACCCACCTTCAACTTCTTCTTCTCTTTCTCCTCTTTTTCTATXXXXXXXXXXCCATGGATCTGTTCCTTACTGAAGCAACTATGTTGATCACTTGATTGTTTGTCAACACATCCCATATCTGCAATTTCATG

>Marker29032

AAGAGGAATCTCAATGGATGAGGGTAGTGGCAAATGAAGATGAGAGCCTCTATGAAGTCATCAAAGAAAGTGTCCTCACGXXXXXXXXXXCGAAGAACTTGAACACATGGGTGCATTTGTAATGTATAACTTAGCCAATTTTTTTTTTTTAATTGCAACCTTATTTTAAT

AAGATGAATCTCAATGGATGAGGGTAGTGGCAAATGAAGATGAGAGCCTCTATGAAGTCATCAAAGAAAGTGTCCTCACGXXXXXXXXXXCGAAGAACTTGAACACATGGGTGCATTTGTAATGTATAACTTAGCCAATTTTTTTTTTTGAATTGCAACCTTATTTTAAT

>Marker29034

CCCAAATTGGTTGCAAGGAAAAGCCAAAACCTCTAGCCCTGCCCTCCCAAACAAACACACATACATACATAAGAACGTGAXXXXXXXXXXAATTCTCTTTACTAGGATTGAAATAATTTTTTGAGTGCTTAAGAACTCTTTTCTTACCATCTATAAAAGCCCTTTTGTAA

CCCAAATTGGTTGCAAGGAAAAGCCAAAACCTCTAGCCCTGCCCTCCCAAACAAACACACATACATACATAAGAACGTGAXXXXXXXXXXAATTCTCTTTACTAGGATTGAAATAATTTTTTGAGTGCTTAAGAACTCTTTTTTTACCATCTATAAAAGCCCTTTTGTAA

>Marker29068

CAAATATCTTCCTACCTTATACCAATCCTTGATCTAAGGGTTTTGGCTTGGTCAATGTCCTCACAGCATAGGACAGCCTTXXXXXXXXXXGTGTGGTTGATGAATCCACAAATTCTGGAGAGGGTGCACAGATATGGCAAGTTTCCTTCTTAATAGCAGCAACGTATGAA

CAAATATCTTCCTACCTTATACCAATCCTTGATCTAAGGGTTTTGGCTTGGTCAATGTCCTCACAGCATAGGACAGCTTTXXXXXXXXXXGTGTGGTTGATGAATCCACAAATTCTGGAGAGGGTGAACAGATATGGCAAGTTTCCTTCTTAATAGCAGCAACGTATGAA

>Marker29082

ATGGTAACTAACTCTTGTATAAAATTTTTCCTATAAATAAAGCCTTTCCCTATTTGCGAGAGGCATAATTCATTCATACAXXXXXXXXXXATTCGCATAAGGCAGGAGATGAGTGGAATACTGCTTTTAAAACAAATGAGGAATTATTTGAATGGCTTGTAATGCCTTTT

ATGGTAACTAACTCTTGTATAAAAATTTTCCTATAAATAAAGCCTTTCCCTATTTGCGAGAGGCATAATTCATTCATACAXXXXXXXXXXATTCGCATAAGGCAGGAGATGAGTGGAATACTGCTTTTAAAACAAATGAGGAATTATTTGAATGGCTTGTAATGCCTTTT

>Marker29181

TGTGGGCTTTTTCCTTTGTTAGAATTATTCTATACGATGTAAATATTTTAACGGTTTGTTATATTTTTTAAAAGACCCCTXXXXXXXXXXCACTCAATAACACACAAAAAAAATTCATGTGTTATAATATCATCTCAAAACTCAACAAGGTAATTGCTTATCTTTTGAGT

TGTGGGCTTTTTCCTTTGTTAGAATTATTCTATACGATGTAAATATTTTAACGGTTTGTTATATTTTTTAAAAGACCCGTXXXXXXXXXXCACTCAATAACACACAAAAAAAATTCATGTGTTATAATATCATCTCAAAACTCAACAAGGTAATTGCTTGTCTTTTGAGT

>Marker29211

ACCCTAGGGGGCATATTTTCTTCATCATGAGGTTTGCTTTTTCTCTCTGCTTGTAACTAATTTCCTAGAAGCGAAATTGTXXXXXXXXXXGGAAACTGATGGGAAATAGAAACCTATATTAGACTAAGAAATAGACCACAGTAGTTTCTTTTACCTTTAATTCCGCCTCG

ACCCTAGGGGGCATATTTTCTTCATCATGAGGTTTGCTTTTTCTCTCTGCTTGTAACTAATTTCCTAGAAGTGAAATTGTXXXXXXXXXXGGAAACTGATGGGAAATAGAAACCTATATTAGACTAAGAAATAGACCACAGTAGTTTCTTTTACCTTTAATTCCGCCTCG

>Marker29350

ACCTCAAAACTGTTCTTGTCAGTGCCAACCATAACTCAGAGAAACTCGAAAATATATTCTAAAACAAACAGAAACTGATTXXXXXXXXXXTAAGGATCAAACTAGAATAATTCCGCAATCAGCAACTATTGGCTTTAGATGATCTGGTATTTCGCAAGATTAGGGTAAGG

ACCTCAAAACTGTTCTTGTCAGTGCCAACCATAACTCAGAGAAACTCGAAAATATATTCTAAAACAAACAGAAACTGATTXXXXXXXXXXGAAGGATCAAACTAGAATAATTCCGCAATCAGCAACTATTGGCTTTAGATGATCTGGTATTTCGCAAGATTAGGGTAAGG

>Marker29386

ATCAATGAAGTGGAGATTCAGAAGATAACTTTGGTGATCTACTCATAATAGTCTCTGGAGAGACTTCAAATAGAAAAGAAXXXXXXXXXXGGAGGCGAGAGAAACAAAGGTATCATAAGAAGAACCTCCTTGCCAATAGCAGAAACAAAATTTGATAGGTTGACGAAGAA

ATCAATGAAGTGGAGATTCAAAAGATAACTTTGGTGATCTACTCATAATAGTCTCTGGAGAGACTTCAAATAGAAAAGAAXXXXXXXXXXGGAGGCGAGAGAAACAAAGGTATCATAAGAAGAACCTCCTTGCCAATAGCAGAAACAAAATTTGATAGGTTGACGAAGAA

>Marker29611

ATTATTTTCCTTATCTGGTTTTATTGTCTAGGTTAACTGGAGATTTGATATCTTATTTTTTGTTATTTAACAGCCTGCTTXXXXXXXXXXGGGTGATTGCAAGTTGAAGTATAGAATTCTGAGCGAAGTTCTTCTGATTAAACAGTGATCTTACTCGCAACCATATTAAT

ATTATTTTCCTTATCTGGTTTTATTGTCTAGGTTAACTGGAGATTTGATATCTTATTTTTTGTTATTTAACAGCCTGCTTXXXXXXXXXXGGGTGATTGCAAGTTGAAGTATAGAATTCTGAGCGAAGTTTTTCTGATTAAACAGTGATCTTACTCGCAACCATATTAAT

>Marker29692

CATACTTCCAGACTGGGTCTTTGCGAGTTGCTTTGCTTCTTGGATCCGCATTTCATTTTATTTTCTTTAGGCTTCAGATCXXXXXXXXXXTCCGCAACTTGAGATTGATTGTCAAGCGACATACCTTCGTTTATGATCTCATGTGCTATCTTCTGAATCTCATGCGATTA

CATACTTCCAGACTGGGTCTTTGCGAGTTGCTTTGCTTCTTGGATCCGCATTTCATTTTATTTTCTTTAGGCTTTAGATCXXXXXXXXXXTCCGCAACTTGAGATTGATTGTCAAGCGACATACCTTCGTTTATGATCTCATGTGCTATCTTCTGAATCTCATGCGATTA

>Marker29702

ATATTATTTTAAGACGATCACAGCACCATCTTCTTAATTACAAACAAAGATTACATAAGATTCCACTTTACAATTGAGCCXXXXXXXXXXAATAAAACACCGAGCGATTGAAAAAAAACATCAAAGAGCGGTTGAGGCAAGCAGGCAAGCTATATTTACATAAGATTCAT

ATATTATTTTAAGACGATCACAGCATCATCTTCTTAATTACAAACAAAGATTAGATAAGATTCCACTTTACAATTGAGCCXXXXXXXXXXAATAAAACACCGAGCGATTGAAAAAAAACATCAAAGAGCGGTTGAGGCAAGCAGGCAAGCTATATTTACATAAGATTCAT

>Marker29784

ACCACCGCCGCAAGCATGCCGTATAGCAGAGATCCGATAGGGATGTTTGTGATCAGAATGTTGTGGTTAACGCCTAAGCTXXXXXXXXXXAATCAAATTAGCAGAGGAAATATGTAGGAGAATTAATTTGAGTTGAATCTAGATTGTTACTTACGCTCGAATGTAGTCTG

ACCACCGCCGCAAGCATGCCGTATAGCAGAGATCCGATAGGGATGTTTGTGATCAGAATGTTGTGGTTAACGCCTAAGCTXXXXXXXXXXAATCAAATTAGCAGAGGAAATATGTAGGAGAATTAATTTGAGTTGAATCTAGATTGTTGCTTACGCTCGAATGTAGTCTG

>Marker29990

TTCTACATCTGTATACAATCTTCAACAAAGTGTCAGCACTTGCATACATTTCAGTAGAAGTTGGATTTTTGTCTCTTACGXXXXXXXXXXATTACTTTTCTGTTTAAATATGCATTACATAACATACTGACGTGGACAAAAGAGAAAAGAAACTTGCAATTAGAACTACT

TTCTACATCTGTATACAATCTTCAACAAAGTTTCAGCACTTGCATACATTTCAGTAGAAGTTGGATTTTTGTCTCTAACGXXXXXXXXXXATTACTTTTCTGTTTAAATATGCATTACATAACATACTGACGTGGACAAAAGAGAAAAGAAACTTGCAATTAGAACTACT

>Marker30107

TATTTAGGAAGGTTTTAACAAGTATAAACGACTAAATTTTAACTTTCTTTCAACAAACCAAATTTGTAGTTTAAACTCACXXXXXXXXXXTTGTGCACTGTCCTTTTTATGATGGTTCCTGCACAACACAATGTTTAAATTTAGTAGAACCACTTTTGAAATACTAAAAA

TATTTAGGAAGGTTTTAACAAGTATAAACGACTAAATTTTAACTTTCTTTCAACAAACCAAATTTGTAGTTTAAACTCACXXXXXXXXXXTTGTGCACTGTCCTTTTTATGATAGTTCCTGCACAACACAATGTTTAAATTTAGTAGAACCACTTTTGAAATGCTAAAAA

>Marker30250

AAACCTAGAGGAGATTCTTCTTTGTGGTGTGGACTTTATCACCATTGTTGTTTATGTAAAACTAATGATACATAGTTGGAXXXXXXXXXXAGGAAAACACTCTAATTTAGGTTGAGTGTCCATGTTGGACATAGATTTGTTTAAACACACTTGGAGGACCAAAAATATGT

AAACCTAGAGGAGATTCTTCTTTGTGGTGTGGACTTTATCATCATTGTTGTTTATGTAAAACTAATGATACATAGTTGGAXXXXXXXXXXAGGAAAACACTCTAATTTAGGTTGAGTGTCCATGTTGGACATAGATTTGTTTAAACACACTTGGAGGACCAAAAATATGT

>Marker30369

AATCTTTGTTTACAGCAACGGATAGGACTCTGACAGTATTTAACTTAGCAATAGGAGAAAAAGTTTTTGAATAATCAACAXXXXXXXXXXAAGGAGTGAAAGCTCTGAACTGTGGTGAGAGATTCTTGTATGACACATAATTACATATGGAGTGCTTTGTGCAGAACCTT

AATCTTTGTTTACAGCAACGGATAGGACTCTGACAGTATTTAACTTAGCAATAGGAGAAAAAGTTTTTGAATAATCAACAXXXXXXXXXXAAGGAGTGAAAGCTCTGAACTGTGGTGAGAGATTCTCGTATGACACATAATTACATATGGAGTGCTTTGTGCAGAACCTT

>Marker30749

TATATTGCTCCCCACGACTCAATTCACATACTGCCACCTAATTCCGACCAGCCACCACCTCTTCTATCATCAAATTTGTAXXXXXXXXXXATAACCTCGTTCCCCACTTTATCCTCTTCTTATGTGACTAATACAGTGGCACAGTTTTCACTCATCTCGAAGCTTTTTGT

TATATTGCTCCCCACGACTCAATTCACATACTGCCACCTAATTCCGACCAGCCACCACCTCTTCTATCATCAAATTTGTAXXXXXXXXXXATAACCTTGTTCCCCACTTTATCCTCTTCTTATGTGACTAATACAGTGGCACAGTTTTCACTCATCTCGAAGCTTTTTGT

>Marker31034

ACGTGCTTGAAACTCATTCTCATTTAAAATTTAATCGATGTAAACAAACTATGCTTCTAATATTTTACCAAAATTTAACGXXXXXXXXXXCTTGAATTACATGACCAAACTTGCCCATTGTCCAAACTACAATTATTAAAATCCCGAAGATCAAAATTTAAAAGTAACGA

ACGTGCTCGAAACTCATTCTCATTTAAAATGTAATCGATGTAAACAAAGTATGCTTCTAATATTTTACCAAAATTTAACGXXXXXXXXXXCTTGAATTACATGACCAAACTTGCCCATTGTCCAAACTACAATTATTAAAATCCCGAAGATCAAAATTTAAAAGTAACGA

>Marker31043

CTATTGTATTGTTCATTAGAAAATAATAACAACAAACAATTGTGGTTTTTCTCTTGATACTGAAACTCGGGTTTCACGTAXXXXXXXXXXCCTACAGAAAACGTCCAAAAATAACTTCCTGTCAGTTCTGTAGTCGTCCGCACCGCCGAAATGCCCCAAGCGCCGCCATG

CTATTGTATTGTTCATTAGAAAATAATAACAACAAACAATTGTGGTTTTTCTCTTGATACTGATACTCGGGTTTCACGTAXXXXXXXXXXCCTACAGAAAACGTCCAAAAATAACTTCCTGTCAGTTCTGTAGTCGTCCGCACCGCCGAAATGCCCCAAGCGCCGCCATG

>Marker31053

TATAAATTCTTGATCAAGACATGCTCTTCGGCATTCCTCTTCCAAGTTATAATAGTCTGTTAAAACTCTTATGCAGTCATXXXXXXXXXXAAATGAGACGATTTAAAAATCTGGTCAGAATGATCTCCAAGGGCTTGAAAACATTTTTTTAAAACTTATGGTTTCTTTTA

TATAAATTCTTGATCAAGACATGCTCTTCGGCATTCCTCTTCCAAGTTATAATAGTCTGTTAAAACTCTTATGCAGTCATXXXXXXXXXXAAATGAGAAGATTTAAAAATCTGGTTAGAATGACCTCTAAGGGCTTGAAAACATTTTTTTTAAACTTATGGTTTCTTTTA

>Marker31097

CAGACATTTCTCTTTTGCGTGGTGTGCTGGGACTAGGATTTTATGGTTTTAAGTCTGTGTTGTTTGGAGAAAACCTAGTTXXXXXXXXXXAATGTGTCCAGTCCAAAACATGATATAAACTTGAAAGTCCTTAAAAGTTGTCATGATCTGTAGCAGCTACTAATGGTTTG

CAGACATTTCTCTTTTGCGTGGTGTGCTGGGACTAGGATTTTATTGTTTTAAGTCTGTGTTGTTTGGAGAAAACCTAGTTXXXXXXXXXXAATGTGTCCAGTCCAAAACATGATATAAACTTGAAAGTCCTTAAAAGTTGTCATGATCTGTAGCAGCTACTAATGGTTTG

>Marker31358

ACAACCATGGACAATACGTCCCAATTCTGCCCATTCCATACTAACAAGCAACTTCAAAACAGTAGTGAAAACAAACGGATXXXXXXXXXXGAATGAGTTTTCCTAAATTTAAATCACCGCTTTGGATACAGTCTTGGAGCAGAGCAGCATAGGAATAAGAATCGAAGTCT

ACAACCATGGACAATACGTCCCAATTCTGCCCATTCCATACTAACAAGCAACTTTAAAACAGTAGTGAAAACAAACGGATXXXXXXXXXXGAATGAGTTTTCCTAAATTTAAATCACCGCTTTGGATACAGTCTTGGAGCAGAGCAGCATAGGAATAAGAATCGAACTCT

>Marker31594

CACCAGATTAGAATGTATAGTCGGGACATAGAGAAAACAGCATTTAGAACCCATGAAGGGCATTATGAATTTCTGGTGATXXXXXXXXXXTAAGGTAGAGTATCTGGGGCATATACTAACTGGAGAAGGAGTAGAGGTAGACCATGATAAGATCAGATCAATCAAGCAAT

CACCAGATTAGAATGTATAGTCAGGACATAGAGAAAACAGCATTTAGAACCCATGAAGGGCATTATGAATTTCTGGTGATXXXXXXXXXXTAAGGTAGAGTATCTGGGGCATATACTAACTGGAGAAGGAGTAGAGGTAGACCATGAGAAGATCAGATCAATCAAGCAAT

>Marker31673

CCAGTTTTCTCCCACTCTATCTATAGTTTCATGTTGCCTTCGTAGAGTCTTGGCGTTAGGGTTTTATAGTTTCCATTGCCXXXXXXXXXXCCAAGAAACTAGCACCAACAATTGGAATATCAGTTGACCATCGCCGTAGGAATCGATCTCTTGAAAGTCTACAGGCTAAT

CCAGTTTTCTCCCACTCTATCTTTAGTTTCATGTTGCCTTCTTAGAGTCTTGGCGTTAGGGTTTTATAGTTTCCATTGCCXXXXXXXXXXCCAAGAAACTAGCACCAACAATTGGAATATCAGTTGACCATCGCCGTAGGAATCGATCTCTTGAAAGTCTACAGGCTAAT

>Marker31832

ATCTAGAACCAATTATATATTGCACAAGAGAAATCAATGTCAAAAATCACAACACTAAACCAATAGAGCAACAAGACAAGXXXXXXXXXXTCACTAGATGCTCAGTTAGGTATTCAGATGTTAAGCCTCTCTTAAGCAATGAAAGCTATTTGTGGTTATTTGTAGTTGTA

ATCTAGAACCAATTATATATTGCACAAGAGAAATCAATGTCAAAAATCACAACACTAAACCAATAGATCAACAAGACAAGXXXXXXXXXXTCACTAGATGCTCAGTTAGGTATTCAGATGTTAAGCCTCTCTTAAGCAATGAAAGCTATTTGTGGTTATTTGTAGTTGTA

>Marker31858

TAGACTCAAAGTGAGAATTGATATTCAACTCTGCTAGAGTTGGAAAATTGAAAAACCCACACCCAAATATTTTGTTTGTGXXXXXXXXXXTTCATATATTTTCCTACTATTTGAGTAGAGATACATGATAGAATATTGGTAGAGGTGCTATTCTTAGAGTTTGGTAACAT

TAGACTCAAAGTGAGAATTGATATTCAACTCTGTTAGAGTTGGAAAATTGAAAAACCCACACCCAAATATTTTGTTTGTGXXXXXXXXXXTTCATATATTTTCCTACTATTTGAGTAGAGATACATGATAGAATATTGGTAGAGGTGCTATGCTTAGAGTTTGGTAACAT

>Marker32164

TCGAGAGCATATCCCTAGATACATTGGATATGATTAATGTGTCTAAAGCTAAAGATTAAGGTGAAGGAAAATCTATGTGGXXXXXXXXXXAGGAATGAGATTTTCTCAATTCGTCTCTTTCAAAGCCAGGCTTCTCAAGAAGCCCATGCATTGACTTCAATAGTAATTCT

TCGAGAGCATATCCCTAGATACACTGGATATGATTAATGTGTCTAAAGCTAAAGATTAAGGTGAAGGAAAATCTATGTGGXXXXXXXXXXAGGAATGAGATTTTCTCAATTCGTCTCTTTCAAAGCCAGGCTTCTCAAGAAGCCCATGCATTGACTTCAATAGTAATTCT

>Marker32481

ACGTGTGGTTCAATTGGAAGATTCTTTATTCTTAAGTGGAGCTGATGACGCAAAGGATGCGTCTGATAAGCTTGGATCATXXXXXXXXXXGTCTTATTGATAAAACAAGTTGATTATTCCACAAGAAATATGATTGTAGATGTAATATAGAGCAAAAAAAATTCATGAAA

ACGTGTGGTTCAATTGGAAGATTCTTTATTCTTAAGTGGAGCTGATGACGCAAAGGATGCGTCTGATAAGCTTGGATCATXXXXXXXXXXGTCTTATTGATAAAACAAGTTGATTATTCCACAAGAAATCTGATTGTAGATGTAATATAGAGCAAAAAAAATTCATGAAA

>Marker32526

TTTATTGTTTATCATAAAGTTAAAAGATTAAAAATATATTAAAGATTTGTCTTATAGGTTAAGTGAACTACATGTTTTAGXXXXXXXXXXTCATTATTTAGAAAAGGTATAGAAAATAGGGACTAAATTGAAAAGACATCCCTAAATGACATCCCTATCGAGGTTTAAAT

TTTATTGTTTATCATAAAGTTAAAACATTAAAAATATATTAAAGATTTGTCTTATAGGTTAAGTGAACTACATGTTTTAGXXXXXXXXXXTCATTATTTAGAAAAGGTATAGAAAATAGGGACTAAATTGAAAAGACATCCCTAAATGACATCCCTATCGAGGTTTAAAT

>Marker32892

ATGCTGAGCAAACAAACTCCAAATCTTCAGAACCATCTCCCTTCGATATGTCAACAAGCACGCTAATATGAAGACGAAGGXXXXXXXXXXCAAAATGTAGGATGAAGAAATGGATCGAAATGAGGGAAAAAAAATCGGCGAACCTGATGAGGAGGGGCGTAACTGGACTG

ATGCTGAGCAAACAAACTCCAAATCTTCAGAACCATCTCCCTTCGATATGTCAACAAGCACGCTAATATGAAGACGAAGGXXXXXXXXXXCAAAATGTAGGATGAAGAAATGGATCGAAATGAGGGGAAAAAAATCGGCGAACCTGATGAGGAGGGGCGTAACTGGACTG

>Marker32955

CTTTGTTCAAGTTTCAGAGACACCATTGAAAAGAACACTCATCTACTTACCCTAAAGTCGAGATGAAGTGAATTTCATCTXXXXXXXXXXGTCATCTATATGAACACGTCGAATCATTGCGTTTTTATCAAATACAAAATGAGTTGTATGCATAGTGTTACCAGGATAAG

CTTTGTTCAAGTTTCGGAGACACCATTGAAAAAAACACTCATCTACTTACCCTAAAGTCGAGATGAAGTGAATTTCATCTXXXXXXXXXXGTCATCTATATGAACACGTCGAATCATTGCGTTTTTATCAAATACAAAATGAGTTGTATGCATAGTGTTACCAGGATAAG

>Marker33054

TTGTTGAATCTCGGTGAGCTTCCAATCATCACACTCAACGGATCAAACATTCCGGCTATTCTGTATATGGACGGACTTTCXXXXXXXXXXGGTGGCAGCTCACGGACTCGTGTTATGGATCTCCATACTAAGGCAGATAAGGATTGGAAGGAGGAGATTTTGTTGGTTTT

TTGTTGAATCTCGGCGAGCTTCCAATCATCACACTCAACGGATCAAACATTCCGGCTATTCTGTATATGGACGGACTTTCXXXXXXXXXXGGTGGCAGCTCACGGACTCGTGTTATGGATCTCCATACTAAGGCAGATAAGGATTGGAAGGAGGAGATTTTGTTGGTTTT

>Marker33242

TTACGTGTTCGGATGAAAATTTTATAATCAAAACGGGGGCACAACGCACTGCTTCCAGTGAAGGTGTTGCTCTAATTGCAXXXXXXXXXXGACCCAGGAGGGTTATATGGTTTGAGTTCTTCTATTGTGAATGTAAAATAGCTTTAACTTCAATGAACTACTGGTTGGAA

TTACGTGTTCGGATGAAAATTTTATAATCAAAGCGGGGGCACAACGCACTGCTTCCAGTGAAGGTGTTGCTCTAATTGCAXXXXXXXXXXGACCGAGGAGGGTTATATGGTTTGAGTTCTTCTATTGTGAATGTAAAATAGCTTTAACTTCAATGAACTACTGGTTGGAA

>Marker33388

AAGGCAAGGCATCATAAAAAGAGAATCTGAAAGTAGGCGGAAAAAGACGAAGACATCGGACCTAAGTAAACAGAAGGACGXXXXXXXXXXCAAGGTTATAAATGTATCAATTATTCCATATCCCCAAAAAGCATGCTACCTCATTAGGAAAGATGCCCAATTGGAAATAA

AAGGCAAGGCATCATAAAAAGAGAATCTGAAAGTAGGCGGAAAAAGACGAAGACATCGGACCTAAGTAAACAGAAGGACGXXXXXXXXXXCAAGGTTATAAATGTATCAATAATTCCATATCCCCAAAAAGCATGCTACCTCATTAGGAAAGATGCCCAATTGGAAATAA

>Marker33472

TACTCTCAATTTTATATTTCATTTTCCTATATTTTGAATTAAAAATGTGTGGGTATTTTCAAACCCTAAATTAAGTTTTAXXXXXXXXXXGAAATATTGGTTACAACTTGTGGAAATATGGTAAAAAAACAAATTGAAACAAAATGAAGTTAAAGGTTCAAAATGGAGTT

TACTCTCAATTTTATATTTCATTTTCCTATATTTTGAATTAAAAATGTGTGGGTATTTTCAAACCCTAAATTAAGTTTTAXXXXXXXXXXCAAATATTGGTTACAACTTGTGGAAATATGGTAAAAAAACAAATTGAAACAAAATGAAGTTAAAGGTTCAAAATGGAGTT

>Marker33589

CATTGATGTGAAATTTCAATGGCACAACAAGCGTTTGACAGTTGGGCAAAACATCCTCTAATAAAAAGACAAGCGAAGCAXXXXXXXXXXAAGAATCAGATAAGAATCTGTTGTCCCCAAAATCCATCTACCGTCATAGGTAACATCTACATGTGTAATAGGTGAACCAA

CATTGATGTGAAATTTCAATGGCACAACAAACGTTTGACAGTTGGGCAAAACATCCTCTAATAAAAAGACAAGCGAAGCAXXXXXXXXXXAAGAATCAGATAAGAATCTGTTGTCCCCAAAATCCATCTACCGTCATAGGTAACATCTACATGTGTAATAGGTGAACCAA

>Marker33647

CTATAATTTGAGTCATCCCAGAACAAATCCGACATGTTTGGGAACCATAGAATACAAAAATACAATAAAATGTTTCATGAXXXXXXXXXXCACATAAATTCCAAACGAGTATTAGGAAACTTGGTCAATATTTAACAATCAAAAAACATGCATATTTATAACATGTGAAG

CTATAATTTGAGTCATCCCAGAACAAATCCGACATGTTTGGGAACCATAGAATACAAAAATACAATAAAATGTTTCATGAXXXXXXXXXXCACATAAATTTCAAACGAGTATTAGGAAACTTGGTCAATATTTAACAATCAAAAAACATGCATATTTATAACATGTGAAG

>Marker33840

CTCATACAGAGCTTACCGTCTATATTTGCACATAGGAATATGTGTTTCCCTACAGGTAATCCCAGGATTTGATCGTCTAAXXXXXXXXXXCGGTGGTGATCAACTCGCCGATTCTGTATTCCTCTAGCATTTTTTTGGCTTTGTCCGAGTGAATGGCGTCGAATTCCTCC

CTCATACAGAGCTTACCGTCTATGTTTGCACATAGGAATATGTGTTTCCCTACAGGTAATCCCAGGATTTGATCGTCTAAXXXXXXXXXXCGGTGGTGATCAACTCGCCGATTCTGTATTCCTCTAGCATTTTTTTGGCTTTGTCCGAGTGAATGGCGTCGAATTCCTCC

>Marker33909

TAGTGAAATAAGTGTTTCTCTTTCTGTGATAGAGTTTGAATGCTTGACGCCCCTAATGGTCAAACTTTCTTAATGAAGACXXXXXXXXXXACTTCTTTCAATAGGATACATCCAACTATAAGAAATTGAATCGGCAATCTTGGTTTCGTATGATAGGTGAATGGCAAGGT

TAGTGAAATAAGTGTTTCTCTTCCTGTGATAGAGTTTGAATGCTTGACGCCCCTAATGGTCAAACTTTCTTAATGAAGACXXXXXXXXXXACTTCTTTCAATAGGATACATCCAACTATAAGAAATCGAATCGGCAATCTTGGTTTCGTATGATAGGTGAATGGCAAGGT

>Marker34032

ATAAAAGAATCTCTTGTAAATTTACGAAACAGTTTTGAGAGTGACAGTTTTTAGTCAAAGATTAAATCATTTTTCTTGTTXXXXXXXXXXTACACGGTCGTGTAGCTTTTTTTAACGATTGGAAAAATGCTTCATATAATTCTTCTTTTAAACGATTATGATACACGTGT

ATAAAAGAATCTCTTGTAAATTTACGAAACAGTTTTGAGAGTGACAGTTTTTAGTCAAAGATTAAATCATTTTTCTTGTTXXXXXXXXXXTACACGGTCGTGTAGCTTTTTTTAACGATTGAAAAAATGCTTCATATAATTCTTCTTTTAAACGATTATGATACACGTGT

>Marker34113

TTTGCTTGTGTTATTCCCTTCTTGCCCTTCATTTTATATGTCTGTAATGTTGGGGGTCTAACATATTACTACTATCAGTTXXXXXXXXXXGCTCATTCCCTATGTTGATCAATGAAACATTGTTCAATGTTTACTTTTCTTCTTGAACTTTATATTGGATGCATACAAAG

TTTGCTTGTGTTATTCCCTTCTTGCCCTTCATTTTCTATGTCTGTAATGTTGGGGGTCTAACATATTACTACTATCTGTTXXXXXXXXXXGCTCATTCCCTATGTTGATCAATGAAACATTGTTCAATGTTTACTTTTCTTCTTGAACTTTATATTGGATGCATACAAAG

>Marker34226

AAACATGGAGTGGACTATACTCCACTCTAATCCTTAAGCCAAATAGCCCCAAAGAGTTTTCATATTTGGAGATTATCCTTXXXXXXXXXXTCCAGAAGGAACAAAACATATTTAATTTTCAACAAAAAAAATTTTTTCAATATATATATTTGTTAATTGAGAGTGGGGAT

AAACATGGAGTGGACTATACTCCACTCTAATCCTTAAGCCAAATAGCCCCAAAGAGTTTTCATATTTGGAGATTATCCTTXXXXXXXXXXTCCAGGAGGAACAAAACATATTTAATTTTCAACAAAAAAGTTTTTTTCAATATATATATTTGTTAATTGAGAGTGGGGAT

>Marker34296

ACTCCACTAAAAACTCACAACTGAACTCTTCTCATTGTAGATATATTTATGTGTTTACATATATAGACCAATAACAGTAAXXXXXXXXXXATTTCATTTTGTGTAATTATGTTTTCAGCTTCCCACTCGGTCTTATCCTCAAAATTATAAGCATATTGAGTTGGTGATCT

ACTCCACTAAAGACTCACAACTGAACTCTTCTCACTGTAGATATATTTATGTGTTTATATATATAGACCAATAACAGTAAXXXXXXXXXXATTTCATTTTGTGTAATTATGTTTCCAGCTTCCCACTCAGTCTTATCCTCAAAATTATAAGCATATTGAGTTGGTGATCT

>Marker34424

CCCATGAATTGAACTCATGACCTCTTAGTTAGTTATTGACACTAACTAAGAATCAACTACTCTCAGGTTAGTATTATCAAXXXXXXXXXXAAACGCACCCAAACTTGGCTGGTCCTCTCTATGCTTTCTTACCCTTAATCTTTTTCTTCCCATAAACTTTCATAATCGGA

CCCATGAATTGAACTCATGACCTCTTAGTTAGTTATTGACACTAACTAAGAATCAACTACTCTCAGGTTAGTATTATCAAXXXXXXXXXXAAACGCACCCAAACTTGGCTGGTCCTCTCTATGCTTTCTTACCCTTATTCTTTTTCTTCCCATAAACTTTCATAATCGGA

>Marker34540

ACATGATTCTACATTTGAATGTTTGACAACTGATAATACTCTGTCTGCACCAAATTCATAAACACCAGAAATGTCCAAACXXXXXXXXXXCCAAAATCTGATCTCTGTTTGATGTTGAAACCGGAGAATTTGAATTTTTTTGTTAAATTATAAAGTTGAATATGCAATGA

ACATGATTCTACATTTGAATGTTTGACAACTGATAATACTCTGTCTGCACCAAATTCATAAACACCAGAAATGTCCAAACXXXXXXXXXXCCAAAATCTGATCTCTGTTTGATGTTGAAACCGGAGAATTTGAATTTTTTTGTTACATTATAAAGTTGAATATGCAATGA

>Marker34551

GAGTCGAGCCCATATTCAAGTCTGTTACTCTTAGTGAAGAAAAAAGATGTGGGATGGGAATTTTGTGTAGATTACCGCAAXXXXXXXXXXAGGAGACCATGAGTTCCTTGTGTCATTTAGGTTAACCAACACCCCCACTACTTTCCAATCCCTAATGAATCAAGTATTTT

GAGTCGAGTCCATATTCAAGTCTGTTACTCTTAGTGAAGAAAAAAGATGTGGGATGGGAATTTTGTGTAGATTACCGCAAXXXXXXXXXXAGGAGACCATGAGTTCCTTGTGTCATTTAGGTTAACCAACACCCCCACTACTTTCCAATCCCTAATGAATCAAGTATTTT

>Marker34827

ATAAAGATTGACCCTGCTGCTATTTTGGATACATGGGCAGCCCAATACACAGATAAAGGGCTGGGAATGAGATGAGCCTAXXXXXXXXXXAGCCCAGCAATGTCCTCATCAAGTAACCCGTTTGCTGCAGATCTACAGCCACTCATCGATCAAGCCAAGCTTCATGGATG

ATAAAGATTGACCCTGCTGCTATTTTGGATACATGGGCAGCCCAATACACAGATAAAGGGCTGGGAATGAGATGAGCCCAXXXXXXXXXXAGCCCAGCAATGTCCTCATCAAGTAACCCGTTTGCTGCAGATCTACAGCCACTCATCGATCAAGCCAAGCTTCATGGATG

>Marker34863

AAATTTCAAATAGATTATTTTGTATAATTCAGTCCATCATTTTTCATTCTGGTTAAACATTTCTACAAATTTCCAAATCGXXXXXXXXXXTTCATCTTCTGTTATTCCCAACCGCTCCTTCTCTTTCTCTCTAAGAAGAAGATATGATGTTACTCGGGAAGCGTCCACGA

AAATTTCAAATAGATTATTTTGTATAATTCAGTCCATCATTTTTCATTCTGGTTAAACATTTCTACAAATTTCCAAATCGXXXXXXXXXXTTCATCTTCTGTTATTCCCAACCGCTCCTTCTCTTTCTCTCTAAGAAGACGATATGATGTTACTCGGGAAGCGTCCACGA

>Marker34913

ATTCTCATTTTAATTGTCCACGGAAACGCCAATTTCTTACATTAGCCTGCCCGTATTTCTCTTCCCTGGAGGCAGAAGCAXXXXXXXXXXTTTCCAGAAATCTAACAAGCGAGAAGAAGAAGACAAAATTTTAAAAACCCCATTCATACCCATTTGCAGAAATCTCATAA

ATTCTCATTTTAATTGTCCACGGAAACGCCAATTTCTTACATTAGCCTGCCCGTATTTCTCTTCCCTGGAGGCAGAAGCAXXXXXXXXXXTTTCCAGAAATCTAACAAGCGAGAAGAAGAAGACAAAATTTAAAAAACCCCATTCATACCCATTTGCAGAAATCTCATAA

>Marker34947

AAGGCACGGGGCAAGGGATGCCTCTCCCCTATCCCATTCTCCATCCCTACAAATTTTCCTGTTGGAACAAGGTGGGTATTXXXXXXXXXXTGTATTACTCGCATAAGCAGAGGATAAAATGGGGAACGAGGTTACCACATTCTTAGAATATATCGGTAAAGGTGCATGAA

AAGGCACGGGGCAAGGGATGCCTCTCCCCTATCCCATTCTCCATCCCCACAAATTTTCCTGTTGGAACAAGGTGGGGATTXXXXXXXXXXTGTATTACTCGCATAAGCAGAGGATAAAATGGGGAACGAGGTTACCACATTCTTAGAATACATCGGTAAAGGTGCATGAA

>Marker35030

TTCAAGAAGCAAGCCGAAACCTCTATCAACCCTAACAACTTTTGCAGAATCATAGATATCACCAATTCTCACGTGCTACAXXXXXXXXXXTAAAGAATCTTTCGAGACATTGATACCATATGTTTGAATCATGTTAATGATTTTAACAATACTACAGTTACATTATTTAT

TTCAAGAAGCAAGCCGAAACCTCTATCAACCCTAACAACTTTTGCAGAATCATAGATATCACCAATTCTCAAGTGCTACAXXXXXXXXXXTAAAGAATCTTTCGAGACATTGATACCATATGTTTGAATCATGTTAATGATTTTAACAATACTACAGTTACATTATTTAT

>Marker35059

CAACCAAGTTGTTGCAAACGTTCTTTTCAATATGCATTATGTCTAGTTTGTGACGTAATAATAATCTCAACCAGTAAGGAXXXXXXXXXXGTCTCATGAAAGATATTTTCTCTCTTATCCCGAAGAATGATCTATCTCCCATGCATATGAGACATGTTTGATACCTTTTT

CAACCAAGTTGTTGCAAACGTTCTTTTCAATATGCATTATGTCTAGTTTGTGACGTAATAATAATCTCAACCAGTAAGGAXXXXXXXXXXGTCTCATGAAAGATATTTTATCTCTTATCCCGAAGAATGATCTATCTCCCATGCATATGAGACATGTCTGATACCTTTTT

>Marker35092

TTGAAGGATTGTTGCTCTCTGATTGTGAAACTGAAAGCTGGAACACATGGAAATCAATTAATAACAGCCTAGATCATACAXXXXXXXXXXATGACCTGACTAAGGTGCTGCTTCTCGGTGCTGCTACCGTAGATTGAATTTCACTTTCAGAGGATGAAGGAAAAAGAGGA

TTGAAGGATTGTTGCTCTCTGATTGTGAAACTGAAAGCTGGAACACATGGAAATCAATTAAAAACAGCCTAGATCATACAXXXXXXXXXXATGACCTGACTAAGGTGCTGCTTCTCGGTGCTGCTACCGTAGATTGAATTTCACTTTCAGAGGATGAAGGAAAAAGAGGA

>Marker35292

CTAAATAAGAATATTAGTATGTTAGTATTTTGGGGTATAAATTATAACGGTCACGAGAGGGAAAGTTAGTTACTGCTGTAXXXXXXXXXXGATCTTTCTTGAAGAAACAATAATCACAATTGCAAGACAATATTCTCAAATGTATTAATTTCCTAGAAACGAAAAGAAAA

CTAAATAAGAATATTAGTATGTTAGTATTTTGGGGTATAAATTATAACGGTCACGAGAGGGAAAGTTAGTTACTGTTGTAXXXXXXXXXXGATCTTTCTTGAAGAAACAATAATCACAATTGCAAGACAATATTCTCAAATGTATTAATTTCCTAGAAACGAAAAGAAAA

>Marker35408

TCAATCCAAAAGAACAAGACCTAATTGATAAATACAATATACGTTAACCACCAGGACCCAAACCAAGGCATTAAACCCAAXXXXXXXXXXCCAAACATTCTCCAAATGATCCAACTCGCGTGACCCTTCATACACTGGTTATCAAGAAAGAAACAAAAGATCCAACTCTT

TCAATCCAAAAGAACAAAACCTAATTGATAAATACAATATACGTTAACCACCAGGACCCAAACCAAGGCATTAAACCCAAXXXXXXXXXXCCAAACATTCTCCAAATGATCCAACTCGCGTGACCCTTGATACACTGGTTATCAAGAAAGAAACAAAAGATCCAACTCTT

>Marker35566

ACTTTTATCTGTAACTTTAGTAGTTTAGTTTATAATTTTTAAAAGTTTAAAAAGAGGGTAAATAGTAAAGCAAGAATAGGXXXXXXXXXXTGGGCATGATTATGAAGAGGACTTAACTACAGAAGGCAAGTCAAATTTCTTTTCTGTTTCAGAAAAATGGGCTTATAGCA

ACTGTTATCTGTAACTTTAGTAGTTAAGTTTATAATTTTTAAAAGTTTAAAAAGAGGGTAAATAGTAAAGCAAGAATAGAXXXXXXXXXXTGGGCATGATTATGAAGAGGACTTAACTACAGAAGGCAAGTCAAATTTCTTTTCTGTTTCAGAAAAATGGGCTTATAGCA

>Marker35748

TAAGTTTTTATTGGTTATAGGGTTGGCTTACATTGGAAAAAAAGTTCCAAAGAATTCAAAGTTTTGGGGAATTTAGGGAAXXXXXXXXXXATATCCTTTTCATCATGATTGGAACCTCACCTTTCTTATTAAATGAACACAATTCAAAGGTGCATGAATGTCACAATTCA

TAAGTTTTTATTGGTTATAGGGTTGGCTTACATTGGAAAAAAAGTTCCAAAGAATTCAAAGTTTTGGGGAATTTAGGGAAXXXXXXXXXXATATCCTTTTCATCATGATTGGAACCTCACATTTCTTATTAAATGAACACAATTCAAAGGTGCATGAATGTCACAATTCA

>Marker35786

TACTTACTTTTAAATTTGAGTGTTTTACATGTTTACTAATTTTTGTTTTAGTTCGTCTTCGAATTACATCAACTCAAACGXXXXXXXXXXTAGACTATATCTAAATTTAGTAAGAAAATTCATTCGGTCTCTTTTGAAAATGACCAATTGATAATATCCATTCATTAAAT

TACTTACTTTTAAATTTGAGTGTTTTACATGTTTACTAGTTTTTGTTTTAGTTCGTCTTCGAATTACATCAACTCAAACGXXXXXXXXXXTAGACTATATCTAAATTTAGTAAGAAAATTCATTCGGTCTCTTTTGAAAATGACCAATTGATAATATCCATTCATTAAAT

>Marker35890

TGTAGTTGGGACAATTCCAAACAATTTTTCTGCATAGGTCAAATTCCTGCCAGATAAGGAAGAGCCTATTAAAAAAGGATXXXXXXXXXXTATCTCACCTATCGAAAACCCAAACTTGTGGAGCGCTTCAAGAATCATTTTGATAGATTGGGACCACGAAAAATAGTTAT

TGTAGTTGGGACAATTCCAAACAATTTTTCTGCATAGGTCAAATTCCTGCCAGATAAGGAAGAGCCTATTAAAAAAGGATXXXXXXXXXXTATCTCACCTATCGAAAACCCAAACTTGTGGAGCCCTTCAAGAATCATTTTGATAGATTGGGACCACGAAAAATAGTTAT

>Marker35915

TTCAATTAAAAATTCATGTGGTTAGCTTGATGAATGCGATGTTTAAGTGTTACGCCAAGTGTATTAATTAAAAGATTATAXXXXXXXXXXTCAATTATGACACATAGTTTTTTATCAATATTCTCAACATGCATGCAACTAGTAAAATAAAATAAAATTTACACTTTTAT

TTCAATTAAAAATTCATGTGGTTAGCTTGATGAATGTGATGTTTAAGTGTTACGCCAAGTGTATTAATTAAAAGATTATAXXXXXXXXXXTCAATTATGACACATAGTTTTTTATCAATATTCTCAACATGCATGCAACTAGTAAAATAAAATAAAATTTACACTTTTAT

>Marker36081

AATTATTATCAAAAGAAAATTATGGTTTTAAAACAACACAAAAAAAGGAATAATAAGAGGAGACATATGTAATGATTGTTXXXXXXXXXXTTTTATTTTTATCCAAAATCAACCATATAAACTACTTTCATTTTCTTTTACTTTAATAATATTAATATAAAACTTAATGG

AACTATTATCAAAAGAAAATTATGGTTTTAAAACAACACAAAAAAAGGAATAATAAGAGGAGACATATGTAATGATTGTTXXXXXXXXXXTTTTATTTTTATCCAAAATCAACCATATAAACTACTTTCATTTTCTTTTACTTTAATAATATTAATATAAAACTTAATGG

>Marker36341

TCTATGACTGTGAGTTCACTTTGTTCTAAATGTTGAAATTCATCTCGCCTTTTTTTTTTGTAGGTATTTAAGAAATACTTXXXXXXXXXXGTTTATGTGGCAATGCATGGTCATTGCATGCAAGATGCTCACTATGTCTCATCCTCACGCAATTACTAAGTCCTCATTGA

TCTATGACTGTGAGTTCACTTTGTTCTAAATTTTGAAATTCATCTCGCCTTTCTTTTTTGTAGGTCTTTAAGAAATACTTXXXXXXXXXXGTTTATGTGGCAATGCATGGTCATTGCATGCAAGATGCTCACTATGTCTCATCCTCACGCAATTACTAAGTCCTCATTGA

>Marker36383

CTTTGAAATGATGTAATGCTACTTTATTATTCCTTTACCTATGATTGATTGATTTTGAAAATTTTCTAAGCAAAGAAACTXXXXXXXXXXGGGACCTCTCGTGAGTCATAGAGACTTGCAGTTTGGATTATATTTAGGTGACTTTGGTATGCCTTTTACCTTTAGAGTTT

CTTTGAAATGATGTAATGCTACTTTATTATTCCTTTACCTATGATTGATTGATTTTGAAAATTTTCTAAGCAAAGAAACTXXXXXXXXXXGGGACCTCTGGTGAGTCATAGAGACTTGCAGTTTGGATTATATTTAGGTGACTTTGGTATGCCTTTTACCTTTGGAGTTT

>Marker36518

TTTCCCTGAGGCAGCGAACAGCTGTTTTCAATAGTTCGCTGACTTCGGGATTATCAGTTTCTCCTAGGCTCCTGCACTCTXXXXXXXXXXGGCGGTATTCAAAACTTAAATCTTGTATAGCAGACAATCCCAAAACAATTGAATTACGATTATAGGTTGAGTGAACACAT

TTTCCCTGAGGCAGCGAACAGCTGTTTTCAATAGTTCGCTGACTTCGGGATTATCAGTTTCTCCTAGGTTCCTGCACTCTXXXXXXXXXXGGCGGTATTCAAAACTTAAATCTTGTATAGCAGACAATCCCAAAACAATTGAATTACGATTATAGGTTGAGTGAACACAT

>Marker36609

ATTGAAAGTGGGCAAGCAACTACTTATGGTAATATGTTAAATTCAATGAGATCCACCATTCGTAATACTGACCTTAACCCXXXXXXXXXXATAATCTCAATGTGAACTAGAATTTAAAATAACTTTATTATTCATTTCGTTATCTTTGGTAAACTAGTATGAGGGATTGG

ATTGAAAGTGGGCAAGCAACTACTTATGGTAATATGTTAAATTCAATGAGATCCACCATTCGTAATACCGACCTTAACCCXXXXXXXXXXATAATCTCAATGTGAACTAGAATTTAAAATAACTTTATTATTCATTTCGTTATCTTTGGTAAACTAGTATGAGGGATTGG

>Marker36955

ATAGTTTGAATTTGATACCAAAAAGGAATGTGTAGAAAAAGGAACAAAAACCTGCCGGATTTGAAATACAAGGAGAGATTXXXXXXXXXXCCTAATTAAGGGAGTAAGGGAGGGGTAAGGCTTCCATTCAAAGCAAAAAAAGGTCTCAGTGTCAAAGAGAAACCTCACAT

ATAGTTTGAATTTGATACCAAAAAGGAATGTGTAGAAAAAGGAACAAAAACCTGCCGGATTTGAAATACAATGAGAGATTXXXXXXXXXXCCTAATTAAGGGAGTAAGGGAGGGGTAAGGCTTCCATTCAAAGCAAAAAAAGGTCTCAGTGTCAAAGAGAAACCTCACAT

>Marker37007

ACAAATATCAATATCAAGGAGACCCACCTGGAAGTCCATGGCAGCTAGGGCAAATGCGAGCTGAGCAGAGAATGTGCTCTXXXXXXXXXXCAAAATGTAGAGTAACCCCTACACTAAATAAAAGAAAGAAAGAAAACTAAGGTATATTCAAAACAAGGCAACATCAGAAA

ACAAATATCAATATCAAGGAGACCCACCTGGAAGTCCATGGCAGCTAGGGCAAATGCGAGCTGAGCAGAGAATGTGCTCTXXXXXXXXXXCAAAATGTAGAGTAACCCCTACACTAAATAAAAGAAAGAAAGAAAACTAAGGTGTATTCAAAACAAGGCAACATCAGAAA

>Marker37108

ACCATGCTTAGCCCCTCGATGTGTTGTCTTTGCTTGAAAGAAGAGGAAACTTTGGACCATTTATTTTTACACTATCTCTTXXXXXXXXXXCACACAGCTTCTTGGTGGAGCACGAATTACACCAAACACTTTTGTAATTATAGCCTTTCAATGATTTTCAACAATTGGAA

ACCATGCTTAGCCCCTCGATGTGTTGTCTTTGCTTCAAAGAAGAGGAAACTTTGGACCATTTATTTTTACACTATCTCTTXXXXXXXXXXCACACAGCTTCTTGGTGGAGCACGAATTACACCAAACACTTTTGTAATTATAGCCTTTCAATGATTTTCAACAATTGGAA

>Marker37151

ATAATTCAAATATCCCAACCTAAAAAACCATTGATTTAGCCCCCCCAGCTACGATAGATTAAAAAAACAAAAAAAAAAACXXXXXXXXXXGCTAACAACCTTGACGATTAGTGTGTGACCATTGGTGCCGGGTTTGAGCTGTTCGATCTTAACAAAGACCGGCTTCCTCA

ATAATTCAAATATCCCAACCTAAAAAACCATTGATTTAGCCCCCCCAGCTACGATAGATTAAAAAAACAAAAAAAAAAACXXXXXXXXXXGCTAACAACCTTGACGATGAGTGTGTGACCATTGGTGCCGGGTTTGAGCTGTTCGATCTTAACAAAGACCGGCTTCCTCA

>Marker37378

AAGATGTAATGCACATCTTTTATACATTGTCCCGTCTCATTTCACCATACACATCTTTTATACATGACAATCTTTACCCCXXXXXXXXXXTCATGTAAATTATTTTGAAGAAACACTCACAACAATTGGTAACTTAAACCCAAAATGCTCATCTTCTTGCATAGATACTT

AAGATGTAATGCACATCTTTTATACATTGTCTCGTCTCATTTCACCATACACATCTTTTATACATGACAATCTTTACCCCXXXXXXXXXXTCATGTAAATTATTTTGAAGAAACACTCACAACAATTGGTAACTTAAACCCAAAATGTTCATCTTCTTGCATAGATACTT

>Marker37662

TCTGTCTAACGTTTACAGAGTGGTCATGCTGTCTGTATAAGATGGCAAGCAAAGAACACCTTTTTTTTAACAGAAATAAAXXXXXXXXXXAGATCATAATTAGTAGTTGTATTGTATGAGCACAACGGGAACCTCCCACCTATCTTCATAATGGTATGATAGTCTACTTT

TCTGTCTAACGTTTACAGAGTGGTCATGCTGTCTGTATAAGATGGCAAGCAAAGAACACCTTTTTCTTAACAGAAATAAAXXXXXXXXXXAGATCATAATTAGTAGTTGTATTGTATGAGCACAACGGGAACCTCCCACCTATCTTCATAATGGTATGATAGTCTACTTT

>Marker37706

CTGTAGACATATAACCCAAAATTAATTAAACAAATACGAGTAGATGAAACTGAATATGTAAAATAATGGAATACGTGGGTXXXXXXXXXXTTGTTCATTCTCAACTTCTTCGTCTTCCAGCACGTTCCATATACGTTTATTTTGGACCACTTAAAATAACCCTGTAATTG

CTGTAGACATATAACCCAAAATTAATTAAACAAATACGAGTAGATGAAACTGAATGTGTAAAATAATGGAATACGTGGGTXXXXXXXXXXTTGTTCATTCTCAACTTCATCGACTTCCAGCACGTTCCATATACGTTTATTTTGGACCACTTAAAATAACCCTATAATTG

>Marker37738

AATTTATTGGTTTTATTTCATTTTTGATTTAGGAATGACTGCACCACAAGCTGCTGGAGTCATTCACTCAGACTTTGAGAXXXXXXXXXXTTTAGGCTTTCTGATTGTATTTTCTGGCTAATAAATCGAGAGTTTGAAGAGTTAGATTTTTTGCAATCGAACTTAAACAT

AATTTATTGGTTTTATTTCATTTTTGATTTAGGAATGACTGCACCACAAGCTGCTGGAGTCATTCACTCAGACTTTGAGAXXXXXXXXXXTTTAGGCTTTCTGATTGTATTTTCTGGCTAATAAATTGAGAGTTTGAAGAGTTAGATTTTTTGCAATCGAACTTAAACAT

>Marker37749

TCTTCGTGAATATGTATATATACTTATAGTAGGTATCAATTGCTAACATTGATAACTAGTATTAATGATAGCCTTCAACTXXXXXXXXXXGCATAAGTTGGACATGAGTTTTTTGGATGGATTGATGGGTAATTTAAGAATTGAATGTAAAGGAATTGGGGAGTATAAAG

CCTCCGTGAATATGTATATATACTTATAGTAGGTATCAATTGCTAACATTGATAACTAGTATTAATGATAGCCTTCAACTXXXXXXXXXXGCATAAGTTGGACATGAGCTTTTTGGATGGATTGATGGGTAATTTAAGAATTGAATGTAAAGGAATTGGGGAGTATAAAG

>Marker37759

AACTTCACTCCACCATGTAGACCTTTTCTAAATGGTTGCCTTTAACCTTATGTGCACATAACATATAGTTCATTCAAAGGXXXXXXXXXXAAAGTTTAAAATTGAATTTAATATATTTACTTTCTACGACGCACAGAAACCAGACATCGCAGAAATTTGCAAATTTAAGT

AACTTCACTCCACCATGTAGACATTTTCTAAATGGTTGCCTTTAACCTTATGTGCACATAACATATAGTTCATTCAAAGGXXXXXXXXXXAAAGTTTAAAATTGAATTTAATATATTTACTTTCTGCGACGCACAGAAACCAGACATCGCAGAAATTTGCAAATTTAAGT

>Marker37815

TGAATTTGCGAGTATTTAGAGAAGTGTGGCGTATCTATCGTTTTTTCTTTTGAAAATTTTAAAAATAGCAAAACATTCAAXXXXXXXXXXGTGTAGGTTAAATATAAAAACACCATTTTAACTGTTTGTGGAGCCGTGAAGTGAAAGTTCAAACGCATTCATCAACATAA

TGAATTTGCGAGTATTTAGAGACGTGTGGCGTATCTATCGTTTTTTCTTTTGAAAATTTTAAAAATAGCAAAACATTCAAXXXXXXXXXXGTGTAGGTTAAATACAAAAACACCATTTTAACTGTTTGTGGAGCCGTGAAGTGAAAGTTCAAACGCATTCATCAACATAA

>Marker37977

CATTATGTTTGTTGATTTGAACTTTATGATCTGTATGCTGACCATATATTTTTACTAAGTTGCTCCAAACTGTTTTGCCAXXXXXXXXXXATTACGTGCATATTTAGACCTAAATTTCTTTTTGTTCTTTCCATGAAGGTGCAAGATGCCTGCAGCAATTCGGAACCTTT

CATTATGTTTGTTGATTTGAACTTTATGATCTGTATGCTGACCATATATTTTTACTAAGTTTCTCCAAACTGTTTTGCCAXXXXXXXXXXATTACGTGCATATTTAGACCTAAATTTCTTTTTATTCTTTCCATGAAGGTGCAAGATACCTGCAGCAATTCGGAACCTTT

>Marker38143

CTTTGTTCAAGTCCCAAAAACACAATTTAAGGGAACACTCATCTACTTACCTTAAAGTCGATTATGAGTGAATTCCATCTXXXXXXXXXXGTCACCTACATGAACGCTTTGGATCATTGCGTTAGTATCAAATACAAAGTGAGTTGTATACATAGTGTTATCAAGATAAG

CTTTGTTCAAGTCCCAAAAACACAATTTAAGGGAACACTCATCTACTTACCTTAAAGTCGATTATGAGTGAATTCCATCTXXXXXXXXXXGTCACCTACATGAACGCTTTGGATCATTGTGTTAGTATCAAATACAAAGTGAGTCGTATACATAGTGTTATCAAGATAAG

>Marker38636

ACTCTTTCCTTGACGCAACTCTTTGCATTCGGAAGGAACTCAATGATAATCATCTTACTGGAATTATTCCAGCTGAGCTCXXXXXXXXXXATGGATGTCTTGTGTCTTCTTTCAAGTCCTCATTCATTTACTTACGCTTTGTAGAAATGTGGCTAATAACAATCTCGGAG

ACTCTTTCCTTGATGCAACTCTTTGCATTCGGAAGGAACTGAATGATAATCATCTTACTGGAATTATTCCAGCTGAGCTCXXXXXXXXXXATGGATGTCTTGTGTCTTCTTTCAAGTCCTCATTCATTTACTTACGCTTTGTAGAAATGTGGCTAATAACAATCTCGGAG

>Marker38857

AGCAAACATACCATCAGTTTCACCATGAAGCCGTTGCTCGTATCTTTACTCCACAACTCCCATTTTTCACGTTTCTTGGTXXXXXXXXXXGTCCTCTGCACAAGCTGTTCTTCTTCTCGAGCCAGGACTCGTTTGACATTGACACGGGTATTAGGTTCGTTCAATATGCA

AGCAAACATACCATCAGTTTCACCATGAAGCCGTTGCTCGTATCTTTACTCCACAACTCCCACTTTTCACGTTTCTTGGTXXXXXXXXXXGTCCTCTGCACAAGCTGTTCTTCTTCTCGAGCCAGGACTCGTTTGACATTGACACGGGTATTAGGTTCGTTCAATATGCA

>Marker38952

CAATATTCTGCCAACACAGCCACAAAATCCACTCTTTCGTTCTCTCCCCACGAAAACAGTGCTAATAAGCTTCTACACTTXXXXXXXXXXCATATTTGCACAGCTGATCCACTACAACAAATATTTTTTTTTGGAAAAGGAGACATGCTTCTTTATTAATAATATTCAAA

CAATATTCTGCCAACACAGCCAGAAAATCCACTCTTTCATTCTCTCCCCACGAAAACAGTGCTAATAAGCTTCTACACTTXXXXXXXXXXCATATTTGCACAGCTGATCCACTACAACAAATATTTTTTTTTGGAAAAGGAGACATGCTTCTTTATTAATAATATTCAAA

>Marker39131

TCAACGCCTTGAACTCGGGGAAGGAAAAATTAAAACGTAAGTTAAAGACTTAGTGAATGGAATTTTAGAAATACCTTTTGXXXXXXXXXXGCATCTTACATTTAGACTACTGTGATGTTCACTCCGTCAACAACATTTGAGAACTTCCCTTATTAATTTCTCGTTGCACA

TCAACGCCTTGAACTCGGGGAAGGAAAAATTAAAACGTAAGTTAAAGACTTAGTGAATGGAAGTTTAGAAATACCTTTTGXXXXXXXXXXGCATCTTACATTTAGACTACTGTGATGTTCACTCCGTCAACAACATTTGAGAACTTCCCTTATTAATTTCTCGTTGCACG

>Marker39270

TAAGCCTAATTAAACAGCTTTTTGTGATCTCGTTTATGATACAATAAGAAGAAAATTGGCGGTTTGTAGAAGTTTCCACAXXXXXXXXXXTCAAACAGATACATTCAAGAAGGAAATAAAGATCTAATGGCTCAGTATAATTCAGGCTTCATCTATACGGTGAAACTCTT

TAAGCCTAATTAAACAGCTTTTTGTGATCTCGTTTATGATACAATAAGAAGAAAATTGGCGGTTTTTAGAAGTTTCCCATXXXXXXXXXXTCAAACAGATACATTCAAGAAGGAAATAAAGATCTAATGGCTCAGTATAATTCAGGCTTCATCTATACGGTGAAACTCTT

>Marker39356

CTCCTTAATTTTCTCAACGTTCTCAAAATGATTGCAAAAGTCCATAATAGTCACTTATGATAGGGGTATGTTTTTTTAGGXXXXXXXXXXATTCAATGTAAAGAACCTTTTAAATTATTATCAAACTCTTTCTTGCAACTTCATTCTATGAATTTTTTTTTTCCGTTTAA

CTCCTTAATTTTCTCAACGTTCTCAAAATGATTGCAAAAGTCCATAATAGTCACTTACGATAGGGGTATGTTTTTTTAGGXXXXXXXXXXATTCAATGTAAAGAACCTTTTAAATTATTATCAAACTCTTTCTTGCAACTTCATTCTATGAATTTTTTTTTTCCGTTTAA

>Marker39385

TTCTTTACCCCTTAAATTTCCAACATCATCAGATCTTTTAATTCATGTTTTAATTTTGAAAAAATGAATAAAAGAAAAACXXXXXXXXXXTTGATATATATTTTCGTGTCGTGATTGAAATAGGTGAATACTTTGAAATTTAAAAAGAAGACTTGAAGAGCATATAGAGG

TTCTTTACCCCTTAAATTTCCAACATCATCAAATCTTTTAATTCATGTTTTAATTTTTAAAAAATGAATAAAAGAAGAACXXXXXXXXXXTTTATATATATTTTCGTGTCGTGATTGAAATAGGTGAATACTTTGAAATTTAAAAAGAAGACTTGAAGAGCATATAGAAG

>Marker39942

AATGTAAGTCACCAACTGCCTTGAAGGGTAAGTAGTAAGGATAAGAATAATGCATGCAAGGAGGAGTTTGGTAACAAACTXXXXXXXXXXCGAGAGAAGACCGCCTTAATCATGTGCCCATTATGCAAATATAGCACGTTAAGGTTATCGCAACACCTAAGCATTTAATA

AATGTAAGTCACCAACTGCCTTGAAGGGTAAGTAGCAAGGATAAGAATAATGCATGCAAGGAGGAGTTTGGTAACAAACTXXXXXXXXXXCAAGAGAAGACCGCCTTAATCATGTGCCCATTATGCAAATATAGCACGTTAAGGTTATCGCAACACCTAAGCATTTAATA

>Marker39949

CCGATGAAACATCGGTCGGTAGAGAATTCAAGGCACGTATTCTGTCTCAAGAGTCGGCTGCGTTCTTGTCACGGTCGCTGXXXXXXXXXXAGGTGCATTGGGGATTACAGTCCCCGGGGGTGATAGTGGTGTCGGGGAAGGAGCGATTGACTCGACAACGATAGAGAAAT

CCGATGAAACATCGGTCGGTAGAGAATTCAAGGCACGTATTCTGTCTCAAGAGTCGGCTGCGTTCTTGTCACGGTCGCTGXXXXXXXXXXAGGTGCATTGGGGATTACAGTCCCCGGGGGCGATAGTGGTGTCGGGGAAGGAGCGATTGACTCGACAACGATAGAGAAAT

>Marker40029

TTCTGAAGATCTCCAAGAAGCTACCTTTTGTTAGAATTTATCTTTTTCTCATTGGTGTAGAGCTTGGGTCTTTTAATAGAXXXXXXXXXXGCATGCAAACTATGTTGTTATTTCAAGTTTTCAGCATATGAGTGATGTTACTTACTTAGCAGTGTGTCAGCATAAAATGG

TTCTGAAGATCTCCAAGAAGCTACCTTTTGTTAGAATTTATCTTTTTCTCATTGGTGTAGAGCTTGGGTCTTTTAATGGAXXXXXXXXXXGCATGCAAACTATGTTGTTATCTCAAGTTTTCAGCATATGAGTGATGTTACTTACTTAGCAGTGTGTCAGCATAAAATGG

>Marker40073

GTAGACTTGGCTCCTCCTGGACACACCACACCGCCACCTGCACTGCCGCCTCCACCCGCCAATCACCCTCTGCCTCCCCCXXXXXXXXXXGTGATCCATTCCGGAGCGACGTAGCCTCTGGTTCCACGAAGCTGTGTGAAGATGGAGGTGTGTTGTTTGTCCATTAATTT

GTAGACTTGGCTCCTCCTGGACACACCACACCGCCACCTGCACTCCCGCCTCCACCCGCCAATCACCCTCTGCCTCCCCCXXXXXXXXXXGTGATCCATTCCGGAGCGACGTAGCCTCTGGTTCCACGAAGCTGTGTGAAGATGGAGGTGTGTTGTTTGTCCATTAATTT

>Marker40128

TTAAAGCTTTGTCTTGTAATTGTATTAAGTTTGATTTTTAGGGAAAAAAGATTTTCAGTTTTCTCAAGTTATTAGCGTTGXXXXXXXXXXATTGTAAGATGAAGTTGTTGATAAAAATACAAAATAGAAAAAGAGCTATAAGTTGAAGAATGAAGACTAAAAAATTGATG

TTAAAGCTTTGTCTTGTAATTGTATTAAGTTTGATTTTTAGGGAAAAAAGATTTTCAGTTTTCTCAAGTTATTAGTGTTGXXXXXXXXXXATTGTAAGATGAAGTTGTTGATAAAAATACAAAATAGAAAAAGAGCTATAAGTTGAAGAATGAAGACTAAAAAATTGATG

>Marker40133

GAGATATCCTTTGTTCCTTTTAGCTTTAGGAAATTCCTCATCTTCTAGACATGGTCCTATTTATCTTAGCTAATGCCTTAXXXXXXXXXXAGTAACACCTAGCCAGAGGTCGCTTGCTCAGATTTTTGGGTGAGCTTAATAAAGAACACCCTCAATGTCTCCAGAGTCTA

GAGATATCCTTTGTTCCTTTTAGCTTTAGGAAATTCCTCATCTTCTAGACATGGTCCTATGTATCTTAGCTAATGCCTTAXXXXXXXXXXAGTAACACCTAGCCAGAGGTCGCTTGCTCAGATTTTTGGGCGAGCTTAATAAAGAACACCCACAATGTCTCCAGAGTCTA

>Marker40175

TTGTCATGCTCTTACAGTATTAAAGTGCAAGACTGCAAGAGATTGGTATCGCAACCAAAGATGCAGAAGCCGCATGCTTAXXXXXXXXXXACTACTTCCCAAACTTCCTCCTGATACATTGAATGGTGTAGAATAACTCATGATAAAAGTATTTGAAGATAACTTACACA

TTGTCATGCTCTTACAGTGTTAAAGTGCAAGACTGCAAGAGATTGGTATCGCAACCAAAGATGCAGAAGCCGCATGCTTAXXXXXXXXXXACTACTTCCCAAACTTCCTCCTGATACATTGAATGGTGTAGAATAACTCATGATAAAAGTATTTGAAGATAACTTACACA

>Marker40176

TTTAAAAAATATTATTATAATTAAACTATGAGCCTATTTGGAACAACTTAGAAAAAGGTTTTTCAAAAATTTATTCTTATXXXXXXXXXXTACCCCTTAGGGTTGAAGAATAATAAGAGCATGTGGGTTATTTTACATCCTAATCCTAATCTCCAACTAAAACTTAACAT

TTTAAAAAATATTATTATAATTAAACTATGAGCCTATTTGGAACAACTTAGAAAAAGGTTTTTCAAAAATTTATTTTTATXXXXXXXXXXTACCCCTTAGGGTTGAAGAATAATAAGAGCATGTGGGTTATTTTACATCCTAATCCTAATCTCCAACTAAAACTTAACAT

>Marker40220

TGTAGTTGCATCCACGCCATTTGTTACACTCCATTTAATTCCATCATTTTGATGATATCAAATCATTAACAAAAGCAACTXXXXXXXXXXCACTTTACATAAGATGTTTCGGTATTTTTTTCTTCAATCGACAAAGTAGTGAGCACAAATCTCTAATTCCAAAATCAATA

TGTAGTTGCATCCACGCCATTTGTTACACTCCATTTAATTCCATCATTTTGATGATATCAAATCATTAACAAAAGCAACTXXXXXXXXXXCACTTTACATAAGATGTTTCGGTATTTTTTTCTTCAATCGACAAAGTAGAGAGCACAAATCTCTAATTCCAAAATCAATA

>Marker40247

CCTCGTAGGGTTGCTATTTTGTATCTTATAATAAACTCGGTATAGACTTAAAGCATTCAAATCATATACTTATGGAAAGCXXXXXXXXXXTTAACAAATTAGAAACTAGTTTATAAAATATTTAAAAGTCACAAATACTTGTTTAACCCTCCAGTCTATAGATTTGCCAA

CCTCGTAGGGTTGCTATTTTGTATCTTATAATAAACTCGGTATAGACTTAAAACATTCAAATCATATACTTATGGAAAGCXXXXXXXXXXTTAACAAATTAGAAACTAGTTTATAAAATATTTAAAAGTCACAAATACTTGTTTAACCCTCCAGTCTATAGATTTGCCAA

>Marker40384

CAGTAACCAAAACAAAGACATTGGATTAAGTTGAGTTGGAAAATTGAAATGACAGAGTTCAAAGAATCATACTAAACGTGXXXXXXXXXXCAAATGACGAAACTGAATCTCATGTGGTCCGACTCGACTACCAATGTCAGTCACACAGTCAAATCCACATTATGTAACAT

CAGTAACCAAAACAAAGACATTGGATTAAGTTGAGTTGGAAAATTGAAATGACAGAGTTCAAAGAATCATACTAAACGTGXXXXXXXXXXCAAATGACGAAACTGAATCTCATGTGGTCCGACTCGACTACCAACGTCAGTCACACAGTCAAATCCACATTATGTAACAT

>Marker40534

TCATGAGACAAACTATTTCCTCAACTGAGATGCGTTAGATAACATGGGAGGTTCATCCTCGGTGGGTGACACTTCAGGTTXXXXXXXXXXAGGGGACAGTCCTATCTCTCCACACGCCCCTCATTTCAACTACACGATTGGTGTGTTGACCAAGACACGTTTATGATCTA

TCATGAGACAAACTATTTCCTCAACTGGGATGCGTTAGATAACATGGGAGGTTCATCCTCGGTGGGTGACACTTCAGGTTXXXXXXXXXXAGGGGACAGTCCTATCTCTCCACACGCCCCTCATTTCAACTACACGATTGGTGTGTTGACCAAGACACGTTTATGATCTA

>Marker40579

CCTTGTTCAAGACTTAGATTCTGCCATTTAAGGAACAACCATCTTGCACTTTATGTTCTCAGCTATCCACTTGATTTACCXXXXXXXXXXCTCTAATATTATATAAACTCTTTACATAGGATGCCCGCACTTCCATGTCTTTACATGAACGATTCAGGATCACATCATTT

CCTTGTTCAAGACTTAGATTCTGCCATTTAAGGAACAACCATCTTGCACTTTATGTTCTCAGCTATCCACTTGATTTACCXXXXXXXXXXCTCTAATCTTATATAAACTCTTTACATAGGATGCCCGCACTTCCATGTCTTTACATGAACGATTCAGGATCACATCATTT

>Marker40888

TTTTCTTAATGTTTCATTTGCCATTACATACCATATTCAGTATTTATGTTTACTTTCTCTCTTCTTTTTCCATAAATCTTXXXXXXXXXXACAACAACTACTCGTCATGTCTATGATATCGCTGCTCAGCATGTTGTTAGTGGCGCTATGGAGGGTGTTAATGGTAAGCT

TTTTCTTAATGTTTCATTTGCCATTACATATCATATTCAGTATTTATGTTTACTTTCTCTCTTCTTTTTCCATAAATCTTXXXXXXXXXXACAACAACTACTCGTCATGTCTATGATATCGCTGCTCAGCATGTTGTTAGTGGCGCTATGGAGGGTGTTAATGGTAAGCT

>Marker40987

TCGTCTTCCTTCTAACTCAGCTTGCAGTGGCAGCTTTCATGTTTGGAAACACTTGCAAGACTGCATTTGAAGCTCTAATCXXXXXXXXXXACTAACTACTACAGAAGTCCAGACATGGGTGACACCGTCGAATTCTCAATCGGTTTTGAAACACCAGTGGAGAGGATTGG

TCGTCTTCCTTCTAACTCAGCTTGCAGTGGCAGCTTTCATGTTTGGAAACACTTGCAAGACTGCATTTGAAGCTCTAATCXXXXXXXXXXACTAACTACTACAGAAGTCCAGACATGGGTGACACCGTCGAATTCTCAATCGGTTTCGAAACACCAGTGGAGAGGATTGG

>Marker41014

ACTTAACTTTAGAGTTCTTATGATTGTGCCACCAAAATGGAAGAGACACTTTGTTGGTATAGCTGTATAGATAGTAATTTXXXXXXXXXXGTCATCGTGCTAGACTAAAGGATTTTAGCCCATCCATGAAAAGGAATACTCTCATCACCTTATCATCCAAAATATTTCGT

ACTTAACTTTAGAGTTCTTATGATTGTGCCACCAAAAAGGAAGAGACACTTTGTTGGTATAGCTGTATAGGTAGTAATTTXXXXXXXXXXGTCATCGTGCTAGACTAAAGGATTTTAGCCCATCCATGAAAAGGAATACTCTCATCACCTTATCATCCAAAATATTTCGT

>Marker41133

TATTTAGTTGCAAGTTGTGATTATGAGAATAATACAATATGGGGTTTGAAGGTATGGTGGTTAAAATTCAAATGATGATGXXXXXXXXXXGATGTGCCCATAACCCTTCTTAGTGTGATGAATCAAACCAAAAGATGGGGTATTGGGCTTTTAGAAGTCAATTTCTCAAA

TATTTAGTTGCAAGTTGTGATTATGAGAATAATACAAAATGGGGTTTGAAGGTATGGTGGTTAAAATTCAAATGATGATGXXXXXXXXXXGATGTGCCCATAACCCTTCTTAGTGTGATGAATCAAACCAAAAGATGGGGTATTGGGCTTTTAGAAGTCAATTTCTCAAA

>Marker41343

ATCGATTTGAGGGTGGTTACTTGATGATATATTCAAACTTTGTCCCCAAGAAAGCGAACAATTCGATCCAAAATGTGATAXXXXXXXXXXCAGAACTAGTAGGGTTCAAGAAGTTCAAAAATCTTTGCCTTTTCAACTTCATCTTACTGGCAAACTAAATCAATCTTAAT

ATCGATTTGAGGGTGGTTACTTGATGATATATTCAAACTTTGTCCCTAAGAAAGCGAACAATTCGATCCAAAATGTGATAXXXXXXXXXXCAGAACTAGTAGGGTTCAAGAAGTTCAAAAATCTTTGCCTTTTCAACTTCATCTTACTGGCAAACTAAATCCGTCTTAAT

>Marker41412

TGTCTCTGTTTCTCTATTTTTGTTGGGATTTTACTCAGTTTCAAAGAATGAATGCTAACAAATTGGTAACAGAGCCGCAAXXXXXXXXXXATGAATCAGTCCTTGAAGGGGAAAATTGAAGGAACGGAAGGGTCGATGCCTCTAGGACAAGGAATGTCAAACAAAAGTAG

TGTCTCTGTTTCTCTATTTTTGTTGGGATTTTACTCAGTTTCAAAGAATGAATGCTAACAAATTGGTAATAGAGCCGCAAXXXXXXXXXXATGGATCAGTCCTTGAAGGGGAAAATTGAAGGAACGGAAGGGTCGATGCCTCTAGGACAAGGAATGTCAAACAAAAGTAG

>Marker41620

CTTTTAATTTTGAACAACGACACATACAAAATGTGGAAAAATGGAAAGGTTAACAGTTAAATGAAAATGAAAATTCCAACXXXXXXXXXXATATTGTAGTATAGCATGGATGCCAAAATGCATAAAACATTCTACGCGTGACAGTGAACAAAATTTCATGGAGATAATAA

CTTTTAATTTTGAACAACGACACATACAAAATGTGGAAAAATGGAAAGGTTAACAGTTAAATGAAAATGAAAATTCCAACXXXXXXXXXXATATTGTAGTATAGCATGGATGCCAAAATGCATAAAACATTCTACGCGTGACAGTGAACAAAATTTCATGGAGATAATGA

>Marker41646

ATATAATAAATTAAAAGATCATTCCAATGATGGGCATCTTCCACATTCTACTCATAAGTGATATCTCGAACTACACTTGCXXXXXXXXXXAATTTGGAGGTGAAAATGAATCCCAAAAAATTTCTGCAAATATGCGAAGCACTAGCAGTAGCAGTGTGAAGATGGTGGGA

ACATAATAAATTAAAAGATCATTCCAATGATGGGCATCTTCCACATTCTACTCATAAGTGATATCTCGAACTACACTTGCXXXXXXXXXXAATTTGGAGGTGAAAATGAATCCCAAAAAATTTCTGCAAATATGCGAAGCACTAGCAGTAGCAGTGTGAAGATGGTGGGA

>Marker41778

AGAGTTAAGTTGCTGCATGTTTCCAAACCGTGAACCAGGATTAAAACCAGCAATCTGAGAGTTTGGAGGTGTGAGATTTGXXXXXXXXXXCAAAGATGTAGATTCTGCAAGAGAGGAAGAATGTGGATGTGATGACCATCTTTTGCCTTCCTGAGCACTTTCAACATACT

AGAGTTAAGTTGCTGCATGTTTCCAAACCGTGAACCAGGATTAAAACCAGCAATCTGAGAGTTTGGAGGTGTGAGATTTGXXXXXXXXXXCAAAGATGTAGATTCTGCAAGAGAGGAAGAGTGTGGATGTGATGACCATCTTTTGCCTTCCTGAGCACTTTCAACATACT

>Marker41871

TTTGAATATATGCACGGAAATGATTAAACGATAAAATCTTTTTTTGTTAGAATAAGTTTTGCGATTAAAAAGTTAATTCAXXXXXXXXXXAACATATATAACTATGCGATAAGGTTATTCATGAAAATTATTATGGTTAAAGTTATTTGCGGTATGGGGTTAGGCGAGAA

TTTGAATATATGCGCGGAAATGATTAAACGATAAAATCTTTTTTTGTTAGAATAAGTTTTGCGATTAAAAAGTTAATTCAXXXXXXXXXXAACATATATAACTATGCGATAAGGTTATTCGTGAAAATTATTATGGTTAAAGTTATTTGCGGTATGGGGTTAGGCGAGAA

>Marker41908

TACCAATCCCATTATCTCAGAGTTAACGAGGAATTTCTGTGAATTCTTAGTTTTGGCAAATACTCTGTTGATATTTTTCAXXXXXXXXXXAAAGAAAAACACTTTAGAAAGAGAGTGAATTTTTAAAACCCCACCTTGTTGTTTTTGTTAAAAGCATATATAAATACACA

TACCAATCCCATTATCTCAGAGTTAACGAGGAATTTCTGTGAATTCTTAGTTTTGGCAAATACCCTGTTGATATTTTTCAXXXXXXXXXXAAAGAAAAACACTTTAGAAAGAGAGTGAATTTTTAAAACCCCACCTTGTTGTTTTTGTTAAAAGCATATATAAATACACA

>Marker41916

ATTTTTCAATCTGAACAGAAGATTTCGACCTCAGTTTGATGAAAGAAGGAGAGAACCAACAGAATACAAGATGAAAGTTGXXXXXXXXXXTATTGCCTCATGGGAGAAAATGAAAAAATAATGAAGACACGTTTCCTTCCTTCCAATTATGAGCAAACTCTTTACAATCA

ATTTTTCAATCTGAATAGAAGATTTCGACCTCAGTTTGATGAAAGAAGGAGAGAACCAACAGAATACAAGATGAAAGTTGXXXXXXXXXXTATTGCCTCATGGGAGAAAATGAAAAAATAATGAAGACACGTTTCCTTCCTTTCAATTATGAGCAAACTCTTTACAATCA

>Marker42449

CAATTCAAATACCGGTGGTAATCTAGATAAGGCATCAGCCGCTTTGTTTTCCAATCCCTTGTATTCAATCACAAAATCATXXXXXXXXXXGTCGTTGATGCTGCATTAGGACAGCCCAATACCCACACTCGAAGCATCAGTTTCCAACACAAAACTGTGTAAAATCAGGA

CAATTCAAATACCGGTGGTAATCTAGATAAGGCATCAGCCGCTTTGTTTTCCAATCCCTTGTATTCAATCACAAAATCATXXXXXXXXXXGTCGTTGATGCTGCATTAGGACAGCCCAATACCCACACTTGAAGCATCCGTTTCCAACACAAAACTGTGTAAAATCAGGA

>Marker42600

ATAGTTGTGTGGTAATATGCATCCTCTTTATGATCATTCCAATCCTGAACAGGAACCTTTGAGATCAATTGATCAAATCTXXXXXXXXXXTGTCACCAGTGCATCTAACATTATCTAATGAAGGACGTGGGCTATCTGAATTTGAAGATACTACCGAAATAGAAGATTGA

ATAGTTGTGTGGTAATATGCATCCTCTTTATGATCATTCCAATCCTGAACAGGAACCTTTGAGATCAATTGATCAAATCTXXXXXXXXXXTGTCACCAGTGCATCTAACATTATCTAATGAAGGACGTGGGCTATCTGAATTTGAAGATACTACCGAAATAGAAGATTGG

>Marker42627

TAGGATACGAGTCTATTATTGATAGACTACTATCAGTGATGGACTAGGATACAAATCTATCATTGATATACTACTATCAAXXXXXXXXXXAGTCTATCATTGATAGACTGCTATCAGTTATAACTTACTATAAGTGATAACTTAGGATATTAGTAATACACTAGGATACA

TAGGATACGAGTCTATTATTGATAGACTACTATCAGTGATGGACTAGGATACGAATCTATCATTGATATACTACTATCAAXXXXXXXXXXAGTCTATCATTGATAGACTGCTATCAGTTATAACTTACTATAAGTGATAACTTAGGATATTAGTAATACACTAGGATACA

>Marker42629

CTACGAATCCAACATGACTGAGGAAGATTCCTACATTTCCACTTTACTTTGTGGTATTGAACTCATTCATTCGGGCGTAAXXXXXXXXXXGTGTTAAACTTGGTTAATGGAGGAATTTCTTTTTAAGGTAATTTCATTGATTGAATAAAAAATCCAAGGATATACAAAAG

CTACGAATCCAACATGACTGAGGAAGATTCCTACATTTCCACTTTACTTTGTGGTATTGAACTCATTCATTCGGGTGTAAXXXXXXXXXXGTGTTAAACTTGGTTAATGGAGGAATTTCTTTTTAAGGTAATTTCATTGATTGAATAAAAAATCCAAGGATATACAAAAG

>Marker42744

CAAATCTCTGCTCTATGGTGAGTCATGTCTTCCCATGACTTGATTATTATAAGTAGGAACTAACTATCACATCCTCCCCCXXXXXXXXXXGTCTCCTATTAGATAAGTAACTTTAAATCAAGACAACTTAAAATCTCATTCAAGAAAAATTCACTGTTTACAAGATATAT

CAAATCTCTGCTCTATGGTGAGTCATGTCTTCCCATGACTTGATTATTATAAGTAGGAACTAACTATCACATCCTCCCCCXXXXXXXXXXGTCTCCTATTAGATAAGTAACTTTAAATCAAGACAACTTAAAATCTCATTCAAGAAAAATACACTGGTTACAAGATATAT

>Marker42910

AATAGGATAATGCAAGAACAGAAGGTGGCAATGAGCACTTAACCATTTATTAGGTGATGAGCTAGGCGAGGAGGAATATGXXXXXXXXXXTAGAAAAGAGAAATTTCTATTAGGGGAGTAAAATGTGAAGTTTCGTTATAGGACTAGGCAACAAGAGGGAAGAAGAGTTG

AATAGGATAACGCAAGAACAGAAGGTGGCAATGAGCAGTTAACCATTTATTAGGTGATGAGCTAGGCGAGGAGGAATATGXXXXXXXXXXTAGAAAAGAGAAATTTCTATTAGGGGAGTAAAAGGTGAAGTTTCGTTATAGGACTAGGCAACAAGAGGGAAGAAGAGTTA

>Marker43161

ATATATATACTTCAACCACAAAATGCCTCCATCTTCCAAAAAACCATTCACATGAATGGAGTGTTCAAAGAAGCAATCCAXXXXXXXXXXAATTTAAAAGAAACAAAGTAGAGACATAGATTAATTGATTACCAATAACTAATTTTTGTCTAGCTTCTTCCATAAGTAGA

ATATATATACTTCAACCACAAAATGCCTCCATCTTCCAAAAAACCATTCACATGAATGGAGTGTTCAAAGAAGCAATCCAXXXXXXXXXXAATTTAAAAGAAACAAAGTGGAGACATAGATTAATTGATTACCAATAACTAATTTTTGTCTAGCTTCTTCCATAAGTAGA

>Marker43421

AAGTTGTGGTGCTTCTTAACTGGCTGTTCCTTGACGAACTTATTTTCCTAACGCTGATAAAAAATATTGCGGATATTATTXXXXXXXXXXCTGGTGTTGTTTTATTTCGTTTTGTTATTTATTTTGCTTTATGGATAGGGTTAAGAGAGAGATTCAATGATATGTTGAAG

AAGTTGTGGTGCTTCTTAACTGGCTGTTCCTTGACGAACTTATTTTCCTAACGCTGATAAAAAATATTGCGGATATTATTXXXXXXXXXXCTGGTGTTGTTTTATTTCTTTTTGTTATTTATTTTGCTTTATGGATAGGGTTAAGAGAGAGATTCAATGATATGTTGAAG

>Marker43469

TTAAGTTTCTTTCAAGGTTCGAGGTTTACACACTATTAAACAATTGGTTTATACGGTCCTTCTAATACTCTCCTTCAATGXXXXXXXXXXCTATTTTATTCTTTGTGCTTGAGCTTTTTCTACTCAACACGTTAGCTATAACATTAGCCATTTTAGGGTGATACGCGATA

TTAAGTTTCTTTCAAGGTTCGAGGTTTACACACTATTAAACAATTGGTTTATACGGTCCTTCTAATACTCTCCTTCAACGXXXXXXXXXXCTATTTTATTCTTTGTGCTTGAGCTTTTTCTACTCAACACGTTAGCTATAACATTAGCCATTTTAGGGTGATACGCGATA

>Marker43494

TAATGCTTCGAACTGTCCCATTTTTCGAATTTAAGGTGAAATTTGCTCCATGCCTGCCATTTACCTAATGCACCAATTAAXXXXXXXXXXTCGAAGGGAATGACAAACTGAGACTTGAGAAGGATCTTTATGGTTGGAATCCGGGCGTGGTTCTATTTTTCCAGATTTAG

TAATGCTTCGAACTGTCCCATTTTTCGAATTTAAGGTGAAATTTGCCCCATGCCTGCCATTTACCTAATGCACCAATTAAXXXXXXXXXXTCGAAGGGAATGACAAACTGAGACTTGAGAAGGATCTTTATGGTTGGAATCCGGGCGTGGTTCTATTTTTCCAGATTTAG

>Marker43663

TTTATACCATTTATTTCGTTTTAAAATCAACAAAGCAACCTGCAAACATAGAGATAAAATCACATTGCAATCTATACATAXXXXXXXXXXCAAATTATTTGAACATAGATACTAAAATCTTAGACCATTAAATAATAGACTAACATAGATATCCCATACAAAATCGATAA

TTTATACCATTTATTTCGTTTTAAAATCAACAAAGCAACCTGCAAACATAGAGATAAAATCACATTGCAATCTATACATAXXXXXXXXXXCAAATTATTTGAACATAGATACTAAAATCTTAGACCATTGGATAATAGACTAACATAGATATCCCATACAAAATCCATAA

>Marker43671

TATGTGTTCTAAGATGAAGGGAGTCAAAATGATTAAGTATAAGAATGATACATTTTAAGAAAGGAGCATGCGTTCTGGCAXXXXXXXXXXAAACCTACACTTGTAAGGCACTAAGTAGGAGCCAACAAACCAAGATGAGTAATCCAATTTAGGATTAATTAGTATAAAAA

TATGTGTTCTAAGATGAAGGGAGTCAAAATGATTAAGTATAAGAATGATACATTTTAAGAAAGGAGCATGCGTTCTGGCAXXXXXXXXXXAAACCTACACTTGTAAGGCACTACGTAGGAGCCAACAAACCAAGATGAGTAATCCAATTTAGGATTAATTAGTATAAGAA

>Marker43754

TAGCTAGTAGCAAGAGGGTTAAGTTAGGTTGTAAAATCGATTTGAGATTCTAATCAAGAAACATTTTTCTAACTTAAGTAXXXXXXXXXXTAATAGGTGACTCAATTATCTGAAATCTAGAAGAATCAATATTCCAATATACCAAAACTAAGCGCTCTTTTTAATTGGTT

TAGCTAGTAGCAAGAGGGTTAAGTTAGGTTGTAAAATCGATTTGAGATTCTAATCAAGAAACATTTTTCTAACTTAAGTAXXXXXXXXXXTAATAGGTGACTCAATTATCTGAAATCAAGAAGAATCAATATTCCGATATACCAAAACTAAGCGCTCTTTTTAATTGGTT

>Marker44119

CATGATTAGGACCAGGATTTGGAATTCGGATCTCCGACATTCCCCTGCATCTCCTGCGACCTGACTGCATCATCTTTACTXXXXXXXXXXCAGATGTGATGCCAGTTCATTCATGTCTCCCTCCTATTCTCGGACGTTACAGATGATGACCAATTGATGTATTTATCTTT

CATGATTAGGACCAAGATTTGGAATTCGGATCTCCGACATTCCCCTGCATCTCCTGCGACCTGACTGCATCATCTTTACTXXXXXXXXXXCAGATGTGATACCAGTTCATTCATGTCTCCCTCCTATTCTCGGACGTTACAGATGATGACCAATTGATGTATTTATCTTT

>Marker44254

AATAATTTGTTATCTTTTTTGTTATTGGCTTTGTTCTCTCAAACCCTCCCATGATTAATATTTTGAACTCCCTAAACCTCXXXXXXXXXXGATTCCCCTTTAAGAATCTTTGGGGTTTAACATATATTTATTGCAATGTGTATTAAAGTGGATTCCCATGATAAGGTGGT

AATAATTTGTTGTCTTTTTTGTTATTGGCTTTGTTCCCTCAAACCCTCCCATGATTAATATTTTGAACTCCCTAAACCTCXXXXXXXXXXGATTCCCCTTTAAGAATCTTTGGGGTTTAACATATATTTATTGCAATGTGTATTAAAGTGGATTCCCATGATAAGGTGGT

>Marker44837

CATCGTCACCTTGTAGAGTCAAGTAAGTAACCTGGTAAAAGAGATGGAAACCAGATAATAGATAAGTTAGAATAACACTGXXXXXXXXXXATTATAAATTTTATCCATGGATGATAGCTACCAATAGAATTTCTATGATTGATAGGAGATTAGTAGATGGACAGGAGTTC

CATCGTCACCTTGTAGAGTCAAGTAAGTAACCTGGTAAAAGAGATGGAAACCAGATAATAGATAAGTTAGAATAACACTGXXXXXXXXXXATTATAAATTTTATCCATGAATGATAGCTACCAATAGAATTTCTATGATTGATAGGAGATTAGTAGATGGACAGGAGTTC

>Marker44848

AATATTGGACACATCATCACCTTATGATGTTGGGTTTTTTGTTTTTATGGTCAAAATTCCATACGTTTTTTGTTAACCTTXXXXXXXXXXCTTACTATTGCATCAGGAGAGATGGAGAATGCATGCATTGATGATGGTGATAGGATTGGAGGATTTTGATGGAGATATTT

AATATTGGACACATCATCACCTTATGATGTTGGGATTTTTGTTTTTATGGTCAAAATTCCATACGTTTTTTGTTAACCTTXXXXXXXXXXCTTACTATTGCATCAGGAGAGATGGAGAATGCATGCATTGATGATGGTGATAGGATTGGAGAATTTTGATGGAGAGATTT

>Marker44880

CCACACGCACGATCTCGTCCTCTTTTTCCTTCCTCATTAATAATTTGTCCATTTACTTTTCTGCCCCTCCTTTGATATGTXXXXXXXXXXTCTCCAGAGGTCATCCATGCTAACTCAATGCGTTCGCTATTTTCCTTATATCTTATGAGTGACTTTATTCTCTCTCATAA

CCACACGCACGATCTCCTCCTCTTTTTCCTTCCTCATTAATAATTTGTCCATTTACTTTTCTACCCCTCCTTTGATATGTXXXXXXXXXXTCTCTAGAGGTCATCCATGCTAACTCAATGCGTTCGCTATTTTCCTTATATCTTATGAGTGACTTTATTCTCTCTAATAA

>Marker45144

TTATTTCGCAAATTCTTTTATCGGAAGCCACCCGACGGGCTTTTAGAGATATCCGAAAGGGTTTATGGTATGTCCTACTTXXXXXXXXXXCTAAACGGAATACCAAGTTACATTATGGTATTTTATAGGAAAATTTGTTACTAAAAGTTACTCTAGCTGTTGTTTTTGTG

TTATTTCGCAAATTCTTTTATCGGAAGCCACCCGACGGGCTTTTAGAGATATCCGAAAGGGTTTATGGTATGTCCTACTTXXXXXXXXXXCTAAACGGAATACCAAGTTACATTATGGTATTTTATAGGAAAGTTTGTTACTAAAAGTTACTCTAGCTGTTGTTTTTGTG

>Marker45176

TTGCTACACACTATTCGCTCACTGCAGTTTGTTTTCCGTTCCTTCATCCTTCTTTTGATTTCTTACTCTCATTCATTCACXXXXXXXXXXTCTGTAGATTATAAGTCCTAGCTCCTATGGATGTTAATACGAAAAAAATTGCATGAAGTTGATTTTACTTTTGAGAATTT

TTGCTACACACTATTCGCTCACTGCAGTTTGTTTTCCGTTCCTTCATCCTTCTTTTGATTTCTTACTCTCATTCATTCACXXXXXXXXXXTGTGTAGATTATAAGTCCTAGCTCCTATGGATGTTAATACGAAAAAAATTGCATGAAGTTGATTTTACTTTTGAGAATTT

>Marker45471

TAAAGTTTACTCTAAGTATTACTATAAAGAGAAAGCTTGCATAATGTATTTTGGGAACGCATAATGTTGAGTAATAAAAGXXXXXXXXXXCAAGTTGCGTCGATTACTATAATGCAAGGTTAGGACACGTGTAGGACAGAGCATGACAAGAACACCACCTGGAAACTAGA

TAAAGTTTACTCTAAGTATTACTATAAAGAGAAAGCTTGCATAATGTATTTTAGGAACGCATAATGTTGAGTAATAAAAGXXXXXXXXXXCAAGTTTCGTCGATTACTATAATGCAAGGTTAGGACACGTGTAGGACAGAGCATGACAAGAACACCACCTGGAAACTAGA

>Marker45673

CAAATCATACAAATTTACACTAGTCCGAAACGTAAGAGATACCTAAAGAAAAATACATGATAACAAATACTACAAATTTAXXXXXXXXXXAAACCTTGAACTAGGTAAGTGATACATACATCATAAAGCGTATTAATGATGTTGTTTCCTGAAGGATTGCAACAAATATT

CAAATCATACAAATTTACACTAGTCTGAAACGTAAGAGATACCTAAAAAAAAATACATGATAACAAATACTACAAATTTAXXXXXXXXXXAAACCTTGAACTAGGTAAGTGATACATAAATCATAAAGCGTATTAATGATGTTGTTTCCTGAAGGATTGCAACAAATATT

>Marker45939

TTTTGCGTAAAATGGAGTTAAAACATGAGATAAAGTGTCCGAATGTATAACTTTTAGGGCAAAATCATAATATCAAAAGAXXXXXXXXXXGATGTGACAAGAAATGATTACTTTCTGCATTGACATTATTTATAGACAAGGACAATTGTGCAAGGTAGGTAAGGACATAC

TTTTGCGTAAAATGGAGTTAAAACATGAGATAAAGTGTCCGAATGCATAACTTTTAGGGCAAAATCATAATATCAAAAGAXXXXXXXXXXGATGTGACAAGAAATGATTACTTTCTGCATTGACATTATTTATAGACAAGGACAATTGTGCAAGGTAGGTAAGGACAGAC

>Marker46233

CCATTCCCTTCACCACACTTCTCGTGGACACTCTTGATCCTCTCCACAGATTTACATATTCCACTGCAACTCCAGTCAAAXXXXXXXXXXACATCATTCATATCTAATGAAAACATTACCTGGCGGCGTCCCACAGCACATATTGCGGTCATCAATATGCTCAACTTCAA

CCATTCCCTTCACCACACTTCTCGTGGACACTCTTGATCCTCTCCACAGATTTGCATATTCCACTGCAACTCCAGTCAAAXXXXXXXXXXACATCATTCATATCTAATGAAAACATTACCTGGCGGCGTCCCACAGCACATATTGCGGTCATCAATATGCTCAACTTCAA

>Marker46503

TTTGGGCTCACAGAAATCAACATCGGTTTTGATCCACGACAATAATCCCGTCACAAATGTTTCTTCCACCACCTTCTCCGXXXXXXXXXXTACCAATTCAGAGCAAGTCCTTCGAAACTAATCATGGCAACCATCATTTTTTCAGATTTAGAAAGTTTGTGGATTTGGAA

TTTGGGCTCACAGAAATCAACCTCGGTTTTGATCCACGACAATAATCCCGTCACAAATGTTTCTTCCACCACCTTCTCCGXXXXXXXXXXTACCAATTCAGAGCAAGTCCTTCGAAACTAATCATGGCAACCATCATTTTTTCAGATTTAGAAAGTTTGTGGATTTGGAA

>Marker46526

TGAAAGCAAGTGGATATTGAATCTCTTGGCTTGGTCTTTGGCTCTCTTGACCATGTATTCGAGGGTCTATTTAGGATACCXXXXXXXXXXGAGTATGATAATGCAAGAGCTGCCAGACAGAAGTTGGATTGTAAGTGTGATTGATCGAGTTCGATCTGATATGTGAGAAG

TGAAAGCAAGTGGATATTGAATCTCTTGGCTTGGTCTTTGGCTCTCTTGACCATGTATTCGAGGGTCTATTTAGGATACCXXXXXXXXXXGAGTATGATAATGCAAGAGCTGCCAGACAGAAGTTGGATTGTAAGTGTGATTGATCGAGTTGGATCTGATATGTGAGAAG

>Marker46676

AATGAATCATCATCTGATTAGGAATGCTTCTTCTCAAAATTAACTTAGATTCATTTTTCTGAACGCAAACTTCTTGTTGGXXXXXXXXXXTAGCAATTTTTTATTTATTACTGTTTCCTTTACATTTGGCTGTGAGTATTTTATATTAGGTTAAATATTAGTTAGTGGGT

AATGAATCATCATCTGATTAGGAATGCTTCTTCTCAAAATTAACTTAGATTCATTTTTCTGAACGCAAACTTCTTGTTGGXXXXXXXXXXTAGCAATTTTTTATTTATTACTGTTTCCTTTACATTTGGCTGTGAGTATTTTATATTAGGTTAAATCTTAGTTAGTGGGT

>Marker46831

CAATAAAGAGGCGCGAAGTTGTCCGCGAGTGAGACTCGGTGAGTGAGGAGAACCCTTCTGGGTTTCGGATAAGCAATGAAXXXXXXXXXXGAGGAACAGGGAGGGGATTTTCACCGCCAAACAAAGCAAGGTTGTTGTCCTTTGGGACCTTGATAACAAACCCCCTCGTG

CAATAAAGAGGCGCGAAGTTGTCCGCGAGTGAGACTCGGTGAGTGAGGAGAACCCTTCTGGGTTTCGGATAAGCAATGAAXXXXXXXXXXGAGGAACAGGGAGGGGATTTTCACCGCCAAACAAAGCAAGGTTGTTGTCCTTTGGGACCTTGACAACAAACCCCCTCGTG

>Marker47526

TCAAACATTAATAAATTTAAAAAAAAAAACGAAGGTTAGAAGAACTTTTTTGACGTCGTAACAAGTAAGAGGGAGTTTTAXXXXXXXXXXATCCATGTTTTTCATTGAAAAATGTCTTGTTGCATCCCCCTCCAAGACAACCTCCTCTCTTGATTTGAGAGACGGTATGA

TCAAACATTAATAAATTTTAAAAAAAAAACGAAGGTTAGAAGAACTTTTTTGACGTCGTAACAAGTAAGAGGGAGTTTTAXXXXXXXXXXATCCATGTTTTTCATTGAAAAATGTCTTGTTGCATCCCCCTCCAAGACAACCTCCTCTCTTGATTTGAGAGACGGTATGA

>Marker47641

ACTTAGCGATATGGGATCCATTTGGGAGATCGTTGATACTAAAAGGGAATTGGGATATTTGATTTTGGATTTCCATTTTTXXXXXXXXXXATTTGGAGCAGGTTCTTCCCCGATAAGGCTGCATCTCTGGCATTTGACATTCTTAAAGAACTAATCGAGGTTAGAACTTG

ACTTAGCGATATGGGATCCATTTGGGAGATCGTTGATACTAAAAGGGAATTGGGATATTTGATTTTGGATTTCCATTTTTXXXXXXXXXXATTTGGAGCAGGTTCTTCCCCGATAAGGCTGCATCTCTGGCATTTGACATTCTTAAAGAACTAATCGAGGTTAGAACTCG

>Marker47929

CATATAAGCGGTTTGGTGGCAGCCAAGGTGTGTAAATTTATTTGACGTTACAGTCCTTGGGACTTGTGATTGAATTCTTTXXXXXXXXXXTTTGTATTTACTGTAGGAATGTGCTAGTCCGTTTGAATATTGTGATATAGTTACTTCAACAACACACAAAAGTCTTCGAG

CATATAAGCGGTTTGGTGGCAGCCAAGGTGTGTAAATTTATTTGACGTTACAGTCCTTGGGACTTGTGATTAAATTCTTTXXXXXXXXXXTTTGTATTTACTGTAGGAATGTGCTAGTCCGTTTGAATATTGTGATATAGTTACTTCAACAACACACAAAAGTCTTCGAG

>Marker48149

TTACCCAACGTATTAGAAGTAGTGGATGCTACAAGAACCCTTGGCTGAGTTAAATACTGAACCTCGTCATAAAGCATCAAXXXXXXXXXXAAATTTTGGAAGCCTTGGTAAGCATTGTATTCTATTTCTAAATCTTCAAGCTAAGTAGCAAGAGCACTTTACTTTAGTTT

TTACCCAACGTATTAGAAGTAGTGGATGCTACAAGAACTCTTGGCTGAGTTAAATACTGAACCTCGTCATAAAGCATCAAXXXXXXXXXXAAATTTTGGAAGCCTTGGTAAGCATTGTATTCTATTTCTAAATCTTCAAGCTAAGTAGCAAGAGCACTTTACTTTAGTTT

>Marker48246

TTAGTTGCTTTTATTATTATTAATAATAATAATATAACAATACAACCAAAATAATCAGATGGGAAGCCACATTTTTGTAAXXXXXXXXXXGTGGTCTATCAACATGAGTAAGCTCCAAGCTAAACACGCTTAACTTTTAAGTTTCAATGATCGAGTCACTAAAAATGAAA

TTAGTTGGTTTTATTATTATTAATAATAATAATATAACAATACAACCAAAATAATCAGATGGGAAGCCACATTTTTGTAAXXXXXXXXXXGTGGTCTATCAACATGAGTAAGCTTCAAGCTAAACACGCTTAACTTTTAAGTTTCAATGATCGAGTCACTAAAAATGAAA

>Marker48353

TTCATTTCCACTACCTGTGAAAGCTTTTCTTTTATAAGCATATCATTTCTTACAGAATCTTGGTGTGCATTTGTTTGACAXXXXXXXXXXTCTGAATTTCTCATAAACACAACAAAGTTACAAAAACTTTATGGATTGACAAGAGAAAAAAGCGTTCTTCAATACAAAAG

TTCATTTCCACTACCTGTGAAAGCTTTTTTTTTATAAGCATATCATTTCTTACAGAATCTTGGTGTGCATTTGTTTGACAXXXXXXXXXXTCTGAATTTCTCATAAACACAACAAAGTTACAAAAACTTTATGGATTGACAAGAGAAAAAAGCGTTCTTCAATACAAAAG

>Marker48503

AATTTCATTGAGGACAAAATGAAAGAATACAATAGGTTAAAAAAACAAAGCCCCACAAATGAGCGCACCCTAAAGAAAGAXXXXXXXXXXCTATTGATGAAAATGAGGAGCTTAAACAAATGAAGCAATTTATTATTAAACATGAATAACATATTTCTTGGCTTTCATTA

AATTTCATTGAGGACAAAATGAAAGAATACAATAGCTTAAAAAAACAAAGCCCCACAAATGAGCGCACCCTAAAGAAAGAXXXXXXXXXXCTATTGATGAAAATGAGGAGCTTAAACAAATGAAGCAATTTATTATTAAACAGGAATAACATATTTCTTGGCTTTCATTA

>Marker48526

CCAGAGCAATAGTCTCTTTTCATTCCGTCAATGAAAAATTATGTTTATGTTTAAAAAAAAAGTTGTCGAACTTGAAATTCXXXXXXXXXXCTCTCAAGATGTCAAGTAGCAAGCTTTGCCTTTGTTAGAAGTCTCTTTGGTTGGAAGGATCTTTTGCCTGGTGAATTCTT

CCAGAGCAATAGTCTCTTTTCATTCCATCAATGAAAAATTATGTTTATGTTTTAGAAAAAAGTTTTCGAACTTGAAATTCXXXXXXXXXXCTCTCAAGATGTCAAGTAGCAAGCTTTGCCTTTGTTAGAAGTCTCTTTGGTTGGAAGGATCTTTTGCCTGGTGAATTCTT

>Marker48600

TCTTCCTACCACCACGTCTCACTCGGACCCTGTAAACCACATAACCCTGCAACAAAAAAGTTCACTCACCAATCACTATTXXXXXXXXXXAAATACTAATAACAGAAAATATTCCTTGTGCTATCAAAATGGTAGCAGACGCAGACAACAAAAATTCCTAATTAAAGAGT

TCTTCCTACCACCACGTCTCACTCGGACCCTGTAAACCACATAACCCTGCAACAAAAAAGTTCACTCACCAATCACTATTXXXXXXXXXXAAATACTAATAACAGAAAATATTCCTTGTGCTATCAAAATGGTAGCAGACGCAGACAACAAAAATTCCAAATTAAAGAGT

>Marker48980

TCTAAAAGTAGGTTTCGAACCTATGACCAATCAATTAACAACCAACCTCTAAGCTAACAACTTATCTTCACTCGTCAACAXXXXXXXXXXAATTTGGGACAAAGCATCACAAAAAGAGATGCCTTTTCCTACTTTGGTCTAAAACACCATTACCAAACTGAGCTGTTTTT

TCTAAAAGTAGGTTTCGAACCTATGACCAATCAATTAACAACCAACCGCTAAGCTAACAACTTATCTTCACTCATCAACAXXXXXXXXXXAATTTGGGACAAAGCATCACAAAAAGAGATGCCTTTTCCTACTTTGGTCTAAAACACCATTACCAAACTGAGCTGTTTTT

>Marker49140

TTCATTAATCCTCAATATATTTCAGATAGTGATTCTTGTTACGAAGTATCCTAACATAGTCCCCTGAAGACAGCTCAGCCXXXXXXXXXXGTGAAAAGAAACAAGAAGCACAAGAATTCTAAAGTGTCACAAAGAAGTTCTAATTTGTTCAAGCTTTTGTGTAAACACTT

TTCATTAATCCTCAATATATTTCAGATAGTGATTCTTGTTACTAAGTATCCTAACATAGTCCCCTGAAGACAGCTCAGCCXXXXXXXXXXGTGAAAAGAAACAAGAAGCACAAGAATTCTAAAGTGTCACAAAGAAGTTCTAATTTGTTCAAGCTTTTCTGTAAACACTT

>Marker49242

ACCTAAAGATAGTTATGAATTCTCTGGTTACAATCTTCCATCAGGGGAGCAAATAAAAACCTTTTTTTTTTAAAATCTTCXXXXXXXXXXATCAAATTCATCATCCCTGAAATTTCTAAATGGAAGTTCATTAGCTTCCAGCCAGAAAAACCAACCATCTCATAAAGTAG

ACCTAAAGATAGTTATGAATTCTCTGGTTACAATCTTCCATCAGGGGAGCAAATAAAAACCTTTTTTTTTTAAATCTTCAXXXXXXXXXXATCAAATTCATCATCCCTGAAATTTCTAAATGGAAGTTCATTAGCTTCCAGCCAGAAAAACCAACCATCTCATAAAGTAG

>Marker49292

TTTGTTCTTTTTACCGTTGTTTAAGGTTAATCAATAATGATAGCTACAATTATAATTGACAAAGAAACCTTCAACTTCACXXXXXXXXXXACAAATCCAGACTTTATCTTTATAAGAAAGCTTGCCTTTTATTTTCCACAAGCAGCTATGAGTCTCACAAGTCACAAGGA

TTTGTTCTTTTTACCGTTGTTTAAGGTTAATCAATAATGATAACTACAATAATAATTGACAAAGAAACCTTCAACTTCACXXXXXXXXXXACAAATCCAGACTTTATCTTTATAAGAAAGCTTGCCTTTTATTTTCCACAAGCAGCTATGAGTCTCACAAGTCACAAGGA

>Marker49302

CAGAATCAGCACAGCCTTAAACAGCAAAAGCTTATAAGATACTGTAATGAAGGATCCTTTCTATTACCTATCAATCATACXXXXXXXXXXTAGTAAATAGATTATTTAATATAAAATGGTTCAAACAGCTCAATCCATAGAATTGAGGATATCGCATGATTACCAAGGGA

CAGAATCAGCACAGCCTTAAACAGCAAAAGCTTATAAGATACTGTAATGAAGGATCCTTTCTATTACCTATCAATCATACXXXXXXXXXXTAGTAAATAGATTGTTTAATATAAAATGGTTCAAACAGCTCAATCCATAGAATTGAGGATATCGCATGATTACCAAGGGA

>Marker49771

AAATAGATAAGCTACACATTTAAAACTTAGTTTTGGTCCTTTTTTGACTCATCATTGCTAGTCCAGACTCAATGAAGTTGXXXXXXXXXXAGGAAACGAAAGAGGATTTCACATTATATCTCCAAAGTCTAGCTAATATACGAGAAGAGACAAATTTATTGATAGTGTTA

AAATAGATAAGCCACACATTTAAAACTTAGTTTTGGTCCTTTTTTGACTCATCATTGCTAGTCCAGACTCAATGAAGTTGXXXXXXXXXXAGGAAACGAAAGAGGATTTCACATTATATCTCCAAAGTCTAGCTAATATACGAGAAGAGACAAATTTATTGATAGTGTTA

>Marker49867

CTACCTAGAGGAGATCATCTTAAAGTTAGCTCTTAGCTCAAATAGTTTCAAAATTGATTTTGAGAAGAATTTAGCTAAGTXXXXXXXXXXTTAAAAATAAAAATGATGATATTTCCTTGTCTCAAAGTGATCCAAACTTAGAAAAAGTCAATTCTAACGAAGAAAACAAG

CTACCTAGAGGAGATCATCTTAAAGTTAGCTCTTAGCTCAAATAGTTTCTAAATTGATTTTGAGAAGAATTTAACTAAGTXXXXXXXXXXTTTAAAATAAAAATGATGCTATTTCCTTGTCTCAAAGTGATCCAAACTTAGAAAAAGTCAATTCTAACGAAGAAAATAAG

>Marker49950

ACGGACGAATCGTGCCCGTATCTAGACCGGCAAGTGTCATGCGTGAAGAACGGACGGGAGGATTCGGATTACCGGCATTGXXXXXXXXXXCATTACAGCGTGGGCAATGGCAATCATTTGTCTGTATGGTTGAGTGGATGATTCCTGAAGATCAAAAATCTCTCAAGCGA

ACGGACGAATCGTGCCCGTATCTAGACCGGCAAGTGTCATGCGTGAAGAACGGACGGGAGGATTCGGATTACCGGCATTGXXXXXXXXXXCATTACAGCGTGGGCAATGGCAATCACTTGTTTGTATGGTTGAGTGGATGATTCCTGAAGATCAAAAATCTCTCAAGCGA

>Marker50158

TCTCAATTCTTAAAATTGTAAGCTTTTTTTAACTCTCAAATCTTCCCTTTTCTCATATTTTAGTTGAAAACTTAGTTCTCXXXXXXXXXXAAAGGAAAATGGTATGGAGGAAGAATACAATTATGTAGGTAAGTTCGAGAAAGGGGAGAAATTGACAATACAAAGCCAAA

TCTCAATTCTTAAAATTGTAAGCTTTTTTTAACTCTCAAATCTTCCCTTTTCTCATATTTTAGTTGAAAACTTAGCTCTCXXXXXXXXXXAAAAGAAAATGGTATGGAGGAAGAATACAATTATGTAGGTAAGTTCGAGAAAGGGGAGAAATTGACAATACAAAGCCAAA

>Marker50205

CATAAATAGAATACAAAAATAAATTTTGTATTTTAAATTTGAATCATCAATCCTATACAATAAAAAATTGTATTATGTTGXXXXXXXXXXTGGGAAAAGTGAAGTAATAAATGAGGAAAGAGAAGGAAAAATGGGTTTAATAGTATTCCTTTTCCTTCCCTTAATCCATG

TATAAATAGAATACAAAAATAAATTTTGTATTTTAAATTTGAATCATCAATCTTATACAATAAAAAATTGTATTATGTTGXXXXXXXXXXTGGGAAAAGTGAGGTAATAAATGAGGAAAGAGAAGGAAAAATGGGTTTAAGAGTATTCCTTTTCCTTCCCTTAATCCATG

>Marker50279

ACCTTTTTAACTCCCCTCACTTTCCTTTCCTTGCGATGCCTTCCACCATCACCTCCCTCTCTGCCACCTCGACTTTTCTTXXXXXXXXXXTATTCCATTTTAACGGTTTTCAAACCCTCGACTCCATCCTATTTGAATACATTTTAACTCCTAAACAAAGTATGAGGGTA

ACCTTTTTAACTCCCCTCACTTTCCTTTCCTTGCGATGCCATCCACCATCACCTCCCTCTCTGCCACCTCGACTTTTCTTXXXXXXXXXXTATTCCATTTTAACGGTTTTCAAACCCTCGACTCCATCCTATTTGAATACATTTTAACTCCTAAACAAAGTATGAGGGTA

>Marker50320

TTGTTAGCTTTGAAGAAGAAAAAGGCACAAGAAGAATTACTGAAGCAAGTTGATGCCTGGCTGATTAATGTTGAACAACAXXXXXXXXXXTGTTTAGAACTTTTTTGTATTTTAGAAATTTGTGTTCGTGCTTTAATATAGTTGTTGGCTCAGTTGTCAGATATTGAATT

TTGTTAGCTTTGAAGAAGAAAAAGGCACAAGAAGAATTACTGAAGCAAGTTGATACCTGGCTGATTAATGTTGAACAACAXXXXXXXXXXTGTTTAGAACTTTTTTGTATTTTAGAAATTTGTGTTCGTGCTTTAATATAGTTGTTGGCTCAGTTGTCAGATATTGAATT

>Marker50374

ACACCTTTCACATAATTACCAACGTCTGTCGCATCGGTTTTTCACGAGATAGTAATCTTACGAGTAAAGAGTGAATTGTGXXXXXXXXXXTTTATATGTTCTTGTTAAGTGCTTATTGAAAAATTACGGACAATTGTTTTTATCTCAAAGTTTAAGGAGCCTATGATAAA

ACACCTTTCACATAATTACCAACGTCTGTCTCATCGGTTTTTCACGAGATAGTAATCTTACGAGTAAAGAGTGAATTGTGXXXXXXXXXXTTTATATGTTCTTGTTAAGTGCTTATTGAAAAATTACGGACAATTGTTTTTATCTCAAAGTTTAAGGAGCCTATGATAAA

>Marker50437

CCTATACTTTAAAAATAGTAACAATTAGTCCTTTTGTTCAATTTTATGTTAACTTGCCGTCAAATCCCTCCTTTGAAGGCXXXXXXXXXXTTAGATGGGTTAAGGTAGGGGACTATAACACTGGCTTTTTCCATAGAATGGCTAGTGGTAGAAGGAACAAGAACTGCATC

CCTATACTTTAAAAATGGTAACAATTAGTCCTTTTGTTCAATTTTATGTTAACTTGCCGTCAAATCCCTCCTTTGAAGGCXXXXXXXXXXTTAGATGGGTTAAGGTAGGGGACTATAACACTGGCTTTTTCCATAGAATGGCTAGTGGTAGAAGGAACAAGAACTGCATC

>Marker50710

ATGCACAAGTCATTTACTTTTGTGGCATTACAGAAACAGGAGAAGCTAAAAGAATATTTGAGTTTGAGTTGTGATTTTCGXXXXXXXXXXTTAGCTTGACCATTTAGAATGATTCAAGTTTTTTTCAATCCTAAGTGTGTTGATAAGAACACTCTTTTGTGAATCTGATA

ATGCACAAGTCATTTACTTTTGTGGCATTACAGAAACAGGAGAAGCTAAAAGAATATTTGAGTTTGAGTTGTGATTTTCAXXXXXXXXXXTTAGCTTGACCATTTAGAATGATTCAAGTTTTTTTCAATCCTAAGTGTGTTGATAAGAACACTCTTTTGTGAATCTGATA

>Marker50882

CCAAAAAAGAAAAAAAAGAAAACCATTGAAGAAGGGAGCCCTCCTAAACAAGGAGCTCCAACTATATGAATTAGAATCTAXXXXXXXXXXCTTTTTTAGCGTATATTTACAAGTATGCAATGCATAAGGCACTAGATACGTTAAATTGAAAAGCTTATAACTTTAAAGTA

CCAAAAAAGAAAAAAAAGAAAACCATTGAAGAAGGGAGCCCTCCTAAACAAGGAGCTCCAACTATATGAATTAGAATCTAXXXXXXXXXXCTTTTTTAGTGTATATTTACAAGTATGCAATGCATAAGGCACTAGATACGTTAAATTGAAAAGCTTATAACTTTAAAGTA

>Marker51064

TTAATTCATGTCTTTTCTGCATTCTTTCCTCATCATCCTGAAGTTCCTGCTGCCGTATTCTAATCTTTGCTTCTATAACAXXXXXXXXXXTGGTGAATAGCTTTAAAGTAAACAAAAAAGACATATTATAAACATGGAGGTATGACATGCATGGTGAAGGATACTGTGTA

TTAATTCATGTCTTTTCTGCATTCTTTCCTCATCATCCTGAAGTTCCTGCTGCCGTATTCTAATCTTTGCTTCTATAACAXXXXXXXXXXTGGTGAATAGCTTTAAAGTAAACAAAAAAGACATAATATAAACATGGAGGTATGACATGCATGGTGAAGGATACTGTGTA

>Marker51112

CACGTTGTTAAAACCCCAAGGTATACATATAACCATTAACTTTAGAGTCTGGTGACCCTTTTCTAGAAAAAATGGATACTXXXXXXXXXXCATAAAACGGTGATTGGCATACATATCATTGTAGCTATGGACAACCTTCTGTTTACTTTCAAATATCATAAGGGTTGTAT

CACGTTGTTAAAACCCCAAGGTATACATATAACCATTAACTTTAGAGTCTGGTGACCCTTTTCTAGAAAAAATGGATATTXXXXXXXXXXCATAAAACGGTGATTGGCATACATATCATTGTAGCTATGGACAACCTTCTGTTTACTTTCAAATATCATAAGGGTTGTAT

>Marker51195

TTATGGAGTGACTCCAGATACATTTACATTTAACATTTTGATTAGAGGATTTTGTATGAATGGTATGGTTGATGATGGTTXXXXXXXXXXGCTGTTTTTGAAGAAATGGTTAATCAAGGATTGAAAGCCAACAACATAACCTACAATACTTTAATTAAGGGGCTTTGTGA

TTATGGAGTGACTCCAGATACATTTACATTTAACATTTTGATTAGAGGATTTTGTATGAATGGTATGGTTGATGATGGTTXXXXXXXXXXGCTGTTTTTGAAGAAATGGTTAATCAAGGATTGAAAGCAAACAACATAACCTACAATACTTTAATTAAGGGGCTTTGTGA

>Marker51766

TAAAAGGGAAAAGGAGATCATGAAGAAATGTAATGGTCACACCAATGGAGTAAAAACAAGTGAATTTTTGTTGACTTTTAXXXXXXXXXXGTGCAACTCGATTATTTCTTAATTCATCGGTATATATATATTAACCAACACAACGTTTTCATTTGGGGTTAGATCCTCAA

TAAAAGGGAAAAGGAGATCATGAAGAAATGTAATGGTCACACCAATGGAGTAAAAACAAGTGAATTTTTGTTGACTTTTAXXXXXXXXXXGTGCAACTCGATTATTTCTTAATCCATCGGTATATATATATTAACCAACACAACATTTTCATTTGGGGTTAGATCCTCAA

>Marker51995

TTGTAAAATTGCATGGCTATGACATTTCCCTTGGAAGCCCAGGCTTCTCTGCCTCACATTGTGGCATGCATCCTTGGTTCXXXXXXXXXXGTCCTCACCAAAGGTTACACTTATAGCATCTGTCCCTGTTATTTCAAGGGATGTTCTCTCTTTGAACTTCAGTTTTTGGT

TTGTAAAATTGCATGGCTATGACATTTCCCTTGGAAGTCCAGGCTTCTCTGCCTCACATTGTGGCATGCATCCTTGGTTCXXXXXXXXXXGTCCTCACCAAAGGTTACACTTATAGCATCTGTCCCTGTTATTTCAAGGGATGTTCTCTCTTTGAACTTCAGTTTTTGGT

>Marker52199

TCCAAAATCGCATTATTTGAAGCCTCCTACTTTTCTCCTAAATTTGAACATATTTCTAATCAGAATTGGCTAATGAGTGCXXXXXXXXXXAAAGATGGGCTGTGAAAACCATTCCAAACCTTACCAAAAACTTAACAAAGTGCCCTTAACTTCAATTAACAGCACCATAA

TCCAAAATCGCATTATTTGAAGCCTCCTACTTTTCTCCTAAATTTGAACATATTTCTAATCAGAATTGGCTAATGAGTGCXXXXXXXXXXAAAGATGGGGTGTGAAAACTATTCCAAACCTTACCAAAAACTTAACAAAGTGTCCTTAACTTCAATTAACAGCACCATAA

>Marker57248

TGTGACTACTAAATTCTTAATAGTTACATATTTGCAACTTGTGAGTTCCTAAGAGATGCAAGAAAATGAAGTGTTATTCTXXXXXXXXXXCCACTGTTTCTTATCTAAAAAAAGTTTCCTAAAGATCAATGTATAACATTTTGGTTCAAATATTTTTTCATCTGTTGACT

TGTGACTACTAAATTCTTAATAGTTACATATTTGCAACTTGTGAGTTCCTAAGAGATGCAAGAAAATGAAGTGTTATTCTXXXXXXXXXXCCACTGTTTCTTATCTAAAAAAAGTTTCCTAAAGATCAATGTATAACATTTTGGTTAAAATATTTTTTCATCTGTTGACT

>Marker57674

TTGCAAATCTATATGGTTTTTATTTAATACATCCATGCATGTAGATCAAAATATTTATAGCATAATTTCTATATCAAATTXXXXXXXXXXAAAATGTGTGATATACTTTTTACTCGAGATATGTTCGTTTAAATGTGTTGAAGGCAAAAATAGTAATTACATTGAAAATA

TTGCAAATCTATGTGGTTTTTATTTAATACATCCATGCATGTAGATCAAAATATTTATAGCATAATTTCTATATCAAATTXXXXXXXXXXAAAATGTGTGATATACTTTTTACCGGAGATATGTTCGTTTAAATGTGTTGAAGGCAAAAATAGTAATTACATTGAAAATA

>Marker57734

CAGGTGTATCAACAATGTTCATCTGCATTAAAAATAATCAGAAAAACTATAATTAGACATCAAATAACACATGTTCGTCTXXXXXXXXXXAAATTCAAGGTATAGCGTTCAGAGTAATATTTTGTAAGACTTGTAAATTGGGTTTTCTTTTAGAACTCAAGGACATAGAT

CAGGTGTATCAACAATGTTCATCTGCATTAAAAATAATCAGAAAAACTATAATTAGACATCAAATAACACATGTTTGTTTXXXXXXXXXXAAATTCAAGGTATAGCGTTCAGAGTAATATTTTGTAAGACTTGTAAATTGGGTTTTCTTTTAGAACTCAAGGACATAGAT

>Marker57895

CGAGTCCAAGCCTACCTAAGACCCATGATATTTCTCTATAAATAGAGACCTTTGACACTCATTTAAGAGGGGTGTTTGGTXXXXXXXXXXGAGATTGTATTCACAATATTCATCAATATACAAAGTTCAATTTTATGAATTACATCTCTTCTGAAATCTCGTATAAACCA

CGAATCCAAGCCTACCTAAGACCCATGAAATTTCTCTATAAATAGAGACCTTTGACACTCATTTAAGAGGGGTGTTTGGTXXXXXXXXXXGAGATTGTATTCACAATATTCATCAATATACAAAGTTCAATTTTATGAATTACATCTATTCTGAAATCTCGTATAAACAA

>Marker58041

AAAACATGTATCGAGCTACTGTAAAGTTATTGAAGGCTATAAACGGGAGTTCATTCTCTCACTTGGGCTCTTAGAGGAAGXXXXXXXXXXCTTTTCTTATGCAAGTAAGATTGGTCACGCGTTTGAGTTATTTTATGATATGATAAGAGATGGTGTTATACCAGATCTTG

AAAACATGTATCGAGCTACTGTAAAGTTATTGAAGGCTATAAACGGGAGTTCATTCTCTCACTTGGGCTCTTAGAGGAAGXXXXXXXXXXCTTTTCTTATGCAAGTAAGATTGATCACGCGTTTGAGTTATTTTATGATATGATAAGAGATGGTGTTATACCAGATCTTG

>Marker58418

TCATTATTTTAATAAAAGAGGTTGTAATAATGATAATGCGCATAAATGACTGTTATTATGGTCCTTTTTGTTAGAATAATXXXXXXXXXXAGAGCGACGTGTGAACGAATCACCCCACACACACAAAAGATGTCGGAATGGGTGCAAGTTTCAAACCTAGAAAGTCGGTG

TCATTATTTTAATAAAAGAGGTTGTAATAATGATAATGCGCATAAATGACTGTTATTATGGTCCTTTTTGTTAGAATAATXXXXXXXXXXAGAACGACGTGTGAACGGATCACCCCACACACACAAAAGATGTCGGAATGGGTGCTAGTTTCAAACCTAGAAAGTCAGTG

>Marker58699

ACCTCCGGAGACAATATGGTGTCTGTAATCTAAAACAAAAATTTCAAAATCAAACCAGTGCCAAATATAGAAAGACTAAAXXXXXXXXXXGAGAGATATTTTGAACATCAAAAAATAAAAACTGTCATTGTCAAACCAAAACCCAGCTAATTGTTGTAACCAGACAAAAT

ACCTCCGGAGACAATATGGTGTCTGTAATCTAAAACAAAAATTTCAAAATCAAACCAGTGCCAAATATAGAAAGACTTAGXXXXXXXXXXGAGAGATATTTTGAACATCAAAAAATAAAAACTGTCATTGTCAAACCAAAACCCAGCTAATTGTTGTAACCAGACAAAAT

>Marker58863

TCCTTCCTGGGAGTGAGCAAAGAATCAAAAGGAGTGAGCAAAGAACTAAAAGGATTGAACTAAACAATATGCAATGACCAXXXXXXXXXXCAGAAGTCCAACGCTCAATTAGTGATGAAGGTCCGCCTTTACTCGGGAGGCTATGGATACCGCTTGGGAGTAAAACAGTT

TCCTTCCTGGGAGTGAGCAAAGAATCAAAAGGAGTGAGCAAAGAACCAAAAGGATTGAACTAAACAATATGCAATGACCAXXXXXXXXXXCAGAAGTCCAACGCTCAATTAGTGATGAAGGTCCGCCTTTACTCGGGAGGCTATGGATACCGCTTGGGAGTAAAACAGTT

>Marker59247

CTACATAATTCATGACATTATTTTCTTCAAACCTACTACTTATTTGGTTATTTTGCAAGCTCCTCCTAGCCCTTTCGGAAXXXXXXXXXXACCACATAATGGTTATGATATTGACTAAGAAGGTTAAATAAAAGTTTATTCGACTAAAACCCATTTCATCGACATGTGAT

CTACATAATTCATGACATTATTTTCTTCAAACCTACTACTTATTTGGTTATTTTGCAAGCTCCTCCTAGCCCTTTCGGAAXXXXXXXXXXACCACATAATGGTTATGATATTGACTAAGAAGGTTAAATCAAAGTTTATTCGACTAAAACCCATTTCATCGACATGTGAT

>Marker59307

TTAATAGCCCTGTAAAGTGTAGAGCGATGAAGGATTTTTTAGCCATGTCTACAGTGGGTTTCTGTTGCATTCTAGAAAGTXXXXXXXXXXATGTGTGTATGCCTCTATTAGTAATATCGAGAGACGTGTTTTATGGCGGTGTGTGTCTAAGATTTCTGCTGGATGGAAAG

TTAATAGCCCTGTAAAGTGTAGAGCGATGAAGGATTTTTTAGCCATGTCTACAGTGGGTTTCTGTTGCATTCTAGAAAGTXXXXXXXXXXATGTGTGTATGCCTCTATTAGTAATATCGAGAGACGTGTTTTATGGCGGCGTGTGTCTAAGATTTCTGCTGGATGGAAAG

>Marker59356

AAAATTTGTATTTTTGTTTTTACAAAAAATTGACCAAATTATTTCTCTACCGAGATCCCCAACTATCGCCTTCCAATTTTXXXXXXXXXXTTAATTATGGTAGTTGATATGCATGTTAATCGCTAAATTAGTTTAGATGCAATTAAAGTAGAATTTATTTGTATTTCGCC

AAAATTTGTATTTTTGTTTTTACAAAAAATTGACAAAATTATTTCTCTACCGAGATCCCCAACTATCGCCTTCCAATTTTXXXXXXXXXXTTAATTATGGTAGTTGATATGCATGTTAATCGCTAAATTAGTTTAGATGCAATTAAAGTAGAATTTATTTGTATTTCGCC

>Marker59722

ATTAAAAATAGATTATTCAAGAAAACCCCCAGGAAATCTTTCTTTTCCACTGAAAAATCCCAGAATACCTCTCATTCCAAXXXXXXXXXXAAAAAAGGGGGTAAAACCCTATTTCAGTTGAGATACCTAACATTCGGACTTCTTTTGTGTAGATGACCTTTTTTACCGTG

ATTAAAAACAGATTATTCAAGAAAACCCCCAGGAAATCTTTCTTTTCCACTGAAAAATCCCAGAATACCTCTCATTCCAAXXXXXXXXXXCAAAAAGGGGGAAAAACCCTATTTCAGTTGAGATACCTAACATTCGGACTTCTTTTGTGTAGATGACCTTTTTTATCGTG

>Marker59781

TGGCGCTAGGGTCTTACCTTATTTATGTATTTTCTGAAATTCTGTATTCCATTGATATATCTTTAGCAATTGAGATATCTXXXXXXXXXXTCTGTTGGTCTGGTCATGCTTTTTGGCACATTATTCTTTATTTTTGGAAATCTATGTTGGATTTTATAGTAACTTTTATG

TGACGCTAGGGTCTTACCTTATTTATGTATTTTCTGAAATTCTGTATTCCATTGATATATCTTTAGCAATTGAGATATCTXXXXXXXXXXTCTGTTGGTCTGGTCATGCTTTTTGGCACATTATTCTTTATTTTTGGAAATCTATGTTGGATTTTATAGTAACTTTTATG

>Marker59882

AACAAGCTTCTGAAAGAAGACTATTTACCACTACTTTCACAGCCCATAATGAACATTACTGCATATGAAAGTCCACGTTCXXXXXXXXXXATAAGTCTTGAAGTGCTTTTTTCAACTTTCCGAACTTTTTGATAGGTTATAAGTTATGAACTTACACTTAAACATTCTTA

AACAAGCTTCTGAAAGAAGACTATTTACCACTACTTTCAGAGCCCATAATGAACATTACTGCATATGAAAGTCCACGTTCXXXXXXXXXXATAAGTCTTGAAGTGCTTTTTTCAACTTTCCGAACTTTTTGATAGGTTATAAGTTATGAATTTACACTTAAGCATTCTTA

>Marker60282

CAACAAGTGGAAATTGATTTTAATTGGGGAGGAAACGACAATGCAGGGGCATGAACACAGGACCTCCTCGGACCACCTGCXXXXXXXXXXGGCACCTCATAGTCCTGATAGAGGTCAGATAAAACCTTTTGTAGCCAAATTTCCTCACATATCCCCAAACACGTAGCTTC

CAACAAGTGGAAATTGATTTTAATTGGGGAGGAAACGACAATGCAGGGGCATGAACACAGGACCTCCCCGGACCACCTGCXXXXXXXXXXGGCACCTCATAGTCCTGATAGAGGTCAGATAAAACCTTTTGTAGCCAAATTTCCTCACATATCCCCAAACACGTAGCTTC

>Marker60325

CTACTTGTTAATTCTTCTATTTAATTTTTGCTTCAAAACTATTTGGTTTCTTGGTTTTGCTTAGAATAATGGTTGAGAAAXXXXXXXXXXGAACAAATTAATTGTTCATCTCCCTCTTCCTCAAATCTAGTGGATCACATGCATGTTACTAAGGAACGAAGGATGAGAAT

CTACTTTTTAATTCTTCTATTTAATTTTTGCTTCAAAACTATTTGGTTTCTTGGTTTTGCTTAGAATAATGGTTGAGAAAXXXXXXXXXXGAACAAATTAATTGTTCATCTCCCTCTTCCTCAAATCTAGTGGATCACATGCATGTTACTAAGGAACGAAGGATGAGAAT

>Marker60498

TCCATTCTTCGACAGTTTCTACTCCGACAACCTTCGTCAAATATTCTGGTTCCGCCTTACTCATCTTTTCCCTCTCGCCGXXXXXXXXXXCTGGAATGTAATTCTGTTTTCTAGGGCTTCTTCGCGAGGTTAACTTCCGATTGACTCCGATCGTCCGTCCCTTGAGGATA

TCCATTCTTCGACAGTTTCTACTCCGACAACCTTCGTCAAATATTCTGGTTCCGCCTTACTCATCTTTTCCCTCTCGCCGXXXXXXXXXXCTGGAAAGTAATTCTGTTTTCTAGGGCTTCTTCGCGAGGTTAATTTCCGATTGACTCCGATCGTCCGTCCCTTGAGGATA

>Marker60588

AACAATTGCGGGTGAGGGTTTGAATCTGGTAGGGTTTATCGAACCTGTAGAGTTAAACCAGCAATAGACACCAACACACGXXXXXXXXXXCCACTGACATCCCTAACTACCATGTTGTCTATCCTGAAATTCCTTTCTTGCCCCTCCGTCTATACCTATGGATGATTGGT

AACAATTGCGGGTGAGGGTTTGAATCTGGTAGGGTTTATCGAACCTGTAGAGATAAACCAGCAATAGACACCAACACACGXXXXXXXXXXCCACTGACATCCCTAACTACCATGTTGTCTATCCTGAAATTCCTTTCTTGCCCCTCCGTCTATACCTATGGATGATTGGT

>Marker60620

TTTGATTTTCTAGTAAAATAAAATAATTACCAAAAAAATAATTTAAATTTATTACTTTTTTAAAAAAAAAAAAGAAGAAGXXXXXXXXXXAATACAAAGAGAACTTGGCAAAACGTGTTTTCTAATTTCTTATGAAAAGTTAAAATAAAAGTTTGCAAGGGAGGGGCACA

TTTGATTTTCTAGTAAAATAAAATAATTACCAAAAAAATAATTTAAATTTATTACTTTTTAAAAAAAAAAAAAGAAGAAGXXXXXXXXXXAATACAAAGAGAACTTGGCAAAACGTGTTTTCTAATTTCTTATGAAAAGTTAAAATAAAAGTTTGCAAGGGAGGGGCACA

>Marker60734

AAGTGCTGGATGTAGAAGGATGACATATGGTGGTCCAGAATCAACATACTTCTCATTACTTTCAACCTGCCATAGGAAGGXXXXXXXXXXGTTTTTAGAGTATAAAACAATTTAAAAGGTCAATCTACAAAATTGAAAACATAAAAAAGACATCCAAACAGCTCCTTAGT

AAGTGCTGGATGTAGAAGGATGAGATATGGTGGTCCAGAATCAACATACTTCTCATTACTTTCAACCTGCCATAGGAAGGXXXXXXXXXXGTTTTTAGAGTATAAAACAATTTAAAAGGTCAATCTACAAAATTGAAAACATAAAAAAGACATCCAAACAGCTCCTTAGT

>Marker60952

TGGTTTTTTTGGGATAAGAAGGAACAAAGAATGATGTATTAAAGAAAGAAAAAGAGAATTGACCTAAAGACCTGAGGGATXXXXXXXXXXAAGGTGGCTAAATCATGATGTTTACCTATTGCTTGGTCTGAGATTGCTTCTTTCATATTACCTGCCTTGGGCTATTTCTT

TGGTTTTTTTGGGATAAGAAGGAACAAAGAATGATGTATTAAAGAAAGAAAAAGAGAATTGACCTAAAGACCTGAGGGATXXXXXXXXXXAAGGTGGCTAAATCATGATGTTTACCTATTGCTTGGTCTGAGATTGCTTCTTTCATATTACCTGCCCTGGGCTATTTCTT

>Marker61126

CTTGTTCTTCTCACACTCTGCTCAATGGAGATAAAATCAAGATAAACATGAGCATCACTGTATGAAAACAGAAAGTTGGAXXXXXXXXXXACCAAATCCATGCTTGTCATTACAGCCACCCGTCTATATTCCAATCCAAGAGACCAAAAAAGAAAAAGAGAAAATCAGCT

CTTGTTCTTCTCACACTCTGCTTAATGGAGATAGAATCAAGATAAACATGAGCATCACTGTATGAAAACAGAAAGTTGGAXXXXXXXXXXACCAAATCCATGCTTGTCATTACAGCCACCCGTCTATATTCCAATCCAAGAGACCAAAAAAGAAAAAGAGAAAATCAGCT

>Marker61140

CATCTTGAGGGTTTTTCTTCTTTCTCCTTGGCTTTCTTGAGGAAGCCATTTGAAAAAAAATTATTTAAAAAAAAAAAGATXXXXXXXXXXTGAATTTGAGACCAAAATGAGCCTTTTTGTGTTAGTTTGAGAGTTTTGGAGAGAGAAAATCGTGTGTGGACCAATGAATG

CATTTTGAGGGTTTTTCTTCTTTCTCCTTGGCTTTCTTGAGGAAGCCATTTGAAAAAAAATTATTTAAAAAAAAAAAGGAXXXXXXXXXXTGAATTTGAGACCAAAATGAGCCTTTTTGTGTTAATTTGAGAGTTTTGGAGAGAGAAAATCGTGTGTGGACCAATGAATG

>Marker61253

TTTTTTCAACGTTGAGAAAAGTTCTCCTGACGCCTTTTTTTCGACGTTCATATCGACGTCTAAATGCACGTTGACAAAACXXXXXXXXXXTGTGTTGCCATATCGACTAGAGGTGGACAAAGGGCATCAATTCTAGGACTCTTTCAGGAGCCCAGATCCACGGACTGGGT

TTTTTTCAACGTTGAGAAAAGTTCTCCTGATGCCTTTTTTTCGACGTTCATATCGACGTCTAAATGCACGTTGACAAAACXXXXXXXXXXTGTGTTGCCATATCGACTAGAGGTGGACAAAGGGCATCAATTCTAGGACTCTTTCAGGAGCCTAGATCCATGGACTGGGT

>Marker61299

TTGTGCATGCATTAAATGGGTTGAAGGATAAGAGAGAGTGATGCTTTGGTTTGACTGCTATATTTAATATGGATAATGAGXXXXXXXXXXGGATAACTTAAATTAATTACTCTTTTTCTCTCATACATAGATGACGATGTTTTATGGCTCATTTTTCAAGACAATCATTG

TTGTGCATGCATTAAATGGGTTGAAGGATAAGAGAGAGTGATGGTTTGGTTTGACTGCTATATTTAATATGGATAATGAGXXXXXXXXXXGGATAACTTAAATTAATTACTCTTTTTCTCTCATACATAGATGACGATGTTTTATGGCTCATTTTTCAAGACAATCATTG

>Marker61379

ATGTTGGTCTCAAAAAAGAAAAAGAAGACTTGCTACTTCCATCGCTTCTCTACCAAAGAGAGGTCATCGGAAACCTATAGXXXXXXXXXXTCAGCGAACCAACAAGTTAGAAGAACAGGCACGGGAAAACCTTCGCAATGCTTTTACGTTAATCTAACGGTTGAGTAAAG

ATGTTGGTCTCAAAAAAGAAAAATAAGACTTGCTACTTCCATCGCTTCTCTACTAAAGAGAGGTCATCGGAAACCTATAGXXXXXXXXXXTCAGCGAACCAACAAGTTAGAAGAACAGGCACGGGAAAACCTTGGCAATGCTTTTACGTTAATCTAACGGTTGAGTAAAG

>Marker61455

ACACTCTGGATTTCCTTATTTATTCCTCATTTTGTTTGTATTTACTCATGTAACCTTTCATTCTAAATCATTCATTCTCAXXXXXXXXXXTGATAAGGTCTACTCAAATTAATCTAATAAATGTTAGTAAAAATTGGGCAAAATAAATTGTTGGACTCAAACCTAGTCTT

ACACTCTGGATTTCCTTATTTATTCCTCATTTTGTTTGTATTTACTCATGTAACCTTTCATTCTAAATCATTCATTCTCAXXXXXXXXXXTGATAAGGTCTACTCAAATTAATCTAATTAATGTTAGTAAAAATTGGGCAAAATAAAATGTTGGACTCAAACCTAGTCTT

>Marker61516

CATCGTAAAGCACTAAAATCCAAACATGTGACAAAGGAAAAAATAACAAGAAAAGAAAAAAATCTAAAAACTTTTTCATGXXXXXXXXXXCAAAATGAATGGTAAAATTGATTTAGCCTATTTTTGGCAGTTAACGTATCTTCCCGAATGGAGCCTAATCCAAATTTTAT

CATCGTAAAGCACTAAAATCCAAACATGTGACAAAGGAAAAAATAACAAGAAAAGAAAAAAATCTAAAAACTTTTTCATGXXXXXXXXXXCAAAATGAATGGTAAAATTGATTTAGCCTATTTTTGGCAGTTAAGGTATCTTCCCGAATGGAGCCTAATCCAAATTTTAT

>Marker61531

TCTCTCTGTCTTGCATTTAAGAAAAAAGAAAATCTATCAAGAGAAACTTGTTTTAATTGAAACTAATCAGAAGTTGTGAGXXXXXXXXXXAAATGTATTAAATGACCCAAGTTTTTTTTATTATACAGCTAGACAGACCCACGAGGGGTCCTCTCTTTTAAACCATAACT

TCTCTCTGTCTTGCATTTAAGAAAAAAGAAAATCTATCAAGAGAAACTTGTTTTAATTGAAACTAATCAGAAGTTGTGGGXXXXXXXXXXAAATGTATTAAATGACCCAAGTTTTTTTTATTATACAGCTAGACAGACCCACGAGGGGTCCTCTCTTTTAAACCATAACT

>Marker62141

TTCATACAGTCTAGTTTAAATTAGGCAAAGTCATGCAAATAAACCATACATATAAGCATAAAGCATACCTAATGAGAGACXXXXXXXXXXAAAAAAAAACTCAAAGAAATACTTACAAATCATCAGTTCATGAATTCAGTTAAACCACAAAATTTTCTAAACAGTTACAA

TTCATACAGTCTAGTTTAACTTAGGCAAAGTCATGCAAATAAACCATAAATATAAGCATAAAGCATACCTAACGAGAGACXXXXXXXXXXAAAAAAAAACTCAAAGAAATACTTACAAATCATCAGTTCATGAATTCAGTTAAACCACAAAATTTTCTAAACAGTTACAA

>Marker62220

ATCAATATTTTCATGAAAGAAAGAAATAGGTCAATATTTATAGACATAGTAAATTCATAATCTTATGGCAACAATTATTTXXXXXXXXXXCATAAGCCATCCGGAAAGAAGAAATACAAACTAAAACCACACTAATTACAGTAGCATAGGCACCTCAATTCTTGTTAGAA

ATCAATATTTTCATGAAAGAAAGAAATAGGTCAATATTTATAGACATAGTAAATTCATAATCTTATGGCAACAATTATTTXXXXXXXXXXCATAAGCCATCCGGAAAGAAGAAATACAAACTAAAACCACACTAATTACGGTAGCATAGACACCTCAATTCTTGTTAGAA

>Marker62282

CAGTGCTGCCACGACGACTGCCAATTTCTTGGTCTCTACTCCACAACCAACCCGTGCATGTCTACATACTAACATTCTACXXXXXXXXXXAAAAATTTACCAAAACAATCAAATGTGGTCTCGGTTTGAGAGAACCTTTCCAATTCGGTATTTTTATTCTCGTAAGAAAA

CAGTGCTGCCACGACGACTGCCAATTTCTTGGTCTCTACTCCACAACCAACCCGTGCATGTCTACATACTAACATTCTACXXXXXXXXXXAAAAATTTACCAAAACAATCAAATGTGGTCTCGGTTTGAGAGAACCTTTCCAATTCGGTATTTTTATTCTCGCAAGAAAA

>Marker62671

TATGAATTTAAACCCCCAACTTTGTTCTGTTAGACCTTTTATCAATTAGGGTTCCCCGTTTTGCCTCCCACTATATTTTCXXXXXXXXXXTATAGAGCAGTTAGATAAGGCTGAAGGGCTTGCAATCATGGAATCAATGAAAGCGGCTTATTGTGCTGTGGCAGTGGAAT

TATGAATTTAAACCCCCAACTTTGTTCTGTTAGACCTTTTATCAATTAGGGTTCCCCGTTTTGCCTCCCACTGTATTTTCXXXXXXXXXXTATAGAGCAGTTAGATAAGGCTGAAGGGCTTGCAATCATGGAATCAATGAAAGCGGCTTATTGTGCTGTGGCAGTGGAAT

>Marker62921

TTTCTCTCTCTCGTATTCCGAAGATTAGAATGTATTTTAATTTTGCAACATTATGGAAAAAGTCATTGTTCCAAAAAAGAXXXXXXXXXXTGTTTTTCCAAAATATCTACCTATAATTTCGATACATATATATTAATTACAAATTAATTAATTGAGTTATGATCGATATG

TTTCTCTCTCTCGTATTCCGAAAATTAGAATGTATTTTAATTTTGCAACATTATGGAAAAAGTCATTGTTCCAAAAAAGAXXXXXXXXXXTGTTTTTCCAAAATATCTACCTATAATTTCGATACATATATATTAATTACAAATTAATTAATTGAGTTATGATCGATATG

>Marker63130

ACGACACTTTCTGACGATATTCTCCCTGTCAAATGTAAATTCTACAATTAGTATAGTGCATGATTGACAGATGTGGAGCAXXXXXXXXXXGGTCAATCGGTGAATAGAGATGCGAGTTTCCATGTTCAATACCTGTCCTCATATATTCTGGTGGGGTGAAAGACAGGTTA

ACGACACTTTCTGGCGATATTCTCCCTGTCAAATGTAAATTCTACAATTAGTATAGTGCATGATTGACAGATGTGGAGCAXXXXXXXXXXGGTCAATCGGTGAATAGAGATGCGAGTTTCCATGTTCAATACCTGTCCTCATATATTCTGGTGGGGTGAAAGACAGGTTA

>Marker63246

TTTGTCACATCCCTTCTCAAATCACAATTGATATTTAAAGTAAACATTTATCAAATAATGTATAAATTCTAGAATAATTAXXXXXXXXXXCACACGTACGTGCAATTATTCTTCTAAACGATCATAGTATGCGATTATGTAGAAAATGATACATGATTGTTCGGATTTTG

TTTGTCACATCCCTTCTCAAATCACAATTGATATTTAAAGTAAACATTTATCAAATAATGTATAAATTCTAGAATAATTAXXXXXXXXXXCACACGTACGTGTAATTATTCTTCTAAACGATCATAGTATGCGATTATGTAGAAAATGATACATGATTGTTCGGATTTTG

>Marker63340

ATTTCCTAACGTGTAAAATTAACAAATAGTTATTTTAAAAAAGAAACCAACATATAAAAGAAAAATCTGGACATTTGGTTXXXXXXXXXXTTAAGGAGAACTGGATTTTGGTAAATTCCATCGATCTACATCCCTAGGATTTAGACAAAATAGCATATAAAATGCAGCCA

ATTTCCTAACGTGTAAAATTAACAAATAGTTATTTTAAAAAAGAAACCAACATATAAAAGAAAAATCTGGACATTTGGTTXXXXXXXXXXTTAAGGAGAACTGGATTTAGGTAAATTCCATCGATCTACATCCCTAGGATTTAGACAAAATAGCATATAAAATGCAGCCA

>Marker63491

ATTAATCAATTTTACCTATATAGAAATATTAACTTTTTGTTTCATTTATTCTTCCTTTCCTTCGAATTTAATTCGTTTCTXXXXXXXXXXTTTTTGGTCCCTAGACTTTGCATACTATCTTTTCTTTATCGCTGTTTAATTTCTATGATGTAAAACACCTCTCATATGTA

ATTAATCAATTTTACCTATATAGAAATATTAACTTTTTGTTTCATTTATTCTTCCTTTCCTTCGAATTTAATTCGTTTCTXXXXXXXXXXTTTTTGGTCCCTAGACTTTGCATACTATCTTTTCTTTATCGTTGTTTAATTTCTATGATGTAAAACACCTCTCATATGTA

>Marker63764

AATAAAGTTTGTTCAATTTCCTCCTTCAATATAGGATGGAAAATTAAACAATAATAATGACTAAAATTTCCTGGGATAACXXXXXXXXXXAATTAAGTAGACGAATGCTGTAACCATATTGCATATTTCCTTAGTTGCCTGGAATCTAATTTTTCTTGTCTGGTCAGGCA

AATAAAGTTTGTTCAATTTCCCCCTTCAATATAGGATGGAAAATTAAACAATAATAATGACTAAAATTTCCTGGGATAACXXXXXXXXXXAATTAAGTAGACGAATGCTGTAACCATATTGCATATTTCCTTAGTTGCCTGGAATCTAATTTTTCTTGTCTGGTCAGGCA

>Marker63772

CCAAAAATTTGTCGAATGAAGTTAGAAAATCTTCTATGGGAACACATATTCTTCTAGAGTTATATTATACTCTACTTACAXXXXXXXXXXGGTTGCATCACAAAGGAAGAAGTGGCATCAATGCTTAGCGTAATAATATTGCTCCTTCCTTGATTTTAATTCCATTCTTG

CCAAAAATTTGTCGAATGAAGTTAGAAAATCTTCTATGGGAACACATATTCTTCTAGAGTTATATTATACTCTATTTACAXXXXXXXXXXGGTTGCATCACAAAGGAAGAAGTGGCATCAATGCTTAGCGTAATAATATTGCTCCTTCCTTGATTTTAATTCCATTCTTG

>Marker64295

ATATCTTCAACTTTGGAAGAATTTTACTCAATCAAACATTAATAAATGCACATTTAATAGACACATCAACTCAACCTTTTXXXXXXXXXXGAATGTAATTAGGAAAACAAATTAAAACTAATCAAATCTTTTTCACTTCATATTTGTTATCATGGCGGATCTATTAAAGA

ATATCTTCAACTTTGGAAGAATTTTACTCAATCAAACATTAATAAATGCACATTTAATAGACACATCAACTCAACCTTTTXXXXXXXXXXGAATGTAATTAGGAAAACAAATTAAAACTAATCAAATCTTTTTCACTTCATATTTGTTATCATGGCGGATCTATTAAAGG

>Marker64322

CCTTAATAAATCTGCCTTTGAGTGGGTTTAAGGCTTTGCAATGTTATAATTTCCTCCATATTTAACATTGTATGGATAATXXXXXXXXXXAAATTTATTTTCAATAAAGTGGAGTTTCATCGATTATATTAAATTTATGTTCACTTATTAGTTAAGTTTTTGGATTTGAT

TCTTAATAAATCTGCCTTTGGGTGGGTTTAAGGCTTTGCAATGTTATAATTTCCTCCATATTTAACATTGTATGGATAATXXXXXXXXXXAAATTTATTTTCAATAAAGTGGAGTTTCATCGATTATATTAAATTTATGTTCACTTATTAGTTAAGTTTTTGGATTTGAT

>Marker64828

AATCCAAAATAAAAGACAATATACACCTGAGATGGAAAGCAAATGGTGAAATGGTTGTTCCAACCGATGAGTTGAGTATTXXXXXXXXXXTTTAATATTTAATGTAGCAGCAGAATGCAGAGAGAGAGAATTTGGGTCAATTCTTGCAGAGTTCCATAGCCCACAGGAAA

AATCCAAAATAAAAGACAATATACACCTGAGATGGAAAGCAAATGGTGAAATGGTTGTTCCAACCGATGAGTTGAGTATTXXXXXXXXXXTTTAATATTTAATGTAGCAGCAGAAAGCAGAGAGAGAGAATTTGGGTCAATTCTTGCAGAGTTCCATAGCCCACAGGAAA

>Marker65023

ATATTAAAGATGTAAATCAATCTATGCATTAAAGATGTGAATGAATCTGAATTTCTTGATTCATTATGACTTTTGGGTGTXXXXXXXXXXGAGTCATAAGGAGGTCACCAGGAAGGCAAAGACTAAAGTTTGCCAACAGTTGTGTTTCTAATCATATTCAATGAAATTGT

ATATTAAAGATGTAAATCAATCTATGCATTAAAGATGTGAATGAATCTGAATTTCTAGATTCATTATGACTTTTGGGTGTXXXXXXXXXXGAGTCATAAGGAGGTCACCAGGAAGGCAAAGACTAAAGTTTGCCAACAGTTGTGTTTCTAATCATATTCAATGAAATTGT

>Marker65125

TTTTTCAGTGTAATGGTTCACCTTGCAGTTCACCTACCATATGAGACTAAGATTGTTAGTCCAGTTTCTTATAGTTGGATXXXXXXXXXXTCCCATTTTTGAAGCATATCACAAGAAGAGCAACAACTCTTCCATTGATACATTTTCAACAATGTCGATGAAATAATGGA

TTTTTCAGTGTAATGGTTCACCTTGCAGTTCACCTACCATATGAGAATAAGATTGTTAGTCCAGTTTCTTATAGTTGGATXXXXXXXXXXTTCCATTTTTGAAGCATATCACAAGAAGAGCAACAACTCTTCCATTGATACATTTTCAACAATGTCGATGAAATAATGGA

>Marker65752

CATTTATGGTCATTTTACTGTCAAATAGCTTCACTTGAGAACTCATGGCTTACTTAGAAGATGAATCGGTCTTCTTTTCTXXXXXXXXXXCATCAATTCACTTTGAGTTCTAAAATTACTTATTGATAATTGCCTCATGCAGCTATCACGGTTGAAAGTTAGAACTCGAA

CATTTATGGTCATTTTACTGTCAAATAGCTTCACTTGAGAACTCATGGCTAACTTAGAAGATGAATCCGTCTTCTTTTCTXXXXXXXXXXCATCAATTCACTTTGAGTTCTAAAATTACTTATTGATAATTGCCTCATGCAGCTATCACGGTTGAAAGTTAGAACTCGAA

>Marker65985

ATTGACGGGGAAACACAATTTGTTGAATTAAACTTCCTAGCGGTGTCGGGGGATACAAAGGTGGCATTGTGTTCAATATTXXXXXXXXXXGGGGATTTCCTAGCAATTAAATTGGGTAAAATGTATGTTATTCTAGGGATGTAGTGTTGCTCACTACCGAATTTATGAGG

ATTGACGGGGAAACACAATTTGTTGAATTAAACTTCCTAGCGGTGTCGAGGGATACAAAGGTGGCATTGTGTTCAATATTXXXXXXXXXXGGGGATTTCCTAGCAATTAAATTGGGTAAAATGTATGTTATTCTGGGGATGTAGTGTTGCTCACTACCGAATTTATGAGG

>Marker66115

CCAAGTTTCGGTCAGTTCCTTGAAACCATTTAACCAAATGCTGCTTGTGCCTTGATTGACATATATATATGAACTAAAAGXXXXXXXXXXTGAGTATTTGTAAAGAGAATCAATCATTGAATGACATATATTCTGATGGATAGAGCTCAAATTGAAGGTGTTTTGTTATG

CCAAGTTTCGGTCAGTTCCTTGAAACCATTTAACCAAATGCTGCTTGTGCCTTGATTGACATATATATATGAACTAAAAGXXXXXXXXXXTGAGTATTTGTAAAGAGAATCAATCATTGAATGACATACATTCTGATGGATAGAGCTCAAATTGAAGGTGTTTTGTTATG

>Marker66317

CATTTTACACCTTTGGGATGATTTTCTTCTTTGGTAAATTAAATAAAAATTTTAAAGGAAAGACCTAATTTTGCTATCATXXXXXXXXXXAGACAGCTATTGGAGGGCTACTAGAGAGTTGTTGACTTTTAAATTTGTTAATTTTGCAATTTAGAAAATGTAGTGACATG

CATTTTACACCTTTGGGATGATTGTCTTCTTTGGTAAATTAAATAAAAATTTTAAAGGAAAGACCTAATTTTGCTATCATXXXXXXXXXXAGACAACTATTGGAGGGCTACCAGAGAGTTGTTGATTTTTAAATTTGTTAATTTTGCAATTTAGTAAATGTAGTGACATG

>Marker66368

TTGGATAATTGAGAATGAAAGATTTGAAAGAAAAGGTTCTCTATCATGTTTTAATGAGGTTCTTGAAATTCTAAACATTTXXXXXXXXXXATATTATTCTGTGATTCCAGATTTTGCAGGTGGTTCACGTCTGCAGTTAATTGAGAAACCAAGTAGAATTTATGTGTCTA

TTGGATAATTGAGAATGAAAGATTTGAAAGAAAAGGTTCTCTATCATGTTTTAATGAGGTTCTTGAAATTCTAAACATTTXXXXXXXXXXATATTATTCTGTGATTCCAGATTTTGCAGGTGGTTCACGTCTGCAGTTAAATGAGAAACCAAGTAGAATTTATGTGTCTA

>Marker66510

TCAAACTACTCCCCCATCCCAAAACGAAAGATTCCAGTTAAATTAGACTATGGCTAAAGTAAATCAAACTTACATCTGATXXXXXXXXXXATAAACAAATTTAGTGTGTTTAGTGAATCAGTGAAAACCAAACTTCCATCAGAAAGTCCATTATGAAGAACTAAAACACA

TCAAACTACTCCCCCATCCCAAAATGAAAGATTCCAGTTAAATTAGACTATGGCTAAAGTAAATCAAACTTACATCTGATXXXXXXXXXXATAAACAAATTTAGTGTGTTTAGTGAATCAGTGAAAACCAAACTTCCATCAGAAAGTCCATTATGAAGAACTAAAACACA

>Marker66550

ACCAACTGTCGGACTTGTATTCAATTTGTTCAAAGTTTTGATTTGTGTGTATTGTATCTGACGCATATTAAACACAAACAXXXXXXXXXXTTGACAATAAGACATGCTATTATGATTGAATGTGATATCTGATTGCAATGGTTTAATCTAATAGAGGTTCTTACTGAGAT

ACCAACTGTCGGACTTGTATTCAATTTGTTCAAAGTTTTGATTTGTGTGTATTGTGTCTGACGCATATTAAACACAAACAXXXXXXXXXXTTGACAATAAGACATGCTATTATGATTGAATGTGATATCTGATTGCAATGGTTTAATCTAATAGAGGTTCTTACTGAGAT

>Marker66730

CTTGCAATGCCAAGACCAACATCTTCTTGACAATTTCAACAGAAGAATCTTCCACTAATGTGAGCAAATTCTTGCCAATGXXXXXXXXXXAGTATAAGTTGCAAAGAATGAATTGCATCCATTTCAAAGTCCTTCCATGGCAAACTCCTTGTCTTAACCACTTCAAGAAA

CTTGCAATGCCAAGACCAACATCTTCTTGACAATTTCAACAGAAGAATCTTCCACTAATTTGAGCAAATTCTTGCCAATGXXXXXXXXXXAGTATAAGTTGCAAAGAATGAATTGCATCCATTTCAAAGTCCTTCCATGGCAAACTCCTTGTCTTAACCACTTCAAGAAA

>Marker66815

GACGTATTCCACATTCTATCGGGGATGTGGAAAACCCCTGCTGAGAGGTGTTTCATGTGGCTGGGAGGATTTCGTTCGTCXXXXXXXXXXACAAGCTGAAGATGCGTTGTCACAAGGCATTGAAGCTTTGCAACAATCTCTTGTAGAAACTCTTTCCTCTGCTTCTTTAG

GACATATTCCACATTCTATCGGGGATGTGGAAAACCCCTGCTGAGAGATGTTTCATGTGGCTGGGAGGATTTCGTTCGTCXXXXXXXXXXACAAGCTGAAGATGCGTTGTCACAAGGCATTGAAGCTTTGCAACAATCTCTTGTAGAAACTCTTTCCTCTGCTTCTTTAG

>Marker67002

ACAAACTGAGAGCTAGAAGTTTGTATCAGGGATAAGACAGTTAATGAACAACCTATCGTGGGGCAATTAATACTCATACTXXXXXXXXXXTATAGGACTTAACATTCGAGAAGCACATGTATTTTCAACAACTGATGGTTACTCCTTGGATGTATTTGTGGTGGATGGAT

ACAAACTGAGAGCTAGAAGTTTGTATCAGGGATAAGACAGTTAATGAACAACCTATCATGGGGCAATTAATACTCATACTXXXXXXXXXXTATAGGACTTAACATTCGAGAAGCACATGTATTTTCAACAACTGATGGTTACTCCTTGGATGTATTTGTGGTGGATGGAT

>Marker67025

AAACCAGGGATTGGAATAGCATTGTAACGTAATTGACAAAAATGTGATAATTCTGACACACATAAGACAACCAAATCAAAXXXXXXXXXXAGCCGTTTTCCTTGTTTAATCGTAAGATTAGCTCCTGCCAAATGAATATGAAACGGGAAATAACAATAAAGGCTTGTTTA

AAAGCAGGGATTGGAATAGCATTGTAACGTAATTGACAAAAATGTGATAATTCTGACACACATAAGACAACCAAATCAAAXXXXXXXXXXAGCCGTTTTCCTTGTTCAATCGTAAGATTAGCTCCTGCCAAATGAATATGAAACGGGAAATAACAATAAAGGCTTGTTTA

>Marker67038

TTTGCTCAGATTCTTCTTGAAAATGACTCGTCTTCCTTGAACCAAAACGCAAACCTTCTCGCATCCAACTTGAATAACAAXXXXXXXXXXCCTCTTAAGCTCCTAATTACACCTCCTATTTCTAATCCACCTCATAAAATGCATGAAAAGTAATTAAGAACCTTGGATTT

TTTGCTCAGATTCTTCTTGAAAATGACTCGCCTTCCTTGAACCAAAACGCAAACCTTCTCGCATCCAACTTGAACAACAAXXXXXXXXXXCCTCTTAAGCTCCTAATTACACCTCCTATTTCTAATCCACCTCATAAAATGCATGAAAAGTAATTAAGAACCTTGGATTT

>Marker67410

CCACGTTTTTCTGCCAACTAAAATCCAAACGACAGTCACCACTCACGAGTCTGAACCGACATCCCAACACATAACTATGCXXXXXXXXXXCTTTACTACGCTTCAATGCAGATGTCTTTAGGGTTTGGTCACGCTTTATTGGAAACGAGCCCACGATCAAGTCATATGTG

CCACGTTTTTCTGCCAACTAAAATCCAAACGACAGTCACCACTCACGAGTCTGAACCGACATCCCAACACATAACTATGCXXXXXXXXXXCTTTACTACGCTTCAATGCAAATGTCTTTAGGGTTTGGTCACGCTTTATTGGAAACGAGCCCACGATGAAGTCATATGTG

>Marker67414

TATTATTTTGACTCAAGGTTCTCAGAGTTTGGCTCCGAGTTCTTTCCACATTGAGAATATGTGGCTTTGGCATCTTTCCTXXXXXXXXXXTTTGGTCCTCAGTAAGATTTCATATTTTCTCTTTGGATTTGCTTTCGAAGGCTTTTTGAAATTACTATCTAGGCAACATT

TATTATTTTGACTCAAGGTTCTCAGAGTTTGGCTCCGAGTTCTTTCCACATTGAGAATATGTGGCTTTGGCATCTTTCCTXXXXXXXXXXTTTGGTCCTCAGTAAGATTTCATATTTTCTCTTTGGATTTGCTTTCGAAGGCTTTTTGAAATTACTCTCTAGGCAACATT

>Marker67672

TAAAATGGAACAGAACAGACTTGAAACTTCCAAAGACTAAAATATATTCTTAAAAGCTGCCTGGAAATGAGATATTTCGAXXXXXXXXXXAACTTGGTTAATATTTGGCGTGTGGAGAGGTTGAATTCGAGTATGTCGACATGTTCTCAGAATTCAGGTTCGGTGTGTAT

TAAAATGGAACAGAACAGACTTGAAACTTCCAAAGACTAAAATATATTCTTAAAAGCTGCCTGGAAATGAGATATTTCGAXXXXXXXXXXAACTTGGTTAATATTTGGCGTGTGGAGAGGTTGAATTTGAGTATGTCGACATGTTCTCAGAATTCAGGTTCGGTGTGTAT

>Marker67814

CCGATATTCTTATTTTATTGATATAACCCAAAAGAAAACATTATAATAAAGCTAATTATAGTCGTTAATACTTGCTAAGCXXXXXXXXXXTCAAAAAAAAAAAAAGTAAACACTGTAGCAAAGAACAAAAATGATAAATGTAAAATAATAAATACTATAGCAAATAGTAA

CCGATATTCTTATTTTATTGATATAACCCAAAAGAAAACATTATAATAAAGCTAATTATAGTCGTTAATACTTGCTAAGCXXXXXXXXXXCTTCAAAAAAAAAAAGTAAACACTGTAGCAAAGAATAAAAATGATAAATGTAAAATAATAAATACTATAGCAAATAGTAA

>Marker68084

TAAAAGGGGGATGATTCTCTTATCCGGTCCTCATTGATTAATAGTATGGAACCACAGACCGACAAGCCCTTGCTGTATGCXXXXXXXXXXGGATGTAGGAGGTTGACAAGATTTATGCTTTCCTTACTGGTCTCAACCCTAAGTTTGATATTGTCCGCGGGCGTATACTG

TAAAAGGGGGATGATTCTCTTATCCGGTCCTCATTGATTAATAGTATGGAACCACAGACCGACAAGCCCTTGCTGTATGCXXXXXXXXXXGGATGTAGGAGGTTGACAAGATTTATGCTTTCCTTACTGGTCTCAACCCTAAGTTTGATATTGTCCGCGGGCATATACTG

>Marker68212

TAAGATTTTCAATGAGAATGCCTCCCATATAGTATAATTTTATAATTGAAAAAATGGGGACATCAAAATTCAGAACCACAXXXXXXXXXXTAATTTTTCTTTTATCTCTCTTCATCTTTCGTCCTAATTCGCCAATGGCGGACCTTCCATCGCCGTCACGAAAAAACAGA

TAAGATTTTAAATGAGAATGCCTCCCATATAGTATAATTTTATAATTGAAAAAATGGGGACATCAAAATTCAGAACCACAXXXXXXXXXXTAATTTTTCTTTTATCTCTCTTCATCTTTCGTCCTAATTCGCCAATGGCGGACCTTCCATCGCCGTCACGAAAAAACAGA

>Marker68623

CCACATTGCATACTTGCAAATGCAATGTGCAACTTTTCGTTTCTCTTTCTATCTATGTATTTTCTAAGGTTTATCTCTACXXXXXXXXXXCTAACAAGTGATTGTTCAGCCCAAGAACAGTCCAACCTTAGGCTTCAATCAATAGCAGTCCCAATCCTCATACACCAACA

CCACATTGCATACTTGCAAATGCAATGTGCAACTTTTTGTTTCTCTTTCTATCTATGTATTTTCTAAGGTTTATCTCTACXXXXXXXXXXCTAACAAGTGATTGTTCAGCCCAAGAACAGTCCAACCTTAGGCTTCAATCAATAGCAGTCCCAATCCTCATACACCAACA

>Marker68752

AAGACCCTTCCTCGTCAGTGCAAAAGAAAGTCCTTACTAGTTAGCAATAAGAGTTTGAACCAAGACCTTAGAAATGATTTXXXXXXXXXXCCTCCCAATATCTTTCAAGTAGAAAGGAGATTCCAAGACTTATTGATCCAAACCACTTTTGCGCGAATCGCTATTGAAAA

AAGACCCTTCCTCGTCAGTGCAAAAGAAAGTCCTTACTAGTTAGCAATAAGAGTTTGAACCAAGACCTTACAAATGATTTXXXXXXXXXXCCTCCCAATATCTTTCAAGTAGAAAGGAGATTCCAAGACTTATTGATCCAAACCACTTTTGCGCGAATCGCTATTGAAAA

>Marker68853

ATTTGCAATATCATAATCAACAGAAGCATATGATGCTGGTCCCAAGACCTTGAACCAAATTAAACAGTCTTATTTCTCAGXXXXXXXXXXAAGATGTCCTTATTCCCTGGTATTAGCAGAAATAAGCAAGCATTCAGTAAAAATTCTTACATTGAAGTAGCATTTTCTTT

ATTTGCAATATCATAATCAACAGAAGCATATGATGCTGGTCCCAAGACCTTGAACCAAATTAAACAGTCTTATTTCTCAGXXXXXXXXXXAAGATGTCCTTATTCCCTGGTATTAGCAGAAATAAGCAAGCATTCAGTAAGAATTCTTACATTGAAGTAGCATTTTCTTT

>Marker68889

AAGAGTTTTGAGCTGTTGAACGTGCTCTGCAGGAAGTGGTGGAGGCAACCAGTCAGATCCTGTCATCTCCCCATCTACATXXXXXXXXXXTTTGGTTTTAGTGTCCCCCACTCCTGTTATCTTTCTCATCCCAAAGACCAATAAGACAGCCCTCGTGAATGGATGTGCTC

AAGAGTTTTCAGCTGTTGAACGTGCTCTGCAGGAAGTGGTGGAGGCAACCAGTCAGATCCTGTCATCTCCCCATCTACATXXXXXXXXXXTTTGGTTTTAGTGTCCCCCACTCCTGTTATCTTTCTCATCCCAAAGACCAATAAGACAGCCCTCGTGAATGGATGTGCTC

>Marker68980

TTCCCTAACTATGACCTTTTGGGTAGGAGCAAAACAAATTGTCCTCAAGATTGATCCCTCTCTCATTAGAGCTGAATGTTXXXXXXXXXXGAGACCATACAAATATGGGCATGTCCAAAAAGAAGAAATTGAGAAACTGGTGGCAGATATGCTACAAACAGGTGTGATTA

TTCCCTAACTATGACCTTTTGGGTAGGAGCAAAACAAATTGTCCTCAAGAGTGATCCCTCTCTCATTAGAGCTGAATGTTXXXXXXXXXXGAGACCATACAAATATGGGCATGTCCAAAAAGAAGAAATTGAGAAAATGGTGGCAGAGATGCTACAAACAGGTGTGATTA

>Marker68994

TCTTTGAGATAGATCTTCTACATACATGTGGTAGGACACTAGACTTGTTAAATGATATTTAGAAGCCTTATTTATACAGCXXXXXXXXXXAAAAAAAAAAGAAACTCAAATGTATAAGAATCCACGACAGGAATCTCAAGAATTCTGATGGAGAGTTTCACTGAAAAGGA

TATTTGAGATAGATCTTCTACATACATGTGGTAGGACACTAGACTTGTTAAATGATATTTAGAAGCCTTATTTATACAGCXXXXXXXXXXAAAAAAAAAAGAAACTCAAATGTATAAGAATCCACGACAGGAATCTCAAGAATTCTGATGGAGAGTTTCACTGAAAAGGA

>Marker69189

CAATAGCTAGATCAAAACTTATTTCAACATGATCACACCAATAGCCACCCACAAGAACATTATAAGAGATTTTTTTTTAAXXXXXXXXXXAAACGCGGGGAGTAGAGAATGAATGGGTCGGCGTGGATTGGCGGCAATTAGAATTAGGAGGCAAAGGAGAAAGGGATTGG

CAATAGCTAGATCAAAACTTATTTCAACATGATCACACCAATAGCCACCCACAAGAACATTATAAGAGATTTTTTTTAAAXXXXXXXXXXAAACGCGGGGAGTAGAGAATGAATGGGTCGGCGTGGATTGGCGGCAATTAGAATTAGGAGGCAAAGGAGAAAGGGATTGG

>Marker69605

CACTATTGACCAACTTGAATATCATATTCAAACACACAAATTTACCGTAAGTGAAAATACGCTCCTTACAGCTACACCTTXXXXXXXXXXAAAGAAACAAAATTTACATTGCTAAGTTGTTTTTTGTTGTTGTTGCTTTGTTGGTCTCATAGAAAAGGTTGAAAGCTTGG

CACTATTGACCAACTTGAATATCATATTCAGACACACAAATTTACCGTAAGTGAAAATACGCTCCTTACAGCTACACCTTXXXXXXXXXXAAAGAAACAAAATTTACATTGCTAAGTTGTTTTTTGTTGTTGTTGCTTTGTTGGTCTCATAGAAAAGGTTGAAAGCTTGG

>Marker69674

CTTTTCTCAACGCAATGGGAATGTCTAGAGAAGGTTCATACTCATAAAGATTTCCTGAACGACCTGTTCAACCTCATTCCXXXXXXXXXXGAACTTAGTTTGTGGCTAGGACCATGGAATGAGGATCAGAGTCAGGTAAGGTAATAGGAGTAGGATTGGTAGACTCTAAG

CTTTTCTCAACGCAATGGGAATGTCTAGAGAAGGTTCATACTCATAAAGATTTCCTGAACAACCTGTTCAACCTCATTCCXXXXXXXXXXGAACTTAGTTTGTGGCTAGGACCATGGAATGAGGATCAGAGTCAGGTAAGGTAATAGGAGTAGGATTGGTAGACTCTAAG

>Marker69735

TTGCCCGACAGTTTCGTTGTGGACATGTTTATGTCTTAACCGTCAATACGCCAACTTAGGCTTTGATACCATTTGTCACAXXXXXXXXXXTGTCAATTGCAAAGAAAGCTTTAATTGCAAAGAGTAAGACAAGGTTTGGTCCAGGATTCAACTATTGAGTCTCCCCTATA

TTGCCCGACAGTTTCGTTGTGGACATGTTTATGTCTTAACCGTCAATACGCCAACTTAGGCTTTGATACCATTTGTCACAXXXXXXXXXXTGTCAATTGCAAAGAAAACTTTAATTGCAAAGAGTAAGACAATGTTTGGTCCAGGATTCAACTATTGAGTCTCCCCTATA

>Marker69781

AAATGGCTGCATCTGATTGGGTGTTGCAAGATGATAAGTCGGAGAGCTTGTGGAATTTCTAAAAGAGTAAATTAAGACTAXXXXXXXXXXTATATATGATATGTTATGTAAAATGTATACTGTTTCTTTAGTTCTAACCACATTCTTGACGATTTCTAGCACAATTGTTT

AAATGGCTGCATCTGATTGGGTGTTGCAAGATGATAAGTCGGAGAGCTTGTGGAATTTCTAAAAGAGTAAATTAAGACTAXXXXXXXXXXTATATATGATATGTTATGTAAAATGTATACTGTTTCTTTAGTTCTAATCACATTCTTGACGATTTCTAGCACAATTGTTT

>Marker70014

AATCAAAATGCACGACGAATATCACTTTGGGCATTTTAGTAATTTGGGTCAGTTTCGGAAGGGCGACGTGGCGTGTTTTGXXXXXXXXXXTTGGACCAATTATGCGATATTATTTAATTTGGTTATTTGTGATTGGAGTTAACTGCACGAAAATAAGTTCAATTCAGGGT

AATCAAAATGCACGACGAATATCACTTTGGGCATTTTAGTAATTTGGGTCAGTTTCGGAAGGGCGACGTGGCGTGTTTTGXXXXXXXXXXTTGGACCAATTATGCGATATTATTTAATTTGGTTATTTGTGATTGGAGTTGACTGCACGAAAATAAGTTCAATTCAGGGT

>Marker70099

TCTATGATCGACCTGGATATGGTGAAAGTGATCCAAATCCCAATAGCTCAGTTAAAAGTGAGGCTTATGACATTCAAGAAXXXXXXXXXXTTATTGCTTTCTTTCACCAAAAATGATTGAATGCAGGTTAGCAGGCACAGCTCTTATAGTTCCTTTAGTGAATTACCAAT

TCTATGATCGACCTGGATATGGTGAAAGTGATCCAAATCCCAATAGCTCAGTTAAAAGTGAAGCTTATGACATTCAAGAAXXXXXXXXXXTTATTGCTTTCTTTCACCAAAAATGATTGAATGCAGGTTAGCAGGCACAGCTCTTATAGTTCCTTTAGTGAATTACCAAT

>Marker70624

TAATTTGACATCCATTTGAAAAGTGAAGAAGGTGAAAAGCATGCATTGTTTTCTTCATTTTATAATATCTGTATTGAACCXXXXXXXXXXTTTCACCCAAAGATACACATAATTTAAATAATTTAGGTGTGAAACCACTTCAAGAATATTGGTCGGTTATACCCTGTTGA

TAATTTGACATCCATTTGAAAAGTGAAGAAGGTGAAAAGCATGCATTGTTTTCTTCATTTTATAATATCTGTATTGAACCXXXXXXXXXXTTTCACCCAAAGATACAAATAATTTAAATAATTTAGGTGTGAAACCACTTCAAGAATATTGGTCGGTTATACCCTGTTGA

>Marker70684

TTTTGACAATGTTTTATTTCTTATTTTTCATTTCCTGCTTCCTCTTTATCAAGAAAGAATAACGTTCCAGATTTCTTTGTXXXXXXXXXXAATTTTTACCTTAGTTTTCATAAACTTAAGTAAAAGAATTTGATTGCTAGCAAATTCCAAAAACAAAAATAGTTTTTTAT

TTTTGACAATGTTTTGTTTCTTATTTTTCATTTCCTGCTTCCTCTTTATCAAGAAAGAATAACGTTCCAGATTTCTTTGTXXXXXXXXXXAATTTTTACCATAGTTTTCATAAACTTAAGTAAAAGAATTTGATTGCTAGCAAATTCCAAAAACAAAAATAGTTTTTTAT

>Marker70689

ATTCATCTTCAAGGCACAAACAAGTTTCGCCAACCTCTTAAACTTGTAATTCATCATAGAAATGCTCCAGAAAGAAACTAXXXXXXXXXXATGATCAGGGGAATTGACAATTGCGGGCAACACAGCAGGACGCTAGAATTTATTGTTATTTGATTTCAGTATAAATAAAG

ATTCATCTTCAAGGCACAAACAAGTTTCGCCAACCTCTTAAACTTGTAATTCATCATAGAAATGCTCCAGAAAGAAACTAXXXXXXXXXXATGATCAGGGGAATTGACAATTGCGGGCAACACAGCAGAACGCTAGAATTTATTGTTATTTGATTTCAGTATAAATAAAG

>Marker71546

TCTGCCAGGATAAGGCTGAGGATCACTCCATAGCATATCGCTCATTAATCCTGAAGAGAAAATGGACAGGGACCTAGAAAXXXXXXXXXXAACTTTACCTTTTTCTGGAGGTTCACAAAAACGGTTTATCATTCTAATGTCAGAGAGTTTTACCCCATCAACGCTGAAAA

TCTTCCAGGATAAGGCTGAGGATCACTCCATAGCATATCGCTCATTAATCCTGAAGAGAAAATGGACAGGGACCTAGAAAXXXXXXXXXXAACTTTACCTTTTTCTGGAGGTTCACAAAAACGGTTTATCATTCTAATGTCAGAGAGTTTTACCCCATCAACGCTGAAAA

>Marker71609

CAACATTGTAGATCTCACTTATGTCTCTAGAAGTTGTAATAAGGTTGCTTGCAGGCAATTCGTGGACTTCTTGTTCGTGGXXXXXXXXXXTCCCCAAATGAAAGCCTTATCCCTTTTAAGTAACTGATGGAAACACATTAATGCATTATATTTCATTTAAACTCATATCA

CAATATTGTAGATCTCACTTATGTCTCTAGAAGTTGTAATAAGGTTGCTTGCAGGCAATTCGTGGACTTCTTGTTCGTGGXXXXXXXXXXTCCCCAAATGAAAGCCTTATCCCTTTTAAGTAACTGATGGAAACACATTAATGCATTATATTTCATTTAAACTCATATCA

>Marker71913

CCATTTTCTCTTCTTTATGTTTCCCTCTAAAATTTTCATTAATCAAAACCCCATTTGTAAATTTCCTTTTTTTGTGTGTGXXXXXXXXXXCAGCTTGGATTTGAAATTGGTTTCGTCGTTATCCTCGTCGCCTTCGTCGTCCTCGCCGAGGAGTAATTATCAGAGCGTTT

CCATTTTCTCTTCTTTCTGTTTCCCTCTAAAATTTTCATTAATCAAAACCCCATTTGTAAATTTCCTTTTTGTGTGTGTGXXXXXXXXXXCAGCTTGGATTTGAAATTGGTTTCGTCGTTATCCTCGTCGCCTTCGTCGTCCTCGCCGAGGAGTAATTATCAGAGCGTTT

>Marker72338

ATAATGGAGGGAGTAAATTGTTAAACTTATAAAAATTAAAAGTGTGTATGGATCAAAAGATTTTAAAGTAGGTTTGTATCXXXXXXXXXXATCTATAATAATTTGATTTTCATTTGCATTTTAAACTAGAAATAGTTTAGATACACAGCAATAATCACACATTTAAATAA

ATAATGGAGGGAGTAAATTGTTAAACTTATAAAAATTAAAAGTGTGTATGGATCAAAAGATTTTAAAGTAGGTTTGTATTXXXXXXXXXXATCTATAATAATTTGATTTTCATTTGCATTTTAAACTAGAAATAGTTTAGATACACAGCAATAATCACACATTTAAATAA

>Marker72368

CGTCTTTGCGGCATGCCATATTACATACAGAGACTTTATAATACTTGCAAAGCTGCATTGTCTCCTAATGGACCTGTGTCXXXXXXXXXXCAATTAAGTAGGATGTTGGAGGTTATACTGATAATAGTTAGATAATACAAAATATCCCCAAGGAGTAGTTATTTCAGTAA

CGTCTTTGCGTCATGCCATATTACATACAGAGACTTTATAATACTTGCAAAGCTGCATTGTCTCCTAATGGACCTGTGTCXXXXXXXXXXCAATTAAGTAGGATGTTGGAGGTTATACTGATAATAGTTAGATAATACAAAATATCCCCAAGGAGTAGTTATTTCAGTAA

>Marker72478

AACCCCACCTAATAAATTAATTAATTATTTAATTTACCACTCTCCCAACAAACTATTTTCTATTTTCTATTTTCTATTTTXXXXXXXXXXGCTTTTCAAAGAGTTTTAGTGCATTATTTCTTATCCCAACCTTTTTGCTACAGTATATATATCATTATTTTAATAATGGT

AACCCCACCTAATAAATTAATTAATTATTTAATTTACCACTCTCCCAACAAACTATTTTCTATTTTCTATTTTCTATTTTXXXXXXXXXXGCTTTTCAAAGAGTTTTAGTGCATTATTTCTTATCCCAACCTTTTTGCTACAGTATATATACCATTATTTTAATAATGGT

>Marker72522

ACGACTACCAAGCACCAATCTCGACCCTTAGACTATTTTTCTGTGTCAAATAATACGCAAAAGATAGGTTAGCAAAATTTXXXXXXXXXXTTTTGAATTTGTTCAAAATTGGGTCTTTTCTTTGTAGGCTATAGCATGACCATTTTTCGAACGCTTTGGGTGCTACCATA

ACAACTACCAAGCACCAATCTCGACCCTTAGACTATTTTTCTGTGTCACATAATACGCAAAAGATAGGTTAGCAAAATTTXXXXXXXXXXTTTTGAATTTGTTCAAAATTGGGTCTTTTCTTTGTAGGCTATAGCATGACCATTTTTCAAACGCTTTGGGTGCTACCATA

>Marker73223

TAGCTAGTAAACTGTTCATGCATATGTTACTTAAATTACACTGAAACAACTCCAATGATCCAAGAGGAAGTGAATGAATAXXXXXXXXXXGGTATAGAATAAAACAACGTCACAACGGGAAATAACAAAACAGAGTGTATGTCACTAAACAAAATTTATCAATCAATTCA

TAGCTAGTAAACTGTTCATGCATATGATACTTAAATTACACCGAAACAACTCCAATGATCCAAGAGGAAGTGAATGAATAXXXXXXXXXXGGTATAGAATAAAACAATGTCACAACGAGAAATAACAAAACAGAGTGTATGTCACTAAACAAAATTTATCAATCAATTCA

>Marker73354

CATTTGAAACAGATGAATTTGTTTTGGATGATTTAAATATTTTCCTAATAGAAAAATGCTTCATGTGACTTGAGAATCGAXXXXXXXXXXGGATATGCCCATTTTTTCGTTTTGGAGATTGCATGGTATTTTGTTGTAGCCAGTGTTTTAAAATGCCTAAGGCGCACTAA

CATTTGAAACAGATGAATTTGTTTTGGATGATTTAAATATTTTCCTAATAGAAAAATGCTTCATGTGACTTGAGAATCGAXXXXXXXXXXGGATATGCCCATTTTTGCGTTTTGGAGATTGCATGGTATTTTGTTATAGCCAGTGTTTTAAAATGCCTAAGGCGCACTAA

>Marker73441

TTGGTAGTAATGCCCCATTACCCTCTCAATTGTAATTTCCGTATCGAGTAATTACAAATCTAGCCTAATTTATCTAAACTXXXXXXXXXXATTTGATATAGTTTAGATAGATAATACATAGTTTATTCATAAGAATTGATAAACATCGCTGAGGTTGCCAACGATTTGAA

TTGGTAGTAATGCCCCATTACCCTCTCAATTGTAATTTCCATATCGAGTAATTACAAATCTAGCCTAATTTATCTAAACTXXXXXXXXXXATTTGATATAGTTTAGATAGATAATACATAGTTTATTCATAAGAATTGATAAACATCGCTGAGGTTGCCAACGATTTGAA

>Marker73554

TCCTTTATCAAAAATGAAATTAGAGAAGTTGTTTTTGAGATGGGTTGTCTTAAATCCCTAGTCTTGATGGCATTACCAGAXXXXXXXXXXTTCTTGTATAAAGTTATTTCCAAGGCGCTCGCAACAAGACATAAAAAAGTCCTTCCTTCGATGATTAATGATTCTCAAAC

TCCTTTATCAAAAATGAAATTAGAGAAGTTGTTTTTGAGATGGGTTGTCTTAAATCCCTAGTCTTGATGGCATTACCAGAXXXXXXXXXXTTCTTGTATAAAGTTATTTCCAAGGCGCTCGCAACAAGACTTAAAAAAGTCCTTCCTTCGATGATTAATGATTCTCAAAC

>Marker73589

CTATTTAACCATTTCCATTTTAATTTTTGTTTTTTAAATTTAAGTTTATTTTCTTTCTATATCTCATAACGATTGGTTTAXXXXXXXXXXTATAGGCAGAATTTTCAAAATCAAAATGCATACTAAATGAGACTTTAATTTTAAAAATCTTGTTTTTAGTTTCGAAATTT

CTATTTAACCATTTCCATTTTAATTTTTGTTTTTTAAATTTAAGTTTATTTTCTTTCTATATCTCATAACGATTGGTTTAXXXXXXXXXXTATAGGCAGAATTTTCAAAACCAAAATGCTTACTAAATGAGACTTTAATTTTAAAAATCTTGTTTTTAGTTTCGAAATTT

>Marker73737

TTGTGGTAGGGATAATGCGTGAAGAGACTTCTGTTGGTAAAACACGTTTCATAAAAGGAGTGTGTGATGGAAGGTCTTTTXXXXXXXXXXTGTAGTCATTCTGCTATGGGAAAATTCACTGAGGGCGACCTATAAATAGAAGAAATTTAGGTCTGTTTAAAGTTGCTTGT

TTGTGGTAGGGATAATGCGTGAAGAGACTTCTGTTGGTAAAACGCGTTTCATAAAAGGAGTGTGTGATGGAAGGTCTTTTXXXXXXXXXXTGTAGTCATTCTGCTATGGGAAAATTCATCGAGGGCGACCTATAAATAGAAGAAATTTAGGTCTGTTTAAAGTTGCTTGT

>Marker73954

TTATGTGATAATTATAAGTAAATGGCACGTGCATACATGTATTAGTTATCATTAAACTGTCAAATAAAATATAGAAATGTXXXXXXXXXXTAAAAATGTGCGGAGAACATGCCTTCTTGGCTAATTATCATACTCTCTCGACTTATTAAAGGAGGGAAAAAAAAGTCATA

TTATGTGATAATTATAAGTAAATGGCACGTGCATACATGTATTAGTTATCATTAAACTGTCAAATAAAATATAGAAATGTXXXXXXXXXXTAAAAATGTGCGGAGAACATGCCTTCTTGGCTAATTATCATACTCTCTCGACTTATTAAAGGAGGGAAAAAAAAGTAATA

>Marker74086

TTATATAATAATTTGTTTTCGTCTCAAGCAAGGCGATAAACTCAAAAATTAGTTGAAAACATTTCATATTTTTGTTCAGCXXXXXXXXXXATTAATCTAAAGTAACAAGTTAAACTGAATCTAAATTATACTTATCCTATAATTCTCCTCATCAGAGTTTTAACCAACAA

TTATATAATAATTTGTTTTCGTCTCAAGCAATGCGATAAACTCAAAAATTAGTTGAAAACATTTCATATTTTTGTTCAGCXXXXXXXXXXATTAATCTAAAGTAACAAGTTAAACTGAATCTAAATTATACTTATCCTATAATTCTCCTCATCAGAGTTTTAACCAACAA

>Marker74465

ATTGAACACCAACTTCACATCCAGGAAATGACCAAATAGGGTTGTTTTTTAAACATGTAAATCTCCTTAGGTGTTAACTTXXXXXXXXXXAAATCTCGATGAGATCAACATGAATTGCCAATTGAACTCATACAACATTGCATCGGGATGACACAAGATAAAGGTTTTAC

ATTGAACACCAACTTCACATCCAGGAAATGACCAAATAGGGTTGTTTTTTAAACATGTAAATCTCCTTAGGTGTTAACTTXXXXXXXXXXAAATCTCAATGAGATCAACATGAATTGCCAATTGAACTCATACAACATTGCATCGGGATGACACAAGATAAAGGTTTTAC

>Marker74525

TTTTACTATTAATTTTAGATGAAAGCATTGAACTTTAATTTTTTTTAAGAATACCCTATAAAACCTACCCTTGCCATTCTXXXXXXXXXXATATACATTAACAAAAGTAGTTAACTATTTTATTGATTGCGATATAGAAAAGCAAGGATAGATAGATCTAACCTGATTAA

TTTTACTATTAATTTTAGATGAAAGCATTGAACTTTAATTTTTTTTAAGAATACCCTATAAAACCTACCCTTGCCATTCTXXXXXXXXXXATATACATTAACAAAAGTAGTTAACTATTTTATTGATTGCGATATAGAAAAGCAAGGATAGATAGATCTAACCTGATCAA

>Marker74628

CCATCCCCCACTAGTCCAATCATAATTTTTATTTTTATTTTCAAGAATGATTTATTATTTTATGTTCTCTTTTTTTTTTTXXXXXXXXXXATCGCGTTCTGTCGTTGGGTTTGCATCTGTTGTTGTTTCCGTCTGCTCAGTGCGGCTGACTCCACGTCCAACCCTTTTAT

CCATCCCCCACTAGTCCAATCATAATTTTTATTTTTATTTTCAAGAATGATTTATTATTTTATGTTCTCTTTTTTTTTTCXXXXXXXXXXATCGCGTTCTGTCGTTGGGTTTGCATCTGTTGTTGTTTCCGTCTGCTCAGTGCGGCTGACTCCACGTCCAACCCTTTTAT

>Marker74674

ATCTCGAGGTCGTGTTCAAGCAAATTTATAAATCGGCAAGGATATAAAGCTTGAATCAATGTGTGAGTGTGAGAAATTAAXXXXXXXXXXGGATTCTTAAAGGGTTTGAAGCATATGATTTTGTAAACGTTTTTATCGAATCCAAGATATCTTAAATAGCTCTTAGCATG

ATCTCGAGGTCGTGTTCAAGCAAATTTATAAATCGGCGAGGATATAAAGCTTGAATCAATGTGTGAGTGTGAGAAATTAAXXXXXXXXXXGGATTCTTAAAGGGTTTGAAGCATATGATTTTGTAAACGTTTTTATCGAATCCAAGATATCTTAAATAGCTCTTAGCATG

>Marker74688

AGAACAAAGTGTTCTTATCGTTCTTCGTCTGGTCGTTTGAGTCAGCCAGGATCCAGTTTGGGTCACGTCTCCAAAACTTCXXXXXXXXXXGCACAACTAATTTGTCGTCGTGTCGTTTTTCATCGCGTCTATATCTAGTTGTGAGAACATCAGGATGAATTCTGCTACTA

AGAACAAGGTGTTCTTGTCGTTCTTCGTCTGGTCGTTTGAGTCAGCCAGGATCCAGTTTGGGTCACGTCTCCAAAACTTCXXXXXXXXXXGCACAACTAATTTGTCGTCGTGTCGTTTTTCATCGCGTCTATATCTAGTTGTGAGAACATCAGGATGAATTCTGCTACTG

>Marker74693

ATAATCATAAATTAGTTTTAAGAGAAGAGAGAGATCAAGTATTAGAAGCATGCGTTGAATTTTTTGTAGGTATCCAAAGCXXXXXXXXXXCCCCTGCTTTTCCTCTCTATCAATACACTCTCTCGAGGTTTTCTCATCATCACCTTTACGCTTCTCTCGTTGCTTAAGGG

ATAATCATAAATTAGTTTTAAGAGAAGAGAGAGATCAAGTATTAGAAGCATGCGTTGAATTTTTTGTAGGTATCCAAAGCXXXXXXXXXXCCCCTGCTTTTCCTCTCTATCAATACACTCTCTCGAGGTCTTCTCATCATCACCTTTACGCTTCTCTCGTTGCTTAAGGG

>Marker74941

TGATATTTGTCTGAATTAGTCTGGAACTATTGCTTAAACTGATGCATTCATATACTACAATCACTTTACAACCATGTAGTXXXXXXXXXXTTATTCTTATAAAAGAAAAAAAAAATTATAATTAACTGTAATCAGTAGATTGAAATGCCTAATATTATTTTCGTTGGGGT

TGATATTTGTCTGAATTAGTCTGGAACTATTGCTTAAACTGATGCATTCATATACTACAATCACTTCACAACCATGTAGTXXXXXXXXXXTTATTCTTATAAAAGAAAAAAAAAATTATAATTAACTGTAATCAGTAGATTGAAATGCCTAATATTATTTTCGTTGGGGT

>Marker74955

ACGCATTAGAATCAACACTACATGACATAGCAGATTAACTTCCTTGTCGGTTACATATCTTGTAGTTGATTTTGATGTTGXXXXXXXXXXTTATGTCTGGAAGAGACGAGGGACGGATTTACAAAGATGTATTGCAAATTTCCCTTCAAACCAAGAATAGATTAATACAA

ACGCATTAGAATCAACACTACATGACATAACAGATTAACTTCCTTGTCGGTTACATATCTTGTAGTTGATTTTGATGTTGXXXXXXXXXXTTATGTCTGGAAGAGACGAGGGACGGATTTACAAAGATGTATTGCAAATTTCCCTTCAAACCAAGAATAGATTAATACAA

>Marker75036

ACTTGTTTTAGTTTAACACATATTAATTGATTTGAATCTCATAATATGACACTTAGGCATATGTTGACCAAATTAGTGCGXXXXXXXXXXAAATCGTTCACCAATATGCAATATTTGGAATACACTTTTGCTTTCATAAGCTACGTTCCACTTTTGGGTCAACCGTGATA

ACTTGTTTTAGTTTAACACATATTAATTGATTTGAATCTCATAATATGACACTTAGGCATATGTTGACCAAATTAGTGCGXXXXXXXXXXAAAACGTTCACCAATATGCAATATTTGGAATACGCTTTTGCTTTCATAAGCTACGTTCCACTTTTGGGTCAACCATGATA

>Marker75384

ATTGAGATTTACCCTTATTCATTATTTACAAAAATGTCTACTGAAAAAAAGAGCTTGATTAGAATAAATTTGTTTGTTTTXXXXXXXXXXACATTGCCGAAAAAATCATTCCAGAATATGTTTCATCCCACCTTATGGTGTCCATTAGAAGAGAAATCTCTTTTTGTTGG

ATTGAGATTTACCCTTATTCATTATTTACAAAAATGTCTACTGAAAAAAAGAGCTTGATTAGAATAAATTTGTTTGTTTTXXXXXXXXXXACATTGCCGAAAAAATCATTCCAGAATATGTTTCATCTCACCTTATGGTGTCCATGAGAAGAGAAATCTCTTTTTGTTGG

>Marker75481

ATGGGAGACCAACTATGCCTACTCCATCAGTTCGTCAACAACTAACCAACTCCATCACGCTCTCTGTAGCCACCCTCGCCXXXXXXXXXXCTCTGCTCCTAACAACCCTGTCACCTGTAATTGTAAGTTTTTATGATTTCATTTTTAGGGTTTTTTCTCTGTCCGTAAAT

ATGGGAGACCAACTATGCCTACTCCATCAGTTCGTCAACAACTAACCAACTCCATCACGCTCTCTGTAGCCACCCTCGCCXXXXXXXXXXCTCTGCTCCTAACAACCCTGTCACCTGTAATTGTAAGTTTTTATGATTTCATTTTTAGGGTTTTTCCTCTGTCCGTAAAT

>Marker75587

ACGTTTGTAATATTTCACATATTTATGATTTTCAAACAATAATCTTAATAAATTGTTTAAATAGATTCAGTTATGAAAATXXXXXXXXXXAAATATGTAAACATTGAAATTGATGTTACAATATGCATTAAAGTCCGTTAGTTGCAAGAAAAAAAGGTTGCTTGCAAATA

ACGTTTGTAATATTTCACATATTTATGATTTTCAAACAATAATCTCAATAAATTGTTTAAATAGATTCAGTTATGAAAATXXXXXXXXXXAAATATGTAAACATTGAAATTGATGTTACAATATGCATTAAAGTCCGTTAGTTGCAAGAAAAAAAGGTTGCTTGCAAATA

>Marker75832

AAATCTTTGGCTTGAGTTTACTCCATTAGATCCTTGGAATGTGAAATGGAAACTCTTAGTTCCTTACTGTGAATTGTCTCXXXXXXXXXXACTATCTTTCCAGATCGTTTTTCCTCTTTTTTCTATTCTTGGAATTAAATGAGCGTTCTGTCTGCTTGCAGCAAAGACCA

AAATCTTTGGCTTGAGTTTACTCCATTAGATCCTTGGAATGTGAAATGGAAACTCTTAGTCCCTTACTGTGAATTATCTCXXXXXXXXXXACTATCTTTCCAGATCGTTTTTCCTCTTTTTTTTATTCTTGGAATTAAATGAGCGTTCTGCCTGCTTGCAGCAAAGACCA

>Marker75963

CTATAGACTACCCAATGTATCACAGTATACTTGAAGTTAATCAAGATTCAACCACAATCTATCAAAGTAATTGGATTTTCXXXXXXXXXXTCAAACTAATAAAGCAAAATTTTCATTAATTGAACGCTTTTGAAGGCTATAGAGATATGCAAAATAGATTGTTTACCTCA

CTATAGACTACCCAATGTATCACAGTATACTTGAAGTTAATCAAGATTCAACCACAATCTATCAAAGTAATTGGATTTTCXXXXXXXXXXTCAAACTAATAAAGCAAAATTTTCATTAATTGAACGCTTTTAAAGGCTATAGAGATATGCAAAATAGATTGTTTACCTCA

>Marker76066

ACTCATACCTTACCTCTTCCTCTTCAATTTATATTTGGACCCATGACTATCCTTATTGGATTTGTCTGTTTTTCCTTTTTXXXXXXXXXXAAACTAAAGATAGTTTTTGTTTGGAAGGGAACAACGATCTATTTTAAAACACAACATAAATATGTATATACAAAGTTTAA

ACTCATACCTTACCTCTTCCTCTTCAATTTATATTTGGACCCATGACTATCCTTATTGGATTTGTCTGTTTTTCCTTTTTXXXXXXXXXXAAACTAAAGATAGTTTTTGTTTGGAAGGGAACAACGATCTATTTTAAAACACAAGATAAATATGTATATACAAAGTTTAA

>Marker76118

ATGTTCATATATATTTTTAAATTTAGGAGGAGGTATATCAAATAAAACTTCAAAATAATTATTTTAAATCTGTAATAAATXXXXXXXXXXCAACTGGCATTGGCATTATTATTCAAAATCCAAAAGAAATGATTGTAAGGGCTGTTTATGGATACAGAAGAACAAGTTAT

ATGTTCATATATATTTTTAAATTTAGGAGGAGGTATATCAAATAAAGCTTCAAAATAATAATTTTAAATCTGTAATAAATXXXXXXXXXXCAACTGGCATTGGCATTATTATTCAAAATCCAAAAGAAATGATTGTAAGGGCTGTTTATGGATACAGAAGAACAAGTTAT

>Marker76133

TTAGCATTTTTTTTAGAACAAAGAGAGGTGCATCCTCAGTTCCAAAAGTGGTTGACTAAACTCCTAGGATTTGATTTTGAXXXXXXXXXXATAAGAAAAGAATAGTATTGTCTAAAACTTCTTCCTTAATAGCCACCCTGCTACATACGTTTTATGATTCTGTTTTGGGA

TTAGCATTTTTTTTAGAACAAAGAGAGGTGCATCCTCAGTTCCAAAAGTGGTTGACTAAACTCCTAGGATTTGATTTTGAXXXXXXXXXXATAAGAAAAGAATAGTATTGTCTAAAACTTCTTCCTTAATACCCACCCTGCTACATACGTTTTATGATTCTGTTTTGGGA

>Marker76359

TTTTTCTGTGTATGGGGCTCTCTCCCTATGATTGTAAAATTTGAAATCAAGATCTTCTGCTTTAATAAATTAAAAGCATAXXXXXXXXXXCACTTGTAGGGATCAACCTCCATATTTTTTAAACTTTGATCTTTAGTTTGTGAGTATTGTTAGATCCCACAGTTTTTAAA

TTTTTCTGTGTATGGGGCTCTCTCCCTATGATTGTAAAATTTGAAATCAAGATCTTCTGCTTTAATAAATTAAAAGCATAXXXXXXXXXXCACTTGTAGGGATCAACCTCCATATTTTTTTAACTTTGATCTTTAGTTTGTGAGTATTGTTAGATCCCACAGTTTTTAAA

>Marker76444

ATTATATGTTCTTAGTAATGCCTTTTGGATTGACCAATATCCTAGCTGCTTTCGTGGACTTAATGAATAGAGTGTTTCTCXXXXXXXXXXTCACGAAAAATTGAGGCAATTGAAAGTTGGGAACATCCTAACACAGTTTCTGAAGTTCGGAGCTTCCTTGGTTTGGCATG

ATTATATGTTCTTAGTAATGCCTTTTGGATTGACCAATATCCTAGCTGCTTTCGTGGACTTAATGAATAGAGTGTTTCTCXXXXXXXXXXTCACAAAAAATTGAGGCAATTGAAAGTTGGGAACATCCTAACACAGTTTCTGAAGTTCGGAGCTTCCTTGGTTTGGCATG

>Marker76558

ATTTGAAATAAACTTTAGAAAAGAACTAGTGCCAAAAGAAATGTTAATACTGTATCCTTATATAACAAATCTTAGCGTGTXXXXXXXXXXGGAACAGAGAATATTAAACAGTGAAAATGGAAGAGAAAATGACAATTAGTGTGGAGGATGATTGTGAAATGGTTAATGTT

ATTTGAAATAAACTTTAGAAAAGAACTAGTGCCAAAAGAAATGTTAATCCTGTATCCTTATATAACAAGTCTTAGCATGTXXXXXXXXXXGGAATAGAGAATATTAAACAGTGAAAATGGAAGAGAAAATGACAATTACTGTGGAGGATGATTGTGAAATGGTTAATGTT

>Marker76566

ATCAAACTTCTACTAGTGTTAAGATTAGACCACCAAACTTACAATGTGGTAGAATTGGACCCCTAAATGAAACTTCTACAXXXXXXXXXXATAACTTTTAAACGTATTGATATAATAAAACTTGCTAACTCATCAAATTAAACTTTTGGGTTGACTGGTAATTTAATATG

ATCAAACTTCTACTAGTGTTAAGATTAGACCACCAAACTTACAATGTGGTAGAATTGGACCCCTAAATGAAACGTCTACAXXXXXXXXXXATAACTTTTAAACGTATTGATATAATAAAACTTACTAACTCATCAAATTAAACTTTTGGGTTGACTGGTAATTTAATATG

>Marker76685

AACCTGCACATAGTTTAGACAGTTTATACATACATTTCCAATAACAAAAAGTGAAAAGCTTGAATCTCCCAATTACCTCAXXXXXXXXXXAAGAGAATGGGCAGCCCATCAACATCCCTTCATTTGCTACAAATACACACTCTCTATTTCAAAATGCACCCATTCTTTTT

AACCTGCACATAGTTTAGACAATTTATACATACATTTCCAATAACAAAAAGTGAAAAGCTTGAATCTCCCAAATACCTCAXXXXXXXXXXAAGAGAATGGGCAGCCCATCAACATCCCTTCATTTGCTACAAATACACACTCTCTATTTCAAAATGCACCCATTCTTTTT

>Marker76737

TTACTTTATTTTATTGATAAAATGAAACAAATGGGGTAAGAACCCAAACACCTAAAGAGGCTATGGTCAGAAAAAAGTGTXXXXXXXXXXCGGGATTTGTTTGCCACGTAAAGGCATTGATGTCTTGTGATAGAAGACATTCTAATTCACCCCCCATCCTCAAGTGGGAA

TTACTTTATTTTATTGATAAAATGAAACAAATGGGGTAAGAACCCAAACACCTAAAGAGGCTATGGTCAGAAAAAAGTGTXXXXXXXXXXCGGGATTTGTTTGCCACGTAAAGGCATTGATGTCTTGTGATAAAAGATATTCTAATTCACCCCCCATCCTCAAGTGGGAA

>Marker76815

CTGAGACATAATGGACCGAAAAATCACCAAGATTACATCAAGAGATATGTGAGGCACCCAAGAGATACACCGACGCAAGCXXXXXXXXXXGATACTGTAAGCTCACATTCCGCTCTCCTTTATTTATTTTAATATCTCCTCAGTTATGAAAAACAAATTAGAATACTTTT

CTGAGACATAATGGACCGAAAAATCACCAAGATTACATCAAGAGATATGTGAGGCACCCAAGAGATACACCGACGCAAGCXXXXXXXXXXGATACTGTAAGCTCACATTCCGCTCTCCTTTATTTATTTTAATATCTCCTCGGTTATGAAAAACAAATTAGAATACTTTT

>Marker77315

ATTGATTATTGGAAGTAAAATGGAGAGATTATTTGAAGAAAATGAGTAAGAGGTAGGAAAGGAAGCAATGAAGAGACGATXXXXXXXXXXGAAAAGGATAAGAAGAATTTCGAAGAGTTTAGTTTATATTTGCTTGGTATGTTGAAAGCCGTCGTCGTTTTGTCTCTCAG

ATTGATTATTGGAAGTAAAATGGAGAGATTATTTGAAGAAAATGAGTAAGAGGTAGGAAAGGAAGCAATGAAGAGACGATXXXXXXXXXXGAAAAGGATAAGAAGAATTTCAAAGAGTTTAGTTTATATTTGCTTGGTATGTTGAAAGCCGTCGTCGTTTTGTCTCTCAG

>Marker77403

ATTATGGGATCTATGTTTATTTGAACTAGTCTGATGTGCTACTTTGACGACGACATTCTAGACTATATTGCTAGTATTTCXXXXXXXXXXAATCCTCCATTTTAGAGTTACCTTTTATTGTTGTCTAAAAGGTAAGGTTTGTTAAAGAAAGCCTTCCTCAAGTCTCGATT

ATTATGGGATCTATGTTTATTTGAACTAGTCTGATGTGCTACTTTGACGACGACATTCTAGACTATATTGCTAGTATTTCXXXXXXXXXXAATCCTCCATTTTAGAGTTACCTTTTATTGCTGTCTAAAAGGTAAGGTTTGTTAAAGAAAGCCTTCCTCAAGTCTCGATT

>Marker77566

CCAGGTATTAACAAGTAGATCATATGTTATAAGACCTAATTATAATGACTCGAGACACTCTAACATTCATGAACAACTTCXXXXXXXXXXTGAGTTTAGAAACATTCAGAAATATATAACTTATTGTTTGTAATTAATGATTATATGAAATTGTTAAATTCTCTTATAGA

CCATGTATTAACAAGTAGATCATATGTTATAAGACCTAATTATAATGACTCGAGACACTCTAACATTCATGAACAACTTCXXXXXXXXXXTGAGTTTAGAAACATTCAGAAATATATAACTTATTGTTTGTAATTAATGATTATATGAAATTGTTAAATTCTCTTATAGA

>Marker77966

CATCAAAAATATATTTTTCATATTTTGTTGGATCCTGTTCCCACTGCAGTCAAATTTTCTGTGCCTGAAGTTAGTTACTTXXXXXXXXXXTTAGTCCTGGGATGTCAAATTTTAAGCTATTCGTCGATGACTACAAGAGCCATGTTTCTTTATGATTATCAAAATTAAAA

CATCAAAAATATACTTTTCATATTTTGTTGGATCCTGTTCCCACTGCAGTCAAATTTTCTGTGCCTGAAGTTAGTTACTTXXXXXXXXXXTTAGTCCTGAGATGTCAAATTTTAAGCTATTCGTCGATGACTACAAGAGTCATGTTTCTTTATGATTATCAAAATTAAAA

>Marker78173

ACAGAACAGAATCCTAGCTTTTACACTACGCACCATCCATTATCCATGCAATGTATTTCATTGAGGTTTCTGCTTTATGGXXXXXXXXXXAAACTCGAAGGACGATATTCTAAAAAGGGAGCTTATGTAGCTGCAAATTTAGCTTCACAATGTCTTACGAGTGAACCGAG

ACAGAACAGAATCCTAGCTTTTACACTACGCACTATCCATTATCCATGCAATGTATTTCATTGAGGTTTCTGCTTTATGGXXXXXXXXXXAAACTCGAAGGACGATATTCTAAAAAGGGAGCTTATGTAGCTGCAAATTTAGCTTCACAATGTCTTACGAGTGAACCGAG

>Marker78264

CGTTAGCATCTAGTATGTTTTTTTGGTTTTCCCCCTTACTGTTTGCTAATGTCACACTTTGAATTGTTGTTATTGAAGAAXXXXXXXXXXCTCCAAATGGTATATCTTCAGTTGAATGGATGGAAGGATCATTGGCTGCACAAGCACCACAACCACTGACTTGGCACAAG

CATTAGCATCTAGTATGTTTTTTTGGTTTTCCCCCTTACTGTTTGCTAATGTCACACTTTGAATTGTTGTTATTGAAGAAXXXXXXXXXXCTCCAAATGGTATATCTTCAGTTGAATGGATGGAAGGATCATTGGCTGCACAAGCACCACAACCACTGACTTGGCACAAG

>Marker79348

TTAATCAGACCAAATTTACCCCTCGAGCTCAGGCATGTGTGTTTGTTGGGTATCCCCTTCACCGGCGTGGTTATAAATGTXXXXXXXXXXCCCTACTAGTCATCCGTCGGCTCCAGTCCAAGACTTTGAACCTCCTCGAGATCAAGGTATGGAAAACCCTACTGAACCTT

TTAATCAGACCAAATTTACCCCTCGAGCTCAGGCATGTGTGTTTGTTGGGTATCCCCTTCACCAGAGTGGTTATAAATGTXXXXXXXXXXCCCTACTAGTCAGCCGTCGGCTCCAGTCCAAGACTTTGAACCTCCTCGAGATCAAGGTATGGAAAACCCTACTGAACCTT

>Marker79475

CCATTGACGAGGGACGGACTTTGGTTGCTAGAGGAGAAGCCTCTTTCTCCACTAGAAAAATGGTTGAATGATTTAGGACAXXXXXXXXXXCCAAAGACAGAAAATTCGAACACTTTTACCTGATCAATTGCACATACATTTTTTGAATAGTGGAAAGTGGAAATGAGAAT

CCATTGACGAGGGACGGACTTTGGTTGCTAGAGGAGAAGCCTCTTTCTCCACTAGAAAAATGGTTGAATGATTTAGGACAXXXXXXXXXXCCAAAGACTGAAAATTCGAACACTTTTACCTGATCAATTGCACATACATTTTTTGAATAGTGGAAAGTGGAAATGAGAAT

>Marker79852

ATAGCACAACCAAGTAGGGTGGCATGCCCTCAGGCTACATGCGTGACTTTCCTAGAAGTGGTTAGCGTGCAGGCTGTGCGXXXXXXXXXXCCGATCCGCAAAACAATCAGAACACAGAAAGTAGTGCAAATAGAACGCAATTTAGCTTAAAACCTTGAAACTGGTCTTAT

ATAGCACAACCAAGTAGGGTGGCATGCCCTCAGGCTACACGCGTGACTTTCCTAGAAGTGGTTAGCGTGCAGGCTGTGCGXXXXXXXXXXCCGATCCACAAAACAACCAGAACGCAGAAAGTAGTGCAAATAAAACGCAATTTAGCTTAAAACCTTGAAACTGGTCTTAT

>Marker79859

TTTGTATGTAAAGAAGACGAGAATCATCCAATACAATGCACCAGTTCTTTTTCAAATAAAGAAAATAATATATAATATATXXXXXXXXXXGTTTTATACGATCTGAGAGCCCAAAGCTGTAGTTTTAGCCTGCAAAAAGGTAGAGCTAACATCTCTAATTACAATTTAAG

TTTGTATGTAAAGAAGACGAGAATCATCCAATACAATACACCAGTTCTTTTTCAAATAAAGAAAATAATATATAATATATXXXXXXXXXXGTTTTATACGATCTGAGAGCCCAAAGCTGTAGTTTTAGCCTGCAAAAAGGTAGAGCTAACATCTCTAATTACAATTTAAG

>Marker80394

AAGGACCTTTCATTTGTTTTTTTAAGATTTGCACGGTATTTCTCGTCCTGCACTCAAATCATCTCCATCAGAGTAAATATXXXXXXXXXXCAGCTTCAAAATATAAATTACCAAACCAGGAAGTTGAATCATTTGCTTTCAAATAAAACAAACATACCTCGACTAACACC

AAGGACCTTTCATTTGTTTTTTTAAGATTTGCACGGTATTTCTCGTCCTGCACTCAAATCATCTCCATCAAAGTAAATATXXXXXXXXXXCAACTTCAAAATATAAATTACCAAACCAGGAAGTTGAATCATTTGCTTTCAAATAAAACAAACATACCTCGACTAACACC

>Marker80398

CAAGCTTGTTTAAGAAATGATTGGAAAAGTAAATGAGATTTCACAGATGCACAACGAACTTGAGTTCGACAAGGATTTTAXXXXXXXXXXATGAATTGGACTTATTGTTGAGATGAGATACTCGAAATAGTATATGAGATGTGGTAAAGTTGAAAACAAGATGATCTTTT

CAAGCTTGTTTAAGAAAGGATTGGAAAAGTATATGAGATTTCATAGATGCACAACGAACTTGAGTTCGACAAGGATTTTAXXXXXXXXXXATGAATTGGACTTATTGTTGAGATGAGATACTCGAAATAGTATATGAGATGTGGTAAAGTTGAAAACAAGATGATCTTTT

>Marker80682

ATATATTATCATTTTTTTTTATCATAACAAAATTACTATTGATGAAATAAGTTTATATGTGGAGAATCTTTTGAAAATAAXXXXXXXXXXATATGAAACTCTATGTTATCTAGTAGTTTAGATCCTTAAAAATAAAAATTTGGAAAGTGAGAGATTCAATAGATACAAAT

ATATATTATCATTTTTTTTTATCATAACAAAATTACCATTGATGAAATAAGTTTATATGTGGAGGATCTTTTGATAATAAXXXXXXXXXXATATGAAACTCTATGTTATCTAGTAGTTTAGATCCTTAAAAATAAAAATTTGGAAAGTTAGAGATTCAATAGATACAAAT

>Marker80836

ACATGAAAATAACGCTCACTTTTCTGCTCCGCTAAACGTCCTTGTTGAATCACCAAAGCATCGAGGGCAAGTTTCTTGTAXXXXXXXXXXGGATTCCTGAAATAAACGTTGCATGTCAGAGAATAATAGGGAGCGACAACAGCAGCAGCAATAATCTTGATCAAAGAGAT

ACATGAAAATAACGCTCACTTTTCTGCTCCGCTAAACGTCCTTGTTGAATCACCAAAGCATCGAGGGCAAGTTTCTTGTAXXXXXXXXXXGGATTCCTGAAATAAACGTTGCATGTCAGAGAATAATAGGGAGCGACAACAGCAGCAGCAATAATCTTGATCAAAGTGAT

>Marker80892

TTGAAAATTAGATAGATGTAATATAACAGGGCAAAACGATTAATCATCACTAAGAAAAGTTTATTACACACACGTAATTGXXXXXXXXXXATTAACGTAATTATTACCTTGTCATCTCCTTGCTCAAAATCAAAAGCACCGGGTCCATCAGTGGTTCCAGCAGCAAAAGA

TTGAAAATTGGATAGATGTAATATAACAGGGCAAAATGATTAATCATCACTAAGAAAAGTTTATTACACACACGTAATTGXXXXXXXXXXATTAACGTAATTATTACCTTGTCATCTCCTTGCTCAAAATCAAAAGCACCGGGTCCATCAGTGGTTCCAGCAGCAAAAGA

>Marker80947

ACTCATCACAAATCAATTTACTCTGATCAGTTTGCACGAATTTTTTGTGTAGATTCGGTCGCTACTGCCTTGTTTTACTGXXXXXXXXXXGAAAAACTTATATATATATATGTATTTATTGATGTGGACTTGTGGTTGAGTGCATGTTGTGACGGACTCGATATGTATAT

ACTCATCACAAATCAATTTACTCTGATCAGTTTGCACGAATTTTTTGTGTAGATTCGGTCGCTACTGCCTTGTTTTACTGXXXXXXXXXXAAAACTTATATATATATATATGTATTTATTGATGTGGACTTGTGGTTGAGTGCATGTTGTGACGGACTCGATATGTATAT

>Marker80985

ATAAATGCCTTGTTGTCGTCGCCCGACTATTTTAGCATATATAGATTTAGGCATATGCCCTCAATGAAAAGGTTAGAGGTXXXXXXXXXXCAAAATAATGGGTTTTGTTTAGTTCCCCCCGCTTTCCTTGGACAAAAAAATAAAGCTGTTACTATTGTGGTGTCCTTGAG

ATAAATGCCTTGTTGTCGTCGCCCGACTATTTTAGCATATATAGATTTAGGCATATGCCCTCAATGAAAAGGTTAGAGGTXXXXXXXXXXCAAAATAATGGGTTTTGTTTAGTTCCCCCCGCATTCCTTGGACAAAAAAATAAAGCTGTTACTATTGTGGTGTCCTTGAG

>Marker81060

AAATAGAATTAATGACACCTCAATTTTTTTAATCAAACTTTCTTTTTCTAATGTCCTCCATTAAAAGAACAAATTAATGGXXXXXXXXXXATAGGTTTCATTAGTCTCCGTTTGCTACAACTGCAAACTGAATTCCAAATATTTATGCTTCTTTTTCTTTGTTTATCGAT

AAATAGAATTAATGACACCTCAACTTTTTTAATCAAACTTTCTTTTTCTAATGCCCTCCATTAAAAAAACAAATTAATGGXXXXXXXXXXATAGGTTTCATTAGTCTCCGTTTGCTACAACTGCAAATTGAATTCCAAATATTTATGCTTCTTTTTCTTTGTTTATCGAT

>Marker81085

ATTGCAGTGGGCAGTTTTTGCCCAGCGGTTACAGCTACTGGGTGCAAACTCAGTAGTTTTGGGGAACGCTAAGCTAATAAXXXXXXXXXXTCTGGCAACTAGTGCTGTTGAGCGTCTGATATGTATGAGCTATCAAATTTCATATTGCTTGAGTGATTATTTAACAATTT

ATTGCAGTGGGCAGCTTTTGCCCAGCGGTTACAGCTACTGGGTGCAAACTCAGTAGTTTTGGGGAACGCTAAGCTAATAAXXXXXXXXXXTCTGGCAACTAGTGCTGTTGAGCGTCTGATATGTATGAGCTATCAAATTTCATATTGCTTGAGTGATTATTTAACAATTT

>Marker81347

ATTCTCCATAAGTTTTGATTGTAGGCATTCCAACTATTCCCCAAATCTTCTCTATTAGTCTATAAATGATTGGATACAATXXXXXXXXXXCAACTCTCGACACTTCTTCGTTAGACATATTCTGGGAATAGTCACCAATTTCCTTCGTAACCAACCCAAATCCTCCTGTC

ATTCTCCATAAGTTTTGATTGTAGGCATTCCAACTCTTCCCCAAATCTTCTCTATTAGTCTATAAATGATTGGATACGATXXXXXXXXXXCAGCTCTCGACACTTCTTCGTTAGACATATTCTGGGTATAGTCACCAATTTCCTTCGTAACCAACCCAAATCCTCCTGTC

>Marker81469

ATCCTCTAGGGGGACATCGTTGCTGTGTCATGCCACGTTGGACCTGATGTCCACAATCAACTCTACACGGCATCAGTCATXXXXXXXXXXCCCAGACCAAATTTTTAAACACGTCCCTCTTAGTGATGATGCACTTGATGTCATCGTTCCAACCGAAGCCACTATCTGTT

ATCCTCTAGGGGGACACCGTTGCTGTGTCATGCCACGTTGGACCTGATGTCCACAATCAACTCTACACGGCATCAGTCATXXXXXXXXXXCCCAGACCAAATTTTTAAACACGTCCCTCTTAGTGATGATGCACTTGATGTCATCGTTCCAACCGAAGCCACTATCTGTT

>Marker81720

TATTTGGTCATAGAAGTTAGTTTTGCAGTCATGATGAAACTCGGTTATGCCCATCTTGAGGTCGCTAGGTCATTGTCAATXXXXXXXXXXACCTGTTGATCACTCTAAAGTTGAGATTCATAGTTCATATACCATTGCATGTGCTAGACATTTAATAATTCAAATTGAGA

TATTTGGTCATAGAAGTTAGTTTTGCAGTCATGATGAAACTCGGTTATGCCCATCTTGAGGTCACTAGGTCATTGTCAATXXXXXXXXXXACCTGTTGATCACTCTAAAGTTGAGATTCATAGTTCATATACCATTGCATGTGCTAGACATTTAATAATTCAAATTGAGA

>Marker81869

CAAGCCCCTTCCAAATGCTTCGTCTGTTTTCTGTCTTGTAGTTGAGACGTGTTTAATGATTTACATCTCACCTGCAGACAXXXXXXXXXXGTTCATTTTTTTCATGTGCGGAGGCTTGGAACAGTGCACTAATTGGCATCAACTTTTAGCTATGTGTAGAGCTTTGTTTG

CAAGCCCCTTCCAAATGCTTCGTCTGTTTTCTGCCTTGTAGTTGAGACGTGTTTAATGATTTACATCTCACCTGCAGACGXXXXXXXXXXGTTCATTTTTTTCATGTGCGGAGGCTTGGATCAGTGCACTAATTGGCATCAACTTTTAGCTATGTGTAGAGCTTTGTTTG

>Marker82407

TTGCTTTCATGGCAGGTTGTCTAACTAGCTAGGCTAGCGTGTGTGGTGTTGAGTGAGTCCAAAAACCTAAACACATGAAGXXXXXXXXXXGTTTGTTGTTAAGCTATTTGACTATATGTGTTTGTAATTTATCAATTTTTCTTAATGTATCAATGTAAATTATTGCTAAT

TTGCTTTCATGGCAGGTTGTCTAACTAGCTAGGCTAGCGTGTGTGGTGTTGAGTGAGTCCAAAAACCTAAACACATGAAGXXXXXXXXXXGTTTGTTGTTAAGCTATTTGACTATATGTGTTTGTAATTTATCAATTTTTCTTAATGTATCAATGTAAATTATTCCTAAT

>Marker82467

CATGACATAAGTCTCTACAAAAATCAGTGTCTAAAACCACAAGATGAGTGAAAAAGAATGCAAAAGATTATGTATGCTTCXXXXXXXXXXGTGTTTTGCTTGAATGAAGGTGATATGAGTTGGAAGACTTCCAGACAAGCAAGTGTTGCCAATTCCACTATAGAAGCAGA

CATGACATAAGTCTCTACAAGAATCAGTGTCTAAAACCACAAGATGAGTGAAAAAGAATGCAAAAGATTATGTATGCTTCXXXXXXXXXXGTGTTTTGCTTGAATGAAGGTGATATGAGTAGGAAGACTTCCAGACAAGCAAGTGTTGCCAATTCCACTATAGAAGCAGA

>Marker82514

AAACAACCCCGTAATGTAGTTCTCTCTCTATGTGACTTTTTGTGTGAATGCAGGGAGACTTTAGTTTCCATGAGAAAACTXXXXXXXXXXCAAACAAAGCCAATGAATCTTCCATCTTTGGTCATTCACCCACTGAAAAATGAAGTCCTTAATGCTTCTCAAAAAGGGAA

AAACAACCCCATAATGTAGTTCTCTCTCTATGTGACTTTTTGTGTGAATGCAGGGAGACTTTAGTTTCCATGAGAAAACTXXXXXXXXXXCAAACAAAGCCAATGAATCTTCCATCTTTGGTCATTCACCCACTGAAAAATGAAGTCCTTAATGCTTCTCAAAAAGGGAA

>Marker82519

ATGCTTGGTTCTGTGAAGATCTCTTACTTTTTGACAATATGATTGCCTTATTGGGTGAAGGCTATCCGAAGTTTGACCTCXXXXXXXXXXTTTTCTCTCTCCCTCTGATTTATTTTTGTTTTAACTCTCTAAATTTGTCTTTTATCCTTCCTTTTTTTCCTTGGAAACGT

ATGCTTGGTTCTGTGAAGATCTCTTACTTTTTGACAATATGATTGCCTTATTGGGTGAAGGCTATCCGAAGTTTGACCTCXXXXXXXXXXTTTTCTCTCTCCCTCTGATTTATTTTTGTTTTAACTCTCTAAATTTGTCTTTTATCCTTTCTTTTTTTCCTTGGAAACGT

>Marker82676

CAAAGAGAACTCAAAAGACAAAAACAGATCAGATTCCCAGTTCCGTTAGCCATTCCAGATTCAATTAACTAAAGAAAACGXXXXXXXXXXAAACCTCTGGATTCCCAATGGCAATGTCTCCAAAGTGGGTCAAATTATGAGCGAGATGAGCCATCCTAAGGGCAAGAGCA

CAAAGAGAACTCAAAAGACAAAAACAGATCAGATTCCCAGTTCCGTTAGCCATTCCAGATTCAATTAACTAAAGAAAACGXXXXXXXXXXAAACCTCTGGATTCCCAATGGCAATGTCTCCAAAGTGGGTCAAATTATGAGCGAGATGAGCCATCCTGAGGGCAAGAGCA

>Marker82817

TTGCTTATCATTAGTATTACTGAAGTTATATATATTTCTTAGACACTCAGCCTCTCAAACATACATGGTTTTATTTGTGAXXXXXXXXXXGATTCATCTTACTCTTCGTATCTGGCTTCTTTGCGGATAGTTTTTGTATTATCTACCAAATAAAAAGATTGAAAATATCA

TTGCTTATCATTAGTATTACTGAAGTTATATATATTTCTTAGACACTCAGCCTCTCAAACATATGTGGTTTTCTTTGTGAXXXXXXXXXXGATTCATCTTACTCTTCGTATCTGGCTTCTTTGCGGATAATTTTTGTATTATCTACCGAATAAAAAGATTGAAAATATCA

>Marker82881

CAACAGAGCTCATATGGTGCCTTAATAACTCCATCGATCATAACTTTTCTAAGTTGTATTTTTCTTTTTCCTTCTTCCACXXXXXXXXXXAAAGTTCAGTTACAGTACAGTTAAGGTCATGTCAAATTAAATGAGGTGTGTTAAACGTGACAAACTACTCTTGGTTCGAG

CAACAGAGCTTATATGGTGCCTTAATAACTCCATCGATCATAACTTTTCTAAGTTGTATTTTTCTTTTTCCTTCTTCCACXXXXXXXXXXAAAGTTCAGTTACAGTACAGTTAAGGTCATGTCAAATTAAATGAGGTGTGTTAAACGTGACAAACTACTCTTGGTTCGAG

>Marker82948

AATATTTATATTTTCTTATCAGTTAGGCTTTTGTTCACGATGTTAGCTCTAAGAAAGTCATATTTATCTCGTTTTTTTTAXXXXXXXXXXTAAATAAGAAAGTCATAGTTATGTTGTTTTTGTTTTTTTATAAGAAACAAATTTCATTGATGTATGAAATTTACAAAAGA

AATATTTATATTTTCTTATCGGTTAGGCTTTTGTTCACGATGTTAGCTCTAAGAAAGTCATATTTATCTCGTTTTTTTAGXXXXXXXXXXTAAATAAGAAAGTCATAGTTATGTTGTTTTTGTTTTTTTATAAGAAACAAATTTCATTGATGTATGAAATTTACAAAAGA

>Marker82950

AAATTCTGTCACAGTCACTGTGAATAATCTTCTTCAAATGTAACTGTCATGCCAGTGGCATCCTTGTTTGCTTGTGCTTCXXXXXXXXXXTTGCAGATTACTGCATTCCCGACCCTGAATTACTGTGTATTTCTCTACATTACTTTGCGAAAACCTCCATAATAAATGGA

AAATTCTGTCACAGTCACTGTGAATAATCTTCTTCAAATGTGACTGTCATGCCAGTGGCATCCTTATTTGCTCGTGCTTCXXXXXXXXXXTTGCAGATTACTGCATTCCCGACCCTGAATTACTGTGTATTTCTCTTCATTACTTTGCGAAAACCTCCATAATAAATGGA

>Marker83086

TATGACTATATCCATGAGAAAAGGTATATTTCACTCTTAAAAACTATGAAAAATATGCGTAAAAATATTTTGAGCACTGTXXXXXXXXXXTTCTTGTGATGAGGAAGTTTGAGTTTCGAAAAGACCTTTCTAATTGCTTTATCAATGGGGAAGGATAGTAAAAGTTCGTT

TATGACTATATCCATGAGAAAAGGTATATTTCACTCTTAAAAACTATGAAAAATATGCGTAAAAATATTTTGAGCACTGCXXXXXXXXXXTTCTTGTGATGAGGAAGTTTGAGTTTCGAAAAGACCTTTCTAATTGCTTTATCAATGGGGAAGGATAGTAAAAGTTCGTT

>Marker83097

GAAGCACATAGTAAGCATATATATAACCCTTGGATTGATCCCAAACAAGTAATGCTGATACAAAAATGCATAAATCGAACXXXXXXXXXXCATTATTTTCTAACCTCATTTATTTTACTTTCTAAGTCTTGAAGATAAGCAGAGAAAGGTATAAAACAGAATAAAAGTGT

GAAGCACATAGTAAGCATATATATAACCCTTGGATTGATCCCAAACAAGTAATGCTGATACAAAAATGCATAAATCGAACXXXXXXXXXXCATTATTTTCTAACCTCATTTATTTTACTTTCTAAGTCTTGAAGATAAGCAGAGAAAGGTATAAAACAGAATTAAAGTGT

>Marker83113

AAATAAAAGTTAAAATCACGCACACAATAAAGATAAAATAATCATCATAAGATATCAAGAGAAGAAGTCATAAGTAGTGCXXXXXXXXXXAAATATATGTATCTCTATTGTTATATATAAATAAAGTAGGGACCCTTGCAAAAATAGCAAAAAAAAATTTATGATAATAG

AAATAAAAGTTAAAATCACGCACACAATAAAGATAAAATAATCATCATAAGATATCAAGAGAAGAAGTCACAAGTAGTGCXXXXXXXXXXAAATATATGTATCTCTATTGTTATATATAAATAAAGTAGGGACCCTTGCAAAAATAGCAAAAAAAAATTTATGATAATAG

>Marker83610

CCATCTAATTTTAGTGATAATATGGAATAACCCATGGTGTTGGATGCTAGATTTTGAAGGTAGTTATGATTTTGTCAATTXXXXXXXXXXGGATCACGTGTGAGCTATTATCGGTCGTCTATTTGGTATCCTACCATAGTCGAATTGGATGAAGGTTGTTACTGTGGCAT

CCATCTAATTTTAGTGATAATATGGAATAACCCATGATGTTGGATGCTAGATTTTGAAGGTAGTTATGATTTTGTCAATTXXXXXXXXXXGGATCACGTGTGAGCTATTATCGGTCGTCTATTTGGTATCCTACCATAGTCGAACTGGATGAAGGTTGTTACTATGGCAT

>Marker83639

ATATGGTTGCTGGCTTTAGCATGCCTTTTGTAGAAGGCTTTCATCCTTGCCACGCCATCTTCAATGGAACTACGCTCCATXXXXXXXXXXGTGAGAGTATATGGACAAAAACTCTTTACATATTCTACCAGTATAAATCTTTTCGATTTTGTTTTCTTTTTCACCTAAAA

ATATGGTTGCTGCCTTTAGCATGCCTTTTGTAGAAGGCTTTCATCCTTGCCACGCCATCTTCAATGGAACTACGCTCCATXXXXXXXXXXGTGAGAGTATATGGACAAAAACTCTTTACATATTCTACCAGTATAAATCTTTTCGATTTTGTTTTCTTTTTCACCTAAAA

>Marker83814

TGAACCATGAGAACTGGCAGGAAAACCTAATTACATCGATAAACTCCATCAGCCTCCAGCTTATGACGTAAATTCATAACXXXXXXXXXXGCTAATAATGATTGATTTGGAACTAACACATCTAGTAACCAGAACCAATAGGCACAAAGTCAACTAGGAAACAAGTTCAA

TGAACCATGAGAACTGGCAGGAAAACATAATTACATCGATAAACTCCATCAGCCTCCAGCTTATGACGTAAATTCATAACXXXXXXXXXXGCTAATAATGATTGATTTGGAACTAACACATCGAGTAACCAGAACCAATAGGCACAAAGTCAACTAGGAAACAAGTTCAA

>Marker83927

TTCAGTAGGCAACAGCCATGCCATAAAGAAATCACCTGTGCAACACAAAACTTACATCATTCACTAACATAGAATGCTCGXXXXXXXXXXCGTTGCCATATGACTTTTATCATTTAATGTTCATTCATCAAATGTTCGTCTCTAACACACAAATCATGCTCATGTTATTA

TTCAGTAGGCAACAGCCATGCCATAAAGAAATCACCTGTGCAACACAAAACTTACATCATTCACTAACATAGAATGCTAGXXXXXXXXXXCGTTGCCATATGACTTTTATCATTTAATGTTCATTCATCAAATGTTCGTCTCTAACACACAAATCATGCTCATATTATTA

>Marker83979

TACCCAATATAATAATTTAACTACTTTTCCAAATAGCCAAAGGTATAAACTAGTGTTCCAAGTGAACAATCAGTGAGTTGXXXXXXXXXXACGAAGGAGTTGAAAATGAAGAGGACGAGGATAATGAAACCCTTTTAGCCATACAAAATCTAAGAGGGAGGAGAGTGTTA

TACCCAATATAATAATTTAACTACTTTTCCAAATAGCCAAAGGTATAAACGAGTGTTCCAAGTGAACAATCAGTGAGTTGXXXXXXXXXXACGAAGGAGTTGAAAATGAAGAGGACGAGGATAATGAAACCCTTTTAGCCATACAAAATCTAAGAGGGAGGAGAGTGTTA

>Marker84039

TTTGTAGATCTTGAACAGTGTATTAAGCTCTCTCTCTCTCTCTATATATATATACCAAAAAGAAAAAGGCTCATGTGATCXXXXXXXXXXGATGCTTCAGCTCGTGGGCTTATTCTCGATCCTGGGCGGGTGACGAAGTGGGATGCCTTTGCCATGGGTTGACTCACTTA

TTTGTAGATCTTGAACAGTGTATTAAGCTCTCTCTCTCTCTATATATATATATACCAAAAAGAAAAAGGCTCATGTGATCXXXXXXXXXXGATGCTTCAGCTCGTGGGCTTATTCTCGATCCTGGGCGGGTGACGAAGTGGGATGCCTTTGCCATGGGTTGACTCACTTA

>Marker84318

TGAATAAAATTATATCTCACGAGTAAGGGGGAGGCAAAATGTTTAAGCTGTTGAAAGTCGTTTGAATTGTCCTATTTCAAXXXXXXXXXXTTCTGTTATTTCCTATAAAGTTGATATCATAAACATCATAGGAACTGAGCTTGCAAATGCAAATAAGTCGCTGTAATCTA

TGAATAAAATTATATCTCACGAGTAAGGGGGAGGCAAAATGTTTAAGCTGTTGTAAGTCGTTTGAATTGTCCTATTTCAAXXXXXXXXXXTTCTGTTATTTCCTATAAAGTTGATATCATAAACATCATAGGAACTGAGCTTGCAAATGCAAATAAGTCGCTGTAATCTA

>Marker84760

CTAGAGTTAGGCTTCTGTTGGCTTTGGCACGAGTAGTAAGTTATCACCTGCTAAATAATAATATGGCATGAGTATAAGTTXXXXXXXXXXGGCTAAAATTAGTTTTTTTTCCTCTCCTCTCGCAAGAGGCATTATGTTGAATCTTATAACATATGTATTGTGCTGAGCTT

CTAGAGTTAGGCTTCTGTTGGCTTTGGCACGAGTAGTAAGTTATTACCTGCTAAATAATAATATGGCATGAGTATAAGTTXXXXXXXXXXGGCTAAAATTAGTTTTTTTTCCTCTCCTCTCGCAAGAGGCATTATGTTGAATCTTATAACATATGTATTGTGCTGAGCTT

>Marker84776

AGATCTTTAATCAATTTTCTATGGCTTCATTTCCTCATAATTTCTACCCTGACCTTCCTTATCCTAACAATTTTTCTGACXXXXXXXXXXCTTCCTAACAATGCAGCATCCTGACCTATACGGTCCAGTCTCCGATATGACCGTTCCATCGTTGCCCGAGTTTAACATGA

AGATCTTTAATCAATTTTCTATGGCTTCATTTCCTCATAATTTCTACCCTGACCTTCCTTATCCTAACAATTTTTCTGACXXXXXXXXXXCTTCCTAACAATGCAGCATCCTGACCTATACGGTCCAGTCTCCGATATGACCGTTCCGTCGTTGCCCGAGTTTAACATGA

>Marker84793

CTTTTAATAAGAATGCAGTAAATGAGCTGCAAAGCCAAAAACTAATGAACAGGGATATAACATCGTTAAAAAATGAAAAAXXXXXXXXXXATATTTTTCAACTTTTTTTAGAGTCGATTGGAGTAATTTCTTAATTAACATGACCTTTTGTATATATGCTTGGCAAAACA

CTTTTAATAAGAATGCAGTAAATGAGCTGCAAACCCAAAAACTAATGAACAGGGATATAACATCGTTAAAAAATGAGAAAXXXXXXXXXXATATTTTTCAACTTTTTTTAGAGTCGATTGGAGTAATTTCTTAATTAACATGACCTTCTGTATATATGCTTGGCAAAACA

>Marker84959

AACTCCTACCAATGCGTCTGAGGTAAGATTCTTTCATGATTTGGCTAGTTTCTATAGGAGATTTATTAAAGATTTTAGTAXXXXXXXXXXGGAGCTCAATTCAACTACTCGACTTATGACAAGGAGCTACATGCGCTTCTGAGGGCTTTGAAAGTTTGGCAACATTACTT

AACTCCTACCAATGCGTCTGAGGTAAGATTCTTTCATGATTTGGCTAGTTTCTATAGGAGATTTATTAACGATTTTAGTAXXXXXXXXXXGGAGCTCAATTCAACTACTCGACTTATGACAAGGAGCTACATGCGCTTCTGAGGGCTTTGAAAGTTTGGCAACATTACTT

>Marker84988

AAAGGGGGTTTGGGGTTTGATTGGTTGGTTTTCTTGGCATTGGTAAAGAGTTGCCATTGAATGGGAAGTTTGTTCTGTATXXXXXXXXXXTGATTGGACATGCAGATCATGGAGAATCAAATTCACTTTTCTACTACATTCAAACAAGTTGGTTCTTCCAGCGAATATAT

AAAGGGGGTTTGGGGTTTGATTGGTTGGTTTTCTTGGCATTGGTAAAGAGTTGCCATTGAATGGGAAGTTTGTTCTGTATXXXXXXXXXXTGATTGGACATGCATATCATGGAGAATCAAATTCACTTTTCTACTACATTCAAACAAGTTGGTTCTTCCAGCGAATATAT

>Marker84994

TGAACTGATTAAAAATTGTAAAGAATACAAATAAGATAATTTAGAGATCATAATTAGCAATGAGAAAAAAGAAGGAAAAAXXXXXXXXXXTACACATTGGTAGGTATAGTGACGGTTTGACGCTCGGATTCGAAAACACGGTTTCGATAGATCCTATCAATGTAATCTTT

TGAACTGATTAAAAATTGTAAAGAATACAAATAAGATAATTTAGAGATCATAATTAGCAATGAGAAAAAAGAAGGAAAAGXXXXXXXXXXTACACATTGGTAGGTATAGTGACGGTTTGACGCTCGGATTCGAAAACACGGTTTCGATAGATCCTATCAATGTAATCTTT

>Marker85069

TCAGTCATGAGTTTTGCACAAGATGAAGTTAATTGGTTCAGGTGGAGCAATAACCAGAAGAAGGTAACGTTGTGGGAGGAXXXXXXXXXXGTTAGCCGTCACCCAAAGCCATAAGAAGAATGCATGAAGGGAGCCCAATTAGTGAATGACCATAACATATCCCTAAAGTT

TCAGTCATGAGTTTTGCACAAGATGAAGTTAATTGGTTCAGGTGGAGCAATAACCAGAAGAAGGTAACGTTGTGGGAGGAXXXXXXXXXXGTTAGCCGTCTCCCAAAGTCATAAGAAGAATGCATGAAGGGAGCCCAATTAGTGAATGACCATAACGTATCCCTAAAGTT

>Marker85173

AATCCTTTTTGACATTATGATTAAATGAAATCCAAATAATTCAATACTTCCTCGTAAAATAAAATACAAACCATTCTTATXXXXXXXXXXTTACACACAAAATAGAACATCGTCTCTGATACAAGACTCCATAATAGGAAAACCAAAACCAACAAGTCTAATGACAAGGT

AATCCTTTTTGACATTATGATTAAATGAAATCCAAATAATTCAATACTTTCTCGTAAAATAAAATACAAACCATTCTTATXXXXXXXXXXTTACACACAAAATAGAACATCGTCTCTGATACAAGACTCCATAATAGGAAAACCAAAACCCACAAGTCTAATGACAAGGT

>Marker85274

TTTTTCGATTATTAACATTCTCAGTCCAATCATTATCATGATAACCTTCAAGCTCGAATTCCTTAGATGAATAATAAAATXXXXXXXXXXTTGGACAGTTTTATCCCAATTTATATCATAGTTGTGACCGTTTAGGATTGATTATCTTGAACTTCTCTAGTGTTTCTCTA

TTTTTCGATTATTAACATTCTCAGTCCTATCACTATCATGATAACCTTCAAGCTCGAATTCCTTAGATGAATAATAAAATXXXXXXXXXXTTGGACAGTTTTATCCCAATTTATATCATAGTTGTGACCGTTTAGGATTGATTATCTTGAACTTCTCTAGTGTTTCTCTA

>Marker85320

ATTTGATTCCTTCAAGGGAGATTGGGGTTCTTCCTTACTTGAGCCCAAATCTTTATCTTCTGATTCTTCAGTCAATGGCTXXXXXXXXXXATGGGCTGATCAGCTTCCGGTTTGGCTGTTGATGAAATGGGGCTATTCCGAAGGCTGAAAGTAGCGGCGTTCTCGTCGTC

ATTTGATTCCTTCAAGGGAGATTGGGGTTCTTCCTTACTTGAGCCCAAATCTTTATCTTCTGATTCTTCAGTCAATGGCTXXXXXXXXXXATGGGCTGATCAGCCTCCGGTTTGGCTGTTGATGAAATGGGGCTATTCCGAAGGCTGAAAGTAGCGGCGTTCTCGTCGTC

>Marker85451

CTGCACCATCACTGTCAAAACAAATACCTTTCTGTTTATCTACTGAACCTGGTGAAGAAAGCGATGATTTTGAAAATGAGXXXXXXXXXXATTTCTTGTGTGATTCTTCAATGGAGCCCTCATCCATTTGCTTCTTCGATGTCTCACTTTTCTTCTTATCTTTTATTCTT

CTGCACCATCACTGTCAAAACAAATACCTTTCTGTTTATCTACTGAACCTGGTGAAGAAAGCGATGATTTTGAAAATGAGXXXXXXXXXXATTTCTTGTGTGATTCTTCAATGGAGCCCTCATCCATTTGCTTCTTGGATGTCTCACTTTTCTTCTTATCTTTTATTCTT

>Marker85477

ATTTTGTTTTAATTATGCAAATGAAAACCTTGATTTTTATATTTTTCGAAGTTTTTGTTTCAAAATTCTCATTGGCTAGCXXXXXXXXXXTACCTTGCTAAATACTAATATGCCCTCCTAATAGTCATATACTAATATTCTCCTAATAATCCTATTATTTCCCAAACTAG

ATTTTGTTTTAATTATGCAAATGAAAACCTGGATTTTTATATTTTTCGAAGTTTTTGTTTCAAAATTCTCATTGGCTAGCXXXXXXXXXXTACCTTGCTAAATACTAATATGCCCTCCTAATAGTCATATGCTAATATTCTCCTAATAATCCTATTATTTCCCAAACTAG

>Marker85643

TTTGCACTTTCAACCTAATCAAACACAAATGGATTCATCATCAAAAGCATTAACCATACTTGTCTGTATCTTTTAAAACAXXXXXXXXXXAATATTTCAAATAATTCTAATTGTGTATGATCAATAAAACATGCTATAATCAATCAAATTATGCAAGATAGAAAAATATA

TTTGCACTTTCAACCTAATCAAACACAAATGGATTCATCATCAAAAGCATTAACCATACTTGTCTGTATCTTTTAAAACAXXXXXXXXXXAATATTTCAAATAATTCTAACTGTGTATGATCAATAAAACATGCTATAATCAATCAAATTATGCAAGATAGAAAAATATA

>Marker85780

TATGGACAATGCAACAAAGTTAAGTAAGGTGTAGACGAGCTAAGTTTGAAGATAGGCATGTAATCTCGTATCTTCTATATXXXXXXXXXXAAATCCCAGTGCTAAGGCAGGGGAATCTTGGTGCTTGATTACTGAGTTGAAAAATAGAAAGGCTTTAAAGATGGGTTTTA

TATGGACAATGCAACAAAGTTAAGTAAGGTGTAGACGAGCTAAGTTTGAAGATAGACATGTAATCTCGTATCTTCTATATXXXXXXXXXXAAATCCCAGTGCTAAGGCAGGGGAATCTTGGTGCTTGATTACTGAGTTGAAAAATAGAAAGGCTTTAAAGATGGGTTTTA

>Marker85806

ATGTGCCTAATTACACTTTCATTTCAACAATAATACAATTTCTTTAGTTTTTCACCTAACCTTTTATTGGTCTTCTGTTTXXXXXXXXXXTAGATCGTCTCAAATACAATTGTTCCAAACTAAACACACTTAATTGCATAATTCCTATGTTTAAGTCACCGAAAAGGAAG

ATGTGCCTAATTACACTTTCATTTCAACAATAATACAATTTCTTTAGTTTTTCACCTAACCTTTTATTGGTCTTCTGTTTXXXXXXXXXXTAGATCGTCTCTAATACAATTGTTCCAAACTAAACACACTTAATTGCAGAATTCCTATGTTTAAGTCACCGAAAAGGAAG

>Marker85900

CGAAGAATTTTCTACTTGATTTAAATTTCCATAAATGTCGGTTTATGATTCTATTCTTATTTCTGGTGTTGCAGGTGCCGXXXXXXXXXXGAACTGCTGTCGATTGGGTCCAGATGCCTGGGATGAAGGTGTTAATGCGTTCTTTCAGTTCGATAGATTTTACTGCTATT

CGAAGAATTTTCTACTTGATTTAAATTTCCATAAATGTCGGTTTATGATTCTATTGTTATTTCTGGTGTTGCAGGTGCCGXXXXXXXXXXGAACTGCTGTCGATTGGGTCCAGATGCCTGGGATGAAGGTGTTAATGCGTTCTTTCAGTTCGATAGATTTTACTGCTATT

>Marker85980

TATAGTCAAACACTTCTACCTGAGTAATTTTAATTAAATGACTTTGAGCTAAAAATGCCTATTCCAAAATCACTCCCAAGXXXXXXXXXXTTGTTGTGCATAGGTTAGTAAGCTATTAGGTGCTTGCATTTTAATTCTTCATTTAGCTGGAACTCTTTTGGCTTAGGTGA

TATAGTCAAACACTTCTACCTGAGTAATTTTAATTAAATGACTTTGAGCTAAAAATGCCTATTCCAAAATCACTCCAAGCXXXXXXXXXXTTGTTGTGCATAGGTTAGTAAGCTATTAGGTGCTTGCATTTTAATTCTTCATTTAGCTGGAACTCTTTTGGCTTAGGTGA

>Marker86059

AAGAGAATTATACTAAGAGCAAAGTAAGAAAAGACAAAAACATCCAAACAAACTGCCTGAAGATGATCTCAGCCAATACGXXXXXXXXXXAAAGCTATCTTGCCAAAGTAGTCCAAAACCCAGAAGAGGAACAAAAAAATCTTTCAAAATATCAGCATCAGCAAAAGGTT

AAGAGAATTATACTAAGAGCAAAGTAAGAAAAGACAAAAACATCCAAACAAACTGCCTGAAGATGATCTCAGCCAATACAXXXXXXXXXXAAAGCTATCTTGCCAAAGTAGTCCAAAACCCAGAAGAGGAACAAAAAAATCTTTCAAAATATCAGCATCAGCAAAAGGTT

>Marker86075

AAAAATGACCTGCTTACCTATTAAAAGATATCCTTTACTTTTGAAAGAACTAAAAAATGTCTCAATTTGATTCTCTCTCTXXXXXXXXXXGTCCTACGGATAAATCCTCCGGACCCTAGAAAGTTGATTTACCATAAATGAAAAGGAAAGACAAAGATGACATACCTCAT

AAAAATGACCTGCTTACCTATTAAAAGATATCCTTTACTTTTGAAAGAACTAAAAAATGTCTCAATTTGATTCTCTCTCTXXXXXXXXXXGTCCTACGGATAAATCCTCCGGACCCTAGAAAGTTGATTTAGCATAAATGAAAAGGAAAGACAAAGATGACATACCTCAT

>Marker86184

TTGCTTGCAAAATTGTGGTTTGAACGGAATCAAAAAAAATTTCATGACAAAAAGTCAAGCTGGTTTAACCATTTCGAAACXXXXXXXXXXTCTTTTTGTTTTGGTTGTTTGCTTTGTATCGCTTGCTTTCTTTTAGTCTCTTTTGTTTGAATTTTCTTTGACAGTATCTT

TTGCTTGCAAAATTGTGGTTTGAACGGAATCAAAAAAAATTTCATGACAAAAAGTCAAGCTGGTTTGACCATTTCGAAACXXXXXXXXXXTCTTTTTGTTTTGGTTGTTTGCTTTGTATCGCTTGCTTTCTTTTAGTCTCTTTTGTTTGGATTTTCTTTGACAGTATCTT

>Marker86228

TGTTGCTACACTGGCATGAGCGCCATGTAAGTTGACTCACCACTATTCTATTTAGAAAGCAAGAGAAGATAAGCATAACCXXXXXXXXXXAAGAAAAAATCTCTCAATATATATGTAATATGAGGTCACAGCAACATCTGAAGTTGATTGTTTGATCATCTTCCAAACTC

TGTTGCTACACTGGCATGAGCGCCATGTAAGTTGACTCACCACTATTCTATTTAAAAAGCAAGAGAAGATAAGCATAACCXXXXXXXXXXAAGAAAAAATCTCTCAATATATATGTAATATGAGGTCACAGCAACATCTGAAGTTGATTGTTTGATCATCTTCCAAACTC

>Marker86294

TCTCCGGTCATCGTCTTCTCCGCCGCCGTCTCTGACGCTATCAGACGGCGGTCGGAAACCCCAAGTGGCACCACAACAGCXXXXXXXXXXTGGGTAAAACTTGAAAATGAATTTAAACCGTTGAGGGGAAATGCAAATAGTTAGGATTAGGATGGATTACCTTTAGCCAT

TCTCCGGTCATCGTCTTCTCCGCCGCCGTCTCTGACGCTAACAGACGGCGGTCGGAAACCCCAAGTGGCACCACAACAGCXXXXXXXXXXTGGGTAAAACTTGAAAATGAATTTAAACCGTTGAGGGGAAATGCAAATAGTTAGGATTAGGATGGATTACCTTTAGCCAT

>Marker86467

ACTCCATCTGAAGACAGTGATGTGACCTAAACATCAGTAGGTCAGTGATTGATTGACCCATCTGCCCAATTTAAGTTTGAXXXXXXXXXXAGAATTGGAGAAGTGTCAAGGATTCGAGATGAAAAACGAGCTCATTTTTTATTAAGATTCTAGACGAGCAGGGAGATTTT

ACTCCATCTGAAGACAGTGATGTGACCTAAACATCAGTAGGTCAGTGATTGATTGACCCATCTGCCCAATTTAAGTTCGAXXXXXXXXXXAAAATTGGAGAAGTGTCAAGGATTCGAGATGAAAAACGAGTTCATTTTTTATTAAGATTCTAGACGAGCAGGGAGATTTT

>Marker86521

TCCTAAATATTCAAAACTTTGACTACATGCTGAAGTTCTTTTAAATTTTGCTATTCTATCACCATATATTTTAGCAACAAXXXXXXXXXXTCTCTCTCGCAAATATTATATGCCCAGTTTCTCACTTCCCTAATGTTTATCCTTGCAAACTGGTTAAGAGTTGAAAAAAG

TCCTAAATATTCACAACTTTGACTACATGCTGAAATTCTTTTAAATTTTGCTATTCTATCACCATATATTTTAGCAACAAXXXXXXXXXXTCTCTCTCTCAAATATTATATGCCCAGTTTCTCACTTCTCTAATGTTTATCCTTGCAAACTGGTTAAGAGTTGAAAAAAG

>Marker86538

TAAAAGAAGGTTAGTTTATCTAACATATGGGTCATCTAAACCCTCCATATTCTCTTAATCAAAGTATTGCTAATGGTTTTXXXXXXXXXXAATCCCACAGTGGGTTTAAGAAGAACTCCTCGTGCTGCCAAACAAGGAGTAGGTTACAACTATACTCCCCCAAATCCTTG

TAAAAGAAGGTTAGTTTATCTAACATATGGGTCATCTAAACCCTCCATATTCTCTTAATCAAAGAATTGCTAATGGTTTTXXXXXXXXXXAATCCCACAGTGGGTTTAAGAAGAACTCCTCGTGCTGCCAAACAAGGAGTAGGTTACAACTATACTCCCCCAAATCCTTG

>Marker86684

TCACATTTTTAAATTTTCCATATTGAAATTTATATTTCAACAAATACACAGTAATTCTTTAATTATATTAGAATAATAAAXXXXXXXXXXAAATTCCATCGCTACAAGTTTATGATCAAGTGGATGTTCTTCGACATTGATGAGTTCATCTACATGTTGTTGCTTTCAAA

TCACATTTTTAAATTTTCCATATTGAAATTTATATTTCAACAAATACACAGTAATTCTTTAATTATATTAGAATAATAAAXXXXXXXXXXAAATTCCATCGCTACAAGTTTATGATCAAGTGGATGTTCTTCGACATTGATGAGTTCATCTACATGTTGTCGCTTTCAAA

>Marker86701

AAATCTAATAAAGCATCCTAATAAAATAAGTGAAATTGCATCAAATGACAAAAACATTTAAGAAAAAACAGCTCATGACAXXXXXXXXXXGGGTCCCATAAAATAACTTGATGTGATAAATTATTGATCTACATACATAGCTGAAAGAAGTTTTCGAGGAGGGGTTGTAA

AAATCTAATAAAGCATCCTAATAAAATAAGTGAAATTGCATCAAATGACAAAAACATTTAAGAAAAAACAGCTCATGACAXXXXXXXXXXGGGTCCCATAAAATAACTTGATGTGATAAATTATTGATCTACATACATAGTTGAAAGAAGTTTTCGAGGAGGGGTTGTAA

>Marker86705

TTTATTATGAATCACTTTTAAGACGAGTAGGACGGGAGAAAAAGAAAGAAAAAGAAGGGAGAGGAGGAGGGGGGGTCCATXXXXXXXXXXAGTTTAAGTTTAACTTTATAGTGTGTTGTGTATGTGGGTTGGGGAGTGGGCATAAACAGCAACATGGAATACCGAGTTGG

TTTATTATGGATCACTTTTAAGACGAGTAGGACGGGAGAAAAAGAAAGAAAAAGAAGGGAGAGGAGGAGGGGGGGTCCATXXXXXXXXXXAGTTTAAGTTTAACTTTATAGTGTGTTGTGTATGTGGGTTGGGGAGTGGGCATAAACAGCAACATGGAATACCGAGTTGG

>Marker86787

CTTCTAATATGTCATGTGCCCAACCATTATTTGAGAGATCAAAATTAAAATGCAAATAATCCTAAATAGTAAATAATGTAXXXXXXXXXXTTTACAAATATCATAATGATATAATATAAATAGGGCTATTCATATACCAATGTGAACATTTACTTAATTTATGTTAAAAA

CTTATAATATGTCATGTGCCCAACCATTATTTGAGAGATCAAAATTAAAATGCAAATAATCCTAAATCGTAAATAATGTAXXXXXXXXXXTTTACAAATATCATAATGATATAATATAAATAGGACTATTCATATACCAACGTGAACATTTACTTAATTTATGTTAAAAA

>Marker86812

GAGTCTCTTAGACTAGAGATTTCAAAGCACGATTGTTTCCCGAATAATCACACATACTCAATTCTCATTTGTGGTATGTGXXXXXXXXXXCAGGGCACCGATAAGGTTTTTGATATTGCCAGTCTCCAAGTTATGATGGAGCGATTATGTGAGTCGGGAATGATTCTTAA

GAGTCTCTTAGACTAGAGATTTCAAACCACGATTGTTTCCCGAATAATCACACATACTCAATTCTCATTTGTGGTATGTGXXXXXXXXXXCAGGGCACCGATAAGGTTTTTGATATTGCCAGTCTCCAAGTTATGATGGAGCGATTATGTGAGTCGGGAATGATTCTTAA

>Marker86906

ATTTTCTAAAAAAATGTAATTTTGCTTTTATCGGCTCAATCTCGGCTTAGAAGCTTTACAAAGTGGTTTGATGAGGTCATXXXXXXXXXXACCATCTTGGGACAACTCTGGTTCGAGATATTGACATATTTGCTTATGTTTTTTAGCTTGGTGCATCTCAGGGTCATGAT

ATTTTCTAAAAAAATGTAATTTTGCTTTTATCGGCTCAATCTCGGCTTAGAAGCTTTACAAAGTGGTTTGATGAGGTCATXXXXXXXXXXACCATCTTGGGACAACTCTGGTTCGGGATATTGACATATTTGCTTATGTTTTTTAGCTTGGTGCATCTCGGGGTCATAAT

>Marker87131

TAGTATACATGGAAAATGACAATGCTTGCAAGATAGTAGGCACTGGGTCTGTTACACTTAAGTTCAAAGATGGGACAACAXXXXXXXXXXAATATTTCATCTTATAATCTATTTTTTGTTCAATATTTAGTAGTTTTCATTTTAACATATTATTACGTAATAATAGGTTT

TAGTATACATGGAAAATGACAATGCTTGTAAGATAGTAGGCACTGGGTCTGTTACACTTAAGTTCAATGATGGGACAACAXXXXXXXXXXAATATTTCATCTTATAATCTATTTTTTGTTCAATATTTAGTAGTTTTCATTTTAACATATTATTACGTAATAATAGGTTT

>Marker87207

GTCTTATTCCACGCATGCATGATAAATTGTAATCAAGTTAATAGAAGACTTAGTTGTTGTTGTCTGTAATGGACTAGTATXXXXXXXXXXGATAAGAGTCACAAGTGAAGTAGGGTTGAAATGCATTGTTAAAAGAAGAACGATGTCGATCGACTTCATGCCACATCTCG

GTCTTATTCCACGCATGCATGATAAATTGTAATCAAGTTAATAGAAGACTTAGTTGTTGTTGTCTGTAATAGACTAGTATXXXXXXXXXXGATAAGAGTCACAAGTGAAGTAGGGTTGAAATGCATTGTTAAAAGAAGAACGATGTCGATCGACTTCATACCACATCTCG

>Marker87295

AAAAGTTAATCAAAAGGAATAGTCAAGTCGTATTTAAATTTCAAGGAAAGAAGAAGCATCAAAAGCCAAACCAAAAAGTCXXXXXXXXXXCACAATGGGATTGTTTGGAGGATCAACCTAAGCATCAACTTAATTAAAACAGTTTCAAAAGCATCACAACAGTTTTAGAA

AAAAGTTAATCAAAAGGAATAGTGAAGTCGTATTTAAATTTCAAGGAAAGAAGAAGCATCAAAAGCCTAACCAAAAAGTCXXXXXXXXXXCACAATGGGATTGTTTGGAGGATCAACCTAAGCATCGACTTAATTAAAACAGTTTCAAAAGCATCACAACAGTTTTAGAA

>Marker87369

TTTAATTTTAATTTTATTATTATGTAATGAGTAATTTCAAATTAAGTTAAATAAATTCATAAATATGTAAAATCCGATTAXXXXXXXXXXTGAAAAAAAAGAAAAAAAATAATAGACCGACACGTGTGAGAAAAGCAAGCAAGAAAGTCGGTGGTGTCACGGTTATAGAA

TTTAATTTTAATTTTATTATTATGTAATGAGTAATTTCACATTAAATTAAATAAATTCATAAATATGTAAAATCCGATTAXXXXXXXXXXTGAAAAAAAAGAAAAAAAAGAATAGACCGACACGTGTGAGGAAAGCAAGCAAGAAAGTCGGTGGTGTCACGGTTATAGAA

>Marker87691

TCTAAATTAGGGATAAAGGAGCTTGTTCCTCCATTTTTAGATCCTAAATTGTGGACAACGATGTGAAAACCGAGTCGGTTXXXXXXXXXXGAACCAATGACGTAAACATCAATTTCTATGACCTTCCCATAAGGCAGCTACAGTATAATATTAGTGGTTACCAAGATTTT

TCTAAGTTAGGGATAAAGGAGCTTGTTCCTCCATTTTTAGATCCTAAATTGTGGACGGCGATGTGAAAACCGAGTCGGTTXXXXXXXXXXGAACCAATGACGTAAACATCAATTTCTATGACCTTCCCATAAGGCAGCTACAGTATAATATTAGTGGTTACCAAGATTTT

>Marker88088

TATTTTTAATATCTTTTATCATCTGTGACTTGTGATGCAGTCGTCATGAGCATGCCGGATAAATATTTTGGTTTGTTTTAXXXXXXXXXXCAAAGAACGACGCTTCTTGGTTTGTAGTAATGCTGGGAGACCATCTAAGTGCTCAGCTGCTATGTTTAATGGTGATCAAT

TATTTTTAATATCTTTTATCATCTGTGACTTGTGATGCAGTCGTCATGAGCATGCCGGATAAATATTTTCGTTTGTTTTAXXXXXXXXXXCAAAGAACGACGCTTCTTGGTTTGTAGTAATGCTGGGAGACCATCTAAGTGCTCAGCTGCTATGTTTAATGGTGATCAAT

>Marker88305

CATGGTATCAGTGTCCAAGCTTCCTTAAATATTGATTTTGGGTCCTTCATAGGAGACCAAATAATTGTTATTTTCCTCTTXXXXXXXXXXCAACTTCTCAGTCAAATTGAAAATTGTGATATTCTAGGCGCGTCGTCATTGTCAAAAGCAAATGAGCGAGACAACCTAAG

CATGGTATCAGTGTCCAAGCTTCCTTAAATATTGATTTTGGGTCCTTCATAGGAGACCAAATAATTGTTATTATCCTCTTXXXXXXXXXXCAACTTCTCAGTCAAATTGAAAATTGTGATATTCTAGGGGCGTCGTCATTGTCAAAAGCAAATGAGCGAGACAACCTAAG

>Marker88410

TTCCCGCCGAGTTTGGGAACTTGACTAATCTACATGTATTAAACGTGGCGGAGAATCGTCTATCCGGTGTCATCTCCAGCXXXXXXXXXXACGTTGCCTTCAGCTCTCGCGAATTGTTCTTCGCTTGTCCATTTGAGTGTTGAGGGAAATGCTCTTCAAGGGGTGATTCC

TTCCCGCCGAGTTTGGGAACTTGACTAATCTACATGTATTAAACGTGGCGGAGAATCGTCTCTCCGGTGTCATCTCCAGCXXXXXXXXXXACGTTGCCTTCAGCTCTCGCGAATTGTTCTTCGCTTGTCCATTTGAGTGTTGAGGGAAATGCTCTTCAAGGGGTGATTCC

>Marker88585

GTAATACAAATCCTTTATAAGCAGAATTAAAGGTCACCAAAACAAGGGGAAGAAAACTTTAACAGTTATAATAAGACTTAXXXXXXXXXXGTAGGACCAACAGCAGGCGTGGAAATATTAGTTCCAACGATCTCTATCTTCATTGGTTTTCCATCAAGCTGAACGTTGTT

GTAATACAAATCCTTTATAAGCAGAATTAAAGGTCACCAAAACAAGGGGAAGAAAACTTTAACAGTTAAAATAAGACTTAXXXXXXXXXXGTAGGACCAACAGCAGGCGTGGAAATATTAGTTCCAACGATCTCTATCTTCATTGGTTTTCCATCAAGCTGAACGTTGTT

>Marker88644

AATATTACAAACCCCATTGTTGTCTTTCAAATTCTAAGCATAATGAAAAGAATGAAGTTCAAATGGAAACTTAATTCATTXXXXXXXXXXTCAATGCGAAAATGGTCGGTGATTGATATTTTTTTTCAAGGACAAGCTTTGATGAGAAGATAAGTAAAGTAACTAAGTAA

AATATTACAAACCCCATTGTTGTCTTTCAAATTCTAAGCATAAAGAAAAGAATGAAGTTCAAATGGAAACTTAATCCATTXXXXXXXXXXTCAATGCGAAAATGGTCGGTGATTGATATTTTTTTTCAAGGACAAGCTTTGATGAGAAGATAAGTAAAGTAACTAAGTAA

>Marker88831

TTCTTTTCTCATACCTTTGTTTATTTATGAGAATTTATCTTTTTCATTAAGAGAAAATGAAAGAATAAAAAAGGAGGCGAXXXXXXXXXXACTTTAGAATAAAGTTTTTATTTTATTTTATATCTATTACTGGTTAAAACCACTTTGCATCTGCATGCGCCATACAAAGA

TTCTTTTCTCATACCTTTGTTTATTTATGAGAATTTATCTTTTTCATTGAGAGAAAATGAAAGAATAAAAAAGGAGGCGAXXXXXXXXXXACTTTAGAATAAAGTTTTTATTTTATTTTATATCTATTACTGGTTAAAACCACTTTGCATCTGCATGCGCCATACAAAGA

>Marker89015

TGTGTTTGGTGTTGAGAAGCATTATTTTTACCTACCATTTCTTTCTAGCGTGATGCTAAATTGATAACAATACTAAGTATXXXXXXXXXXACTGGTTCCTCCACTGGTAATATATTAATGTTGGAAACTGATCAAAGGAAACTGTAGGAGAAAGGTTAGAACAGTTGAAG

TGTGTTTGGTGTTGAGAAGCATTATTTTTTCCTACCATTTCTTTCTAGGGTGATGCTAAATTGATAACAATACTAAGTATXXXXXXXXXXACTGGTTCCTCCACTGGTAATATATTAATGTTGGAAACTGATCAAAGGAAACTGTAGGAGAAAGGTTAGAACAGTTGAAG

>Marker89101

CATTACTTTTTAGAATCTCACGTTTCAAAGAAAAACAATAACCTTACTGCATCGTATATTTTCAAAACTATACCCAAAGGXXXXXXXXXXGCCAACAGCAAACAAACAATTACAAGGCTTCCTTTTCTTTCTATCAACCGCATTATAAATGATAAGGGGGAAAAAAATAA

CATTACTTTTTAGAATCTCACGTTTCAAAGAAAAACAATAACCTTACTGCATCGTATATTTTCAAAACTATACCCAAAGGXXXXXXXXXXGCCAACAGCAAACAAACAATTACAAGGCTTCCTTTTCTTTCTATCAACCGCATTATAAATGATAAGGGGGGAAAAAATAA

>Marker89426

CTTCTCTCCACAGGAAGAGTTAAGAAGGGACTCCTCAATCAACACACCATCTCTATGTCAAGCAGCCGTAATGCCAAATGXXXXXXXXXXATACTTCAATGGAGGAAAATTTTGGTCTCCTAATTATTAAGATAGATCTAAAGTGAGCATAGCTCAACACTAATTGACAG

CTTCTCTTCACAGGAAGAGTTAAGAAGGGACTCCTCAATCAACACACCATCTCTATGTCAAGCAGCCGTAATGCCAAATGXXXXXXXXXXATACTTCAATGGAGGAAAATTTTGGTCTCCTAATTATTAAGATGGATCTAAAGTGAGCATAGCTCAACACTAATTGACAG

>Marker89581

CCTTCCTTCTGACCCTCTCTTTTCCCCTAATTCGTTTACCAAGTTCGATCTGGGGAATGGAGGACAATATAATTGGACCTXXXXXXXXXXCTAAACAACAGATTTAAGTTTCATAACAATCAAAGAATTATGACCTTACCGTCCTTTGAAACTCAGATGACAGAGATATA

CCTTCCTTCTGACCCTCTCTTTTCCCCTAATTCGTTTACCAAGTTCGATCTGGGGAATGGAGGACAATATAATTGGACCTXXXXXXXXXXCTAAACAACAGATTCAAGTTTCATAACAATCAAAGAATTATGACCTTACCGTCCTTTGAAACTCAGATGACAGAGATATA

>Marker89696

TTAAAGCACGGACATTTTAGAACTTCACTCGAATAATGTTGTGTGTTCAATACTACTAGAGGTAAAGTTCAAAACCAAGTXXXXXXXXXXGGAAGTAGTTGAGTCTTCATCAACTTAAAAAATGTGAAAGTCTCCATTATTTCATCTCCTTAAAATAGTGGGTAATTTAT

TTAAAGCACGAACATTTTAGAACTTCACTCGAATAATGTTGTGTGTTCAATGCTACTAGAGGTAAAGTTCAAAACCAAGTXXXXXXXXXXGGAAGTAGTTGAGTCTTGATCAACTTAAAAAATGTGAAAGTCTCCATTATTTCATCTCCTTAAAATAGTGGGTAATTTAT

>Marker89931

TAGATTTTGAAAGATTTAATCAAATGACAGAAACTAAATGCAACAAAACAATTAAAATAGAACATAATGAGAATGACATGXXXXXXXXXXAGAAGCTCCTTCAAAATCTTCAAGTTACCTCCTCTGGCGGCGGCATGAACACCTCTGTTCATCATTTCCCATTTATAAAC

TAGATTTTGAAAGATTTAATCAAATGACAGAAACTAAATGCAACTAAACAATTAAAATAGAACATAATGAGAATTACATGXXXXXXXXXXAGAAGCTCCTTCAAAATCTTCAAGTTACCTCCTCTGGCGGCGGCATGAACACCTCTGTTCATCATTTCCCATTTATAAAC

>Marker90011

CGTCCTACTTACCGAGCATGACGTTATTGTCTGTCAGGTTGCATCGTCTGTAGAGAGAATATGCTTTAGTTGGAAACTTAXXXXXXXXXXACATATGAATATGATCTCTCTCTCTTTTTAGTTTTTGGTTTTGTAATGGAAACACTGACAGTGGTTGATGACTACAGGGT

CGTCCTACTTACCGAGCATGACGTTATTGTCTGTCAGGTTGCATCGTCTGTAGAGAGAATATGCTTTAGTTGGGAACTTAXXXXXXXXXXACATATGAATATGATCTCTCTCTCTTTTTAGTTTTTGGTTTTGTAATGGAAACACTGACAGTGGTTGATGACTACAGGGT

>Marker90056

TTGGGTTTTTTATTTAGATCCTGCACGATTAAGGATCTCAGTAATAGGCTTAAATCCATTCCAAATGCAGTAAAAACACCXXXXXXXXXXACTGCAACTTTAACAAAAAGAGGGAACAATAAAATATTTAAAACTTATTCCCAAATCATTCTCAAAATAATTAACACAAG

TTGGGTTTTTTATTTAAATCCTGCACGATTAAGGATCTCAGTAATAGGCTTAAATCCATTCCAAATGCAGTAAAAACACCXXXXXXXXXXACTGCAACTTTAACAAAAAGAGGGAACAATAAAATATTTAAAACTTATTCCAAAATCATTCTCAAAGTAATTAACACAAG

>Marker90520

TGTCTCAATCATGTCAAATACAGGCATTGCATAGATCTACACATAACAAACACCATATTAATTACATCAAAATCAAATAGXXXXXXXXXXCATCCAGTAGCCGATGATGGCAACGGGGAAGTAGCAAAGGGCAACAACAATGTAGGCAACAATCACACCTCTCCACATGG

TGTCTCAATCATGTCAAATACAGGCATTGCATAGATCTACACATAACAAACACCATATTAATTACATCAAAATCAAATAGXXXXXXXXXXCATCCAGTAGCCGATGATGGCAACGGGGAAGTAGCAAAGGGCAACAACGATGTAGGCAACAATGACACCTCTCCACATGG

>Marker90643

AGAAATTCTCTCCCAGTCCCAAACTGACTTCGTATTGTATGCCTATCGTTCTCATTTTAAAAAAGCCATACAGGAAATTCXXXXXXXXXXGTGAACTATTTTGGTTTCTTAGTTGTAACTACACTATTATGTTTCTATCTAAAGTTTAAACCACCATTGTTGATGGTTCT

AGAAATTCTCTCCCAGTCCCAAACTGACTTTGTATTGTATGGCTATCGTTCTCATTTTAAAAAAGCCATACAGGAAATTCXXXXXXXXXXGTGAACTATTTTGGTTTCTTAGTTGTAACTACACTATTATGTTTCTATTTAAAGTTTAAACCACCATTGTTGATGGTTCT

>Marker90824

TTGTTATCTTATCATCTCGTAGGCTATATATATATATGGTATAATGTTGCATCAGTTACTATGACTATGTTTTTCCTATTXXXXXXXXXXTTAATTAAAGTTGTATTACTCTAAACTAAAATATTGTTGGTAATGACAATGGAACCCTTCATGTCTTCACAAATTGTGTT

TTGTTATCTTATCATCTCGTAGGCTATATATATATATGGTATAATGTTGCATCAGTTACTATGACTATGTTTTTCCTATTXXXXXXXXXXCGAATTAAAGTTGTATTACTCTAAACTAAAATATTGTTGGTAAAGACAATGGAACCCTTCATGTCTTCACAAATTGTGTT

>Marker91240

ATGGTTTAGAAATGATTTAGTTCAATGTTTAAGTTATGATTATGAAAATTTCTTCGAGCATGTTATGATTTAAAGTATTTXXXXXXXXXXGATGTTTATCTCATAGAAACGAGTTAGGGACGCCTACCTCGTGGGAACAGGGATCAGGGATGCTCATCTTTGTTATTTAT

ATGGTTTAGAAATGATTTAGTTCAATGTTTAAGTTATGATTATGAAAATTTCTTCGAGCATGTTATGATTTAAAGTATTTXXXXXXXXXXGATGTTTATCTCGTAGAAACGAGTTAGGGACGCCTACCTCGTGGGAACAGGGATCGGGGATGCTCATCTTTGTTATTTAT

>Marker91404

TTTTCAGCCATCTTTCTTTGAAGAGACACAATCTTTAGCTGCTCCTCGAGTTCTGCAACCTATAGTAAACAAAATATATTXXXXXXXXXXCATGGCAGTTGTGTCCTCCACACCAGGAACACTCCTGATACAACCAATGATCATTAAAACACACTAAAATCAGAGAGAGA

TTTTCAGCCATCTTTCTTTGAAGAGACACAATCTTTAGCTGCTCCTCGAGTTCTGCAACCTATAGTAAACAAAATATATTXXXXXXXXXXCATGGCAGTTGTGTCCTCCACACCAGGAACACTCCTGATGCAACCAATGATCATTAAAACACACTAAAATCAGAGAGAGA

>Marker91680

CGTTTCGCTACTCTGATTGCAATGATTTTTCTACTTTTCTTTATAGGTAAAAACTTTAACGTGTGGAATGGGAGTTATGGXXXXXXXXXXTCGTATTCATGCTCGCTTTCGCATTCTTCATCATCTTTTTCACTGGCTTCCTCGAATTTCCGCTGGCATCCACTTCCATC

CGTCTCGCTACTCTGATTGCAATGATTTTTCTACTTTTCTTTATAGGTAAAAATTTTAACGTGTGGAATGGGAGTTATGGXXXXXXXXXXTCGTATTCATGCTCGCTTTCGCATTCTTCATCATCTTTTTCACTGGCTTCCTCGAATTTCCGCTGGCATCCACTTCCATC

>Marker91689

TAGGATAAGAAATAACTAGGAGGTATGTTTTGTTTTTTTAATTTATGTAAAACTTAAGTTCCTTCCACACAAAATGTATGXXXXXXXXXXATTGTGGCTGAGAAGCTCAAAATCCGTAATCCAATTGACTGTTTATGAAGACAGCTCATTACAAATGGTTACTACGTGGA

TAGGATAAGAAATAACTAGGAGGTATGTTTTGTTTTTTTAATTTATGTAAAACTTAAGTTCCTTCCACACAAAATTTATGXXXXXXXXXXATTGTGGCTGAGAAGCTCAAAATCCGTAATCCAATTGACTGTTTATGAAGACAGCTCATTACAAATGGTTACTACGTGGA

>Marker91767

AGGGATGATTCTGAAAGCTACAACCAAGAAGGTGGAGGCAACTTTGAAGATTCTCCTAAAGCAGATATCTTAGAGGTAGAXXXXXXXXXXGGCATCAATTATCACTTAAGTGGACAACAGGAGCTGGTCCGAGAATTGGTTGTGTAGCTGACTATCCCGTGGAACTTAGA

AGGGATGCTTCTGAAAGCTACAACCAAGAAGGTGGAGGCAACTTTGAAGATTCTCCTAAAGCAGATATCTTAGAGGTAGAXXXXXXXXXXGGCATCAATTATCACTTAAGTGGACAACAGGAGCTGGTCCGAGAATTGGTTGTGTAGCTGACTATCCCGTGGAACTTAGA

>Marker91801

GATTATCGTCTCCTCCATTTCTTGGACAATTCTTTTGTCTTTTCTTAGCAAATGACTTGTCGATATTGGTTTCATTGTAAXXXXXXXXXXTGAGAATGAAAACTTTTAAATCGAAGAAAATGATCGATGGAAATATATACCAGATAAAGCAACCAAATCCTTTGGGGAAA

GGTTATCGTCTCCTCCATTTCTTGGACAATTCCTTTGTCTTTTCTTAGCAAATGACTTGTCGATATTGGTTTCATTGTAAXXXXXXXXXXTGAGAATGAAAACTTTTAAATCGAAGAAAACGATCGATGGAAATATATACCAGATAAAGCAACCAAATCCTTTGGGGAAA

>Marker91940

CAGTCAAGCATAACAAGTTTATCAATAAGGCAATGAATGTATCAAGAAGAATAAATTGTAACAAAAAGTATTAGGTGTATXXXXXXXXXXGGAAATAAATTTCCTTAGTGTTCTAATGTGCCATGTGTAGAGACTAAGTTAAGCATGATTAAGTTAAATCAAGCTTACAC

CAGTCAAGCATAACAAGTTTATCAGTAAGGCAATGAATGTATCAAGAAGAATAAATTGTAACAAAAATTATTAGGTGTATXXXXXXXXXXGGAAATAAATTTCCTTAGTGTTCTAATGTGCCATGTGTAGAGACTAAGTTAAGCATGATTAAGTTAAATCAAGCTTACAT

>Marker92361

TTCCAAACCATGCCCCAAGCACACGGACCTCAAATCAAGTAGGTTGCTGCAGTTTGAAACGAACTAATTCAGTCAAAACAXXXXXXXXXXAGAAAAGATGTAAAACAATTTAAAAAATAATATGAAATTAAAAAAGAGGGGAGAAGATCAAAGCCTCGAGCCATATAAGA

TTCCAAACCATGCCCCAAGCACACGGACCTCAAATCAAGTAGGTTGCTGCAGTTTGAAACGAACTAATTCAGTCAAAACAXXXXXXXXXXAGAAAAGGTGTAAAACAATTTAAAAAATAATATGAAATTAAAAAAGAGGGGAGAAGATCAAAGCCTCGAGCCATATAAGA

>Marker92506

TATAAGATTTGGATATTATTTTTAATTAGTTTTATAGTTTTGGAGTCTATAGGAATGGTATTAGATGCCATTCCATTTTTXXXXXXXXXXACCAATGATTAAGTTTTGTGTAGAAAACCTGTAAAATGTTATCTGTTAATTAACTTGTAAGACACAAGGTATAAAAATGT

TATAAGATTTGGATATTATTTTTAATTAGTTTTATAGTTTTGGAGTCTATAGGAATGGTATTAGATGCCATTCCATTTTTXXXXXXXXXXACCAATGATTAAGTTTTGTGTAGAAAACCTGTGAAATGTTATCTGTTAATTAACTTGTAAGACACAAGGTATAAAAATGT

>Marker92567

CGTGGCACCTAAGAGGAAAACCTATGCTACTCCGCAAATGGATTCCAGGTATTGTCCCTGAAACTTTTGTTTTTGATACTXXXXXXXXXXAGAATAGTTCATTGTGAACGTTACGTATGAGTGGAAGCAGAGAAAGTGTAACGTGTGTCGTTCTTTTGGACACTCTAATG

CGTGGCACCTAAGAGGAAAACCTATGCTACTCCGCAAATGGATTCCAGGTATTGTCCCTGAAACTTTTGTTTTTGATACTXXXXXXXXXXAGAATAGTTCATTGTAAACGTTACGTATGAGTGGAAGCAGAGAAAGTGTAACGTGTGTCGTTCTTTTGGACACTCTAATG

>Marker92691

CAAACACCAAAGAAGTCTACTGAGGAACTGTTAGGAACCTCTAATAACAAGTCTTGAAAGTGTTTAAAATAGTAAAAACAXXXXXXXXXXCCAAATTTTGTCCCCCATTTACTGTCCTTTTAAATATTTCTTTCTTGAAATCCCAGTAATCATTTTTTATCCCTTTTTTT

CAAACACCAAAGAAGTCTACTGAGGAACTGTTAGGAGCCTCTAATAACAAGTCTTGAAAGTGTTTAAAATAGTAAAAACAXXXXXXXXXXCCAAATTTTGTCCCCCATTTACTGTCCTTTTAAATATTTCTTTCTTGAAATTCCAGTAACCATTTTTTATCCCTTTTTTT

>Marker92707

TCCTCTTCTCTTCTTCTTACACCATACCCTGTGAAATTACCATAAGAGAACATAAAGAGTGAATAGAATCAAGGACTAATXXXXXXXXXXTGTTTTAGGTCAAGATTGTTATCTGGAATTTACCAAACTAACTAACAGTAAAATAAAAATTCAAATTAGGTTCTGTAACT

TCCTCTTCTCTTCTTCTTACACCATACCCTGTGAAATTACCATAAGAGAACATAAAGAGTGAATAGAATCAAGGACTAATXXXXXXXXXXTGTTTTAGGTCAAGATTGTTATCTGGAATTTACCAAACTAACTAACAGTAAAATAAAAATTCAAATTTGGTTCTGTAACT

>Marker92752

CTTATAGGCACGAACCAGGTTGGTTAAGTTCTGCCTTTGTCTATAAATCCAGTTTTTGCATCCACTGGTGTTATAACTCAXXXXXXXXXXCCAAAATCTGCCTCACGGCTTTGAAGTTTTCTTCTGAAAAGGTAGGAATGCTCGGTCGCTGAATTGTTAAGGCTCCATTT

CTTATAGGCACGAACCAGGTTGGTTAAGTTCTGCCTTTGTCTATAAATCCAGTTTTTGCATCCACTGGTGTTATAACTCAXXXXXXXXXXCCAAAATCTGCCTCACGGCTTTGAAGTTTTCTTCTGAAAAGGTAGGAATGCTAGGTCGCTGAATTGTTAAGGCTCCATTT

>Marker92945

CATGCAACGCTTGTGAACAGAGAGATGCTACTTCCATTGTGGGATCTAGAGAATGGTAGGGGCATCAACAGTCAGAGCATXXXXXXXXXXTATTATGGAATTCTTCACTTTTCGTTGTTGATAATGTGTGTAAATATTCATCATTATAGCTCCTTTTATTGGAATAATGA

CATGCAACGCTTGTGAACAGAGAGATGCTACTTCCATTGTGGGATCTAGAGAATGGTAGGGGCATCAACAGTCAGAGCATXXXXXXXXXXTATTCTGGAATTCTTCACTTTTCGTTGTTGATAATGTGTGTAAATTTCCATCATTATAGCTCCTTTTATTGGAATAATGA

>Marker93147

TCCTTCACACTCTCCCCTGTTGATTCAGCCACACTCTTTGCAGCAGACAATGGCTTCCCTGCTATGGCTCCAGCATACTCXXXXXXXXXXTCAAGGTAATGAGCTGTTTTTTGTCCTGCGGAAACTGCAACGTCCTTGGCTTCTATTGCCTTTTGAACTGTGTAACCTTT

TCCTTCACACTCTCCCCTGTTGATTCAGCCACACTCTTTGCAGCAGACAATGGCTTCCCTGCTATGGCTCCAGCATACTCXXXXXXXXXXTCAAGGTAATGAGCTGTTTTTTGTCCTGCGGAAACTGCAACGTCTTTGGCTTCTATTGCCTTTTGAACTGTGTAACCTTT

>Marker93225

TACATGAAAATGCCAAAGCATACAATCATGTTCATAAGTAACAAAACAGGAAGATAAAAGACTAGTCCTATAAATGCTACXXXXXXXXXXTAAGAAATTTTGGACACTTGCAAAACGTTATGTAAGAAGAGCCCTGCATAGAGAGAAATCTTCCCCTTCCTAACAATGGG

TACATGAAAATGCCAAAGCATACAATCATGTTCAGAAGTAACAAAACAGGAAGATAAAAGACTAGTCCTATAAATGCTACXXXXXXXXXXTAAGAAATTTTGGACACTTGCAAAACGTTATGTAAGAAGAGCCCTGCATAGAGAGAAATCTTCCCCTTCCTAACAATGGG

>Marker93641

AAGCGCCACCATGGTCGTAGTGGTCTCCTTTTCGACCCCTTTTGGACTTGAAAAAGCTAAAGATTGAAAAAGATGAGTGAXXXXXXXXXXTTTGTAGGCTAATTATTAAAACACTTTTCATTTCCTCCATTGCTTTAACAAACATGTTGACTAAAAGAATCATTTGCTTG

AAGCGCCACCATGGTCGTAGTGGTCTCCTTTTCGACCCCTTCTGGACTTGAAAAAGCTAAAGATTGAAAAAGATGAGTGAXXXXXXXXXXTTTGTAGGCTAATTATTAAAACACTTTTCATTTCCTCCATTGCTTTAACAAACATGTTGACTAAAAGAATCATTTGCTTG

>Marker93719

TTTATAGATGTCATGTGCATGTTTAGGTTAAATTTCGACAAACAGGAGGTGTCAATAATTTCTCATGTAAGGTTCAATATXXXXXXXXXXTTCTCGAAAGGATTTTGAATAGTCTTCACTTCAAGGAGGCTACAGAACACAATACGTAAAGGTTTTCGCTGAAATTTGAG

TTTATAGATGTCATGTGCATGCTTAGGTTAAATTTCGATAAACAGGAGGTGTCAATAATTTCTCATGCAAGGTTCAATATXXXXXXXXXXTTCTCGAAAGGATTTTGAATAGTCTTCACTTCAAGGAGGCTACAGAACACAATACGTAAAGGTTTTCGCTGAAATTTGAG

>Marker93956

AACCCCTATAAAATAATCCTATTTTGTTTAAAACAAAACATTATTGAATATATATTGTAATTTAACACTTCGAAATTAAAXXXXXXXXXXATGATAAGATAACCCATTTATATATATTAATTAATTGCATCATTAACGCGTTTTTTTTATGATTATATAAATGACCTTAA

AACCCCTATAAAATAATCTTATTTTGTTTAAAACAAAACATTATTGAATATATATTGTAATTTAACACTTCGAAATTAAAXXXXXXXXXXATGATAAGATAACCCATTTATATATATTAATTAATTGCATCATTAACGCGTTTTTTTTATGATTATATAAATGACCTTAA

>Marker93957

TATTTATTCTTTTCAACATATTCTAAACATATAACAGAGAAAAATTGACATAAAGAAATGGATACAGTATACTGACTAAAXXXXXXXXXXGAAGAATACCAAATAAGAATATCATACCATGAACAAGTCCAATCTTTTCACATACAAATGCATCTCCAATTTGGTAATAT

TATTTATTCTTTTCAACATATTCTAAACATATAACAGAGAAAAATTGACATAAAGAAATGGATACAGTATACTGACTAAAXXXXXXXXXXGAAGAATACCAAATAAGAATATCATACCATGAACAAGTCCAGTCTTTTCACATACAAATGCATCTCCAATTTGGTAATAT

>Marker94398

TCATAGACTAGAAGTTTTTCTTCTCCTTCAACACAACATCCCAAGAGCCTGACCAGATTCTTATGTTGAAGCTTCCAAATXXXXXXXXXXCACTAACCTTGTAAACTGGACCAAACCCCCCTTCTCCAAGTTTATTAACATCTGAAAAGTTATTTGTAGCAGCTTTTAGA

TCATAGACTAGAAGTTTTTCTCCTCCTTCAACACAACATCCCAAGAGCCTGACCAGATTCTTATGTTGAAGCTTCCAAATXXXXXXXXXXCACTAACCTTGTAAACTGGACCAAACCCCCCTTCTCCAAGTTTATTAACATCTGAAAAGTTATTTGTAGCAGCTTTTAGA

>Marker94595

TATTGTTATATCATTTTTCCCGTCTTTTTACTTGGTTGTGTAAAATCCATTTGCTTGCCAAATTTCAATGTATTACCTCAXXXXXXXXXXATCTTGGCTTATCTTCAACTCTAGCGACTTTTGTCCCATGATTTGCTCTTGAAGAGGAGGTTCGATCATCCCATTTTGAA

TATTGTTATATCATTTTTCCCGTCTTTTTACTTGGTTGTGTAAAATCCATTTGCTTGCCAAATTTCAATGTATTACCTCAXXXXXXXXXXATCTTGGCTTATCTTCAACTCTAGCTACTTTTGTCCCATGATTTGCTCTTGAAGAGGAGGTTCGATCATCCCATTTTGAA

>Marker95448

TCAACTCCAAGTTTGATGTAGTTTATGATTGTATATTAGGTCATAAATTTATTTCCTTTTTTATAGAGGTCTATTATGAAXXXXXXXXXXGTAAGAAACATCCTCTAATCGAAAAACAAAGTTCATGGTGAGCATATGTGAGTAAATTTGCTAGAACCTCTCAAATATCT

TCAACTCCAAGTTTGATGTAGTTTATGATTGTATATTAGGTCATAAATTTATTTCCTTTTTGATAGAGGTCTATTATGAAXXXXXXXXXXGTCATAAACATCCTCTAATCGAAAAACAAAGTTCATGGTGAGCATATGTGAGTGAATTTGCTAGAACCTCTCAACTATCT

>Marker95548

CCTACGAGGTTTTGATCTCGTGAAAGACAGAGAGACTGGAAATTCAAAAGGATATGCATTTTGTGTTTACCAAGATCTTTXXXXXXXXXXCAATTGCCTCCTTTCTACCTAAGAATTTTTAAATACCAGTTCATCATTGAAATTATCAATTGAATAATTTCGGGGAAAAA

CCTACGAGGTTTTGATCTCGTGAAAGACAGAGAGACTGGAAATTCAAAAGGATATGCATTTTGTGTTTACCAAGATCTTTXXXXXXXXXXCAATTGCCTCCTTTCTACCTAAGAATTTTTAAATACCAGTTCATCATTGAAATTATCAATTGAATAATTTTGGGGAAAAA

>Marker95579

TTGTTGTTCCCAATGATGCCACCCTTGTCACTGTTCTATCTGCTTGTGCAAGATTAGGAGCTCTTGACTTGGGAAAGTGGXXXXXXXXXXTGCATTAACTTTGTTTCACCAGATGAAGATCAATGGCGAAAAACCAGATGGAATCACCTTCATCGGGGTGTTGTGCTCCT

TTGTTGTTCCCAATGATGCCACCCTTGTCACTGTTCTATCTGCTTGTGCAAGATTAGGAGCTCTTGACTTGGGAAAGTGGXXXXXXXXXXTGCATTAACTTTGTTTCACCAGATGAAGATCAATGGCGAAAAACCAGATGGAATCACCTTCATTGGGGTGTTGTGCTCCT

>Marker95600

ATGAGTTTGGGAATCTATGAGGAAATTTCGCTTCAAATGGTGTTATCTGATCTTCACCAGGACTATGAGGTGTCTATGAAXXXXXXXXXXGTTAGCAAGTTGGGTCTTTTTGACATTTACGGTCCAACTTGAGGGGGAATGTTAGCAAGTTAGGTCTTTTTGACATTTAC

ATGAGTTTGGGAATCTATGAGGAAATTTCGCTTCAAAAGGTGTTATCTGATCTTCACCAGGACTATGAGGTGTCTATGAAXXXXXXXXXXGTTAGCAAGTTGGGTCTTTTTGACATTTACGGTCCAACTTGAGGGGGAATGTTAGCAAGTTAGGTCTTTTTGACATTTAC

>Marker95826

TTTTTCTAGTTTTCAAAACTTGTTTGCCAATTTTGAAAATATGGTGAAAGAGTTTTTATAACAAACAAGATATTTAGAGAXXXXXXXXXXCTTTTGTGGGAAGCAAATGGTGCGTTGGCTTAGATTGCCCTAAAGCAATGATGCAAACGATGATGTATCTTGGATTTAAA

TTTTTCTAGTTTTCAAAACTTGTTTGCCAATTTTGAAAATATGGTGAAAGAGTTTTTATAACAAACAAGATATTTAGAGAXXXXXXXXXXCTTTTGTGGGAAGCAAATGGTGTGTTGGCTTAGATTGCCCTAAAGCAATGATGCAAACGATGATGTATCTTGGATTTAAA

>Marker96122

CTCGAATCACTTGGTTTATTGTGTATTTCTGCTATTTTATATATTCAATATATACAGGTTCTATCAGGTCGCTTCATGAAXXXXXXXXXXATAGTGCATGTAAGAAATACATAATAAAATTGATCAACTAAAACTAGTAATTAGTGCAGTTTTAACTAAAACAAAAATAA

CTCGAATCACTTGGTTTATTGTGTATTTTTGCTATTTTATATATTCAATATATACAGGTTCTATCAGGTCGCTTCATGAAXXXXXXXXXXATAGTGCATGTAAGAAATACATAATAAAATTGATCAACTAAAACTAGTAATTAGTGCAGTTTTAACTAAAACAAAAATAA

>Marker96278

CACCACACCTCCGACGATCAATAACACTTGGGTAACCAATTTTAATGACCAACTTTAACGTTCATTGTTTAGAAGTGGATXXXXXXXXXXTATGAATCTTAATTTTAATTTTAATCCGAGTAATCTCTAGGGTTTATCGGGGCTTGTCTCTAGCCCTTAGATCTTTTGAT

CACCACACCTCCGACGATCAATAACACTTGGGTAACCAATTTTAATGACCAACTTTAACGTTCATTGTTTAGAAGTGGATXXXXXXXXXXTATGAATCTTAATTTTAATTTTAATCCTAGTAATCTCTAGGGTTTATCGAGGCTTGTCTCTAGCCCTTGGATCTTTTGAT

>Marker96284

ACAACGAGGTCGAAATGAGGGAACTCACATTTCAAAGCATCTTGACATATGACCTACAAAACCCCACCGCCAGCAGTTTAXXXXXXXXXXCTCCAAGACCGTATCGGTCGGAAGTATTGCAGATTTTCGAACAATAGAGTCCAGAATTCGTTGGTTTGTAAGAATGTGCT

ACAACGAGGTCGAAATGAGGGAATTCACATTTCAAAGCATCTTGACATATGACCTACAAAACCCCACCGCCAGCAGTTTAXXXXXXXXXXCTCCAAGACCGTATCGGTCGGAAGTATTGCAGATTTTCGAACAATAGAGTCCAGAATTCGTTGGTTTGTAAGAATGTGCT

>Marker96335

CGCCCACCGACGATCTGAGCCTCTCCCCACCTGAGTTTGCTTCTGACCCTTTCATTGATTTGATCGCAGCTTTCAAGAAAXXXXXXXXXXGTGTTCTTGTGGATTGAGCTGTCTTTGGACTAAATCTTCTGTTCTTGCTGATAAACCCGACGCCTTGTTATTCGAAACCA

CGCCCACCGACGATCTGAGCCTCTCCCCGACTGAGTTTGCTTCTGACCCTTTCATTGATTTGATCGCAGCTTTCAAGAAAXXXXXXXXXXGTGTTCTTGTGGATTGAGCTGTCTTTGGACTAAATCTTCTGTTCTTGCTGATAAACCCGACGCCTTGTTATTCGAAACCA

>Marker96573

TTCGATCACCATCTTTCCGATGAGATGCTACAAATGAGTTTGAGGATTCTAAGCCGAGGTTGTTGGAACTAGTAAAATTTXXXXXXXXXXATCTCGTTTCATGGTCGTCATATCATTTGCTTCCTCTATAGTTGTGACTTTCATATTAAATCGAGAAGGAAGAGATCTTA

TTCGATCACCATCTTTCCGATGAGATGCTACAAATGAGCTTGAGGATTCTAAGCCGAGGTTGTTGGAACTAGTAAAATTTXXXXXXXXXXCACCCGTTTCATGGTCGTGATATCATTTGCTTCCTCTATAGTTGTGACTTTCATATTAAATCGAGAAGGAAGAGATCTTA

>Marker96607

TTTCCTGATTATGAAATTTTGGGTTGAAACAAGGCAGGTTACTCTTAAGGGGGATCCCTCCTTGGTGAGATCAGAATGTTXXXXXXXXXXCTAAAAGAGAAGTTGATCATCGAATCTTGATGATGCCCAAGCAAAAACCAATTAACGTGAGACCTTACAAGTATGGACAT

TTTCCTGATTATGAAATTTTGGGTTGAAACAAGGCAGGTTACTCTTAAGGGGGATCCCTCCTTGGTGAGATCAGAATGTTXXXXXXXXXXCTAAAAGAGAAGTTGATCACCGAATCTTGATGATGCCCAAGCAAAAACCAATTAACGTGAGACCTTACAAGTATGGACAT

>Marker96639

AAAACAAGGTGTTTTTGTCGTTCTCCGTCTGGTCGTCTGAGTCAGCCTTGATCCAGTTTGGGTCACGTCTCCAAAACATCXXXXXXXXXXACAAAAGTAATTTGTCGCCATGATGTTTTTCATCTTGTTTATATCTAGTTGTGAGAACATTGGGATGATTCTTGCAACTA

AAAACAAGGTGTTCTTGTCGTTCTCCGTCTGGTCGTCTGAGTCAGCCTTGATCCAGTTTGGGTCACGTCTCCAAAACATCXXXXXXXXXXACAAAAGTAATTTGTCGCCATGATGTTTTTCATCCTGTTTATATCTAGTTGTGAGAACATTGGGATGATTCTTGCAACTA

>Marker96777

CAAAGATGATGATAAAAACATTTTCCAAATACCTACAAAAGTTTCCTATTAAACATACTAGAGTTACACGAACTTAACGAXXXXXXXXXXTGTAAGATTGTGTGTTTTGGAATGGTGTAGCAAAGTTATACCAAAAATTTCGAAAAAACAAATTTGAGATACAGAGTTTA

CAAAGATGATGATAAAAACATTTTCCAAATACCTACAAAAGTTTCCTATTAAACATACTAGAGTTACACGAACTTAACGAXXXXXXXXXXTGTAAGATTGTGTGTTTTGGAGTGGTGTAGCAAAGTTATACCAAAAATTTCGAAAAAACAAATTTGAGATACAGAGTTTA

>Marker96955

GCATCCATAGTGTTACCGGAATAAGGCGTGCCTAACCTTATTCATACATTATAGACCATTTAGGCTATGAACTCAAACTTXXXXXXXXXXCATCCAATAAACTTTGGACCACGATCTACGGTCATCGATGATACCAACAACATAGTCAAATAAGAATCTCAAATTTGATG

GCATCCATAGTGTTACCGGAATAAGGCGTGCCTAACCTTATTCATACATTATAGACCATTTAGGCTATGAACTCAAACTTXXXXXXXXXXCATCCAATAAACTTTGGACCACGATCTACGGTCATCGATGATACCAACAACATAGTCAAATAAGAATCTCAAATCTGATG

>Marker97015

TCCTCAGTGTTTCCAGTTTCACCAGCATTGTGAGAAGATTAGTTTAACTCATCTAACCTTAGCGGATGATCTTATGATATXXXXXXXXXXTCTTGGGTTTCCTTTACTTTCTGGGAGGTTCAGGCTAACTGATTGTGCTCCTCTTATTCAGTGGATTACTAGTCAGATCC

TCCTCAGTGTTTCAAGTTTCACCAGCATTGTGAGAAGATTAGTTTAACTCATCTAACCTTAGCGGATGATCTTATGATATXXXXXXXXXXTCTTGGGTTTCCTTTACTTTCTGGGAGGTTCAGGCTAACTGATTGTGCTCCTCTTATTCAGTGGATTACTAGTCAGATCC

>Marker97361

GGAGTTTGGGCTTGGGGTTGAGTTAATTATGCCCTAAAATTCTCGCCGTAATTGACGGAAGCATTTAAACGGCAAATTTAXXXXXXXXXXACACTTCCCACACTAACGCACGCGTAAACTTTCCACAGCCCCTCTCCATGCCGTCCTTACTCGTCATATAATACGCTGCG

GGAGTTTGGGCTTGGGGTTGAGTTAATTATGCCCTAAAATTCTCGCCGTAATTGACGGAAGCATTTAAACGGCAAATTTAXXXXXXXXXXACACTTCCCACACTGACGCACGCGTAAACTTTCCACAGCCCCTCTCCATGCCGTCCTTACTCGTCATATAATACGCTGCG

>Marker97881

ACTTCATTTAATAGGATCTTAATAAATATAATACTCCACACGTTAGATATTAAACTCACGAAATTAACTAAATTGATGAAXXXXXXXXXXAATAGAATTAAGAAGGGATGTTGGTTGCAAGAATCAACCGCTAGATTTGTTCGCACCCGATCCACGCGGTGAACTTCCAT

ACTTCATTTAATAGGATCTTAATAAATATAATACTCCACACGTTAGATATTAAACTCACGAAATTAACTAAATTGATGAAXXXXXXXXXXAATAGAATTAAGAAGGGATGTTGGTTGCAAGAAGCAACCGCTAGATTTGTTCGCACCCGATCCACGCGGTGAACTTCCAT

>Marker97928

ATTATGAGTAGGAGTGAGTATGATAAAGAAAAAATCCGAGCTGACCGACCAGATCAAAGTTGGTCAATCAAATAATGAGAXXXXXXXXXXGTAAGCACCTTACTCGAGGAAGGTGTAGTTCTCACCATCTACCAAAGGTCAATTTCTTACTTATTAAAATTTGAGAATAA

ATTATGAGTAGGAGTGAGTATGATAAAGAAAAAATCCGAGCTGACCGACCAGATCAAAGTTGGTCAATCAAATAATGAGAXXXXXXXXXXGTAAGCACCTTACTGGAGGAAGGTGTAGTTCTCACCATCTACCAAAGGTAAATTTCTTACTTATTAAAATTTGAGAATAA

>Marker97957

TAGACACTTATGGATATATAAAAAAAATGCATAAAGACATAAGATATGAGAGATTTCACCCACCTTACCTAAAGCACAACXXXXXXXXXXCACCAAGTTGCCACAACAGGTTTTGAAATAGGAAACAAAAATTTCCATTGATATAATGAAAGGAGATAAGGCTCAAAAAA

TAGACACTTATGGATATATAAAAAAAATGCATAAAGACATAAGATATGAGAGATTTCACCCACCTTACCTAAAGCACAACXXXXXXXXXXCACCAAGTTGCCACAACAGGTTTTAAAATAGGGAACAAAAATTTCCATTGATATAATGAAAGGAGATAAGGCTCAAAAAA

>Marker97994

TTCTACTTCTAAGCTCAGTGTATTAAGCAAGATGTGAAGTAAGATGTGTCAAAATAGTAAATGACTGCCAACATGAGCTTXXXXXXXXXXAAAAAAGTAAAAACAATATCAAAGGTTTAGGAATTAATACAGCGAGAGGCTATATTTTTGTATATATAAAAAACACCAAC

TTCTACTTCTAAGCTCAGTGTATTAAGCAAGATGTGAAGTAAGATGTGTCAAAATAGTAAATGACTGCCAACATGAGCTTXXXXXXXXXXAAAAAAGTAAAAACAATATCAAAGGTTTAGGAATTAATACAGCGAGAGGTTATATTTTTGTATATATAAAAAACACCAAC

>Marker98073

TATGTTATTCCAGTAAAAATGAAAAGGCAACACAAAACAATACCTTGCACACAGTAAAAATTTTTGAAAGGGCTTTTCTCXXXXXXXXXXATTAAAAGAAAAGCAATATGATATGAAGCGTATGGAGAACTGGTGAAGTATAACACAAAGTGACGTCCGAAGATAAACAG

TATGTTATTCCAGTAAAAATGAAAAGGCAACACAAAACAATACGTTGCACACAGTAAAAAATTTTGAAAGGGCTTTTCTCXXXXXXXXXXATTAAAAGAAAAGCAATATGATATGAAGCGTATGGAGAACTGGTGAAGTATAACACAAAGTGATGTCCGAAGATAAACAG

>Marker98090

CAAATAAATAAAATCTTATTTATGATAACTACATGATAGCTCCATTTCCCCCCTCTTTTGTCCAATTGGCTGTCTTTTGTXXXXXXXXXXATTGTATTTGTAAGATTAGTGAATCACTTTACTACAGACGTGATAATGCAAGCATTGAGACTTGGTTTCTAAAAACTTGT

CAAATAAATAAAATCTTATTTATGATAACTACATGATAGCTCCATTTCCCCCCTCTTTTGTCCAATTGGCTGTCTTTTGTXXXXXXXXXXCTTGTATTTGTAAGATTAGTGAATCACTTTACTACAGACGTGATAATGCAAGCATTGAGACTTGGTTTCTAAAAACTTGT

>Marker98468

AGAAAAATGATATTTAGGGTTTCAAAAGCTACAAATAGTCAAATAATATTATTTTTATATTAAATTTTAAATGGGTGTCAXXXXXXXXXXTGCATATACTTTTTTGGCAAAGGGCATTAGACTTTTTAGGTCTTTGCAATAATCCCACTAAAGTTTGTCACCCTTCCAAA

AGAAAAATGATATTTAGGGTTTCAAAAGCTACAAATAGTCAAATAATATTACTTTTATATTAAATTTAAAATTGGTGTCAXXXXXXXXXXTGCATATACTTTTTTGGCAAAGGGCATTAGACTTTTTAGGTCTTTGCAATAATCCCACTAAAGTTTGTCACCCTTCCAAA

>Marker98518

GTGCCAATGTCATTCCCTGGTTGTCTTGGACCCGATATCAACATTGATAACATCATGAACTTTCTTTTCATGCACAACCAXXXXXXXXXXCGCTCATTAGCATGCCAAATCAAATTTTTAGCATTGTCAATACTTCGAAACAATCTTTTGGAACGTGGAATCGGTGGAAA

GTGCCAATGTCATCCCCTGGTTGTCTTGGACCCGATATCAACATTGATAACATCATGAACTTTCTTTTCATGCACAACCAXXXXXXXXXXCGCTCATTAGCATGCCAAATCAAATTTTTAGCATTGTCAATACTTCGAAACAATCTTTTGGAACGTGGAATCGGTGGAAA

>Marker98618

CATATACAATCAATCATACATACAAGTTGCTAAAGAAATTTGTCTTAAGAATTGTTGTCAGTATTATCATCACAACAAACXXXXXXXXXXTCTTCTTTCAATCAAATAGAAAAGAGACTTACTCGTTACCATTCGAAAAGTTTATATCCTTTTCTATAACTTAGCTATTA

CATATACAATCAACCATACATACAAGTTGCTAAAGAAATTTGTCTTAAGAATTGTTGTCAGTATTATCATCACAACAAACXXXXXXXXXXTCTTCTTTCAACCAAATAGAAAAGAGACTTACTCGTTACCATTCGAAAAGTTTATGTCCTTTTCTATAACTTAGCTATTA

>Marker98645

GTTCTTAGTTGCTAGTTTATGTTTTTAGATTTTTAGGTAGAAATCTGAGGTGGATGGGAAATCTAATGTAGTTATTATTGXXXXXXXXXXATGCAAAATCCTATTCTCGTGGCATTTCGGAGCAATGTTGCCAAGTTCTATTGCTTATTCATGAAATCTCGACCGAGAAT

GTTCTTAGTTCTTAGTTTATGTTTTTAGATTTTTAGGTAGAACTCTGAGGTGGATGGGAAATCTAATGTAGTTATTATTGXXXXXXXXXXATGCAAAATCCTAATCTCGTGGCATTTCGGAGCAATGTTGCCAAGTTCTATTGCTTATTCATGAAATCTCGATCGAGAAT

>Marker99235

CTCTTTAAAGAAAATGGTATATAATGTTATGTTTGAAGTGATAACTTTTGTAATTGATAAACGGATGAAAGCCCAAGCATXXXXXXXXXXTAGTTGCAATTATACTTTCAAACTTTCCATCATAAAAATTGAACGCCCACAAACTTATACAATAAAACACCCACAAACGT

CTATTTAAAGAAAATGGTATATAATGTTATGTTTGAAGTGATAACTTTTGTAATTGATAAACGGATGAAAGCCCAAGCATXXXXXXXXXXTAGTTGCAATTATACTTTCAAACTTTCCATCATAAAAATTGAACGCCCACAAACTTATACAATAAAACACCCACAAACGT

>Marker99238

AAGAAAGTTCTCACCACAGGTGTTAACGGTGCACTTCACCCATCAAGATTTTGTGAAAAAGATTTATTGAAAGTGGTTGAXXXXXXXXXXGTAGAAAATGCAAGATTGTCCTATGAAAATGGAACGTCCACCATTGAAAACCAAATTAAAGAATGTAGATCTTATCCATT

AAGAAAGTTCTCACCACAGGTGTTAACGGTGCACTTCACCCATCAAGATTTTGTGAAAAAGATTTACTGAAAGTGGTTGAXXXXXXXXXXGTAGAAAATGCAAGATTGTCCTATGAAAATGGAACGTCCACCATTGAAAACCAAATTAAAGAATGTAGATCTTATCCATT

>Marker99314

ATGGATCCACGTAAATCTGTGAGAAACAACAAAACTATTCACCTTCTCCTTATTCCTTGGTTCATAACTAGCAGTCATGGXXXXXXXXXXATATTAACACAAACATCATTTTAATGCAATAGCTCATTGCGTTTAACTGTCACAGACAGGTCCAAGGAGTATCATCGTTG

ATGGATCCACGTAAATCTGTGAGAAACAACAAAACTATTCACCTTCTCCTTATTCCTTGGTTCATAACTAGCAGTCATGGXXXXXXXXXXATATTAACACAAACATCATTTTAATGCAATAGCTCATTGCGTTTAACTGTCACAGACAGGTCCAAGGAGTATCTTCGTTG

>Marker99527

ATTGATGTGTTGATTATCTTTACGTTTTTGTTTTGTATTGCTTAATTGTTGATTTCCTAATTGAAGAGGGTGTTAAAGGAXXXXXXXXXXGTCCTTTTATCGTTAATTACCTAGGTTATGTTTGTCGGTTATCTTGATCTTGAGTAGTTGTTTGGTTATTTATAGTTGTG

ATTGGTGTGTTGATTATCTTTACGTTTTTGTTTTGTATTGCTTAATTGTTGATTTCCTAATTGACGAGGGTGTTAAAGGAXXXXXXXXXXGTCCTTTTATCGTTAATTACCTAGGTTATGTTTGTCGGTGATCTTGATCTTGAGTAGTTGTTTGGTTATTTATAGTTGTG

>Marker99649

CATCATTTAAATATGCATATATATGAAATTATGTTGACCTCTCAATCCTTTTGAGCCAAGACAAAAAGTGGGGCATGGGCXXXXXXXXXXTTTTCAATTTTGTGGAATTTCTCTGAGGAATTGTTCCTCGCAGGGAGGAATTGTTCCTTGCATGGAGTTGTTCCTGGCAA

CATCATTTAAATATGCATATATATGACATTATGTTGACCTCTCAATCCTTTTGAGCCAAGACAAAAAGTGGGGCATGGGCXXXXXXXXXXTTTTCAATTTTGTGGAATTTCTTTGAGGAATTGTTCCTCGCAGGGAGGAATTGTTCCTTGCATGGAGTTGTTCCTTGCAA

>Marker99674

CAACATATAATCTTCCTGTTCGAAATAACCAATATGTTAGATTTACAATAATTGAATGAACAAATACAAAAAAGACAAATXXXXXXXXXXTATTTTTTTTCATACAACCAATCAAATCCAATAATAATAGAGAATACTATGAAGAATTAACAATTTTATAACAAAATCCA

CAACGTATAATCTTCCTGTTCGAAATAACCAATATGTTAGATTTACAATAATTGAATGAACAAATACAAAAAAGACAAATXXXXXXXXXXTATTTTTTTTCATACAACCAAGCAAATCCAATAATAATAGAGAATACTTTGAAGAATTAACAATTTTATAACAAAATCCA

>Marker99872

ATATTTTGGGGAAAATGTAGAATTATCTCAGAAATTACAAAGTGCTTTAGAACTTTCGAGGCACTACAAAAGCTTCCTTAXXXXXXXXXXAATTTATAATATTATATATTATTTAAGTGGCGGATTGTTTCTAATTTGCATCTTCGGTTTCTTGATTGTTGTGGAAGTTC

ATATTTTGGGGAAAACGTAGAATTATCTCAGAAATTACAAAGTGCTTTAGAACTTTCGAGGCACTACAAAAGCTTCCTTAXXXXXXXXXXAATTTATAATATTATATATTATTTAAGTGGCGGATTGTTTCTAATTTGCATCTTCGGCTTCTTGATTGTTGTGGAAGTTC

>Marker99899

TTTATGGAAACTAATTAAGGACAGAGAAATTCAGTTCATAAGATTCTAGTTCAGTGGAGTTTGCATGGGAGCAAAGATCTXXXXXXXXXXCTTAAATACATATGATCATCCTCTTATCTAGCTAATATGGAACTTTGGTTGTATTCTCAACAAAGCTCCCCTTGAACAAA

TTTATGGAAACTAATTAAGGATAGAGAAATTCAGTTCATAAGATTCTAGTTCAGTGGAGTTTGCATGGGAGCAAAGATCTXXXXXXXXXXCTTAAATACATATGATCATCCTCTTATCTAGCTAATATGGAACTTTGGTTGTATTCTCAACAAAGCTCCCCTTGAACAAA

>Marker99928

AGTTTAACTAGTTTTGAATGCTTACCACTAGTCGATTGCCGACATTATTTCAGTTTTGAATTGAAGAAAGTTTAGGGAAAXXXXXXXXXXGTTAGTGAGTGAGCCTTTTCTAACTGTTTGATCACAAGCAATTGTATCCCCCTGTAATTGATCATTTATTGCTTGGTTTT

AGTTTAACTAGTTTTGAATGCTTACCACTAGTCGATTGCCGACATTATTTCAGTTTTGAATTGAAAAAAGTTTAGGGAAAXXXXXXXXXXGTTAGTGAGTGAGCCTTTTCTAACTGTTTGATCACAAGCAATTGTATCCCCGTGTAATTGATCATTTATTGCTTGGTTTT

>Marker100329

AGCAGCAGAGAATTTGGTTCCGACACCTTTATTGATAAATTTCTCATTTCGGATGGAATCTTATCTAAATTTGACTTTCTXXXXXXXXXXGGAATTCATCCACTGACTCTAACTACAAAAGTCAGCTTCAGATAGTGCATAATAAGTCATTGACCCAAATGAGGAAAATG

AGTAGCAGAGAATTTGGTTCCGACACCTTTATTGATAAATTTCTCATTTTGGATGGAATCTTATCTAAATTTGACTTTCTXXXXXXXXXXGGAATTCATCCACTGACTCTAACTACAAAAGTCAGCTTCAGATAGTGCATAATAAGTCATTGACCCAAATGAGGAAAATG

>Marker100687

CATTTGATGAAGTTGAAAGTTTGTAGTATAAAGTAAAAATAGAATTTTCCCCTAACTTTCTTCTATTTCAACAACATAATXXXXXXXXXXCCATTATAAGTTATTAATAGCATTCTGAATTCTCAATTTTGGAAGAGAGCTCTCTACAAAATGAAGGAAAATACATCAAT

CATTTGATGAAGTTGAAAGTTTATAGTATAAAGTAAAAATAGAATTTTCCCCTAACTTTCTTCTATTTCAACAACATAATXXXXXXXXXXCCATTATAAGTTATTAATAGCATTCTGAATTCTCAATTTTGGAAGAGAGCTCTCTACAAAATGAAGGAAAATACATCAAT

>Marker100813

TTAAGAACAATTGTAACAACTACAAAGCATGCCATATTCGTAGTGTCATTAGGATAAGGTATCCAGCTTTATCCATCTACXXXXXXXXXXATACAATTACAAACTACAAGACCACAAGATTTAGGGCATAAACCCCAACAATCTCTCGCTTGTCCTAAATTTAGTGGGGT

TTAAGAACAATTGTAACAACTACAAAGCATGCCATATTCGTAGTGTCATTAGGATAAGGTATCCAACTTTATCCATCTACXXXXXXXXXXATACAATTACAAACTACAAGACCACAAGATTTAGGGCATAAACCCCAACAATCTCTCGCTTGTCCTAAATTTAGTGGGGT

>Marker101218

CCAAAAAAAAAAAGTGTCCAGGTGACAAACTTTGACGTGCTTCATACACTAGCACAAGGGAGATTTTTTTAACTTAAAAAXXXXXXXXXXAGAATAAGATACATAAAAGTAGTAATGTTTGATATTTCATAAAGTTTTGGTCGTTTTTAGTAAATATCTCAATGTAGTGG

CCAAAAGAAAAAAGTGTCCAGGTGACAAACTTTGACGTGCTTCATACACTAGCACAAGGGAGATTTTTTTAACTTAAAAAXXXXXXXXXXAGAATAAGATACATAAAAGTAGTAATGTTTGATATTTCATAAAGTTTTGGTCGTTTTTAGTAAATATCTCAATGTAGTGG

>Marker101250

CAAAATGAGAAATACCAAAAATGACCCATGATTAATTGGTATCTAGCACTTGTAATATATTTCGGAAACAATAATTTATAXXXXXXXXXXACGATTTTTTAAAAAACTCTTTGGCACACGATGTTTAGATTTGGGTAGCCAAATCTAAACAATTTTTTTAAATTTTTTTG

CGAAATGAGAAATACCAAAAATGACCCATGATTAATTGGTATCTAGCACTTGTAATATATTTCGGAAACAATAATTTATAXXXXXXXXXXACGATTTTTTAAAAAACTATTTGGCACACGATGTTTAGATTTGGGTAGCCAAATCTAAACAATTTTTTTAAATTTTTTTG

>Marker101628

TCCAATCATTCACATATATAAATGTAAAAGCGAAGCTGTGAGGGGTGTGATGGGAGTTACACAGAAGATAGTTTGTGGGGXXXXXXXXXXAGCATAGCAATGTCTCTCTTTCTCTACTCATGTTAGAAATTTTCTGATTTTCATAGGATCCTGAAGAAGGATCCTAACAA

TCCAATCATTCACATATTTAAATGTAAAAGCGAAGCTGTGAGGGGTGTGATGGGAGTTACACAGAAGATAGTTTGTGGGGXXXXXXXXXXAGCATAGCAATGTCTCTCTTTCTCTACTCATGTTAGAAATTTTCTGATTTTCATAGGATCCTGAAGAAGGATCCTAACAA

>Marker101724

TCATATTGAAATTTAAGGCATGACGTTCACATTTCAATCGGAATTAATGTAAATTATTTGAGAAAAGGTCAAAACAAAAAXXXXXXXXXXGATGTGCACTTTAAATAAAATTCAAATTTGAGGGACGAAAAGTATGTTTAATAGTATCGACACGTCGGAAAACTGAGGCA

TCATATTGAAATTTAAGGCATGACGTTCACATTTCAATCGGAATTAATGTAAATTATTTGAGAAAAGGTCAAAACAAAAAXXXXXXXXXXGATGTGCACTTTAAATAAAATTCAAATTTGAGGGGCGAAAAGTATGTTTAATAGTATCGACACGTCCGAAAATTGAGGCA

>Marker101890

CGTTAGAGAATTTCTCACTATTGTTGCAGTGATAGTGGATTTTGTATGATCTGATGACAACTTAGCTAATCCTTTGACGAXXXXXXXXXXGAATTATTAGCATAACATCCTAAAGCATATGAAAAGGTTGAATTTAGTTATTAATGAATTTCATACTTGATATTAGTAAG

CGTTAGAGAATTTCTCACTGTTGTTGCAGTGATAGTGGATTTTGTATGATCTGATGACAACTTAGCTAATCCTTTGACGAXXXXXXXXXXGAATTATTAGCATAACATCCTAAAGCATATGAAAAGGTTGAATTTAGTTATTAATGAATTTCATACTTGATATTAGTAAG

>Marker102029

TTCAAGCTGGACGTTAAATTTGGGGTGCAATGGAAAACTCGTTCCTGATCTCCTGGCATCAAGATTGGGGATAAAGGAGCXXXXXXXXXXAGTGATGGTTTGACTGTTGTGAGTGTAGGAACTAATGACTTCCTATTTAATTTCTATGGCATTCCTAGAAGTTCATTGGA

TTCAAGCTGAACGTTAAATTTGGGGTGCAATGGAAAACTCGTTCCTGATCTCCTGGCATCAAGATTGGGGATAAAGGAGCXXXXXXXXXXAGTGATGGTTTGACTGTTGTGAGTGTAGGAACTAATGACTTCCTATTTAATTCCTATGGCATTCCTAGAAGTTCATTGGA

>Marker102236

ATTGGAGTGGGTTAAACGTAACTCTAAAAGGATACACAACGACGAAAACGACGAAGACGACAACAAAAAAAGGAGGAGTTXXXXXXXXXXTTTGTCCTGAAGGGACGACATGTCGAGAGCCGTATTTGTTGAGATTTAGTCCACTTTTTGCTGAATTGACAGATGAAATA

ATTGGAGTGGGTTAAACGTAACTCTAAAAGGATACACAACGACGAAAACGACGAAGACGACAACAAAAAAAGGAGTTCTAXXXXXXXXXXTTTGTCCTGAAGGGACGACATGTCGAGAGCCGTATTTGTTGAGATTTAGTCCACTTTTTGCTGAATTGACAGATGAAATA

>Marker102420

AAGGTCGAGCTCAATAATCTCACACAGGTAGTCAATCTGGATCAACCTTCTACTCTGAAACACCCATAGTGACTCAAATCXXXXXXXXXXTTTGATTGTTTTATAGGTAATCTATTTTCTAAATTACAAATTTACCATTGATGACATGTGAAAGTTAGATGAGTTCACTT

AAGGTCGAGCTCAATAATCTCACACAGGTAGTCAATTTGGATCAACCTTCTACTCTGAAACACCCATAGTGACTCAAATCXXXXXXXXXXTTTGATTGTTTTATAGGTAATCTATTTTCTAAATTACAAATTTACCATTGATGACATGTGAAAGTCAGATGAGTTCACTT

>Marker102530

ACAGATGAGTGCAACCAAAATGCATCCATTCCCAACCATTTTCCTTTCCTCCTTCCTTAGAATGTTACCCGGGTTGGTTGXXXXXXXXXXTTTTCTATCATCTGTAACAAAAGGAGTATATAAGAGAAGCTCAGTCTCGAAGAGATGATAATAAAATTCAATGACAAATC

ACAGATGAGTGCAACCAAAATGCATCCATTCCCAACCATTTTCCTTTCCTCCTTCCTTAGAATGTTACCCGGTCCCGTAGXXXXXXXXXXTTTTCTATCATCTGTAACAAAAGGAGTATATAAGAGAAGCTCAGTCTCGAAGAGATGATAATAAAATTCAATGACAAATC

>Marker102584

TTTTCATAGCGTTCTTTAGCCGCTCGTATTGCCTCATTAGATCTCTCCTGTGATATAGCTCTGTAATTTGAAATTTCTTCXXXXXXXXXXTCTCAATTGGCTTTCCACCTTCACCTTCAAATCTATGTCTTTCCACCTCTTTTCCAGGAAGTTCCCTCTTAGCAATGCTG

TTTTCATAGCGTTCCTTAGCCGCTCGTATTGCCTCATTAGATCTCTCCTGTGTTATAGCTCTGTAATTTGAAATTTCTTCXXXXXXXXXXTCTCAATTGGCTTTCCACCTTCACCTTCAAATCTATGTCTTTCCACCTCTTTTCCAGGAAGTTCCCTCTTAGCAATGCTG

>Marker102806

TTAAGAGAACAAATTACCTTCTAACCCTAAAAATGGGCAAGAGTGAATTTTGTCTTGCACTATACGTTCCTAGCTAATTCXXXXXXXXXXAGTGACTATTTTATAGTCTGATCTTATACAATCACTTTTCATAGAACACACCACTCACATGTCTCCATATGTGCGTCTCG

TTAAGAGAACAAATTACCTTCTAACCCTAAAAATGGGCAAGAGTGAATTTTGTCTTGCACTATACGTTCCTAGCTAATTCXXXXXXXXXXAGTGACTATTTTATAGTCTGATCTTATACAATCTCTTTTCATAGAACACACCACTCGCATGTCTCCATATGTGCGTCTCG

>Marker102836

TCTTCCACCACGCTCCTTACTTACCACACACAAACCCCAAACCCCACCATTTTTTCTTCTTTCGCTCTCTCCATTAACAAXXXXXXXXXXACTCAATTGGGGTTTGGCTGCTGATTCCCTCAAGGGAAGCCATCTTGATGAGGTGAAGCGCATGGTCCAGGAGTATCGAA

TCTTCCACCACGCTCCTTACTTACCACACACAAACCCCAAACCCCACCATTTTTTCTTCTTTCGCTCTCTCCATTAACAAXXXXXXXXXXACTCAATTGGGGTTTGGCTGCTGATTCCCTCAAGGGAAGCCATCTTGATGAGGTGAAGCGCATGGTCCACGAGTATCGAA

>Marker103158

ATGATTTGGATTTTGTTTCAGAGCTCAGCAAAATTGCAAACCGTCTTGACTCTATGAGTTTGACCCGTTGCATTTCTCTAXXXXXXXXXXTGTTCAACTTTTGCATAGAAAATTAATTTAGTTATTTATCACTTAACGAAATTCTGGATCCATTTTGAAATATGGGAATT

ATGATTTGGATTTTGTTTCAGAGCTCAGCAAAATTGCAAACCGTCTTGACTCTATGAGTTTGACCTGTTGCATTTCTCTCXXXXXXXXXXTGTTCAACTTTTGCATAGAAAATTAATTTAGTTATTTATCACTTAACGAAATTCTGGATCCATTTTGAAATATGGGAATT

>Marker103509

CTCTAAGGCTCCCAAAAAAAGTGGGTAATCTTGTGTAGGAGAGTCATTAGAAGATGGGTGGATAATCCAGTTTGTATGCTXXXXXXXXXXGATATTGTAAGAAACTAGATTTCTTGAAGATACTGTTGTATTGCCAAATGGAAAATTGATCCTACAAATTTACAAATTTT

CTCTAAGGCTCCCAAAAAAAGTGGGTAATCTTGTGTAGGAGAGTCATTAGAAGATGGGTGGATAATCCAGTTTGTATGCTXXXXXXXXXXGATATTGTAAGAAACTAGATTTCTTGAAGATACTGTTGTATTGCCAAATGGAAAATTGATCCTGCAAATTTACAAATTTT

>Marker103958

GAAGTTATTTGCAGACATCCGCAAACTTCGGAAGATTGTATGAAAGAAGCCCAGTTGGTCAATGATAGGAATTTGGCATTXXXXXXXXXXTGAAAGATATTCGCCAAGAAATAGGTGCAAAATGAAGGAAAAAAAGGAGTTGATGTTGTTCATCCTAAATGAAGAGGAGA

GAAGTTATTTGCAGACATCCGCAAACTTTGGAAGATTGTATGAAAGAAGCCCAGTTGGTCAATGATAGGAATTTGGCATTXXXXXXXXXXTGAAAGATATTCGCCAAGAAATAGGTGCGAAATGAAGGAAAAAAAGGAGTTGATGTTGTTCATCCTAAATGAAGAGGAGA

>Marker103977

CTCTGATACCAGTTCAATTCAGGCTGTCCACTTTTGATCTGTCTCTGAAGTGCTTCATACAGGTTGCCATTGGTCATGTAXXXXXXXXXXATTTCATCTGCATCGATTTCCACTTGATGGAATGATGCAATTTTCCATTGTGGAGCTCCTTGTCGGTCCCCTTCCCAACT

CTCTGATACCAGTTCAACTCAGGCTGTCCACTTTTGATCTGTCTCTGAAGTGCTTCATACAGGTTGCCATTGGTCATGTAXXXXXXXXXXATTTCATCTGCATCGATTTCCACTTGATGGAATGATGCAATTTTCCATTGTGGAGCTCCTTGTCGGTCCCCTTCCCAACT

>Marker103995

CGACTTATCAAAAGGAGCCCATTTAGACAATGGTTTCCCTTTTATTTATATAGAAAATAAAAGGAAAAATAGCGCAGAAAXXXXXXXXXXATATAATTTTAAACTTTCTCGAAAAAAGAATAGATAAACTGTGAAAGGCATAATAATAATAATAATGATAAAGAATTGTC

CGACTTATCAAAAGGAGCCCATTTGGACAATGGTTTCCCTTTTATTTATATAGAAAATAAAAGGAAAAATAGCGCAGAAAXXXXXXXXXXATATAATTTTAAACTTTCTCGAAAAAAGAATAGATAAACTGTGAAAGGCATAATAATAATAATAATGATAAAGAATTGTC

>Marker104251

GCCTTCTTGGATTCTTTGATGACACACATGAAAGAATGTTGGTCTATGAATTCATGAGCAATGGCACTCTCCATGACCATXXXXXXXXXXCTCTCGTTAATGGGACCAGATGATGGGGAGTCACATCTGTCACTACGTGCAGCAGGGACAGTAGGATATATGGATCCAGA

GCCTTCTTGGATTCTTTGATGACACACATGAAAGAATGTTGGTCTATGAATTCATGAGCAATGGCACTCTCCATGACCATXXXXXXXXXXCTCTCGTTAATGGGACCAGATGATGGGGAGTCACATCTGTCACTACGTGCAGCAGGGACGGTAGGATATATGGATCCAGA

>Marker104263

ATGATTTCAGATACTAAAAAAAAGTGTTTGGCATTAGGAATCAAAATAAAGTAAGTTGAAATTTGATGTTTTCTCTGTTTXXXXXXXXXXATTTGTTGGTCTTGACTTTAAAATAACCCTTCGACTAAAGTTGGGTGTGGACCTCGAAGCAAACAAAATACTCTGCATAT

ATGATTTCAGATACTAAAAAAAAGTGTTTGGTATTAGGAATCAAAATAAAGTAAGTTGAAATTTGATGTTTTCTCTGTTTXXXXXXXXXXATTTGTTGGTCTTGACTTTAAAATAACTCTTCGACTAAAGTTGGGTGTGGACCTCGAAGCAAACAAAATACTCTGCATAT

>Marker104349

TTTTGGGGTGAAACCAAAAGCATAACTTTCATGTTGAAATTCGAACTCAGTTAAGTGGTGCTCTTAAAAGCTTACAGAGTXXXXXXXXXXTTGTATCTTATGCCCCAAACAACATTTGTTTCACATTCCTCCCAAAATAATTGGTTATACTTGCTTCGTCCAAGATGTTC

TTTTGGGGTGAAACCAAAAGCATAACTTTCATGTTGAAATTCGAACTCAGTTAAGTGGTGCTCTTAAAATCTTACAGAGTXXXXXXXXXXTTGTATCTTATGCCCCAAACAACATTTGTTTCACATTCCTCCCAAAATAATTGGTTATACTTGCTTCGTCCAAGATGTTC

>Marker104448

TACGATTTACTTTATCTAGAAGGAGTGTAATCATCGTCTCCATGGAGGTCAGGCTCGTGAGCCAATGGTTCTGTTTCTGCXXXXXXXXXXCTTTTGATTTGGCTCGTGCCTTTCTTTGGTCTTTTAAAATTTTGTCATGTCTATGTGAGCGTTATCCATTCTTGTGCCTA

TACGATTTACTTTATCTAGAAGGAGTGTAATCATCGTCTCCATGGAGGTCAGGCTCGTGAGCCAATGGTTCTGTTTCTGCXXXXXXXXXXCTTTTGATTTGGCTAGTGCCTTTCTTTGGTTTTTTAAAATTTTGTCATGTCTATGTGAGCGTTATCCATTCTTGTGCCTA

>Marker104468

ACTTAATCTTTTAACATAACAGATCGTGAATGTTTAATTATGTTAGATGATAATTTTCGAATATCTATCTATTATTACCTXXXXXXXXXXTTTTTGAGTTTATGAATCTCCTACGCCATTTTAATATTACCTCTTCATATTGGAATATTTCTAAAGAAAAAAACTTATAT

ACTTAATCTTTTAACATAACAGATGGTGAATGTTTAATTATGTTAGATGATAATTTTCGAATATCTATCTATTATTACCTXXXXXXXXXXTTTTTGAGTTTATTAATCTCCTACGCCATTTTAATATTACCTCTTCATATTGGAATATTTCTAAAGAAAAAAACTTATAT

>Marker104517

ATCATTCTGTCATTCTTATTTTACATTCATTGTCTCAGTTAAAACCGGTTAAAATGGTCCCCTAGGATCTTAAAAGGTTCXXXXXXXXXXGTCACCTTTTTAGTTTATCTTCTAATAATTATTAAAAATAAAGATAAAAGCCTTTTAGTTTAAATACAAATTAGATTTGG

ATCATTCTGTCATTCTTATTTTACATTCATTGTCTCAGTTAAAACCGGTTCAAATGGTCCCCTAGGATCTTAAAAGGTTCXXXXXXXXXXGTCACCTTTTTAGTTTATCTTCTAATAATTATTAAAAATAAAGATAAAAGCCTTTTAGTTTAAATACAAATTAGATTTGG

>Marker104790

CACTTCTCACTAATCTTGGGAGTGTGGAGAAGATTCTCCCATTCTAAAGAGTTTTTAAGTTATTGAGCTTTTTGTTTGAAXXXXXXXXXXACTCATGACAAACTTGACGTTTAATTCATACAATTAATTATAAGTGTGACAATGATGTTTTTCTAATAAAATTTGAAGGA

CACTTCTCACTAATCTTGGGAGTGTGGAGAAGATTCTCCCATTCTAAAGAGTTTTTAAGTTATTGAGCTTTTTGTTTGAAXXXXXXXXXXACTCATGACAAACTTGACGTTTAATTCATACAATTAATTATAAGTGTGAGAATGATGTTTTTCTAATAAAATTTGAAGGA

>Marker105063

CTATATAATCAAAAGCCAAATTTATAAGAGTAATATATAAAGCTCGCGTGATACGTGTCAAACATATACATATAGTAATAXXXXXXXXXXGATGGACAAAACCAACTTTGACACTTCAAATGTGTCAAGTAAAAATGAATAATACAATTTCACAAAATGTGTTTGAAATT

CTATATAATCAAAAGCCAAATTTATAAGAGTAATATATAAAGCTCGCGTGATACGTGTCAAACATATACATATAGTAATAXXXXXXXXXXGATGGACAAAACCAACTTTGACACTTCTAATGTGTCAAGTAAAAATGAATAATACAATTTCACAAAATGTCTTTGAAATT

>Marker105110

CCTTCAGAACTTGATCCCGAACAAAGTGAACATCTATTTCAATATGCTTCTTATGAGCATGAAACACTGGATACATTGCTXXXXXXXXXXTTCGATGCCCAGTCATCTCGGAATATGCAAAAATAGTCGGATCATCACCATGTTGAAACATTATTAAACCAAAGGATTTT

CCTTCAGAACTTGATCCCGAACAAAGTGAACATCTATTTCAATATGCTTCTTATGAGCATGAAACACTGGATACATTGCTXXXXXXXXXXTTCGATGCCCAGTCATCTCGGAATATGCAAAAATAGTCGGATTATCACCATGTTGAAACATTATTAAACCAAAGGATTTT

>Marker105162

AAGGTAAGAGAATTCGAGTTCATCATTTCAAAATTCAAACATAAACCATGGCATGAAATCCTTCATTAACTTTGTTGTTTXXXXXXXXXXAAGCTTAAGTTTATGGATTTTGAATTCAATAAATCGTGTTAGCTAGACGTTCAATCACGTTAACAAAAGACATGGCTCAA

AAGGTAAGAGAATTCGAGTTCATCATTTCAAAATTCAAACATAAACCATGGCATGAAATCCTTCATTAACTTTGTTGTTTXXXXXXXXXXAAGCTTCAGTTTATGGATTTTGAATTCAATAAATCGTGTTAGCTAGACGTTCAATCACGTTAACAAAAGACATGGCTCAA

>Marker105169

AGTCTTTTATTTCTCTCAATTAAAGATTTGTTCTTATATAAAAAATTAAAAGTCAAATGCTAAAGGAAAACACTGACCGTXXXXXXXXXXATGAGTAGAGTATATACAGAGAGAATGAAACTCAAACCTTAGATAGAGAGCACAAGAGCTTTGCCCAAGTAAATAATCAG

AGTCTTTTATTTCTCTCAATTAAAGATGTGTTCTTATATAAAAAATTAAAAGTCAAATGCTAAAGGAAAACACTGACCGTXXXXXXXXXXATGAGTAGAGTATATATAGAGAGAATGAAACTCGAACCTTAGATAGAGAGCACAAGAGCTTTGCCCAAGTAAATAATCAG

>Marker105191

AATAATGCAGCTCCATCAGATTTGACAAGATCCATTATATTAGGAGTTCTTGACACAATCCCTAAAGGAGCATCACGCATXXXXXXXXXXCTCTTATGTTTTTGCAAAGAAGCATCATTGTTGTTGCTTTGATCAAAATTCTCTTCTTCTTCTTCTTCATTTACCACAAT

AATAATGCAGCTCCATCAGATTTGACAAGATCCATTATATTAGGAGTTCTTGACACAATCCCTAAAGGAGCGTCACGCATXXXXXXXXXXCTCTTATGTTTTTGCAAAGAAGCATCATTGTTGTTGCTTTGATCAAAATTCTCTTCTTCTTCTTCTTCATTTACCACAAT

>Marker105674

ATATTGTCCAAAGGTTGGCTAAACCTGAAAGTGTTTTGTAAAGATCGAAATTATTGTTTTCAAATATAGATATTTTCTTCXXXXXXXXXXTGTAAAATAAGATTTGGTTCCATAATCTTTTACCTTTTTGAAAAAGTATTTCTCTCCATTTCCAAATTTGTTGTAGTTTG

ATATTGTCCAAAGGTTGGCTAAACCTGAAAGTGTTTTGTAAAGATCGAAACTATTGTTTTCAAATATAGATATATTCTTCXXXXXXXXXXTGTAAAATAAGATTTGGTTCCATAATCTTTTACCTTTTTGAAAAAATATTTCTCTCCATTTCCAAATTTGTTGTAGTTTG

>Marker105912

AATCAAATTCAAATTCAATTATGAAATGCAACTACATCACTAATAGTCAACTACATATAATATTAGTGATAAGAGTTTTTXXXXXXXXXXACCATAAAATATCTGTAAAAACAGCTAATGCTCAACATTCAACTAGTAATTTTGTTTAAAATGTCAAATCCCCATTAAAT

AATCATATTCAAATTCAATTATGAAATGCAACTACATCACTAATAGTCAACTACATATAATATTAGTGATAAGAGTTTTTXXXXXXXXXXACCATAAAATATCTGTAAAAACAGCTGATGCTCAACATTCAACTAGTAATTTTGTTTAAAATGTCAAATCCCCATTAAAT

>Marker106228

ACCACTTCTCTTAGCCTTCATTTAGTTAGAAAAAAAACTAAAAGTTCTAAAACATCCATTTTGTTTTTTCTTCAAGCAACXXXXXXXXXXGCGACTTGGACAGTTTTGATGCCTCTAAGTGAAATGCAAAATGACTAAGGCTTAACTTAACTTGATCTTAAACAACTTAA

ACCACTTCTCTTAGCCTTCATTTACTTAGAAAAAAAACTAAAAGTTCTACAACATCCATTTTGTTTTTTCTTCAAGCAACXXXXXXXXXXGCGACTTGGACAGTTTTGACGCCTCTAAGTGAAATGCAAAATGACTAAGGCTTAACTTAACTTGATCTTAAACAACTTAA

>Marker106357

AAGCAACTTCCTCCTCCTTCACAAATTAATGGATCTTCTTCATCTTCAGATAATCTTCCTGTTTCTTCTCAGAGAATCAAXXXXXXXXXXCTACTGAGTCTGCTTTCAATTTAGTTATTGATGGAGTTTTGTTGTTTTAGGAACTGATTTTTGAGTTGGGGATTTACTAT

AAGCAACTTCCTCCTCCTTCACAAATTAATGGATCTTCTTCATCTACAGATAATCTTCCTGTTTCTTCTCAGAGAATCAAXXXXXXXXXXCTACTGAGTCTGCTTTCAATTTAGTTATTGATGGAGTTTTGTTGTTTTAGGAACTGATTTTTGAGTTGGGGATTTACTAT

>Marker106523

ATAATTTTATGCTCTACAAATTGATAGTATTATCTGCATCTTTAGGGAAAAAACCAGCAGAACATAATGGTGAATTTATGXXXXXXXXXXTTATTTTCTATAAGAAGTCTCTTTTATAGTCTAAATTTCAACCTTTGGAGTTAAACACAAGTGAGTTTGTGATCATTAAT

ATAATTTTATGCTCTACAAATTGATAGTATTATCTGCATCTTTAGGGAAAAAACCAGCAGAACATAATGGTGAATTCATGXXXXXXXXXXTTATTTTCTATAAGAAGTCTCTTTTATAGTCTAAATTTCAACCTTTGGAGTTAAACACAAGTGAGTTTGTGATCATTAAT

>Marker106999

TTTAAATCTACGAATTATCCTATAATGAGGGGCGTAGTCAGAATTAATATAATTAAATCATGCCCATGACAAGTTGTTCTXXXXXXXXXXGAGTAAATAATCTATAGTATACGAATACGATTGGGCACCTTATTTTGGGGACATTACGGATGCGACCCGCTTTATAGTTA

TTTAAATCTACGAATTATCCTATAATGAGGGGCGTAGTCAGAATTAATATAATTAAACCATGCCCATGACAAGTTGTTCTXXXXXXXXXXGAGTAAATAGTCTACAGTATACGAATACGATTGGGCACCTTATTTTGGGGACATTATGGATGCGACCCGCTTTATAGTTA

>Marker107108

AATAATAAAGTAGAGACTGATTTTCTCTATTCGATGATCGAAACATACTATGACCTATTTGTGATGGTATAAGTCAAAAGXXXXXXXXXXCATATGAGAAAACTATTCTATAATTATCTTAGCCATCTAGACCAATTCAATCACACGAGAGAGTATGTGCATTTTTCTTT

AATAATAAAGTAGAGACTGATTTTCTCTATTCGATGATCGAAACATACTATGACCTATTTGTGATGGTATAAGTCAAAAGXXXXXXXXXXCATATGAGAAAACTATTCTATAATTATCTTAGCCATCTAGACCAATTCAATCACAGGAGAGAGTATGTGCATTTTTCTTT

>Marker107136

TTTGCAGCCCTCGGATGTCCCGATCCAAGTTCGTTTGATACTCGAACGCTGAACAAGAAGAGAGAATGAAAAACTTCCTTXXXXXXXXXXTTTCAAAATTGGAGAGGGATGTGTGAAAACTAATTACCAAATAGAGAGTGAACCAACAGCAATAATAGGATCATCAAGGT

TTTGCAGCCCTCGGATGTCCCGATCCAAGTTCGTTTGATACTCGAACGCTGAACAAGAAGAGAGAATGAAAAACTTCCTTXXXXXXXXXXTTTCAAAATTGGAGAGGGATGTGTGAAAATTAATTACCAAATAGAGAGTGAACCAACAGCAATAATAGGATCATCAAGGT

>Marker107277

ATTGAAGAAATATATGGTGTTGGTCCCGTAGTTTTTTGAAAGAATGCACCAGTTGGGGTTGAGGTTAAGGGGAGGAAGGGXXXXXXXXXXCCATTTTGGTTTAAGTGGAGTATTTTCTTTTTCCTAGTGGTTGATGGGTGCATGACTGGCATAGCTTGTTTATTGTGGGT

ATTGAAGAAATATATGGTGTTGGTCCCGTAGTTTTTTGAAAGAATGCACCAGTTGGGGTTGAGGTTAAGGGGAGGAAGGGXXXXXXXXXXCCATTTTGGTTTAAGTGGAGTATTTTCTTTTTCCTAGTGGTTGATGGGTGCATGACTGGCATACCTTGTTTATTGTGGGT

>Marker107583

TCCTTCTAGCGTTTGTTTTTCAATCTTCATGGATGACCGATGATAGACCAAATCGTTGATCTCTAAAGAGAAAAAATCATXXXXXXXXXXCGCTAGTCTCACGTTCCATCTGACATTTGAATGTTCAAACCTAAATCAACTTTTTAAGAGGTAACATTTCTTAAAAATGC

TCCTTCTAGCGTTTGTTTTTCAATCTTCATGGATGACCGATGATAGACCAAATCGTTGATCTCTAAAGAGAAAAAATCATXXXXXXXXXXCGCTAGTCTCACGTTCGATCTGACATTTGAATGTTCAAACCTAAATCAACTTTTTAAGAGGTAACATTTCTTAAAAATGC

>Marker107624

TTCCGTGTTTTTGCGTGTCTAATAGATTCATAAATTTTAAACATATCTTTAATGGTTGTGAGTAGACAGTTTTGTGACAAXXXXXXXXXXCCCTGGGCTCTGCTCGTCGGAAGAGGGAGCTCAACTTCTAAAATCAAAGCTCCAGATGAGTTGACGAGACTAAAGGCGGA

TTCCGTGTTTTTGCGTGTCTAATAGATTCATAAATTTTAAACATATCTTTAATGGTTGTGAGTAGACAGTTTTGTGACAAXXXXXXXXXXCCCTGGGCTCTGCTCGTCGGAAGAGGGAGCTCAACTCCTAAAATCAAAGCTCCAGATGAGTTGACGAGACTAAAGGCGGA

>Marker107711

TCTTTTACTCTGCACCCGCTTCAGACTGGTTAAGGAGACGAAGGCGGCGACCAATAGAATGAAAAGTCATCGGCTCGACTXXXXXXXXXXAATTTTTTATTCATTGATCTTCATTTCCTTTGACCCTTTTAGCTTTCAAATCCGATCAGCCCTCTTAGCTCTAGGAAATG

TCTTTTACTCTGCACCCGCTTCAGACTGGTTAAGGAGACGAAGGCGGCGACCAATAGAATGAAAAGTCATCGGCTCGACTXXXXXXXXXXAATTTTTTATTCATTGATCTTCATTTCCTTTGACCCTTTTAGCTTTCAAATCCGATCAGCCCTCTTAGCTGTAGGAAATG

>Marker107907

AATCAAATACCAACAAACTTCAAGAGTAAATTCTTTTTCTTTCCCTATAAAGACCCCTCCCCAAAAGAAAAAAAAGTAATXXXXXXXXXXACAACAACATGAACATAACTATAACACCTCCTGCTTTAATATTATTAACAATATTCAACCTCATCAAGTTCTTATGGTCA

AATCAAATACCAACAAACTTCAAGAGTAAATTCTTTTTCTTTCCCTCTAAAGACCCCTCCCCAAAAGAAAAAAAAGTAATXXXXXXXXXXACAACAACATGAACATAACTATAACACCTCCTGCTTTAATATTATTAACAATATTCAACCTCATCAAGTTCTTATGGTCA

>Marker107946

TTCAAGCTAATGAAGAAAACTTATGGTTTGGTGCCAGAGATGGAACATTATGCCTGTATCGTGGATCTCTTAAGTCGTGTXXXXXXXXXXAGATGGACTTAGTTTGCGGCATTTGATGAAAGAGCAGGGGGTAAAAAAGGAACCCGGATGTAGTTGGATCTCTGTGAATG

TTCAAGCTAATGAAGAAAACTTATGGTTTGGTGCCAGAGATGGAACATTATGCCTGTATCGTGGATCTCTTAAGTCGTGTXXXXXXXXXXAGATGGACTTAGTTTGCGGCATGTGATGAAAGAGCAGGGGGTAAAAAAGGAACCCGGATGTAGTTGGATCTCTGTGAATG

>Marker108372

AAAAGGCATCTTAAAATCTTCATAACTCATTAATATTTTCACCTAACAATTAATTAATAAACTCTTTGTTGCACAACCAAXXXXXXXXXXTCCATTACGTATATGAAATATATCTCTTTAAAATCTTACTAGAAAAACCTAATGAAAACAAATACAGGGAAGAAAAAAGA

AAAAGGCATTTTAAAATCTTCATAACTCATTAATATTTTCACCTAACAATTAATTAATAAACTCTTTGTTGCACAACCAAXXXXXXXXXXTCCATTACGTATATAAAATATATCTCTTTAAAATCATACTAGAAAAACCTAATGAAAACAAATACAGGGAAGAAAAAAGA

>Marker108766

AAGCCCACTAAATGTAACATTATTTTTGCTAAGTATACTTTTCATATTAGTTGGCAAATATATGGGTGATTCCAATGCTCXXXXXXXXXXTCAACATAAACAATCAATACTGCAACCTTCCCAGCCTCAGAGATCTTTGTAAACAAAATATGGTCAGATTGCCTCTGATT

AAGCCCACTAAATGTAACATTATTTTTGCTAAGTATACTTTTCATATTAGTTGGCAAATATATGGGTGATTTCAATGCTCXXXXXXXXXXTCAACATAAACAATCAATACTGCAACCTTCCCAGCCTCAGAGATCTTTGTAAACAAAATATGGTCAGATTGCCTCTGATT

>Marker109071

TCGGTGGGAAGCCTTTTAGAGGAATTCGCAGCAAGTCAAGGAGCCCCTTACATCAAATTTACTTTAGAGTTAGAGCTGGGXXXXXXXXXXTGTTGAAGGATGTTTGCTTTCGAATATAATGCTTGAGGATTTTCGGGGCTTGAAAAAATTTGTTACCTTTTGCTAATTTA

TCGGTGGGAAGCCTTTTAGAGGAATTCGCAGCAAGTGAAGGAGCCCCTTACATCAAATTTACTTTAGAGTTAGAGCTGGGXXXXXXXXXXTGTTGAAGGATGTTTGCTTTCGAATATAATGCTTGAGGATTTTCGGGGCTTGAAAAAATTTGTTACCTTTTGCTAATTTA

>Marker109173

AACTACTGTTAAACACATGTTTAATGCACCAACATGAACCGGAGTGTCAAACTAAAGTGTCACTGTTTCATTGAGTCAAAXXXXXXXXXXAGTAACTAGTGTCTTTATCCACTTAACTATGCTCGGATTAACATTTCAATCTCACTCTTTTGAAAAAAAAAACAGAGAAA

AACTACTGTTAAACACATGTTTAATGCACCAACATGAACCGGAGTGTCAAACTAAAGTGTCACTGTTTCATTGAGTCAAAXXXXXXXXXXAGTAACTAGTGTCTTTATCCACTTAACTATGATCGGATTAACATTTCAATCTCACTCTTTTAAAAAAAAAAACAGAGAAA

>Marker109466

ACAAAAACTTAAGAACCTGAATTCAATTTCTCTCTATCTAATTCTTCTTCTTCTTCTTCTTTCATTAAAAATGAGGAGTTXXXXXXXXXXAGATTGTGTCTGCAACCTTCAATCTCTTACACTAACTATACCATACAGAGTTGAAGCATGGAATGTGTTCAAAACATTAT

ACAAAAACTTAAGAACTTGAATTCAATTTCTCTCTATCTAATTCTTCTTCTTCTTCTTCTTTCATTAAAAATGAGGAGTTXXXXXXXXXXAGATTGTGTCTGCAACCTTCAATCTCTTACACTAACTATACCATACAGAGTTGAAGCATGGAATGTGTTCAAAACATTAT

>Marker109693

ATTCGCCTCACCACTTTTCTCTATAAGATCACTCCTACAGATTTATCAGAGAGATTAAACAAGTTCTTCCATCCACCATCXXXXXXXXXXATTTATCCATTATCATCAAAGGAAAAATGTGAAGCCCCAAGTGACAAAAACTTATAGTAGGAAGGGCAGAAAGGGAACTG

ATTCGCCTCACCACTTTTCTCTATAAGATCATTCCTAGAGATTTATCAGAGAGACTAAACAAGTTCTTCCATCCACCATCXXXXXXXXXXATTTATCCATTATCATCAAAGGAAAAATGTGAAGCCCCAAGTGACAAAAACTTATAGTAGGAAGGGCAGAAAGGGAACTG

>Marker109749

TCGAAAACTTTTACAAATGAGAAGCCATTGTATGATAAAGACACGATTCTCAACTATATCCTATCAAACTTCTTCATATAXXXXXXXXXXGGCTGATAGGTCCATTGTTGTTGTTTGTTATTAAATGAGTTGAAGATGGCGTAGAGAAGATAAGAGAAGAGGTTGATGAT

TCGAAAACTTTTACAAATGAGAAGTCATTGTATGATAAAGACACGATTCTCAACTATATCCTATCAAACTTCTTCATATAXXXXXXXXXXGGCTGATAGGTCCATTGTTGTTGTTTGTTATTAAATGAGTTGAAGATGGCGTAGAGAAGATAAGAGAAGAGGTTGATGAA

>Marker109965

CATGATGTAGTTGATGATGAAGGATTGTGGAAAAAGAAACTCACTAGTCAGTCTCTTGAACTTTTTGAGTTTTTACCTCAXXXXXXXXXXCTAGAATAGAAACAGAGAAAATGCTTATTCAGATGGTTGAAACTGAGTTGGAGAAGAGAAAGAGCGAAGGTGCATACAAA

CATGATGTAGTTGATGATGAAGGATTGTGGAAAAAGAAACTCACTAGTCAGTCTCTTGAACTTTTTGAGTTATTACCTCAXXXXXXXXXXCTAGAATAGAAACAGAGAAAATGCTTATTCAGATGGTTGAAACTGAGTTGGAGAAGAGAAAGAGCGAAGGTGCATACAAA

>Marker110049

CTACAGTCTTGATTTCTCATGAAGTGCCTTTTTCGCACTCTTTGAACTTTTTAGTGGGTCATAAGTTACGAACATGCACTXXXXXXXXXXTATACTTCAGCATTGTTAGGTCATTTTAAACACTTTTTAGGTGTGTTTGAAATTAAGTTTGTTACACTTACACACTTGAT

CTACAATCTTGATTTCTCATGAAGTGCCTTTTTCGCACTCTTTGAACTTTTTAGTGGGTCATAAGTTACGAACATGCACTXXXXXXXXXXTATACTTCAGCATTGTTAGGTCATTTTAAACACTTTTTAGGTGTGTTTGAAATTAAGTTTGTTGCACTTACACACTTGAT

>Marker110175

CCAATCCCCTTAAGCAACAGACTTCCATTAGATATAACTGTAGATATGAAATCCTGGAATTAGTCACTTTGTATATTCTAXXXXXXXXXXAAGGAAAGTAATACAAAGGGTAGTAAAAATGGAATTAACCTAGATTAACCACAAATCCTCCCCACACATCAAATTGCTTT

CCAATCCCCTTAAGCAACAGACTTCCATTAGATATAACTGTAGATATGAAATCTTGGAATTAGCCACTTTGTATATTCTAXXXXXXXXXXAAGGAAAGTAATACAAAGGGTAGTAAAAATGGAATTAACCTAGATTAACCACAAATCCTCCCCACACATCAAATTGGTTT

>Marker110187

ATTACCTAGAAAGTAATATTCATAATATGTGTAACAGAAAATGTTCATAGACCACAAATCAACAAGAACCCGTATCAAATXXXXXXXXXXATAAATCCCCAAAACAACTTTAAACAAAATACACATAAAATGAAATAGTTTTTTTGACAGGAATTGATGGTCCCTAATAG

ATTACCTAGAAAGTAATATTCATAATATGTGTAACAGAAAATGTTCATAGACCACAAATCAGCAAGAACCCGTATCAAATXXXXXXXXXXATAAATCCCCAAAACAACTTTAAACAAAATACACATAAAATGAAATAGTTTTTTTGACAGGAATTGATGGTCCCTAATAG

>Marker110294

AAATTATTATGTTTAGACAATTAAACTTTTCCTTCTTCGCCATGCTTATAATGAGTTCATATTCCATTGTTCCATCTCGCXXXXXXXXXXCGCTTACACTTGGACGATTGAGGGAAAAATTTGCGCTATAATATTTATTTTTATCAATTTAGCAATGCATGAGTAAGAGT

AAATTATTATGTTTAGACAATTAAACTTTTCCTTCTTCGCCATGCTTATAATGAGTTCATATTCCATTGTTCCATCTCGTXXXXXXXXXXCGCTTACACTTGGACGATTGAGGGAAAAATTTGCGCTATAATATTTATTTTTATCAATTTAGCAATGCATGAGTAAGAGT

>Marker110366

TCAATTTGTTGATTTTTCAACACTGCACAAAAATTGAGATGAACAAATCAACGACGCCAGTTGGGGAGGAAGAGAGCTATXXXXXXXXXXGGTTTAAAGCTTTTTGTAAATGTTTTGATACTGGTATTAGAGCTTTGGAAGCTGTTTTAGGATATTATTAAAACTATCAT

TCAATTTGTTGATTTTTCAACACTGCACAAAAATTGAGATGAACAAATCAACGACGCCAGTTGGGGAGGAAGAGGGCTATXXXXXXXXXXGGTTTAAAGCTTTTTGTAAATGTTTTGATACTGGTATTAGAGCTTTGGAAGCTGTTTTAGGATATTATTAAAACTATCAT

>Marker110428

ATACCTTCGTATTTATTATACATCCTCTACTTCCAAGTTGTTAAAATTTATTTAAAATCTTTATATAATCATTTCTAGGTXXXXXXXXXXTTTTTTTTTTAAAATAGAAAATATATAGTAATTTTGGTGTTTTCCTTTTCTGTAGCATTAAATATCATTAAAACAAGTAG

ATACCTTCGTATTTATTATACATCCTCTACTTCCAAGTTGTTAAAATTTATTTAAAATCTTTATATAATCATTTCTAGGTXXXXXXXXXXTTTTTTTTTTTAAATAGAAAATATATAGTAATTTTGGTGTTTTCCTTTTCTGTAGCATTAAATATCATTAAAACAAGTAG

>Marker110541

AAATACTCATACTGAAGAAAGAAAGAAAAAAACCCTCGAACAAACTCCCGTGAAAAAGGGGAGGGAAGGGATAGATATCAXXXXXXXXXXTACTTCGATAACAACTAATTGTGGAAGTTTCAGTAGTTTTTTTTTACCTTCATGAACATTAAGGAAAGAACCAATTCCAT

AAATACTCATACTGAAGAAAGAAAGAAAAAAACCCTCAAACAAACTCCCGTGAAAAAGGGGAGGGAAGGGATAGATATCAXXXXXXXXXXTACTTCGATAACAACTAATTGTGGAAGTTTCAGTAGTGTTTTTTTACCTTCATGAACATTAAGGAAAGAACCAATTCCAT

>Marker110902

TTTGGATTCCTTTAACTCCACGTGTCCCCTTATAAAATAAATAATATTATCTCTGCTCTAAAAGAAATTGTTTTCGCTTGXXXXXXXXXXATATGCTTTTAATTATATTTTCTATCTCAAAAGTTGTTTTAGGAAAAAGAATTATGAAATCCCCTCTCCTTTCTTATAAA

TTTGGATTCCTTTAACTCCACGTGTCCCCTTGTAAAATAAATAATATTATCTCTGCTCTAAAAGAAATTGTTTTCGTTTGXXXXXXXXXXATATGCTTTTAATTATATTTTCTATCTCAAAAGTTGTTTTAGGAAAAAGAATTATGAAATCCCCTCTCCTTTCTTATAAA

>Marker111204

ATTACACAGCATTTGTCCATGTAAGTGATGTTACTCTTTCTTTTCCCAAGATATCTCACCATTTCTTCTGGTTTTCTACCXXXXXXXXXXTTGGCAGAAGAAATGACAGGGCAATGATTTCTGCCTCAATTCAGTTGAATATGTAATCATATAGACAGTTGAACCTTGAA

ATTACACAGCATTTGTCCATGTAAGTGATGTTACTCTTTCTTTTCCCAAGATATCTCACCATTTCTTCTGGTTTTCTACCXXXXXXXXXXTTGGCAGAAGAAATGACAGGGCAATGATTTCTGCCTCAATTCAGTTGAATATGTAATCATATAGACAATTGAACCTTGAA

>Marker111487

TTGAAAATTTTTAATACAAACATTTATTATAAAAAGAAATTAGGGTTTAGGCTAATCTTCAGTGACTCTCGACCAATCTTXXXXXXXXXXATCAATCTTGAAAAAAATTAATACAAATATTTTTAGTATATATAACAAACCAAATAACTTTATATACAAATATTTTTCTA

TTGAAAAATTTTAATACAAACATTTATTATAAAAAGAAATTAGGGTTTAGGCTAATCTTCGGTGACTCTCGACCAATCTTXXXXXXXXXXATCAATCTTGAAAAAAATTAATACAAATATTTTTAGTATATATAACAAACCAAATAACTTTATATACAAATATTTTTCTA

>Marker111701

CTCATCATTCACCAGGATACGGTCTAGCCTCCTTATCAAACCAGACCCATGGACCTTACTAGTCCAAGTAAACCAGTTTTXXXXXXXXXXTTACCAAACCAGAGATAAATTGATATGACACCTCACTAGGCGTAAACATGAATCTATTACGTTTCCACAAACCCACATAT

CTCATCATTCACCAGGATACGATCTAGCCTCCTTATCAAACCAGACCCATGGACCTTACTAGTCCAAGTAAACCAGTTTTXXXXXXXXXXTTACCAAACCAGAGATAATTTGATATGACACCTCACTAGGCGTAAACATGAATCTATTACGTTTCCACAAACCCACATAT

>Marker112052

GCCGCCGAAGCAAAGAGGACTAAAATGGCAAACAAATATAGAGACGATGGTGCTGCCATAGTGCCAAAATGTTTGTTTTCXXXXXXXXXXAGAAAAAAGAAAAAAAGATTAATAAGGAGGGGAAACAAAAATGCTTAGTTCAACATTATTGAAAAGTTGTTACACCCACT

GCCGCCGAAGCAAAGAGGACTAAAATGGCAAACAAATATAGAGACGATGGTGCTGCCATAGTGCCAAAACGTTTGTTTTCXXXXXXXXXXAGAAAAAAGAAAAAAAGATTAATAAGGAGGGGAAACAAAAATGCTTAGTTCAACATTATTGAAAAGTTGTTACACCCACT

>Marker112516

GTTGGACAATTGGTCAGTCTAGCCATTGAAATGATATTTAAAAATGTTTTCGATTTGAATTTTTTTCAAACATTCACCGAXXXXXXXXXXTTAATATACATACATAAGGGACCAAATAAATGTATCTATTCCTCCAGATTCAAAGATGAAAAACATGTTTTTAAGTTTGT

GTTGGACAATTGGTCAGTCTAGCTATTGAAATGATATTTAAAAATGATTTCGATTTGAATTTTTTTCAAACATTCACCGAXXXXXXXXXXTTAATATACATACATAAGGGACCAAATAAATGTATCTATTCCTCCAGATTCAAAGATGAAAAACATGTTTTTAAGTTTGT

>Marker112596

AAACAAGATTATGCCCTACTGCTATAACATAAATTGTGCATGCAAAGCTTGAATCAGCCAATTGAGATTTTACCGCATATXXXXXXXXXXCGCCAAAAATATAAATATAATGTCTCTAGTCACAAAAAATCGAAATTTCAGGGTTCGCCATTTTCTGTCAGCAACAGCAT

AAACAAGATTATGCCCTAATGCTATAACATAAATTGTGCATGCAAAGCTTGAATCAGCCAATTGAGATTTTACCGCATATXXXXXXXXXXCGCCAAAAATATAAATATAATGTCTCTAGTCACAAAAAATCGAAATTTCAGGGTTCGCCATTTTCTGTCAGCAACAGCAT

>Marker112854

CCATGCATCTTCAATTTAAATATTGTGTGTGAACTTTAACTGCTCAACTGAGATTACAACTATATTCAACTTCTAATATAXXXXXXXXXXAAAGCATATCGATCTTTTAAACGATTACATTTTATATAGAATATCTAAATCTAGTAAGTCGAGGTGCACGTGCTAGTTTT

CCATGCATCTTCAATTTAAATATTGTGTGTGAACTTTAACTGCTCAACTGAGATTACAACTATATTCAACTTCTAATATAXXXXXXXXXXAAAGCATATCGATCTTTTAAACGATTACATTTTATATAGAATATCTAAATCTAGTAAGTCGGGGTGCACGTGCTAGTTTT

>Marker112960

TATCTTCTTTGGCTGTAATTTACTGTATTTTCCAGCTGGAAGTCTTGTTTTTCAAAGATATATCATGACTAGATCTCCTTXXXXXXXXXXATACGATTGTAGGCGAACTCGGCTTGTGCTAAGATGGGATCTCATGCTCATTCACAATGATCCTACACCACAAGTATTCA

TATCTTCTTTGGCTGTAATTTACTGTATTTTCCAGCTGGAAGTCTTGTTTTTCAAAGATATATCATGACTAGATCTCCTTXXXXXXXXXXATATGATTGTAGGCGAACTCGGCTTGTGCTAAGATGGGATCTCATGCTCATTCACAATGATCCTACACCACAAGCATTCA

>Marker112970

CAGCAATTTTCATATGGAACTTTCGGTTTTTTTCAATGAATATTGTGTTTAAATAATTGCTACAATAGTATCTCTCTAAAXXXXXXXXXXAAAAATTGAATTTCAATGAATAGTTAGCTGAATTCGCCAATCTCTGGAACCAGAATCATGATCGAGGTTTATGTAGACGA

CAGCAATTTTCATATGGAACTTTCGGTTTTTTTGAATGAATATTGTGTTTAAATAATTGCTACAATAGTATCTCTCTAAAXXXXXXXXXXAAAAATTGAATTTCAATGAATAGTTAGCTGAATTCGCCAATCTCTGGAACCAAAATCATGATCGAGGTTTATGTAGACGA

>Marker112985

ATTGCTGACTTCGCTGGTTCAAGAGATCCTTATCAACGACGATGGCTTCGATTCCTCTTTTTTCTCAACACTTCACAGTCXXXXXXXXXXAAAAATAATATAAAATTCCAAGTGGAATAATGAAGTTGAAAAGTTGATTGCATTAGCATCCACGTCGTATCGCGTTAAAG

ATTGCTGACTTCGCTGGTTCAAGAGATCCTTATCAACGACGATGGCTTCGATTCCCCTTTTTTCTCAATACTTCACAGTCXXXXXXXXXXAAAAATAATATAAAATTCCAAGTGGAATAATGAAGTTGAAAAGTTGATTGCATTAGCATCCACGTCGTATCGCGTTAAAG

>Marker113466

CTCCTTTGCCTTTCTCCGCAAAGAGTGTCGACGTGAATTATGAACCCCGTGTGTCCCATCATTTATTTCTTCCCTACTTGXXXXXXXXXXACAGTATTTGCATTTGATCTTCTGGCGACCTCCATTGACCATAATTCCATGAGCCCAACCAGGATCAGAAGCTCGTGGAG

CTCCTTTGCCTTTCTCCGCAAAGAGTGTCGACGTGAATTCTGAACCCCGTGTGTCCCATCATTTATTTCTTCCCTACTTGXXXXXXXXXXACAGTATTTGCATTTGATCTTCTGGCGACCTCCATTGACCATAATTCCATGAGCCCAACCAGGATCAGAAGCTCGTGGAG

>Marker113563

ATATTAGAAATGTAGGGTCCATCATGCAATGGGTTTGGTTGGAACGATGAGGTGAAGTGCATGATCGCTAAGAAGGAATTXXXXXXXXXXTCGACGACATTGTGGGCACACTACCTGGTCACTCAAGTGAATGTAGAACTGGTTCAAGTGGATCAAAGAGGAAACGAGAA

ATATTAGAAATGTGGGGTCCATCATGCAATGGGTTTGGTTGGAACGATGAGGTGAAGTGCATGATCGCTAAGAAGGAATTXXXXXXXXXXTCGACGACATTGTGGGCACACTACCTGGTCACTCAAGTGAATGTAGAACTGGTTCAAGTGGATCAAAGAGGAAACGAGGA

>Marker113776

AACAAAACAACCGCATTAAAAAAGGGGAACCAACTAAGTAAAATATTGTCTATGGAATAGTTACAAAAGTTCTTCAAAATXXXXXXXXXXACACTGCCCTCGCAAACTTGCACTCCCAGAAAAGATGATCAAGATCTTCCTCTGCCTCCCGACAAAGCATGTAGCAAAAA

AACAAAACAACCGCATTAAAAAAGGGGAACCAACTAAGTAAAATATTGTCTATGGAATAGTTACAAAAGTTCTTCAAAATXXXXXXXXXXACACTGCCCTCGCAAACTTGCACTCCCAGAAAAGATGATCAAGATCTTCCTCCGCCTCCCGACAAAGCATGTAGCAAAAA

>Marker113899

TCCATGAACAAAATAACTGGCATCTATTGAATGGATTGAATGGACGTAGAACCATACAAACACCGATTTTATAATTAAATXXXXXXXXXXACAAAGAACAAGAGCACATGATATGAGAATAGAAACGAATAAGAGAAGAGCACCACGCGCTCACCACACCATAGGGTTGG

TCCATGAACAAAATAACTGGCATCTATTGAATGGATTGAATGGACATAGAACCATACAAACACCGATTTTATAATTAAATXXXXXXXXXXACAAAGAACAAGAGCACATGATATGAGAATAGAAACAAATAAGAGAAGAGCACCACGGGCTCACCACACCATAGGGTTGG

>Marker113922

TTGATATGGTTTGAGGCTTGCTTCTTAGTTTGTATAAGAATAGGATTTGCATGAATCAATTCAAAGTTTAAGAATTAAAAXXXXXXXXXXCTACTTTTTCATTAATGTGTCTGTCTCTTCCAGAGGGTGCCATGAAAAAAATTATATGAACTCTATTTGTGGGATTTCTT

TTGATATGGTTTCAGGCTTGCTTCTTAGTTTGTATAAGAATAGGATTTGCATGAATCAATTCAAAGTTTAAGAATTAAAGXXXXXXXXXXCTACTTTTTCATTAATGTGTCTGTCTCTTCCAGAGGGTGCCATGAAAAAAATTATATGAACTGTATTTGTGGGATTTCTT

>Marker113984

AAAAACTATCTCCCAAGGCACAAGCTAAGTCAGTATAGGAGCGGGAGTTAATGGCTGTGGTGCTCTCTGTGCCGAAATGGXXXXXXXXXXTGTCAAGAGTAGAACAACCACTAGAACTTAACATTATGACTACTACGGGGATTTTGAACATGGAGATAGTCAATGAAGAA

AAAAACTATCTCCCAAGGCACAAGCTAAGTCAGTATAGGAGCGGGAGTTAATGGCTGTGGTGCTCTCTGTGCCGAAATGGXXXXXXXXXXTGTCAAGAGTAGAACAACCACTAGAACTTAACATTATGACTACTACGGGGATATTGAACATGGAGATAGTCAATGAAGAA

>Marker114413

AAATAGAGAAGATAGCCTCTAAAGACTATGTGAGATAACCTCCAAAGAGATTATCTATGCGTTTGAGCAAGGACTTGACGXXXXXXXXXXAAGACAAGTATGTGACATGCTCTAAGCAAACAGTTTAAAGTTAATCTAGGTGTTCGAATATTGTTAACATACATTTTTGT

AAATAGAGAAGATAGCCTCTAAAGACTATGTGAGATAACCTCCAAAGAGATTATCTATGCGTTTGAGCAAGGACTTGACGXXXXXXXXXXAAGACAAGTATGTGACATGCTGTAAGCAAACAGTTTAAAGTTAATCTAGGTGTTCGAATCTTGTTAACATACATTTTTGT

>Marker115212

ATGGATTAAATTGACAAAAGCTACAGAATATTTAAAGTGAAGAATCTATACAGTTGCATCAAAATTATTGTTCACCAAGTXXXXXXXXXXCAAGGGGAATCATTAAAAGCTACTCCCAAGAAAATGAAAAGGGAGAAGAAAAAAAAAGTGCACGAATTGCAATGAAATGT

ATGGATTAAATTGACAAAAGCTACAGAATATTTCAAGTGAAGAATCTATACAGTTGCATCAAAACTATTGTTCACCAAGTXXXXXXXXXXCAAGGGGAATCATTAAAAGCTACTCCCAAGAAAAGGAAAAGGGAGAAGAAAGAAAAAGTGCACGAATTGCAATGAAATGT

>Marker115327

CATCTACACGTTGTTTAAAAGAGTTGAAATATATCAAGAATCAAACAAGAATTGGATTGCATATGAAATCGAAGGAGGAAXXXXXXXXXXTTACATGTTTGATGGGATGGAGCTCTTATACAAATGCCTAAGGGGCAAACTTTCAAATTTTGATTGTTTTTATCTCATTT

CATCTACACGTTGTTTAAAAGAGTTGAAATATATCAAGAATCAAACAAGAATTGGATTGCATATGAAATCGAAGGAGGAAXXXXXXXXXXTTACATGATTGATGGGATGGAGCTCTTATACAAATGCCTAAGGGGCAAACTTTCTAATTTTGATTGTTTTTATCTCATTT

>Marker115336

AATTTATTATTTCATTGATGACAATTTTTTGGTTCATTGTCTAATGATAAATGCTAAAATTGATTGATAAGGCTATGGTAXXXXXXXXXXAAAAAATATATAAATTTAGAAGGGAAAAGGCATGAGTATATCTCTATTCCATTTATTTAGACCAAACGAATTAAACTAAA

AATTTATTATTTCATTGATGACAATTTTTTGGTTCATTGTCTAATGATAAATGCTAAAATTGATTGATAAGGCTATGGTAXXXXXXXXXXAAAAAATATATAAATTTAGAAGGGAAAAGGCATGAGTATATCTCTATTCCATTTATTTAGACCAAACGAAATAAACTAAA

>Marker115604

CTAATAATTGGAGTGAAATATTAGAAGCCCGTTGGTCATATATTAACAAAAACTAAGATTTGTTTTAATATAATTTCTTTXXXXXXXXXXAACCTAGAATGATGTTTATTTTTCTAATATAGCATATATAAACATGTCTTTTCCTATCATATTTGATCTTTTTCTTTTGT

CCAATAATTGGAGTGAAATATTAGAAGCCCGTTGGTCATATATTAACAAAAACTAAGATTTGTTTTAATATAATTTCTTTXXXXXXXXXXAACCTAGAATGATGTTTATTTTTCTAATATAGCATATATAAACATGTCTTTTCCTATCATATTTGATCTTTTTCTTTTGT

>Marker115716

TAAGCCATTCTACACTACATACGTTTCAAAACTATTCAAGACACTTTCTGTGCCAAATTGATTTTACTTTTATACCTCAAXXXXXXXXXXAACAATTACGGACGTGAACGATTATCTAAACCGATCGAAATAAAAAACGGGTAACACAAACTTTCATTTGGTAAAACGGT

CAAGCCATTCTACACTACATACGTTTCAAAACTATTCAAGACACTTTCTGTGCCAAATTGATTCTACTTTTATACCTCAAXXXXXXXXXXAACAATTACGGACGTGAACGATTATCTAAACCGATGGAAATAAAAAACGGGTAACACAAACTTTCATTTGGTAAAACGGT

>Marker115826

TATACCAATTTTTTTATAAGTTTAATTAATACCTTTAGTGTCACACTCACTCCCAAGTCATCTATTCTTAACCCAAGTGAXXXXXXXXXXTAAGGATATTGATTGATTTTGTTAATTGTAGTGCTAGTTAAGCCCAACTTAATGTCATTTTTACAGATTATGTTAGAGTT

TATACAAATTTTTTTATAAGTTTAATTAATACCTTTAGTGTCACACTCACTCCCAAGTCATCTATTCTTAACTCAAGTGAXXXXXXXXXXTAAGGATATTGATTGATTTTGTTAATTGTAGTGCTAGTTAAGCCCAACTTAATGTCATTTTTACAGATTATGTTAGAGTT

>Marker115880

TAATATTGATCAAACTCCCTCAGCATCTCTCAAAGATCTTGTAAAGTTTCTCCAATGATGCAACCTGTGAAAGTCAATGAXXXXXXXXXXCAACAAATTCAATGAAAAGAAAAGTCCTAATTTATCGAACTTCTGCCGAAGGTTTTCTCATATCTCCAACCTCAAGAAAA

TAATATTGATCAAACTCCCTCAGCATCTCTCAAAGATCTTGTAAAGTTTCTCCAATGATGCAACCTGTGAAAGTCAATGAXXXXXXXXXXCAACAAATTCAATGAAAAGAAAAGTCCTAATTTATCGAACTTCTGCTGAAGGTTTTCTCATATCTCCAACCTCAAGAAAA

>Marker115993

ACCGATTATTGTCTTTGACAATCGTCGAACAACTCTGACCACCACCACCTCCTTTTTTTGTAATTTAATATTAATAAAATXXXXXXXXXXTGAAAAAGCAGGACACTCGAAAAAAAAGTCTAATTGTATTAGTGACTAACAAGATAGTGATTCAAATTTCCCTCTTTATT

ACCGATTATTGTCTCTGACAATCGTCGAACAACTCCGACCACCACCACCTCCTTTTTTTGTAATTTAATATTAATAAAATXXXXXXXXXXTGAAAAAGCAGGACACTCGAAAAAAAAGTCTAATTGTATTAGTGACTAACAAGATAGTGATTCAAATTTCCATCTTTATT

>Marker116258

TACTTCTTGACTTTGATTTTAATTTTTTTCTGTGAAGAATGCTCCTCCTTCCTTGTTCTCAATTTTTATCTACATCATATXXXXXXXXXXGATGATTTTAGCAAGGTTATGCTGCTAGATTTTTAACTCCATGTTACATATGTCATCAGAGCAAAATAAATTATTTCTTT

TACTTCTTGACTTTGATTTTAATTTTTTTCTGTGAAGAATGCTCCTCCTTCCTTGTTCTCAATTTTTATCTACATCATATXXXXXXXXXXAATGATTTTAGCAAGGTTATGCTGCTAGATTTTTAACTCCATGTTACATATGTCATCAGAGCAAAATAAATTATTTCTTT

>Marker116263

ATATAATGAATCAATTCAGTGTGATGTGAGTGGAGTGAGTCATGTGTGCTATTTTGTGGAGCTTTGGAGAGGGAGAAACAXXXXXXXXXXTTTTTTTTCTTATCGAATGTCAACTTTCTTCAGCAAACAAAAATAAAAAGAAAAAAAGACAATCCAGTAGAAATGCCAAA

ATATAATGAATCAATTCAGTGTGATGTGAGTGGAGTGAGTCATGTGTGCTATTTTGTGGAGCTTTGGAGAGGGAGAAACAXXXXXXXXXXTTTTTTTTCTTATCGAATGTCAACTTTCTTCAGCAAACAAAAAGAAAAAGAAAAAAAGACAATCCAGTAGAAATGCCAAA

>Marker116686

CTATGCCTACGAAAGTGTGGCGAGAAGTAGGTGTCCTGTATTGACCTAACTAGACGATCTAGAAAGTATGTATCTGATGTXXXXXXXXXXGGCTAAAAATTAAAGGAAGAAGAGAGTCTTGGTTGATGAAAGATAACATTTCTGTTGATAGGGACAAGAGGAATTTCGGA

CTATGCCTACGAAAGTGTGGCGAGAAGTAGGTGTCCTGTATTGACCTAACTAGATGATCTAGAAAGTATGTATCTGATGTXXXXXXXXXXGGCTAAAAATTAAAGGAAGAAGAGAGTCTTGGTTGATGAAAGATAACAATTCTGTTGATAGGGACAAGAGGAATTTCGGA

>Marker116820

CAGAAATTTCTTGTATATAGAATCTCTAATTCATCTTGTTTGGCATTATTGATAAGAATTCCCTTTGCAGAACGTGAAATXXXXXXXXXXTGTATATGGGACTGAGCTTTGCCTTCCTGTAGTGGTGTGGGTCACAGTGTCAGGTGAGGTAAGAATCCAAGGGCAGGAGG

CAGAAATTTCTTGTATATAGAATCTCTAATTCATCTTGTTTGGCATTATTGATAAGAATTCCCTTTGCAGAACGTGAAATXXXXXXXXXXTGTATATGGGACTGAGCTTTGCCTTCCTGTTGTGGTGTGGGTCACAGTGTCAGGTGAGGTAAGAATCCAAGGGCAGGAGG

>Marker117047

AAGAAGAAGCCAAACCTGCATTTAAGGTGAGTGGTCTACTACTTGTATATAATAGCCATTCCCGACTGCCCATGCGCACAXXXXXXXXXXATTAGTCGTTTTTGGTAAACTCTTGATATAACAGTGGTTATTGTTCAGTGCTTATTAACATAGTTGCTCTTTAACTGCTA

AAGAAGAAGCCAAACCTGCATTTAAGGTGAGTGGTCTTCTACTTGTATATAATAGCCATTCCCGACTGCCCATGCGCACAXXXXXXXXXXATTAGTCGTTTTTGGTAAACTCTTGATATAACAGTGGTTATTGTTCAGTGCTTATTAACATAGTTGCTCTTTAACTGCTA

>Marker117134

CTTTTATGTAGACCAAATAAAGAAAGGCAAGACCTAAACTACATATATATTGAATGTGCTCATCCAAAATAATCACAACAXXXXXXXXXXAGTGTTTTATTAATTTATTTATTGGTCAGCTACTTGTTTAACTGTATATCCAAGGATCTCTCTTCATTTGATCGTCTTTT

CTTTTATGTAGACCAAATAAAGAAAGGCAATACCTAAACTACATATATATTGAATGTGCTCATCCAAAATAATCACAACAXXXXXXXXXXAGTGTTTTATTAATTTATTTATTGGTCAGCTACTTGTTTAACTGTATATCCAAGGATCTCTCTTCATTTGATCGTCTTTT

>Marker117194

TAAGTTAGATTTAAGAGAGATAAAATGGTGTGTATTAGAAAAAGTTGAAAGGAAAATGGAAGGTTCAAAGGTTGAGTGGGXXXXXXXXXXATATTGGACCTAAGCCCAACTGTTTGGACCACTTTAATGGGCTGACATTGCTCCCAAATTCAACAATTTACGGGCTATTC

CAAGTTAGATTTAAGAGAGATAAAATGGTGTGTATTAGAAAAAGTTGAAAGGAAAATGGAAGGTTGAAAGGTTGAGTGGGXXXXXXXXXXATATTGGACCTAAGCCCAATTGTTTGGACCACTTTAATGGGCTGACATTGCTCCCAAATTCAACAATTTACGGGCTATTC

>Marker117269

AATAAATGTAATATATTCTTTTTCTTTTTTATAAAGGCGATAATATTATTAATTAATATGAGGTAGATACAAATATAATTXXXXXXXXXXGAAAATGATATATATAAAAATATTTGGAACTTTGCAATAATAGGTTTTTTCGTATTAATGTGAGCATAACTCTCAAATGT

AATAAATGTAATATATTCTTTTTCTTTTTTATAAAGGCGATAATATTATTAATTAATATGAGGTAGATACAAATATAATTXXXXXXXXXXGAAAATGATATATATATAAATATTTGGAACTTTGCAATAATAGGTTTTTTCGTATTAATGTGAGCATAACTCTCAAATGT

>Marker117365

ATTTAATTTACATACTTTCGAATTTATGTATAAATTTATAGATTTTAAGAGGCATAGTTAAAGTAGTTTTTTTAATTTTCXXXXXXXXXXCAAAATTGGTAAAGCATGATCGAGTTAAAATTTTAATGAAAAATGGCTCTCTCAATGTTAATGAAATATGACCCAACAGA

ATTTAATTTACATACTTTCGAATTTATGTCTAAATTTATAGATTTTAAGAGGCATAGTTAAAGTAGTTTTTTAATTTTCAXXXXXXXXXXCAAAATTGGTAAAGCACGATCGAGTTAAAATTTTAATGAAAAATGGCTCTCTCAATGTTAATGAAATATGACCCAACAGA

>Marker117418

AAGGAGGATGCTAGAAAAAGTGGTGCAATTACCAATCAACAGGTTTGTATATTTTAAAATTATTTGCTTCTCTCACATTTXXXXXXXXXXTTTTCTTCCTATTAATTGGGTCTAATGGTTTTTCTTTTGTTTATGTCTCTTTCTTTTATTAAATAGGATGGTGAGTTCGA

AAGGAGGATGCTAGAAAAAGTGGTGCAATTACCAATCAACAGGTTTGTATATTTTAAAATTATTTGCTTCTCCCACATTTXXXXXXXXXXTTTTCTTCCTATTAATTGGGTCTAATGGTTTTTCTTTTGTTTATGTCTCTTTCTTTTATTAAATAGGATGGTGAGTTCGA

>Marker117653

TCTTCATGCTTCTGATTTACTTCCTGTGCCTGACTGTCAAGAAGAGCAAGAGTTTTCATGGTGCTCTCTAGCTCCTCGTGXXXXXXXXXXATTACTTAGTGTGCAGCTAAAGATTGCATGCCTTATTAATATCAAAGAGACACATTTCATTAAAAATTTCGTGCCAATCA

TCTTCATGCTTCTGATTTACTTCCTGTGCCTGACTGTCAAGAAGAGCAAGAGTTTTCATGGTCCTCTCTAGCTCCTCGTGXXXXXXXXXXATTACTTAGTGTGCAGCTAAAGATTGCATCCCTTATTAATATCAAAGAGACACCTTTCATTAAAAATTTCGTGCCAATCA

>Marker117735

ATTCGATCATATAGAGATTTGCCTTTTTTTTCATATACCAATTGAAATAAATTATTAATCTTATTAATCAGTAGGTTACAXXXXXXXXXXGTTTGTATATTATATAGGGAAAAAAAGAGGAAGAAGAAGAGGGAGAGAAAGAGGATTTTAGTAGATTAAGGGGGGAGTGG

ATTCGATCATATAGAGATTTGCCTTTTTTTTTATATACCAATTGAAATAAATTTTTAATCTTATTAATCAGTAGGTTACAXXXXXXXXXXGTTTGTATATTATATAGGGAAAAAAAGAGGAAGAAGAAGAGGGAGAGAAAGGGGATTTTAGGAGATTAAGGGGGGAGTGG

>Marker117919

CTTTGTTTTAATTTTATAAAACATGTATTTTGACTAAATGAGTAAATATTCACTTCTTAAAAACATGTATATATAAAAATXXXXXXXXXXAAACTACCTCTCTTCTACTTCTATATTTGTGTTCATAATTAATACATATAATATAATTTTAACCTTTTGCATGCATGGAT

CTTTGTTTTAATTTTATAAAACGTGTATTTTGACTAAATGAGTAAATATTCACTTCTTAAAAACATGTATATATAAAAATXXXXXXXXXXAAACTACCTCTCTTCTACTTCTATATTTGTGTTCATAATTAATACATATAATATAATTTTAACCTTTTGCATGCATGGAT

>Marker118181

TTAAAATTCCTTAGGATGAATATGAATGAGGCTGAAGATTGAGAGTAGTGTATATAGCCACATAGGTATTAACTAGAACCXXXXXXXXXXAAAGAAAATGCTATATTCTGTCATGTAAATGGTGGTAAAAAGCTGCCTTAAGCACATTACTATTGTTTTTTGTTCCAAAG

TTAAAATTCCTTAGGATGAATATGAATGAGGCTGAAGATTGAGAGTAGTGTATATAGCCACATAGGTATTAACTAGAACCXXXXXXXXXXAAAGAAAATGCTATATTCTGTCATGTAAATGGTGGTAAAAAGCTCCCTTAAGCACATTACTATTGTTTTTTGTTCCAAAG

>Marker118570

TGTTGATGGTTATTGGAGTAATCTAAGAAAACCCCAGAAGAGATTGAAGCCGAAAAAAGAAGAAATAATGATTCGATTGGXXXXXXXXXXGATGAAAATATATTATTTCCTCCAAAAGTTTCTCTCACTTTTCTCCTCTTCTCAGTGTGGGCTTAATTCTACTCCCACGA

TGTTGATGGTTATTGGAGTAATCTAACAAAACCCCAGAAGAGATTGAAGCCGAAAAAAGAAGAAATAATGATTCGATTGGXXXXXXXXXXGATGAAAATATATTATTTCCTCCAAAAGTTTCTCTCACTTTTCTCCTCTTCTCAGTGTGGGCTTAATTCTACTCCCACGA

>Marker118589

TCACACGAACAGATATCATTCTCTACTCAACAATAATTGCTAGCCCTAACTTCTAATACCTTTACTTGAATAACTCGGGGXXXXXXXXXXTCGGCTAATATCACAATTTCTCATATTTTCGACCTCGCAATTTGGTTCACACAAACACGCATGACGCAAGTGGAGAGTAA

TCACACGAACAGATATCATTCTCTACTCAACAATAATTGCTAGCCCTAACTTCTAATACCTTTACTTGAATAACTCGGGGXXXXXXXXXXTCGGCTAATATCACAATTTCTCATATTTTCGACCTCGCAATTTGGTTCATACAAACACGCATGACGCAAGTGGAGAGTAA

>Marker118916

ACCACCCAACAATATAAACCACCTGAGCCAAGGAGATTCCCCACGCAGACACGTCGTAAGCCGTGGCTGCTCCGTCCGTCXXXXXXXXXXGTAGAGTGGTAGAAGAACGATGCATGCACAGAAAAGGATTATCCACGAGCGTTGCATGTAGATTCCTAGCATGTTCATTT

ACCACCCAACAATATAAACCACCTGAGCCAAGGAGATTCCCCACGCAGACACGTCGTAAGCCGCGGCTGCTCCGTCCGTCXXXXXXXXXXGTAGAGTGGTAGAAGAACGATGCATGCACAGAAAAGGATTATCCACGAGCGTTGCATGTAGATTCCTAGCATGTTCATTT

>Marker119052

TTCTTTGGGAGCCTTGTATTTAGGAGAGCAAATCCATACCCATGTTATCAAGACTGGGTTTCAGTTGAATGTCTATGTTTXXXXXXXXXXAATATCGAGGAATTCAATTCGACAATATTGGATTTGCCAGTGCCATTAGTGCATGCGCAGGTATCCGGGCACTCCGTCAA

TTCCTTGGGAGCCTTGTATTTAGGAGAGCAAATCCATACCCATGTTATCAAGACTGGGTTTCAGTTGAATGTCTATGTTTXXXXXXXXXXAATATCGAGGAATTCAATTCGACAATATTGGATTTGCCAGTGCCATTAGTGCATGCGCAGGTATCCGGGCACTCCGTCAA

>Marker119145

TCTTTTTAAGAGATCGGTTTGTCCTACATATTTTCTTCAAGTTCTTTTTTTTTAAAAAGAAAAGTTGTTAATAGTTAAAGXXXXXXXXXXAAGAGCTTAAAATAATAGAAGCAATTATTGCTTTTGGTATACAAAAATAAATTCAATCATGATGATGCTTACAATTATAA

TCTTTTTAAGAGATCGGTTTGTCCTACATATTTTCTTCAAGTTCTTTTTTTTAAAAAAGAAAAGTTGTTAATAGTTAAAGXXXXXXXXXXAAGAGCTTAAAATGATAGAAGCAATTATTGCTTTTGGTATACAAAAATAAATTCAATCATGATGATGCTTACAATTATAA

>Marker119244

ATATTGATAAAGCATCGTGGACCCATTGGACTGCTCCTTAAACACGACGCAAACGAAATGATGCCTTGTCTTTCACCTCTXXXXXXXXXXAACTGAGCAGGGGTTGCATTAACTTGGAGAAATCGTCCGAGGAGGTTGCTACAATGTGATGTATAAAAGAGAAGAAAAAA

ATATTGATAAAGCATCGTGGACCCATTGGACTGCTCCTTAAACACGACGCAAACGAAATGATGCCTTGTCTTTCACCTCTXXXXXXXXXXAACTGAGCAGGGGTTGCATTAACTTGGAGAAATCATCCGAGGAGGTTGCTACAATGTGATGTATAAAAGAGAAGAAAAAA

>Marker120718

TAGTGTTTAAATAGTTACCAAAACTGAATATAACATTTTAAAATCTGAAAAACCAAATCGAAATTAGACTTACGAACTAAXXXXXXXXXXTTTAACAAAAATCAATTTATTTAGAAGACCTAATATTGAATCATATATACAAACTAATTATATTAAGTATGGAATAATGA

TAGTGTTTAAATAGTTACCAAAACTGAATATAACATTTTAAAATCTGAAAAACCAAATCGAAATTAGACTTACGAACTAAXXXXXXXXXXTTTAACAAAAATCAATTTATTTAGAAGACCTAAAATTGAATTATATATACAAACTAATTATATTAAGTATGGAATAATGA

>Marker120789

TTCTGTTCCCTTTCTTGTCAAAGTCTGTAATGCCTATATATATTTTGTATATCTTCCATTGGCTAATACATAGTGAAATAXXXXXXXXXXGATGGTTACTTGGTTCGATGACCCTAGCCGCTTGTGGCATCAGAGGTCGTTAATCTGATAATGTCAAGAGAGTTATGGTT

TTCTGTTCCCTTTCTTGTCAAAGTCTGTAATGCCTATATATATTTTGTATATCTTCCATTGGCTAATACATAGTGAAATAXXXXXXXXXXGATGTTTACTTGGTTCGATGACCCTAGCCGCTTGTGGCATCAGAGGTCGTTAATCTGATAATGTCAAGAGAGTTATGGTT

>Marker121308

ACGACTTTAACAATTTTAGAAGATGTGCCAGTAAATATTTCTCTTAGCCAAAAGTGGATTATTCCCTTAATCAAAAGCCAXXXXXXXXXXAGAAGCACATTCAGGTATATGTGGTGCTCACCAGTCTGGTCCAAAACTTCAGCATCAGTTGAAAAGAATGGGTTACTATT

ACGGCTTTAACAATTTTAGAAGATGTGCCAGTAAATATTTCTCTTAGCCAAAAGTGGATTATTCCCTTGATCAAAAGCCAXXXXXXXXXXAGAAGCACATTCAGGTATATGTGGTGCTCACCAGTCTGGTCCAAAACTTCAGCATCAGTTGAAAAGAATGGGTTACTATT

>Marker121700

GAGACAAATATGGCAGTGTGTTCAAAGAGTATTTTTCATGGAGAGAACAATGAAGAACTCTAAGATTATCCTCTACAAGAXXXXXXXXXXATCTTCTTGAAATAATTAGAACATTTTCTTGAAATAATTACACTTGGGCAGTCAAAAGACAATCTACCAAGCAGATTCTA

GAGAAAAATATGGCAGTGTGTTCAAAGAGTATTTTTCATGGAGAGAACAATGAAGAACTCTAAGATTATCCTCTACAAGAXXXXXXXXXXATCTTCTTGAAATAATTAGAACATTTTCTTGAAATAATTACACTTGGGCAGTCAAAAGACAATCTACCAAGCAGATTCTA

>Marker122077

TGAAAATTCCTTTGTGTAGGAAATCAACCCAACCACTATGATTTGAAAGAAATTCATGAAGAACTGAAAGATTAATCAAAXXXXXXXXXXAATACTAAATAGGAGGATCATCACTTCATTTGGAGAAACTACCTGAAGAAGAGTCCCAATGCCAATTGCAGATTTCATTC

TGTAAATTCCTTTGTGTAGGAAATCAACCCAACCACTATGATTTGAAAGAAATTCATGAAGAACTGAAAGATTAATCAAAXXXXXXXXXXAATACTAAATAGGAGGATCATCACTTCATTTGGAGAAACTACCTGAAGAAGAGTCCCAATGCCAATTGCAGATTTCATTC

>Marker122326

ATTTACGAAAAAAGAAGAGTATCAGAAATAGAAACTTAATTACAGATTTAAGTGTTTATGTAACGGGACTTACCTGAAGTXXXXXXXXXXATTGTTCATCATCATCGTCTATAAAGTCTTAAACAACATGGTGCACATCCGATCTTCCCACCACTGTAGGATCAACCTCA

ATTTACGAAAAAAGAAGAGTATCAGAAATAGAAACTTAATTACAGATTTAAGTGTTTATGTAACGGGACTTACCTGAAGTXXXXXXXXXXATTGTTCATCATCATCGTCTATAAAGTCTTAAACAACATGGCGCACATCCGATCTTCCCACCACTGTAGGATCAACCTCA

>Marker122451

CTGTAAGAAATATAAAACAAAATAGTAATTGGGACTGGGGAGTCATCTGCCGAAAGAAAGGGGCGCCCTAAGTGTAATAAXXXXXXXXXXGAATAAAAGGAAGCCGTATCATGACCATCATATCATAATTAACAACATCATATATCTTTACAGAATGATCATTTGATATT

CTGTAAGAAATATAAAACAAAATAGTAATTGGGACTGGGGAGTCATCTGCCCAAAGAAAGGGGCGCCCTAAGTGTAATAAXXXXXXXXXXGAATAAAAGGAAGCCGTATCATGACCATCATATCATAATTAACAACATCATATATCTTTACAGAATGATCATTTGATATT

>Marker122555

TTTCCAAGAACACCCTTTCCCTATTTTGGTTGATTTATTAAGCAATAATAATACATTTAAATCTCTCTCTTTGATTACAGXXXXXXXXXXAAACTCCGCCATCGCCATCTGGTTTCTCTCATCGGTTTCTGCGACGAACAATCCGAGATGATTCTGGTTTACGAATACAT

TTTCCAAGAACACCCTTTCCCTATTTTGGTTGATTTATTAAGCAATAATAATACATTTAAATCTCTCTCTTTGATTACAGXXXXXXXXXXAAACTCCGCCATCGCCATCTGGTTTCTCTCATCGGTTTCTGCGACGAACAGTCCGAGATGATTCTGGTTTACGAATACAT

>Marker122784

TAGACTAGAAATGTTGCTAATTTGGGTGGCTCTTCTTAATTTTTTACATTGTCGGCGTGACTGACGTTTGATTTTCTTCAXXXXXXXXXXTAAAATAGAGAGGACGCCAGCGTCAACACCTGGAGGACCTCGATCTAAGGAAGAGAAGATCGTTGTTACTGTGCGGTTAA

TAGACTAGAAATGTTGCTAATTTGGGTGGCTCTTCTTAATTTTTTACATTGTCGGCGTGACTGACGTTTGATTTTCTTCAXXXXXXXXXXTAAAATAGAGAGGACGCCAGCGTCAACACCTGGAGGACCTCGATCTAAGGAAGAAAAGATCGTTGTTACTGTGCGGTTAA

>Marker122958

TTTATTGAATTGACCACTGAATTTATTGTTGTTTTCGTTTTTCTTTTTCATTCTTAAGATTAAATGGTTTGTATTTTATCXXXXXXXXXXTGTAAAATCAATTGTTAAACGGATTTCGTAAGTGTAGGTGACTTTGTAAATGTAAGTGATTTCAAATATGATAAAAGTCT

TTTATTGAATTGACCACTGAATTTATTGTTGTTTTCGTTTTTCTTTTTCATTCTTAAGATTAAATGGTTTGTATTTTATCXXXXXXXXXXTGTAAAATCAATTGTTAAATGGATTTCGTAAGTGTAAGTGACTTTGTAAATGTAAGTGATTTCAAATATGATAAAAGTCT

>Marker123136

GCCCATCTTAAGCAGTTGTGATAATTTATTTCATTTAAGCATATCTTGTCATTTAGATGACGATCAACCCAAAATACTAGXXXXXXXXXXGTATTGGTAAGCTACTTTGGAATTAAACTGGTCAGCTGCTTCTTTAAGAATAGGATCTGAGGTGTGTCTGATTATCACAA

GCCCATCTTAAGCAGTTGTGATAATTTATTTCATTTAAGCATATCTTGTCATTTAGATGACGATCAACCCAAAATACTAGXXXXXXXXXXGTATTGGTAAGCTACTTTGGAACTAAACTGGTCAGCTGCTTCTTTAAGAATAGGATCTGAGGTGTGTCTGATTATCACAA

>Marker123251

AACCTTTTGCTGATGCTGATATTATGAAAGATTTTTTTGTTCCTCTTCTGGGTTTGGACTACTTTGGCAAGTTAGCTTTGXXXXXXXXXXTCCTTTTATTGACCGAGGTCGTCTTTGGGCAGTTTGTTTGGTTGTTTGTCTTTATTTTTTGCTCTTTGTATAATTCTCTT

AACCTTTTGCTGATGCTGATATTATGAAAGATTTTTTTGTTCCTCTTCTGGGTTTGGACTACTTTGGCAAGTTAGCTTTGXXXXXXXXXXTCCTTGTATTGACCGAGGTCGTCTTTGGGCAGTTTGTTTGGTTGTTTGTCTTTATTTTTTGCTCTTTGTATAATTCTCTT

>Marker123519

ACAGGCTGAGATTGGGAAAAACTACAAGTAGAAAAAGAGGCAGAACAAAAACAGCTGATGAGAGATCCAAAACAGAGTAGXXXXXXXXXXGCTATGGGGTGTCAACCTAGTTGAGATGCCCAAGTGCACTTCTTGATCTATGATTTACATGCTCATTGTATAATTCTCTT

ACAGGCTGAGATTGGGAAAAACTACAAGTAGAAAAAGAGGCAGAACAAAAACAGCTGCTGAGAGATCCAAAACAGAGTAGXXXXXXXXXXGCTATGGGGTGTCAACCTAGTTGAGATGCCCAAGTGCACTTCTTGATCTATGATTTACATGCTCATTGTATAATTCTCTT

>Marker123577

ACGACTTTAGACCCTCGACAAAATAAAAAACTTTATCTTTCTCAGACATATCATGTATGTCTAACATCAGCACCGCAAAGXXXXXXXXXXGATCTCCACCACAATTTGGCATCTTTAGAGAGATGCATAGTCGTCAATGCCAATTGGGCTTCTTTTTTTATTGTATTTGT

ACGACTTTAGACCCTCGACAAAATAAAAAACTTTATCTTTCTCAGACATATCATGTATGTCTAACATCAGCACCGCAAACXXXXXXXXXXGATCTCCACCACAATTTGGCATCTTTAGAGAGATGCATAGTCGTCAATGCCAATTGGGCTTCTTTTTTTATTGTATTTGT

>Marker123671

CTTGTAAGTTATATATTATTCCATTTTGTAAACTTCTTAGTCTTGCTTTGAAAAAAAACGACTTAAAACAACTTTATCCTXXXXXXXXXXCATTAAGGGGGAAGATTTTCATTTCTTACTTGATATTGTTATATAGTTAATTAATTAAAGGCAAGTTAATTTGTTTCGGT

CTTGTAAGTTATATATTATTCCACTTTGTAAACTTCTTAGTCTTGCTTTGAAAAAAAACGACTTAAAACAACTTTATGCTXXXXXXXXXXCATTAAGGGGGAAGATTTTCATTTCTTACTTGATATTGTTATATAGTTAATTAATTAAAGGCAAGTTAATTTGTTTCGGT

>Marker123723

TTACACAATGTATGGGGGGTGACCTTCCGTCTGCCACAAGCCAAAGAAGAAAGCATTTAGTGTTACATAAAACCTATAAGXXXXXXXXXXAAAGACATCAATTCAAAATCATAACCATAAAGATCAGCTCTAAAAAATAGCACACGTAGTAGAAAGTTTAGATAGATGGG

TTACACAATGTATGAGGGGTGACCTTCCGTCTGCCACAAGCCAAAGAAGAAAGCATTTAGTGTTACATAAAACCTATAAGXXXXXXXXXXAAAGACATCAATTCAAAACCATAACCATAAAGATCAGCTCTAAAAAATAGCACACGTAGTAGAAAGTTTAGATAGATTGG

>Marker124372

TTAACATGCATGTTTTTTACATCCACCTTGTTTCCACTAACAACAATTGGAATATTCTCACACCCTGAGAGTTATTAAGTXXXXXXXXXXAAAAACAAAAACAATCTAACAAATCAAGTGTGCTAGAGAATATTGAAATTTCAACAACAGTGTTCAGAATAAAAAGTGAA

TTAACATGCATGTTTTTTACATCCACCTTGTTTCCACTAACAACAATTGGAATATTCTCACACCCTGAGAGTTATTAAGTXXXXXXXXXXAAAAACAAAAACAATCTAACAAATCAAGTGTGCTAGAGAATATTGAAATTTCAACAATAGTGTTCAGAATAAAAAGTGAA

>Marker125003

TTTAACATAATGATATGTGATGAAGACCCACAAACCCATTATTAGAATTATAATATCGTTCGATTTTTGTTTTTTTTCCTXXXXXXXXXXTCCCACATATGTATATATGACCTAATATGTGTATACATAGAGAGGAAGATGTGTGTCTCATCTCGACTCGACTCGAACTA

CTTAACATAATGATATGTGATGAAGACCCACAAACCCATTATTAGAATTATAATATCGTTCGATTTTTGTTTTTTTTCCTXXXXXXXXXXTCCCACATATGTATATATGACCTAATATGTGTATACATAGAGAGGAAGATGTGTGTCTCATCTCGACTCGACTCGAACTA

>Marker125199

TAGCAATACCCCCATGGAGAAGAGACACCTTGTCCTCAAGGTGGAATTCTGGGTATTAATTCGCAAGCATCGCATAGGACXXXXXXXXXXGGAAAACCAGATGTATTTTGGTAGTTTTTGGTAGGTCCAGCATGTAAGCTACTTCTCCGACTCTACCCAATATCATGTAT

TAGCAATACCCCCATGGAGAAGAGACACCTTGTCCTCAAGGTGGAATTCTGGGTATTAATTCGCAAGCATCGCATAGGACXXXXXXXXXXGGAAAACCGGATGTATTTTGGTAGTTTTTGGTAGGTCCAGCATGTAAGCTACTTCTCCGACTCTACCCAATATCATGTAT

>Marker125228

TTAAATTGTTTGTTTGGCATGCCGTAGTGACACAAATGGAGGCTTATACCCATTATTAATGTATTGTATTTTGACATTTAXXXXXXXXXXTTTTGTTGTCATGCTTAACGATTCTCAACTTTTCAATTTCAGATATGATTTATCTTACTATGTGGGAAATTGATAAAAAC

TTAAATTGTTTGTTTGGCATGCCGTAGTGACACAAATGGAGGCTTATACCCATTATTAATGTATTGTATTTTGACATTTAXXXXXXXXXXTTTTGTTGTCATGCTTAACGATTCTCAACTTTTCAATTTCAGATATGATTTATTTTACTATGTGGGAAATTGATAAAAAC

>Marker125256

ACATCTCCACTATAGATAGTTCTGTGAACTTGTATTTATAATCCAGGCTTAAAGCTTTCTATCGGCTTATGTAGTCGAATXXXXXXXXXXTTTTCGCATGTTTCAACAAAGTGAGCAATATGTTTCTTTTGATTGCCCTTTTCGTCGAATTGTTGGAAATTTGGAAGTTG

ACATCTCCACTATAGATAGTTCTGTGAACTTGTATTTATAATCCAGGCTTAGAGCTTTCTATCAGCTTATGTAGTCGAATXXXXXXXXXXTTTTCGCATGTTTCAACAAAGTGAGCAATATGTTTCTTTTGATTGCCCTTTTCGTCGAATTGTTGGAAATTTGGAAGTTG

>Marker125263

ATATCCCACTTTTAAAAATGTGAAACTTTGTTACAACTCTTCAATGAAAAATGGTTTGTATCTCTTTTGTAAGACTTCTTXXXXXXXXXXTATAAATATAATAAAATATCACAACTTATTTACGATAGGCTATGGATGATCACCGTCTCCATGTTTGGGAGAAAAACTAA

ATATCCCACTTTTAAAAATGTGAAACTTTGTTACAACTCTTCAATGAAAAATGGTTTGTATCTCTTTTGTAAGACTTCTTXXXXXXXXXXTATAAATATAATAAAATATCACAACTTATTTACGATAGACTATGGATGATCACCGTCTCCATGTTTGGGAGAAAAACTAA

>Marker125388

TTGCTTGCGGATCTGGGGAAGGGTATTATTCCCTTCGATACGGAGACCGATGTGGAAGGATCCATGGGGCTCTTCTTTATXXXXXXXXXXCCAACTTTGTGGTTGACTCTTTGGATGTCTAGTAGCGAACCCATTTCATGGTCTTGCAAGTTGCAGCAGTCCTAAATCAT

TTGCTTGCGGATCTGGGGAAGGGTATTATTCCCTTCGATACGGAGACCGATGTGGAAGGATCCATGGGGCTCTTCTTTATXXXXXXXXXXCCAACTTTGTGGTTGACTCTTTGGATGTCTAGTAGCGAACCCATTTCATGGTCCTGCAAGTTGCAGCAGTCCTAAATCAT

>Marker125872

TTATAGATAATGCTCCATGGTGAGTTATGACCTCCTAAGATCAATATGCTGAACATGAAGTTACAATCCTAGCTTTGGATXXXXXXXXXXAATGGTCGTCTGTAACTGACATGAATTCATCCATTTCCTGATTTCTTCATCTTCTTCCTTGGAAATTGCTTGGTTTTTTT

TTATAGATAATGCTCCATGGTGAGTTATGACCTCCTAAGATCAATATGCTGAACATGAAGTTACAATCCTAGCTTTGGATXXXXXXXXXXAATGGTCGTCTGTAACTGACATGAATTCATCCATTTCCTGATTTCTTCATCTTCTTCCTTGGCAATTGCTTGGTTTTTTT

>Marker125917

TCTACATGCTTTTGCAAATTGGTTTCTATAATTCAGGTAGATTTAGAAAACTATTTTATTTTGCCTAACCAACATATTGTXXXXXXXXXXTTTAAATGTCTGTTTTCGAATTGACATCAGATACTTATAATGAGGAATTACACACTGCATGGTATGGTGTGTCATCGTAG

TCTACATGCTTTTGCAAATTGGTTTCTATAATTCAGGTAGATTTAGAAAACTATTTTATTTTGCCTAACCAACATATTGTXXXXXXXXXXTTTAAATGTCTGTTTTCGAATTGACATCAGATACTTATAATGAGGAATTACACACTGCATGGTATGATGTGTCATCGTAG

>Marker125962

TCTTCTGTCAATGAAGGATGACGGTGAAGATGAGAATGAGAAGCTCAATGATACTGAGATTAAGGCTTTGTTGTTGGTATXXXXXXXXXXCAATTTCTCTATGATCGTTATTATTTCATACAGAACATGTTCACAGCTGGAACCGACACGTCAGCAAGCACGGTGGAATG

TCTTCTGTCAATGAAGGATGACGGTGAAGATGAGAATGAGAAGCTCAATGATACTGAGATTAAGGCTTTGTTGTTGGTATXXXXXXXXXXCAATTTCTGTATGATCGTTATTATTTCATACAGAACATGTTCACAGCTGGAACCGACACGTCAGCAAGCACGGTGGAATG

>Marker126015

AACTAATCTTGCTTTATATGTTGTCGTGATCTCATTGTTTTCATTTCTTTTTCTCACAAATACTCATTTATATCCCACAGXXXXXXXXXXGCAAAAATGTTGTCAACTAAAGTGAGTCTTTTCACGTGACATAAGTGTTGGATAGTTGATAGGAAGATCTGTAACTATAT

AACTAATCTTGCTTTATATGTTGTTGTGATCTCATTGTTTTCATTTCTTTTTCTCACAAATACTCATTTATATCCCACAGXXXXXXXXXXGCAAAAATGTTGTCAACTAAAGTGAGTCTTTTCACGTGACATAAGTGTTGGATAGTTGATAGGAAGATCTGTAACTATAT

>Marker126178

TAGAGAAGAGAGCAGGAAATTAATACTTCCTAGTAAATGAAGATGATAGATGGAAACTATGCTATTTTTAATTGTCAGAAXXXXXXXXXXTTCACTATCGATAGATTCATTACTTGATGAAGGAATATGAAGTTTCTCTTTCCAACTTACCAACCGTCATCTAGATGGAA

TAGTGAAGAGAGCAGGAAATTAATACTTCCTAGTAAATGAAGATGATAGATGGAAACTATGCTATTTTTAATTGTCAGAAXXXXXXXXXXTTCACTATCGATAGATTCATTACTTGATGAAGGAATATGAAGTTTCTCTTTCCAACTTACCAACCGTCATCTAGATGGAA

>Marker126191

CTTTTCTTCATTTCCAACAAATCTTAGTAATGTTTCGTCATACTCTATCAACAGGCAGATCCATAAAACAACAGAAAGAAXXXXXXXXXXTCAATGCATCAAAAAGTCATGAGAATAGTTGGTCACCTTCAAAGCTGCTTCAGTAGGCATTCCATTTGCAACAAAATGAT

CTTTTCTTCATTTCCAACACATCTTAGTAATGTTTCGTCATACTCTATCAACAGGCAGATCCATAAAACAACAGAAAGAAXXXXXXXXXXTCAATGCATCAAAAAGTCATGAGAATAGTTGGTCACCTTCAAAGCTGCTTCAGTAGGCATTCCATTTGCAACAAAATGAT

>Marker126674

AGAGTGGGAGATGCGGGTGGTAGCAGCTGCGGATGGGGGCGGTCGCAGATGGAAGCGAGTGAGTGTGTTTGGAATGGGTTXXXXXXXXXXTAGTGCATCGAAAATCATACAAAAATGCGTCGGAAGAGGTTTGCCCGATGTCAGAAGCTCAGTCAGGGAGGACATTGGGA

AGAGTGGGAGATGCGGGTGGTAGCAGCTGCGGATGGGGGCGGTCGCAGATGGAAGCGAGTGAGTGTGTTTGGAATGGGTTXXXXXXXXXXTAGTGCATCGAAAATCATACAAAAATGCATCGGAAGAGGTTTGCCCGATGTCAGAAGCTCAGTCGGGGAGGACATTGGGA

>Marker127251

TCTTTGCAACCTATGCTTTCTTATTGCAATTGACAGTCTTCAATGCTTGTTTTAACAATGTTTTCAATCAATTTAATCATXXXXXXXXXXTGAATATTCACCTGTTTTGAACATTTAGTCTCCTATAATATAAGGCTGAAAAAAGGTAGTTTGGATATGTTCTCCCTTTG

TCTTTGCAACCTATGCTTTCTTATTGCAATTGACAGTCTTCAATGCTTGTTTTAACAATGTTTTCAATCAATTTAATCATXXXXXXXXXXTGAATATTCACCTGTTTTGAACATTTAGTCTCCTATAATATAAGGCTGAAAAAAGGTAGTTTGGATATGTTCTCCCTTCG

>Marker127343

TCCTAATTTTCTCCATGAAAGCTCTGTTTTTCCCTTTATTTATATTATATGGCACTGCAGGCTGTTTTCATGATGTGTGCXXXXXXXXXXCATTCCAAATTCTTATTTATCATTGATAACTTTTTGAAATAAGAATTGTGAAATTCAATTAAAAGGGTTTAAGGTTTAGG

TCCTAATTTTCTCCATGAAAGCTCTGTTTTTCCCTTTATTTATATTATATGGCACTGCAGGCTGTTTTCATGATGTGTGCXXXXXXXXXXCATTCCAAATTCTTATTGATCATTGATAACTTTTTGAAATAAGAATTGTGAAATTCAATTAAAAGGGTTTAAGGTTTAGG

>Marker127354

AAATCCCCTACTTCCTATTGGAAAGAGAACGACCACGAGCTCCCAGACCAAACAAGGAAATCAACAATAATGTAATTTTAXXXXXXXXXXAGCTGCATTCATGTCAATTTTCCTTGATTACGTCTCATCTAATTCCTTCCAAGTTTTCCTCCATGATTGAAGCTTGATAG

ACATCCCCTACTTCCTATTGGAAAGAGAACGACCACGAGCTCCCAGACCAAACAAGGAAATCAACAATAATGTAATTTTAXXXXXXXXXXAGCTGCATTCATGTCAATTTTCCTTGATTACGTCTCATCTAATTCCTTCCAAGTTTTCCTCCATGATTGAAGCTTGATAG

>Marker127872

TTCTAAAATATTTTAATATTATTTTCCTGCTTGTTTATTTTTAAAAATAAAATAAAATTCACCTAGTTTTTAACTTTTTTXXXXXXXXXXGAACTTCAAAAGTTATTTAGACACCAAAAAGTCATAATAGATCTAGAAATTTAGATTCATTCACATATTTGATGTTTTAT

TTCTAAAATATTTTAATATTATTTTCCTGCTTGTTTATTTTTAAAAATAAAATAAAGTTCACCTAGTTTTTAACTTTTTTXXXXXXXXXXGAACTTCAAAAGTTATTTAGACACCAAAAAGTCATAATAGATCTAGAAATTTAGATTCATTCACATATTTGATGTTTTAT

>Marker128135

TACTTTAATTTAATTTTTTTTAAAGTCTTTCTTAATCTTACTTTCATGTTCTTAGAATTTATTTCATTCAGAGGTCATATXXXXXXXXXXTTCAACTTCCTTCCTTCCTTGTAGCCCCTGCTTCATAATGTAACTGCACACTAGGAAAATTCACATCTTTTACGTAACAA

TACTTTAATTTAATTTTTTTTAAAGTCTTTCTTAATCTTACTTTCATGTTCTTAGAATTTATTTCATTCAGAGGTCATATXXXXXXXXXXTTCAACTTCCTTCCTTCCTTGTAGCCCCTGCTTCATAATGTAACTGCACACTAGGGAAATTCACATCTTTTACGTAACAA

>Marker128200

AAACTATTTAAACTCTAGGACAAAAAACCACTAAAGGCGACATAAACTCATTACACTTGATTTTGCCATCAAGTGTAAATXXXXXXXXXXGTCTATCAATCAATCATTCTGCTCCCTCTATGCTTGCTTTGATTCGAAGTTGAACATTTCGATTGTTCAAAGGTTGCAAA

AAACTATTTAAACTCTAGGGCAAAAAACCACTAAAGGCGACATAAACTCATTACATTTGATTTTGCCATCAAGTGTAAATXXXXXXXXXXGTCTATCAATCAATCATTCTGCTCCCTCTATGCTTGCTTTGATTCGAAGTTGAACATTTCGATTGTTCAAAGGTTGCAAA

>Marker128306

TCTTTGCAAGCAAACCTTTTCCCTTGCAACTGACAATTTTCAATGCATATTGTAACGTTGATTTCAATGTATTTCTTTCTXXXXXXXXXXTTTGGAAGTGTTAAAAACCTCAGAACACAGTTAGCATGTGGCTTAAGCCATGTAGCCAAGTGGGGTGTCTTGCGACCATA

TCTTTGCAAGCAAACCTTTTCCCTTGCAACTGACAATTTTCAATGCATATTGTAACGTTGATTTCAATGTATTTCTTTCTXXXXXXXXXXTTTGGAAGTGTTAAAAACCTCAGAACACAATTAGCATGTGGCTTAAGCCATGCAGCCAAGTGGGGTGTCTTGCGACCATA

>Marker128384

TTATGTGATTAATCACTCAACCTCAGAAAATGTGTGAAATATAATCTTCTAATTACATGTAATGCATTAGGGCTATACAAXXXXXXXXXXTATGTGTAACTCAGAGTAAACAAAATGAATAATAATTTATGGAAAAACTACATTTCTAATCCTCAAAATTTGAAAAATTT

TTTTGTGATTAATCACTCAACCTCAGAAAATGTGTGAAATATAATCTTCTAATTACATGTAATGCATTAGGGCTATACAAXXXXXXXXXXTATGTGTAACTCAGAGTAAACAAAATGAATAATAATTTATGGAAAAACTACATTTCTAATCCTCAAAATTTGAAAAATTT

>Marker128656

ACTTGATTATTCTTACATTTTTAAAATATCAAATTTATATTATTTTTCAGTTTGGATTCCTATATGTAAAATCATATAATXXXXXXXXXXATATATTGAGTTAAGGGAACTATAGTATGGTTGGAGTAAGCTTAAGGGAAAAGTTTAAACATCTTCTTCCTATTCCCTAT

ACTTGATTATTCTTACCTTTTTAAAATATCAAATTTATATTATTTTTCAGTTTGGATTCCTATCTGTAAAATCATATAATXXXXXXXXXXATATATTGAGTTAAGGGAACTACAGTATGGTTGGAGTAAGCTTAAGGGAAAAGTTTAAACATCTTCTTCCTATTCCCTAT

>Marker128759

ATTGGCTAAGTAAAGTTAGGTTGAGTGTAGCTATAATTTTTTATAATAATAGATCGAGAAATCAGAAATTATTTTTTCTTXXXXXXXXXXCTTCAATTCTCTTCGCAACACTATCAAATTAAATCAATACACTCACACAACTTCTAAAAGTAATTAGAAAATATAGTAGA

ATTGGCTAAGTAAAGTTAGGTTGAGTGTAGCTATAATTTTTTATAATAATAGATCAAGAAATCAGAAATTATTTTTTCTTXXXXXXXXXXCTTCAATTCTCTTCGCAACACTATCAAATTAAATCAATACACTCACACAACTTCTAAAAGTAATTAGAAAATATAGTAGA

>Marker128773

ACTTAGAATTAGGTTTCCAGCAAAGAGATACAACAGAAGAAGGGGTAGCTCTACTTGTCTTAATCGCAGAGAAGTTCATTXXXXXXXXXXAGAGCCAATTATGCTTCAATTTTAATGGGTAATCTTCGATTTTGAAGATGGTAAGTAAAGGGAAATGGAAAATTCAGAAA

ACTTAGAATTAGGTTTCCAGCAAAGAGATACAACAGAAGAAGGGGTAGCTCTACTTGTCTTAATCGCAGAGAAGTTCATTXXXXXXXXXXAGAGCCAATTATGCTTCAATTTTAATGGGTAATCTTCGATTTTGAAGCTGGTAAGTAAAGGGAAATGGAAAATTCAGAAA

>Marker128925

ATCAGATCCAAGGTTGAGGATGAGCAGTCTGATGTTTTCTTTGCAACACTGTAGGGACAAGTGTTTGTCCTAAAAACAACXXXXXXXXXXTAAAAGAACCAGGCTGAGCAATCATTACCGTTCTCTGCGCTTCTCTAAAAACCGAGCCAAGGATGCCTTTCGAGCCTGAG

ATCAGATCCAAGGTTGAGGATGAGCAGTCTGATGTTTTCTTTGCAACACTGTAGGGACAAGTGTTTGTCCTAAAAACAACXXXXXXXXXXTAAAAGAACCAGGCTGAGCAATCATTACCGTTCTCTGCGCTTCTCTAAAAACCGAGCCAAGGATGCCTTTCGAGCCTAAG

>Marker129025

AACAAGTGATTTCATTTACTGTTGCAATGTAAGAATTTGGTTGAATCTTACTCTTACATTTAATTTTTTATTTTTCAACGXXXXXXXXXXTCTTGTGTCAAACATGCAGTTGGTTGTATGATAATCTCATCCCATCTCGATGTTTATATCAACTCTCATTTAGCTAAGTT

AACAAGTGATTTCATTTACTGTTGCAATGTAAGAATTTGGTTGAATCTTACTCTTACATTTAATTTTTTATTTTTCAACGXXXXXXXXXXTCTTGTGTCAAACATGCAGTTGGTTGTATGATAATGTCATCCCATCTCGATGTTTATATCAACTCTCATTTAGCTAAGTT

>Marker129054

TATGTTTCACATATTATTAAGTAGATGTCAAATATTTTCACATGCAAACAACAGGGTGCTCACAGATACTCTTCCAGAATXXXXXXXXXXTCAGATTCAAAAAGGATTGAACCAAGATGTGAGGAAAGAAACGAGCATATATCATTCGGGTAACTTCAACCGGTTGAAGA

TATGTTTCACATATTATTAAGTAGATGTCAAATATTTTCACATGCAAACAACAGGGTGCTCACAGATACTCTTCCAGAATXXXXXXXXXXTCAGATTCAAAAAGGATTGAACCAAGATGTGAGGCAAGAAACGAGCATATATCATTCGGGTAACTTCAAACGGTTGAAGA

>Marker129537

ACTCCTTTATAGCTATAAATAATAAATGCTCAAATCTTTTTCTCCAAATTCTCAAGTTTATGTCTCTTCTCCAATATTTTXXXXXXXXXXATAAAAATGCAAAACCAAAACAAAAAAATGAAATCAATTTTACAATCTAAATGCAGATTGATTGTATAAATGGAAGCGAA

ACTCCTCTATAGCTATAAACAATAAATGCTCAAATCTTTTTCTCCAAATTCTCAAGTTTATGTCTCTTCTCCAATATTTTXXXXXXXXXXATAAAAATGCAAAACCAAAACAAAAAAATGAAATCAATTTTACAATCTAAATGCAGATTGATTGTATAAATGGAAGCGAA

>Marker129627

CAAGATAGAAAAACTAGTCCTAGAAGTGCTACTAGAGGAAGCATCATTGTCAAGGATGTAGAGTCCCCTGCTGCAGTGCCXXXXXXXXXXCTGAGTTGTGTCCCAAATATCTTTAGCAGTTGTAGCGTATCTGACTTTCCAATATGTGGCTCCATCATGTTGATTAACGT

CAAGATAGAAAAACTAGTCCTAGAAGTGCTACTAGAGGAAGCATCATTGTCAAGGATGTAGAGTCCCTTGCTGCAATGCCXXXXXXXXXXATGAGTTGTGTCCCAAATATCTTTAGCAGTTGTAGCGTATCTGACTTTCCAATATGTGGCTCCATCATGTTGATTAATGT

>Marker129639

TCACTCGTAATTTATTGTAGGAGTTAGTGTGGTCCATAACTCATGCAAGTGAAGAAAGCACCGAATATATATATTCAACTXXXXXXXXXXATGTGATTTGGGCTGATTTTGTTTAGGCACCAGTCAACCCACTTGATGGGTTTCATAAATTTAGTTCTATCAATACTTTA

TCACTCGTAATTTATTGTAGGAGTTAGTGTGGTCCATAACTCATGCAAGTGAAGAAAGCACCGAATATATATATTCAACTXXXXXXXXXXATGTGATTTGGGCTGATTTTGTTTAGGCACCAGTCAACCCACTTGATTGGTTTCATAAATTTAGTTCTATCAATACTTTA

>Marker129687

CTCTACATGTATTTCCAACCCTTTGATGGAATTGCCTATTGTCTCCATCAATATTCTTTTCTCTCCACTTATCTTTGAGAXXXXXXXXXXGATGTTATGAAGTTAACAATGTCCATTTTGTCATTTTTGACAATTTCTTTATATAAAAACTAATTAAAATAAAAGATTAA

CTCTACATGTATTTCCAACCCTTTGATGGAATTGCCTATTGTCTCCATCAATATTCTTTTCTCTCTACTTATCTTTGAGAXXXXXXXXXXGATGTTATGAAGTTAATAATGTCCATTTTGTCATTTTTGACAATTTCTTTATATAAAAACTAATTAAAATAAAAGATTAA

>Marker129967

CCCGATGGAGTTGCCAGCTCGACCACTAGAGATGAAACTTGTAAACCAATCTCCTCTCATCTCTCTGCATTCCCTAAGAGXXXXXXXXXXGATCTCGAGGATGAGGAGGTGTCAGAATCATGGCGGTTGATCCTGCCATTCCCAGTTTTGACGTCATTGCTCTTTCAGTG

CCCGATGGAGTTGCCAGCTCGACCACTAGAGATGAAACTTGTAAACCAATCTCCTCTCATCTCTCTGCATTCCCTAAGAGXXXXXXXXXXGATCTCGAGGATGAGAAGGTGTCAGAATCATGGCGGTTGATCCTGCCATTCCCAGTTTTGACGTCATTGCTTTTTCAATG

>Marker129996

TTAATTAATCACTTAAACCAATCAAACTTATTTATTTAGTTTCCTTTAATTTTGACGTGGAAGATGTTGATTGGTGCTATXXXXXXXXXXTACAACATTAACTACTTTTGACTTTGTTATTATACTACAAATTTAATTCTAACTTATACAAAATAAATAAAAGAAAAAGA

TTAATTAATCACTTAAACCAATCACACTTATTTATTTAGTTTCCTTTAATTTTGACGTGGAAGATGTTGATTGGTGCTATXXXXXXXXXXTACAACATTAACTACTTTTGACTTTGTTATTATACTACAAATTTAATTCTAACTTATACAAAATAAATAAAAGAAAAAGA

>Marker130024

TTAATTATTTTTATTTAAATTGTTATGTAGGAGTGTAAGACCAACCCAAATAATCCGACAACTCGGACTACCTAACCCATXXXXXXXXXXAAGTTTCCTCGGGATCATCAACTAACACCAATACTACAAATTCTCAATACTCATGTGGAAGATTGTGAATCGAACAACTG

TTAATTATTTTTATTTAAATTGTTATGTAGGAGTGTAAGACCAACCCAAATAATCCGACAACTCGGACTACCTAATCCATXXXXXXXXXXAAGTTTCCTCGGGATCATCAACTAACACCAATACTACAAATTCTCAATACTCATGTGGAAGATTGTGAATCGAACAACTG

>Marker130605

ATTTCTTTTAGACATTCTTCCAATGGGTTTGGTCACCCCAGTTTTACAACTTCGGTCAAAATTTATGAGTAAGAAATTTAXXXXXXXXXXTTTTGTAGTGCAGTAATTCGAGTATCTTTGCAAAATATTATTCTTATGTATTTATTTTGCATATTATTTCCTTAAGTAAA

ATTTCTTTTAGACATTCTTCCAATGGGTTTGGTCACCCCATTTTTACAACTTCGGTCAAAATTTATGAGTAAGAAATTTAXXXXXXXXXXTTTTGTAGTGCAGTAATTCGAGTATCTTTGCAAAATATTATTCTTGTGTATTTATTTTGCATATTATTTCCTTAAGTAAA

>Marker130732

TTGTGAAAAGAAATTGAAAATTGTTAAAATTAGTTAAAAATACTTTCATTAATTTACAAGAATAGTCAAATACACACTCTXXXXXXXXXXTTCTATAAAGGACTCAATGGAGGAAAAAGGCAGAGAGACGTTAAAGAAAAGTGTTGAGAAAATTATAAAGAAGAGTTTAG

TTGTGAAAAGAAATTGAAAATTGTTAAAATTAGTTAAAAATAATTTCATTAATTTACAAGAATAGTCAAATACACACTCTXXXXXXXXXXTTCTATTAAGGACTCAATGGAGGAAAAAGGCAGAGAGACGTTAAAGAAAAGTGTTGAGAAAATTATAAAGAAGAGTTTAG

>Marker130803

TAGGATTACTGGGATAAAACCGAAAGTTCATTGCAGTTCATGATTAAAAATAAAATTTCCAGCAATATAGAAATTGTAAAXXXXXXXXXXAACTCCTGCTCTAACCTTCAATAGTAAATATGATACACAATCATTAAAAGAAACATGGATTCAAAACTCGAGTCCCGTTT

TAGGATTACTGGGATAAAACCGAAAGTTCATTGCAGTTCATGATTAAAAATAAAATTTCCAGCAATATAGAAATTGTAAAXXXXXXXXXXAACTCCTGCTCTAACCTTCAATAGTAAATATGATACACAATCATTAAAAGAAACATGGATTCAAAATTCGAGTCCCGTTT

>Marker131316

GTCAGTTAACCATTTTATTTTTCATTTTTAAAATCATTTCAACTACTATTTTCTTTTTCTTTTTAATCTACTATTTATCCXXXXXXXXXXTGATTACTAAATAGAATCTCGAGTTAAAATTGATAGGAACCAATAAAGCGAGCATTAGAGTCATCAAAATTGTAGATGGA

GTCAGTTAACCATTTTATTTTTCATTTTTAAAATCACTTCAACTACTATTTTCTTTTTCTTTTTAATCTACTATTTATCCXXXXXXXXXXTGATTACTAAATAGAATCTCGAGTTAAAATTGATAGGAACCAATAAAGCGAGCATTAGAGTCATCAAAATTGTAGATGGA

>Marker131358

TTATTGAGAACTTATGTATCACGCGATGAAGAGGCACATAGTAAGCACCTTACATGCTATGATTATGACCCACGACTCATXXXXXXXXXXGGTTCTGTAGTTTAGGGATATCTTGCAGTTGTTTCACGACTTCAACACACAATTCAACCTTTGTTGCACGCTCTTCTTTT

TTATTGAGAACTTATGTATCACGCGATGAAGAGGCACATAGTAAGCACCTTACATGCTATGATTATGACCCACGACTCATXXXXXXXXXXGGTTCTGTAGTTCAGGGATATCTTGCAGTTGTTTCACGACTTCAACACACAATTCAACCTTTGTTGCACGCTCTTCTTTT

>Marker131385

TCCAAACACAACCTAAGCCATGAGCCATGAGTATAATTATTACCATGTAAAAGAATGCACCCATGATAGGCTTTGATCATXXXXXXXXXXGTTAAATTGAGGAGTGGAATTGAATTAGTTAAAGCGAGAATCGAAGATACAAAGGGAGCTGCTTGTAGCTGAATGAACGA

TCCAAACACAACCTAAGCCATGAGCCATGAGTATAATTATTACCATGTAAAAGAATGCACCCATGATAGGCTTTGATCATXXXXXXXXXXGTTAAATTGAGGAGTGGAATTGAATTAGTTAAAGCGAGAATCGAAGATGCAAAGGGAGCTGCTTGTAGCTGAATGAACGA

>Marker131574

CACACTGCTATGCATGTTTTATCTTTTACATACAACATTTTTTCTTATGATCCAATTTATACATCAATAAGTATAATTTGXXXXXXXXXXAATATTGATTCAATTGCTCAAATCATATATATTTATCCATTTAATGAGCTATATTTGCATATTCTTGTAGATTTAAGAAG

CACACTGATATGCATGTTTTATCTTTTACATACAACATTTTTTCTTATGATCCAATTTATACATCGATAAGTATAATTTGXXXXXXXXXXAATATTGATTCAATTGCTCAAATCATATATATTTATCCATTTAATGAGCTATATTTGCATATTCTTGTAGATTTAAGAAG

>Marker131688

TATGTTTGACTGAGGGTGCAAAATATACAAAAGTTTGAGAGCTTCCTATGAAGCATAGTGTTATAAACTTTCAAATAATTXXXXXXXXXXATTGCAGTCTAAAGAGTGGCGGTTATCCAACTGCAGGAGAGAAGTAGAGGTGCTGTTGAGTATATTATCAATGAAAAGAA

TATGTTTGACTGAGGGTGCAAAATATACAAAAGTTTGAGAGCTTCCTATGAAGCATAGTGTTATAAACCTTCAAATAATTXXXXXXXXXXATTGCAGTCTAAAGAGTGGCGGTTATCCAACTGCAGGAGAGAAGTAGAGGTGCTGTTGAGTATATTATCAATGAAAAGAA

>Marker131874

ACCAATTTTTTTATTTTTTTGGTTAGAAAATGAGTTTTGATTGGAAATATAAAAGATTAAAATTACCTAAGTTGAGAATCXXXXXXXXXXCACAAAAATCCATTTCCCCATTTTCAAAATTCTCTAAAATTCTATCATTTTCGCCGGAATTTCTTCATCGACGCTACGAT

ACCAATTTTTTTATTTTTTTGGTTAGAAAATGAGTTTTGATTGGAAATATAAAAGATTAAAATTACCTAAGTTGAGAATCXXXXXXXXXXCACAAAAATCCATTTCCCCATTTTCAAAATTCTCTAAAATTCTATCATTTTCGCCGGAATTTCTTCATCGAAGCTACGAT

>Marker131878

TATTGAAACAAAGCAGTTTAAGAGATTAGAAAAATTGGATGGAACAATTTTGAAAACTAATGGATTATAAAGGAGATTTAXXXXXXXXXXTATATTAGAAATCTACTAATAATTGAAAATCACAATCAATGTAGAGTAAGATAAAAAACCAAAGAAATAAACAAAATTTG

TATTGAAACAAAGCAGTTTAAAAGATTAGAAAAATTGGATGGAACAATTTTGAAAACTAATGGATTATGAAGGAGATTTAXXXXXXXXXXTATATTAGAAATCTACTCATAATTGAAAATCATAATCAATGTAGAATAAGATAAAAAACCAAAGAAATAAACAAAATTTG

>Marker131933

TAAATCAATTATGATATCTCACATCCAACATTTTAACTTTTAATGTTATTAAGTAGAGAATTAATAGTATATTAGGTTTTXXXXXXXXXXTAGAGATAAGCTCTGACAAACAGAAAAAAAAAATCATCAAATGAGAGCACCGATGTTTAGAGCTATGACAGTATAGGAGA

TAAATCAATTATGATATCTCACATCCAACATTTTAACTTTTAATGTTATTAAGTAGAGACTTAATAGTATATTAGGTTTTXXXXXXXXXXTAGAGATAAGCTCTGACAAACAGAAAAAAAAAATCATCAAATGAGAGCACCGATGTTTAGAGCTATGACAGTATAGGAGA

>Marker132536

TTGAAGATTCATGAGCTAACCGATACAAAGAAGATGACGGTGGCAGTGATAAGTTTTGATGGACCTGCCCTGGACTGGTAXXXXXXXXXXATAGTTCTGGAAGAGACCTTTATGAATGGGCTAAGCCCATGGTTGAATTCTGAAGTGGATGTGTTGGAGCCCAGAGGGTT

TTGAAGATTCATGAGCTAACCGATACAAAGAAGATGACGGTGGCAATGATAAGTTTTGATGGACCTGCCCTGGACTGGTAXXXXXXXXXXATAGTTCTGGAAGAGACCTTTATGAATGGGCTAAGCCCATGGTTGAATTCTGAAGTGGATGTGTTGGAGCCCAGAGGGTT

>Marker133285

TAAAAAAATCTACCCTTTATAACAAATTCAGCCATGTTAGCATTTTCCAAAGGGTAATTATAATCAAGGGTAGTTTTAGAXXXXXXXXXXAAATAGATCCAGGCTTGACTCAGAGTCAAGAAGTCCAAACCCATTTATTCATTGGGTTGGGTTTTTTTGAAAATTGGTTG

TAAAAAAATCTACCCTTTATAACAAATTCAGCCATGTTAGCATTTTCCAAAGGGTAATTATAATCAAGGGTAGTTTTAGAXXXXXXXXXXAAATAGATCCAGGCTTGACTCAAAGTCAAGAAGTCCAAACCCATTTATTCATTGGGTTGGGTTTTTTTGAAAATTGGTTG

>Marker133295

TCATGCAAGGAACTCTATAAATAGATGACAACTTCAGTATTGAATTTCTCACAATCCACTCGTGCGTTTTGAATGAAATTXXXXXXXXXXAAACTACTTTGTAGAAGGAAAAATTTTCATTTGAGTTCTGGATTTTATGTTATTCAGCTCTAAAATAGAAGAAATATTAT

TCATGCAAGGAACTCTATAAATAGATGACAACTTCAGTATTGAATTTCTCACAATCCACTCGTGCGTTTTGAATGAAATTXXXXXXXXXXAAACTACTTTGTAGAAGGAAAAATTTTCATTTGAGTTCTGGATTTTATGTTATTCAGCTCTAAAATAGAAGAAATGTTAT

>Marker134123

AAACATGAGAAGGATATAGAAGATATTTTAAACCCTAATTGGTCTTGTGTTTTTGAATCACAACTTTAAATATGTTGTGTXXXXXXXXXXCTATAATATGAGTTATTCTCTCAAATAATAAACTACTTTTTCTTTCTTTGTGCATAGATGTAACTAAACACACATTGATA

AAACATGAGAAGGATATTGAAGATATTTTAAACCCTAATTGGTCTTATGTTTTTGAATCACAACTTTAAATATGTTGTGTXXXXXXXXXXCTATAATATGAGTTATTCTCTCAAATAATAAACTACTTTTTCTTTCTTTGTGCATAGATGTAACTAAACACACATTGATA

>Marker134159

TGCATATACACTTAGTATTCTTCATCGATACTCGATTGAATAAATACACTAGCTAGATATACTTGAAGTCCTACCAATATXXXXXXXXXXTGATACACTTAATACAAAATTTATTATATACTTAACCATAGCTACAATGAACTGACAAAAATTTTAAAAATAGAAATCAT

TGCATATACACTTAGTATTCTTCATCGATACTCGATTGAATAAATACACTAGCTAGATATACTTGAAGTCCTACCAATATXXXXXXXXXXTGATACACTTAATACAAAATTTATTATATACTTAACCATAACTACAATGAACTGACAAAAATTTTAAAAATAGAAATCAT

>Marker134491

AATCAATGATGAGAGGGATGAATCTATTACCTGGGATTTTAGTATAAAGACTACCAAAGAAGCTCAACACTATCCTTTCTXXXXXXXXXXTCATTTACGCCTATCGAATCTGCTATAGCGTCACAAATTTCCAGCTCCGCAAGGAGAGATTTTTCTTTTTCTTTAAGCTT

AATCAATGATGAGAGGGATGAATCTATTAGCTGGGATTTTAGTATAAAGACTACCAAAGAAGCTCAACACTATCCTTTCTXXXXXXXXXXTCATTTACGCCTATCGAATCTGCTATAGCGTCACAAATTTCCAGCTCCGCAAGGAGAGATTTTTCTTTTTCTTTAAGCTT

>Marker134639

TACAGCAAAGGGAGAAGCATCATCGTCAAAAAAAAGCTCCAAAGGGCAAAATGAGACAACAAAAAAGACGAGAATTATAAXXXXXXXXXXTTTTCACCGTTAAATGCTACAACCCCAACAAAAGAGCCTCGGTTCATTTGCACTCTATGAGGATGATAGTTACTAGCATG

TACAGCAAAGGGAGAAGCATCATCGTCAAAAAAAATCTCCAAAGGGCAAAATGAGACAACAAAAAAGACAAGAATTATAAXXXXXXXXXXTTTTCACCGTTAAATGCTACAACCCCAACAAAAGAGCCTCGGTTCATTTGCACTCTATGAGGATGATAGTTACTAGCATG

>Marker134696

TTAAGTTAAATTGTGTGTTTTAACTCGTTTTTAACGTATTTAAATCTTTTAAGAATTAAATGTAAAATTGAATGTCTAATXXXXXXXXXXTTAATTCCAACCTCAATTCCTACCCCTTAGTTAGTTATTGACATTTTTATCGCTAAGCCAACTCAACAAAAATACTATTA

TTAAGTTAAATTGTGTGTTTTAACACGTTTTTAACGTATTTAAATCTTTTAAGAATTAAATGTAAAATTGAATGTCTAATXXXXXXXXXXTTAATTCCAACCTCAATTCCTACCCCTTAGTTAGTTATTGACATTTTTATCGCTAAGCCAACTCAACAAAAATACTATTA

>Marker134760

ACAGCACAACCAAGACACACAACACAATACAGTTCACACACAATACATATATGAGAGGAGAAGAGATATAGGTATATTCAXXXXXXXXXXCCACTAGCCATCCTAACAACCATGTTGTCTAGTTTGAATTTCCCTGCTTACCCTTCCTTTGGTAAACTTGGGTGATTGGA

ACAGCACAACCAATACACACAACACAATACAGTTCACACACAATACATATATGAGAGGAGAAGAGATATAGGTATATTCAXXXXXXXXXXCCACTAGCCATCCTAACAACCATGTTGTCTAGGTTGAATTTCCCTGCTTACCCTTCCTTTGGTAAACTTGGGTGATTGGA

>Marker135080

TTAGAGCTCGAATGAATGCCGAGTATAAAGAAACCGTCGAGCGAAGGTAATTCTCAAATCTCAATCCCAAACCAACTTCAXXXXXXXXXXTAAATTTTATTATATCTACAAATTACTTTATATGATTGGGTTGATTGTTATATTCCCTTCCACAAATAACATAAAAATGA

TTAGAGCTCGAATGAATGCCGAGTATAAAGAAACCGTCGAGCGAAGGTAATTCTCAAATCTCAATCCCAAACCAACTTCAXXXXXXXXXXTAAATTTTATTATATCTACAAATTACTTTATATGATTGGGTTGATTGTTATATTCCCTTCGACAAATAACATAAAAATGA

>Marker135402

AAAAGGATGAGTTAAAAGGATAAGAGTTGTGTATGAAAGTTAAAGCAGAAAGGAGAACATATTTAAAGAAAGGAGTATGGXXXXXXXXXXCAAAAAATTTGAACAAAATTTTATCAAATATATGATGTGTTACCATTGTCTTGAGTCGCTTCAGAGAGTTTCTTCAGAGA

AAAAAGATGAGTTAAAAGGATAAGAGTTGTGTATGAAAGTTAAAGCAGAAAGGAGAACATATTTAAAGAAAGGAGTATGGXXXXXXXXXXCAAGAAATTTGAACAAAATTTTATCAAATATGTGATGTGTTACCATTGTCTTGAGTCGCTTCAGAGAGTTTCTTAAGAGA

>Marker135570

CCTTGTTGAAGAGTCAGGCGAGTTAAGATTATTGAAAAACTTCTCTGGTTAGTTGGAAGTTGAACACTGATGCTTCTTTAXXXXXXXXXXCATTATTAATGAAAATGGACCCATCACCAAATTTGATTGAGAGGATTTTTCTTATAGCATTTATAAAAACAAAATTTTAG

CCTTGTTGAAGAGTCAGGCGAGTTAAGATTATTGAAAAACTCCTCTGGTTAGTTGGAAGTTGAACACTAATGCTTCTTTAXXXXXXXXXXCATTATTAATGAAAATGGACCCATCACCAAATTTGATAGAGAGGATTTTTCTTATAGCATTTATAAAAACAAAATTTTAG

>Marker135659

TATGGTGTGATATTCAAAGGTTCTCTGATAGAAGTTGTCAGATGTAACACTTGCGGAAGAAGAAAGAGTTATCAACTAGAXXXXXXXXXXTGACAACTAAAGAGTCAACCCTATAAAGATTATGAGGTCCCTTGATTAAGGTGAGATCCTCAACATTCTCTCTCAAGATG

TATGGTGTGATATTCAAAGGTTTTCTGATAGAAGTTGTCAGATGTAACACTTGCGGAAGAAGAAAGAGTTATCAACTAGAXXXXXXXXXXTGACAACTAAAGAGTCAACCCTATAAAGATTATGAGGTCCCTTGATTAAGGTGAGTTCCTCAACATTCTCTCTCAAGATG

>Marker135698

CAATTTCCCCCCAACAATCTGATTTGGAGAAGAAAATCCAGCAGGAATTGCTGGAGGGTGAGGCAAGAAGGTTGGTTGTGXXXXXXXXXXTGAGTTTCTGAGCCTCATCCCTTAGCTGTATAATCATTCGAACTGCATCACCCAAAATAGCAGACTTGTCAATTTTGGGA

CAATTTCCCCCCAACAATCTGATTTGGAGAAGAAAATCCAGCAGGAATTGCTGGAGGGTGAGGCAAGAAGGTTGGTTGTGXXXXXXXXXXTGAGTTTCTGAGCCTCATCCCTTAGCTGTATAATCATTCGAACTGCATCACCCAAAATAGCAGACTTATCAATTTTGGGA

>Marker135701

ATATGATCAAGTTAAAGTTCTGATGGGTGCACTACTCATTGTGCAGATCGTGATAGCTGATCCTACATGACTTTATTTTTXXXXXXXXXXGAGAGCGAGCTCTCATGGCTCTTGAAACTAAAAGAGCTCAGCAAGAAGCTGCTGCAATAAAGTCCAAGATAGAAACTGTT

ATGTGATCAAGTTAAAGTTCTGATGGGTGCACTACTCATTGTGCAGATCGTGATAGCTGATCCTACATGACTTTATTTTTXXXXXXXXXXGAGAGCGAGCTCTCATGGCTCTTGAAACTAAAAGAGCTCAGCAAGAAGCTGCTGCAATAAAGTCCAAGATAGAAACTGTT

>Marker135721

TGATAGACTGAAAGAGTTACCAAAAAAGATCTCCAACTATTCAGTTTTGAATACAGAGAATACCTATGAAATATTTTCTAXXXXXXXXXXGATTCTTGGGTAAGCAGTCCTTCCTTTCCTAAAGAAAATTTTCTGTTGCAGCTTTGTTTCAAGAGATATAGAAGAGTTGT

TGATAGACTGAAAGAGTTACCAAAAAAGATCTCCAACTATTCAGTTTTGAAGACAGAGAATACCTATGAAATATTTTCTAXXXXXXXXXXGATTCTTGGGTAAGCAGTCCTTCCTTTCCTAAAGAAAATTTTCTGTTGCAGCTTTGTTTCAAGAGATATAGAAGAGTTGT

>Marker136113

ACTTTCAAAAACTTATGAAGATTACTCTATTTAAGAACTAAACTTGAATAGGCGGGCATTCATTTTGTTATGGTATATATXXXXXXXXXXGATTAGAACATTCGTCAATTTTGTTATTCTTTAACAACTACATTCCATTAGGGTTTAAGTTTGGGCAACCAAGTTCGAGT

ACTTTCAAAAACTTATGAAGATTACTCTATTTAAGAACTAAACTTGAATAGGCGGGCATTCATTTTGTTATGGTGTATATXXXXXXXXXXGATTAGAACATTCGTCAATTTTGTTATTCTTTAACAACTACATTCCATTAGGGTTTAAGTTTGGGCAACCAAGTTCGAGT

>Marker136518

TCATTTTTTCTTGTTATTTTCTTGTCTTTTATTTTTAGTATTTTTTTCCCCCACTTTCTTATGTTGGTATTTTCCCTTTCXXXXXXXXXXATATCGTGCTTCCAGACTTTCTTAATTAAGAATTATAATAACCAACTTCTTATCCCATCTAATTTATAATATAGATGTAT

TCATTTTTTCTTGTTATTTTCTTGTCTTTTATTTTTAGTATTTTTTTCCCCCACTTTCTTATGTTGGTATTTTCCCTTTCXXXXXXXXXXATATCTTGCTTTCAGACTTTCTTAATTAAGAATTATAATAACCAACTTCTTATCCCATCTAATTTATAATATAGATGTAT

>Marker136701

ATGTTCACTGATTCATAATCAAAATTATTCATATTGTAATAGGTCTCTTTTGATGTATGAGTCGTAACGAGTCTCAAACAXXXXXXXXXXATGGATGAATTACGTTTTAAGGAAAATGCAAAGCAATCAAACCTTTCTTGTTGATGACTTCAGGGGCAACATAAGCAGGG

ATGTTCACTGATTCATAATCAAAATTATTCATATTGTAATAGGTCTCTTTTGATGTATGACTCGTAACGAGTCTCAAACAXXXXXXXXXXATGGATGAATTACGTTTTAAGGAAAATGCAAAGCAATCAAACCTTTCTTGTTGATGACTTCAGGGGCAACATAAGCAGGG

>Marker137086

ATCCATGATATAACAAGTTACGCCCTTCATTCATCAACTCTTAAAAAATGTTACTAATATCCATTTCATCGACATCTTCTXXXXXXXXXXTTATTATATAAAGAAGATATTCCTTATGTAAATAAATGACGCTCTACTCCATCCAATGTTTGTGTCATTGAACTTTGACG

ATCCATGATATAACAAGTTACGCCCTTCATTCATCAACTCTTCAAAAATGTTACTAATATCCATTTCATCGACATCTTCTXXXXXXXXXXTTATTATATAAAGAAGATATTCCTTATGTAAATAAATGACGCTCTACTCCATCCAATGTTTGTGTCATTGAACTTTGACG

>Marker137214

AACAAAAAATGAATCAGGGAAATTCTTCGATACGCAAGATGCCTGTGAGAGTGAATTCCAGTAGTCCTGACGTATGGAGTXXXXXXXXXXGAAGAAGGGTGAGGTGAGTTGCTACGTGTTGAGGTGGGCAGGCTAGTAGTTGAGATGAAGAAGGTATGCTAGGCGTGGAA

AACAAAAAATGAATCAGGGAAATTCTTCGATACGCAAGATGCCTGTGAGAGTGAATTCCAGTAGTCCTGACGTATGGAATXXXXXXXXXXGAAGAAGGGTGAGGTGAGTTGCTACGTGTTGAGGTGGGCAGGCTAGTAGTTGAGATGAAGAAGGTATGCTAGGCGTGGAA

>Marker137589

TTCTTCGCATTTGGAGATGACTTCTTCGTGTGAGTGAGAGTTTTTTATTTGGGGGAAAATTTTTATTTGGAAAAAAAATGXXXXXXXXXXAGAACTGTCGTAGTAGAGAGTTTTTCTTGTAGTGAATTCGATAAAATTTGCCTCTCTATTTCAAATTTTGAACAATCTCG

TTCTTCGCATTTGGAGATGACTTCTTCGTGTGAGTGAGAGTTTTTTATTTGGGGGAAAATTTTTATTTGGAAAAAACTGTXXXXXXXXXXAGAACTGTCGTAGTAGAGAGTTTTTCTTGTAGTGAATTCGATAAAATTTGCCTCTCTATTTCAAATTTTGAACAATCTCG

>Marker137668

TCCACACCAAAACAAAGCATCTTGAGATCGATACTTACTGTGTTCATGACTTGGTTTTTCAAAGAAAAATAGAAGTTCGTXXXXXXXXXXTTTTTGAGGTATACTTTGTTGTGGGTGAGTGTAGGGTAAGAATTTTTCCTCCCAAGCTTTCTATATATACTTCCAATAGT

TCCACACCAAAACAAAGCATCTTGAGATCGATACTTACTATGTTCATGACTTGGTTTTTCAAAGAAAAATAGAAGTTCGTXXXXXXXXXXTTTTTGAGGTATACTTTGTTGTGGGTGAGTGTAGGGTAAGAATTTTTCCTCCCAAACTTTCTATATATACTTCCAATAGT

>Marker137693

AAACAACTTGATATATATTTTTGTGGATGTCTTCCTTGATAATGTTTCGTCATTTAATAGATTGGGAGAAAGAGGCGCAAXXXXXXXXXXTTACACAATTTTTTTTTACAAAACTTTGACACTCAACCCATCTGCAATTTGACTGGTAAGAGTTTCTTCTTTCTCTCTAA

AAACAACTTGATATATATTTTTGTGGATGTCTTCCTTGTTAATGTTTCGTCATTTAATAGATTGGGAGAAAGAGGCGCAAXXXXXXXXXXTTACACAATTTTTTTTTACAAAACTTTGACACTCAACCCATCTGCAATTTGACTGGTAAGAGTTTCTTCTTTCTCTCTAA

>Marker137704

CATCGAGTTCTTTCTGCTTTAATAGAAGAGCATGATTGTGATGAATATTACCACCAAAGTGAAGGGAAGCACACTTTTCTXXXXXXXXXXATTTATCAAATTGTGATGTTGGACACACCAGTGAAATATGTTCAAATGATTCTTTCCAGCTACAATCTGGGGATTTTAAC

CATCGAGTTCTTTCTGCTTTAATAGAAGAGCATGATTGTGGTGAATATTACCACCAAAGTGAAGGGAAGCACACTTTTCTXXXXXXXXXXATTTATCAAATTGTGATGTTGGACACACCAGTGAAATATGTTCAAATGATTCTTTCCAGCTACAATCTGGGGATTTTAAC

>Marker137730

TACATCAGAAGATTTATATCTAATCTTGCGGGTCGATGTCAACTATTCCAAAGATTGATGAGAAAGGATGCAGTCTTTAAXXXXXXXXXXAGATAAGCTAAGACATTATATGCAAGCTTTCACTATACATTTAGTGGCAAAAGTTGATCCTGTTAAGTATATCTTATCAA

TACATCAGAAGATTTAGATCTAATCTTGCAGGTCGATGTCAACTATTCCAAAGATTGATGAGAAAGGATGCATCTTTAATXXXXXXXXXXAGATAAGCTAAGACATTATATGCAAGCTTTCACTATACATTTAGTGGCAAAAGCTGATCCTATTAAGTATATCTTATCAA

>Marker137754

CAAAACATTATATTTCAAATTGTAGAATCAAACGACGATTAATTGATAATTATCCTTGTCGTTCTCATTAGTCTCCTCACXXXXXXXXXXATATATTTTTCCTCTCTTCTAAATGTTGTATATAATATCTCTTAACATTAAAGAATATAAATTGATTCGCTATAAAAATG

CAAAACATTATATTTCAAATTCTAGAATCAAACGACGATTAATTGATAATTATCCTTGTCGTTCTCATTAGTCTCCTCACXXXXXXXXXXATTTATTTTTCCTCTCTTCTAAATGTTGTATATAATATCTCTTAACATTAAAGAATATAAATTGATTCGCTATAAAAATG

>Marker137906

ATTTATTTATGGACATTTATACTTACGCAACAGCAAAGAAGGAGTAGCCATCTAAGTGCCATGACTGGAGGCTCTTTTCCXXXXXXXXXXCAACGTTAGTGGCAGAATTGACTATCTTGATGGTGCGAGTAATGTTGATAGAACCATAGTGATACGAGCCTTGAGGATTG

ATTTACTTGTGGACATTTATACTTACGCAACAGCAAAGAAGGAGTAGCCATCTAAGTGCCATGACTGGAGGCTCTTTTCCXXXXXXXXXXCAACGTTAGTGGCAGAGTTCACTATCTTGATGGTGCGAGTAATGTTGATAGAACCATAGTGATACGAGCCTTGAGGATTG

>Marker138661

AACCCAGAAGCAAGTTCAAAACAGAATTAAGAAACAAAGATGGAAATTCAAGATTACCAATGCTAAACTCAAAGACGACAXXXXXXXXXXCTATGAGAAACGGAGGAACAACAGGAAGGATTCTCATGAATGGTTTGAATGGCGTTGAAACCCAGGTCGGGAAATTCCTT

AACCCAGAAGCAAATTCAAAACAGAATTAAGAAACAAAGATGGAAATTCAAGATTACCAATGCTAAACTCAAAGACGACAXXXXXXXXXXCTATGAGAAACGGAGGAACAACAGGAAGGATTCTCATGAATGGTTTGAATGGCGTTGAAACCCAGGTCGGGAAATTCCTT

>Marker138738

TTTCATGTTAGCAATGCTTCAACCTAACTGAAAAAAGTGTTTGAAGGAGGTAAATGATGTTAAACCAAGGCTACAAACATXXXXXXXXXXGGATGTGACAAAACAGACAGAAACAAGAGGAAGAAAGGTATATAAAACTAACTTAAGTAATGATGAGCCTGTAAACTTTG

TTTCATGTTAGCAATGCTTCAATCTAACTGAAAAAAGTGTTTGAAGGAGGTAAATGATGTTAAACCAAGGCTACAAACATXXXXXXXXXXGGATGTGATAAAACAGACAGAAACAAGAGGAAGAAAGGTATATAAAACTAACTTAAGTAATGATGAGCCTGTAAACTTTG

>Marker139708

TAGAGTTTACTCTTGGAAAGAAGTGGTTAAACTTTCAAATCTCTCAAAGAGCTTGACGGTTGTATTCTTGCTGTTTCGTGXXXXXXXXXXACATTCCAAACTTCTTCAACAAATGCCAATATACTCCAAATATTGATAGACACTTTCTCAACGAAAAGTAAAATATGAAT

TAGAGTTTACTCTTGGAAAGAAGTGGCTAAACTTTCAAATCTCTCAAAGAGCTTGACGGTTGTATTCTTGCTGTTTCGTGXXXXXXXXXXACATTCCAAACTTCTTCAACAAATGCCAATATACTCCAAATATTGATAGACACTTTCTCAACGATAAGTAAAATATGAAT

>Marker139741

AGAGATATGGAAAGACTCGGGAGGAAAAGTCGATGCTTTGGTTGTAGGGATTGGAACTGGAGGGACAATTACTGGTGCAGXXXXXXXXXXGTATTGAATTGTGAGAAAGCAAATGCAATTTTCAAAAAGTAAACAGAATCAAACGAGGCTGAAGGGGTCATTATTAAATG

AGAGATATGGAAAGACTCGGGAGGAAAAGTCGATGCTTTGGTTGTAGGGATTGGAACTGGAGGGACAATTACTGGTGCAGXXXXXXXXXXGTATTGAATTGTGAGAAAGCAAATTCAATTTTCAAAAAGTAAACAGAATCAAACGAGGCTGAAGGGGTCATTATTAAATG

>Marker139997

TACTTGATTTGCTTAACCTTACCTTGGTGTTGTTGAGAGGGCAATTTAGGAATTTCACTTTGCAAGTTTCCACTTAGTAGXXXXXXXXXXTTTATACATACACTTAATTCTTTTATCAATCTAACATTTTTGTTAGTGTGTTATGTGGGATGACAATTTACAATCCTTTA

TACTTGATTTGCTTAACCTTACCTTGGTGTTGTTGAGAGGGCAACTTAGGAATTTCACTTTGCAAGTTTCCACTTAGTAGXXXXXXXXXXTTTATACATACACTTAATTCTTTTATCAATCTAACATTTTTGTTAGTGTGTTATGTGGGATGACAATTTACAATCCTTTA

>Marker140018

TGTAATCGGATGTCATAAAGTATTTCTCCTTCTTTGCAGTAAGGGCAATACAGGTGATGTTCACAGCTTTGTTTGTGAAAXXXXXXXXXXTCAATGAGCCAAAGATCTCTTCTATTATCAATATAAATGTCTTTAAAGTGACCAAGGCTTTTTCGAACTCCTCATCTATT

TGTAATCGGATGTCATAAAGCATTTCTCCTTCTTTGCAGTAAGGGCAATACAGGTGCTGTTCACAGCTTTGTTTGTGAAAXXXXXXXXXXTCAATCAGCCAAAGATCTCTTCTATTATCAATATAAATGTCTTTAAAGTGACCAAGGCTTTTTCGAACTCCTCATCTATT

>Marker140076

TAGATATAAAGTAGCAAATTAATAGTAGTATTGTTATTAGAAATGTAATGAGTTTAGCTTAACCGACATTCTAGAGGTCAXXXXXXXXXXAAATGTTTTTGATCTTTTTTATTTTTTATATTCGCTAACTGTAAAACTAATGAAAATTTACCATTTTTAGTGGTAAAAAT

TAGATATAAAGTAGCAAATTAATAGTAGTATTGTTATTAGAAATGTAATGAGTTTAGCTTAACCGACATTCTAGAGGTCAXXXXXXXXXXAAATGTTTTTGATCTTTTTTATTTTTTATATTAGCTAACTATAAAACTAATGAAAATTTACCATTTTTAGTGGTAAAAAT

>Marker140414

ACACTTTGGATAGGGAGAAGGGTAGTTTGGGAAACAATATTTTTATGAACCTTTTTGCAACAAAGTGCTATAGGGTGGAAXXXXXXXXXXATGTAGTGTTTTATTAATTATTATTAAAGAAAGAAGGTGTGGAGATAAGAATAAGAAAGACCTGATGTTTGCGGTGATTG

ACACTTTGGATAGGTAGAAGGGTAGTTTGGGAAACAATATTTTTATGAACCTTTTTGCAACAAAGTGCTATAGGGTGGAAXXXXXXXXXXATGTAGTGTTTTATTAATTATTATTAAAGAAAGAAGGTGTGGAGATAAGAATAAGAAAGCCCTGAGGTTTGCGGTGATTG

>Marker140783

AATCCAGGGCTGGTTGTCCCACAGCTTTATGTCCTAAGAAGGAAAGGGATAACATTATACAATTCAGCCAACCAAAATTTXXXXXXXXXXTTAGTTTTCAAAACTTGGTTTGGTTTTGAAACATTGGTAAAAGGTAGATAACAAAATAAGAGACTTAAAGGTGAAAAATG

AATCCAGGGCTGGTTGTCCCACAGCTTTATGTCCTAAGAAGGAAATGGACAACATTATACAATTCAGCCAACCAAAATTTXXXXXXXXXXTTAGTTTTCAAAACTTGGTTTGGTTTTGAAACATTGGTAAAAGGTAGATAACAAAATAAGAGACTTAAAGGTGAAAAATG

>Marker141011

ACTCCGTGTGGTTATTTACGTGTTCCTGTAGCTAGATTCACAAGGTTATTAGGCACGAAATATTCACCTAATAGAAAGTTXXXXXXXXXXTTTTTAAAATATTTTTATTCTTAATTCAATCCAAACAAACTTGAACATATTGACATAGAAAATAACGTGAAAATGGGTAA

ACTCCGTGTGGTTATTTACGTGTTCCTGTAGCTAGATTCACAAGGTTATTAGGCACGAAATATTCACCTAATAGAAAGTTXXXXXXXXXXTTTTAAAAATATTTTTATTCTTAATTCAATCCAAACAAACTTGAACATATTGACATAGAAAATAACGTGAAAATGGGTAA

>Marker141108

TAGAATGGACAAACAAAATTTTGTCATTCTATGTTATTTATTGAGGACAACTGCTGGTTTGATGTTGACGGAAATTGTCGXXXXXXXXXXCATTCTTCAATTTTGTTACTTATGAATCATGAAGTCCAACTACATGAATGCACTATGTTTCAATATGCCAAACTGCCTTG

TAGAATGGACAAACAAAATTTTGTCATTCTATGTTATTTATTGAGGACGACCGCTGGTTTGATGTCGACGGAAATTGTCGXXXXXXXXXXCATTCTTCAATTTTGTTACTTATGAATCATGAAGTCCAACTACTTGAATGCACTATGTTTCAATATGCCAAACTGCCTTG

>Marker141208

TCAAAGAAATTAGAAGTTATTATGTGCGCTACCAACAAGTTACACAATTTTTGTTGGTGGTTTAATCATATGATTTCAAAXXXXXXXXXXTTAATTGAACTATCATTAATTGATCAGCTAGTGGTCAATAATGGAGTCATCAATTTGATACAGGAAATGAGAGAGAATCG

TCAAAGAAATTAGAAGTTATTATGTGCGCTACCAACAAGTTACACAATTTTTGTTGGTGGTTTAATCATATGATTTCAAAXXXXXXXXXXTTAATTGAACTATCATTAATTGATCAGCTCGTGGTCAATAATGGAGTCATCAATTTGATACAGGAAATGAGAGAGAATCG

>Marker141782

CCGTTACTTAAACAATCCCTTAGCTTAGGAAGGGGAATTCTTCATCACCCTTCTTTTGGTATAACTTTCTTGTCTAATAAXXXXXXXXXXCTTGCTGGTGCTTCAACCAAAAAGACCTCTTTTGTAATTATAGTGTCTATTACAAATTAGAAAGCTTTTCTGTGATAGTT

CCGTTACTTAAACAATCCCTTAGCTTAGGAAGGGGAATTCTTCATCACCCTTTTTTTGGTATAACTTTCTTGTCTAATAAXXXXXXXXXXCTTGCTGGTGCTTAAACCAAAAAGACCTCTTTTGTAATTATAGTGTCTATTACAAATTAGAAAGCTTTTCTGTGATAGTT

>Marker141897

TACCATTGGCATGCAGAGATGGAAGACGTGATATCTGTATTTGAGAGTAGAACAATGAAACTCCACACAACAAGAAGTTGXXXXXXXXXXTTGCCACTTGGTGTGATTGTTGAATTCGATAAATGAGTTTTCTTATGTTCAGGGATTCAATTACAAACTATTTTGGTTTT

TACCATTGGCATGCAGAGATGGAAGACGTGATATCTGTATTTGAGAGTAGAACAATGAAACTCCACACAACAAGAAGTTGXXXXXXXXXXTTGCCACTTGGTGTGATTGTTGAATTCCATAAATGAGTTTTCTTATGTTCAGGGATTCAATTACAAACTATTTTGGTTTT

>Marker141955

CAACAAGGGTTACAGAATTAGCCGAGGAATTTTTTAATTCAAAGTCCACCCCTAACCTCAAATGGACAGTCGAACCTAACXXXXXXXXXXCCAAAACAAGTTGGGTAAGTCAATCCAAAATTGGGTTGATAAAAACTCAAAAGAAGACAGATGTCTCAAGGCAAAGCTAA

CAACAAGGGTTACAGAATTAGCCGAGGAATTTTTTAATTCAAAGTCCACCCCTAACCTCAAATGGACAGTCAAACCTAACXXXXXXXXXXCCAAAACAAGTTGGGTAAGTCAATCCAAAATTGGGTTGATAAAAACTCAAAAGAAGACAGATGTCTCAAGGCAAAGCTAA

>Marker142007

ATCATATTCTGAAAGAATACTTACCCTCAACCCTCCTTCTTTTACGCCAATAGATAAAGCAAGCAACAAAAATCAAGAAGXXXXXXXXXXGGAAGCATTTTCATATCCACCAATCTCTCAAACCATATGATGCAGCCATTGTCTCCTGTGGAAAACTCCATCCTTCCGTA

ATCATATGCTGAAAGAATACTTACCCTCAACCCTCCTTCTTTTACGCCAATAGATAAAGCAAGCAACAAAAATCAAGAAGXXXXXXXXXXGGAAGCATTTTCATATCCACCAATCTCTCAAACCATATGATGCAGCCATTGTCTCCTGTGGAAAACTCCATCCTTCCGTA

>Marker142198

AAAATATCCGACATGATTTCTATTAACCAAAAACAGAAAAAACATTTGTGCTAAAAATGATAGGATCTCTCGATCAGCCTXXXXXXXXXXCTTTGCGAAGCCAGTTGATAAAATCTCCGGGTTCGAAAGAAGAGTGCAGCAGCAACAAGAAATCCCAAGAAAGCCACGAT

AAAATATCCGACATGATTTCTATTAACCAAAAACAGAAAAAACATTTGTGCTAAAAATGATAGGATCTCTCGATCAGCCTXXXXXXXXXXCTTTGGGAAGCCAGTTGATAAAATCTCCGGGTTCGAAAGAAGAGTGCAGCAGCAACAAGAAATCCCAAGAAAGCCACGAT

>Marker142267

TTCATGGCTAAGATAACTTTGGGTAGAACCACAGTCTTTGCCCTTTTATACTTGAAACTAACCGCCTCATGAGGGTTAAAXXXXXXXXXXGAATAAAAACTGAGGATATAAAAGAATGAGAGGACCCGAAGTCAAATAATACTAATCCAAAGTGCCCCAAGATTGGAAGC

TTCATGGCTAAGATAACTTTGGGTAGAACCACAGTCTTTGCCCTTTTATACTTGAAACTAACCGCCTCATGAGGGTTAAAXXXXXXXXXXGAATAAAAACTGAGGATATAAAAGAATGAGAGGACCCGAAGTCAAATAATACTAATGCAAAGTGCCCCAAGATTGGAAGC

>Marker142624

CCTTCTTTTAATTTTGAAATATTGGACCCAAATTCTTTCAACATATGAATCCAAATTCCCCGACAACACAACCACCAATAXXXXXXXXXXCGTTACATTGAACTAATTTCTTAAAATGTTGGAAAAAGAAGAATATTTTTAATTTCAAGATCTTGGCTTTTGATATACTA

CCTTCTTTTAATTTTGAAATATTGGACCCAAATTCTTTCAACATATGAATCCAAATTCCCCGACAACACAATCACCAATAXXXXXXXXXXCGTTACATTGAACTAATTTCTTAAAATGTTGGAAAAAGAAGAATATTTTTAATTTCAAGATCTTGGCTTTTGACATACTA

>Marker142862

ATTACTTCATGGTATTGAATGAATCCATAAAATCTTTCCTAAGTGACTAAGAAAAATTGTGTCTCCATTTGAATTACAAAXXXXXXXXXXCAAAATCAATCGAAATGTTGTTCTAAATGTTATCTGGAATAGAGACTTGTGACTAAAGATACACTCGTTCAAGCTATTAA

ATTACTTCATGGTATTGAATGAATCCATAAAATCTTTCCTAAGTGACTAAGAAAAATTGTGTCTCCATTTGAATTACAAAXXXXXXXXXXCAAAATCAATCGAAATGTTGTTCTAAATGTTATATGGAATAGAGACTTGTGACTAAAGATACACTCGTTCAAGCTATTAA

>Marker142918

AATTATATCTCGACTACCGAGTGATGTCTATGAATCATTAAGAGAGGCATTTGTGAAGCTGACGAGTAGCCTATCACCGGXXXXXXXXXXGGTAGCTTCCAACAACAAGGAATACGTGTTAGTTATGACTTGACAAACTCCCTCGTTGGATTCTCAACTAATAAATGTTA

AATTATATCTCGACTACCGAGTGATGTCTATGAATCATTAAGAGAGGCATTTGTGAAGCTGACGAGTAGCCTCTCACCGGXXXXXXXXXXGGTAGCTTCCAACAACAAGGAATACGTGTTAGTTATGACTTGACAAACTCCATCGTTGGATTCTCAACTAATAAATGTTA

>Marker142950

TTGAGTTGAAATATTATGACTATCTTACAGATACGATAGTCAATCATGCATATAGTGGCATTTTACAAATAGATTTTTGGXXXXXXXXXXTTCTTGTCGTAACATTTTTAGAACATTCTTTTGGGTTTTTGGATGGTTTGAAACATATATGAGGATTTTTTGTCTTAAAA

TTGAGTTGAAATATTATGACTATCTTACAGATACGATAGTCAATCATGCATATAGTGGCATTTTACAAATAGATTTTTGGXXXXXXXXXXTTCTTGTCGCAACATTTTTAGAACATTCTTTTGGGTTTTTGGATGGTTTGAAACATATATGAGGATTTTTTGTCTTAAAA

>Marker143464

AGCTCCAAAAGTTCAGCCCTTGAAGCAATTTTTTGGTTTCAGCTATCAAGTGAGTCTCGGCAGAGTCAGTAGGACATTCCXXXXXXXXXXTTAGCCTACCAGAGAAAGAAAGAATGATTCTCTGCAAATTTCAATTGCAAGAGGCTGATGTTAGAATAGACCAATGAATG

AGCTCCAAAAGTTCAGCCCTTGAAGCAATTTTTTGGTTTCAGCTATTAAGTGAGTCTCGGCAGAGTCAGTAGGACATTCCXXXXXXXXXXTTAGCCTACCAGAGAAAGAAAGAATGATTCTCTGCAAATTTCAATTGCAAGAGGCTGATGTTAGAATAGACCAATGAATG

>Marker143560

AGTATATTAACTTCAAATGCTTTCATTTTACCCACAATGTGATATATTGGTGGTTCCTTTTATGGAAAACATTCCTCATAXXXXXXXXXXCAATGTGTTTGATGAAATGCCTCAATGAAGCATTTAGTTGATTTTTAGTTGTTTTTTATTCGTTCGAATTAGGAAATCAA

AGTATATTAACTTCAAATGCTTTCATTTTACCCACAATGTGATATATTGGTGGTTCCTTTTATGGAAAACATTCCTCATAXXXXXXXXXXCAATGTGTTTGATGAAATGCCTCAATGAAGCATTTAGTTGATTTTTAATTGTTTTTTATTCGTTCGAATTAGGAAATCAA

>Marker143653

AATATTTGGTCTTTATTATCTTGATTTATTTACTCTTTTGAACAAATATTTTTTTTCATTGATGACTTTTGATGTTATGAXXXXXXXXXXAGAGATCAAATAATAAAGTTGAAGTCTTTATTATTATTATTATTTTAAACGGAAACAAGATTTTCATGAAATGATTGGAG

AATATTTGGTCTTTATTATCTTGATTTATTTACTCTTTTGAACAAATATTTTCTTTCATTGATGACTTTTGATGTTATGAXXXXXXXXXXAGAGATCAAATAATAAAGTTGAAGTCTTTATTATTATTATTATTTTAAACGGAAACAAGATTTTCATGAAATGATTGGAG

>Marker143708

ATGATGTCAATGTCTTGCTGTTACATTAAAAAGCCATTAAAGATCTCATCGATAATATATTTAGGTCCAATTTTGTATGAXXXXXXXXXXCAAATGGTTCAAATTTGATTCTTCAAACTTATCCGTTATAGAAATTGAACTTCCAAATGATAAAAGTTGAATGGTTGAAA

ATGATGTCAATGTCTTGCTGTTACATTAAAAAGCCATTAAAGATCTCATCGATAATATATTTAGGTCCAATTTTGTATGAXXXXXXXXXXCAAATGGTTCAAATTTGATTCCTCAAACTTATCCGTTATAGAAATTGAACTTCCAAATTATAAAAGTTGAATGGTTGAAA

>Marker143867

TCATTATATATATTTGTGTGTAAATTTGTAAGCATGAACTCTTCCAATATATTTTTAATTATATATTGATTTTCCATTAAXXXXXXXXXXAAATATTTTTTAAAAAAAATTAAAATGGGAAACAAAATAGTTGTGAAAGAGAATGAGGCTTGAGCCCATATGTAAAGTTG

TCATTATATATATTTGTGTGTAAATTTGTAAGCATGAACTCTTCGAATATATTTTTAATTATATATTGATTTTCCATTAAXXXXXXXXXXAAATATTTTTTAAAAAAAATTAAAATGGGAAACAAAATAGTTGTGAAAGAGAATGAGGCTTGAGCCCATATGTAAAGTTG

>Marker143881

CCACATTAATTCAATAGTGGATTTGTAAGTAAGTATTAAGTTATTGAGTGCTTCAGAAAAGAAAACACCAAAAGAGTTTCXXXXXXXXXXTTCTACATATATGTGCGAGCAAGAGCACTAAATAACCATGAAGCTGACACAATATGGCACAAGTTTATTGAGTAAGTATG

CCACATTAATTCAATAGTGGATTTGTAAGTAAGTATTAAGTTATTGAGTGCTTCAGAAAAGAAAACACAAAAAGAGTTTCXXXXXXXXXXTTCTACATATATGTGCGAGCAAGAGCACTAAATAACCATGAAGCTGACACAATATGGCACAAGTTTATTGAGTAAGTATG

>Marker143973

CAATACTTATTTAATCTCACATCAATAATTAACTCAATCACCAAAAATTATTTCCATCTTGCAAATTTCAAATACACATTXXXXXXXXXXGAGCACGTGTGAGGGCATTGCCAGCACACGCAATGAAATCCACACAAAAACTGACGTGGCGCCCCGCACGTGATGACAAG

CAAAACTTATTTAATCTCACATCAATAATTAACTCAATCACCAAAAATTATTTCCATCTTGCAAATTTCAAATACACATTXXXXXXXXXXGAGCACGTGTGAGGGCATTGCCAGCACACGCAATGAAATCCACACAAAAACTGACGTGGCGCCCTGCACGTGATGACAAG

>Marker144029

CACAATTTTAAAAAATTTGTAAATATAACAAAATTTGTCAAATTTTATCAATGATATAAGTTTATTACTGATAGCCCATGXXXXXXXXXXATCTATCAATCATATTTGCTATATTCATTCTACCAACCAAAATTTTGATATATATTTAATTATTATTCTTCAAATAACTA

CACAATTTTAAAAAATTTGTAAATATAACAAAATTTGTCAAATTTTATCAATGATATAAGTTTATTACTGATAGACCATGXXXXXXXXXXATCTATCAATCATATTTGCTATATTCATTCTACCAACCAAAATTTTGATATATATTTAATTATTATTCTTCAAATAACTA

>Marker144286

TAATTTGCTTGGAACAATAAAGAAATTTCATGAGTAACTAGCTCAATCCATAAAAAGAAATTTCGTATTTTCACAACATTXXXXXXXXXXCCAACGAAGAAATGCTAACAAGCCAACGAAGAATGAAGTGTTTAAAACCCAACAAAATTAAAGAACTAAATGAACTTAAT

TAATTTGCTTGGAACAATAGAGAAATTTCATGAGTAACTAGCTCAATCCATAAAAAGAAATTTCGTATTTTCACAACATTXXXXXXXXXXCCAACGAAGAAATGCTAACAAGCCAACGAAGAATGAAGTGTTTAAAACCCAACAAAATTAAAGAACTAAATGAACTTAAT

>Marker144413

TAACAACCAAATTAAGGCAAAATAGCATGGCGTCTTTGACAACCTGCCACATTTAGTATTTTTCTACCTATCTTAGTTAGXXXXXXXXXXGAGGTAAAATACTTCAAACAAATAATTATGTCAAGGGCAAAAGATAAACAAACACATTTTAACAAGATAAATGCTTCAAA

TAACAACCAAATTAAGCCAAAATAGCATGGAGTCTTTGACAACCTGCCACATTTAGTATTTTTTTACCTATCTTAGTTAGXXXXXXXXXXGAGGTAAAATACTTCAAACAAATAATTATGTCAAGGGCAAAAGATAAACAAACACATTTTAACAAGATAAATGCTTCAAA

>Marker144608

CTCTCACACTGCAAACTTCTTGAGAGTGAGAATGCCGTTGGAGTTGAAGAAATTTAAGAGAAAGTTGTAAGAGAGCGGCAXXXXXXXXXXCATATATATGAAAACATCTCAAATGTGGCACCTATGTGAAACTTAAAATTGTAGACTACGTATACGATTTATGGTATCAT

CTCTCACACTGCAAACTTCTTGAGAGTGAGAATGCCGTTGGAGTTGAAGAAATTTAAGAGAAAGTTGTAAGAGAGCGGCAXXXXXXXXXXCATATATATGAAAACATCTCCAATGTGGCACCTATGTGAAACTTAAAATTGTAGACTAGGTATACGATTTATGGTATCAT

>Marker144803

TTTCCTAGTGCCAATTCAACCTCCTCCCTCACCTGTCCAGCACACAATTAGCAAAGCTTACATCAAATTTTCAAAATTGCXXXXXXXXXXCCACCCTTATAACAGAAGTATTCAATATTCAAGAACTGTAGGAGAACTAAGTAAGATCCCAAAGCTGAGACCACAACCTA

TTTCCTAGTGCCAATTCAACCTCCTCCCTCACCTGTCCAGCACACAATTAGCAAAGCTTACATCAAATTTTCAAACTTGCXXXXXXXXXXCCACCCTTATAACAGAAGTATTCAATATTCAAGAACTGTAGGAGAACTAAGTAAGATCCCAAAGCTGAGACCACAACCTA

>Marker144868

CTTTGGTTTCTCACAAATTTCGTCCTTGCTGTTTGCTCCGCTGAAGAATGGTTACATAGTTAAGACGCTATAGGAAGTTGXXXXXXXXXXTAGAATCTGAAATATGTTGGCAGTAGAACTCGTTTATCTTGATTTTAAACTCTTGGAATTGGAAGGATGGAATCCTTTAG

CTTTGGTTTCTCACAAATTTCGTCCTTGCTTGTTGCTCCGCTGAAGAATGGTTACATAGTTAAGACGCTATAGGAAGTTGXXXXXXXXXXTAGAATCTGAAATATGTTGGCAGTAGAACTCGTTTATCTTGATTTTAAACTCTTGGAATTGGAAGGATGGAATCCTTTAG

>Marker145125

CCAACCATGTCCTTTTGAGTAGCCCAATCGTTTACCCAAAACAGTCTCGCATATCTTGTCCTCGTAGAGTGGTTGAGAACXXXXXXXXXXAACGATTTGATTTTGAAACCCACACTGGAAGTTTAAAATGTGCGAGATGGGTGCAAGATGCGTGCGAGATAAAATATAAA

CCAACCATGTCCTTTTGAGTAGCCCAATCGTTTACCCAAAACAGTCTCGCATATCTTGTCCTCGTAGAGTGGTTGAGAACXXXXXXXXXXAACAATTTGATTTTGAAACCCACAGTGGAAGTTTGAAATGTGCGAGATGGGTGCAAGATGCGTGCGAGATAAAATATAAA

>Marker145352

ACGTGGGATATAATCATTGAGTTGCACCTAAAATTCCATTAATACATGGTAACACAATTGATGAGATTAAGTGGTGTGCTXXXXXXXXXXTTTAGTTTTAGAGTTTAGGATCATGGAGTAGAGTGTGGTCCATTTAGATTAAGGATTTAGAATGGTAATCATCATTATGT

ACGTGGGATATAATCATTGAGTTGCACCTAAAATTCCATTAATACATGGTAACACAATTGATGAGATTAAGTGGTGTGCTXXXXXXXXXXTTTAGTTATAGAGTTTAGGATCATGGAGTAGAGTGTGGTCCATTTAGATTAAGGATTTAGAATGGTAATCATCATTATGT

>Marker145439

AATAGAGGATATTTGGGCGTTTTGCTTGAGGTTTGGGGGAGGTGGTGAGGCTTGTGGCTGACCTAGGCAAGTCAGTTGAGXXXXXXXXXXGTGAGGAAGATTAAGTTTGAGCTAAAGAAAGAGAGAAATGTCATTTTTCGAATAAAACAAAGTGGTGTGCACTTTTTGGA

AATAGAGGATATTTGGGCGTTTTGCTTGAGGTTTGGGGGAGGTGGTGAGGCTTGTGGCTGACCTAGGCAAGTCAGCTGAGXXXXXXXXXXGTGAGGAAGATTAAGTTTGAGCTAAAGAAAGAGAAAAATGTCATTTTTCGAATAAAACAAAGTGGTGTGCACTTTTTGGA

>Marker145678

TTGGTGTTCTCATTCCCTAATTTCATCCAATTTATTTACTTTTCTGAATAAGATCCCTTTCCTCCCTTCTATATAGTTTCXXXXXXXXXXTTCCCGAATCCAAGGAGTTCTCAATGAGTCTACAGCAATGTTTGTCCAACAGCCAACTATTGCAGAAACGAAAAGGGGAT

TTGGTGTTCTCATTCCCTAATTTCATCCAATTTATTTACTTTTCTGAATAAGATCCCTTTCCTCCCTTCTATATAGTTCCXXXXXXXXXXTTCCCGAATCCAAGGAGTTCTCAATGAGTCTACAGCAATGTTTGTCCAACAGCCAACTATTGCAGAAACGAAAAGGGGAT

>Marker145748

ACCAAATTTCTCTAAAAGTTACCTTAGTTCATCGTGGAAATTGAAGAAGCAAACAAATTTCGATGAACAAGAGTAAACATXXXXXXXXXXCTTTTGACATAATTTTAAAGTCCAAAGTGACGCGACGCCTGATTTTTACAAAATAATGAAACTACTATTGAACTTGTTTC

ACCAAATTTCTCTAAAAGTTACCTTAGTTCATCGTGGAAATTGAAGAAGCAAACAAATTTCGATGAACAAGAGTAGACATXXXXXXXXXXCTTTTGACATAATTTTAAAGTCCAAAGTGACGCGGCGCCTGATTTTTACAAAATAGTGAAACTACTATTGAACTTGTTTC

>Marker145843

AAAATGTTTCTGGAAGATGACTTTGTTTTTAACTAGCCAACTCTAACATCTCTTTCACTATCAACTATATGCCATCCCACXXXXXXXXXXTGGTTATTGACAATGAAATTTAGCTGATTGCTTCTTGATAATGTTATTCGTTCTCCTTTTATGATAGAGGCAAATAGAAC

AAAATGTTTCTGGAAGATGACTTTGTTTTTAATTAGCCAACTCTAACATCTCTTTCACTATCAACTATATGCCATCCCACXXXXXXXXXXAGGTTATTGACAATGAAATTTAGCTGATTGCTTCTTGATAATGTTATTCGTTCTTCTTTTATGATAGAGGCAAATAGAAC

>Marker145948

TTTTATGGAAGAAGTTCTGGAGTTGAACTTCCGAAGAGCTAGAAGAGGCATTCTTGACTTGGCTGAACCTGATGACAACTXXXXXXXXXXCCAGAGTTTAATACTATAGGTAGCTGGGTCTCTATGGTTTGCCAATATGTTGGTTTTGTAGCTTTGCCCTTTGATCACCA

TTTTATGGAAGAAGTTCTGGAGTTGAACTTCCGAAGAGCTAGAAGAGGCATTCTTGACTTGGCTGAACCTGATGACAACTXXXXXXXXXXTCAGAGTTTAATACTATAGGTAGCTGGGTCTCTATGGTTTGCCAATATGTTGGTTTTGTAGCTTTGCCCTTTGATCACCA

>Marker145961

CCATTGCCAATAACTATATCGATGACTATCTGATTTATTTGTTACAATATTCATAAGGGGAGATATTTTTTTTTTGGAAAXXXXXXXXXXGGACAATAATAATGTAGAGTTGCATGAAGAATTAATTTGGAGTGTATTTAGATAAGTCTAATCAAGAATACAAAAGGGAT

CCATTGCCAATAACTATATCGATGACTATTTGATTTATTTGTTACAATATTCATAAGGGGAGATATTTTTTTTTTTGGAAXXXXXXXXXXGGACAATAATAATGTAGAGTTGCATGAAGAATTAATTTGGAGTGTATTTAGATAAGTCTAATCAAGAATACAAAAGGGAT

>Marker146063

AAAGCAAGTCTAAACTTCTTGACCATTATGACACTGATCAACCAACAATGTCATAATATCGTGTGTATGGTGCATCTTCAXXXXXXXXXXATAAAAAAAATTATAAACTATAAATGGTTCAAGTATCTAATTACATAAAATTATAAGTGAATAAATTTATAAATAAAATA

AAAGCAAGTCCAAACTTCTTGACCATTATGACACTGATCAACCAACAATGTCATAATATCGTGTGTATGGTGCATCTTCAXXXXXXXXXXATAAAAAAAATTATAAACTATAAATGGTTCAAGTATCTAATTACATAAAATTATAAGTGAATAAATTTATAAATAAAATA

>Marker146119

TCTTACTCTATTTTTCCTTGTTCGATTGAAGCCGCGGTCGCTTGGGGACCTGGACAACCATTGGTGATTGAGGAGGTGGAXXXXXXXXXXTGTCCTGTGATTGGTTTTGTTTTGTTCAATGTGTATATGAAATTTATAGTAATATTGTTTGTATAATGAATGGAGACATT

TCTTACTCTATTTTTCCTTGTTCGATTGAAGCCGCGGTCGCTTGGGGACCTGGACAACCATTGGTGATTGAAGAGGTGGAXXXXXXXXXXTGTCCTGTGATTGGTTTTGTTTTGTTCAATGTGTATATGAAATTTATAGTAACATTGTTTGTATAATGAATGGAGACATT

>Marker146259

CATAAATAGCAATAAGCCACCAACTAGTATTGTTAATATCATTTGGGAAAGAAATTGTGATGGAGAGAGAAATTTTTCACXXXXXXXXXXTTGAGATCTTTTTGAAGGAGAGCTTAACCCTTTAGTGCTCCAACATACAACTTTCATGGGAAACTAGTTTGTGTCATTTG

CATAAATAGCAATAAGCCACCAACTAGTATTGTTAATATCATTTGGGAAAGAAATTGTGATGGAGAGAGAAATTTTTCACXXXXXXXXXXTTGAGCTCTTTTTGAGGGAGAGCTTAACCCTCTAGTGTTCCAACATACAACTTTCATGGGAAACTAGTTTGTGCCATTTG

>Marker146266

TTCGTGTATACTAGTAAATTTGGTCTAGAAAATCACATTTGTTTTTTGTTCAACATCACATTGTCTTGCTTCTGAAAGAAXXXXXXXXXXAGCTTAAGATTGTTGGAACCATCCCTTACATCTCTGATATTATATATCAATAAATATGCCTATGCAATTATCATGATTAG

TTCGTGTATACTAGTAAATTTGGTCTAGAAAATCACATTTGTTTTTTGTTCAACATCACATTGTCTTGCTTCTGAAAGAAXXXXXXXXXXAGCTTAAGATTGTTGGAACCATCCCTTACATCTCTGATATTCTATATCAATAAATATGCCTATGCAATTATCATGATTAG

>Marker146370

AGGAACTACCTATTTATCATGAAATTTGGGTATACAAAAGCATAGAGTGCTTCTGATCCAAGTCGACCAATATCATCACCXXXXXXXXXXTCCATCTAAATAGTTGTCACAAAAAACCTGCAAAAAACCAATGAAACGTATTAGCAAAACCCACAAGAATTGTGGTAACA

AGGAACTACCTATTTATCATGAAATTTGGGTATACAAAAGCATAGAGTGCTTCTGATCCAAGTCGACCATAATCATCACCXXXXXXXXXXTCCATCTAAATAGTTGTCACAAAAAACCTGCAAAAAACCAATGAAACGTATTAGCAAAACCCACAAGAATTGTGGTAACA

>Marker146781

CCACCTCTTGATCCCAATAACAATGAATGGATTTCAAAAACAATCATGTTTATTGAACTATATACGTGTATATGTCCACAXXXXXXXXXXTAGTCATCCATTCCAATTTCCCTTAAACAAATCTAAAAAGTTTACAGAATGATCTCCTTCATAAATATCTAATAAAGAAA

CCACCTCTTGATCCCAATAACAATGAATGGATTTCAAAAACAATCATGTTTATTGAACTATATACGTGTATATGTCTCCAXXXXXXXXXXTAGTCATTCATTCCAATTTCCCTTAAACAAATCTAAAAAGTTTACAGAATGATCTCCTTCATAAATATCTAATAAAGAAA

>Marker146859

TTTATTATTATTTGGATATTTGAGATGGGTCAAGTGGATGGATTAAGGGATTAAATCCAATCCCAACTTTTAGCATTTGAXXXXXXXXXXTTGTGATCTACCAACTTTAAGTGAAGAAAAATGAAAATTTCAATTCGTCAAATTGCACTTCAACTTCCATAACAAGTGTT

TTTATTATTATTTGGATATTTGAGATGGGTCAAGTGGATGGATTAAGGGATTAAATCCAATCCCAACTTTTAGCATTAGAXXXXXXXXXXTTGTGATCTACCAACTTTAAGTGAAGAAAAATGAAAATTTCAATTCGTCAAATTGCACTTCAACTTCCATAACAAGTGTT

>Marker146865

TGTTTTGAGAGTGTTATGTTTATAGAATGTTCGAATCGCTTTTAATGTGGGAGACCTTCACATTCCTTTTACAATCAAAAXXXXXXXXXXTAAACTTCACACCTCAAGTTGCTCCTAGAAAGCCTTCTACTAATAGGTGTTTATCAACCCGAAGGTCCTAACTCCTAGTG

TGTTTTGAGAGTGTTATGTTTATAGAATGTTCGAATCGCTTTTAATGTGGGAGACCTTCTCATTCCTTTTACAATCAAAAXXXXXXXXXXTAAACTTCACACCTCAAGTTGCTCCTAGAAAGCCTTCTACTAATAGGTGTTTATCAACCCGAAGGTCCTAACTCCTAGTG

>Marker147031

CAACGTAAGTAGTTCGTGTGACGAAACGAAGGCAACGAGAGGGCTGAAACTTCATTTGCTTAACCAAGAAAGACTCCAACXXXXXXXXXXGACGCCCTTCCACATTGGCTTCTTAGACGGGCAGTCCGGTGTGGAAGGGATAGTGGCTTGAATCTCAAGCACCACATTGT

CAACGTAAGTAGTTCGTGTGACGAAACGAAGGCAACGAGAGGGCTGAAACTTCATTTGCTTAACCAAGAAAGACTCCAACXXXXXXXXXXGACGCCCTTCCACATTGGCTTCTTAGACGGGCAGTCCGGTGTGGAAGGGATGGTGGCTTGAATCTCAAGCACCACATTGT

>Marker147073

TTTCAACATGCCATCTGCATAACACGAAATAAATTAATACATAATCATCAATTTGAACATAAACTAAATATATGCATACTXXXXXXXXXXAAAAAATCAAATTTATAATTGATCTTTTTAACCAAAACAATCAAATGATGATTATCATTAAAGAAACAAACCAACACTAT

TTTCAACATGCCATCTGCATAACACGAAATAAATTAATACATAATCATCAATTTGAACATAAACTAAATATATGCATACTXXXXXXXXXXAAAAAATCAAATTTATAATTGATCTTTTTAACCAAAACAATCAAATGATGATTATCATTAAAGAAACAAACCTACACTAT

>Marker147183

CTAATATTCTTCCAGCTGCAATCTTCCTCCTTTTGTCTAAGGGAACATCGTCTGGAACATCCTTTTTCTGCATGATTGTTXXXXXXXXXXGTATTTTCTAATTTCCCTAACATCCTAACAAATAGGTAAGGATCAAAGAGTAGAATGAACTCACAGCTGGACTACGAGGG

CTAATATTCTTCCAGCTGCAATCTTCCTCCTTTTGTCTAAGGGAACATCGTCTGGAACATCCTTTTTCTGCATGATTGTTXXXXXXXXXXGTATTTTCTAATTTCCCTAACATCCTAACAAATAGATAAGGATCAAAGAGTAGAATGAACTCACAGCTGGACTACGAGGG

>Marker147196

ACAACTATAGTGGGTGAAACTCTTTTTCTTGAAACGAAGGGATTTTTGGTTATAAGAGGAAGACTGGGACCGTTTACTTAXXXXXXXXXXATTTCACCCAATTTGGATTGGTTTGTCTCAAATGTAGCTTGAGCGTGCATTTTATTTAGTAAATTTTTTAAGCTGGACTT

ACAACTATAGTGGGTGAAACTCTTTTTCTTGAAACGAAGGGATTTTTGGTTATAAGAGGAAGACTGGGACCGTTTACTTAXXXXXXXXXXATTTCACCCAATTTGGATTGGTTTGTCTCAAATGTAGCTTGAGCGTGCATTTTATTTAGTAAATTTTTTGAGCTGGACTT

>Marker147202

GCATGCTCCGGGAAGAGCTCCAGCTACTTCAAGAGCCAGGATCTTATGTTGGTGAAGTTGTTAAAGTCATGGGAAAGAACXXXXXXXXXXTTTCTGTAAAGTTCAATATTCTGTAGTAAAAAGGTGAAAATGTGCTGAAGTTGCATGCCATCCGGTCCATGGTATTAAAA

GCATGCTCCGGGAAGAGCTCCAGCTACTTCAAGAGCCAGGATCTTATGTTGGTGAAGTTGTTAAAGTCATGGGAAAGAACXXXXXXXXXXTTTCTGTAAAGTTCAATATTCTGTAGTAAAAAGGTGAAAATGTGCTGAAGTTGCATACCATCCAGTCCATGGTATTAAAA

>Marker147338

ATATGATCAAATAAAGTCCATAAATGTAGAATAGCTGTTATATCATACCTTGTAAATTTCAGCATTCAGAAGGTGACATGXXXXXXXXXXTACCTTCTAATGTCGATGTCTAAATGATAAAGAAAAAAAGAAAGCAGATGCTTTGCATTCCAAATACTCAAAAAGGGAGT

ATATGATCAAATAAAGTCCATAAATGTAGAATAGCTGTTATATCATACCTTGTAAATTTCAGCATTCAGAAGGTGACATGXXXXXXXXXXTACCTTCTAATGTCAATGTCTAAATGATAAAGAAAAAAAGAAAGCAGATGCTTTGCATTCCAAATACTCAAAAAGGGAGT

>Marker147578

CATAGATCCATTTACCTCATGATCTACTACACTTCCTACCACTTTAATATCATTGAACTTGGTCCTTAAGTTTTTAAACTXXXXXXXXXXATCCTTTTTGGGTGTGTGATCATACTTGCAAGCAATACTATTAGTGCACAAAATCTATCAAAACCCCGAGTGTGAGAATA

CATAGATCCATTTACCTCATGATCTACTACACTTCCTACCACTTTAATATCATTGAATTTGGTCCTTAAGTTTTTAAAATXXXXXXXXXXATCCTTTTTGGGTGTGTGATCATACTTGCAAGTAATACTATTAGTGCACAAAATCTATCAAAACCCCGAGTGTGAGAATA

>Marker148126

CTAAAATTCAATTGGTCCCCAGTCATGCCATGTGGTCTTATGTTATTGCACACTACATGGAACTCCTTGATGGATTTGTGXXXXXXXXXXTTTTTATACTATCAAATGAAACTCAAAACGCAATGACAGTCAACCTTAAATGCCAAATGTGATAAATAACTTAACTAAGT

CTAAAATTCAATTGGTCCCTAGTCATTCCATGTGGTCTTATGTTATTGCACACTACATGGAACTCCTTGATGGATTTGTGXXXXXXXXXXTTTTTATACTATCAAATGAAACTCAAAACGCAATGACAGTCAACCTTAAATGCCAAATGTGATAAATAACTTAACTAAGT

>Marker148210

AAATCTATGGTCTAATAAGGAAAATCCATGGTTCTGGAAAAGGTATACGACCCACATGAGACTAAACTAATATCAACACAXXXXXXXXXXAAATGGTAGTTAATAATTTACTTTAAAAGTTTAGCAGGGGTTGATAATGAACCTGAGAAGATAAAACTTTAAGTAAAAGT

AAATCTATGGTCTAATAAGGAAAATCCATGGTTCTGGAAAAGGTATACGACCCACATGAGACTAAACTAATATCAACACAXXXXXXXXXXAAATGGTAGTTAATAATTTACTTTAAAAGTTTAGCAGGGGTTGATAATGAACGTGAGAAGATAAAACTCTAAGTAAAAGT

>Marker148280

TTCATCCTTGCTCATCCTTGAATGAAGATTCCGATTGGAGCATAGTCCTCCGAAGTCTCCTCCTCCCCCTTTTTGCAGCTXXXXXXXXXXGCTAGGGGGTGCTAACCTAGTTGAGATGTCTTGGTGCGCCTACTGGTCCTTAGGTTTATTGCTCTTTGTATATCCCTCAT

TTCATCCTTGCTCATCCTTGAATGAAGATTCCGATTGGAGCATAGTCCTCCGAAGTCTCCTCCTCCCCCTTTTTGCAGCAXXXXXXXXXXGCTAGGGGGTGCTAACCTAGTTGAGATGTCTTGGTGCGCCTACTGGTCCTTAGGTTTATTGCTCTTTGTATATCCCTCAT

>Marker148307

ACTATTTCTTAACATTGTGCATATTCTTAAGAAGTTGAGTTTAAGTTACGTTTAAGCTCGGACGTTCTGGCATTTTACTTXXXXXXXXXXCTGTCATACTTGACCATGCGTATACGGTTCGTTCTGACGTGTAAATCATTGATAGGCTAAAGCTCCTACCTTAACCGAGG

ACTATTTCTTAACATCGTGCATATTCTTAAGAAGTTGAGTTTAAGTTACGTTTAAGCTCGAACGTTCTGGCATTTTACTTXXXXXXXXXXCTGTCATACTTGACCATGCGTATACGGTTCGTTCTGACGTGTAAATCATTGATAGGCTAAAGCTCCTACCTTAACCGAGG

>Marker148563

CATTTTAAAAATTAAAATGCAAATAAAAATTCCTAATTGTAAATTTTCTTTTTTGGTCGAAATGGCTCTTTTTCATTCATXXXXXXXXXXTAAATAAAATCGTGAGTTTGTTGTATATACCACATTTTTACCAAACCATTTCTATTTCTAAATTCACTTTTAGCTCAAAT

CATTTTAAAAATTAAAATGCAAATAAAAATTCCTAATTGTAAATTTTCTTTTTTGGTCGAACTGGCTCTTTTTCATTCATXXXXXXXXXXTAAATAAAATCGTGAGTTCGTTGTATATACCACGTTTTTACCTCACCATTTCTATTTCTAAATTCACTTTTAGCTCAAAT

>Marker149170

CTTCTAATAGGTTTCTTGTTGAGACGAGACTACATAAGTGGAGGATGTGTTACATATTGTCTTTTGGTCTCAGGTATGGGXXXXXXXXXXTAGAATCTAGAGCACATTGATATATGAAGCTTCACATTTAACTACCTCGATCCAAACAATTGTAACAACGGGGTAAAATA

CTTCTAATAGGTTTCTTGTTGAGACGAGACTACATAAGTGGAGGATGTGTTACATATTGTCTTTTGGTCTCAGGTATGGGXXXXXXXXXXTAGAATCTAGAGCACATCGATACATGAAGCTTCACATTTAACTACCTCGATCCAAACAATTGTAACAACGGGGTAAAATA

>Marker149199

ACGATTGTTTAGATCATATCCACACGTTCGTGTAATTCTTTTTAAACAATGGAAAAAAGGCTTTAAATCTAAATATTTGTXXXXXXXXXXTTCTTAATTTTATTTATACTTCAATGTTTTGTTCTCATTAGTCACTTTTAATGTTCAAGACTTCTCTACAATCGATCATG

ACGATTGTTTAGATCATATCCACACATTCGTGTAATTCTTTTTAAACAATGGGAAAAAGGCTTTAAATCTAAATGTTTGTXXXXXXXXXXTTCTTAATTTTATTTATACTTCAATGTTTTGTTCTCATTAGTCACTTTTAATGTTCAAGACTTCTCTACAATCGATGATG

>Marker149230

ACCATTCTAAACTTATCTATAAGATACATAGTTCTAAAAAAACAAAAAACAAAACTAAACCCTAGACGACCCACCTTATTXXXXXXXXXXGATTTAACTCTGACTCACAAGTCAACAATCACAAAAATCAGTCTGATCGCTACCCAAAAAATAAAGTAATTATTGGAATT

ACCATTCTAAACTTATCTATAAGATACATAGTTCTAAAAAAACAAAAAACAAAACTAAACCCTAGACGACCCACCTTATTXXXXXXXXXXGATTTAACTCTGACTCACAAGTCAACAATCACAAAAATCAGTCTGATCTCTACCCAAAAAATAAAGTAATTATTGGAATT

>Marker149330

AGATATTGTGATAGATGCTTGCATTGAATCATCAAATATACTAATTTAGACTCGTTTAAAGTTGGTAGATTTTTTTTTGTXXXXXXXXXXATTGAATTGAAAAAAATTGGAAATGGCTTGTTAAATTTATTTCAATAATAAATATAAAAATGGTATATCCCCTTTCATTG

AGATATTGTGATAGATGCTTGCATTGAATCATCAAATATACTAATCTAGACTCGTTGAAAGTTGATAGATTTTTTTTTGTXXXXXXXXXXATTGAATTGAAAAAAATTGGAAATGGCTTGTTAAATTTATTTCAATAATAAATATAAAAATGGTATATCCCCTTTCATTG

>Marker149379

TAATATGAAGAAGATGTATAAGCATTCAATGTGAAATTCGAAACCAACCAAAAAATGACAATCAAATATGTGTCATTTATXXXXXXXXXXGTGATGAATTCAAAGAGATGAACTTCGATGGTTGTGAACATGAATTTTCTTCAAATACATTCAATGAGTTGGACATGAAT

TAATATGAAGAAGATGTATAAGCATTCAATGTGAAATTCGAAACCAACCAAAAAATGACAAGCAAATATGTGTCATTTATXXXXXXXXXXGTGATGAATTCAAAGAGATGAACTTCGATGGTTGTGAACATGAATTTTCTTCAAATACATTCAATGAGTTGGACATGAAT

>Marker149479

AACTTCCAAGATAGCACTCAATTCAATTTCTTCAGTTTCAACGTCCAGAGCATAGATTGCATAGTCATTTGGTTTATTTTXXXXXXXXXXAACATGGTAAAACTCATTAGTGGTAGGATTAAATATGCCATGAGACATTGTGTTGAGTTTGCTTTTGCAAACAAACAGTA

AACTTCCAAGATAGCACTCAATTCAATTTCTTCAGTTTCAACGTCCAGAGCATAGATTGCATAGTCATTTGGTTTATTTTXXXXXXXXXXAACATGGTAAAACTCATTAGTGGTAGGATTAAATATGCCATGACACATTGTGTTGAGTTTGCTTTTGCAAACAAACAGTA

>Marker149532

TTCTTTAATGTTGGAATTGGTTTTTTTAATCTCGTTTATCTCATGGTTTGTAGTTTTGGCAACATAGATTATTTATCTAAXXXXXXXXXXAATGGATAAAGATTAGGTGTCTTATCCTGATAATTTGCAGATACAACTCACACTTTATCATTGTTACATGAGACGTAAGT

TTCTTTAATGTTGGAATTGGTTTTTTGAATCTCGTTTATCTCATGGTTTGTAGTTTTGGCAACATAGATTATTTATCTAAXXXXXXXXXXAATGGATAAAGATTAGGTGTCTTATCCTGATAATTTGCAGATACAACTCACACTTTATCATTGTTACATGAGACGTAAGT

>Marker149796

ATAGAATTTTGGTGAAAGTTTTTCGCTCCTCTTCCTCGCCAAAGACCTTTGTCGGTAGGGTCTTAGTTTCAAGTAGACTTXXXXXXXXXXAAAACGATTTGAAAGGGGGTGGACATTGTTGATGCGTGGAAAATTGTGTTATACCACAATTTGGTCCAGGGTATGAATTT

ATAGAATTTTGGTGAAAGTTTTTCGCTCCTCTTCCTCACCAAAGACCTTTGTCGGTAGGGTCTTAGTTTCAAGTAGACTTXXXXXXXXXXAAAACGATTTGAAAGGGGGTGGACATTGTTGATGCGTGGAAAATTGTGTTATACCACAATTTGGTCCAGGGTATGAATTT

>Marker149803

GTTTTGTTTGTAATCACTATTTTTTGTTTTAATTTATTCCATTGTATTTTGAGCTTTAGTCTCTCTTCATTCTTTTTATTXXXXXXXXXXAGTAAATTGCGGAGGCATAGACATATAATCAATAGCATTCCATTTATTCAAGATTTCTTTAAATAGAATATCAGCTCATT

GTTTTGTTTGTAATCACTATTTTTTGTTTTAATTTATTCCATTGTATTTTGAGCTTTAGTCTCTCTTCATTCTTTTTATTXXXXXXXXXXAGTAAATTGCGGAGGCATAGACATATAATCAATAGCATTCCATTTATTCAAGATTTCTTTAAATAGAATATCAGCTCATC

>Marker149931

TACTATCAATTATCTGATAGAACACTGATATAGTGTTTCTATTTATTGATATCTTCAATAGAATTACAATATAAAAGCAGXXXXXXXXXXTCATCTACTAACAATATTAAAACTATTAAGGCTATTCTCCCTGGCACGTGCGGTTAACCTTGCTTCTTCTTCCTTCTACC

TACGATCAATTATCTGATAGAACACTGATATAGTGTTTCTATTTATTGATATCTTCAATAGAATTACAATATAAAAGCAGXXXXXXXXXXTCATCTACTAACAATATTAAAACTATTAAGGCTATTCTCCCTGGCACGTGCGGTTAACCTTGCTTCTTCTTCCTTCTACC

>Marker150270

AAGTCCACTAATTATAACAATAAGAATAAGAAAATGAATAAAAAAAAACTTGCAAATTCATCAATGACTGTTATCAAATTXXXXXXXXXXTGCAGAAATGTGAAACCTATTGGCAAAAGGGGAAAATAATCTATTTGCATTGACCTGAATGTTTTTAAGCGCTCTGAGAA

AAGTCCACTAATTCTAACAATAAGAATAAGAAAATGAATAAAAAAAAACTTGCAAATTCATCAATGACTGTTATCAAATTXXXXXXXXXXTGTAGAAATGTGAAACCTATTGGCAAAAGGGGAAAATAATCTATTTGCATTGACCTGAATGTTTTTAAGCGCTCTGAGAA

>Marker150926

ATAAATCATGTTTACTTAACATTATCCAATCCATAATTAAATGGATGATGTGCAGGAGACAACGTGTTTGTGTTCGAGAGXXXXXXXXXXGCTACTAGACGCAACATGACACGCAACTTAAATTATATTGAATATAATCAATTAATTTTCAAAACTTTCAAATATGTTAG

ATAAATCATGTTTACTTAACATTATCCAATCCATAATTAAATGGATGATGTGCAGGAGACAACGTGTTTGTGTTCGAGAGXXXXXXXXXXGCTACTAGACGCAACATGACACGCAACTTAAATTATATTGAATATAATCAACTAATTTTCAAAACTTTCAAATATGTTAG

>Marker151184

AAAACCAAACGAAAATCATCATCCTTAATTTTAATCCATAGTTTTATGGGTTCTTTTTTATTTCTTCCAACATTTTAAAAXXXXXXXXXXAATGGAGAAGGTCGACCTCCGCCCTATTTTTTCACCACCCAAATGGTTCATAGTATGCTCTAATCATTGAATATAGAAAG

AAAACCAAACGAAAATCATCATCCTTAATTTTAATCCATAGTTTTATGGGTTCTTTTTTATTTCTTCCAACATTTTAAAAXXXXXXXXXXAATGGAGAAGGTCGACCTCCGCCCTATTTTTCCACCACCCAAATGGTTCATAGTATGCTCTAATCATTGAATATAGAAAG

>Marker151249

CCTCAGATCGGCTTTCAGTAATTTCCTTCTCATCTGCAGCACGACGCCTCTTTCCTCTTCGCCGGATGACCGAAAGTGGAXXXXXXXXXXCTACCTTTCACTTCTTCCAGTATCAATCCATCGCAATAACAACACAGCGCTGCAGATACCCGTTCATTCATTTGGTTTTG

CCTCAGATCGGCTTTCAGTAATTTCCTTCTCTTCTGCAGCACGACGCCTCTTTCCTCTTCGCCGGATGACCGAAAGTGGAXXXXXXXXXXCTACCTTTCACTTCTTCCAGTATCAATCCATCGCAATAACAACACAGCGCTGCAGATACCCGTTCATTCATTTGGTTTTG

>Marker151380

CTTGCAAACTTCTCCCAGCACACCTACACAACACACACAAGAAATTTACGCAGAGACTTCCCCAGAATCCCAAATATAGTXXXXXXXXXXGAAGAAAGATGAGAGTAGAAACAGGAAAACTGGGAGAGAGTCCAATGAAAGGAAAATACCTGTAGCTCGGTAGTGACAGG

CTTGCAAACTTCTCCCAGCACACCTACACAACACACACAAGAAATTTACGCATAGACTTCCCCAGAATCCCAAATATAGTXXXXXXXXXXGAAGAAAGATGAGAGTAGAAACAGGAAAACTGGGAGAGAGTCCAATGAAAGGAAAATACCTGTAGCTCGGTAGTGACAGG

>Marker151457

AATTTAGATCGAAGGTTTAGAGAATTTATCTCGCTTTCTTTAACTCTTTTCTCAGTTTTCTTTGATTGCGTGCAACAACAXXXXXXXXXXATCCAAACTATTAACGTTAATAATATTTACCAGAATTGATTAAACCTTTAATTTAGTTTTTCAAAATTATAATAATAATA

AATTTAGATCGAAGGTTTAGAGAATTTATCTCGCTTTCTTTAACTCTTTTCTCAGTTTTCTTTGATTGCGTGCAACAACAXXXXXXXXXXATCCAAACTATTAACGTTAATAATATTTACCAGAATTGATTAAACCTTTCATTTAGTTTTTCAAAATTATAATAATAATA

>Marker151530

TTTTGATGCTTGAGTTCTGCCTCGTGGTCATCCATTTATAAAGATTGAATATTTGGGGTCGCGCACACAACCCATTATCAXXXXXXXXXXTAATGCTTCGCATGGTCTTCTCAAGTTTTTCAGCCAATACTTTTGCCACCAGCTTGCAAGTCAAAGTAGTAAGACTTGTT

TTTTGATGCTTGAGTTCTGCCTCGTGGTCGTCCATTTATAAAGATTGAATATTTGGGGTCGCGCACACAACCCATTATCAXXXXXXXXXXTAATGCTTCGCATGGTCTTCTCAAGTTTTTCAGCCAATACTTTTGCCACCAGCTTGCAAGTCAAAGTAGTAAGACTTGTT

>Marker151821

CCAAGTTGATTCCTTATGATCCCTTCTCTTATTCTCAGAATTTCGATCAAGGCACCGCCGGAGAAGAACTTGACAATCTTXXXXXXXXXXTAGTTCTGAGTATAATTACTTCAAATTTACATGGAATAGATTAACTTTAGGATCATTTATGTCAAATTGTAAAAGATAAA

CCAAGTTGATTCCTTATGATCCCTTCTCTTATTCTCAGAATTTCGATCAAGGCACCGCCGGAGAAGAACTTGACAATCTTXXXXXXXXXXTAGTTCTTAGTATAATTACTTCAAATTTACATGGAATAGATTAACTTTAGGATCATTTATGTCAAATTGTAAAAGATAAA

>Marker151852

TCTCATGTAGTTGTAGAATGAGATCATTTGTAATATTATACTACTAATGGTCTCATAAGATCATATACAAATCTAGCAATXXXXXXXXXXTTCTTTATGTATTCCACTTTCAATGTTAGGGGACGGAAAAGGGTATTTTTAAAAAGGATCATTTTTCATTATTACATAAA

TCTCATGTAGTTGTAGAATGAGATCATTTGTAATATTATACTACTAATGGTCTCATAAGATCATATACAAATCTAGCAATXXXXXXXXXXTTCTTTATGTATTCCACGTTCAATGTTAGGGGACGGAAAGGGGTATTTTTAAAAAGGATCATTTTTCATTATTACATAAA

>Marker152047

ACTTGACCTAGAAGTTAGAGGATTAAATCTCTTATACCATTTATTGTTGAACTCAGAAACTGTTTTCTAAAAGATTGAACXXXXXXXXXXTAGCTAATATAATATGTTCTACACTACCAAAATTTGATTGTAATTTGAAATCAAATTTATGGTAAATCGTGTTCAACCAA

ACTTGACCTAGAAGTTAGAGGATTAAATCTCTTATACCATTTATTGTTGAACTCAGAGACTGTTTTCTAAAAGATTGAACXXXXXXXXXXTAGCTAATATAATATGTTCTACACTACCAAAATTTGATTGTAATTTGAAATCAAATTTATGGTAAATCGTGTTCAACCAA

>Marker152056

TGAACATAAAGGATTTCATTGTTGGGATCCCATCTCTAAACGATTACGTATATCTCACCATGTCACCTTTTAGGAAGATCXXXXXXXXXXAATCTCCTCCTCATCTTAAAGATTACCATTTTTTTCCACTATCATGTCTTTGGTTGATCCTTCCTCGTATAAGAAAGCCA

TGAACATAAAGGATTTCATTGTTGGGATCCCATCTCTAAACGATTACGTATATCTCACCATGTCACCTTTTAGGAAGATCXXXXXXXXXXAATCTCCTCCTCATCTTAAAGATTACCATTTTTTCCCACTATCATGTCTTTGGTTGATCCTTCCTCGTATAAGAAAGCCA

>Marker152062

CAAATACTTTGTTAAATTTGAATGATTTCCTTCCCTCCTTGCCATATTTCGATGGTGTCATAATGCTCATATTCCCTTCAXXXXXXXXXXTATTCTGAAGCAGCATAAGCCACACTATACATGCGCTTACCTAATTTAGGCGGGCAGTTAGAATGAGAATACATTTGAAG

CAAATACTTTGTTAAATTTGAATGATTTCCTTCCCTCCTTGCCATATTTCGATGGTGTCATAATGCTCATATTCCCTTCAXXXXXXXXXXTATTCTGAAGCAGCATAAGCCACACTATACATGCGCTTACCTAATTTAGGCGGGCAGTTAGACTGAGAATACATTTGAAG

>Marker152200

TTCTCCAAATCAAACTCAAGAATCTGTTAGATATTATATTAAATTTACCTTTACCTATTAGCTTAAGCTTTTGGGTTACAXXXXXXXXXXTTCTGCTTGAACCTTTGTGATGAGCTTGTTGATTGAAGTTGTGATACAATCTTACTTGAACAAGATTTATTCTATAGGTT

TTCTCCAAATCAAACTCAAGAATCTGTTAGATATTATATTAAATTTACCTTCACCTATTAGCTTAAGCTTTTGGGTTACAXXXXXXXXXXTTCTGCTTGAACTTTTGTGATGAGCTTGTTGATTGAAGTTGTGATACAATCTTACTTGAACAAGATTTATTCTATAGGTT

>Marker152443

AAGTATTCCTCTTTTTTCCCCTAATTTGTAGTATAAATTTTTAACCTCTAAATTGCATTATAAATCTTTTGAGAGTTTGTXXXXXXXXXXTGAGTTGATATTGAATTTTTTAGCTTAAAAGGAGGGGATCCAATCTCTCATCCTTCGTCCTACCTATCTCTAAGTGATAG

AAGTATTCCTCTTTTTCCCCCTAATTTGTAGTATAAATTTTTAACCTCTAAATTGCATTATAAATCTTTTGAGAGTTTGTXXXXXXXXXXTGAGTTGATATTGAATTTTTTAGCTTAAAAGGAGGGGATCCAATCTCTCGTCCTTCGTCCTACCTATCTCTAAGTGATAG

>Marker152499

TAATATTGATTTTTACTTGTTGGGTCTTTTACAAATTTTCTACCTCACAAATGAGTGATTGTGTTAGAACGTCGCTATAAXXXXXXXXXXCTACCTAGGAATTATCTTACGAGTTTCTTTGACTCCTAATGTTGTAAGGTCAAGTGGGTTGTCCCATGAGATCAGTTGAG

TAATATTGATTTTTACTTGTTGGGTCTTTTACAAATTTTCTACCTCACAAATGAGTGATTGTGTTAGAACGTCGCTATAAXXXXXXXXXXCTACCTAGAAACTATCTTACGAGTTTCTTTGACTCCTAATGTTGTAAGGTCAAGTGGGTTGTCCCATGAGATCAGTTGAG

>Marker152708

ATAGATTAAGTTACATTTAATACATTCTCAATCTACATTAATCTAAAAAAAATGTTGTGTATTTTTGTGTGGGTCATGAAXXXXXXXXXXGATGTCTTTTTACTGCTTTACTTGAGCAGTTAAATTTGTTATTAATTTTTTTTTAAATTTAGTTTTAATTTGAAGAAAAT

ATAGATTAAGTTACATTTAATACATTCTCAATCTACATTAATCTAAAAAAAATGTTGTGTATTTTTGTGTGGGTCATGAAXXXXXXXXXXGATGTCTTTTTACTGCTTTACTTGAGCAGTTAAATTTGTTATTAGTTTTTTTTTAAATTTAGTTTTAATTTGAAGAAAAT

>Marker152898

GAAGACAAGGAAGTTGCATGGGGATCGGTTGAGGAGGACGGCAGCGGCGACGGAGATTTCTTTGAATGTTTGTTGTGGGGXXXXXXXXXXTTGCCTCATTCTTATTATTATTTTTCCTTTTGTTGTTGAAGAAGTAAAGAGATTTGAGATTTGGGATATGGAAATTTAAA

GAAGACAAGGAAGTTGCATGGGGATCGGTCGAGGAGGACGGCAGCGGCGACGGAGATTTCTTTGAATGTTTGTTGTGGGGXXXXXXXXXXTTGCCTCATTCTTATTATTATTTTTCCTTTTGTTGTTGAAGAAGTAAAGAGATTTGAGATTTGGGATATGGAAATTTAAA

>Marker153326

CATCGGTTTGTGTCTGCATTTGTAGAATTCAAAAGATTAAAATATGTATCCAATTTTTTTACAAATAGATTAGCAAATTCXXXXXXXXXXAGATTCATACTTCTCATTATTGATACACTTTCACCATTACCAAGCAGTAATTTTGCAGCTTCGTCAAGTGAAATCAGATG

CATCGGTTTGTGTCTGCATTTGTAGAATTCAAAAGATTAAAATATGTATCCAATTTTTTTACAAATGGATTAGCAAATTCXXXXXXXXXXAGATTCATACTTCTCATTATTGATACACTTTCACCATTACCAAGCAGTAATTTTGCAGCTTCGTCAAGTGAAATCAGATG

>Marker153437

TTTATTCATGGGTTTACAAGTAGCTACAATTACCCATTTAACACAAGTAACAAATGATTTAACTAAGCCATAACAAAAGTXXXXXXXXXXAATAAAACTTGACTAAATTAAATTACTGGTGAGAGCTTTAATATGGTATCAGATTAACCAATCCCCTCTTAAGAGTTACA

TTTATTCATGGGTTTACAAGTAGCTACAATTACCCATTTAACACAAGTAACAAATGATTTAACTAAGCCATAACAAAAGTXXXXXXXXXXAACAAAACTTAACTAAATTAAATTACTGGTGAGAGCTTTAATATGGTATCAGATTAACCAATCCCCTCTTAAGAGTTACA

>Marker153531

TCGATCTCTAGGTCATTTGTAACGACCCAACTTTCAATATCTCTAATTAACTAAGGTTGTTACTTTAAAATTGATAAAAGXXXXXXXXXXATATGTTCTTTGCCTTACCTGAAAAACATGAAATAAGAAAGGATGAGTATAAAAATATTCAATAAGTAATCCCTTATCGA

TCGATCTCTAGGTCATTTGTAACGACCCAACTTTCAATATCTCTAATTAACTAAGGTTGTTACTTTAAAATTGATAAAAGXXXXXXXXXXATATGTTCTTTGCCTTACCTGAAAAACATGAAGTAAGAAAGGATGAGTATAAAAATATTCAATAAGTAATCCCTTATCGG

>Marker153679

GTCCATTTTCATTGAAAACAATCCTTAAGTTCAAGCTAGATAGCACGAGCTTAATCAACAAAATGCTAGAAGCAGAGGAAXXXXXXXXXXTTGTGAGTAATTCTACAACTAAAACAAGATTGAATGTATCACTATGATGAAGGAGATAAGGACGCTTCGATACTTCAGGT

GTCCATTTTCATTGAAAACAATCCTTAAGTTCAAGCTAGATAGCACAAGCTTAATCAACAAAATGCTAGAAGCAGAGGAAXXXXXXXXXXTTGTGAGTAATTCTACAACTAAAACAAGATTGAATGTATCACTATGATGAAGGAGATAAGGACGCTTCGATACTTCTGGT

>Marker153776

AGACGAATGGTCTGACCAAAATCGACATTGCATTTTCACCATCCAAGGAAAACTGCCGGCTAAATCACTGTCTTTCAAGCXXXXXXXXXXCACCCCCACCACACACAAACACACGAAATCTCTTCCAATAAGAAGAATCAAGGCAAAGGTAAGGCTGCAAAGTTAAAACA

AGACGAATGGTCTGACCAAAATCGACGTTGCATTTTCACCATCCAAGGAAAACTGCCGGCTAAATCACTGTCTTTCAAGCXXXXXXXXXXCACCCCCACCACACACAAACACACGAAATCTCTTCCAATAAGAAGAATCAAGGCAAAGGTAAGGCTGCAAAGTTAAAACA

>Marker153851

TTAACCCTTTTCTACATCCTCCAACTTTTCAATCGTTAATATGTTAGCAAACGTGTGGAATAAAGAAATATAGGTTATGCXXXXXXXXXXATACCAGGGGACCTTCAAATTATAGAGCAAACTTGAAAGGTGACTTTCTTCTATCCCCTGTATTATTTTGCTACAATTAT

TTAACCCTTTTCTACATCCTCCAACTTTTCAATCGTTAATATGTTAGCAAAGGTGTGGAATAAAGAAATATAGGTTATGCXXXXXXXXXXATACCAGGGGACCTTCAAATTATAGAGCAAACTTGAAAGGTGACTTTCTTCTATCCCCTGTATTATTTTGCTACAATTAT

>Marker153882

CTTTTGCAATCCATCTACGTTTTTACTGTTTACGTGTCCTTGTGTTATTTGTCGTTTAAATTTGTGATTAGTTCTTGATAXXXXXXXXXXGGAGCTCTATAAGTTTTATACGTGAATACTTTTAGATTTACCTTGTTTAGTTAGGCTTAGTCATTTGAATTCCGAAAGGA

CTTTTGCAATCCATCTACGTTTTTACTGTTCACGTGTCCTTGTGTTATTTGTCATTTAAATTTGTGATTAGTTCTTGATAXXXXXXXXXXGGAGCTCTATAAGTTTTATACGTGAATACTTTTAGATTTACCTTGTTTAGTTAGGTTTAGTCATTTGAATTCCGAAAGGA

>Marker154049

ATTCAAAGCAGTGTCGATATCTAAAGTGCAAAGGAAGTCAATCAGATCCTGGTTGGCTGCAGAAAGGCTAATTTTTTGCAXXXXXXXXXXGACCTACTGATCTCATAGGAGAAAACTCTGTTGTTCGAACCCAAAGATAGTCATAATCTTCTATGCAAAGACGGTAGCGA

ATTCAAAGCAGTATCGATATCTAAAGTGCAAAGGAAGTCAATCAGATCCTGGTTGGCTGCAGAAAGGCTAATTTTTTGCAXXXXXXXXXXGACCTACTGATCTCATAGGAGAAAACTCTGTTGTTCGAACCCAAAGATAGTCATAATCTTCTATGCAAAGACGGTAGCGA

>Marker154229

AAAAGGGAAAAGGGAAAAGGAGAAAGGAAAAAAATAATGATGATGATTGAAAAGGTGATGTTTGAAAGTGAGCATAGGATXXXXXXXXXXGAAACGAAAGATGATTTGTTTTTCGTAATCATTTTAAGAAATAAATAACCGAGAAAAAGGCATGTTAAATCCCATCATGA

AAAAGGGAAAAGGGAAAAGGAGAAAGGAAAAAAATAATGATGATGATTGAAAAGGTGATGTTTGAAAGTGAGCATAGGATXXXXXXXXXXGGAAACGAAGATGATTTGTTTTTCGTAATCATTTTAAGAAATAAATAACCGAGAAAAAGGCATGTTAAATCCCATCATGA

>Marker154408

TCCTTCAATGCCTCATAATCATGCCACTCTCCATTTATGGAAAACATGGACTTCTAACTATCGTAAACATCACTCGATTCXXXXXXXXXXAAAAACATCAACAGAGAACACTATGCAGGTAGACATAAACCCAGAACTAACATCTGAGAGGAACATTAACAGATAAATTT

TCCTTCAATGCCTCATAATCATGCCACTCTCCATTTATGGAAAACATGGACTTCTAAGTATCGTAAACATCACTCGATTCXXXXXXXXXXAAAAACATCAACAGAGAACACTATGCAGGTAGACATAGACCCAGAACTAACATCTGAGAGGAACATTAACAGATAAATTT

>Marker154641

TCAGTTCGTGTGATGTGTGGTATTGAACATTTTGTTTATGACATTGTGACTGATTTGTTTGAGAAGATCCGTTCAAGTTAXXXXXXXXXXTGTGTATGAAGATCTTGCTGTTACTTATCAATCTTATGAGCAATTGGTAGTGGAACTCACTAGTGGCATAGTTCACTCTT

TCAGTCTGTGTGATGTGTGGTATTGAACATTTTGTTTATGACATTGTGACTGATTTGTTTGAGAAGATCCGTGCAAGTTAXXXXXXXXXXTGTGTATGAAGATCTTGCTGTTACTTATCAATCTTTTGAGTAATTGGTAGTGGAACTCACTAGTGGCATAGTTCACTCTT

>Marker154912

TCTCTGAAATTGTCTATCAATGAAGTTCTCTTTAATTCCTTAAAGTATGAATTCAGCGTTTAACTCTATGTTGTAAGAAGXXXXXXXXXXGTGTAACTTTTAATTTATGTTCAGGTATTACAACTCTTCCCCTCTTGGAGATTTCATCCTTGAAATTTAGACAAAAGACA

TCTCTGAAATTGTCTATCAATGAAGTTCTCTTTAATTCCTTAAAGTATGAATTCAGCGTTTAACTCTATGTTGTAAGAAGXXXXXXXXXXGTGTAACTTTTAATTTATGTTCAGGTCTTACAACTCTTCCCCTCTTGGAGATTTCATCCTTGAAATTTAGACAAAAGACA

>Marker154945

TGAAGTGGCGACTTAAATGTGGGTGGCAACATCATTTATTTAAGTTTTGAGTTATCCAATGGGATTGTGCATGGTGACGTXXXXXXXXXXCTTTTCTTCTTTGCTGCATGTGTATTCACGCACCAACCAGTTCTCATGTGATGTGGCTTTTGCTTTGGACCCAACCCAAG

TGAAGTGGCGACTTAAATGTGGGTGGCAACATCATTTATTTAAGTTTTGAGTTATCCAATGGGATTGTGCATGGTGACGTXXXXXXXXXXCTTTTTTTCTTTGCTGCATGTGTATTCACGCACCAACCAGTTCTCATGTGATGTGGCTTTTGCTTTGGACCCAACCCAAG

>Marker155009

TTCATTTTAAACGATCGTGTAGATCATAATACACGATCTTGAGCCTTAATTGCAATGAGATGCCCTAGGATGAAAGAATTXXXXXXXXXXCATTACCCTGAGATGCCTCGGGATGAAAGACTTCTGCCTTTAGTCATTCAATATTAAACCATAATTAATTGCATCGACAT

TTCATTTTAAACGATCGTGTAGATCATAATACACGATCTTGAGCCTTAATTGCAATGAGATGCCCTAGGATGAAAGAATTXXXXXXXXXXCATTACCCTGAGATGCCTCGGGATGAAAGACTTCTGCCTTTAGTTATTCAATATTAAACCATAATTAATTGCATCGACAT

>Marker155854

AATGAAATTCAGTTTTTGCATCAGTGAAAAACCGTCTACATTCCTGACTCTCAAGGCAAAGTCAGTCTCAAATGGTTGGAXXXXXXXXXXCATTTAATCAACACGCCTGATGAAGCAGATGTTTGTTTAACGGGAAAGGAAAATGTAAATCCAAGTTCCTTTCTCCTAGG

AATGAAATTCAGTTTTTGCATCAGTGAAAAACCGTCTACATTCCTGACTCTCAAGGCAAAGCCAGTCTCAAATGGTTGGAXXXXXXXXXXCATTTAATCAACACGCCTGATGAAGCAGATGTTTGTTTAACGGGAAAGGAAAATGTAAATCCAAGTTCCTTTCTCCTAGG

>Marker155900

AGAGTTTCTCACTTAACGTCTTCATTCAGTATTAAAAGTGATACAATATTACAGTTAATAACGTTATCACAACTATTTCAXXXXXXXXXXATATATCTAAGCACTAACCAATGACAGATACAAGTAGCAGCAACAAGTCACACTATTGAAAGAGAACTTACACGCTTTTG

AGAGTTTCTCACTTAACGTCTTCATTCAGTATTAAAAGTGATACAATATTACAGTTAATAACATTATCACAACTATTTCAXXXXXXXXXXATATATCTAAGCACTAACCAATGACAGATACAAGTAGCAGCAACAAGTTACACTATTGAAAGAGAACTTACACGCTTTTG

>Marker156172

TTGAACACCGCCTGGTATCATGAGTGTTTTAGAGAATTTTGCCTTAAAATTCTCACAGAAGCGACTCTTTCACATTCACAXXXXXXXXXXGGTTGCCTCTCTACATAACTTCATATTATAGGCTTTAATCTCATTCTATTTTTATAAATTTTCAATCAAATTCCTAGTTA

TTGAACACCGCCTGGTATCATGAGTGTTTTAGAGAATTTTGCCTTAAAATTCTCACAGAAGCGACTCTTTCACATTCACAXXXXXXXXXXGGTTGCCTCTCTACATAACTTCATATTATAGGCTTTAATCTCATTCTATTTTTATAAATTTTCAATCAAGTTCCTAGTTA

>Marker156427

AAGGGGTCCTCTTTTTTCCGTATGGTCGTTTTGTTGTATACTTCTTATGAGGATGGCAGAGGATCTTGATTTACTCTTAGXXXXXXXXXXTGAGAGATCTTTGGTGATGCTTCGTCCCTTATCAGATTCTCTGTTTCTCTTTAAGCTTGGGTGATTAGGTCTTTTGGTAA

AAGGGGTCCTCCTTTTTCCGTATGGTCGTTTTGTTGTATACTTCTTATGAGGATGGCAGAGGATCTTGATTTACTCTTAGXXXXXXXXXXTGAGAGATCTTTGGTGATGTTTCGTCCCTTATCAGATTCTCTGTTTCTCTTTAAGCTTGGGTGATTAGGTCTTTTGGTAA

>Marker156576

ACTTCTATATCTCTCCAATCTCATTGGACTTAACCCCCAATTCCATCTCCCCCTCTCTATTCTTCCATTTCAATCAATCAXXXXXXXXXXATACAACTTATCGAAATGTGTAATAACTTTCACATAATATAATTTGAAAAAAAAATAGAGGTGTAAATCGATGTGTTTAT

ACTTCTATATCTCTCCAATCTCATTGGACTTAACCCCCAATTCCATCTCCCCCTCTCTATTCTTCCATTTCAATCAATCAXXXXXXXXXXATACAACTTATCAAAATGTGTAATAACTTTCACATAATATAATTTGAAAAAAAAATAGAGGTGTAAATCGATGTGTTTAT

>Marker157153

TATAAAAGGTGAGTATGTGGCATTTCAACTTCTCTGGACCTTGAAAGTTTATATTATCCATTTATCTCAAATACTTCTATXXXXXXXXXXTTCTTGCTGTGAGGATAAATTGGTGTTTTATATTTCACAGTATACAGTGGAGGGATTGAACCTCAAATCTTAATGCTATA

TATAAAAGGTGAGTATGTGGCATTTCAACTTCTCTGGACCTTGAAAGTTTATATTATCCATTTATCTCAAATACTTCTATXXXXXXXXXXTTCTTGCTGTGAGGATAAATTGGTGTTTTATATTTCACAGTATACGGTGGAGGGATTGAACCTCAAATCTTAATGCTATA

>Marker157226

ACTATAGAACAGATCATGACATTTAATTCCAAGAAAACTTTATCTTTTTCGATTTCCAAAAATAAAAATAAGGTCACCTCXXXXXXXXXXAAAGTGCATTCCCGAAAAAAAAAAAACATGTTCTTGCAGCATAAACAAGGACACTTACCAAGCTATTTTGTTGTGAAGGT

ACTATAGAACAGATCATGACATTTAATTCCAAGAAAACTTTATCTTTTTCGATTTCCAAAAATAAAAATAAGGTCACCTCXXXXXXXXXXAAAGTGCATTCCCGAAAAAAAAAATACATGTTCTTGCAGCATAAACAAGGACACTTACCAAGCTATTTTGTTGTGAAGGT

>Marker157230

ACTAGCCTCGTAATTCAGCTGATGCATATACAACTCCGAACTGTAATATCACAGGTTGTCAATGCTCTACTTACACAGATXXXXXXXXXXAGTTGGCATACTCATCTGAGTGCAACTAGTCACTAGAAAAAAAAGGAAAAGATAAGAAAACTGAAATCGTTTCAACTTTT

ACTAGCCTCGTAATTCAGCTGATGCATATACAACTCCGAACTGTAATATCACAGGTTGTCAATGCGCTACTTACACAGATXXXXXXXXXXAGTTGGCATACTCATCTGAGTGCAACTAGTCACTAGAAAAAAAAGGAAAAGGTAAGAAAACTGAAATCGTTTCAACTTTT

>Marker157243

TAGGGTTTGACATAAATTTGTGTGTTCTTTGTCTCTACTTTAAATGAGTTGTTTATTGAAAGTTTTTTGGGTTGGAGATTXXXXXXXXXXCACTGATGCAGTTACAATTAACTGTGAAGCGAGCTTCAAAAACTGTTTATCAGGTGAAACATTATCTTAGGATGCTTGAT

TAGGGTTTGACATAAATTTGTGTGTTCTTTGTCTCTACTTTCAATGAGTTGTTTATTGAAAGTTTTTTGGGTTGGAGATTXXXXXXXXXXCACTGATGCAGTTACAATTAACTGTGAAGCGAGCTTCAAAAACTGTTTATCAGGTGAAACATTATCTTAGGATGCTTGAT

>Marker157338

TAATTGATGACAGATACAAGAAGCAGCAAAAAATCACACCATTAAGAGAGAATTTATGTTACACCCCAATCCGTAAGATAXXXXXXXXXXCTTGACCAAATAAATGATGCTCAACTATTTCCAAAATGAGAGTCATCTCGTCGCAAATCCTTCAACAGTCTTCTCTAATT

TAATTGATGACAGATACAAGAAGCAGCAAAAAATCACACCATTAAGAGAGAATTTATGTTACACCCCAATCCGTAAGATAXXXXXXXXXXCTTGACCAAATAAATGATGCTCAACTATTTCAAAAATGAGAGTCATCTCGTTGCAAATCCTTCAACCGTCTTCTCTAATT

>Marker157817

TTTTCTTTTTTGGCTCCCTTTTATTGTGGAATTGTATTTTCTGGATGTTTGTGTAATCTTCATTTTTTCTCCATGAAAGTXXXXXXXXXXGGTGAGGGGTCAATATAATATGTTTTTACGGCAGAGTAGGATTTTTAGTGTATGTGTTTGAATTAAATACCTTTTAGATT

TTTTCTTTTTTGGCTCCCTTTTATTGTGGAATTGTATTTTCTGGATGTTTGTGTAATCTTCATTTTTTCTCCATGAAAGTXXXXXXXXXXGGTGAGGGGTCAATATAATATGTTTTTACGGCAGAGTAGGATTTTTAGTGTATGTGTTTAAATTAAATACCTTTTAGATT

>Marker157941

TATAAGACCCATTGCAAGTCCCCACGAAGTATGTTCTTCAAGATGACAATGTATAAACCAAACTCCAGGATTATCCGCCTXXXXXXXXXXTCTGTGTTCATGTTTCTGGATAATGGGTTTACGCCAGTGTAATAAAAAAACCTGATGGGTTTTTCTGGAAAATCAGTGGA

TATAAGACCCATTGCAAGTCCCCACGAAGTATGTTCTTCAAGATGACAATGTATAAACCAAACTCCAGGATTATCCGCCTXXXXXXXXXXTCTGTGTTCATGTTTCTGGATAATGGGTTTACGCCAGTGTAATCAAAAACCCTGATGGGTTTTTCTGGAAAATCAGTGGA

>Marker158223

TCTCCACTGCTTTGCCCCACTCCATCATTTGGATTCTTTCTTGCTCCCACCATTTCAAGAACTAACATTCCATAGCTATAXXXXXXXXXXGCAAGTATTCTAATCCTCGTGCCACACCGATAACGATGCTATGAAGTGTGTTCCAATCCAGCTCAATGTTGTTTCTGTGT

TCTCCACTGCTTTGCCCCACTCCATCATTTGGATTCTTTCTTGCTCCCACCATTTCAAGAACTAACATTCCATAGCTATAXXXXXXXXXXGCAAGTATTCTAATCCTCGTGCCACACCGATAACGATGCTATAAAGTGTGTTCCAATCCAGCTCAATGTTGTTTCTGTGT

>Marker158332

CATTATATAATTTCTCACTACTGATGCAGTGATAGTGGATTATGTATGGTCTGATGACAATTTGGTGGATTCTTTGACGAXXXXXXXXXXAGTGATTAACACAACATTCTAAAGCATATGAAAAGGTTGAACTTTGTTCTTAATGAATTCTATACTTGATATCAAGTAGG

CATTATATAATTTCTCACTACTGGTGCAGTGATAGTGGATTATGTATGGTCTGATGACAATTTGGTGGATTCTTTGACGAXXXXXXXXXXAGTGATTAACACAACATTCTAAAGCATATGAAAAGGTTGAACTTTGTTCTTAATGAATTCTATACTTGATATCAAGTAGG

>Marker158460

TCGTTGCCCCAAAGGAAGAAACAAGTCTGCAGCGCCACAGTTTATTCAAGACAAAGTGCACAATAAATAGGTGGGTATGTXXXXXXXXXXTCTTTCCATTGGAAACGGGTATAAAGATCAGATTGCTTGTGATGTAATTGACATGGATGTGTGTCACCTTCTCTTAGGAA

TCGTTGCCCCAAAGGAAGAAACAAGTCTGCAGCGCCACAGTTTATTCAAGACAAGGTGCACAATAAATAGGTGGGTATGTXXXXXXXXXXTCTTTCCATTGGAAACGGGTATAAAGATCAGATTGCTTGTGATGTAATTGACATGGATGTGTGTCACCTCCTCTTAGGAA

>Marker158542

AGCCCCGCGACGCCAATGTTGCAGCAATAGCAGATGTTGCTAGTTTCAGAACAGAAATTGTATATACAATAAGGTTCTTAXXXXXXXXXXCATTGTTCTATTTTTTGGACATTAATCTGTGTTAAATTAATATTCAAACCGTGGGGAAGGAGTTAGTTGATGATTTTTCA

AGCCCCGCGACGCCAATGTTGCAGCAATAGCAGATGTTGCTAGCTTCAGAACAGAAATTGTATATACAATAAGGTTCTTAXXXXXXXXXXCATTGTTCTATTTTTTGGACATTAATCTGTGTTAAATTAATATTCAAACCGTGGGGAAGGAGTTAGTTGATGATTTTTCA

>Marker158619

TTCAATGCTTTGATTTATCCCTGATTAAGGTAATAAAACTTAGCAAGCAAATAGTTAAATTAACTTGCTTGGTATTGGTGXXXXXXXXXXTAGACTTGAGCAAATAAACTTGATCTAGAGTTCAATGATCAATTGACTAATTGGAGAAGCATAACATCAAATCCAAGCTA

TTCAATGCTTTGATTTATCCCTGATTAAGGTAATAAAACTTAGCAAGCAAATAGTTAAATTAACTTGCTTGGTATTGGTGXXXXXXXXXXTAGACTTGAGGAAATAAACTTGATCTAGAGTTCAATGATCAATTGACTAATTGGAGAAGCATAACATCAAATCCAAGCTA

>Marker158627

ATAAGTCTTGGCAGCTGTTTGTCAGCTCATGTAGTCTTAGAATTTTTACGAACCAGATGGTTTTTGGTTGGAATCAAGATXXXXXXXXXXGGTCTCATTTTGTTTGTCTGTGTATTTAGTTTCCAGTTTCTTTGTCCCTTAGAAATTCAAACTATTTGTATATTTCTCTT

ATAAGTCTTGGCAGCAGTTTGTCAGCTCATGTAGGCTTAGAATTTTTACGAACCAGATGGTTTTTGGTTGGAATCAAGATXXXXXXXXXXGGTCTCATTTTGTTTGTCTGTGTATTTAGTTTCCAGTTTCTTTGTCCCTTAGAAATTCAAACTATTTGTATATTTCTCTT

>Marker159019

TCCTTGGCAAAGACAAAGGGGACAATTCCTTGTGGGAGAGCTGCCTGTTTTATTATATATTTTTTTCCAAGAAGAAAAAGXXXXXXXXXXCAACTTCTTTTATTCCCACTTTTTCACAATTATATTACCAAAAACGGTTAAATTATAAATTTGGTTTCATTTTTACAAAA

TCCTTGGCAAAGACAAAGGGGACAATTCCTTGTGGGAGAGCTGCCTGTTTTATTATATATTTTTTTCCAAGAAGAAAAAAXXXXXXXXXXCAACTTCTTTTATTCCCACTTTTTCACAATTATATTACCAAAAACGGTTAAATTATAAATTTGGTTTCATTTTTACAAAA

>Marker159112

CAAGCTTTATGTAAATACTACTAAGGTAATCGTCTGACTTGAATATTTGGGTCAGTCAAAAACTTCAAATAGCAATGGCAXXXXXXXXXXATCGACGAGCCAGAGGAGAAGGGCTACTTCGAACCAGTTAAATGGAGCATATTCTCTCATGTCTGCACTGCTCCAGTAGA

CAAGCTTTATGTAAATACTACTAAGGTAATCGTCTGACTTGAATATTTGGGTCAGTCAAAGACTTCAAATAGCAATGGCAXXXXXXXXXXATCGACGAGCCAGAGGAGAAGGGCTACTTCGAACCAGTTAAATGGAGCATATTCTCTCATGTCTGCACTGCTCCAGTAGA

>Marker159261

CAGGACATTTCAGATATGATAATTTGGCATTTGCAAGTGTATTCAGATATAGACACTTGAGTGAATATCTTAACTTGATAXXXXXXXXXXTTGAAACCGAAGTCCACAAAAAGACATGAAAACAAGCAAAAGACCAAATCTCACTAGGGTCTTTATCCACTCCTCTAAAA

CAGGACATTTCAGATATGATAATTTGGCATTTGCAAGTGTATTCAGATATAGACACTTGAGTGAATATCTTAACTTGATAXXXXXXXXXXTTGAAACCGAAGTCCACAAAAAGACATGAAAGCAAGCAAAAGACCAAATCTCACTAGGGTCTTTATCCACTCCTCTAAAA

>Marker159295

GAGACTCGAAGGTATTGTTTGATTCGGTGTAACTAATTGCTTTGAAAATTTGCGATCATTAAGATCTTAAAATTTTGATCXXXXXXXXXXACAGTAGATAGAATCAGGAATTAGGCTTCCTTCCCTCTCCTTCTTCCTCTTTCCCGCAATTGAGACCATTCTCGCTAGTG

GAGGCTCGAAGGTATTGTTTGATTCAGTGTAACTAATTGCTTTAAAAATTTGCGATCATTAAGATCTTAAAATTTTGATCXXXXXXXXXXACAATAGATAGAATCAGGAATTAGGCTTCCTTCCCTCTCCTTCTTCCTCTTTCCCGCGATTGAGACCATTCTCGCTAGTG

>Marker159539

CAATGGTATTCAATTATATATCAATTCTTAATAAGTTTAGGGTTTGGTAGAACAAAGATAGTGTGACGTTAAGGGATGAAXXXXXXXXXXGGTTGACTAGTTGAGCTAGTAAATTTATACCAATTGAGAAACAAATGAGTTGAGGCTGGACTAAGACCTTGTAACTTCAA

CAATGGTATTCAATTATATATCAATTCTTAATAAGTTTAGGGTTTGGTAGAACAAAGATAGTGTGACGTTAAGGGATGAAXXXXXXXXXXGGTTGACTAGTTGAGCGAGTAAATTTATACCAATTGAGAAACAAATGAGTTGAGGCTGGACTAAGACCTTGTAACTTCAA

>Marker159592

CTCAGTTTTAGGTAGATTGGATCATATCAATGTGATCTGCTGCATATTGTCTTACCATTTATAACCCCGAACAAATAATAXXXXXXXXXXTTGCAAAATTTTTAAAATATTGTTATATACTTAATTATTTTGAATCTAATTGTTAAATTTGCAACAATTCAATTATTATA

CTCAGTTTTAGGTAGATTGGATCATATCAATGTGCTCTGCTGCATATTGTCTTACCATTTATAACCCCGAACAAATAATAXXXXXXXXXXTTGCAAAATTTTTAAAATATTGTTATATACTTAATTATTTTGAATCTAATTGTTAAATTTGCAACAATTCAATTATTATA

>Marker159987

TTCTTATGAACTAATGATATTTTTTATCTTTACTTTGATAAAAATCATATGAAATCATGAAATGAAAGCAAAGAAAAGCAXXXXXXXXXXCATTTTGCAAATAAAGCCTTCTCACTTTGTGCTTTGTTCTACTAGAAATGAATTATTTACATTTTTCTTGGTCAAGTTTG

TTCTTATGAACTAATGATATTTTTTATCTTTACTTTGATAAAAATCATAAGAAATCATGAAATGAAAGCAAAGAAAAGCAXXXXXXXXXXCATTTTGCAAATAAAGCCTTCTCACTTTGTGCTTTGTTCTACTAGAAATGAATTATTTACATTTTTCTTGGTCAAGTTTG

>Marker160230

AAAGGATCATTCAGTATTTGCAGGTTTTTCTTTTAGTGTTCAATAATATTGCTATTGCTACCTTAGAGTTGGTTGACTCCXXXXXXXXXXGAGGAAAGATAGTGTTCATAACTAAGTTACTCATAGCATTTAGTTTTGTTTTGATATCTACCATCAACAAGTAGTTAATT

AAAGGATCATTCAGTATTTGCAGGTTTTTCTTTTAGTGTTCAATAATATTGCTATTGCTACCTTAGAGTTGGTTGACTCCXXXXXXXXXXGAGGAAAGATAGTGTTCATAACTAAGTTACTTATAGCATTTAGTTTTGTTTTGATATCTACCATCAACAAGTAGTTAATT

>Marker160517

CATATTTCCATTCTTCACATCGCGTTGTTTGATTCTTTTGCCTTCTGCCCCTTTCTTTATGTTATCTCAAAATGCCTACTXXXXXXXXXXCCAAAGAATAAAGGAGTGATTGCAATAATGGAAATGCTTCAAATATGAAGGTGGGCAATGTTGTTTTAATAATCTTCTTA

CATATTTCCATTCTTCACATCGCGTTGTCTGATTCTTTTGCCTTCTGCCCCTTTCTTTATGTTATCTCAAAATGCCTACTXXXXXXXXXXCCAAAGAATAAAGGAGTGATTGCAATAATGGAAATGCTTCAAATATGAAGGTGGGCAGTGTTGTTTTAATAATCTTCTTA

>Marker160588

TCCTTGTAGATGAAAGATTTGTAGTTTGGTGGTTGGTCAATGTGGGTAGTGAAATGTGGCAATGGCTGCACCAGTTTGTCXXXXXXXXXXGACATCTCCAACGTTGATAACAAGAGTGTTTTGGTTGGGAATAATTGGGATCCATTGATGATCTTCTGTTTTAACCTCAA

TCCTTGTAGATGAAAGATTTGTAGTTTGGTGGTTGGTCAATGTGGGTAGTGAAATGTGGCAATGGCTGCACCAGTTTGTCXXXXXXXXXXGACATCTCCAACGTTGATAACAAGAGTATTTTGGTTGGGAATAATTGGGATCCATTGATGATCTTCTGTTTTAACCTCAA

>Marker160849

TATATATAGAGATCATTTCCTTCATTTATACGCAAGAGAAAAGAATAAAATCAATGGAAAAGCAAATTGAAGTTTAGAAAXXXXXXXXXXAGGCTAAATTAATAATAGAGAGAAATAAAGTTAAGGTTGTATTATTAATGGTATATGAAAATTTCAACTTTTCACAGGGT

TATATATAGAGATCATTTCCTTCATTTATACGCAAGAGAAAAGAATAAAATCAATGGAAAAGCAAATTGAAGTTTAGAAAXXXXXXXXXXAGGCTAAATTAATAATAGACAGAAATAAAGTTAAGGTTGTATTATTAATGGTATATGAAAATTTCAACTTTTCACAGGGT

>Marker160882

ACTGAGTTGTTCGAGCACCTGCAAATAAATAACCGATATAAGAAATCAATCGGGATGCAAATAGGGTGAAGCTATAAAGGXXXXXXXXXXTGAATCAATGAGCTAGTCCTCTACAAATATGACACAGCAATCATAAGAGATGAAGTAAATATGAAGAGATAGTAGAAACG

ACTGAGTTGTTCGAGCACCTGCAAATAAATAACCGATACAAGAAATCAATCGGGATGCAAATAGGGTGAAGCTATAAAGGXXXXXXXXXXTGAATCAATGAGCTAGTCCTCTACAAATATGACACAGCAATCATAAGAGATGAAGTAAATATGAAGAGATAGTAGAAACG

>Marker161356

AATCCCGACATAGTCTTTTTTGGTGTTGAAAAAAGTTACAATAATTACTTAAAAGTTGAACCTTTGCCAACGTGATCAACXXXXXXXXXXGTCGCAAGAACCATTTTTTCTTGTAGTGAATGTATCTTTTTAGGATTTAGATTGAAACCTATATTATGTATTAAAAAAAT

AATCCCGACATAGTCATTTTTGGTGTCGAAAAAAGTTACAATAATTACTTAAAAGTTGAACCTTTGCCAACGTGATCAATXXXXXXXXXXGTCGCAAGAACCATTTTTTCTTGTAGTGAATGTATGTTTTTAGGATTTAGATTGAAACCTATGTTATGTATTAAAAAAAT

>Marker161519

AAGGCAATATTGTTAAAATAATGTGTTAAAAGAATGTTTGTGTAATATCAACTTATTGTTTTTATTATTAAATAATATATXXXXXXXXXXAAAACATTTTAATATAATAGCAAAGCTAATTCTGTTGATCGTATTTCCATGAATTAGTGGCATGAAACTATAGATGATGA

AAGGCAATATTGTTAAAATAATGTGTTAAAAGAATGTTTGTGTAATATCAACTTATTGTTTTTATTATTAAATAATATATXXXXXXXXXXGAAACATTTTAATATAATAGCAAAGCTAATTCTGTTGATCGTATTTCCATGAATTAGTGGCATGAAACTATAGATGATGA

>Marker161538

CAATGATAGACAACTAATTGGGTTCCACTTTGCTATTTGGGCTTTCTCAGCCCAGCCCAATTCGTGCGGCTTAATGCCTAXXXXXXXXXXATTGCTTCCACTCTCAAATGCATCCATCATTTGTCTCCACCCGAATCTATCTCGCCTGATTTCACCAAGGAAGCAGGTAA

CAATGATAGACAACTAATTGGGTTCCACTTTGCTATTTGGGCTTTCTCAGCCCAGCCCAATTCGTGCGGCTTAATGCGTAXXXXXXXXXXATTGCTTCCACTCTCAAATGCATCCATCATTTGTCTCCACCCGAATCTATCTCGCCTGATTTCACCAAGGAAGCAGGTAA

>Marker161618

CAGTATCCTTGCCTAGAAACTGCTTAAATTATTTTTTTATTTTCTCACTTGTCTGGTTAAGTTTGCATCAATATTTTATTXXXXXXXXXXCGAGTTCATGATTATGTATTATATTCTCAGATCTCAACCGTTTCTGGAGAGCTGCGGTATATTGATGCAGTAAGATTGTT

CAGTATCCTTGCCTAGAAACTGTTTAAATTATTTTTTTATTTTCTCACTTCTCTGGTTAAGTTTGCATCAATATTTTATTXXXXXXXXXXCGAGTTCATGATTATGTATTATATTCTCAGATCTCAACCGTTTCTGGAGAGCTGCGGTATATTGATGCAGTAAGATTGTT

>Marker161894

TTCCATTTCGAAAGTTCTTTGGATGGCTAACCTTCGATCAACAGCACTTCCAAGTAAGAATGCTATGCTACTTCTTAGTTXXXXXXXXXXGGGGGATGAGATACCAATCAGCTTCAACATGGACAGTAAAGTAGACGAGGAAACTATAAAATTAGTAAGCTCCTTTGAGT

TTCCATTTCGAAAGTTCTTTGGATGGCTAACCTTCGATCAACAGCACTTCCAAGTAAGAATGCTATGCTACTTCTTAGTTXXXXXXXXXXGGGGGATGAGATACCAATCAGCTTCAACATGGACAGTAAAGTAGACGAGGAAACTATAAAATTAGTAAGTTCCTTTGAGT

>Marker161910

AATCGATCATCATCTTAGTTTAAAGAGCTTAATGCTCGGACTTTCTTACTTGTATGGACAAAAATGTGTAAAAAAGTAAAXXXXXXXXXXTCATGACAGGCTGATCTTGACGGTGATGGAACATTGAATTATTCAGAGTTCGTGGCTGTTTCTGTCCATCTTAAGAAGAT

AATCGATCATCATCTTAGTTTAAAGAGCTTAATGCTCCGACTTTCTTACTTGTATGGACAAAAATGTCTAAAAAAGTAAAXXXXXXXXXXTCATGACAGGCTGATCTTGACGGTGATGGAACATTGAATTATTCAGAGTTCGTGGCTGTTTCTGTCCATCTTAAGAAGAT

>Marker162015

ATTTGTGATGTCTCAGCAAATGGATGTCAATAAGAGCACAACAGAGAAAGATACATGGAAATAGTTTAGAAAAATCAGAAXXXXXXXXXXCATCGATACATACTGAGATATGACAAGGAGAACAGGTAATACAAGGTATGCTGCAGTGTCAGACCAGCCCAATGGTGGAT

ATTTGTGATGTCTCAGCAAATGGATGTCAATAAAAGCACAACAGAGAAAGATACATGGAAATAATTTAGAAAAATCAGAAXXXXXXXXXXCATCGATACATACTGAGATATGACAAGGAGAACAGGTAATACAAGGTATGCTGCAGTGTCAGACCAGCCCAATGGTGGAT

>Marker162183

ATCCTTGCTTATCTAGAGACTTGCAAAACAATTTAAACACAAAGCAGACCAGGCAGATATCGGAGATTTCCGATTCTCATXXXXXXXXXXCAAGAGAAAAATTGTAGACTTGGAAAGTCAAAAAGTTAGCATTCAACACTTAACTGGCTCGAATAAAACCGAATATAGTA

ATCCTTGCTTATCTAGAGACTTGCAAAACAATTTAAACACAAAGCAGACCAGGCAGATATCGGAGATTTCCGATTCTCATXXXXXXXXXXCAAGAGAAAAATTGTAGACTTAGAAAGTCAAAAAGTTAGCATTCAACACTTAACTGGCTCGAATAAAACCGAATATAGTA

>Marker162367

TGGATGTCAGGAAAAGTATAAAAAGACATTTGTAATGTCGCATAGTAGTGTCAGAGAATTAATGGTGTCCATATTGGCACXXXXXXXXXXAAAACTTTAAAATAAAACTCCATACAACCCGCATCTAATACTACTAATAGAACTAATGAATTGACCAAAGAGATACTATT

TGGATGTCAGGAAAAGTATAAAAAGACATTTGTAATGTCGCATAGTAGTGTTAGAGAATTAATGGTGTTCAGGTTGGCACXXXXXXXXXXAAAACTTTAAAATAAAACTCCATACAACCCGCATCTAATACTACTAATAGAACTAATGAATTGACCAAAGAGATACTATT

>Marker162620

AAGCTTCCTCCTAACCCTGCCGTCAACCAATTCTTTAACAACAAAACCTCAGCCCCTTCCCCACCCTTCACCGATTTGATXXXXXXXXXXCTAAAATCCGCCCAACAACAGTGGTGTCTTCCCGTGCTTTGCTGTCTAAGCAAGTCTACAAGCGTCCTGATTTTCTTATT

AAGCTTCCTCCTAACCCTGCCGTCAACCAATTCTTTAACAACAAAACCTCAGCCCCTTCCCCACCCTTCACCGATTTGATXXXXXXXXXXCTAAAATCCGCCCAACAACAGTGGTGTCTTCCCGCGCTTTGCTGTCTAAGCAAGTCTACAAGCGTCCTGATTTTCTTATT

>Marker162791

AATAGATATATACTAAAAATAAAAATTAAGAGACCTAAAGATTAGGAGACACACCCAAACATCTCAACTAGGTTGCCACCXXXXXXXXXXTACCATCGAATACCCTTTGATTCTATTCGAACCAAATTTCTTGAAGCAGTGATTTGGACAAGTTAGTCCAAAGAAGTCAT

AATAGATATATACTAAAAATAAAAATTAAGAGACCTAAAGATTAGGAGACACACCCAAACATCTCAACTAGGTTGCCACCXXXXXXXXXXTACCATCGAATACCCTTTGATTCTATTCGAACCAAATTTCTTGAAGCAGTGATTTGGGCAAGTTAGTCCAAAGAAGTCAT

>Marker162855

GTATATAAGTGATGATGGACTTCAATTGTTTATAAACTCATGGTCTATCCGTGTTGTATCAATATAATTATTTTAAAATGXXXXXXXXXXTGTTTCACGTTTACCCATATTGTTATGGTAAGAAATAATGAACTTTATTTGCATATACTATTCTTTTATACTAATTTTGA

GTGTATAAGTGATGATGGACTTCAATTGTTTATAAACTTATGGTCTATCCGTGTCGTATCAATATAATTATTTTAAAATGXXXXXXXXXXTGTTTCACGTTTACCCATATTGTTATGGTAAGAAATAATGAACTTTATTTGCATATACTATTCTTTTTTACTAATTTTGA

>Marker163504

AACTCGAGTTATTTCATGTTAGAGATAACCACAGAAAGGCGTGGGCACTCTATCATGCCACTCTTCAACTTCCTATTGCAXXXXXXXXXXATGATTTATTTCACATGACGACTATCATGTAAAAAGAGACCTTGTAGAGAAACCATTGGCTTGCCCTTTTGCAAATTATT

AACTCGAGTTATTTCATGTTAGAGATAACCACAGAAAGGCGTGGGCACTCTATCATACCACTCTTCAACTTCCTATTGCAXXXXXXXXXXATGATTTATTTCACATGACGACTATCATGTAAAAAGAGACCTTGTAGAGAAACCATTGGCTTGCCCTTTTGCAAATTATT

>Marker163512

ACACATCTGCCTCCTCCACAGAACAATGGGGAATCAGTGGGCTTGTCTGTTTCTTCCCCAAGAATCAGACTCAGAGATGGXXXXXXXXXXGGATAGATTATTATGCTGCTTTAAATTCCATAATGATATCGTAACGTAAGCAAAAGGTGGAAAAATGTTGAGAGGTTACA

ACACAGCTGCCTCCTCCACAGAACAATGGGGAATCAGTGGGCTTGTCTGTTTCTTCCCCAAGAATCAGACTCAGAGATGGXXXXXXXXXXGGATAGATTATTATGCTGCTTTAAATTCCATAATGATATCGTAACGTAAGCAAAAGGTGGAAAAATGTTGAGAGGTTACA

>Marker163634

ACTTGCACAATTAGGAGTGTTTGTATGATCTCACTCGCTCAAGTGTGCTCCATTGGATACACAACATTATTATTATGTTTXXXXXXXXXXCTATGTATTAGATTTTTAGTATTTTGGTTTTAGAAACCTTATTTAAAGTTTATCTTTATTTCTTTTAAAAAAAATTATTA

ACTTGCACAATTAGGAGTGTTTGTATGATCTCACTCGCTCAAGTGTGCTCTATTGGATACACAACATTATTATTATGTTTXXXXXXXXXXCTATGTATTAGAATTTTAGTATTTTGGTTTTAGAAACCTTATTTAAAGTTTATCTTTATTTCTTTTAAAAAAAATTATTA

>Marker163753

TATGAGTGCAGACATGATGCCAAACCACAGCCCCTATTAAGGATTGATTACAATAGATCATCAGGAACAGAAGGACATTAXXXXXXXXXXAGTATATGTGGGACACCAGTAAAGTTATAATGTAGGGTAGGCATGTAAACCCAAGTCTAATATGAACACAAAATTCATTT

TATGAGTGCAGACATGATGCCAAACCACAGCCCCTATCAAGGATCGTTTACAATAGATCATCAGGAACAGAAGGACATTAXXXXXXXXXXAGTATATGTGGGACACCAGTAAAGTTATAATGTAGGGTAGGCATGTAAACCCAAGTCTAATATGAACACAAAATTCATTT

>Marker163814

TACTTTTATCTGTTTTACTTTGTGGCTTCAATGTGATGAGTTGTAATGTTACTTTCAATTACATGAATTTATGTGGGGAAXXXXXXXXXXCCCACCCCCACCTTCATTGTATGACTTCCACCAATCACCATAATAAGCTAGACTTAGGTGAATAGTAAAAAGAATTCAAA

TACTTTTATCTGTTTTACTTTGTGGCTTCAATGTGATGAGTTGTAATGTTACTTTCAATTACATGAATTTATGTGGGCAAXXXXXXXXXXCCCACCCCCACCTTCATTGTATGACTTCCACCAATCACCATAATAAGCTAGACTTAGGTGAATAGTAAAAAGAATTCAAA

>Marker163956

TTCATATGAACTATAAGGAGTGTTTGTGGTAAGGGAAACTCAGCCTTATGTTGTAGTAATCAAAGTCCTGAATAACTTATXXXXXXXXXXTTAGTTGGTTATTATAGTCTACTTTATGTGTTATAATAATTTGTGTTTATAATTGAAAACTTTAATTATTATAATTTGAG

TTCATATGAACTATAAGGAGTGTTTGTGGTAAGGGAAACTCAGCCTTATGTTGTAGTAATTAAAATCCTGAATAACTTATXXXXXXXXXXTTAGTTGGTTATTATAGTCTACTTTATGTGTTATAATAATTTGTGTTTATAATTGAAAACTTTAATTATTATAATTTGAG

>Marker163987

ATAGTAGGAAACTCCATATCGATCTTATCTTTTGGTTGAAATCCCTCGGATCTGTCCAACACATTGGACCTAACATTAGTXXXXXXXXXXCCTATCCCAACTGTCAGAAACATCCTTCTCCATAACGATACACATAGCCTCGGCATTCTATCTTAACCCACCGCATGTTG

ATAGTAGGAAACTCCATATCGATCTTATCTTTTGGTTGAAATCCCTCGGATCTGTCCGACACATTGGACCTAACATTAGTXXXXXXXXXXCCTATCCCAACTGTCAGAAACATCCTTCTCCATAACGATACACATAGCCTCGGCATTCTATCTTAACCCACCGCATGTTG

>Marker164417

CAAACTTCATCAAAAGAACCAAGCATTCTACTAAAATAAAGTTCCCAGAATATTAAGGACATACTTGCAAATATGACTATXXXXXXXXXXAAGAAAACTAAACTACAAATCCAACAACACCCTCAAGAGGAAAGCAAGTTTGTTCATTAGAAATATTAATAATAACAAAA

CAAACTTCATCAAAAGAACCAAGCATTCTACTAAAACAAAGTTCCCAGAATATTAAGGACATACTTGCAAATATGACTATXXXXXXXXXXAAGAAAACTAAACTACAAATCCAACAACACCCTCAAGAGGAAAGCAAGTTTGTTCATTAGAAATATTAATAATAACAAAA

>Marker164530

CCACTGGCTTCTCCAACATATCTCAACACTCATAGTGTATCTATATATGAAGAATCAAGCAACAATAATCACCAGCCATAXXXXXXXXXXTACAGGGTATCGAAGCAACTGTGTTGGGGCTGCATTAGCGAGTCCATAGAGTTTCTATTTGCGCACAATCTGGTTCGAGC

CCACTGGCTTCTCTAACATATCTCAACACTCATAGTGTATCTATATATGAAGAATCAAGCAACAATAATCACCAGCCATAXXXXXXXXXXTACAGGGTATCGAAGCAACTGTGTTGGGGCTGCATTAGCGAGTCCATAGAGTTTCTATTTGCGCACAATCTGGTTCGAGC

>Marker164620

TATACTACTACTACAAATGTCCTCTGCAAGAAACCAATTTTTTGTTTCTTCTTTCTTTCTCTTTACTTCTTCTTCTTAATXXXXXXXXXXACTTCTGGGGGTTCTTCTAATAAAAAATCCGCATCTGCCTTCGGTGGAGCTCCTGCGGCGGCGCCACCGCCCCCGTGTCA

TATACTACTACTACAAATGTCCTCTGCAAGAAACCAATTTTTTGTTTCTTCTTTCTTTCTCTTTACTTCTTCTTCTTAATXXXXXXXXXXACATCTGGGGGTTCTTCTAATAAAAAATCCGCATCTGCCTTCGGTGGAGCTCCTGCGGCGGCGCCACCGCCCCCGTGTCA

>Marker164947

CTAATCACATCATAATTGCATAAGCATATCTATTGATATATAATATCAGAGATGCAAGGGATGGTTCCAACAATCTTAAGXXXXXXXXXXGAAGATGTTCCTTTTCAGAAGCAAGACATTGAGATGTTCAACAAAAAACAAATGTGATTTTCTAGACCAAGTTTACTACT

CTAATCACATCATAATTGCATAAGCATATCTATTGATATATAATATCAGAGATGCAAGGGATGGTTCCAACAATCTTAAGXXXXXXXXXXGAAGATGTTCCTTTTCAGAAGCAAGACATTGAGATGTTCAACAAAAAACAAATGTGACTTTCTAGACCAAGTTTACTACT

>Marker165236

AAATCAGAGGGAAATTTTCAGGTTAACAATGCACTTGTCGCCCTCTTGCATGACTGGTTTCGAATTGGCTACTCAGGAAAXXXXXXXXXXTTTCTTGCACTGCTCCCGCTTATGGAACATCTTATTCTAGTGGAATCAATCTGCAAATACATTTTCCAGGAACAGTGAAG

AAATCAGAGGGAAATTTTCAGGTTAACAATGCACTTGTCGCCCTCTTGCATGACTGGTTTCGAATTGGCTACTCAGGAAAXXXXXXXXXXTTTCTTGCACTGCTCCCGCTTATGGAACATCTTATTCTAGTGGAATCAATCTGCAAATACATTTTCCAGGAACAGTTAAG

>Marker165274

ATATCTGATTCAAACAATGGCACAAAACACACACGATTTCAAAATCCATGCACAAACACAACAATCCACCAAAATATTCTXXXXXXXXXXAATCATCATTTCAAAATCATTATTATTAGCAGCAAGAGAATTGCACAAACGACCGATACCAATTCCAGTGGCATGGACAA

ATATCTGATTCAAACAATGGCACAAAACACACACGATTTCAAAATCCATGCACAAACACAACAATCCACCAAAATATTCCXXXXXXXXXXAATCATCATTTCAAAATCATTATTATTAGCAGCAAGAGAATTGCACAAACGACCGATACCAATTCCAGTGGCATGGACAA

>Marker165469

CTTAAGTAGTAATATTGCAGTCAAAGAGTGTTACTCTACTTAGAGTTAGAGTTGGTTAGGTAGATCTTTGGTTTGCACTCXXXXXXXXXXGACATACAACATGAGTTCTTAGGTTTGATTTCATCTCATCTCTATAAAACAAACAAAAATCATGTGGATGAGGAACTAAG

CTTAAGTAGTAATATTGCAGTCGAAGAGTGTTACTCTACTTAGAGTTAGAGTTGGTTAGGTAGATCTTTGGTTTGCACTCXXXXXXXXXXGACATACAACATGAGTTCTTAGGTTTGATTTCATCTCATCTCTATAAAACAAACAAAAATCATGTGGATGAGGAACTAAG

>Marker165714

TTCAGTTTGAAACCATACTCTCAGTTTCCTTAACAAAGCCATCGCCTGTCCAGTCTTTATCAGATTGCAGACCCTTCTTCXXXXXXXXXXAGAAGTTGTGAAGTTTCATGAAGTCCTGAAGCTCAGCACATAGTTTCTTTACAACGCCATACCCATGCATCATCACTCCT

TTCAGTTTGAAACCATACTCTCAGTTTCCTTAACAAAGCCGTCGCCTGTCCAGTCTTTATCAGATTGCAGACCCTTCTTCXXXXXXXXXXAGAAGTTGTGAAGTTTCATGAAGTCCTGAAGCTCAGCACATAGTTTCTTTACAACGCCATACCCATGCATCATCACTCCT

>Marker165776

AAGAAACTAACATGGGCATATTTTTTGACATTTGGTCACCCTTTTTGCTTCTACAGTCTTTTACTCCATTTTTAAAAATTXXXXXXXXXXTACTGTGCCAGATTCTACAAAATATTGTTTCTCATGCTAAAGATACTGGTTCTACGTCACAATTACTCAGCACGAAAGAG

AAGAAACTAACATGGGCATATTTTTTGACATTTGGTCACCCTTTTTGCTTCTACAGTCTTTTACTCCATTTTTTAAACTTXXXXXXXXXXTACTGTGCCAGATTCTACAAAATATTGTTTCTCATGCTAAAGATACTGGTTCTACGTCACAATTACTCAGCACGAAAGAG

>Marker165781

TCAGTCTTGTTGATGTAGGGTATACAGATTTTGATTCACAAATTTGCTTCATTTTAAATGACTAATTTTGCCCTTCATTTXXXXXXXXXXACTTAACTGCTGTAATCTATTTCATGGTTGCTTACTTCACTGCTTTTTCTAAAATTAAATTTCCTTCTTTTTAAAAAATA

TCAGTCTTGTTGATGTAGGGTATACAGATTTTGATTCACAAATTTGCTTCATTTTAAATGACTAATTTTGCCCTTCATTTXXXXXXXXXXACTTAACTGCTGTAATCTATTTCATGGTTGCTTACTTAACTGCTTTTTCTAAAATTAAATTTCCTTCTTTTTAAAAAATA

>Marker165800

TGCAACGAACTGGCGTTGCTTTTGTTCTTGGCACAAATGCTAAACCGTCTCGCTCTCTTTTGCGTCGCCGAAGAATTACTXXXXXXXXXXCACGCAACAACGCATATGATGGGCTCTCTCTCTCTCTCTCTCGAGATTGTTTGGGCTTCTTCACTCAAACCCAACATGTG

TGCAACGAACTGGCGTTGCTTTTGTTCTTGGCACAAATGCTAAACCGTCTCGCTCTCTTTTGCGTCGCCGAAGAATTACTXXXXXXXXXXCACGCAACGACGCATATGATGGGCTCTCTCTCTCTCTCTCTCGAGATTGTTTGGGCTTCTTCACTCAAACCCAACATGTG

>Marker165934

CAATCCAATTTCATACACCTAGAAGTCGAGCATACAAGATTAGTCTCTAAATCAGAGTCAACAGTTGCCTTTTGTGTTCAXXXXXXXXXXGGTTGAGTAATTGTTTTTTCAATGATGGTTCCTATATTGGTATGTAGTATTAGTGTGCTTATACTTTCCTTTAATTTAGG

CAATCCAATTTCATACACCTAGAAGTCGAGCATACAAGATTAGTCTCTAAATCAGAGTCAACAGTTGCCTTTTGTGTTCAXXXXXXXXXXGGTTGAGTAATTGTTTTTTCAATGATGGTTCCTATCTTGGTATGTAGTATTAGTGTGCTTATACTTTCCTTTAATTTAGG

>Marker165940

TGGGTTTATGAAAAAAAGCTGAAGTTATTATTTGATGATTTATGAATTAGTTAATTAAGTTGGGAATCACAGAAAATGGGXXXXXXXXXXAGAGAAAGAAAGAAAGAAGAGAGTATTTAAGAGAAATAAAAGAAAGAGGAGAAGGTGAAATTTGGTGTGTATTTGACTAT

TGGGTTTATGAAAAAAAGCTGAAGTTATTATTTTATGATTTATGAATTAGTTAATTAAGTTGGGAATCACAGAAAATGGGXXXXXXXXXXAGAGAAAGAAAGAAAGAAGAGAGTATTTAAGAGAAATAAAAGAAAGAGGAGAAGGTGAAATTTGGTGTGTATTTGACTAT

>Marker166148

ATGAAGCATCAGGGTAACTTCTTCTAACTGAATTTTTTATTTCCTAATTTTGTGGTTGAATCCTTCTTCTTACTGGCTGTXXXXXXXXXXCCTAGAATTGGTAACCTGCAAGAGCAAAATTCTTGATAAGGAAACCCATGGAAAAGCATGGAAGAGAAACACCAAAAAGA

ATGAAGCATCAGGGTAACTTCTTCTAACTGAATTTTTTATTTCCTAATTTTGTGGTTGAATCCTTCTTCTTACTGGCTGTXXXXXXXXXXCCTAGAAGTGGTAACCTACAAGAGCAAAATTCTTGATAAGGAAACCCATGGAAAAGCATGGAAGAGAAACACCAAAAAGA

>Marker166188

ATATGATACTTTGATCAAGCTTATTGAATTCAAAACATAAGCAAATCCACTGTAGTTTATAGACCGCTCAAATTTATGTTXXXXXXXXXXATCTGACGTTGAATTAACTTTGCATGGCGCTAAATTTGAAGAATTTCAACAAAGGCGGTTATGTATTTTCAAAAATTGAA

ATATGATACTTTGATCAAGCTTATTGAATTCAAAACATAAGCAAATCCACTGTAGTTTATAGACCACTCAAATTTATGTTXXXXXXXXXXATCTGACGTTGAATTAACTTTGCATGGCGCTAAATTTGAAGAATTTCAACAAAGGCGGTTATGTATTTTCAAAAATTGAA

>Marker166252

TTTGATTCCGACCATTGCACAATCGATTAGTGCTGGAAATACCGAAGAGGGAATAAATAATTGAGGGCAATAGCGTATACXXXXXXXXXXAAAAATCTGCAAGCAAGATCAGCAGTTTAACTGTTAGCGTAACACAATAATCGAAATTTCATAGCGAAGTGAGATGTATG

TTTGATTCCGACCATTGCACAATCGATTAGTGCTGGAAATACTGAAGAGGGAATAAATAATTGAGGGCAATAGCGTATACXXXXXXXXXXAAAAATCTGCAAGCAAGATCAGCAGTTTAACTGTTAGCGTAACACAATAATCGAAATTTCATAGCGAAGTGAGATGTATG

>Marker166295

ATGACTCGAACATCATCTGAATCAATGGTTGATAATGTGGTCCAATTATGTGCAGTAGCAGCCCCAACGCCACTGCAGAAXXXXXXXXXXGCATTGTCGACCAAGAGTAGCCATCCATCGTTTTGCGCCAAAGGCAAGCCCACATGTCACCACTCCTTTGTATAAACTGT

ATGACTCGAACATCATCTGAATCAATGGTGGATAATGTGGTCCAATTATGTGCAGTAGCAGCCCCAACGCCACTGCAGAAXXXXXXXXXXGCATTGTCGACCAAGAGTAGCCATCCATCGTTTTGCGCCAAAGGCAAGCCCACATGTCACCACTCCTTTGTATAAACTGT

>Marker166360

ATACTATTCCTTGAACGCTACTAATCATCATCTCTTCACCTTCATTACAAACCACCAAAAGACAAATATTTGTTAATACAXXXXXXXXXXCACATCATTGGTCCCTGATGTATTTTTTGAAGAGCTATTATTAGGCGAAACAAGTTATATAGATTTAAAGTTTTAGTATT

ATACTATTCCTTGAACGCTACTAATCATCATCTCTTCACCTTCATTACAAACCACCAAAACACAAATATTTGTTAATACAXXXXXXXXXXCACATCATTGGTCCCTGATGTATTTTTTGAAGAGCTATTATTAGGCGAAACAAGTTACATAGATTTAAAGTTTTAGTATT

>Marker166466

TACCTTTTTTCTCTCTTTCTTTATTTTATTTTGTTTTTCTATAAAACAAAGATCCCTACTTCTATAATACAGCTGGCAGCXXXXXXXXXXCTAATTTACGTTTATGTTTTCTTCTTATATTAATATTCTTTTTAGTTTAATGTATATATCATTTTTCAACTTAAATTTTT

TACCTTTTTTCTCTCTTTCTTTATTTTATTTTGTTTTTCTATAAAACAAAGATCCCTACTTCTATAATACAGCTGGCAGCXXXXXXXXXXCTAATTTACGTTTATGTTTTCTTCTTATATTAATATTGTTTTTAGTTTAATGTATATATCATTTTTCAACTTAAATTTTT

>Marker166591

TTCTCGCATCAGATTCAAGGTTTGGAACAAACAAGTTGAAGGTATGTCGGTCAATTTTTATGTCTTCGTTTTTTCAACATXXXXXXXXXXGGGCTCTTCATAGTAGAGTTCAAACCAAAGATCCTTCATGGAGATTAGCTGGCTTTGTTGTGGATGATCCTCTTGCTGAT

TTCTCGCATCAGATTCAAGGTTTGGAACAAACAAGTTGAAGGTATGTCAGTCAATTTTTATGTCTTCGTTTTTTCAACATXXXXXXXXXXGGGCTCTTCATAGTAGAGTTCAAACCAAAGATCCTTCATGGAGATTAGCTGGCTTTGTTGTGGATGATCCTCTTGCTGAT

>Marker166616

ACAGTAAAAGTTGTGATACATATTTGTATGGAATTTCCACTGTAAAAGAAAAGACAAAGTTCTCCGTATAAAAGAGGTGAXXXXXXXXXXAAAATCAGGATTACAAAAGATTCCTCGTTATTAAAAGAGAGGAAAATCGGGATAATACACCAAGATACATAGAAACCCTA

ACAGTAAAAGTTGTGATACATATTTGTATGGAATTTCCACTGTAAAAGAAAAGACAAAGTTCTCCTTATAAAAGAGGTGAXXXXXXXXXXAAAATCAGGATTACAAAAGATTCCTCGTTATTAAAAGAGAGGAAAATCGGGATAATACACCAAGATACATAGAAACCCTA

>Marker166751

CACAAAATTTAGAAGCTATAATGAAGTCCCATGTTTGAGTCCAAGAAGCTCAAATGGGTATCCGGTTAAAAAAAAGAATGXXXXXXXXXXTTTCAGTTCATAGACCTGCACCTAAAAATGTCTATAAACATTAAAGACCCAAACATCCATCAATAGACTCTTATAGTTGA

CACAAAATTTAGAAGCTATAATGAAGTCCCATGTTTGAGTCCAAGAAGCTCAAATGGGTATCCGGTTAAAGAAAAGAATGXXXXXXXXXXTTTCAGTTCATAGACCTGCACCTAAAAATGTCTATAAACATTAAAGACCCAAACATCCATCAATAGACTCTTATAGTTGA

>Marker167131

AGAGAACAGTTCTCTAGCTTGATCTTTCATTTTCCAAACAGTTCAAATTTTAGGATTGGTTCTAATGACTTGTCTTCAATXXXXXXXXXXAAATAACGAGTTCCAATTACCTGGCAATTTGAACAATACTCATAAGGGGCAGAGATATATAACGAGAGGGAATAAGTTTT

AGAGAACAGTTCTCTAGCTTGATCTTTCATTTTCCAAACAGTTCAAATTTTAGGATTGGTTCTAATGACTTGTCTTCAATXXXXXXXXXXAAATAACGAGTTCCAATTACCTGGCAATTTGAACAATACTCATAAGGGGCAGAGATATGTAACGAGAGGGAATAAGTTTT

>Marker167335

AACATATCATATTATAACGTCCACGACATAATACTTTACTCTTTCTCGCCAACATACTTTGGCTTGTCGAAAGCCTCTTTXXXXXXXXXXTGTAACCAATGACATTGGATTGAAGTCTATCGATTATTATCTCCTTCGTGAGTTATTTTTTTAAGGGGTTCAATTGGTGG

AACATATCATATTATAACGTCCACAATATAATACTTTACTCTTTCTCACCAACATACTTTGGCTTGTCGAAAGCCTCTTTXXXXXXXXXXTGTAACCAATGACATTGGATTGAAGTCTACCGATTATTATCTCCTTCGTGAGTTATTTTTTTAAGGGGTTCAATTGGTGG

>Marker167542

AAGATACATCCAGTGCGGGAAATGAATGTATAATTTTGAACTCAATTGCTTAAAAATGAACGAATATATGAACTCATCAAXXXXXXXXXXGAAGAGTGACCCTGTTTTTTTACACGAGAAAACCCTAAAATCTATGAAACTAAGAGCTAGTGAACTTGGTAACAGCCTTG

AAGATACATCCAGTGCGGGAAATGAATGTATAATTTTGAACTCAATTGCTTAAAAATGAACGAATATATGAACTCATCAAXXXXXXXXXXGAACAGTGACCCTGTTTTTTTACACGAGAAAACCCTAAAATCTATGAAACTAAGAGCTAGTGAACTTGGTAACAGCCTTG

>Marker167641

TGTTTTGTTCTAGTCCAGGGACTTGGATCTTGACACAACACGTATCAAGCTAGTTGGGGTTTGGTCATTCTTTGTTATAGXXXXXXXXXXGTTAATGTTTTTGTCTGGTAGATTCTCCATGGAAGAGAAATAACACCTTGGGTAGGAGTTTGAGGCTACTCGATTTTATG

TGTTTTGTTCTAGTCCAGGGACTTGGATCTTGACACAACACGTATCAAGCTAGTTGGGGTTTGGTCATTCTTTGTTATAGXXXXXXXXXXGTTAATGTTTTTGTCTGGTAGATTCTCCATGGAAGAGAAATAAGACCTTGGGTAGGAGTTTGAGGCTACTCGATTTTATG

>Marker167933

CATATTCTTTAAAATTTCCACGTCATTTCTCACCTCATATTTATGACTACTAAGTTCCCATAATTTTAAATCATTGACTGXXXXXXXXXXATTAATATATGATCATAGATCCATATATAACTACCAAGTTAAATCCTATTATCAAAATCTATAGTGTATGTTTGGAGTAA

CATATTCTTTAAAATTTCCACGTCATTTCTCACCTCATATTTATGACTACTAAGTTCCCATAATTTTAAATCATTGACTGXXXXXXXXXXATTAATATATGTTCGTAGATCCATATATAACTACCAAGATAAATCCTATTATAAAAATCTATAGTGTATGTTTGGAGTAA

>Marker167941

CCAAAGAACACTCCGCCCTACCATGACATTTCCTGCTCGAAGTCGTCAACGGTAGCCAACAGTAGTATCTGGAAACTTCAXXXXXXXXXXTTGTTGTAAGTTGTAAATGAATAAGAATTGGGGGTGAAATATTTTAAATTTGGGTGGGGGTAGTGAACAACTGTTAAAAA

CCAAAGAACACTCCGCCCTACCATGACATTTCCTGCTCGAAGTCGTCAACGGTAGGCAACAGTAGTATCTGGAAACTTCAXXXXXXXXXXTTGTTGTAAGTTGTAAATGAATAAGAATTGGGGGTGAAATATTTTAAATTTGGGTGGGGGTAGTGAACAACTGTTAAAAA

>Marker167951

GAAAGCAGCACGAGCAACATTGGTGAGAAATGATACAGCGGCTTGGCTGCTACCCATCCTGAAGAGAAAATGGAGAACACXXXXXXXXXXTTCTGCCTCAGTGATTTGTATTCTGACCCTAATAATTACTAATCGGTTTCCATCATCTGTTTGCTTTGAAGTAAAATTGT

GAAAGCAGCACGAGCAACATTGGTGAGAAATGATACAGCGGCTTGGCTGCTACCCATCCTGAAGAGAAAATGGAGAACACXXXXXXXXXXTTCTGCCTCAGTGATTTGTATTCTGACCCTAATAATTACTAATCGGTTTCCATCATCTGTTTGCTTTGAAGTGAAATTGT

>Marker168166

AATATCTCCATATGCAATTGTTGTTCTTCGGTCGGACAAGGCAAGATGCAATAATTGTATTATATTTTATACCAACAAAGXXXXXXXXXXTCAATATATCATAAATCAGAATTTCTTACTTTCTCTCTTCTAGCATTCCGAGTAAAATTAGTGTTTGTTTGAGTGTTGGT

AATATCTCCATATGCAATTGTTGTTCTTCGGTCGGACAAGGCAAGATGCAATAATTGTATTATATTTTATACCAACAAAGXXXXXXXXXXTCAATATATCATAAATTAGAGTTTCTTACTTTCTCTCTTCTAGCATTCCGAGTAAAATTAGTGTTTGTTTGAGTGTTGGT

>Marker168841

CATGAGATTTGTTGCCACAAGAATACAAAACCTGCTCATCTCCATCACCACTCATGGGATGAATTGTAATATTCAATGCCXXXXXXXXXXAATTTTTCCAAGCAGGGACAAACATCTTCCTAGTTTAGTGAGGAAAGGAGGAGAGAACGGGTATATATTTTAGAGTAAAA

CATGAGATTTGTTGCCACAAGAATACAAAACCTGCTCATCTCCATCACCACTCATGGGATGAATTGTAATATTCAATGCCXXXXXXXXXXAATTTTTCCAAGCAGGGACAAACATCTTCCTAGTTTAGTGAGGAAAGGAGGAGAGAACGGGTATATATTTCAGAGTAAAA

>Marker168857

TCTTTGTTTAATATTTCTGTTATATACTACACTCTAAACAAACAAAATTCAAGTTTCAATTAAATGAACGAATTTCAAACXXXXXXXXXXTTTTTGGTCATCTTTCCAGGTTGCTCGAGGGTAATAATCCTTTCACAATAAATTTTTACATGTTTAGAAGATATATATTT

TCTTTGTTTAATATTTCTGTTATATACTACACTCTAAACAAACAAAATTCAAGTTTCAATTAAATGAACGAATTTCAAACXXXXXXXXXXTTTTTGGTCATCTTTTCAGGTTGCTCGAGGGTAATAATCCGTTCACAATAAATTTTTACATGTTTAGAAGATATATATTT

>Marker169004

TTTTTTCATTATTCATATATGTTTCATTTATGACACTTCATTTCATCTTCTCAACATTGTTTGCATTAATTCATACATTAXXXXXXXXXXGGTTGATGACAAAACATATTATATATAGTTTATTTTTCCTATTGATTTCTTGTTTCACGAAACCTGTTATAACTTGAATG

TTTTTTCATTATTCATATATGTTTCATTTATGACACTTCATTTCATCTTCTCAACATTGTTTGCATTAATTCATACATTAXXXXXXXXXXAGTTGATGACAAAACATATTATATATAGTTTATTTTTCCTATTGATTTCTTGTTTCACGAAACCTGTTATAACTTGAATG

>Marker169057

TTTTGAAGAACTTGAAGTAGCAGGGATCGTGCTGTAAGAAATGAACGGAGGACCGCTAAGTGAGAGCTTTCCAAGATAGGXXXXXXXXXXTGGGAGGAGAGAGGAGACCATTCTCCTAGAGGTTCCCTTAGAAATGATTAGTAAACAGGGCTTAACTTTTTAATCCCTTA

TTTTGAAGAACTTGAAGTAGCAGGGATCGTGCTGTAAGAAATGAACGGAGGACCGCTAAGTGAGAGCTTTCCAAGATAGGXXXXXXXXXXTGGGAGGAGAGAGGAGACCATTCTTCTAGAGGTTCCCTTAGAAATGATTATTAAACAGGGCTTAACTTTTTAATCCCTTA

>Marker169198

GAACATAATACGATTGGGGTTCCAAAGAATGGAATACGTGTGGAAATCAGCAGTGGGGTCAAACCAAAGATGGAACTGTTXXXXXXXXXXTTAATTACATAATAAGCAGTGACAGTGCCAGCAGAATTGCCAGCCACAAGCTTGAGTTGCATATCAATCTTTCCAAACAA

GAACATAATACGATTGGGGTTCCAAAGAATGGAATAGGTGTGGAAATCAGCAGTGGGGTCAAACCAAAGATGGAACTGTTXXXXXXXXXXTTAATTACATAATAAGCAGTGACAGTGCCAGCAGAATTGCCAGCCACAAGCTTGAGTTGCATATCAATCTTTCCAAACAA

>Marker169220

ACATCGACATATCCAAATGCGTTGGTGCCAGGGGGGCACTCGAGGGCAGCTTCAAGAACCCTATGATGTATTCCATGGGAXXXXXXXXXXAGGTATCTTCTTCCTAAGCCAACCAATCCTGATGGGCTGTTTTTATCTGCATTTGGGTTTCTCTGAGATAAGATTTTAAG

ACATCGACATATCCAAATGCGTTGGTGCCAGGGGGGCACTCGAGGGCAGCTTCAAGAACCCTATGATGTATTCCATGGGAXXXXXXXXXXAGGTATCTTCTTCCTAAGCCAACCAATCCTGATGGGCTGTTTTTATCTGCATTTGGGTTTCTCTGAGACAAGATTTTAAG

>Marker169579

TTCTCTTGACTTTAGACGTTACAAATGTATACAATTTGAAAGCATAACCTAACCAAATTGTTAGAAATCAAATTCCAAAAXXXXXXXXXXCAAAACAATCGATAAAAAGTAGATGACAAAATCAAAAATTCAAAACTGAACTATAGTGTGTATAAGCTTAATGGTTACTT

TTCTCTTGACTTTAGACGTTACAAATGTATACAATTTGAAAGCATAACCTAACCAAATTGTTAGAAATCAAATTCCAAGAXXXXXXXXXXCAAAACAATCGATAAAAAGTAGATGACAAAATTAAAAATTCAAAACTGAACTATAGTGTGTATAAGCTTAATGGTTACTT

>Marker169693

TATTAGAGAATTTTATCCTTAAAAACATCAATGAAAAGAGTTTGTATCTAGTTAAAAAAAACATACAAGAACAACTAGAAXXXXXXXXXXGTCTGTTAGATGAGAAAAAAACGTCAATAACAGTCTAACAAAGAAGACTTAGGAATGTAAACAAAATGAAAAAGATGGTG

TATTAGAGAATTTTATCCTTAAAAACATCAATGAAAAGAGTTTGTATCTAGTTAAAAAAAACACACAAGAACAACTAGAAXXXXXXXXXXGTCTGTTAGATGAGAAAAAAACGTCAATAACAGTCTAACAAAGAAGACTTAGGAATGTAAACAAAATGAAAAAGATGGTG

>Marker169858

GGACGGGGATTGGTCTGTGGGTGGGTCCCGCAGGTGTGTTTTCTCTCTTCTTTTAATTTGTGAGGAAAAATGATGTTTGTXXXXXXXXXXTTGGGAATTTTAGATGGCAAATGTATTGTGTATCTTCATCCCTGTTTTACCAACCGATCGAAAACTTCCCATCCCAAATA

GGACGGGGATTGGTCTGTGGGTGGGTCCCGCAGGTGTGTTTTCTCTCTTCTTTTAATTTGTGAGGAAAAATTATGTTTGTXXXXXXXXXXTTGGGAATTTTAGATGGCAAATGTATTGTGTATCTTCATCCCTGTTTTACCAACCGATCGAAAACTTCCCATCCCAAATA

>Marker169868

ATTGCTCCAAGGGTAACAAAAAAGAACAAGAGAACGAAAGTAAAAGAAAATTATTTATGTAGGATATATTAGAAAAAGAAXXXXXXXXXXTCAAAAAAAAATCCAAATGAAGTCACTTTTCTCACTTCACTGAAGAATTTTGCATACTAAAAACCTGAGTTTTTCTAATT

ATTGCTCCAAGGGTAACAAAAAAGAACAAGAGAACGAAAGTAAAAGAAAATTATTTATGTAGGATATATTAGAAAAAGAAXXXXXXXXXXTCAAAAAAAAATTCAAATGAAGTCACTTTTCTCACTTCACTGAAGAACTTTGCATACTAAAAACCTGAGTTTTTCTAATT

>Marker169887

TCAAGAAGTAAATTTATCTATAACACATCATATTCAGCTTCAAGTTTTAACATGTTTCTCATGTTAATTTTTTTCCTCTAXXXXXXXXXXGTTCAAGAGAGCATAGCTCGTGGTTTCTGTCAACAGCTGGTCCAGATGTGCCAGATCTCTGGCATGGTAAACAGTTCCCT

TCAAGAAGTAAATTTATCTATAACACATCATATTCAGCTTCAAGTTTTAACATGTTTCTCATGTTAATATTTTTCCTCTAXXXXXXXXXXGTTCAAGAGAGCATAGCTCGTGGTTTCTGTCAACAGCTGGTCCAGATGTGCCAGATCTCTGGCATGGTAAACAGTTCCCT

>Marker170085

TTTTAATGAAGATTAAAGTAGTATTTTTCAATATACACATAAGAAATAACAACATATAGTGAATAAAATGGGGAGAGGTGXXXXXXXXXXACGAACATGCTTAACCTAAGTTGCGATTTATCTAAATGACTTCCACAAAGCATTTAAATAGCAATTGAACCTATAAAATT

TTTTAATGAAGATTAAAGTAGTATTTTTCAATATACACATAAGAAATAACAACATATAGTCAATAAAATAGGGAGAGGTGXXXXXXXXXXACGAACATGCTTAACTTAAGTTGCGATTTATCTAAATGACATCCACAAAGCATTTAAATAGCAATTGAACCTATAAAATT

>Marker170263

TAACCCTAACCGTAGTAAAACAATTACAACCTTTTATACACAATACATTTGCATACATAGAGAGAGATATTCAAAGCCATXXXXXXXXXXCAGTCTCTCCATGTCTTCTCTCTATTTCCATCTCTCGCTGCCACCGTCGTCCCTCCACCGTGAAGCTCGTCTTCACCAAT

TAACCCTAACCGTAGTAAAACAATTACAACCTTTTATACACAATAGATTTGCATACATAGAGAGAGATATTCAAAGCCATXXXXXXXXXXCAGTCTCTCCATGTCTTCTCTCTATTTCCATCTCTCGCTGCCACCGTCGTCCCTCCACCGTGAAGCTCGTCTTCACCAAT

>Marker170639

CTTACCATTTCTTTTTTAAGAAACTGAGCTTCCAATGAGCAAAATGAAGGAATATACAAGAAATTACAAAGTTCTGGGCAXXXXXXXXXXTTGGAGGGAGTCAGACTATTTGAGTGAAACAAACTGAATGTGAAACTAGTGAAAAAGCTTATGCCAATGTTTACTTGAAG

CTTACCATTTCTTTTTTAAGAAACTGAGCTTCCAATGAGCAAAATGAAGGAATATACAAGAAATTACAAAGTTCTGGGCAXXXXXXXXXXTTGGAGGGAGTCAGACTACTTGATTGAAACAAACTGAATGTGAAACTAGTGAAAAAGCTTATGCCAATGTTTACTTGAAG

>Marker171007

CCGGAAAGAATGAGCGGCATGGTTTTCCTCTGAATTGGAGTGGGCACTCTGTAACCCTTGCGCTTGATTCCCCTGAAGACXXXXXXXXXXGGAATTCAATTCGGCGTGCGGCTGCTGCAACCATGAACGAGGGAGAAGTGAACACCATATGCTCGTAGACCAATAAGACA

CCGGAAAGAATGAGCGGCATGGTTTTCCTCTGAATTGGAGTGGGCACTCTGTAACCCTTGCGCTTGATTCCCCTGAAGACXXXXXXXXXXGGAATTCAATTCGGCGTGCGGCTGCTGCAACCATGAACAAGGGAGAAGTGAACACCATATGCTCGTAGACCAATAAGACA

>Marker171237

TCTGTAACTTTGTATGTGCTGTATCTTGATGGAATTATGTCATTTTGATTTAGAAGATGCATATACTGCTTAGTGAAATAXXXXXXXXXXGTGGTCAAGCATTTGAGGGTCTTTTGCTACTTCAAGTTTTCTTATGGGTTTACTCTTTACTTACTAATAACTTTTTTCTT

TCTGTAACTTTGTATGTGCTGTATCTTGATGGAATTATGTCATTTTGATTTAGAAGATGCATATACTGCTTAGTGAAATAXXXXXXXXXXGTGGTCAAGCATTTGAGGGTCTTTTGCCACTTCAAGTTTTCTTATGGGTTTACTCTTTACTTACTAATAACTTTTTTCTT

>Marker171334

CAACCCATAAAAAGTTATCTAGATTTTGCAAATTTTGTTGTTTTCCGCTTGGTTTGTGTTGTTAATGGAAAGAAGTTGAAXXXXXXXXXXTCTTTGAGGGACCTCGAGTGCAGTGATGCTTAGAAAGTGGAAAAAAAAAACAAAGAAACAAAAAAGAAGTGTTTCTGTTG

CAACCCATAAAAAGTTATCTAGATTTTGCAAATTTTGTTGTTTTCCGCTTGGTTTGTGTTGTTAATGGAAAGAAGTTGAAXXXXXXXXXXTCTTTGAGGGACCTCGAATGCAGTGATGCTTAGAAAGTGGAAAAAAAAAACAAAGAAACAAAAAAGAAGTGTTTCTGTTG

>Marker171364

CTTTCAGCTCTGAAAACTCCAATCTCCTCTTCTGAACTTGCCGTCTTCTTCATGTTTTTAGAACACAAAGCCTTTTGGTTXXXXXXXXXXCCGAGGGGGAGAAGAGAGCACACCACACAGAATGAAGGGGAGAGAAGCTGGTGGGAAGAACAGAAACAAACAAGAAACAA

CTTTCAGCTCTGAAAACTCCAATCTCCTCTTCTGAACTTGCCGTCTTCTTCATGTTTTTAGAACACAAAGCCTTTTGGTTXXXXXXXXXXCCGGGGGGGAGAAGAGAGCACACCACACAGAATGAAGGGGAGAGAAGCTGGTGGGAAGAACAGAAACAAACAAGAAACAA

>Marker171748

AATACTTATTAGATACCTATTAAACTTTAGATATTTTTAAACACGAGATTACGACGTCTAAAAGTTTTTTAGTAAAATTTXXXXXXXXXXTTTCAATTATAAAACAATGTATAAAGCCCAACATTATCATCTCCACTAAAGAAATAATGAATTTGAAAGTTTGGATTATG

CATACTTATTAGATACCTATTAAACTTTAGATATTTTTAAACACGAGATTACGACGTCTAAAAGTTTTTTAGTAATCTTTXXXXXXXXXXTTTCAATTATAAAACAATGTATAAAGCCCAACATTATCATCTCCACTAAAGAAATAATGAATTTGAAAGTTTGGATTATG

>Marker171904

CGAGCATCAAAGGCATTTGAAACCAGCCCAAATCCATGCATAGGTCTCTGTTGTAGAGAAACCTAAAAAGAAGACAAGTTXXXXXXXXXXAAAAAAAAGAATGCTAACTGACTAGCTCAAGTTAGATATTATCAAAGAGTTCTTCCTCCATCCTCCTCTTTTCATGGAAG

CGAGCATCAAAGGCATTTGAAACCAACCCAAATCCATGCATAGGTCTCTGTTGTAGAGAAACCTAAAAATAAGACAAGTTXXXXXXXXXXAAAAAAAAGAATGCTAACTGACTAGCTCAAGTTAGATATTATCAAAGAGTTCTTCCTCCATCCGCCTCTTTTCATGGAAG

>Marker172185

CTTTATTATTATTATTTTTGTTTTCCTTTTCTGGTATATAGTCATAAAAAACAAGTGTGGTTAAATAATCGAATCCAAATXXXXXXXXXXTAAATTCATAATAGTAAATATGTTCTTCAGATTCATAAGCCCAAAAATTACAATGATATTCATTACATCAATCAATTTAT

CTTTATTATTATTATTTTTGTTTTCCTTTTCTGGTATATAGTCATAAAAAACAAGTGTGGTTGAATAATCGAATCCAAATXXXXXXXXXXTAAATTCATAATAGTAAATATGTTCTTCAGATTCATAAGCCCAAAAATTACAATGATATTCATTACATCAATCAATTTAT

>Marker172234

ATTTGTCGACATTGCACAGACCATAAGAAGCGCCATCGCTGTCCGTCGTAGTCTACCTTTGCCGATCGCCAGGAAACACCXXXXXXXXXXTCTAGGGTTTTGTTCCAATTTTGCAGCTGTTTGGATTGATTTTTTTTTTGTTTAAGTGGACTACAATCAGTGTGCAGTAT

ATTTGTCAACATTGCACAGACCATAAGAAGCGCCATCGCTGTCCGTCGTAGTCTACCTTTGCCGATCGCCAGGAAACACCXXXXXXXXXXTCTAGGGTTTTGTTCCAATTTTGCAGCTGTTTGGATTGATTTTTTTTTTGTTTAAGTGGACTACAATCAGTGTGCAGTAT

>Marker172301

CTCTTTCACCCAACCAATTCTTCACCTCAACCAAGCCAGGTATTCAAATTCTTTCCTATTTCCTCCTCTTAACTCTTAATXXXXXXXXXXTTTTCCTGTTTGTATGAATTTTGTTTTTGTCGTAGAGGAGAGAATAGAGTGTTGCATTATGAGGGGAGAACTACAAATGG

CTCTTTCACCCAACCAATTCTTCACTTCAACCAAGCCAGGTATTCAAATTCTTTCCTATTTCCTCCTCTTAACTCTTAATXXXXXXXXXXTTTTCCTGTTTGTATGAATTTTGTTTTTGTCGTAGAGGAGAGAATAGAGTGTTGCATTATGAGGGGAGAACTACAAATGG

>Marker172678

AAACGCACGTTAACAATAGTTAAATAAAATAGAATATTAAATAAAGCGAGGGGACAAAAAGCCAAAATTTAGGGCACTGCXXXXXXXXXXTATATAGTAGTAGTTTGGATTTAATTAGTATTCCACTTTATGCTTCTAGAGGGACCTTTTTCAATTGTATTAGCCAATAT

AAACGCACGTTAACAATAGTTAAATAAAATAGAATATTAAATAAAGCGAGGGGACAAAAAGCCAAAATTTAGGGCACTGCXXXXXXXXXXTATATAGTAGTAGTTTGGATTTAATTAGTATTCCACTTTATGCTTCTAGAGCGACCTTTTTCAATTGTATTAGCCAATAT

>Marker172797

ATTATAGAACACACCCAAATGTGGTATTGAAGTTAATATCAATCAATTAAAACTAAAACCAACTATAGAAATGACCTGTAXXXXXXXXXXTCACCTAATGATCCTCTTGATTGATTACTACTGCAAATACAACATACAATGTTTACCTGCAAAATTTGACAAGAATCAAT

ATTATAGAACACACCCAAATGTGGTATTGAAGTTAATATCAATCAATTAAAACTAAAACCAACTATAGAAATGACCCGTAXXXXXXXXXXTCACCTAATGATCCTCTTGATTGATTACTACTGCAAATACAACATACAATGTTTACCTGCAAAATTTGACAAGAATCAAT

>Marker172846

CAACAAGTGGAAACTAATTTTAATTGGGGAGGAAGCAACAATGCAGGGGCTTGAACGCAGGACCTCCCTGGACCACCTGCXXXXXXXXXXATTCAAAGGAGTGAATAATAAAGAATAATCACCAAAAGCCTTAGTAACGGAAGCCTAAAGGGAACATGGGACCTAGCAAA

CAACAAGTGGAAACTAATTTTAATTGGGGAGGAAGCGACAATGCAGGGGCTTGAACGCAGGACCTCCCTGGACCACCTGCXXXXXXXXXXATTCAAAGGAGTGAATAATAAAGAATAATCACCAAAAGCCTTAGTAACGGAAGCCTAAAGGGAACATGGGACCTAGCAAA

>Marker173034

AAAGGTGAATTAACAAGTTGGCTCAATAGAGATTCGGTTGATCGTCAAATTGTGAATATAATTTTATAGAGTTGGTTGTAXXXXXXXXXXTTCGTCATCAAGATTCCAGCTCCCTCTCTAATCCACTTCACAAAAGTAGAGACAAAAACCAAGATTAAAAGATATCACTA

AAAGGTGAATTGACAAGTTGGCTCAATAGAGATTCGGTTGATCGTCAAATTGTGAATATAATTTTATAGAGTTGGTTGTAXXXXXXXXXXTTCGTCATCAAGATTCCAGCTCCCTCTCTAATCCACTTCACAAAAGTAGAGACAAAAACCAAGATTAAAAGATATCACTA

>Marker173058

CCTCGTCGAAAGCCTTTCGTGCAGCTGATACTGCACGGTTGACATCCTCAGCATCACCCTCGGCAACATGAGCAATCACTXXXXXXXXXXAAGAAAGCTAAAACAGTAACAGCACAAACAATTCTGTTAGATGAAGAAAAAAAATTCACCGGATGCAGAATCAACAAACT

CCTCGTCGAAAGCCTTTCGTGCAGCTGATACTGCACGGTTGACATCCTCAGCATCACCCTCGGCAACATGAGCAATCACTXXXXXXXXXXAAGAAAGCTAAAACAGTAACAGCATAAACAATTCTGTTAGATGAAGAAAAAAAATTCACCGGATGCAGAATCAACAAACT

>Marker173651

TGTGTTTAGCTTCGGTGATTCATGCTGTGGGTGATGGGTCTGGGGCTAAAGGGGAGCTTCTATCCCATGAATGTTTTATTXXXXXXXXXXGAAAAAACATAAGGGCGACTGTGATAAGATTGCTACAAAATTAAGGAAGCTTGATAGTTCTATGATTATGGAACGAAGAA

TGTGTTTAGCTTCGGTGATTCATGCTGTGGGTGATGGGTCTGGGGCTAAAGGGGAGCTTCTATCCCATGCATGTTTTATTXXXXXXXXXXGAAAAAACATAAGGGCGACTGTGATAAGATTGCTACAAAATTAAGGAAGCTTGATAGTTCTATGATTATGGAGCGAAGAA

>Marker173924

AGACAAGGCAGCATGATAAAAAGGTAATAGTTGCTCTATAAAAGAAAACAGTAATGGAAAAGGCGTTTTAAAACACAAACXXXXXXXXXXACAGGAATAAGCAGAAGGAAGGTCGAAGGAACTGCAGTTCAAGCAGAGGATGCAGCTTACCTTAACGGTGTCAAAAGGGT

AGACAAGGCAGCGTGATAAAAAGGTAATAGTTGCTCTATAAAAGAAAACAGTAATGGAAAAGGCATTTTAAAACACAAACXXXXXXXXXXACAGGAATAAGCAGAAGGAAGGTCGAAGGAACTGCAGTTCAAGCAGAGGATGCAGCTTACCTTAACGGTGTCAAAAGGGT

>Marker174325

CCTATATACCGTATACCCACGGGTCCGACCTTAAAAGATTTGGATGCGTGCTTTTTAACATATCACTCCCTTTCCACACCXXXXXXXXXXTTTGTTCAAGAGTTTGAGTAACTTTTCAAAGTTTGTAGGACCTGAATTTCACATTAAATGTTATTTCAAGAATACAAAAT

CCTATATACCGTATACCCACGGGTCCGACCTTAAAAGATTTGGATGCGTGCTTTTTAACATATCACTCCCTTTCCACACCXXXXXXXXXXTTTGTTCAAAAGTTTGAGTAACTTTTCAGAGTTTGTAGGACCTGAATTTCACATTAAATGTTATTTCAAGAATACAAAAT

>Marker174528

ATGCTATGAGCATAAACATTATTTTAATTTCATGTTTGGATTTGGGCTATTTTAGATTTGTTATTGAGAATAACAACCTTXXXXXXXXXXGATTTCGAATCATTTGAATCAGGTCAAACTTGTTTGTTAAGGAAAATGACAAAGAGTCCTTTTTGTCAAAAGGGTGAAAA

CTGCTATGAGCATAAACATTATTTTAATTTCATGTTTGGATTTGGGCGATTTTAGATTTGTTATTGAGAATAACAACCTTXXXXXXXXXXGATTTTGAATCATTTGAATCAGGTCAAACTTGTTTGTTAAGGAAAATGACAAAGAGTCCTTTTTGTCAAAAGGGTGAAAA

>Marker174621

TTTTCTGTAGTTAGTATGGTTAGAAAGAAATTAAAGGATTTTTGGAACCAAGGAAAGGGTCTATGTGGACTTTTTTGACAXXXXXXXXXXAAATATGAGTTTTTGGGCATTTACTTGGACATGTCCTCCATGGACCACTGCCTCTTTCGTTGGCTGCAAAATTGGTGTTT

TTTTCTGTAGTTAGTATGGTTAGAAAGAAATCAAAGGATTTTTGGAACCAAGGAAAGGGTCTATGTGGACTTTTTTGACAXXXXXXXXXXAAATATGAGTTTTTGGGCATTTACTTGGACATCTCCTCCATGGACCACTGCCTCTTTCGTTGGCTGCAAAATTGGTTTTT

>Marker174867

TTTAGACGACAGCAACTTACTGATAAATCTGATGTTTACTCATTTGGGGTTGTTCTTTTTGAGGTTCTATGTGCAAGACAXXXXXXXXXXGCTGGATTTAGGATAGCCTAACTTATGTTTAGAGGAAGAGCTGTGACACCTGAGATTAATTGGAGTAACTAAGGGTTTAG

TTTAGACGACAACAACTTACTGATAAATCTGATGTTTACTCATTTGGGGTTGTTCTTTTTGAGGTTCTATGTGCAAGACAXXXXXXXXXXGCTGGATTTAGGATAGCCTAACTTATGTTTAGAGGAAGAGCTGTGACACCTGAGATTAATTGGAGTAACTAAGGGTTTAG

>Marker175001

ATCACCTAGAAAAAACCCAACAGAAATGTGAGACGTTCGTTCTCCATTTCATAAAATATCAAACTTGCAAAACTATGAGGXXXXXXXXXXAAACTTCAAAAAAATTTATGTATTTGTCCAAATCTCTGTGCTAAAAGTATACAAAGAAGCCAGTTTCTGCCTGCCGTTTG

ATCACCTAGAAAAAACCCAACAGAAATGTGAGACGTTCGTTCTCCATTTCATAAAATATCAAACTTGCAAAACTATGAGGXXXXXXXXXXAAACTTCAAAAAATTTTATGTATTTGTCCAAATCTCTGTGCTAAAAGTATACAAAGAAGCCAGTTTCTGCCTGCCGTTTG

>Marker175190

TATTAGTGCTTGTAGCATCCTGGGGATTTATCACTTCGAGGAACTTGTGAAACGTGGGGCAGTTCCATTTAAAGGTATTAXXXXXXXXXXTCTTCTTCTCCCACGTGTTCTCTGTTTTCTCTTCTCCTCCCACACTGACACTGCCCAACCAGTCACTACACTGTTCACTG

TATTAGTGCTTGTAGCATCCTGGGGATTTATCACTTCGACGAACTTGTGAAACGTGGGGCAGTTCCATTTAAAGGTATTAXXXXXXXXXXTCTTCTTCTCCCACGTGTTCTCTGTTTTCTCTTCCCCTCCCACACTGACACTGCCCAACCAGTCACTACACTGTTCACTG

>Marker175606

TATTCATTCTTTTATAACCATGCAAATGGGATCGTAATATTTTAGATTTGATCTGTATATTTTTCATTGTATATAAATTTXXXXXXXXXXTAAAACTCTAAAATATATAGACAAAGGTGATATTTGTTTTAAGAGTGTAGGTTTGAAATTTGAATCATTTCAAATATTCA

TATTCATTCTTTTATAACCATGCAAATGGGATCGTAATATTTTAGATTTGATCTGTATATTTTTCATTGTCTATAAATTTXXXXXXXXXXTAAAACTCTAAAATATATAGACAAAGGTGATATTTGTTTTAAGAGTGTAGGTTTGAAATTTGAATCATTTCAAATATTCA

>Marker176037

CCTACTCTCATCAAACTGTGAATAACCATATTCATTTTTCTGTTGGTGTATACATGGAAAAAAGATGTGATGAACTGTGTXXXXXXXXXXTTTGAAATGATGTAGAATTGATTAATAACATTGGTTATATATCTTTAGGAAAAGTTGACGAAACAAACTTTAATGCAATT

CCTACTCTCATCAAACTGTGAATAACCATATTCATTTTTCTGTTGGTGTATAAATGGAAAAAAGATGTGATGAACTGTGTXXXXXXXXXXTTCGAAATGATGTAGAATTGATTAATAACATTGGTTATATATCTTTAGGAAAAGTTGACAAAACAAACTTTAATGCAATT

>Marker176062

TCTTAGATTGACGGTGTAACAAGTGAGTCATGGAAACTCCAACCTCCATAACTATCATTGTATTAAAGAAAGAAAAGACAXXXXXXXXXXCGAGAGGTGAAGTTTAAATAAAACGGTCACTATTGCTCGACTATTTATAAGGTATGAAGGCTGATGAATATTGGAACATA

TCTTAGATTGACGGTGCAACAAGTGAGTCATGGAAACTCCAACCTCCATAACTATCATTGTATTAAAGAAAGAAAAGACAXXXXXXXXXXCGAGAGGTGAAGTTTAAATAAAACGGTCACTATTGCTCGACTATTTATAAGGTATGAAGGCTGATGAATATTGGAACATA

>Marker176271

AAGAAGGAAAAAGCCAAAAATCATTCAAAAGTAATGGGTGCCAAATTGTATATAAATCCTCAAGTCAATGCATTCAGACAXXXXXXXXXXTCTTCCAAAACCATGGAGAAGAATTGCTTCTTTTCTTCTCTAGCATTCTTTTCATACATTGTAATTGCCACTATTTCCAT

AAGAAGGAAAAAACCAAAAATCATTCAAAAGTAATGGGTGCCAAATTGTATATAAATCCTCAAGTCAATGCATTCAGACAXXXXXXXXXXTCTTCCAAAACCATGGAGAAGAATTGCTTCTTTTCTTCTCTAGCATTCTTTTCATACATTGTAATTGCCACTATTTCCAT

>Marker176405

TAACCAACGGGAATGAACTAAGGAAATTCCTCATGGGATGCTGATAAATTGAATTCTAGGTCTATGCGTGGGAATGATTCXXXXXXXXXXTAAGGCGTTGAGACCACGTAAAGATGAGTAAGGCGAGTTGCTAGGGGTTTAAGTGAAGCGTAGTTGTGCTACGTGTTGAA

TAGCCAACGGAAATGAACTAAGGAAATTCCTCATGGGATGCTGATAAATTGAATGTTAGGTCTATGCGTGGGAATGATTCXXXXXXXXXXTAAGGCGTTGAGACCACGTAAAGATGAGTAAGGCGAGTTGCTAGGGGTTTAAGTGAAGCGTAGTTGTGCTACGTGTTGAA

>Marker176490

CCAGTCAATATCATCGACAATCAACGTGATAACTCCGTAAATGAGCTAGGTTTTCTAGGTTAAAACTCTCCAGATAAAAAXXXXXXXXXXAAAAGACAGAATGCATCAAAATTCTGCAGTGCCATGACACTCTCATTAGTGTCGTATTGCCAGTCCAATACCCAATCATA

CCAATCAGTATCATCGACAATCAACGTGATAACTCCGTAAATGAGCTAGGTTTTCTAGGTTAAAACTCTCCAGATAAAAAXXXXXXXXXXAAAAGACAGAATGCATCAAAATTCTGCAGTGCCATGACACTCTCATTAGTGTCGTATTGCCAGTCCAATACCCAATCATA

>Marker176801

AGGACTTACAAATTTCAATGACTCTAATTTTAGTCCCTTGTTGTTCTTCTACTTTCATTCAAATTAAACTTGTATATTGGXXXXXXXXXXTTTTTTTGGCTCGTCAGGATAATGCAGTTGTCATACATGCAATTGTTGGATGGAAATCGTCTATTGGTCGATGGTCTCGA

AGGACTTACAAATTTCAATGACTCTAATTTTAGTCCCTTGTTGTTCTTCTACTTTCATTCAAATTAAACTTGTATATTGGXXXXXXXXXXTTTTTTTGGCTTGTCAGGATAATGCAGTTGTCATACATGCAATTGTTGGATGGAAATCGTCTATTGGTCGATGGTCTCGA

>Marker176933

ATTGTATCTGGGTTTGGTGGCAGTTTGATGTCGGCTCTGTTTATCCAGTCGGGTATCTCAGTAGGTGCTTCGGGTGCTCTXXXXXXXXXXTTGGAGTGCTTCCACATGTTGATAATTTTGCTCATATTGGAGGCTTTATCTCAGGTTTTCTTCTGGGGTTTGTGTTTTTG

ATTGTATCTGGGTTTGGTGGCAGTTTGATGTCGGCTCTGTTTATCCAGTCGGGTATCTCAGTAGGTGCTTCGGGTGCTCTXXXXXXXXXXTTGGAGTGCTTCCACATGTTGATAATTTTGCTCATATTGGAGGATTTATCTCAGGTTTTCTTCTGGGGTTTGTGTTTTTG

>Marker177273

TTATGTCCAGCCTGAAGCCTCAAGACCCTGAATTTCCAAAGCAGCATAACCAATTCGTAAATACAAAGACTTCAAATCAAXXXXXXXXXXCAAATGAAGAAGAAAAGGAAGAGGACAATACCCACGAATGGTGCGCCAAAGGATCTTGGCGGGAGCACGGAAGTGAATGG

TTATGTCCAGCCTGAAGCCTCAAGACCCTGAATTTCCAAACCAGCATAACCAATTCGTAAATACAAAGACTTCAAATCAAXXXXXXXXXXCAAATGAAGAAGAAAAGGAAGAGGACAATACCCACGAATGGTGCGCCAAAGGATCTTGGCGGGAGCACGGAAGTGAATGG

>Marker177546

ATCTGTAGATGTTCCAGGCAGACAGTCTTCATTATTATCGCTTAAACATTCTAGCATATGGGATGAAAATGTTGCTGTTAXXXXXXXXXXAGGTGATGTGGATGCAGCATACGTGGAAGCCTTACGTTCTGGTGATGAAGTTGTTCTACTTGAACTTCTTGATCAAACAG

ATCTGTAGATGTTCCAGGTAGACAGTCTTCATTATTATCGCTTAAACATTCTAGCATATGGGATGAAAATGTTGCTGTTAXXXXXXXXXXAGGTGATGTGGATGCAGCATACGTGGAAGCCTTACGTTCTGGTGATGAAGTTGTTCTACTTGAACTTCTTGATCAAACAG

>Marker178311

AAAAGAATTACACAAAAAGCAGAAAAAATTGAACCTAGAGATCAGGAGATGCACCCGAGCATCTTAACTTGGTTGATATCXXXXXXXXXXTCTCTCTCCTCCTTCACCCTCCCTTCATTATCATTGTCAGCCTATTACCGTCTTTGGTAGACATTGCTATATATCGACTT

AAAAGAATTACACAAAAAGCAGAAAAAATTGAACCTAGAGATCAGGAGATGCACCCGAGCATCTTAACTAGGTTGATATCXXXXXXXXXXTCTCTCTCCTCCTTCACCCTCCCTTCATTATCATTGTCAGCCTATTACCGTCTTTGGTAGACATTGCTATATATCGACTT

>Marker178738

TCATTTGTATACAGGGACACTCTTATGTGCCCCAATATGATGCAAGAAAAATAGGGGTCCATTGGGTAATTGTGTAATATXXXXXXXXXXCAACATGTCTTCTCTATTGCGGTCTCAGAACCTTGGGCTGAAAGCCATCCCCTAGCTCTTTCCAATCCAAGTTCCCCTTG

TCATTTGTATACAGGGACACTCTTATGTGCCCCAATATGATGCAAGAAAAATAGGGGTCCATTGGGTAATTGTGTAATATXXXXXXXXXXCAAAATGTCTTCTCTATTGCGGACTCAGAACCTTGGGCTGAAAGCCATCCCCTAGCTCTTTCCAATCCAAGTTCCCCTTG

>Marker179007

GAAGATGTGCATATGTTTCTTTCTAGATTCTCATGTTTCTCATCTTTTCCTTTGGTTGTGTTTGGAAGTTGGCACTTGGCXXXXXXXXXXAAATATAAAAGAGAGCTATCTATCCCCCAACCCAGTCCACCTCGTTTCCTTTTCCCTTCCCACCTTCATCGCGCCCCCCA

GAAGATGTGCATATGTTTCTTTCTAGATTCTCATGTTTCTCATCTTTTCCTTTGGTTGTGTTTGGAAGTTGGCACTTGGCXXXXXXXXXXAAATATAAAAGAGAGCTATCTATTCCCCAACCCAGTCCACCTCGTTTCCTTTTCCCTTTCCACCTTCATCGCGCCCCCCA

>Marker179554

ATTTAGTCATCTCTAATCATTGGAATTTATGTTAACTCCTTCAGGAACAACCAATAAAATTGAAAAGGTCGACAAAAAGCXXXXXXXXXXAAGGGATAGTTGCTAGCATTAACTTTCCTGCAAAGGGCATTAGTGTCCAACATAAACCCCCAACCTCATTTATCAACAAA

ATTTAGTCATCTCTAATCATTGGAATTTATGTTAACTCCTTCAGGAACAACCAATAAAATTGAAAAGGTCGACAAAAAGCXXXXXXXXXXAAGGGATAGTTGCTAGCATTAACTTTCCTGCAAAGAGCATTAGTGTCCAACATAAACCCCCAACCTCATTTATCAACAAA

>Marker179831

TATTTCTTGGTTGTGTGGGGAAACTTAGGGCTGATAATATTCATCAAAAGTTTCAAGTTTGTGTTTGAATTTGTGTTCTAXXXXXXXXXXTGGTTATTATAAATCTCATACGTTGGAACTTGAAACTTTAATTCTTTGTTCAACTACTAACTTTTTGTTATCCGTAAAGG

TATTTCTTGGTTGTGTGGGGAAACTTAGGGCTGATAATATTCATCAAAAGTTTTGAGTTTGTGTTTGAATTTGTGTTCTAXXXXXXXXXXTGGTTATTATAAATCTCATACGTTGGAACTTGAAACTTTAATTCTTTGTTCAACTACTAACTTTTTGTTATCTGTAAAGG

>Marker179961

CAGTGATAGCTGCTATTACTATTAATCACTTCTATCACTAATTGTATTAGTTGCACTTATTTGTATCAATGATAAATACTXXXXXXXXXXAGAAATTGAGTGATGAACATTTTCTATATATAAATATTTAAAAGTTGTGTTATAGTTTTTTTTAGATTATTTTGTTGATT

CAGTGATAGCTGCTATTACTATTAATCACTTCTATCACTAATTGTATTAGTTGCACTTATTTGTATCAATGATAAATACTXXXXXXXXXXAGAAATTGAGCGATGAACATTTTCTATATATAAATATTTAAAAGTTGTGTTATAGTTTTTTTTAGATTATTTTGTTGATT

>Marker179999

GCCGTTCGACCACTCCCCACCGACGCCCGCGTTGGTCCGATAAGTTGCGCGCCGCCTAACGATCTTGCCATCAGCCCTCTXXXXXXXXXXAATTGTTGTTAGTTACTGTCCTGCAAGTTCGGGACCGGTTTCGGCAAGAGGTGGATAAGTATTTGTCTTTGAGGTTTGAG

GCCGTTCGACCACTCCCCACCGACGCCCGCGTTGGTCCGATAAGTTGTGCGCCGCCTAACGATCTTGCCATCAGCCCTCTXXXXXXXXXXAATTGTTGTTAGTTACTGTCCTGCAAGTTCGGGACCGGTTTCGGCAAGATGTGGATAAGTATTTGTCTTTGAGGTTTGAG

>Marker180043

TAAAATTTACCCATTATAATAATTTAATTTTGAATTTTGTTGATGAATCGTTTAATTACTCTAATTTAAAATTTTATAATXXXXXXXXXXGAAAAAAAAGTAGGGATGAGAAAGCGAAAGAGAAAGAGGCGGGAATTGTCTTATGCGAGGAAGTGAGTCAAAAGAAATGA

TAAAATTTACCCATTATAATAATTTAGTTTTGAATTTTGTTGATGAATCGTTTAATTACTCTAATTTAAAAATTTATAATXXXXXXXXXXGAAAAAAAAGTAGGGATGAGAAAGCGAAAGAGAAAGAGGCGGGAATTGTCTTATGCGAGGAAGTGAGTCAAAAGAAATGA

>Marker180129

CAAAATTGCAACAAGAAAATCAAGCTCTTGATCAGTTAGATGCTTTTCGTTCTGTAACTCAAATTCGAGACTCAATGTATXXXXXXXXXXAAAAATTAACACAAAATAACTACAAAACATCATCCAAATCCTGGGAGTAGAAAAACTGTCTATTTAAAATAAAGCGATTT

CAAAATTGCAACAAGAAAATCAAGCTCTTGATCAGTTAGATGCTTTTCGTTCTGTAACTCAAATTCGAGACTCAATGTATXXXXXXXXXXAAAAATTAACACAAAATAACTACAAAACATCATCCAAATCCTGGGAGTAGAAAAACTGTCTATTTAAAATTAAGCGAATT

>Marker180326

ATATGAGAGCCTCCACTGTTTTAGGAGCCAATGAGCAACTCATGAATCAACATGTCCTCCACTCAGGTGCTACAATCAAAXXXXXXXXXXCATAGACAAAATCTTCACCTACCTTTTCATTTAGATCAAAATCAATAATCGCATCCAAGACCGAGACTGTTTGTGTAGTA

ATATGAGAGCCTCCACTGTTTTAGGAGCCAATGAGCAACTCATGAATCAACATGTCCTCCACTCAGGTGCTACAATCAAAXXXXXXXXXXCATAGACAAAATCTTCACCTACCTTTTCATTTAGATCAAAATCAATAATCGCATCCAAGACCGAGACTGTTTGCGTAGTA

>Marker180328

TTACCTTTTTTCCCATGAGTGATTATACACATAGACATTTTAAAAAATAGCAAAATAAACTAAGATATTTACAACATACAXXXXXXXXXXAACGACGTGATACTACCAAATACATATCATGTGTGACTAAATAAAGCACATTTTTCCGAATGAGTGTAAGATACTTTATG

TTACCTTTTTTTCCATGAGTGATTATACACATAGACATTTTAAAAAATAGCAAAATAAACTAAGATATTTACAACATACAXXXXXXXXXXAACGACGTGATACTACCAAATACATATCATGTGTGACTAAATAAAGCACATTTTTCCGAATGAGTGTAAGATACTTTATG

>Marker180536

AGCTTAAGCCAAGAGTTCCTTGAACTTGTCAATGATATTGACACATTATTGGCTTGTCACGAGGGATTTCTTTTGGGACCXXXXXXXXXXTGACAACACAGAGGAAGAAGCAAGTTTGCTTCGTGATTATGGTAGTGATTATTATCATTTATCATTGAGATTAGGCAATT

AGCTTAAGCCAAGAGTTCCTTGAACTTGTCAATGATATTGACACATTATTGGCTTGTCACGAGGGATTTCTTTTGGGACCXXXXXXXXXXTGACAACACAGAGGAAGAAGCAAGTTTGCTTCGTGATTATGGTAGTGATTATTATAATTTATCATTGAGATTAGGCAATT

>Marker180553

TACTCTATTTCTGTGTTTTTCTGCATGGGACCTTTCAACCAATTTTCCGCCTCTAAATTCTTTTTTATTTTACATCTTTCXXXXXXXXXXAACAATTTTTGTTGTAATAATGATGACAATATCAGTAATCCAATTAAGCTTAATTATTTTCAAGTATAATTGGATTGAGT

TACTCTATTTCTGTGTTTTTCTGCATGGGACCTTTCAACCAATTTTCCGCCTCTAAATTCTTTTTTATTTTACATCTTTCXXXXXXXXXXAAGAATTTTTGTTGTAATAATGATGACAATATCAGTCCTCCAATTAAGCTTAATTATTTTCAAGTATAATTGGATTGGGT

>Marker180621

TCTTCTTCATATATATCTTCAAACTTCATTGTGAAACGTTTATTCTAACTAAACTCAAATATTTAAACACAACTTACTTAXXXXXXXXXXTCCATATCTTCATTTGAATACCAAATTAAGATTAAAAGATACTATTTTAATCTTGATAATTTAGAATGATTTCGATTTTA

TCTTCTTCATATATATCTTCAAACTTCATTGTGAAACGTTTATTCTAACTAAACTCAAATATTTAAACACAACTTACTTAXXXXXXXXXXTCCATATCTTCATTTGAATACCAAATTAAGATTAAAAGATACTATTTTAATCTCGATAATTTAGAATGATTTCGATTTTA

>Marker181707

TTGTTGTAATCTTACTCTCTGTTTTAATAATTTGAGAGAATTGGTGCATGATTATTCATAATTCATCTTTATTTGCGATGXXXXXXXXXXGTTTTGCTGCTAGTAGGAGGTAGGAGAATTGTTGTGTGTCCTAAAAGACTAAACTGATGGCTTATAATTTTAACTTCAAT

TTGTTGTAACCTTACTCTCTGTTTTAATAATTTGAGAGAATTGGTGCATGATTATTCATAATTCATCTTTATTTGCGATGXXXXXXXXXXGTTTTGCTGCTAGTAGGAGGTAGGAGAATTGTTGTGTGTCTTAAAAGACTAAACTGATGGCTTATAATTTTAACTTCAAT

>Marker181722

TCGGGGACCCCTTTGGAGAAGCCGGAAAGCAATTGGTAAAGAAGCACTTTTCGTTATTCAAGGATTGAAGAGATTCAAGGXXXXXXXXXXTTTTGTTGCTCTTTCTGGATCCTCACATTCATTTTAGATTTTACGTAATGGGTCGTCTCAGAAATTGATAATTGGCTTCT

TCGGGGACCCCTTTGGAGAAGCCGGAAAGCAATTGGTAAAGAAGCACTTTTCGTTATTCAAGGATTGAAGAGATTCAAGGXXXXXXXXXXTTTTGCTGCTCTTTCTGGATCTTCACATTCATTTTAGATTTTACGTAATGGGTCGTCTCAGAAATTGATAATTGGCTTCT

>Marker182093

CCTTCACCTTTAAATTCAATGACACCAAAACCGAGTTCGCCACTACCATCCAAATGAAAATCCTAGAGAACCTTTGAGAAXXXXXXXXXXTTTACCTTTACACAGAAGGGGATTGAAATAATGAGCAGCAGAGAGATGGTTTAGTTTGGCATTTTACTCTGAGGGAATTT

CCTTCACCTTTAAATTCAATGACACCAAAACCGAGTTCGCCACTACCATCCAAATGAAAATCCTAGAGAACCTTTGAGAAXXXXXXXXXXTTTACCTTTACACAGAAGGGGATTGAAATAATGAGCAGCAGAGAGATGGTTTAGTTTGGCATTTTACTCCGAGGGAATTT

>Marker182400

TACAGCTACTAACATGAAGATGAAGAGAGTGGAGATTGAGAGGAGGTTTGAAAGGATCTCTAGAGTTGTGAAGAAGGCGAXXXXXXXXXXCATACCTGTTCTTTCATTGACTTTGGCGAACCAGGGGGAGACCATGTGAGTCCTGGCAATGTGTGTGAGGTATCGCGCTT

TACAGCTACTAACATGAAGATGAAGAGAGTGGAGATTGAGAGGAGGTTTGAAAGGATCTCTAGAGTTGTGAAGAAGGCGAXXXXXXXXXXCATACCTGTTCTTTCATTAACTTTGGCGAACCAGGGGGAGACCATGTGAGTCCTGGCAATGTGTGTGAGGTATCGCGCTT

>Marker182822

ACCATCGCTTCTGCAGTCAAATATGTATGTCCTGCCACCCACTAACCTTAGCTATCATCCCGATATCAGGAATCCTCAAAXXXXXXXXXXAAGCACAATGGAAAACAGTTCTTGTCTATGAGCATTGCAAAAAACAATGGCATACCAAGGAACAGCCTTTGAAGTTACAT

ACCATCGCTTCTGCAGTCAAATATGTATGTCCTGCCACCCACTAACCTTAGCTATCATCCTGATATCAGGAATCCTCAAAXXXXXXXXXXAAGCACAATGGAAAACAGTTCTTGTCTATGAGCATTGCAAAAAACAATGGCATACCAAGGAACAGCCTTTGAAGTTACAT

>Marker182844

AAAAATCTGACCAGGGGGAGGGAGGGAAGGTGAAAGAGATTCAAGAGTAGCACCTAGTTTGAATCTTTATGAATCTAATTXXXXXXXXXXGCCAGCCACCAGCGGCATGCATAGTATTGATATCACCATTCTTGATCCTGTCATTGGGAATGCGTAATCAAAATGACACA

AAAAATCTGACCAGGGGGAGGGAGGGAAGGTGAAAGAGATTCAAGAGTAGCACCTAGTTTGAATCTTTATGAATCTAATTXXXXXXXXXXGCCAGCCACCAGCGGCATGCATAGTATTGATATCACCATTCTTGATCCTGTCATTGGGAATGCGTAATCAAAATGATACA

>Marker183050

CACTAAATTAACATAGTAGATGGTATTTTAGTTTATATATATTAATTTATTTTTAAACAAATAAAATTGATAAATAAAAGXXXXXXXXXXCCACAAGCCAATAAGGACGAGTCGCGTAATTAGTTAAGCCTGTGATAGGGGATGGGAGTGCCCACAACACGCGACAATAT

CATTAAATTAACATAGTAGATGGTATTTTAGTTTATATATATTAATTTATTTTTAAACAAATAAAATTGATAAATAAAAGXXXXXXXXXXCCACAAGCCAATAAGGACGAGTCGCGTAATTAGTTAAGCCTGTGATAGGGGATGGGAGTGCCCACAACACGCGACAATAT

>Marker183108

TTAATTAGTTAAATTAATGGCTCAATCTTGTAGACCATACTCGAACCCAATCTCCATATAAAGGCATTATAACATAAGCTXXXXXXXXXXTATTATGTTTAAAAAAGCAGTATATGATATCCTAAATTTATGAATGGAATAAAAAGAAAAGAAAGGAAGAAAAATGAAGT

TTGATTAGTTAAATTAATGGCTCAATCTTGTAGACCATACTCGAACCCAATCTCCATATAAAGGCATTATAACATAAGCTXXXXXXXXXXTATTATGTTTAAAAAAGCAGTATATGATATCCTAAATTTATGAATGGAATAAAAAGAAAAGAAAGGAAGAAAAATGAAGT

>Marker183177

CAACATAAATACACCCAACATTAGGAAATGCACAATCCATATCTGAAAAATGAGAAATCATTTGAACGATTAAGTTGAAAXXXXXXXXXXCTCATGAATTTTAACCATAGGCTCGTCAACCTTTCCATTAGTAGCGGAGTTAACCAAATAAGCACATAGGGCTATAGAGA

CAACATAAATACACCCAACATTAGGAAATGCACAATCCATATCTGAAAAATGAGAAATCATTTGAACGATTAAGTTGAAAXXXXXXXXXXCTCATGAATTTTAACCATAGGCTCATCAACCTTTCCATTAGTAGCGGAGTTAACCAAATAAGCACATAGGGCTATAGAGA

>Marker183358

AAACCTCCCATGGCAGATTGTCACAATGAGGTCGATGGCTTTACTGAGCAACAAAAGGAGTTGAATGAGAAACAGGCACAXXXXXXXXXXTGAAGCTCTGTCCCTTAATCTCATATTGAATGTGTTTCGTTCCTTCTTCTTTACTGGAATTCTCATTGTAGAATCTCTCA

AAACCTCCCATGGCAGATTGTCACAATGAGGTCAATGGCTTTACTGAGCAACAAAAGGAGTTGAATGAGAAACAGGCACAXXXXXXXXXXTGAAGCTCTGTCCCTTAATCTCATATTGAATGTGTTTCGTTCCTTCTTCTTTACTGGAATTCTCATTGTAGAATCTCTCA

>Marker184156

AGCAATGATCATCAGAGGTGATGTCCGAAAGTTGGTCGACCAACTTCCCAGCTCTTGAAACAAATACCCCTAGAATTAAAXXXXXXXXXXTGAAGATGCTTGTGTTCCTTAATTCTTCTGTTTCCTAAAAAGATAATATTTATCCTAGTTGCTAGATCCAATCTTTTTAA

AGCAATGATCATCAGAGGTGATGTCCGAAAGTTGGTCGACCAACTTCCCAGCTCTTGAAACAAATACCCCTAGAATTAAAXXXXXXXXXXTGAAGATGCTTGTGTTCCTTAATTCTTCTGTTTCCTAAAAAGATAATATTTATCCTAGTTGCTAGATCCGATCTTTTTAA

>Marker184475

TTAAATTAATTCAAACAACAATTTAAATTATGTTTCTACCTTGTAAAATAATTTTAAATATTAAAAAAAAAAAAAAAGTTXXXXXXXXXXATGATGAAAAGTATAAATAATTGAATTTGGAACTAATTAAAAATTGACATGTGTCCCAATTAAAATTGAAAACGTAAATT

TTAAATTAATTCAAACAACAATTTAAATTATGTTTCTACCTTGTAAAATAATTTTAAATATTAAAAAAAAAAAAAAGTTGXXXXXXXXXXATGATGAAAAGTATAAATAATTGAATTTGGAACTAATTAAAAATTGACATGTGTCCCAATTAAAATTGAAAACGTAAATT

>Marker184610

AATCATCCATGTTCCCAAGATTGCCAAGCTCTCCCAAGGTTTTGGGGTCCGAAATCTCGGAAACACCTCCCAAAAAATACXXXXXXXXXXGTTACACCTTGTTATAAAATAAGGGAGGGTAAGTGAGAGAGGGAGGTTATTCTGTGGAGGGATTTAGGGCTTGGGTGAGA

AATCATCCATGTTCCCAAGATTGCCAAGCTCTCCCAAGGTTTTGGGGTCCGAAATCTCGGAAACACCTTCCAAAAAATACXXXXXXXXXXGTTACACCTTGTTATAAAATAAGGGAGGGTAAGTGAGAGAGGGAGGTTATTCTGTGGAGGGATTTAGGGCTTGGGTGAGA

>Marker184985

AATCTATAAAGATCATAACAAAGAGAAACAGAGTGGGAGCAATAATAATACTAATGGCATCTCAATGGCTTTTCTCTTTTXXXXXXXXXXTCATTTATCCCATATGTATCATCTTTCATTTAAAATGAGATATATAAAATATGAGCTTTCTTTTGTTTGAGTTAGTATAT

AATCTATAAAGATCATAACAAAGAGAAACAGAGTGGGAGCAATAATAATACTAATGGCATCTCAATGGCTTTTCTCTTTTXXXXXXXXXXTCATTTATCCCATATGTATCACCTTTCATTTAAAATGAGATATATAAAATATGAGTTTTCTTTCGTTTGAGTTAGTATAT

>Marker185295

ACCCCTTCAAATGAGCAAAAGAACAACTGTCGGCTAGCATTATACCTGCAGTTTTTTGGAAGTTTTGGGAGGGATGAAATXXXXXXXXXXGGGCTTCTTTTCTACATCTTTGGATATTTCATTCAATGAATGGAATTTTCTTATAAAAAGGTCTTTTGGTTATGGTTCAT

ACCCATTCAAATGAGCAAAAGAACAACTGTCGGCTAGCATTATACCTGCAGTTTTTTGAAAGTTTTGGGAGGGATGAAATXXXXXXXXXXGGGCTTCTTTTCTACATCTTTGGATATTTCATTCAATGAATGGAATTTTCTTATAAAAAGGTCTTTTGGTTATGGTTCAT

>Marker185629

AAGTCGACCGTTGGTTGGTTGACGATAGGTTTAAGCATTTGAAGATTTTAAAAAATCCAAACTGACACCAAATGAATCTTXXXXXXXXXXACAACGATATATCTTAGAGCAACCTCGATTGTAAGGATTAGCCGGTGGCGGAGCACAACTTCCACTTCTGGTGTAAGGTT

AAGTCGACCGTTGGTTGGTTGACGATAAGTTTAAGCATTTGAAGATTTTAAAAAATTCAAACTGACACCAAATGAATCTTXXXXXXXXXXACAACGATATATCTTAGAGCAACCTCGATTGTAAGGATTAGCCGGTGGCGGAGCACAACTTCCACTTCTGGTGTAAGGTT

>Marker185843

AGTAATTTTGTCCTAAACGAGGAACCAGTCAGTATCAAAAATAAAAATGCAATTATTATCAAAGCAAAATTTATGAACAAXXXXXXXXXXGAAATTATTAGTATTTTTAGCAGGAGATTAAGAATTCATAGAGTTTACCGACATTGCAACTAATTGAGAACATGGATGTC

AGTAATTTTATCCTAAATGAGGAACCAGTCAGTATCAAAAATAAAAATGCAATTATTATCAAAGCAAAATTTATGAACAAXXXXXXXXXXGAAATTATTAGTATTTTTAGCAGGAGATTAAGAATTCATAGAGTTTACCGACATTGCAACTAATTGAGAACATGGATGTC

>Marker185906

ACTCTCACCCATATGAAAATCAAAAGATTGCCTTCATAGACATGAGTTCACAATTCACTTAAGATTCAGGTCAAGTCACTXXXXXXXXXXTGTGACAACTATAAAGCAGACAATTTTTATAGTGTCACCAGAATAAGGTTCTCCATCTACTACAAGACCACAAGATTTAG

ACTCTCACCCATATGAAAATCAAAAGATTGCCTTCATAGACATGAGTTCACAATTCACTTAAGATTCAGGTCAAGTCACTXXXXXXXXXXTGTGACAACTATAAAGCATACAATTTTTATAGTGTCACCAGAATAAGGTTCTCCATCTACTACAAGACCACAAGATTTAG

>Marker185910

GAGAGCTCTTTGTGGGAAGCTCCAACTACCCACTATGCCTTTCCTTCCTATTTCTTAGCCTACAATCTCATTGTATAAAGXXXXXXXXXXTCCTTTGAACGTGTGAGCTAACCAAGTCCCATTTTAGGGATCACTTTTGGCGTGAGTCCCCAACTTGTGGGGTTGTTTAG

GAGAGCTCTTTGTGGGAAGCTCCAACTACCCACTATGCCTTTCCTTCCTATTTCTTAGCCTACAATCTCATTGTATAAAGXXXXXXXXXXTCCTTTGAACGTGTGAGCTAACCAAGTCCCATTTTAGGGATTACTTTTGGCGTGAGTCCCCAACTTGTGGGGTTGTTTAG

>Marker185934

ACTAACCTCGAAATGAGAGACCCTTCAATCTCATAGATGACATCGGTAATGATCCAATTATCTGGGTAAGGCTTTCCACTXXXXXXXXXXAATTGCCTCAATGGTGCCATTGGGAAAAGTAGGGATTTAAGATGGACAAAGCTCCCCCGAGATTGGAGGACGGTGAACTG

ACTAACCTCGAAATGAGAGACCCTTCAATCTCATAGATGACATCGGTAATGATCCAATTATCTGGGTAAGGCTTTCCACTXXXXXXXXXXAATTGCCTCAATGGTGCCATTGGGAAAAGTAGGGATTTAAGATGGACAAAGCTCCCCTGAGATTGGAGGACGGTGAACTG

>Marker185936

ATATATATGTATATACATATTTAAAAAAAACAACACCAAACAAACAAAAAAGGAGGAGAAGAAAGAAAGAAAGAACTGGAXXXXXXXXXXTTCAGCAGATGTTGCAGCACAAGAAGTTGGAGCACTGCTGTCGGTTAAAGAGTTCTCTGGGTTGCTCTCTCTTCTATTGT

ATATATATGTATATACGTATTTAAAAAAAACAACACCAAACAAACAAAAAAGGAGGAGAAGAAAGAAAGAAAGAACTGGAXXXXXXXXXXTTCAGCAGATGTTGCAGCACAAGAAGTTGGAGCACTGCTGTCGGTTAAAGAGTTCTCTGGGTTGCTCTCTCTTCTATTGT

>Marker185987

AAAGTTATTGAGTTGGAAGCAACCTCGACGGGAGTGATCGCAAGGATGACTGAGATAGAAGGAAGAGCAAAGGCTCAATTXXXXXXXXXXGTTTGTGTGTGGGTTGAAGGAAGAAATCCAAAGTGATTCGAAAATTGAACCGAGTAGGTCTGGAGGCTAAAATGATACAT

AAAGTTATTGAGTTGGAAGCAACCTTGACGGGAGTGACCGCAAGGATGACTGAGATAGAAGGAAGAGCAAAGGCTCAATTXXXXXXXXXXGTTTGTGTGTGGGTTGAAGGAAGAAATCCAAAGTGATTCGAAAATTGAACCGAGTAGGTCTGGAGGCTAAAATGATACAT

>Marker186244

AAATGATTCTTTATAGTAAATAATCAATTTGGTTCCAGTTTAAAATTCAATGACCGAGTGAGTGATAGCTGTTTACCATTXXXXXXXXXXAGGAAGCAAATTTCTTTGATATAATTGTCTATAAAAATCATTGACGTTGATCTGAAAATAAAATTTTGAATACTATTTTG

AAATGATTCTTTATAGTAAATAATCAATTAGGTTCCAGTTTAAAATTCAATGACCGAGTGAGTGATAGCTGTTTACCATTXXXXXXXXXXAGGAAGCAAATTTCTTTGATATAATTGTCTATAAAAATCATTGACGTTGATCTGAAAATAAAATTTTGAATACTATTTTG

>Marker186356

TTTTGCAATTTAAAAAATGTAGTGAGATGATCTTTTATCATAATTTTTTGTTGCTATTTTTGTAAATGCCCCAAAAATCAXXXXXXXXXXTCTAATACAAGATTTACAAGCTTAATTTTGTAGGATTACTCGTTAAGACTACTTAGACTACATCAAAATCAAAAGTAAGG

TTTTGCAATTTAAAAAATGTAGTGAGATGATTTTTTATCATAATTTTTTGTTGCTATTTTTGTAAATGCCCCAAAAATCAXXXXXXXXXXTCTAATACAAGATTTACAAGCTTAATTTTGTAGGATTACTTGTTAAGACTACTTAGACTACATCAAAATCAAAAGTAATG

>Marker186595

TTCTCATTCTAACCTTTGGATAATGTGTGTGCGCTTTTTTAATGTCAACTTTTACAAATTCCATACAAAAATTTGATTCTXXXXXXXXXXGGCATGGATCAATTGACTAACCCTTCAATTGAAAACCTATGATTCGATCTTTGGGCTTACTCATTATTAGATCAAAGTTT

TTCTCATTCTAACCTTTGGATAATGTGTGTGCGCTTTTTTAATGTCAACTTTTACAAATTCCATACAAAAATTTGATTCTXXXXXXXXXXGGCATGGATCAATTGACTAACCCTTCAATTGCAAACCTATGATTCAATCTTTGGGCTTACTCATTATTAGATCAAAGTTT

>Marker186724

ACACATTTGAAGAACTAGCAATTCATGCTTATGATATGGAATTGAGCATTGTCAATAGGGGAATCAAGAATTTTTTTGCCXXXXXXXXXXTTGCTCTCATCTTCACTAGCCCAAATAAATTCCATATCTCTCTTTCTTACTATTCCCACTAAAATCAAGCATAATCCATT

ACACATTTGAACAACTAGCAACTCATGCTTATGATATGGAATTGAGCATTGTCAATAGGGGAATCAAGAATTTTTTTTGCXXXXXXXXXXTTGCTCTCATCTTCAGTAGCCCAAATAAATTCCATATCTCTCTTTCTTACTATTCCCACTAAAATCAAGCATAATCCATT

>Marker186848

TTTTTATTCTTTAGTTAGACAGAGTAATGTTTCTTCATACCTCATCACTTGAACAAACTGATTTTTTTTAGATGAAACAAXXXXXXXXXXGGAGCATTTAATTGTTTGCAACTTTTTAACAACTATATTCATGGGCATCCTCTCTCTAGGTGTTGTTGAGGAGCATGACA

TTTTTATTCTTTAGTTAGACAGAGTAATGTTTCTTCATACCTCATCACTTGAACAAACTGATTTTTTTTAGATGAAACAAXXXXXXXXXXGGAGCATTTAATTGTTTGCAACTTTTTAACAACTACATTCATGGGCATCCTCTCTCTAGGTGTTGTTGAGGAGCATGACA

>Marker187155

TTATTTCTACCAAAATTGATACTTCTTATTCAATCTTACCTTGTTGAGCCATCTAAATAAACTTTGTCATATCTTCTGATXXXXXXXXXXAAAACCAAACTAACACATGTTTCTTCATTTAAAAACTTCCAACTTTTTGGAGGATCTTTTCAAACTATTACAACATCAAG

TTATTTCTACCAAAATTGATACTTCTTATTCAATCTTACCTCGTTGAGCCATCTAAATAAACTTTGTCATATCTTCTGATXXXXXXXXXXAAAACCAAACTAACACATGTTTCTTCATTTAAAAACTTCCAACTTTTTGGAGGATCTTTTCAAACTATTACAACATCAAG

>Marker187701

ATTCTTGATTATTATAATTACTAATAATTCTTTAATTAAAGATTGCATTGACATAGTAATTATATTGTAAGAGATCTCAAXXXXXXXXXXCAAACGTCTATTTAAGCATCCGATTTCACACATTTTTCTGCCTCTTTGTTTACTGGGTTGTATTTTACAGCTTCTCTAAT

ATTCTTGATTATTATAATTACTAATAATTCTTTAATTAAAGATTGCATTGACATGGTAATTATATTGTAAGAGATCTCAAXXXXXXXXXXCAAACGTCTATTTAAGCATCCGATTTCACTCATTTTTCTGCCTCTTTGTTTACTGGGTTGTATTTTACAGCTTCTCTAAT

>Marker187859

TTTTTTATTATTTAAAGTTACTGTCATAAAGTCATGTTTTAATAGGCAACAATAAATTCAATGAAAAACTAAAATATCATXXXXXXXXXXTATTTTTTTAAAAGGGAATGAATGCTCTCTATTGTTATTGTTTTGTTGTTGTCATTATTATTATTTCTTAAAGAGAATGA

TTTTTTATTATTTAAAGTTACTGTCATAAAGTCATGTTTTAACAGGCAACAATAAATTCAATGAAAAACTAAAATATCATXXXXXXXXXXTATTTTTTTAAAAGGGAATGAATGCTCTCTATTGTTATTGTTTTGTTGTTGTCATTATTATTATTTCTTAAAGAGAATGA

>Marker187860

ACCCCAACTTTAAATATATGAAATACCTATTTCCTCATATTTTATCTAAAGTTGATGTTTCTTTTTTATCTTGTGATGTGXXXXXXXXXXCATCGGGCTTAGGCTTGCGTGTTTGTTGGGTATCCTTTACACCAACGAGGCTATAAATGCTTCCATCCATCTTCACGCAA

ACCCCAACTTTAAATATATGAAATACCTATTTCCTCATCTTTTATCTAAAGCTGATGTTTCTTTTTTATCTTGTGATGTGXXXXXXXXXXCATCGGGCTTAGGCTTGCGTGTTTGTTGGGTATCCTTTGCACCAACGAGGCTATAAATGCTTCCATCCATCTTCACGCAA

>Marker188361

TTTATACTTTTATGCTTGTGTTCCATTTTTTTCTCACTGAATTTGGGGAGCAACTTTTTCACTTTTTATGCCCATGAGGGXXXXXXXXXXCAGAAAATGATTTTTATTTTAATCAAATCAACGTTGTCTATATGTGAATTTACTTCAATCACATATATTGTCCAATTTAT

TTTATACTTTTATGCTTGTGTTCCATTTTTTTCTCACTGAATTTGGGGAGCAACTTTTTCACATTTTATGCCCATGAGGGXXXXXXXXXXCAGAAAATGATTTTTTTTTTAATCAAATCAACGTTGTCTATATGTGAATTTACTTCAATCACATATATTGTCCAATTTAT

>Marker188640

ATAGATGATCTTTAAATAAAAGACGGAGAAAAAAAAGGTAAACTAGGTGTTGATAAAAAAATTACAACCAATCATAAATTXXXXXXXXXXATTTATCAATAATTAATTCAAAATAATAACATGACATTTCGGAGATGGCTGAAATTTCTGGAGTCTCACCTGTATTCACA

ATAGATGATCTTTAAATAAAAGACGGAAAAAAAAAAGGTAAACTACGTGTTGATAAAAAAATTACAACCAATCATAAATTXXXXXXXXXXATTTATCAATAATTAATTTAAAATAATAACATGACATTTCGGAGATGGCTGAAATTTCTGGAGTCTCACCTGTATTCACA

>Marker188738

CTAATCCGAAAAAGGAAGCCTTGTGGTGAAGGACTCTTGCTTGAAGTGTTGGGACGTCCTGCAAGAAAGGATGCAAAAAGXXXXXXXXXXATATCTTGTCAAAGTGACATGACAACTCGTAGTGTGAAAGAAAACGACGATGACTTCTCAGGAAGAGAAAAATGATAGTT

CTAATCCGAAAAAGGAAGCCTTGTGGTGAATGACTCTTGCTTGAAGTGTTGGGACGTCATGAAAGAAAGGATGCAAAAAGXXXXXXXXXXATATCTTGTCAAAGTGACATGACAACTCGTAGTGTGAAAGAAAACGACGATGACTTCTCTGGAAGAGAAAAATGATAGTT

>Marker188996

CTTATTGTGGTTAATAACTTGTCATCAGCTTTCCATCTAAAATGAGGCAAATCTTAGTCTATAGTAGAAATATCTAATGTXXXXXXXXXXGTGTGAGGACTTGGGTTTAAAAATTTATTATTTTGCAGAACGTGGACTATTGTGATCATGGCACGAGATAGTTGATTGGA

CTTATTGTGGTTAATAACTTGTCATCAGTTTTCCATCTAAAATGAGGCAAATCTTAGTCTATAGTAGAAATATCTAATGTXXXXXXXXXXGTGTGAGGACTTGGGTTTAAAAATTTATTATTTTGCAGAACGTGGACTATTGTGATCATGGCACGAGATAGTTGATTGGA

>Marker189191

CCTGTGAGAAACTCTGACATTTTTTAAATTCTTGTGGTCTGCAGCTTTATTAGCCCTCTCTCTTTCTTCAAATGATGCATXXXXXXXXXXAAATTCTCATCCAAAAGTGATGTCAGCAGAGTCAAGACCTAGTAAAAAAAATTGTAGTCTAGCAAACATGAAAAATACTC

CCTGTGAGAAACTCTGACATTTTTTAAATTCTTGTGGTCTGCAGCTTTATTAGCCCTCTCTCTTTCTTCAAATGATGCATXXXXXXXXXXAAATTCTCATCCAAAAGTGATGTCAGCAGAGTCAAGATCTAGTAAAAAAAATTGTAGTCTAGCAAACATGAAAAATACTC

>Marker189486

TATAAATGATTTGATCCTAATCATTCATGTCGAGACAATAGAGTGGGGGTATTATATACAAAGAGTTTGTATAAGACCGGXXXXXXXXXXTATAGATAGAGTAAGTAGAGAAATTGCTCTCTTAAGAGTTGATTCTATGGCTTGAAAAATGTGGTGCCACACCCTCTCTT

TATAAATGATTTGATCCTAATCATTCATGTCGAGACAATAGAGTGGGGGTATTATATACAAAGAGTTTGTATAAGACCGGXXXXXXXXXXTCTCTATAGATAAAGTAGAGAAATTGCTCTCTTAAGAGTTGATTCTATGGCTTGAAAAATGTGGTGCCACACCCTCTCTT

>Marker189527

TTACTGCGATCTACGGTGTCCATGTTCATCTTCGTGGTAATTTTCGTTTCATCTATTTTTCCTTTCAATCTCATGACCGAXXXXXXXXXXCTTTTGGGCTCTGATACTAAGTGTCAGACTACCAACCCCAAATATCAAATAAAACACACAAACAATAGCAAACAGAGACA

TTACTGCGATCTACAGTGTCCAAGTTCATCTTCGTGGTAATTTTCGTTTCATCTATTTTTCCTTTCAATCTCATGACCGAXXXXXXXXXXCTTTTAGGCTCTGATACTAAGTGTCAGGCTACCAACCCCAAATATCAAATAAAACACACAAACAATAGCAAACAGAAACA

>Marker189823

CAAAATTTGCGAAGAAAAATGTTAAGGAATACATGACCAAAAACCCAATTCGATTCTCTTTTAAAATCCAATGGTTGTAAXXXXXXXXXXTATGTCTTTCTGGAAAAGGTTTTGGCTATAAAATGCAATGTCTAGTAAGAACCAAGTGGTTGTGGTTCCAACCAAGTGAA

CAAAATTTGCAAAGAAAAATGTTAAGGAATACATGACCAAAAACCCAATTCGATTCTCTTTTAAAATCCAATGGTTGTAAXXXXXXXXXXTATGTCTTTCTGGAAAAGGTTTTGGCTATAAAATGCAATGTCTAGTAAGAACCAAGTGGTTGTGGTTCCAACCAAGTGAA

>Marker189863

CTAATGTTATATCAAATAAATATGCTATTTGAAAACCAGGTAGTAGTTAACAATTCTGATGAGAACTGTGAGATATCTCCXXXXXXXXXXAACAGAAAGGAAATGGTGATGAGCAAAATAAGTTATTTTATTTTATGTCACTGATTTGAATGAGCTAAAAATTATGTGTA

CTAATGTTATATCAAATAAATATGCTATTTGAAAACCAGGTAGTAGTTAACAATTTTGATGAGAACTGTGAGATATCTCCXXXXXXXXXXAACAGAAAGGAAATGGTGATGAGCAAAATAAGTTATTTTATTTTATGTCACTGATTTGAATGAGCTAAAAATTATGTGTA

>Marker189955

ATTTAAAAGAGACCATATCTTCCTCTATCACTCTTTCCCTTTCTTCCACTGTATACAAGACTCCACTCACTGGTTACACTXXXXXXXXXXAGTTTGATGTCCATATTTAGAGCTTAATTGGGTAAAAACGAAGCCTTGAAGCATCACTCAAGGCGAAACTTGAAGAAAAA

ATTTAAAAGAGACTATATCTTCCTCTATCACTCTTTCCCTTTCTTCCACTGTATACAAGACTCCACTCACTGGTTACACTXXXXXXXXXXAGTTTGATGTCCATATTTAGAGCTTAATTGGGTAAAAACGAAGCCTTGAAGCATCACTCAAGGCGAAACTTGAAGAAAAA

>Marker190298

ACATATGGGCTTTGAATTGAGTTTTCTTGTCTTCTTCAACCATAATTCTCCAAATTTCTTCTTCTTCTCTTCTCCAATTAXXXXXXXXXXACTTTACTGCTCCAAGCTTAAGTGAACGAAAATGAATAGGATGGTATAAAATTTATATAACATGCCAATGATGGTATAAT

ACATATGGGCTTTGAATTGAGTTTTCTTGTCTTCTTCAACCATAATTCTCCAAATTTCTTCTTCTTCTCTTCTCCAATTAXXXXXXXXXXACTTTACTACTCCAAGTTTAAGTGAACCAAAATGAATAGGATGGTATAAAATTTATATAACATGCGAATGATGGTATCAT

>Marker190501

ACATCTTTTATAGGCTTATAGGCAACAATGTGTTTTGTTTATGAATTCTCTCCAACAGTTGTTTAATTTGTTCTGGATTTXXXXXXXXXXTCTATTCACATAGAAACCCAAAGCTCAGGCTCAGATCAGAGAGGAAACTTATCTAATTATTGTTAAGTAAGTTTACGTTG

ACATCTTTTATAGGCTTATAGGCAACAATGTGTTTTGTTTATGAATTATCTCCAACAGTTGTTTAATTTGTTCTGGATTTXXXXXXXXXXTCTATTCACATAGAAACCCAAAGCTCAGGCTCAGATCAGAGAGGAAACTTATCTAATTATTGTTAAGTAAGTTTACTTTG

>Marker190787

TTTGTTTTAGATTGTTACTTCACCATGAAGGCGTGGAATGCACAAAATGGTGGAGCTGCAGCAATTCTTGTTGCAGATGAXXXXXXXXXXTCTTGATTGGACAGAAGCTCTTCCACATCCCGATGATCGCGTTGAGTATGAATTTTGGACAAATAGCAATGATGAGTGTG

TATGTTTTAGATTGTTACTTCACCATGAAGGCGTGGAATGCACAAAATGGTGGAGCTGCAGCAATTCTTGTTGCAGATGAXXXXXXXXXXTCTTGATTGGACAGAAGCTCTTCCACATCCCGATGATCGCGTTGAGTATGAATTTTGGACAAATAGCAATGATGAGTGTG

>Marker190830

TTGGATATTGGCTGCTGGTTGCATGCTTACATTGAGAAATATTTTGTTTTGACTGATCTCGAGCTTAGCACTGCACTTGTXXXXXXXXXXTTTATGTCTAATAAGAGTTAACACTGATACAGCATCTTAATTAGATTAGTAAGTGATGGATTTATATGAACAAGAGAATA

TTGGATATTGGCTGCTGGTTGCATGCTTACATTGAGAAATATTTTGTTTTGACTGATCTCGAGCTTAGCACTGCACTTGTXXXXXXXXXXTTTTTGTCTAATAAGAGTTAACACTGATACAGCATCTTAATTAGATTAGTAAGTGATGGGTTTACATGAACAAGAGAATA

>Marker191098

ATATTTGTCTTTCCAAAAGAAAATCAGTTTGTAATGAATGATCTTCAATGCATTTACCACAACTTGCCCATAGTAAAGGTXXXXXXXXXXTTTTTTTTATAATTATTATTACTACTTTTGCTTGCAGATTTTGCAGCTTTTTTATGATTCTAAAGATAAGGGTAGGTCAA

ATATTTGTCTTTCCAAAAGAAAATCAGTTTGTAATGAATGATCTTCAATGCATTTACCACAACTTGCCCATAGTAAAGGTXXXXXXXXXXTTTTTTTTATAATTATTATTACTACTTTTGCTTGCAGATTTTGCAGCTTTTTTATGATTCTAAAGATAAGGGTAGGTTAA

>Marker191287

AAGGTTCAGTAGAGTTTTCCATACCTTGATCTCAAGGAGGTTCAGAGTCTTGGATTGGAGCCGGTGGCTGACTAGTAGGGXXXXXXXXXXACATTTATAACCATGCTGGTGAAAGGGATACCCAACAAACACAAATGTCTGAGCTCGATGGGTAAATTTGGTCTGATTAG

AAGGTTCAGTAGAGTTTTCCATACCTTGATCTCAAGGAGGTTCAGAGTCTTGGATTGGAGCCGGTGGCTGACTAGAAGGGXXXXXXXXXXACATTTATAACCATGCTGGTGAAAGGGATACCCAACAAACACACATGCCTGAGCTCGATGGGTAAATTTGGTCTGATTAG

>Marker191342

ATGGAATCAACCGAATTTGAACTGTCTCCAATTCTCTGATTCTCAACCTAAATATTTCATCAATCTGGGTAACTCTTTGTXXXXXXXXXXTATTTGATAGTGGTAGAGTAAATTAGAGGGGGGCGGGGGTTTTTCTTCAGCGATGGGATTATGGCTGTTAGCTTCAACTG

ATGGAATCAACTGAATTTGAACTGTCTCCAATTCTCTGATTCTCAACCTAAATATTTCATCAATCTCGGTAACTCTTTGTXXXXXXXXXXTATTTGATAGTGGTAGAGTAAATTAGAGGGGGGCGGGGGTTTTTCTTCAGCGATGGGATTATGGCTGTTAGCTTCAACTG

>Marker191908

ATGACTTTGAGAAAAGTTAAACATTAAATTAATTATTTCAACTTTCGTTGACGATTCTTGTCGTGACGTCGGAGAAAGTTXXXXXXXXXXTGGTGCTTTTCGAGCGGTGGGAATGGTAGGACATTCCCAACCTTCGTTTTGGGGTCAAAAATCGTCCAATTTCTTGTAGT

ATGACTTTGAGAAAAGTTAAACATTAAATTAATTATTTCAACTTTCGTTGACGATTCTTGTCGTGACGTTGGAGAAAGTTXXXXXXXXXXTGGTGCTTTTCGAGCGGTGGGAATGGTAGGACATTCCCGACCTTCGTTTTGGGGTCAAAAATCGTCCAATTTCTTGTAGT

>Marker192229

ACTGTCATGTATTCTTCAGTTTTCTAACTTGTATATTGTTCATTAAATTAAGTCATTTCTTACACACTGGCAGCCACAAAXXXXXXXXXXTCCTCTGACAGCTCTATGATGGTTGCCATTTCCTTCATCCTCTATCTCTCTTGCTTTGCTCTATACCTTCCTGTTTTTTG

ACTGTCATGTATTCTTCAGTTTTCTAACTTGTATATTGTTCATTAAATTAAGTCATTTCTTACACACTGGCAGCCACAAAXXXXXXXXXXTCCTCTGACAGCTCTATGATGGTTGCCATTTCTTTCATCCTCTATCTCTCTTGCTTTGCTCTATACCTTCCTGTTTTTTG

>Marker192253

AGTCTATTGTCTCTTCAAATATCTTTATTAATATGATATTGTTTACTTTGGATATATAGTATCACCTCAAATACCTCATAXXXXXXXXXXTCAATAAGATAGAAACAATTAATGGAGACATCAGGTGAGTGAACTAGGGCTTGGGAGAGATCTCAAGAGGGAGGGTGAAA

AGTCTATTGTCTCTTCAAATATCTTTATTAATATGATATTGTTTACTTTGAATATATAGTATCACCTCAAATACCTCATAXXXXXXXXXXTCAATAAGATAGAAACAATTAATGGAGACATCAGGTGAGTGAACTAGGGCTTGGGAGAGATCTCAAGAGGGAGGGTGAAA

>Marker192695

TCTATTTTAACCTATTTAAGCTGATAGGAATTTTGATCCGTTAGTCTATAGATTTATACGTATTGTGGGAACTTCTGTCTXXXXXXXXXXGAAGATTTGAAGTTCAAATTTCTAGGAGCCTCTTTGATATACTTTGGTTTTTCATGAACAGAATCTCAGTTCTATTGGTT

TCTATTTTAACCTATTTAAGCTGATAGGAATTTTGATCCGTTAGTCTATAGATTTATACGTATTGTGGGAACTTCTGTCTXXXXXXXXXXGAAGATTTGAAGTTCAAATTTCTAGGAGCCTCTTTGATATACTTTGGGTTTTCATGAACAGAATCTCAGTTCTATTGGTT

>Marker192903

TTTTGCAATAAGTTAGAGCCACACAAGGAGAGTCCACCTTTAGTTATATTTAGTTCCTAAACTAAATTCCAAAAATTAACXXXXXXXXXXATCACTTAAACTAATTTTGAATACGAATCTCCACTAATATTGTTAGTTAGAAACGTGGGTTGATTCTTGAATAATGATGG

TTTTGCAATAAGTTAGAGCCACACAAGGAGAGTCCACCTTTAGTTATATTTAGTTCCTAAACTAAATTCCAAAAATTAACXXXXXXXXXXATCACTTAAACTAATTTTGAATATGAATCTCCACTAATATTGTTAGTTAGAAACGTGGGTTGATTCTTGAATAATGATGG

>Marker193229

CTTGCTCAAGTAAGCTTTGAGAGCTTACCAATATTTTTTTGTTAGAAAATGATGTGAGACTTTGAGTCTCTCTTCTAGTCXXXXXXXXXXTTGTTTTGCTATATCAATTTTGATTGGCATAGGGTTTGTTCCTCGTTTCTTCCCTAAAATCTATGCTTGACCTAAGTATG

CTTGCTCAAGTAAGCTTTGAGAGCTTACCAATATTTTTTTGTTAGAAAATGATGTGAGACTTTGAGTCTCTCTTCTAGTCXXXXXXXXXXTTGTTTTGCTATATCAATTTTGATTGGCATAGGGTTTATTCCTCGTTTCTTCCCTAAAATCTATGCTTGACCTAAGTATG

>Marker193311

TTTATAGAACCTTGATCCATTATTGTAATCTAGTAACTACTCTCTTGGAGTTATTACAAAATCCTACTAGATAGCACACAXXXXXXXXXXGCAGAAGCCAGTAGTGCTATAATCTGTTTAAATTTGACAAAGTAAGTATCTGTGACTCTGAGGCACTATACCCGCATAGT

TTTATAGAACCTTGATCCATTATCGTAATCTAGTAACTACTCTCTTGGAGTTATTACAAAATCCTACTAGATAGCACACAXXXXXXXXXXGCAGAAGCCAGTAGTGCTATAATCTGTTTAAATTTGACAAAGTAAGTATCTGTGACTCTGAGGCACTATACCCGCATAGT

>Marker193434

ATAAACAACTTTAAACCTATGGGTAATGGGCAAAGCTTGACTGAAAAAAGCCAATGGATGTTGATGCCTGTTTCTAGCACXXXXXXXXXXCACATTCTTGGGTGCTGGCAATTTAACCATAATTGCTATTTTTGTTGGATTCGTCGTCACCCCATCGAACAAGATAATGT

ATAAACAACTTTAAACCTATGGGTAATGGGCAAAGCTTGACTGAAAAAAGCCAATGGATGTTGATGCCTGTTTCTAGCACXXXXXXXXXXCACATTCTTGGGTGCTGGCAATTTAACCATAACTGCTATTTTTGTTGGATTCGTCGTCACCCCATCGAACAAGATAATGT

>Marker193901

ACCCACCCAAGAATTATTTCCAGTGAGATTAATTGAGGTGCACGTAAGTTGACCCATGATCTAATGACACCCAACAAGGAXXXXXXXXXXATTTTGGCTGTTTTGCACTGCTGCAATTGCTGTATTTGGACTTCTTATGGTCTTTGGATTTTCTTTGGGATAAGATGTTG

ACCCACCCAAGAATTATTTCCAGTGAGATTAATTGAGGTGCACATAAGTTGATCCATGATCTAATGACACCCAACAAGGAXXXXXXXXXXATTTTGACTGTTTAGCACTGCTGCAATTGCTGTATTTGGACTTCTTATGGTCTTTGGATTTTCTTTGGGATAAGATGTTG

>Marker194182

CAGCTTGCTGATCCGGCTCGAGGTCGAGTCTAGTGTTAGGGGGTGGATTATGGTGTAAACAAAGCTATAGAATAAAAGACXXXXXXXXXXGAATCCAATATATTCTTTTTTGATATAAACAAGCGCTTTCATTGAGAAAGAAGGAAGAAATACATGGCATATAAAAAGGG

CAGCTTGCTGATCCGGCTCGAGGTCGAGTCTAGTGTTAGGGGGTGGATTATGGTGTAAACAAAGCTATAGAATAAAAGACXXXXXXXXXXGAATCCAATATATTCTTTTTTGATATAAACAAGCGCTTTCATTGAGAAAGAAGGAAGAAATACATGGCATACAAAAAGGG

>Marker194289

AATGCTCCAAGATAGGAGAGGAAAATCAATTACACAAAACTATTAATCCATTTGGTGATGAAAATGTTTACAAGAACCAAXXXXXXXXXXTTAATTCCTAGGTAAGCGGCAACCATGGATCAAACCCATGACCTCTTAATTATTGAGATTGTGTCTATTTTTTACCACAA

AATGCTCCAAGATAGGAGAGGAAAATCAATTACACAAAACTATTAATCCATTTGGTGATGAAAATGTTTACAAGAACCAAXXXXXXXXXXTTAATTCCTAGGTAAGTGGCAACCATGGATCAAACCCATGACCTCTTAACTATTGAGATTGTGTCTATTTTTTACCACAA

>Marker194606

AGGACTGAGTAATACGACATCGGTTTGAAAGGGTTTGATGTAGGAAGCGTCGACGTCGACGACGGTGAGAGAATGGTTGGXXXXXXXXXXATTAGGTATCAGCTTAAACTAAAATTAGTTTAGTGAGCTTTTAAAAAATGGAGTCAAACAAAGTAAGATTAATAGTGAGA

AGGACTGAGTAATACGACATCGGTTTGAAAGGGTTTGATGTAGGAAGCGTCGACGTCGACGACGGTGAGAGAATGGTTGGXXXXXXXXXXATTAGGTATTAGCTTAAACTAAAATTAGTTTAGTGAGCTTTTAAAAAATGGAGTTAAACAAAGTAAGATTAATAGTGAGA

>Marker194646

TCTCACCCATGAGAATAACAATATTAAAAAGATACAAGAATGGACATAAAGTCAAGAATGGTGTCGAGCAGCAGAATGCAXXXXXXXXXXATGCCTTGTTGAGCAACTTGTTCATATCCTCTGCAATTGTTAGTGCAGAAAGCATTTGCTTTAAGTCACATCAACTCCAA

TCTCACCCATGAGAATAACAATATTAAAAAGATACAAGAATGGACATAAAGTCAAGAATGGTGTCGAGCAGCAGAATGCAXXXXXXXXXXATGCCTTGTTGAGCAACTTGTTCATATCCTCTGCAATTGTTAGTGCAAAAAGCATTTGTTTTAAGTCACATCAACTCCAA

>Marker194924

TTAAATTGATCACATGGAAGACCTCTACCTATAAATTATTCAATTCTTTATATGTTATTTAATTTCATTAATACTTTTTTXXXXXXXXXXTAGATGACTAATCACATCCATAAGGATTGGTGACAACAATGAACCTTTCACCTCGGAAGAGACTTTTAGGGAAGAATGCC

TTAAATTGATCACATGGAAAACCTCTACCTATAAATTATTCAATTATTTATATGTTATTTAATTTCATTAATACTTTTTTXXXXXXXXXXTAGATGACTAATCACATCCATAACGATTGGTGACAACAATGAACCTTTCACCTCGGAAGAGACTTTTAGGGAAGAATGCC

>Marker195201

ATTATCCTCATGATGATAAGATAATCTCTCTCTGCAAGAAAAACTTGCACAGAGAAGCTCTTAAAGCATTTGACATCTTTXXXXXXXXXXGGGTCTTTGAAGGAAGCAAGAAATATGTTTGATTCAATGCCTCTGAAGAATGTAGTATCTTGGACCTCCATGATATCTGG

ATTATCCTCATGATGATAAGATAATCTCTCTCTGCAAGAAAAACTTGCACAGAGAAGCTCTTAAAGCATTTGACATCTTTXXXXXXXXXXGGGTCTTTGAAGGAAGCAAGAAATGTGTTTGATTCAATGCCTCTGAAGAATGTAGTATCTTGGACCTCCATGATATCTGG

>Marker195472

TCAGCCACATTATTCGAACACAATTCATTGAGTGTGAAGTTATATGGCAACATATGTTTCTTTGGAGAGATAAAGACAATXXXXXXXXXXTTACTGCTTTTTGGAAAATATATACAATATCATATTGTTGGAGTATAATTGCCCATTTAGCAAGACGTCTCGAGATGATT

TCAGCCACATTATCCGAACACAATTCATTGAGTGTGAAGTTATATGGCAACATATGTTTCTTTGGAGAGATAAAGACAATXXXXXXXXXXTTACTACTTTTTGGAAAATATATACAATATCATATTGTTGGAGTATAATTGCCCATTTAGTAAGACGTCTCGAGATGATT

>Marker195516

CAAATTAAAGTGATTAGCTGCTCAATTTTGAGAGACATTAAATGGTAGATTATGGGCTTTTCCAAGTTGACAGGAATCATXXXXXXXXXXATGTTGAAACTGTCGTTTTCTACCTTCACTGTTCTCCAACTCACCAACTGACTTCATTGCAACACCTTCAAGGTGATAGA

CAAATTAAAGTGATTAGCTGCTCAATTTTGAGAGACATTAAATGGTAGATTATGGGCTTTTCCAAGTTGACAGGAATATAXXXXXXXXXXATGTTGAAACTGTCGTTTTCTACCTTCATTGTTCTCCAACTCACCAACTGACTTCACTGCAACACCTTCAAGGTGATAGA

>Marker196480

TCCTAATCAGAATTCTTATGAATCACTTTAATCAGATAATTAAAATAATATAATTTAGTGTTATAAAATTCAGTCACCCTXXXXXXXXXXAGCCAATCGCGCCTAAGCGATTCATTTCGTTTTCATTTTTCGCGTCTTTCTTTTGAAAAGCACAAGCAAAGGATCTTTTT

TCCTAATCATAATTCTTATGAATCACTTTAATCAGATAATTAAAATAATATAATTTAGTGTTATAAAATTCAGTCACCCTXXXXXXXXXXAGCCAATCGCGCCTAAGCGATTCATTTCGTTTTCATTTTTCGCGTCTTTCTTTTGAAAAGCACAAGCAAAGGATCTTTTT

>Marker196613

CTATTCATGAGAAAGCATGTCGTTAATTTTTCCATATGGATCATACCAAATTTCCAAGCATTATCCAACCACTAGTTTAAXXXXXXXXXXTTACTCGAAATATTTATGATTTCTTAGTCAAGAGACAATTCATTAATGGAAAAAGAAGTATGTGAAAAGGGGGAGTCATT

CTATTCATGAGAAAGCATGTCGTTAATTTTTCCATATAGATCATACCAAATTTCCAAGCATTATCCAACCACTAGTTTAAXXXXXXXXXXTTGCTCGAAATATTTATGATTTCTTAGTCAAGAGACAATTCATTAATGGAAAAAGAAGTATGTGAAAAGGGGGAGTCATT

>Marker196738

CTCGTAATAGGCTCGTGCCTGTCCAGCCGGCTACTCTATGTCTGCAGCATGAATGAAGGTTAGCACAGCCCTGTTGGTGCXXXXXXXXXXGACCTCTCATGGTGCTCATTGTTAGTTTTCGGGATCTGCCATAGAGCAGTTGGCTGGACAGGTGCAAGTGCAAAGAACAC

CTCGTAATAGGCTCGTGCCTGTCCAGCGGGCTACTCTATGTCTGCAGCATGAATGAAGGTTAGCACAGCCCTGTTGGTGCXXXXXXXXXXGACCTCTCATGGTGCTCATTGTTAGTTTTCGGGATCTGCCATAGAGCAGTTGGCTGGACAGGTGCAAGTGCAAAGAACAC

>Marker197281

TTCTTTCTCTCATTTTCTTTCATGTTTCTCAATGAACGTGTAGTTTCTTATTGATAAAAAAAATTGATGCAACTACAAAAXXXXXXXXXXGACGCACAAGAAACTAAAACACGCTTGAGCAAGGAAGCTGTTGCTCTATCCTTCCGGTCCATAGCACTGCGAAAATTCAT

TTCTTTCTCTCATTTTCTTTCATGTTTCTCAATGAACGAGTAGTTTCTTATTGATAAAAAAAATTGATGCAACTACAAAAXXXXXXXXXXGACGCACAAGAAACTAAAACACGCTTGAGCAAGGAAGCTGTTGCTCTATCCTTCCGGTCCATAGCACTGCGAAAATTCAT

>Marker197343

AATTCGTAACCGTCTTGATTGATGATTTTGTATGCTCCATTTGTGAAAACTTCTTTGACGATGTAGGGTCTGTCCCATTTXXXXXXXXXXGCGTAATCTAGCGTTGTCTTCAGTAGTTAGTCCTTCTTGAATTGTCATTCTCAATGATGGAATTTCTCTCTTTAGCAGAA

AATTCGTAACCGTCTTGATTGATGATTTTGTATGCTCCATTTGTGAAAACTTCTTTGACGATGTAGGGTTCGTCCCATTTXXXXXXXXXXGCGTAATCTAGCGTTGTCTTCAGTAGTTAGTCCTTCTTGAATTGCCATTCTCAATGATGGAATTTCTCTCTTTAGCAGAA

>Marker197588

ACTCCCACAAGAAAATTGAGGGAGGGAAAGATTCTGAATATTATTTTCGATTGTTGATATATAACTAAGTAAAAGAAATTXXXXXXXXXXAAAACCTGTTTCCAAGCATTTGATGTAGATTAATAATGGTTTCCTCTTCTTCTGCGGTAAAATTTCCTCGTTTAATGTCT

AATCCCACAAGAAAATTGAGGGAGGGAAAGATTCTGAATATTATTTTCTATTGTTGATAAATAACTAAGTAAAATAAATTXXXXXXXXXXAAAACCTGTTTCCAAGCATTTGATGTAGATTAATAATGGTTTCCTCTTCTTCTGCGGTAAAATTTCCTCGTTTAATGTCT

>Marker197825

ATTTAAAGAGTTTACTCACAGTATTATTCACCGTTCTGGACTCATACAGCATACAATCTGACAACCTGGTCAATCTTGGCXXXXXXXXXXGTCAATTGGACTTGAATCGATTGAAGGATAGTGAATTGCACCATTGTCAAGAATCTCATCGGCTGCCAATGGGGCTGAAG

ATTTAAAGAGTTTACTCACAGTATTATTCACCGTTCTGGACTCATACAGCATACAATCTGACAACCTGGTCAATCTTGGCXXXXXXXXXXGTCAATTGGACTTGAATCGATTGAAGGATAGTGAATTGCACCGTTGTCAAGAATCTCATCGGCTGCCAATGGGGCTGAAG

>Marker197968

TAATGCTGGATTATACAACCCTTTACCGATTCCTACCACTATATGGAAAGATTTATCAGTAGACTTCGTATTGGGGTTACXXXXXXXXXXTGTGTTTGACAGGGACGTGAAATTCTTAAACCACTTTTGGTGCATTTTGTGGAAGAAACTAGATACCACACTCAAATTCA

TAATGCTGGATTATACAACCCTTTACCGATTCCTACCACTATATGGGAAGATTTATCAGTAGACTTCGTATTGGGGTTACXXXXXXXXXXTGTGTTTGACAGGGACGTGAAATTCTTAAACCACTTTTGGTGCATTTTGTGGAAGAAACTAGATACCACACTCAAATTCA

>Marker198124

CTAATTGAACAACAGCCCCATTGAGGAGGCAACACCAGGATCACTTTAGCAATTCACCTAAGACGGTGACTAGGGAGCACXXXXXXXXXXAAGCAATAGAATTAGTAGCAGATACAGTCATGCTGTTTATAGCACCTGGCTGAGAACACACTTCCATAAGAGAAGGTATA

CTAATTGAACAACAGCCCCATTGAGGAGGCAACACCAGGATCACTTTAGCAATTCACCTAAGACGGTGACTAGGGAGCACXXXXXXXXXXAAGCAATAGAATTAGTAGCAGATACAGTCGTGCTGTTTATAGCACCTGGCTGAGAACACACTTCCATAAGAGAAGGTATA

>Marker198243

TTTGACAATGTGTTGGAAGAGAATCTGGCATTGAAACAAACATATCGATTATCATACCGCACCCCAAGCATTTGCTTGCTXXXXXXXXXXAAAACATGAAGATATAAACTTTTAATAAATGCAGTAATACTTTTAATAAATCTATTCTTCCCATGATTGAAAATTTGATT

TTTGACAATGTGTTGGAAGAGAATCTGGCATTGAAACAAACATATCGATTATCATACCCCACCCCAAGCATTTGCTTGCTXXXXXXXXXXAAAACATGAAGATATAAACTTTTAATAAATGCAGTAATACTTTTAACAAATCTATTCTTCCCATGATTGAAAATTTGATT

>Marker198978

TCTTTGATGTCCCCAGAGTCTCACGACCATATTCAACTGGTTCATATCTATTATCATACTCCATTTCAGTAGTCAATGACXXXXXXXXXXTTCCCAAAACCTTGAATTCGGCGATTAAGATTCTCAGATGGTGCAGGCTTGTTATCCTCTTCAGCAAAGATAGAAGAAAT

TCTTTGATGTCCCCAGAGTCTCACGACCATATTCAACTGGTTCATATCTATTATCATACTCCATTTCAGTAGTCAATGACXXXXXXXXXXTTCCCAAAACCTTGAATTCGACGATTAAGATTCTCAGATGGTGCAGGCTTGTTATCCTCTTCAGCAAAGATAGAAGAAAT

>Marker199043

TCCTTTTCGGTGCCTAGAGCGATAAGGTTCCAAAAACTAGGAAGCGCGAAAGGATGGAGGGAGCCGAGGTAGACAATATGXXXXXXXXXXACAAGATTTGCAAAAGGAATTCATGTCCTTAGAAGAAGAAGCAAAGGAAGAGAGTGGGAGCCAAAGGTGGGGGAGGACGT

TCCTTTTCGGTGCCTAGAGCGATAAGGTTCCAAAAACAAGGAAACGCGAAAGGATGGAGGGAGCTGAGGTAGACAATATGXXXXXXXXXXACAAGATTCACAAAAGGAATTCATGTCCTTAGAAGAAGAAGCAAAGGAAGAGAGTGGGAGCCAAAGGTGGGGGAGGACGT

>Marker199536

TTTGAATAATAAGAATGATCGTTTGTGTCATCCTTGTGCCTTAGAAAAATCTATTACCTACCTTTTACTGATTCAACTACXXXXXXXXXXTCACATATCAAGCCTTTTCAATTTTCATTAAACAAACATCTCCTTGAAGGCAGAGAGTGGCAACGCATATGAATAAGTTG

TTTGAATAATAAGAATGATCGTTTGTGTCATCCTTGTGCCTTAGAAAAATCTATTACCTACCTTTTACTGATTCAACTACXXXXXXXXXXTCACATATCAAGCCTTTTCAATTTTCATTAAACAAACATCTCCTTGAAGGCAGAGAGTGGCAATGCATATGAATAAGTTG

>Marker199914

CACTATGCCTTATGGCTACTCATCAACTCATTTGTTGCTTCTAATTCCTTCTTCAAACATGGAACTCTTAACTCATGATAXXXXXXXXXXTCTATTTTGAACCAAGGTTTCTGGGTTGAGAGTAAAAAATTAACCCATTGGACCTTTTTGCCTTGGCTTCTTCAAGCTCG

CACTCTGCCTTATAGCTACTCATCAACTCATTTGTTGCTTCTAATTCCTTCTTCAAACATGGAACTCTTAACTCATGATAXXXXXXXXXXTCTATTTTGAACCAAGGTTTCTGGGTTGAGAGTAAAAAATTAACCCATTGGACCTTTTTGCCTTGGCTTCTTCAAGCTCG

>Marker199944

AAGAGAGAAGAAGCCTTGGGGAAGAGGAGAGGGCTCGAAGAATGTGGCTCCGAGGTTGCTTGGACCGCCGTCGTGGGTGGXXXXXXXXXXAATTTAAATATATTTTGTTGAAATTTATTCTAATTAATTAAATTTTAAAATCAAGTTTTTTCCTAATAATTATTTCTTTA

AAGAGAGAAGAAGCCTTGGGGAAGAGGAGAGGGCTCGAAGAATGTGGCTCCGAGGTTGCTTGGACCGCCGTCGTGGGTGGXXXXXXXXXXAATTTAAATATATTTTGTTGAAATTTATTCTAATTAATTAAATTTTAAAATCAAGTTTTTTCCTAATAATTGTTTCTTTA

>Marker200073

TTTGCACACTTTCACTCCAAATTGGAGATAGTTATGTGGTGGAGCCACATCTTCATTTGCCTTCATAAATCCCACTGACCXXXXXXXXXXTAATCCAAGAATTGGAGTTAATAAAGAAAAAGTAACTTTTAGTAAATCCAATAGGAGCAAATGTTATATTCAATTGGTTT

TTTGCACACTTTCACTCCAAATTGGAGATAGTTATGTGGTGGAGCCACATCTTCATTTGCCTTCATAAATCCCACTGACCXXXXXXXXXXCAATCCAAGAATTGGAGTTAACAAAGAAAAAGTAACTTTTAGTAAATCCAATAGGAGCAAATGTTATATTCAATTGGTTT

>Marker200293

AAAGCATGAGTATTCAATTTTGACATTATAGAATTTGAAAGCCTAGCTTTTATGATTGAACAATTATATTCCAATCGTAAXXXXXXXXXXAGGATTTAGGGTGGTGATTTAAACTTCTTTAGCCAAGAACAGAACATATGAATTATTCAGTTGGGTTATTTTAAAGTTTA

AAAGCATGAGTATTCAATTTTGACATTATAGAATTTGAAAGCCTAGCTTTTATGATTGAACAATTATATTCCAATAGTAAXXXXXXXXXXAGGATTTAGGGTGGTGATTTAAACTTCTTTAGTCAAGAACAGAACATATGAATTATTCAGTTGGGTTATTTTAAAGTTTA

>Marker200326

TATACCTCAACCCCTTTATAATGTCAAAATCCTCTCGCCCTAAGAAGACCTTATCACCCAGTAATTTAAAAATAATTATGXXXXXXXXXXAAGGAAGGAGTCAGAATTGCAGATTTTGGTGATAAGAGGCATTTGCAAAACAGAAAAAAATACATAACAAAAAATGATAA

TATACCTCAACCCCTTTATAATGTCAAAATCCTCTCGCCCTAAGAAGACCTTATCACCCAGTAATTTAAAAATAATTATGXXXXXXXXXXGGATAAGGAGTCAGAATTGCAGATTTTGGTGATAAGAGGCATTTGCAAAACAGAAAAAAATACATAACAGAAAATGATAA

>Marker200725

ATACTTAGAGAAAATAGTCTTAAGGTTAGCTTTTAGCTCAAATAGTTTCAAAATTGATTTTGAGAAGAATTTAACTAAGTXXXXXXXXXXACAAAAATGATGATATCTCTTTGTCTCAAAATGATCCTAACTTAGAGAATGTTGATTCAAATGCCTAAAATGAAAAGAAG

ATACTTAGAGAAAATAGTCTTAAGGTTAGCTTTTAACTCAAATAGTTTCAAAATTGATTTTGAGAAGAATTTAACTAAGTXXXXXXXXXXACAAAAATGATGATATCTCTTTGTCTCAAAATGATCCTAACTTACAGAATGTTGATTCAAATGCCTAAAATGAAAAGAAG

>Marker200958

TCAAACTGGTCGATCTTGGAGTCATACAAAATTAAAGCCCTTTGAGAAATAAGAAACAACATCGTTTCATCTTCAAAAACXXXXXXXXXXAATAAGAAAAGCATCATCTCTCACAACTTGAGGAAAAGAAACAACTTCAAATTTCTCATCTTCTACATCAAAACGTAAAA

TCAAACTGGTCGATCTTGGAGTCATACAAAATTAAAGCCCTTTGAGAAATAAGAAACAACATCTTTTCATCTTCAAAAACXXXXXXXXXXAATAAGAAAAGCATCATCTCTCACAACTTGAGGAAAAGAAACAACTTCAAATTTCTCATCTTCTACATCAAAACGTAAAA

>Marker201152

ATCATACAAGGAAAGAACTTTTTAGGATCTCATATTAAACCTAAAGCAAATAGTAAATAGTGCACAATTTCTACTTAGTAXXXXXXXXXXGGACAACTTCGAGTGGATACGTGGGTATGACACAAGGTTTGGGCTTTTCTATGTGGATCACTTGAAAACCCTTGAACGGA

ATCATACAAGGAAAGAACTTTTTAGGATCTCATATTAAACCTAAACCAAATAGTAAATAGTGCACAATTTCTACTTAGTAXXXXXXXXXXGGACAACTTCGAGTGGATACGTGGGTATGACACAAGGTTTGGGCTTTTCTATGTGGATCACTTGAAAACCCTTGAACGGA

>Marker201316

ATTAATAGTTTGTTCGAGATGGGACCAATAAAAACTTCATGCATAATTTTTTTTCTTTGAAAGTGAAGGTTGATGACAATXXXXXXXXXXAGTGTCCATCATTACTAGAAATTACAATATTTGGCACGTGCTTTTGGATGTGGTAGTGAGACAATGAGCTCTAATATTGA

ATTAATAGTGTGTTCGAGATGGGACCAATAAAAACTTCATGCATAATTTTTTTTCTTTGAAAGTGAAGGTTGATGACAATXXXXXXXXXXAGTGTCCATCATTACTAGAAATTACAATATTTGGCACGTGCTTTTGGATGTGGTAGTGAGACAATGAGCTCTAATATTGA

>Marker201363

ACTCACTCACTATACTCACTTTATCCTACTTCCCACTAAATGCAAAGCTACACTTTCCCTTTGTCAAGAAGGATCAAAACXXXXXXXXXXGAATTGGGAGTTCTAACGAAGGAGATCTGGTAGTTTCCCCCTGTCAGAGGCGCGGTGCTGTAAAACACCAATTCCTCTAA

ACTCACTTACTATACTCACTTTATCCTACTTCCCACTAAATGCAAAGCTACACTTTCCCTTTGTCAAGAAGGATCAAAACXXXXXXXXXXGAATTGGGAGTTCTAACGAAGGAGATCTGGTAGTTTCCCCCTGTCAGAGGCGCGGTGCTGTAAAACACCAATTCCTCTAA

>Marker201795

TGCCATCATCTTTAGTTTCATTAATCTTTTTCTTGGAAAGAAAAATAAACTTTAGTTTCCATCTTGTATTCCCTCCTTTTXXXXXXXXXXGACCTTCTTGCCCCAAGAAGGTAATTCTCTCGTAAATTCAAGCCCCCCCATGCCCAGTGCGAAAAAACCGAACTCGAAGA

TGCCATCATCTTTAGTTTCATTAATCTTTTTCTTGGAAAGAAAAATAAACTATAGTTTCCATCTTGTATTCCCTCGTTTTXXXXXXXXXXGACCTTCTTGCCCCAAGAAGGTAATTCTCTCGTAAATTCAAGCCCCCCCATGCCCAGTGCGAAAAAACCGAACTCGAAGA

>Marker201894

CTCCGTGATGTTGATCCTGGTAAGTTTCTTTTGTGTTTCGTTACAGGTTCAAGGGATGTATACATAAAATCAACATAGGAXXXXXXXXXXACTCTGCTGCACCGAGGGTTTGGTTTTATTTGCAACTGTGCACGATGTTCATATGGTGATTAAGGGGAGTTTAATTATTG

CTCCGTGATGTTGATCCTGGTAAGTTTCTTTTGTGTTTCGTTATAGGTTCAAGGGATGTATACATAAAATCAACATAGGAXXXXXXXXXXACTCTTCTGCACCGAGGGTTTGGTTTTATTTGCAAATGTGCACGATGTTCATATGGTGATTAAGGGGAGTTTAATTATTG

>Marker202173

TCGAGGCAAAAGCCTCTTCTTCAAACTAAGTTGAAGTAAAATTGGTCTTCTAGCAGCGAAAGATGATTCGTATTCCATTTXXXXXXXXXXTGAGTGTTGAATCCCATTTCCTTAACTCTCTCTACAGTCTCCTTGAATTGGATGGAATTTGTCATGAACACTCTAGGCTG

TCTAGGCAAAAGCCTCTTCTTCAAACTAAGTTGAAGTAAAATTGGTCTTCTAGCAGCGAAAGATGATTCGTATTCCATTTXXXXXXXXXXTGAGTGTTGAATCCCATTTCCTTAACTCTCTCTACAGTCTCCTTGAATTGGATGGAATTTGTCATGAACACTCTAGGCTG

>Marker202279

ATATAGTATCCGTTGAATAAAATAGGAAATAAATGTTCTACTTTATTAATTTGAATGAGATAGACGTTACATCCGGCTATXXXXXXXXXXTACTATGAGCACACCAAAGTGCTCAACATGTGGGACATGTTTGATCCAAGAAGTAGGAGTTAGGAGAAGTGGAGTTGTTG

ATATAGTATCCGTTGAATAAAATAGGAAATAAATGTTCTACTTTATTAATTTGAATGAGATAGACGTTACATCCGGCTATXXXXXXXXXXTACTATGAGCACATCAAACTGCTCAACATGTGGGACATGTTTGGTCCAAGGAGTAGGAGTTAGGAGAAGTGGAGTTGTTG

>Marker202315

CATTTGACTCCAGTGACAATAACTTCAATTAACAGCTTCAAAAAGAAAAAGGGTCAATCCTACTGCCATTTTTTAGTGGGXXXXXXXXXXAACAACTTGAAACAAAAAATTAACAAATAAAGCCAAATCATTACCTCCAACTCAAATTCTGAACGATTAAGCTTCAAGAG

CATTTGACTCCAGTGACAATAACTTCAATTAACAGCTTCAAAAAGAAAAAGGGTCAATCCTACTGCCATTTTTTAGTGGGXXXXXXXXXXAACAACTTGAAACAACAAATTAACAAATAAAGCCAAATCATTACCTCCAACTCAAATTCTGAACGATTAAGCTTCAAGAG

>Marker202525

TATCCTGAATATGAATATTGCGAACTATAAGATTTCTAGTGTATAATAACTAGTATTGCCATAAAAGTAGAGAAGGGAAAXXXXXXXXXXTAGAGTTTGGGCACAGCATATGCGTAGGTGTGTCTACTAGGTGGAGAAACGTTTTATTGGGGTTCTACAACAGCATATTT

TATCCTGAATATGAATATTGCGAACTATAAGATTTCTAGTGTATAATAACTAGTATTGCCATAAAAGTAGAGAAGGGAAAXXXXXXXXXXTAGAGTTTGGGCACAGCATATGCGTAGGTGTGTCTACTAGATGGAGAAACGTTTTATTGGGGTTCTACAACAGCATATTT

>Marker202757

CTATTAAACTAATATAAGCTATAAATTATTGAGTCGACCCATTCCACATCTTTTTGAATTTTACAAATCAATTAGAAAGAXXXXXXXXXXGTAGAGAGAGAAGTTGAGAAAAAGTAGTAAAAAAAAAGAAAAGAAAAGAAAAAGAAAAATGGCAGTCTGGTGAAACGGTA

CTATTAAACTAATATAAGCTATAAATTGTTGAGTCGACCCATTCCACATCTTTTTGAATTTTACAAATCAATTAGAAAGAXXXXXXXXXXGTAGAGAGAGAAGTTGAGAAAAAGTAGTAAAAAAAAAGAAAAGAAAAGAAAAAGAAAAATGGCAGTCTGGTGAAACGGTA

>Marker202813

TTTTGAATTCAAATTCAAATTATGACTTTAATATGAATAAGATAAGATTAAGAAGAATTAGGAGACTAATGATCGGCTAAXXXXXXXXXXATGCACTTGTCCCGTTCCGTTGCCGCGTATCCCCCGAATCCAAACAAACGTGCAAGGAAGGTTTTACGGACTGAGGGTGA

TTTTGAATTGAAATTCAAATTATGACTTTAATATGAATAAGATAAGATTAAGAAGAATTAGGAGACTAATGATCGGCTAAXXXXXXXXXXATGCACTTGTCCCGTTCCGTTGCCGCGTATCCCCCGAATCCAAACAAACGTGCAAGGAAGGTTTTACGGACTGAGGGTGA

>Marker202818

ATAAAGATGAAAGGCAACGATACACAATGTAAGTCAATCTACAAAACCTAAAACTTAGGTTATGATACCAACTGTAATGAXXXXXXXXXXAGAAAGAGTGAGTATAAAAATACACAGTAAGGGACCCACTATTGGTCACGATAGGTGAATTGTTAACTTCCTGCTAAGAG

ATAAGGATGAAAGGCAACGATACACAATGTAAGTCAATCTACAAAACCTAAAACTTAGGTTATGATACCAACTGTAATGAXXXXXXXXXXGAAAAGAGTGAGTATAAAAATACATAGTAAGGGACCCACTATTGGTCACGATAGATGAATTGTTAACTTCCTGCTAAGAG

>Marker202887

AAGTGTTGTGTATTCTCTTTCAAATTTTGTGAGTATCATTGCCACCACATCTATAACGACATTAATCCCCTCAAGTCATTXXXXXXXXXXCTAAAATACCCTAATAAAACTGTTATTGGCATCAATGCTATCAATCGTCGCTATTGGGTTCGTGCTATGATGGAGGTATT

AAGTGTTGTGTATTCTCTTTCAAATTTTGTGAGTATCATTGCCACCACATCCATAACGACATTAATCCCCTCAAGTCATTXXXXXXXXXXCTAAAATACCCTAATAAAACTGTTATTGGCATCAATGCTATCAATCGTCGCTATTGGGTTCGTGCTATGATGGAGGTATT

>Marker203610

CTTTTAGGAAGCAAACCAAAACAGCTCATTAGATTGTTAGCCATTGATTGATACAAAGCATTGTTTCATGTTTCATGTTTXXXXXXXXXXTGACATTAGGAACGTGAACTGAGATCAAGAAGTAATATGTTATGCAATGGATAACCGAAAGAAACCGAAAGAAACCGCAA
[truncated: 278,755 more chars]
